# Next-Generation Phage Display: Integrating and Comparing Available Molecular Tools to Enable Cost-Effective High-Throughput Analysis

**DOI:** 10.1371/journal.pone.0008338

**Published:** 2009-12-17

**Authors:** Emmanuel Dias-Neto, Diana N. Nunes, Ricardo J. Giordano, Jessica Sun, Gregory H. Botz, Kuan Yang, João C. Setubal, Renata Pasqualini, Wadih Arap

**Affiliations:** 1 David H. Koch Center, The University of Texas M. D. Anderson Cancer Center, Houston, Texas, United States of America; 2 Department of Critical Care, The University of Texas M. D. Anderson Cancer Center, Houston, Texas, United States of America; 3 Virginia Bioinformatics Institute, Virginia Polytechnic Institute and State University, Blacksburg, Virginia, United States of America; 4 Computer Science Department, Virginia Polytechnic Institute and State University, Blacksburg, Virginia, United States of America; Instituto Butantan, Brazil

## Abstract

**Background:**

Combinatorial phage display has been used in the last 20 years in the identification of protein-ligands and protein-protein interactions, uncovering relevant molecular recognition events. Rate-limiting steps of combinatorial phage display library selection are (i) the counting of transducing units and (ii) the sequencing of the encoded displayed ligands. Here, we adapted emerging genomic technologies to minimize such challenges.

**Methodology/Principal Findings:**

We gained efficiency by applying in tandem real-time PCR for rapid quantification to enable bacteria-free phage display library screening, and added phage DNA next-generation sequencing for large-scale ligand analysis, reporting a fully integrated set of high-throughput quantitative and analytical tools. The approach is far less labor-intensive and allows rigorous quantification; for medical applications, including selections in patients, it also represents an advance for quantitative distribution analysis and ligand identification of hundreds of thousands of targeted particles from patient-derived biopsy or autopsy in a longer timeframe post library administration. Additional advantages over current methods include increased sensitivity, less variability, enhanced linearity, scalability, and accuracy at much lower cost. Sequences obtained by qPhage plus pyrosequencing were similar to a dataset produced from conventional Sanger-sequenced transducing-units (TU), with no biases due to GC content, codon usage, and amino acid or peptide frequency. These tools allow phage display selection and ligand analysis at >1,000-fold faster rate, and reduce costs ∼250-fold for generating 10^6^ ligand sequences.

**Conclusions/Significance:**

Our analyses demonstrates that whereas this approach correlates with the traditional colony-counting, it is also capable of a much larger sampling, allowing a faster, less expensive, more accurate and consistent analysis of phage enrichment. Overall, qPhage plus pyrosequencing is superior to TU-counting plus Sanger sequencing and is proposed as the method of choice over a broad range of phage display applications in vitro, in cells, and in vivo.

## Introduction

For over two decades, phage display has been used to identify relevant protein interaction and recognition sites in receptor-ligand and antigen-antibody binding systems. Because of the strong predictive value of functional relationships revealed by specific protein interactions, peptide-protein or antibody-antigen pairs selected from phage display libraries serve as potential reagents in a vast range of biomedical and translational applications [1]–[4].

A conventional phage display selection typically starts with exposure of a library to targets of interest *in vitro* or *in vivo*. After the unbound and non-specific binding populations are removed, the remaining phage particles are recovered by infection and amplification in host bacteria growing in medium under either a selective genetic pressure (such as antibiotic resistance) or a differential identifying color scheme. Host bacteria allow viral multiplication and generate thousands of newly-formed phage particles. Upon plating of the host bacteria, lysogenic phage (i.e., non-lytic M13-derived) yields bacterial colonies whereas lytic phage (i.e., Lambda-derived) generates plaques in a bacterial lawn; each resulting bacterial colony or phage plaque is considered a transducing-unit (TU). Amplified phage populations serve for additional selection round(s) and allow enrichment of selective clones, which may be determined by comparing unselected to selected libraries through DNA sequencing [1]–[4]. Thus, the two most labor- and cost-intensive steps in phage display selection are (i) the counting of transducing units and (ii) the phage DNA sequencing (from each individual colony or plaque) to determine the corresponding peptide sequence of the encoded ligand(s). This straightforward, if cumbersome, approach has long provided biomedical findings of value; however, new large-scale technologies for DNA quantification and sequencing can potentially overcome some of the practical limitations of the conventional methodology for phage display selection.

To address this prospect, we have adapted emerging genomic methodologies to eliminate these two rate-limiting steps: we used real-time PCR for rapid TU quantification and next-generation sequencing for large-scale analysis. We present a side-by-side comparison of all steps and report an integrated experimental system that greatly streamlines and expedites the entire procedure while markedly reducing its associated costs about 250-fold compared to conventional phage display selection. We show that this new methodology is not only much faster and less labor-intensive, but allows rigorous quantification with improved overall accuracy. In as-yet unpublished work, deep amplicon sequencing of phage particles (i.e., tens of thousands of clones) recovered from *in vivo* selections in human patients in a timeframe of up to 72 hours post library administration (in contrast to less than 24 hours for the current methodology), has allowed coverage of the full repertoire of targeted particles in the analyzed tissue samples, as shown by saturation curves and the identification of a panel of previously unrecognized ligand-receptor candidates. These molecular tools will enable a new generation of high-throughput and cost-effective phage display selection on an unprecedented large-scale.

## Results

### DNA-Based Analysis of Phage Quantification

Development of a high-throughput platform for phage display selection should ideally be “bacteria-free” with all required steps--including quantification, internalization, and determination of the insert sequence--to be largely based on direct nucleic acid analysis, with an expectation that it might be faster and more cost-effective than the current methodology. Thus, to validate this platform, all intermediate steps from phage quantification to sequence determination were evaluated in a direct, simultaneous (i.e., side-by-side) comparison with the conventional methodology.

**Figure 1:**
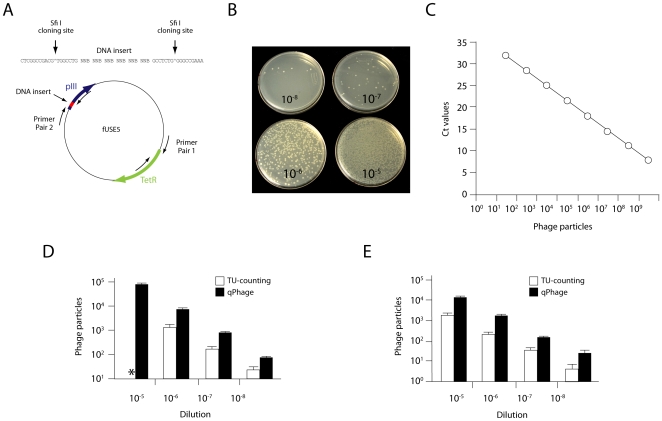
Comparison of TU-counting and qPhage. (A) Representation of phage genome and relative location of the cloning site and two sets of primers used. Primer set #1 targets the Tet^R^ gene and was used for quantification with real-time PCR; primer set #2 flanks the insert coding for the peptide displayed in pIII, and served for large-scale sequencing (B and C, respectively). TU-counting and qPhage titration output [determined by cycle-thresholds (Ct)]; note the limited quantification range for TU-counting relative to qPhage. Comparative results of TU-counting and qPhage titration of Fd-tet (D) and RGD-4C phage (E) are shown. Asterisk indicates that the high bacterial density prevented accurate TU determination.

We started by comparing the phage quantification range obtained through conventional TU-counting versus a quantitative real-time PCR-based approach (termed “qPhage”). For the qPhage methodology, we designed oligonucleotides targeting the TetR gene, a common feature to most vectors derived from fUSE5 (Fig. 1A). Both methods (TU-counting and qPhage) were used to determine phage particle content and titers in multiple serial dilutions from defined stocks. During conventional TU counting, high bacterial colony density prevented accurate determination of particles at the lower plating dilutions (Fig. 1B), a result mimicking experiments *in vivo* when high concentrations of phage are found in a targeted tissue. In contrast, the qPhage method consistently detected and linearly quantified phage content in the range of dilutions evaluated (Fig. 1C). The results of several (n = 7) independent amplifications of such phage dilutions showed a mean correlation coefficient of 0.998±0.002 and a mean amplification slope of −3.42±0.06, an amplification efficiency of ∼96%. The direct comparison of both methods, by the use of the same preparation of 4 points of 10-fold serial dilutions of an insertless phage [5] and a phage displaying an RGD-4C peptide targeting αv integrins [6]-[9], showed that qPhage is at least 5-fold more sensitive than the conventional TU-counting. This increased sensitivity is derived from the capability of the DNA-based method to quantify non-infective phage particles. When the same serial dilutions of RGD-4C phage or Fd-tet phage (Figs. 1D and 1E) were either plated or quantified by qPhage, each 10-fold dilution point resulted on average in a proportional variation of 7-fold for TU-counting and 9.3-fold for qPhage, with an SEM of the triplicates at all dilutions of 32.5% for TU-counting, versus 7.8% for qPhage. This direct comparison allowed an evaluation of both approaches in the detection of pre-determined amounts of phage particles, as well as measurement of their detection limits and linear quantification ranges. Based on these direct comparative results, we conclude that the qPhage methodology is superior and less human error-prone, particularly in high-density colony plating settings and under experimental conditions in which suitable dilutions for TU-counting and relative quantification are difficult to predict.

### Phage Binding and Internalization into Cells

**Figure 2:**
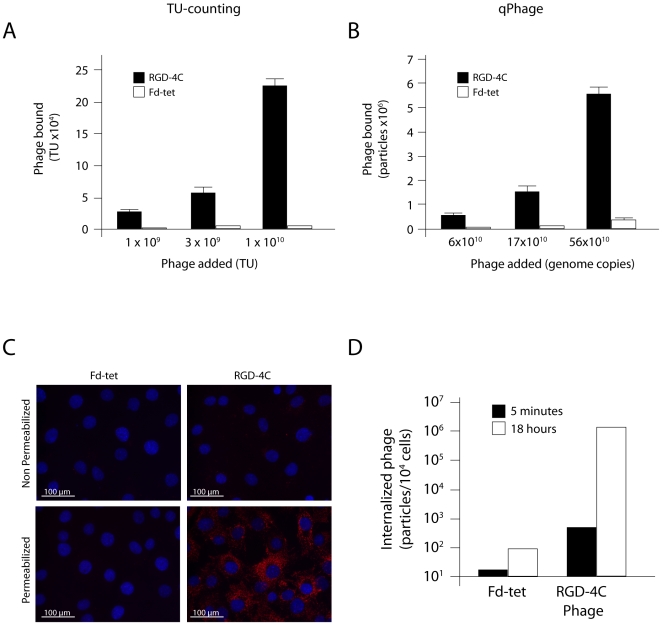
Phage binding and internalization assays. Binding of αv integrin-binding ligand phage (displaying RGD-4C) or insertless phage to endothelial cells. Quantification by conventional TU-counting (A) and qPhage (B) are shown. For internalization, endothelial cells were incubated with RGD-4C phage or insertless phage for short (5 minutes) or long (ON) incubation. Internalized phage particles were detected by immunostaining (C) or qPhage (D).

Having shown that the qPhage methodology is better than conventional TU-counting for quantification, we next sought to compare these techniques in phage display selection assays [9]–[17]. Binding of RGD-4C phage [6]–[10] or insertless control Fd-tet phage [5] to endothelial cells was performed by the Biopanning and Rapid Analysis of Selective Interactive Ligands (BRASIL) method [9] and cell-bound phage was quantified concomitantly with either TU-counting (Fig. 2A) or qPhage (Fig. 2B). Good correlation between both methods was observed, indicating that qPhage recapitulates a state-of-the-art methodology for phage-cell binding.

Another application of qPhage was the precise quantification of phage uptake in mammalian cells, thereby allowing analysis of its internalization dynamics. In the conventional methodology, phage internalization is achieved only after prolonged incubation, defined here as an overnight (ON), of phage and cells as detected by cell membrane permeabilization and visualization with anti-phage antibodies (Fig. 2C). Given the inherent technical challenges of staining-based immunodetection, this approach allows one to determine only whether or not a certain targeting peptide mediates cell internalization of a phage particle in a non-quantitative or at best, semi-quantitative manner. In contrast, an accurate quantification of phage internalization was obtained with the qPhage method. Traces of phage internalization, even after very short incubation intervals (<10 min), were detectable by qPhage but not by immunofluorescence (Fig. 2D). These results show that the targeted RGD-4C phage internalizes far more than the Fd-tet phage. Even after prolonged incubation, internalization of RGD-4C phage was 10^4^-fold higher than that of Fd-tet phage for the same period of time. The detection of internalized RGD-4C phage after the short incubation interval used was not as dramatic as that seen after ON incubation, but still points to an uptake ∼10-fold higher than that observed with insertless phage, which suggests rapid cell internalization of RGD-4C. These results indicate that internalization of peptide-guided phage can be finely quantified by qPhage. The DNA-based qPhage methodology allows full quantification of phage content, binding, and cell internalization, with a superior performance compared to conventional TU-counting.

### Next-Generation Phage Amplicon Deep Sequencing

To address the other rate-limiting step of conventional phage display, we reasoned that this DNA-based system would have to allow determination of the sequences of the encoded ligands (targeting peptides, unless otherwise specified) in large-scale. We adapted the next-generation pyrosequencing methodology (454/Roche) for phage DNA; for proof-of-principle, we used tissues obtained after intravenous administration of a phage library to end-of-life patients [18]–[20]. To determine the capacity of this approach for the high-throughput generation of phage sequences, we produced insert-containing amplicons from surgical biopsies of four human tissues (skin, white adipose tissue, bone marrow, and skeletal muscle), with oligonucleotides flanking the DNA insert coding for the peptide in the pIII gene (Fig. 1A).

In a single next-generation run, we generated a total of 319,361 sequences: 251,032 derived from tissues plus another 68,329 derived from the unselected CX7C library before administration. After filtering (see Methods), the dataset was compared to 3,840 sequences derived from the same samples by conventional Sanger-sequencing of phage recovered from host bacteria (Table 1).

**Table 1:** Sequence datasets derived from conventional and DNA-based phage display selection.

	TU-counting & Sanger Sequencing					qPhage & Next-generation sequencing					
Sample	Total raw sequences	Sequences remaining after eliminating inserts ≠21 nt (%)	Sequences remaining after eliminating non-NNB inserts (final dataset)	Distinct nucleotide sequences	Distinct peptide sequences	Total raw sequences	Sequences remaining after eliminating inserts ≠21 nt (%)	Sequences remaining after eliminating non-NNB inserts	Sequences remaining after eliminating singletons (final dataset)	Distinct nucleotide sequences	Distinct peptide sequences
**Bone marrow**	1056 (100)	979 (92.7)	953 (90.2)	555	553	55,350 (100)	39,847 (72)	37,421 (67.6)	37,049 (66.9)	2541	2539
**Fat**	672 (100)	648 (96.4)	625 (93)	357	356	56,543 (100)	46,356 (82)	43,548 (77)	43,439 (76.8)	460	459
**Muscle**	1056 (100)	1008 (95.5)	974 (92.2)	522	522	78,157 (100)	65,512 (83.8)	61,005 (81.2)	60,872 (77.9)	1014	1011
**Skin**	1056 (100)	1029 (97.4)	1002 (94.9)	496	496	60,982 (100)	49,332 (80.9)	46,166 (75.7)	46,032 (75.5)	979	976
**CX7C library**	N/A	N/A	N/A	N/A	N/A	68,329 (100)	57,089 (83.6)	49,252 (72.1)	49,252* (72.1)	44,618	44,606
**TOTAL** **	3840 (100)	3664 (95.4)	3554 (92.6)	1289	1285	319,361 (100)	258,163 (80.8)	237,392 (74.3)			

* The final dataset for the CX7C reads includes singletons.

** Not including sequences from the non-injected CX7C library for TU counting.

**Figure 3:**
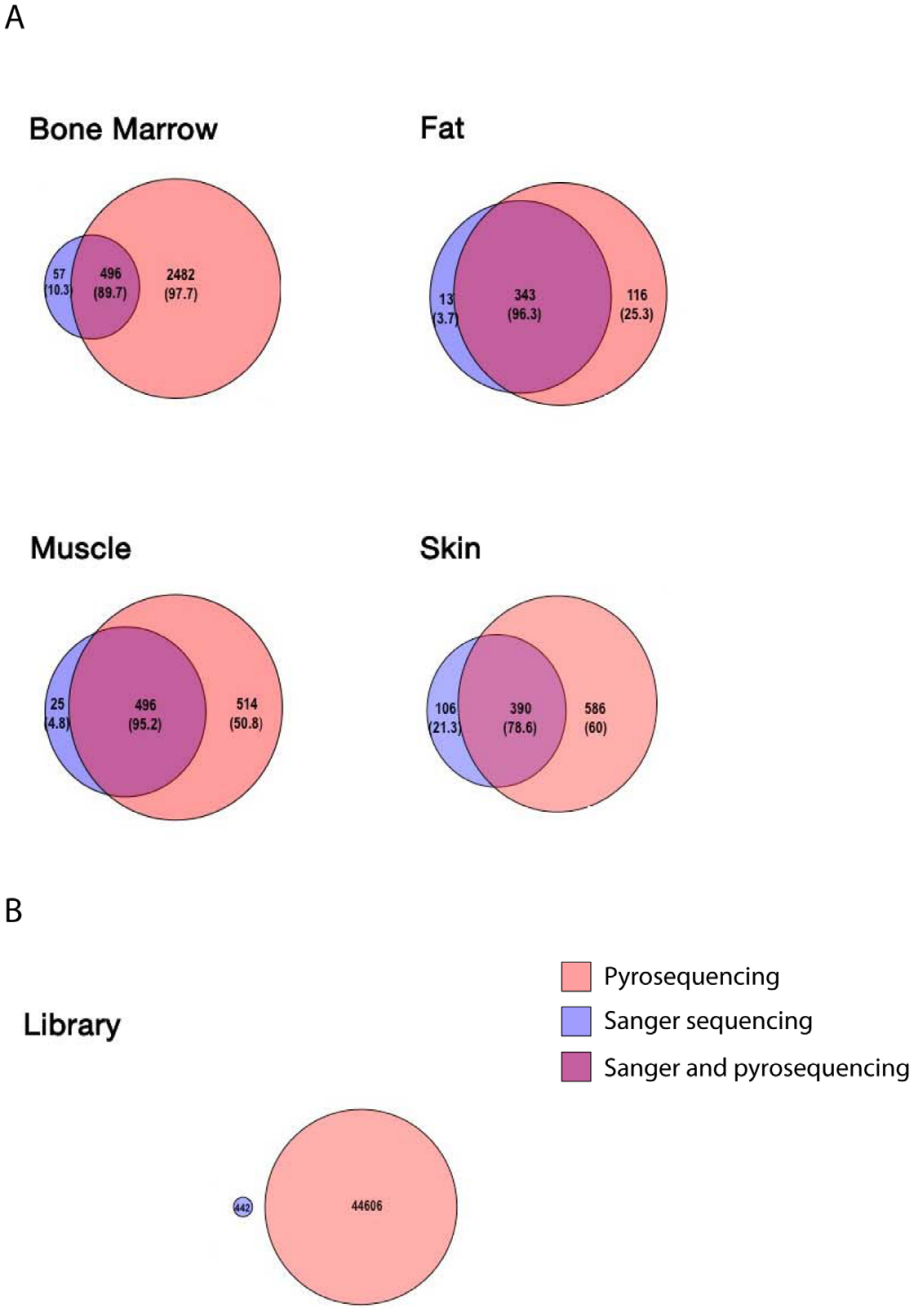
Overlap of sequences revealed by Sanger sequencing and next-generation pyrosequencing. Venn diagrams represent the peptides revealed by Sanger sequencing (purple) and large-scale next-generation pyrosequencing (salmon) in tissues such as bone marrow, fat, muscle, skin (A) or the non-selected CX7C library (B). The dark-pink area represents sequences found by both approaches. Numbers indicate the total peptides in each group and their percentage relative to TU-counting (purple and overlap areas) or relative to next-generation sequencing (salmon-colored area). No overlap was seen for the non-administered phage library sequences, produced by both methods. Circle sizes are proportional to the number of sequences revealed by each strategy.

Our initial concern was to evaluate and, if possible, rule out eventual discrepancies caused by PCR amplification as a previous step to phage amplicon deep sequencing. Thus, quality-control and quality-assurance approaches were undertaken to compare the DNA sequences derived from both methodologies, including GC content, codon-usage, and amino acid frequencies in the encoded peptides as well as the overlap of actual peptides observed by the two methods and the frequency of homopolymers in inserts derived from each dataset. After evaluating a non-redundant set of 10,983 nucleotides from Sanger sequences and 55,587 nucleotides from the pyrosequencing platform, we observed no significant differences between the two approaches, which showed respective GC contents of 60.11% and 60.19% (2-sample, unequal variance T test; t-score = 0.437, p-value ∼0.3). Strong correlations were observed for amino acid frequencies and codon utilization when 3,668 (Sanger-derived) or 18,445 (next-generation-derived) amino acid residues were compared (Pearson correlation analysis coefficients (r = 0.996, p<0.001 and r = 0.999, p<0.001, respectively), reinforcing the absence of nucleotide representation biases from DNA amplified phage (Figure S1 and Table S1). We conclude that there are no significant differences between phage DNA sequences recovered by the conventional methodology or by this alternative DNA-based approach. Moreover, when DNAs encoding peptide datasets derived from both methods were compared among human tissues (Fig. 3A) and the non-selected library (Fig. 3B), we observed that large-scale sequencing uncovered between 78.6 and 96.3% of the peptides revealed by TU-counting; whereas between 3.7 and 21.3% of the TU-derived sequences were not seen by next-generation sequencing, between 25.3 and 97.7% of the peptides revealed by deep sequencing could not be detected by TU-counting. Finally, no overlaps were seen when the non-selected phage library was sequenced by either method, a result indicating the absence of dominant clones prior to its administration (Fig. 3B).

As the next-generation DNA pyrosequencing methodology used here is known for its relatively high error-rate in DNA fragments containing homopolymers ≥5nt [21]–[23] we performed a careful evaluation of this technical issue by comparing both sequence sets (i.e., Sanger-based versus 454 pyrosquencing-based). Due to the determined length of the peptide inserts (21nt), false-positives due to sequencing errors are not expected. However, a fraction of the rejected sequences may be false-negatives (i.e., phage containing valid sequences discarded due to a wrongly assigned non-21nt insert) derived from homopolymer sequencing errors. To determine the extent of this potential effect, we investigated the frequency of homopolymer-containing sequences in both, Sanger- and 454 pyrosequencing sets. When the accepted sequence sets derived from both methods were compared (Table S4, online), we observed that the frequency of homopolymer-containing sequences to be only slightly higher (and non-statistically significant, Chi-square test; P-value = 0.9955) in the Sanger-dataset (20.4%) than in the pyrosequencing dataset (18.6%). If one assumes the percentage of 5nt-homopolymer sequences from the Sanger-set (20.4%) to be true and takes into account the known15% error rate of the pyrosequencing platform [22] one reaches an expected error rate of ∼3% (0.204×0.15) which is close to the 1.8% difference observed in the homopolymer frequency between datasets. Thus, together with other analyses presented in the remaining Supplementary Tables online, we conclude that the bias against homopolymer-containing inserts is small and non-significant in our dataset.

### Phage Diversity and Motif Enrichment

**Figure 4:**
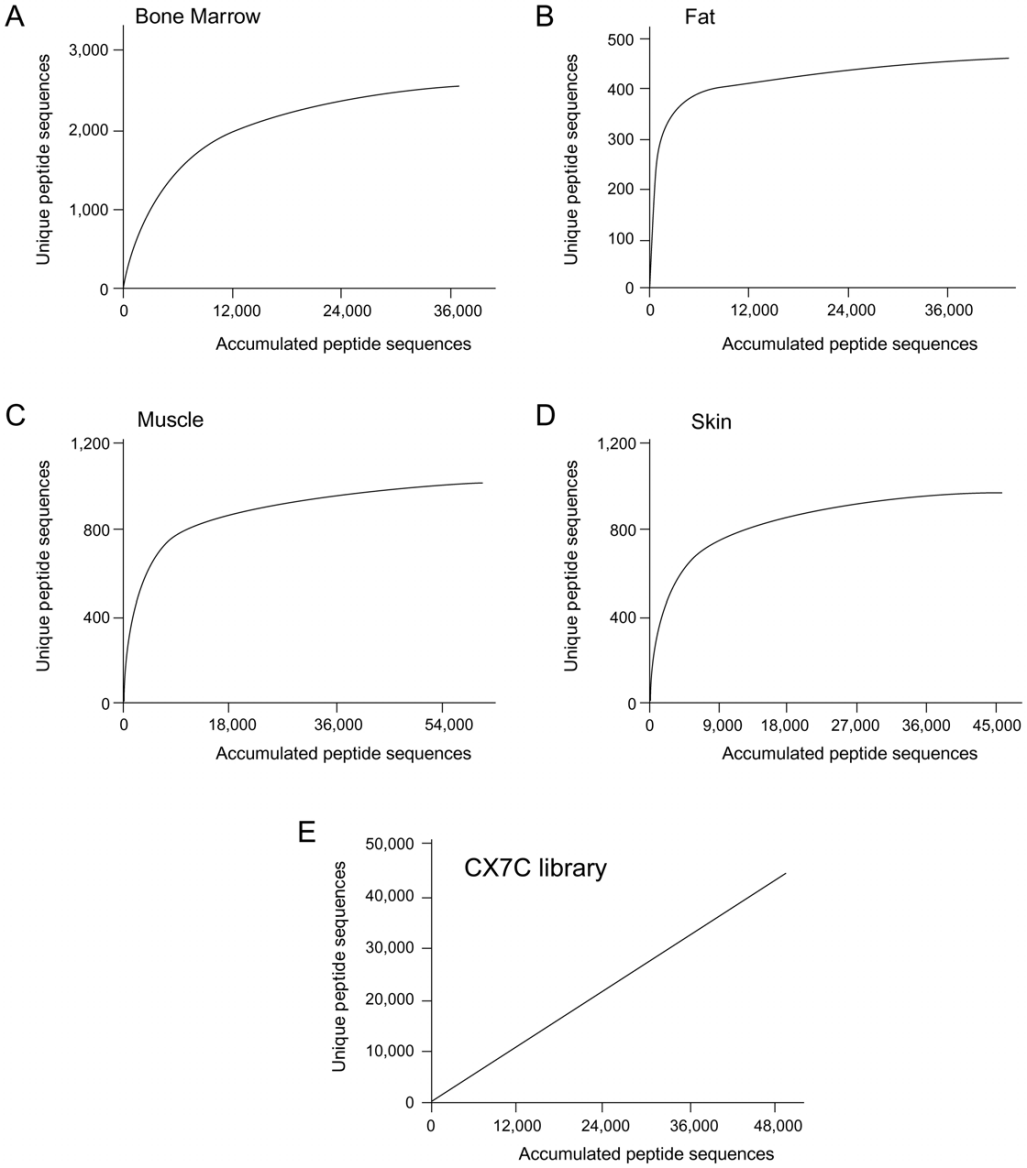
Saturation plots of peptide diversity coverage after next-generation sequencing. The plot shows the number of distinct peptides observed in bone marrow (A), fat (B), muscle (C), skin (D) or the non-selected library (E), as a function of the total number of sequences evaluated for each tissue after filtering. All tissues investigated attained or nearly attained saturation (as determined by the predicted number of distinct peptides in each tissue), whereas nothing approaching saturation was observed for the unselected library (straight line).

To determine phage diversity and candidate peptide motif enrichment, we calculated saturation curves from large-scale sequences produced from either each tissue and/or unselected library and showed that the coverage of phage particles present in these targeted tissues varied from 93.3 to 94.4% of the available predicted distinct total peptides in each tissue. The high coverage achieved for all tissues after the sequencing of 40,000 to 63,000 phage amplicons in this round of synchronous selection [29] strongly suggests that most of the diversity in these tissues has been covered (Figs. 4A–D). In striking contrast to the targeted tissues, the sequencing of the non-selected library showed its predicted diversity, which was far from saturation post evaluation of 5×10^4^ DNA sequences (Fig. 4E). Finally, we investigated whether this large dataset would allow the discrimination of more and/or longer motifs in the distinct tissues than previously reported [20]. Indeed, we observed that many more 3-mer, as well as 4-mer and 5-mer motifs, were revealed by next-generation sequencing (from 10- to 100-fold increment) in comparison to those observed in the more limited, Sanger-sequencing-derived dataset (Figure S2). These results show that DNA deep-sequencing through next-generation approaches (i) is technically feasible and (ii) offers an unprecedented high-coverage of the displayed ligand repertoire. All phage sequences produced here are available online (Phage Sequences File S1).

### A Comparative Analysis of Time- and Cost-Effectiveness

**Figure 5:**
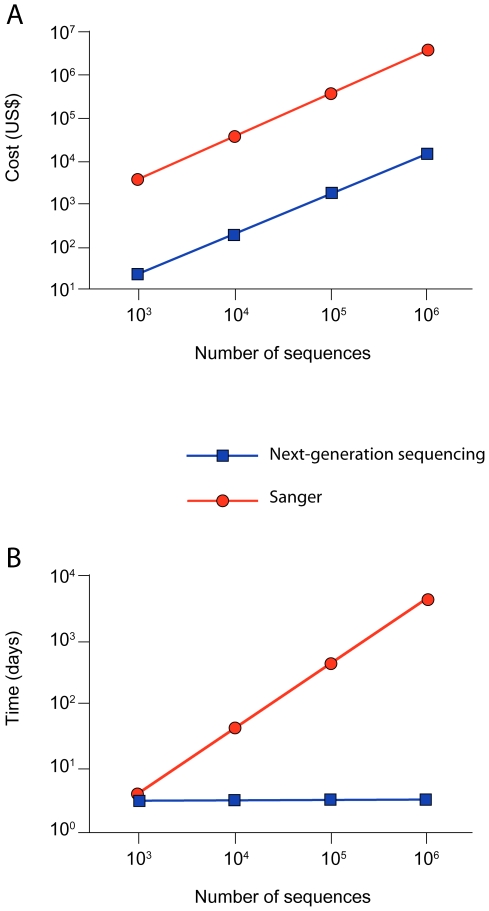
Analysis of cost and time required to generate phage sequences using Sanger- or 454-pyrosequencing methods. Cost (A) and time (B) to generate sequences with Sanger-sequencing of individual TU (red) versus DNA amplification (qPhage) followed by next-generation sequencing (blue). Data and estimates used for this analysis are presented in Table S6 online.

We performed a comparative analysis of time and cost required to reach from 10^3^ to 10^6^ sequences from a phage library by using both approaches (Table S6, online). Even with labor costs excluded from the calculation (i.e., only reagents and plastic-ware), there is a clear advantage of qPhage plus next-generation sequencing over TU-counting plus traditional Sanger-sequencing. Specifically, the generation of 10^3^ to 10^6^ sequences costs ∼250-fold less than the conventional methodology. This strong cost-effectiveness remains constant regardless of the increasing sequence number within this range (Fig. 5A). Moreover, whereas the generation of 10^3^ sequences is only 0.25-fold faster, the generation of 10^4^, 10^5^, and 10^6^ sequences will be 13-fold, 130-fold, and 1,300-fold faster, respectively. Therefore, in contrast to the high fixed-cost differential, the marked improvement in time-effectiveness becomes even more evident by the generation of larger number of sequences, which may actually be required to cover the full ligand diversity (Fig. 5B).

## Discussion

In this study, we address the two least efficient steps of phage display library selection: quantification and analysis of the displayed ligands. We introduce an integrated, robust, and readily available set of DNA-based molecular tools that will markedly improve combinatorial analysis *in vitro* and *in vivo*, and at an extremely low cost in labor and time.

Phage library quantification is currently scored through phage infection of host bacteria, serial dilution, and TU-counting (i.e., individual colony or plaque). In this process, phage recovery and library titer depend on a number of factors such as peptide-target affinity, bacterial toxicity of encoded peptides, viability of phage after panning and particle recovery, as well as inherent infection and replication properties of targeted phage clones. Indeed, certain phage encoded-peptide sequences may be non-accurately represented due to preferences in the bacteria codon-usage. Notably, molecular events of host binding, entry, and infection rates depend on the number of ligands displayed and on the nature of the hybrid fusion partner; these currently unquantifiable factors could affect infection and replication capabilities of particular phage clones and might influence the prospect of uncovering rare ligands and/or the true library size. Suggested methods, developed to overcome some of these technical challenges include bacterial infection-independent procedures, such as enzyme-linked immunosorbent assay (ELISA) with antibodies that bind specifically to the phage coat [24] and/or phage-DNA-based approaches such as quantitative-PCR [25]-[26]. However, biochemical approaches clearly still lack the required sensitivity when phage titers are low. Moreover, the concept of “high-throughput” pyrosequencing of phage display libraries has now evolved from a very complex protocol that yielded merely ∼10^2^ amplicons [27]–[28] to a far easier technique that enables the sequencing of 10^6^ amplicons as presented here. Finally, quantitative-PCR plus next-generation sequencing methodologies have not as yet been systematically compared to TU-counting plus Sanger sequencing in terms of speed, cost, and--most importantly--accuracy.

The next-generation phage display approach introduced here includes DNA-analysis of clones from the initial quantification steps to the final large-scale sequencing, which are relatively much faster and far less expensive. Replacement of bacterial overnight growth and TU-counting with DNA extraction and qPCR not only permits the quantification of non-infective/degraded phage, but it also yields phage homing results in only a few hours after tissue removal, simultaneously for dozens of samples in parallel. In real-time PCR phage quantification (qPhage), reproducible quantification was attained over a broad concentration range and was linear over at least eight orders of magnitude, far better than with the conventional approach and also much more sensitive than real-time PCR phage quantification reports [25]–[26].

Our qPhage strategy allows fast and precise validation of target tissue-specificity in the homing of selected phage or in large-scale evaluation of dozens of samples, which is particularly appealing for studies *in vivo*, including patients [29]–[30]. These goals are reachable with only small design modifications through simultaneous administration of multiple independent targeted phage particles in a single animal, followed by specific detection of each one with appropriate primers or probes in a multiplexed PCR. After homing validation, peptides shown after sequencing saturation of a number of vascular beds that appear to be specific to a particular target tissue, can be considered further as promising probes to be developed as agents for imaging and drug delivery in normal or tumor target sites.

Minor drawbacks remain. Host bacteria are still required for phage library generation and amplification between screening rounds; given the phage life cycle, this is unlikely to change. Another potential drawback is the need to re-clone the phage of interest (if desired), after its displayed peptide is determined. Nevertheless, deep sampling of the targeted peptide repertoire leads to a more reliable phage selection, and the regeneration of the selected particle(s) of interest can easily be accomplished with straightforward cloning protocols.

After titration with qPhage, the extracted DNA can be used directly for high-throughput determination of the displayed ligands (i.e., peptides or antibodies). In our tests, the generation of a large number of sequences allowed good coverage of the repertoire in all tissues studied, and included most of the sequences derived from conventional TU-counting. The availability of a larger nucleotide sequence dataset derived from high-throughput sequencing has also allowed the adoption of more stringent criteria to validate sequences. For example, we require that a peptide-encoding phage insert is accepted only if its sequence occurs at least twice, leading to the exclusion of singletons from the final dataset; such a “two-hit requirement” reduces sequencing errors in the final dataset of large-scale DNA sequencing-based approaches [31]–[32], and sharply increases our confidence in the displayed peptide list. This stringent criterion is not applicable to extremely diverse datasets in which repeats are not expected (i.e., first-round selection or library sequencing) or in reduced sequence datasets that are not large enough to cover the entire phage diversity; indeed, in such cases, the presence of sequencing errors or artifacts may be one of the factors potentially explaining why large-scale sequencing may not necessarily exhaust smaller sequence data sets.

As the new methodology proposed here is PCR-based (in contrast to a host bacteria-dependent approach), a number of potential advantages and disadvantages emerge. In general, a PCR-based approach is capable to reveal real binding peptides whose representation may be negatively impacted by the requirement of bacterial infection and multiplication. On the other hand, such PCR-based approach may acquire background noise due to errors in library construction or assembly of non-infective phage particles. Our analysis shows that most of the rejected sequences shown in Table 1 are derived from “empty” phage particles or amplification artifacts. However, the bioinformatic filters implemented here have allowed the prompt identification and discarding of these artifacts, revealing the relevant sequences in an unprecedented scale.

As the conventional phage display approach has long been validated, a central concern of this work was to evaluate whether any biases were introduced in the new steps that produced the amplicons to be sequenced. Our comparative analysis based on GC content and homopolymer frequency in the inserts, as well as codon usage, and residue or peptide frequencies and overlaps indicated that (i) there was no preferential amplification of certain inserts but (ii) both datasets share essentially the same sequence properties. As the sequencing of homopolymer-containing regions is a well-known limitation of the 454-Roche pyrosequencing platform used here, this issue was investigated in detail. As presented in the online Supplementary Tables, the rejected 454 sequence-dataset contains more homopolymers than the accepted 454 sequence-set (chi-square test, P<0.001; Table S2) and the more abundant classes of homopolymer-containing sequences (frequency >3) appear to be somewhat under-estimated (Table S3). This suggests that insert sequences containing homopolymers >5nt are under-estimated after 454-pyrosequencing. However, when all accepted sequences were evaluated (Table S4), we observed a non-statistically significant trend for a reduced frequency of homopolymers ≥5 in the 454-derived dataset when compared to the Sanger-derived sequence set (chi-square test, P = 0.9955). The fact that both datasets are similar in terms of homopolymer-containing sequences is likely due to a simple fact. After PCR, each phage is amplified generating millions of copies of the original molecule. A certain percentage (∼15%) of the homopolymer-containing amplicons, will not be correctly sequenced by 454. However, due to the massive capability of this approach, enough molecules will still be correctly sequenced and represented in the final dataset. Thus both sequence sets (454-pyrosequencing and Sanger) will be similar when the distinct sets of homopolymer sizes are considered.

To reinforce the similarity of both datasets, when sequences derived from both DNA sequencing methods (N = 1645) or sequences exclusively found by 454-pyrosequencing (N = 1202) were compared, we observe no significant differences in the frequency of homopolymers of all sizes (4 to 7 repeated bases). The comparison of homopolymer-containing inserts between these large groups and the group of sequences exclusively found by Sanger-sequencing is not informative, as it lacks precision due to its relatively small size (N = 87, compared to >1000 for the other groups). However, it is interesting to note that the frequency of homopolymers in sequences found only by the Sanger method was higher for all classes of homopolymer sizes. This effect may be real, but is certainly small as we can see from the small size of this group (Table S5).

As noted above, the sequencing of homopolymers is an established technical issue for the pyrosequencing methodology. However, from the analysis presented here, we can conclude that this technical limitation has only had a very small effect on the universe of phage particles revealed by the large-scale approach. As the 454-method allowed a high coverage of the Sanger dataset for all tissues evaluated (ranging from 78.6 to 96.3%), and it also uncovered a significant fraction of peptides (25.3 to 97.7%) not revealed by the low-throughput Sanger-sequencing approach, we conclude that the benefits of this approach certainly compensate the known disadvantages and challenges of this particular sequencing platform. As demonstrated for other platforms (such as the SOLiD, Applied Biosystems), technical alternatives exist for the particular sequencing of homopolymeric-rich regions. In the future, the integrated approach presented here may eventually be chosen for use with alternative high-throughput sequencing approaches other than the one developed by 454-Roche.

The large sequence dataset presented here has covered over 90% of the phage diversity of all human tissues we investigated and has provided a high-confidence list of tissue-specific ligands. Sets of high-confidence tissue-specific peptides along with improved statistical analysis of longer motifs can be undertaken after the sequencing of phage DNA recovered from a large number of tissues, as well as from specific tissue samples recovered by micro-dissection from paraffin-embedded tissues. In fact, large-scale sequencing of naïve (unselected and unamplified) libraries may--for the first time--provide an accurate measurement of their size (i.e., number of unique sequences), a result allowing the empiric (rather than theoretical) demonstration of the true randomness of insert sequences. In this study, it should be noted that we used targeting peptides for validation, but there is no reason that antibodies would not be as effective. Indeed, one might speculate that the DNA-based approaches introduced in this study will eliminate the need for “helper” phage for phage antibody-display selection, and finally enable its *in vivo* application.

One technical aspect merits an additional brief commentary. Next-generation sequencing approaches are being improved constantly, and the newest chemistry platforms [such as SOLiD™ (Applied Biosystems) or Illumina Genome Analyzer (Illumina, Inc.)] may actually permit the generation of >100 million sequencing “reads” per run without the inherent challenge of homopolymer sequencing of this pyrosequencing platform. For both sequencing technologies, a major limitation is the short length of possible DNA analytes (<100 nucleotides), which may prove suitable for combinatorial phage libraries (displaying small peptides). Nevertheless, because the accuracy and cost-effectiveness of such methods has not been vetted, it remains to be determined whether other massive sequencing platforms may eventually replace the platform used here.

Technological advances have already brought about a new era for genomics, epigenomics, and transcriptome studies. We predict the same will happen for phage display analysis. We show that the integration of DNA-based quantification and large-scale sequencing methodology presented here produces unbiased data and allows the full determination of the whole pool of ligand sequences available after “n” rounds of selection. Our results show that *in tandem* qPhage quantification and next-generation DNA sequencing will set a new gold standard for phage display for accuracy, running time, diversity coverage, and cost-effectiveness. Overall, the enabling platform introduced and optimized in this work is superior to TU-counting plus Sanger sequencing. As such, it may become the method-of-choice for a broad range of phage-display applications *in silico*, in cells, and *in vivo*; this will be particularly the case if the extreme molecular diversity observed during large-scale screenings in patients is considered.

## Materials and Methods

### Ethics Statement

This study design was reviewed and approved by the Institutional Review Board of the University of Texas M. D. Anderson Cancer Center and followed a pre-established ethics framework [18]–[19].

### Phage Preparation

Insertless Fd-tet [5] or RGD-4C phage [6]–[10] were amplified overnight (ON) at 37°C from a single MC1061 *E. coli* as described [1]. Phage particles were precipitated with ice-cold phosphate-buffered saline (PBS) containing 15% NaCl and PEG 8000 for 1 hour, centrifuged for 20 minutes at 4°C at 10,400 g, re-suspended in 5 ml sterile PBS, and again precipitated in ice-cold PBS containing 15% NaCl and PEG 8000 for 30 minutes. The final pellet was re-suspended in 100 µl sterile PBS and centrifuged for 2 minutes at maximum g force, and the supernatant was sterile filtered.

### Cell Binding and Internalization Assays

Bone marrow-derived endothelial cells [33] were incubated (10^5^ cells in 100 µl) with Fd-tet or RGD-4C phage (10^9^, 3×10^9^, and 1×10^10^ TU) in ice-cold DMEM containing 3% BSA for 3 hours. Cell suspensions were placed in Eppendorf tubes and centrifuged at 10,000 g for 10 minutes through an aqueous-organic interface [9], [16], [17], [29]. Cell pellets carrying membrane-bound phage were resuspended in 100 µl of PBS. Phage titers were quantified by TU-counting or qPhage.

Cells were seeded onto 12-well plates (10^5^ cells/well) ON, blocked with Dulbecco's modified Eagle's medium (DMEM) containing 30% fetal bovine serum (FBS) at 37°C and incubated with 4×10^8^ TU of phage in DMEM containing 2% FBS (400 µl of a 2×10^9^ TU/ml solution). Internalized phage were detected with a rabbit anti-bacteriophage antibody (Sigma) and Cy3-conjugated anti-rabbit antibody or were quantified by qPhage after DNA extraction.

### Patient Tissue Collection

After surrogate written informed consent was obtained from the legal next of kin [18], [19], [20], short-term intravenous infusion of phage was performed as described [20] followed by representative tissue biopsies of skin, fat-tissue, bone marrow and skeletal-muscle. Besides the screening reported here, which derived from biopsies taken from a single individual, the library was previously administered in another two patients [20] using the synchronous selection methodology [29]. Biopsy samples served for simultaneous histopathology analyses, host bacterial infection, qPhage, and next-generation sequencing.

### DNA Extraction and qPhage

DNA extractions were performed with DNeasy (Qiagen). Phage content was determined by quantitative PCR (qPCR). PCR templates consisted of 5 µl of a 1∶20 dilution of DNA, 1x Power SYBR Green PCR Master Mix (Applied Biosystems) and 3.75 pmol of each oligonucleotide primer (fUSE5F1: 5′-TGAGGTGGTATCGGCAATGA-3′ and fUSE5R1: 5′-GGATGCTGTATTTAGGCCGTTT-3′) directed to the amplification of a fragment of the Tet^R^ gene, in a final reaction volume of 15 µl. The program consisted of 50°C for 2 minutes, 95°C for 10 minutes, followed by 40 amplification cycles of 95°C for 15 seconds and 60°C for 1 minute. Standard curves were generated with serial phage dilutions (from 3 to 3^8^ plasmids) for each run. Each point of the curve and each sample DNA were amplified in triplicates. The standard curve was calculated by a linear regression analysis and serial dilutions. Amplification efficiency (AE) of each PCR cycle was calculated from the slope (*s)* of the standard curve by the equation: AE = 10^1/(−*s*)^.

### Phage DNA Amplification for Next-Generation DNA Sequencing

The amplification of the insert-containing region in the pIII gene was performed with the oligonucleotide set fUSE5454F: 5′-CGCAATTCCTTTAGTTGTTCC-3′ and fUSE5454R: 5′-TGAATTTTCTGTATGAGGTTTTGC-3′. The reaction mix consisted of 6 pmol of each primer, and 12.5 µl of the 2x Phusion hot-start high-fidelity DNA polymerase mix (Finnzymes) in 25 µl final volume. Amplifications were performed with a high-fidelity DNA polymerase mix. Cycles varied from 20- 25, and the number of reaction tubes varied from 2–6, according to the amount of phage available in each sample. Resulting amplicons (12–18 µg) served for adaptor ligation and next-generation sequencing with the FLX platform (Roche/454). All sequences described here are presented in a Supporting Information file accompanying this paper, available on line.

### Bioinformatics

DNA sequencing data were filtered to keep only sequences expected from a 21-nt inserts (termed NNB) of a CX7C library. Due to the much larger-scale nature of our sequencing approach, we adopted stringent criteria for accepting DNA sequencing reads; we applied a singleton-elimination filter where sequences that appeared only once in the whole dataset were not considered. This filter cannot be used in datasets derived from Sanger-sequencing, due to their relatively small size, or in non-selected CX7C library datasets, where only singletons are expected. Tests were performed to compare the similarity of Sanger- and next-generation sequencing-derived datasets in terms of peptide composition, GC and homopolymer content, codon usage, and residue frequency. Saturation plots were obtained by randomly shuffling the order of the filtered nucleotide or peptide sequences, and by calculating the number of distinct accumulated sequences at every 30^th^ sequence. We defined coverage as the percent ratio between the number of observed distinct peptides and the total number of predicted distinct peptides in each tissue as estimated with EstimateS [34]. Three estimators were used: abundance-based coverage estimator (ACE), incidence-based coverage estimator (ICE), and CHAO 1 mean [35]. The adopted total number of predicted distinct peptides per sample was the average of these three estimates. Data processing scripts were written in Perl v5.8 and analyses were done with The R Project for Statistical Computing R-2.6.2.

## Supporting Information

Figure S1 The amino acid frequency association between Sanger and next-generation pyrosequencing phage data was evaluated by applying a chi-square test, which indicated a significant association between both methods (p<0.001). Pearson correlation analysis indicated a strong positive correlation between sequences obtained by both approaches (r = 0.996, p<0.001).
(1.41 MB TIF)

Figure S2 Enriched motifs revealed by Sanger-sequencing and next-generation sequencing. The graph shows the number of distinct, statistically significant (Fisher's exact test, one-sided, P<0.05) tri, tetra, and penta amino acid motifs enriched in all tissues, after their frequencies were compared between every target tissue and the non-selected phage display library. Motifs derived from Sanger-sequencing are shown in the white bars.
(0.84 MB TIF)

Table S1 Codon usage: TU-counting versus pyrosequencing. The codon usage frequency between both approaches was evaluated by applying a chi-square test, which indicated a significant association between both methods (p = 0.004). Pearson correlation analysis indicated a strong positive correlation between pyrosequencing and colony-counting derived sequences (r = 0.999, p<0.001).
(0.10 MB DOC)

Table S2 Homopolymer-containing sequences in rejected- and accepted-454-pyrosequencing datasets*. *The null-hypothesis that there is no significant difference between the rejected and accepted sets in terms of the homopolymer-containing sequences that they contain can be rejected based on a Chi-square test (P-value = 0.000001). However, one should also note that the fractions for 4-mers are very close, suggesting that this effect is noticeable for k-mers with k = 5 or greater.
(0.03 MB DOC)

Table S3 Homopolymers (≥5 nt) in rare, medium, or abundant frequency groups.
(0.03 MB DOC)

Table S4 Homopolymer-containing inserts in the accepted sequence datasets*. *Chi-square test, P = 0.9955.
(0.03 MB DOC)

Table S5 Homopolymers in inserts found by each or both sequencing methods.
(0.03 MB DOC)

Table S6 Estimated time and costs required for generation of (1,000 to 1,000,000) DNA sequences with TU-counting versus pyrosequencing. * - Considering ideal bacterial densities in all plates, allowing the recovery of 250 colonies/plate and a cost of US$0.46/plate ** - Considering the availability of a dedicated DNA sequencer running 3.3 plates/day, at a cost of US$3.00/sample. *** - The time required for this step is the same for the number of sequences given in this example or for a full 454 Titanium platform (1,00,000 reads). Cost of this step is proportional to the overall cost of US$13,000/1 million reads at the DNA sequencing Core facility at The University of Texas M. D. Anderson Cancer Center.
(0.07 MB DOC)

Phage Sequences File S1 All phage sequences produced by the approach presented here are included together with the encoded peptide and the frequency of the insert for all tissues and the non-injected CX7C library.
(2.5 MB DOC)
