# Supplementary figures and images for: Next-Generation Phage Display: Integrating and Comparing Available Molecular Tools to Enable Cost-Effective High-Throughput Analysis
Source: PLoS One. 2009 Dec 17;4(12):e8338. doi: 10.1371/journal.pone.0008338 (PMC2791209; doi:10.1371/journal.pone.0008338)

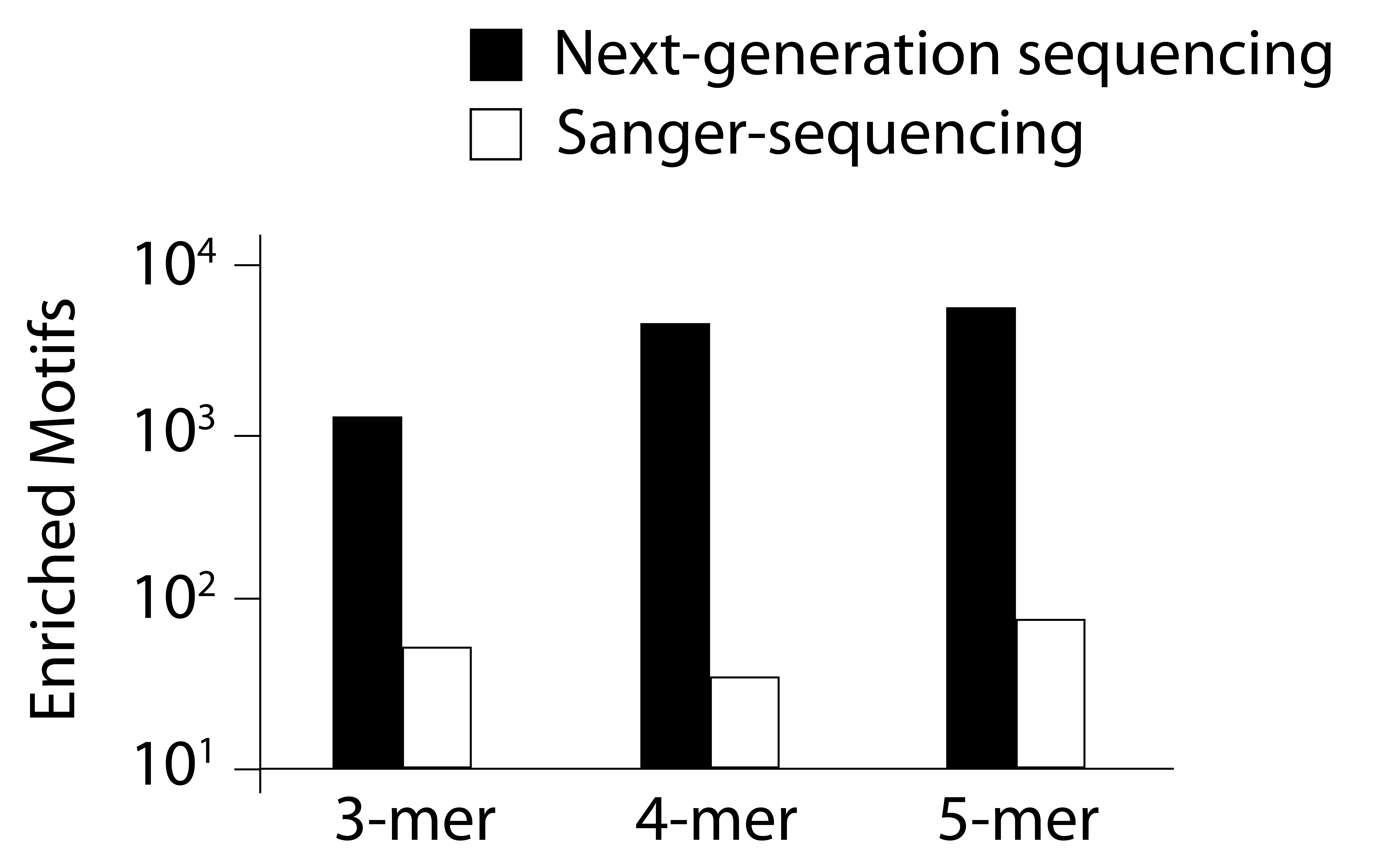

Supplement: Figure S2 — Enriched motifs revealed by Sanger-sequencing and next-generation sequencing. The graph shows the number of distinct, statistically significant (Fisher's exact test, one-sided, P<0.05) tri, tetra, and penta amino acid motifs enriched in all tissues, after their frequencies were compared between every target tissue and the non-selected phage display library. Motifs derived from Sanger-sequencing are shown in the white bars. (0.84 MB TIF) [file pone.0008338.s002.tif]
