# Supplementary material for: Next-Generation Phage Display: Integrating and Comparing Available Molecular Tools to Enable Cost-Effective High-Throughput Analysis
Source: PLoS One. 2009 Dec 17;4(12):e8338. doi: 10.1371/journal.pone.0008338 (PMC2791209; doi:10.1371/journal.pone.0008338)
Supplement: Table S1 — Codon usage: TU-counting versus pyrosequencing. The codon usage frequency between both approaches was evaluated by applying a chi-square test, which indicated a significant association between both methods (p = 0.004). Pearson correlation analysis indicated a strong positive correlation between pyrosequencing and colony-counting derived sequences (r = 0.999, p<0.001). (0.10 MB DOC) [file pone.0008338.s003.doc]

**Table S1 – Codon usage: TU-counting versus pyrosequencing**

|  | | **Next-generation Pyrosequencing** | | **Colony-counting** | |
| --- | --- | --- | --- | --- | --- |
| **Amino Acid** | **Amino Acid** | **Codon** | **Percentage(%)** | **Codon** | **Percentage(%)** |
| A | A | GCG | 60.3 | GCG | 57.9 |
| A | A | GCT | 32.8 | GCT | 35.9 |
| A | A | GCC | 6.9 | GCC | 6.2 |
| C | C | TGT | 99.5 | TGT | 100.0 |
| C | C | TGC | 0.5 | TGC | 0 |
| D | D | GAT | 83.7 | GAT | 87.2 |
| D | D | GAC | 16.3 | GAC | 12.8 |
| E | E | GAG | 100.0 | GAG | 100.0 |
| F | F | TTT | 81.9 | TTT | 87.3 |
| F | F | TTC | 18.1 | TTC | 12.7 |
| G | G | GGG | 55.5 | GGG | 56.0 |
| G | G | GGT | 35.9 | GGT | 37.8 |
| G | G | GGC | 8.6 | GGC | 6.2 |
| H | H | CAT | 84.1 | CAT | 88.2 |
| H | H | CAC | 15.9 | CAC | 11.8 |
| I | I | ATT | 83.7 | ATT | 88.8 |
| I | I | ATC | 16.3 | ATC | 11.2 |
| K | K | AAG | 100.0 | AAG | 100.0 |
| L | L | TTG | 43.8 | TTG | 42.8 |
| L | L | CTG | 29.8 | CTG | 32.2 |
| L | L | CTT | 20.8 | CTT | 19.9 |
| L | L | CTC | 5.6 | CTC | 5.1 |
| M | M | ATG | 100.0 | ATG | 100.0 |
| N | N | AAT | 84.9 | AAT | 91.7 |
| N | N | AAC | 15.1 | AAC | 8.3 |
| P | P | CCG | 54.4 | CCG | 55.0 |
| P | P | CCT | 37.3 | CCT | 38.3 |
| P | P | CCC | 8.3 | CCC | 6.7 |
| Q | Q | CAG | 100.0 | CAG | 100.0 |
| R | R | CGG | 35.9 | CGG | 36.5 |
| R | R | AGG | 34.7 | AGG | 36.0 |
| R | R | CGT | 23.9 | CGT | 23.6 |
| R | R | CGC | 5.5 | CGC | 3.9 |
| S | S | TCG | 33.7 | AGT | 33.4 |
| S | S | AGT | 32.7 | TCG | 32.5 |
| S | S | TCT | 22.1 | TCT | 23.6 |
| S | S | AGC | 6.6 | AGC | 6.2 |
| S | S | TCC | 4.8 | TCC | 4.3 |
| T | T | ACG | 55.9 | ACG | 56.2 |
| T | T | ACT | 37.8 | ACT | 38.3 |
| T | T | ACC | 6.3 | ACC | 5.5 |
| V | V | GTG | 54.7 | GTG | 55.3 |
| V | V | GTT | 37.9 | GTT | 39.4 |
| V | V | GTC | 7.4 | GTC | 5.3 |
| W | W | TGG | 100.0 | TGG | 100.0 |
| Y | Y | TAT | 85.7 | TAT | 87.4 |
| Y | Y | TAC | 14.3 | TAC | 12.6 |
