# Supplementary material for: Next-Generation Phage Display: Integrating and Comparing Available Molecular Tools to Enable Cost-Effective High-Throughput Analysis
Source: PLoS One. 2009 Dec 17;4(12):e8338. doi: 10.1371/journal.pone.0008338 (PMC2791209; doi:10.1371/journal.pone.0008338)
Supplement: Table S2 — Homopolymer-containing sequences in rejected- and accepted-454-pyrosequencing datasets*. *The null-hypothesis that there is no significant difference between the rejected and accepted sets in terms of the homopolymer-containing sequences that they contain can be rejected based on a Chi-square test (P-value = 0.000001). However, one should also note that the fractions for 4-mers are very close, suggesting that this effect is noticeable for k-mers with k = 5 or greater. (0.03 MB DOC) [file pone.0008338.s004.doc]

**Table S2 - Homopolymer-containing sequences in rejected- and accepted-454-pyrosequencing datasets***

| **Homopolymer size (nt)** | **454-sequences** | |
| --- | --- | --- |
|  | **Rejected set** | **Accepted set** |
| 4 | 1128/3826 (29.5%) | 813/2847 (28.6%) |
| 5 | 821/3826 (21.5%) | 337/2847 (11.8%) |
| 6 | 459/3826 (12.0%) | 128/2847 (4.5%) |
| 7 | 184/3826 (4.8%) | 49/2847 (1.7%) |
| 8 | 94/3826 (2.5%) | 16/2847 (0.6%) |
| ≥4 | 2686/3826 (70.2%) | 1343/2847 (47.2%) |
| ≥5 | 1558/3826 (40.7%) | 530/2847 (18.6%) |
