# Supplementary material for: Next-Generation Phage Display: Integrating and Comparing Available Molecular Tools to Enable Cost-Effective High-Throughput Analysis
Source: PLoS One. 2009 Dec 17;4(12):e8338. doi: 10.1371/journal.pone.0008338 (PMC2791209; doi:10.1371/journal.pone.0008338)
Supplement: Table S3 — Homopolymers (≥5 nt) in rare, medium, or abundant frequency groups. (0.03 MB DOC) [file pone.0008338.s005.doc]

**Table S3. Homopolymers (≥5 nt) in rare, medium, or abundant frequency groups**

| **Frequency groups** | **Accepted sequences** | |
| --- | --- | --- |
|  | **454-pyrosequencing** | **Sanger-sequencing** |
| Rare (1-2) | 582/3074 (18.9%) | 240/1296 (18.5%) |
| Medium (3-10) | 1527/8650 (17.6%) | 297/1070 (27.8%) |
| Abundant (>10) | 15024/175717 (8.55%) | 364/1188 (30.6%) |
