# Supplementary material for: Next-Generation Phage Display: Integrating and Comparing Available Molecular Tools to Enable Cost-Effective High-Throughput Analysis
Source: PLoS One. 2009 Dec 17;4(12):e8338. doi: 10.1371/journal.pone.0008338 (PMC2791209; doi:10.1371/journal.pone.0008338)
Supplement: Table S4 — Homopolymer-containing inserts in the accepted sequence datasets*. *Chi-square test, P = 0.9955. (0.03 MB DOC) [file pone.0008338.s006.doc]

**Table S4 - Homopolymer-containing inserts in the accepted sequence datasets***

| **Homopolymer size (nt)** | **Accepted sequences** | |
| --- | --- | --- |
|  | **454-Pyrosequencing set** | **Sanger-sequencing set** |
| 4 | 813/2847 (28.6%) | 371/1289 (28.8%) |
| 5 | 337/2847 (11.8%) | 159/1289 (12.3%) |
| 6 | 128/2847 (4.5%) | 65/1289 (5.0%) |
| 7 | 49/2847 (1.7%) | 24/1289 (1.9%) |
| 8 | 16/2847 (0.6%) | 15/1289 (1.1%) |
| ≥4 | 1343/2847 (47.2%) | 634/1289 (49.2%) |
| ≥5 | 530/2847 (18.6%) | 263/1289 (20.4%) |
