# Supplementary material for: Next-Generation Phage Display: Integrating and Comparing Available Molecular Tools to Enable Cost-Effective High-Throughput Analysis
Source: PLoS One. 2009 Dec 17;4(12):e8338. doi: 10.1371/journal.pone.0008338 (PMC2791209; doi:10.1371/journal.pone.0008338)
Supplement: Table S5 — Homopolymers in inserts found by each or both sequencing methods. (0.03 MB DOC) [file pone.0008338.s007.doc]

**Table S5 - Homopolymers in inserts found by each or both sequencing methods**

| **Homopolymer size (nt)** | **Sanger only**  **(N=87)** | **454-pyrosequencing only**  **(N=1645)** | **Sanger and 454-pyrosequencing**  **(N=1202)** |
| --- | --- | --- | --- |
| 4 | 18/87 (20.7%) | 460/1645 (28.0%) | 353/1202 (29.4%) |
| 5 | 18/87 (20.7%) | 196/1645 (11.9%) | 141/1202 (11.7%) |
| 6 | 9/87 (10.3%) | 72/1645 (4.3%) | 56/1202 (4.6%) |
| 7 | 5/87 (5.7%) | 30/1645 (1.8%) | 19/1202 (1.6%) |
| 8 | 8/87 (0.9%) | 9/1645 (0.6%) | 7/1202 (0.6%) |
| ≥4 | 58/87 (66.7%) | 767/1645 (46.6%) | 576/1202 (47.9%) |
| ≥5 | 40/87 (46.0%) | 307/1645 (18.7%) | 223/1202 (18.6%) |
