# Supplementary material for: Next-Generation Phage Display: Integrating and Comparing Available Molecular Tools to Enable Cost-Effective High-Throughput Analysis
Source: PLoS One. 2009 Dec 17;4(12):e8338. doi: 10.1371/journal.pone.0008338 (PMC2791209; doi:10.1371/journal.pone.0008338)
Supplement: Table S6 — Estimated time and costs required for generation of (1,000 to 1,000,000) DNA sequences with TU-counting versus pyrosequencing. * - Considering ideal bacterial densities in all plates, allowing the recovery of 250 colonies/plate and a cost of US$0.46/plate ** - Considering the availability of a dedicated DNA sequencer running 3.3 plates/day, at a cost of US$3.00/sample. *** - The time required for this step is the same for the number of sequences given in this example or for a full 454 Titanium platform (1,00,000 reads). Cost of this step is proportional to the overall cost of US$13,000/1 million reads at the DNA sequencing Core facility at The University of Texas M. D. Anderson Cancer Center. (0.07 MB DOC) [file pone.0008338.s008.doc]

**Table S6 – Estimated time and costs required for generation of**

**(1,000 to 1,000,000) DNA sequences with TU-counting versus pyrosequencing**

| **TU-counts** | | |  | **454-Pyrosequencing** | | |
| --- | --- | --- | --- | --- | --- | --- |
| **To reach 1,000 sequences** | | | | | | |
| **Activity** | **Time required** | **Cost (US$)** |  | **Activity** | **Time required** | **Cost (US$)** |
| **K91 infection, plating and colony growth*** | 16h | 1.84 |  | **DNA extraction** | 1h | 2.2 |
| **Colony picking** | 2.5h | 0 |  | **PCR** | 45min | 2.3 |
| **PCR** | 24h | 385.00 |  | **Gel analysis and**  **sample concentration** | 45min | 1.75 |
| **Gel electrophoresis** | 3h | 50 |  | **DNA quantification** | 20min | 1.00 |
| **DNA sequencing**** | 72h | 3,000 |  | **Ligation of adapters and DNA sequencing**** | 72h | 13.00 |
| **TOTAL** | 93.5h (3.9 days) | 3,436.84 |  | **TOTAL** | 74.8h | 20.25 |
| **To reach 10,000 sequences** | | | | | | |
| **Activity** | **Time required** | **Cost (US$)** |  | **Activity** | **Time required** | **Cost (US$)** |
| **K91 infection, plating and colony growth*** | 24h | 18.4 |  | **DNA extraction** | 1h | 2.2 |
| **Colony picking** | 25h | 0 |  | **PCR** | 45min | 2.3 |
| **PCR** | 240h | 3850 |  | **Gel analysis and**  **sample concentration** | 45min | 1.75 |
| **Gel electrophoresis** | 6h | 100 |  | **DNA quantification** | 20min | 1.00 |
| **DNA sequencing**** | 720h | 30,000 |  | **Ligation of adapters and DNA sequencing**** | 72h | 130.00 |
| **TOTAL** | 1,015h  (42 days) | 33,968.4 |  | **TOTAL** | 74.8h | 137.25 |
| **To reach 100,000 sequences** | | | | | | |
| **Activity** | **Time required** | **Cost (US$)** |  | **Activity** | **Time required** | **Cost (US$)** |
| **K91 infection, plating and colony growth*** | 36h | 184 |  | **DNA extraction** | 1h | 2.2 |
| **Colony picking** | 250h | 0 |  | **PCR** | 45min | 2.3 |
| **PCR** | 2400h | 38,500 |  | **Gel analysis and**  **sample concentration** | 45min | 1.75 |
| **Gel electrophoresis** | 12h | 200 |  | **DNA quantification** | 20min | 1.00 |
| **DNA sequencing**** | 7200h | 300,000 |  | **Ligation of adapters and DNA sequencing**** | 72h | 1300 |
| **TOTAL** | 9,898h  (412 days) | 338,884 |  | **TOTAL** | 74.8h | 1307.25 |
| **To reach 1,000,000 sequences** | | | | | | |
| **Activity** | **Time required** | **Cost (US$)** |  | **Activity** | **Time required** | **Cost (US$)** |
| **K91 infection, plating and colony growth** | 54h | 1,840 |  | **DNA extraction** | 1h | 2.2 |
| **Colony picking** | 2500h | 0 |  | **PCR** | 45min | 2.3 |
| **PCR** | 24000h | 385,000 |  | **Gel analysis and**  **sample concentration** | 45min | 1.75 |
| **Gel electrophoresis** | 24h | 400 |  | **DNA quantification** | 20min | 1.00 |
| **DNA sequencing**** | 72,000h | 3,000,000 |  | **Ligation of adapters and DNA sequencing***** | 72h | 1200.00 |
| **TOTAL** | 98,578h  (4,106 days) | 3,387,240 |  | **TOTAL** | 74.8h | 13007.25 |
