# Supplementary material for: Next-Generation Phage Display: Integrating and Comparing Available Molecular Tools to Enable Cost-Effective High-Throughput Analysis
Source: PLoS One. 2009 Dec 17;4(12):e8338. doi: 10.1371/journal.pone.0008338 (PMC2791209; doi:10.1371/journal.pone.0008338)
Supplement: Phage Sequences File S1 — All phage sequences produced by the approach presented here are included together with the encoded peptide and the frequency of the insert for all tissues and the non-injected CX7C library. (2.5 MB DOC) [file pone.0008338.s009.doc]

start non-injected CX7C library

GTGAGGAGTGTTGCTTTGGCT VRSVALA 1

GGTACTTTTCTCTCGAAGCAG GTFLSKQ 2

CTGCGCGAGTTTTGGTGGGGG LREFWWG 1

CATCCGTTGTCGTGTTTGGCT HPLSCLA 1

TGCTGCCGCACCCTTTCGATG CCRTLSM 1

CTTAGCGGCGCTTGTTGCAGT LSGACCS 1

CTTTGGCTGCGGATGGACTCG LWLRMDS 1

GGGAGCGTGTGCTCTATGCTG GSVCSML 1

AGCGTTCGCTGGGTTGAGAAG SVRWVEK 1

GTTTCTTTGCGTTGCGGTAGT VSLRCGS 1

TGGCGTTGTGTTGTGGTGCGG WRCVVVR 1

CCGTGGTCGAAGCAGTCCGTC PWSKQSV 1

TGGGGCGGGCCCGTCGAGCTT WGGPVEL 1

GTGCCTCGGTACCTCAAGGAG VPRYLKE 1

CCCTTTAGGGGTCGTCTGGTG PFRGRLV 1

CGCCAGCCCACGTGGGCTGTC RQPTWAV 1

AGTCGTGGCCGCGTTGTGTGG SRGRVVW 2

GCCCCGGCTAGTTGCGTGGGG APASCVG 1

TATTGCGGGGGCCTGGTCGCT YCGGLVA 1

GCGGACTATGCCTGCTACTGT ADYACYC 1

GATCTGGCGCTTCCCAATCAT DLALPNH 1

GTTTTCATCATTCGTAAGTCT VFIIRKS 1

CGTGAGGTCGGCTACATTCAG REVGYIQ 2

TCCACGTTTCGTGCCCTCGAT STFRALD 1

GTGTTGCCCGAGGTCCAGCGT VLPEVQR 1

GGCACCGGCCGCCATTTGTTC GTGRHLF 1

GCTGAGTGGCTTGACGGCATT AEWLDGI 1

CGTACGGTCGGTAGCGATGAG RTVGSDE 1

TGTCTGGCTTCTTGGCGCCCG CLASWRP 1

CCCAGCGTCTTCCGGGTTGTG PSVFRVV 1

TGCTGCATGCGGCAGGCGTGG CCMRQAW 1

GACTACTGCCTGCTGAACGGG DYCLLNG 1

ATCTATCGCTGGGGCGTTCCT IYRWGVP 1

TTGGCTCGCGGGTTGCCGCCC LARGLPP 1

CTGTGGTCGGTGCTTGATAGG LWSVLDR 1

TATACCCTGTTGGGGTTGCCT YTLLGLP 1

GCCGATTTGTGGTGGGCGACC ADLWWAT 1

CAGCATGACCCGAAGTCGCTT QHDPKSL 1

TTCAAGGCGGTCGGTAAGCCC FKAVGKP 1

ATGGTGAGGCGGGCTTTGGTT MVRRALV 1

TGGGTGATGAATGTCGGTATG WVMNVGM 1

GGGCATTTCCGGAGGGCTAAT GHFRRAN 1

CAGCTTCACACGTGGGTTGAG QLHTWVE 1

CTGGGTGGCTCGCCTGTGCGG LGGSPVR 1

GGGCCCGCTGTTTGTTGCATT GPAVCCI 1

TGTTACACCATTGTGATGTGC CYTIVMC 1

CGGGTTGATATTTGCAATTAC RVDICNY 1

GCCGCTTTCTCTCTGCTGAGT AAFSLLS 1

CTGGTCTTCATGGACTACCCT LVFMDYP 1

CACAGGTTCGCCTCTCGCAAC HRFASRN 1

TTCGTGGTGGAGTTGATGTCG FVVELMS 1

GTCCGCCCTAGCATCTCCCGT VRPSISR 2

TCGGTGAACAGGAATGGCTGG SVNRNGW 1

CGCTCTGAGGCGGTCGCGTTG RSEAVAL 2

GGGTTGGCTGGCACGTGTGCG GLAGTCA 2

AACCAGCACGGTTGCAGGTCT NQHGCRS 1

AACTCCCGCACGCGTGGTATT NSRTRGI 1

GCTTACATGGCGAGCGCCCCT AYMASAP 1

CTGTCTCAGTTGTATTTGGAT LSQLYLD 1

CTGTGTGACCCGGTGGACGAT LCDPVDD 1

GCCCCGCACCGGGGGTCTGTG APHRGSV 1

GACACGTGGACTTTGGGGCCG DTWTLGP 1

ACGGTGGACTGGGCGGGCTCG TVDWAGS 1

TGTCCCGTGCTTCAGTGCTCG CPVLQCS 1

GGGCAGTCGCGCATGTCGGGT GQSRMSG 1

ATGATCGTGCACGCGGCCTGT MIVHAAC 1

GGCTTGGACCTCTATTTGCGT GLDLYLR 1

ATGTGGGAGGCCGCGTGGCGT MWEAAWR 1

TTTGTGTTCGATGTCAGGACC FVFDVRT 1

ATTTTCTTGGCTGTTTTTACG IFLAVFT 1

GTCGAGCCGTGGCGTGTTAGT VEPWRVS 1

AGGGGGGCGGTGTTGTCGCGG RGAVLSR 1

AATTGCATCCATTTGGACACT NCIHLDT 1

GCCAGCTGGCACCAGAATTAT ASWHQNY 1

CACCCGCTCAGCGGTAGGAGG HPLSGRR 1

GGGGACTTGGCTTTCATCAAG GDLAFIK 1

TGGAGGTTCACCGCCTTCGCT WRFTAFA 1

GGGATCAAGCCCATCTATCCG GIKPIYP 1

GCTGTTGGGCCGGATTACCTG AVGPDYL 2

TGGGTGCTGTTTTCGGAGTTT WVLFSEF 1

GGGAGCGGTGGCCGGATGGTG GSGGRMV 1

GTTATGATCAGTCACGTGTCT VMISHVS 1

TCTGCGGGGGTGCGGGGGTCG SAGVRGS 1

TCTGGGGCTATCGGTGTGCAT SGAIGVH 1

GCCTCGGTGCTCGCCGAGGAT ASVLAED 1

CTGTGGTCGTTGGATGTTAAT LWSLDVN 1

GGGGCTCTGCTCCGCTGGCCT GALLRWP 1

GCTGGGTTGGCCAATTGCACT AGLANCT 1

CCCGGGGTCGGCCTGTCCGTG PGVGLSV 1

AGTGGCATCAGGCGTACTTCT SGIRRTS 1

TGTTGCCGGGCCTCCGCCTAT CCRASAY 1

ACGATGCAGGGCTGCTTGTTT TMQGCLF 1

ATTTGCTACGGGATGGGTGCT ICYGMGA 1

CGGTCGTTGTACGCCGCTGGC RSLYAAG 1

ATCGGTTTCAGGGTCAGGAGT IGFRVRS 2

GTCCCGGCCAGGCGGCGGTTG VPARRRL 1

GCTGTCAGTACCTTGTTGCGT AVSTLLR 1

TCCTATCCGGCGGGTAGTCGG SYPAGSR 1

CGCTTTGGCATGCGCTATTGT RFGMRYC 1

CGTTGCGTCGCCTGCCACTCT RCVACHS 1

TGTTTGGCTCCCTTCCACCCT CLAPFHP 1

TACAGGCTCGGGTTCGGGTCT YRLGFGS 1

TGTGCTGCTCCGGGCCGGGGG CAAPGRG 2

GGGGGGCATGCGGTCGGGACC GGHAVGT 1

GGTTGGCGTGAGTCGTATGTT GWRESYV 1

GAGCACATTGGCTGGTACGAG EHIGWYE 1

GTGCGCGGGGCGGGCTTGACT VRGAGLT 1

GATGTCTGGTCCAGCGGCTTT DVWSSGF 1

TGTTTGGCGTCGCGGGGTTGT CLASRGC 3

TGCCACTTCAAGGTCCGTTCC CHFKVRS 3

GCCTGGCGGTACTCGCGTACT AWRYSRT 1

TTTTATAGCAGCTTGGGGCAG FYSSLGQ 1

CGTGGGCACTACGTCGTGAGG RGHYVVR 1

TGGTCTGATGTGGATCACAAT WSDVDHN 1

GTCTACGCCGTCGGGCTTTGG VYAVGLW 1

TCCAAGGGCGTCGAGTCCTTG SKGVESL 1

GAGTCCCGGGTTTACCTGGCC ESRVYLA 1

TGGAGGAGTTCGTGGGAGTTG WRSSWEL 1

GGCGAGGACTGCACGGTGTCT GEDCTVS 1

TCCGGTGGCCCGCACGTCCCT SGGPHVP 1

ACTATTCTGGACCGGGGTATT TILDRGI 1

CTGGGCCGGCGTACCGGGCCG LGRRTGP 1

GTTGGTCAGAGCTGGTCGCAT VGQSWSH 1

GGCTCCGGCAGCGGCGGGGCT GSGSGGA 1

AGGGGTACGGGCATGCAGGTT RGTGMQV 1

TTTTGCCGGAGCGGCATGTCT FCRSGMS 1

TACAGCGACGGGTGGCGTCTT YSDGWRL 1

ATCTTTACCGGGCGTATCGGT IFTGRIG 1

CGCGCTAGTAGTCCCAGTTCC RASSPSS 1

AGCGGTGGCCCCCGGCGTCCT SGGPRRP 1

TTCGGGACTGTCCGCGCGTCG FGTVRAS 1

TGTGCGCCCAGGGCCTCGTTG CAPRASL 1

GTGTGGGCCGGTGTCCTGAAG VWAGVLK 1

ATCCTTGTCTGGTCTCGGGAT ILVWSRD 1

CTCTATCTGTTGGATCGTCGC LYLLDRR 1

TACATCATCAGGGCCGTTAGC YIIRAVS 1

ATGCCCTCTTTCTTGACCCGC MPSFLTR 1

CGGTTGGAGGACCGGCGCACT RLEDRRT 1

GGTTGCGCTGTTCGCAATTAT GCAVRNY 1

CTTGAGTGCGGGGGTGGTGCT LECGGGA 1

GCTTGCAAGGGTGTTGTTGCT ACKGVVA 2

CCTCTTAGTCGCCAGTTCCGT PLSRQFR 1

GGGTGCTACTTTCCCGTGGCG GCYFPVA 1

TTCTTGCCTCGTTCTTCGAGC FLPRSSS 1

CGGAGTTGGGTGATCTTGTTT RSWVILF 1

ATTTCGCAGGATTTGGCGTGG ISQDLAW 1

TACGCTGAGTCGACTGCTATG YAESTAM 1

AAGATGTGGGGTTTTCTCCAC KMWGFLH 4

TTTGGGCTTTGCAGTCGGGGT FGLCSRG 1

AGTTGCTTCGGTGGCCGGGAG SCFGGRE 1

CACTGGCGGAGCGCTTTGGTT HWRSALV 1

AATTGTTTGCCGATCGGGTTT NCLPIGF 2

ACTCAGCGCAGTGAGCTCCAG TQRSELQ 1

AGTGAGCCTGATGGCCTGGGC SEPDGLG 1

GTCCTTAGTACTGGCTTGGGT VLSTGLG 1

ACGGGGGTGAACCCGGGCGTG TGVNPGV 1

GCCTTCTATCCCCCTGTTGCG AFYPPVA 1

GTGCTGGTTATCATGACTGAT VLVIMTD 1

CGGGATTGTTGGCGCTCCTGT RDCWRSC 1

GGTCTGGTCCTGCTGCCCCTT GLVLLPL 1

TTCATCGGTTGCTGGTCTACT FIGCWST 2

CCCATTCAGCGGGATGATGAG PIQRDDE 1

GTCTGCATGGCGAGGCGGTGT VCMARRC 1

TTCTTCCTCCCGTCCGGTACT FFLPSGT 1

GGGGGCCGTCCGACCGCTCTG GGRPTAL 2

GATTGTGGGGGGCGGTGCCGG DCGGRCR 1

TCCGTTGTGCGTGATGACCAG SVVRDDQ 1

TGCGGGGACGCGTCTGCTTCT CGDASAS 1

TTCAGTCTGGGTGAGGATCCT FSLGEDP 1

CGGTGGGATGTCCTGTTTTCG RWDVLFS 1

ACTGCGAGGGCGTGGAAGGTG TARAWKV 1

GGTGTGTCTTGCGCCGGTGGG GVSCAGG 1

GGTAAGTACCGCAGCTGTATG GKYRSCM 1

GCGCCTGGCATTGTGTGGCTG APGIVWL 1

CTCCTGTTTGAGAGGCCTTCC LLFERPS 1

CGGTGCGGCGTTCGCGGTGTG RCGVRGV 1

GACTTGTGCGTGGCTGCCTGC DLCVAAC 1

TGTGCCCGCTCCGCCCATGTG CARSAHV 1

GGCGTGCAGCTCCACGGTCAT GVQLHGH 1

CGGGTTGCGCGGATCGGGTGG RVARIGW 1

GGGCAGCCTCCTAGCGCGGCT GQPPSAA 1

CGGTTTTATGCGTGCGGTCGT RFYACGR 1

ATGGTCCCGAATCACGAGGAT MVPNHED 2

GGTCGGTGGATGTGTTTTCGG GRWMCFR 1

GGCTTGCCTCTGAAGCACATT GLPLKHI 1

CGCGGGATCGGGTGCGGCATT RGIGCGI 1

CTTATGGGCTTTAGGAACGCT LMGFRNA 1

ATCCACCTCGGCCTCAGGGGT IHLGLRG 1

CACCAGTGCAGGCTCTGGTGT HQCRLWC 1

ATGGGGCACCTCGGTTCCGGT MGHLGSG 2

TGTTGGAACCGTTGCAGGGGG CWNRCRG 1

TGGTGCGGCAACGCGGGCTCT WCGNAGS 1

AATGTTTCTCGCGTGGGGTGC NVSRVGC 1

CCGTGCGAGATCGCCAAGGGG PCEIAKG 1

GGGGGCGGTAAGACGGCTCGC GGGKTAR 1

ACCAATGATCTCTGGATGGCG TNDLWMA 1

TGTCGGTGTAGGAGTGTGCGT CRCRSVR 1

TTGAGTCGGGGGCCCGTCGCG LSRGPVA 1

GTGTGGTCCTCCACTTTGCGT VWSSTLR 1

TTCATCAACATTTGGGCGTGT FINIWAC 1

TATGTCAATGACTTCGAGTGG YVNDFEW 1

GGGGCGCGGAAGTTGTGGCAG GARKLWQ 1

GCTAGGTGCCGGAGCGAGGTT ARCRSEV 1

GAGGGCAAGGCCGACGTTGCT EGKADVA 2

CTCCGGGCCGTTTGGGAGATG LRAVWEM 1

GAGAATTCGCCGGGTGCGTAC ENSPGAY 1

TCTTCCGGGGTCGCGAAGTGG SSGVAKW 1

ATTTTCCGCAGGGATTGGCGT IFRRDWR 1

AAGCGGAACCACCGGTGGATG KRNHRWM 1

GAGGGGCTCCAGGGGCGTGCT EGLQGRA 1

GTGCGTTGGCGCTGGCTGCCG VRWRWLP 2

CGGCTTGATTGCTCGCCGGTT RLDCSPV 1

TCTAGTCGTGGCGCGGGTGTT SSRGAGV 1

GTTGGGTTGCACCAGGTGGCT VGLHQVA 1

TGTTCCGGCAAGGTTAGTCCT CSGKVSP 1

GGGCTTGGCCGCTTGGGTTGT GLGRLGC 1

TATAACTGGTGGTGCTCCCGT YNWWCSR 1

TGTGGTCGTATGGCGCTGATG CGRMALM 1

TCGGGCGACAACGTTGCTCGT SGDNVAR 1

GGTGGTAGGAGTGGCCGGGAC GGRSGRD 1

TGCAGGGCGACCCCTGAGTCT CRATPES 1

TCGATGCGCAGCGATGGTCGG SMRSDGR 3

TTTGGCGTTCGGCCCCTGGGT FGVRPLG 1

GTGCACGCTTTGTCCGGTCAG VHALSGQ 1

GTGCTTCGGACGTCCCGCAAT VLRTSRN 1

CTCAATCGGGTGCGGGGGCAG LNRVRGQ 1

GGGTGTCGTATGGGGGATAGG GCRMGDR 1

AATTTCCGCGCGCAGAACGCT NFRAQNA 1

GGCAAGCTGGCGAGGCGTGAC GKLARRD 1

GGCGTGTGCAAGTTCTGCGAG GVCKFCE 2

GGCGAGCGGTTCCCGAACTAT GERFPNY 1

GGGGGCGCTTCTCGTAGGTCC GGASRRS 2

CGTAGGTGGATGCTCTTGGAG RRWMLLE 1

GTGTCGGGTGTGAGCGTGTCG VSGVSVS 1

GGCCAGATTGGGGCGTTTCTG GQIGAFL 1

GTCATGTTCTTCGTGTGGCGG VMFFVWR 1

GTGAAGTTTACGGCCCCTGGG VKFTAPG 1

GTCACGGTTGGGTTGCCTGCT VTVGLPA 1

TGCTCCTTGCCTAAGAGTCAG CSLPKSQ 1

AGCTTGAGCTTGCGGGTGACT SLSLRVT 1

TGGAGCTCTATGTGGCAGCCG WSSMWQP 1

AGCGCTGATGGGTTGTCTTGT SADGLSC 1

ATCGGCGACCCGTCCGGGGCT IGDPSGA 1

GTTTCTTGCAGCCGCATCTCG VSCSRIS 1

TGGAGCTTGGAGTCGGTGCCT WSLESVP 1

CATAGTGTTGCCCTGCCGCGT HSVALPR 1

TCTCTTCACACTCCGGCTCTG SLHTPAL 1

GCGTGCATCGTCAGCGGCGCT ACIVSGA 1

GGGACTCTGAGTTATCTTGAG GTLSYLE 1

GGGGCGGGTCTGATCCGTTTT GAGLIRF 1

TACTCGGGGGCGCCTGCTACG YSGAPAT 1

ATGGAGGCGCCGGTGCCCGCG MEAPVPA 1

GGGGGCTCCGTGGGCTTTGTG GGSVGFV 1

GTCCAGCGTGGGTTGATCCAT VQRGLIH 1

AGTGCGCTCGTCCTGTTGCTT SALVLLL 1

TCTTACATTGTTATTAACGGT SYIVING 1

CTGGTTTGGGTGGGCGCGTCG LVWVGAS 1

CGGTCGGTCCACTTCTGGAGG RSVHFWR 1

CTGTTGCTGCCTTCCTGTGAT LLLPSCD 1

GGTCGGAGCCGGATCTCCTCT GRSRISS 1

GCGTGTAGTTCCAGCGAGCGT ACSSSER 1

GTGCCGTGGCCGCTGTATTGT VPWPLYC 1

GTCTCTAGGACCGCGGATCTG VSRTADL 2

CGCTCGGCCGTGCCTTGTCGG RSAVPCR 2

GTGGTTGCCTGGCCCCAGAGC VVAWPQS 1

CATCACTGGTGGAGGGCGGCT HHWWRAA 1

GATAGTGTCCTTGTGCACTGT DSVLVHC 1

AGTCAGGGTGTCCTGTTGCGG SQGVLLR 1

TCGGTGATGAGGTACCTCAAG SVMRYLK 1

GGCCGCCTCTATCCTGTTACG GRLYPVT 2

CACGCTGTCTGGAGGCACAGT HAVWRHS 1

TGTGAGTCGGGGAGTTCGCCT CESGSSP 2

TTGTGGTCTGGGAAGAGCCCT LWSGKSP 4

ACGGGGACTGGGGGCTTCCAG TGTGGFQ 1

TCGCTGTCCTGGCTGTGTGCT SLSWLCA 1

AGGCGCGGCACGAGCGTGCCG RRGTSVP 1

GCGCTTCTTAGCGGGGCTTGT ALLSGAC 1

TCGGGTGTCGGGGTGCGTTCG SGVGVRS 1

GGGCAGGTGGTTGTTTATTGC GQVVVYC 1

GAGACGTGCAGTTCGACCGCG ETCSSTA 1

GGTGCTCCGAGGGCTTACCTT GAPRAYL 1

GAGGTCTCGGAGTGGTGGGTG EVSEWWV 1

TCGATGGCTTTGGATCGCAAG SMALDRK 1

GTGAAGGTGATCGTCTGGTGT VKVIVWC 1

GCTTGGCGGCGGGTCAGGCCG AWRRVRP 1

GTGAGGTTCCGCTTCCGTTGT VRFRFRC 1

CGGCGGTGGAATGCGCACAGT RRWNAHS 1

GGTATCGACACTTCGGTTCCT GIDTSVP 1

TCTTGGGGCTCCCTGTGCTGG SWGSLCW 1

ACTAATCAGTCGGGTTCGGGT TNQSGSG 1

AATGTGGTTGCCATCAATTGG NVVAINW 2

TTGTTGGAGTTGGGCGACGCT LLELGDA 1

AGGTTGGGCTGTAGCGCGCGG RLGCSAR 1

GGCTCGCGTGGTTATGGTCGG GSRGYGR 1

AGTTCGGTGTATGGTTATAAT SSVYGYN 1

TTGTGGTCGGTTGGTTCTGCG LWSVGSA 2

ACTGGTAGGACGAATGACAAC TGRTNDN 1

CTCGCCACTGAGCTGTGGCAT LATELWH 1

GGGTGGTGCAGGCCTGTCCTG GWCRPVL 1

CGGGATGGTGCCACCGTGAGC RDGATVS 1

GGTCCGAGCGAGTGGGGTTGG GPSEWGW 1

CCCAGCGTGGGCGGCTTTGGT PSVGGFG 1

AGCAGCTTTCCGCGCGGTCCG SSFPRGP 1

CGTTGCTGTTGCATTCGGTTG RCCCIRL 1

GAGTTGCTTACCGGCGCTTGC ELLTGAC 1

TTTCTTTTCCGTCCTGGGGAG FLFRPGE 1

TGCGATAGGCTTCGTGGTGGT CDRLRGG 1

TTCATGGATTTGCGTGGGAGT FMDLRGS 1

CTTGGGGTCTCGCCGCACCGT LGVSPHR 1

ATGTGGGAGTGCGGGGGTTCG MWECGGS 1

TTGCGCGTGCACCTGTCGCCG LRVHLSP 1

GGCTTGAGCTTGGCGTTTGCT GLSLAFA 1

GGGGGCGTTGGTAGTGAGCGG GGVGSER 2

CGGACTGACTCTACGCGTGTT RTDSTRV 1

CGGGTTACCGCTTGGTTCCTT RVTAWFL 1

AGTCGCCGCTATTATGATTGT SRRYYDC 2

TTCGCGTTGGTCCGTGGCAGC FALVRGS 1

TGGGTGGCGTTGCGGGGGATT WVALRGI 1

ACGATGCACCTGCCGTCCTGT TMHLPSC 1

CCGTACCATTGTGGCGCCCCT PYHCGAP 1

CTGTGGTTCAGTGTCACTGTT LWFSVTV 1

GAGGCCGACATTCGCGAGCTT EADIREL 1

CGGGGGGCCTGCCTTGCTGAT RGACLAD 1

CAGGATCGCGACGCCCTTCAG QDRDALQ 1

AGCTTCTCGCGGGCGAATAGG SFSRANR 1

AGGTGGGACGTTGATGGTGGT RWDVDGG 1

GTGTGGCCCCGCCCCGGCGCG VWPRPGA 1

AGCCATAGCGTCCAGGGGCCG SHSVQGP 1

ACTAAGAATAAGTGCGGCGGT TKNKCGG 1

GGTACGCGGAGTACCTGGGCT GTRSTWA 1

GTCTCTATCGGCCGCTCGGTG VSIGRSV 1

GTGTGCTGTCCCGGCGCGCTT VCCPGAL 1

AGTAACGGGTTCACGGCCATT SNGFTAI 2

GCGAGTCAGTGGGGCTCGTTT ASQWGSF 1

CTCCGTTGCTACGTCGGGAAT LRCYVGN 1

AGTCTGTCGTGTGACCATTGC SLSCDHC 1

CTTTTGTCGAGGCCGGCGGTT LLSRPAV 1

TGTCAGTTGGGCCAGCGCAAT CQLGQRN 1

AGTCGCATGATGCTCTTGGCG SRMMLLA 1

TTCGAGGCGAAGTGGGCGGAG FEAKWAE 1

GGGCTCGAGACGTCTACGGGT GLETSTG 1

CGCGAGCTTTGGTCTGGGCGG RELWSGR 2

GGGAAGGCTGCGAGCTATTGG GKAASYW 1

AGCCAGCTTAGCCGCGCGGAT SQLSRAD 1

ATTCGGTCCACGGGTGCTCGG IRSTGAR 1

GATCACTGGGTCTGTTTTCTT DHWVCFL 1

GGTCGCGACAACTGGCCGGCC GRDNWPA 1

AATTGCGTCGAGCTGGCTTTG NCVELAL 1

CTGCGGGTTCGCGAGTGTTGT LRVRECC 1

TTTGTTTACACGCGGTCGTCG FVYTRSS 1

TATCAGCGTAGTCACATCAAC YQRSHIN 1

TGGGCCTGGGCGAGCGCGTCT WAWASAS 2

TTCGGCATTGGTTATCGGTGT FGIGYRC 1

TGCCGTGCGTGGACTGCGCCG CRAWTAP 1

GCTGTCTTGAGTCAGTCGCCT AVLSQSP 2

ATTGGTGGCTGTGTGCTCTCC IGGCVLS 1

GCGATTTTGCGCGTTGTCGAT AILRVVD 1

TCGATGTTGAGCTGGCGCGAC SMLSWRD 1

TATCTTTATATGCGCGTGAGT YLYMRVS 1

CAGACCGACTGGGGGGTCTCG QTDWGVS 3

AGGCGGTGCGGGAGCTACCTT RRCGSYL 1

GGTAAGCGCAGGTGGGCTATT GKRRWAI 1

ACTTGCACCGCTTGGGTTTGT TCTAWVC 1

AATCGCGATTCTATGAAGGGT NRDSMKG 2

CTTTTGAACGGCCCGGAGGGT LLNGPEG 1

TCGACCGGGCATAGGGTCGTC STGHRVV 1

AGGTCCCAGTTCTTCGTTTGG RSQFFVW 1

GGCGGGGCGCTGACGTGTGGT GGALTCG 1

GCTCGGCTTCGCCACACGTCG ARLRHTS 1

GCCGACTCGTGTAGCGTGTGG ADSCSVW 1

GGCTTTGGGAGCACTGTCATG GFGSTVM 1

GACGTGATCTTGGTGGAGTGC DVILVEC 1

GGGGACTATACGGTTTGCCTT GDYTVCL 1

CCGTTTAACAATGGGCTGCTT PFNNGLL 1

TGGATCGCGGGCGCGTTCAAG WIAGAFK 2

GATCGGGTCTGCGGCCTTTAT DRVCGLY 1

GCTCTGTTCTTGCTGCCCACG ALFLLPT 1

ACCCTGGTGGGTGCCCTGCTC TLVGALL 1

CTCAGCGCGGTGGGTGAGGCT LSAVGEA 2

AGTTGGACGGTGGGCGGTCTT SWTVGGL 2

CAGGATGCGTGCTCGGTCACT QDACSVT 2

AGCTCCCTCTCTTTCGGCAGG SSLSFGR 1

CCTCCGCTCAGCGTCGGGCCG PPLSVGP 1

ATTGTGGTCTCGTGCTTCGTT IVVSCFV 1

GGTGTTTCTTTCGACTGCCGG GVSFDCR 1

GATTTTCGGCTTCTCTTGGGT DFRLLLG 1

GTGCGCTTCAGTGTGAGCTCT VRFSVSS 1

CTGACTCACGATACCGAGTGG LTHDTEW 1

CTGAGGAGCGGGGGGGATAGG LRSGGDR 1

GAGATGGTTTCCCGCACGGGG EMVSRTG 3

TGCCTTACTAAGCGGCTTCTT CLTKRLL 1

CGCGTTCAGCGGGTGACTTTG RVQRVTL 1

CCGTGTTCTGTCAGGGTCTGT PCSVRVC 1

TTTTTTAGTTCGGCGTGGGGG FFSSAWG 1

CGGCGCTGGGGTCTCACCCGG RRWGLTR 1

TGGGAGCAGTCGGGTGGGTGT WEQSGGC 1

CCTGTCAGCGTTAGCGGGTTC PVSVSGF 1

ACGGCCCGTAAGTTTACCGCG TARKFTA 1

CGGGTGGGTCTTGCCTGTCCG RVGLACP 1

ATGGACGGCAGCTGGATGGTT MDGSWMV 1

TACCCTGTGTATCCCGATGTT YPVYPDV 1

CAGGTTCTGTCTCGCCGTGTT QVLSRRV 1

CCCGGGTGTTGTGTTCTGATC PGCCVLI 2

GGCCGGGGCTGGCCCGCGTAT GRGWPAY 1

CGTTGTTGTGCCAGGAGTATT RCCARSI 1

GAGGTGTGGTCGAGTCCTTGT EVWSSPC 1

ATGACGCGGTGCTCGCGCGAG MTRCSRE 1

GGTTGTGGTAGTTGCTGTTGT GCGSCCC 1

GTGGAGCGGACCTGCATTGTG VERTCIV 1

GTGATGTACCGCCACGAGGTG VMYRHEV 1

GATTGGCCGTCTCATATGGCG DWPSHMA 1

CCGTGGTGCAGCATTGGCCTG PWCSIGL 1

CGGTTGCCGGCTGATGCCGGT RLPADAG 1

GGGTTGCCGAGGCGCGGGCGG GLPRRGR 1

GCTTTCCTGGGTCGGAGTGAG AFLGRSE 1

GGGTGCCTGCCCAACCTGACG GCLPNLT 1

ATGCATCGCGAGTGCTCTCTT MHRECSL 1

CTCGGTAGCTCCCGCGTCTTT LGSSRVF 1

GGGTTCTCCTGTCTGGCCGGG GFSCLAG 1

TGGGTTCTCGGCGGCGAGATT WVLGGEI 1

AGGAAGCAGTTTGGCAAGCTG RKQFGKL 1

GGCATGCTTACTACCTCTCCT GMLTTSP 1

GCCTGGACGAGGGCGTGTATG AWTRACM 1

CTTCTTGGCGCTTTCATGAGT LLGAFMS 1

TCGCGCCGCGTTTATGGCCCG SRRVYGP 1

CGCGGGCCGGCCGATGTTGAG RGPADVE 1

AGGTTCACGGTGCTTATGCTT RFTVLML 1

CCGAGTGTGAGGCCCGTGTCG PSVRPVS 1

TTGAGGGAGACCCATGTTAGT LRETHVS 1

TTTCGGCGCAATATCGGGAGC FRRNIGS 1

CCTGCCGCGAGGGGGGCGCTT PAARGAL 1

AATTTTGGGGTCGCTCTCGGT NFGVALG 1

CTCTCGCGCAGGAGCGTGTAT LSRRSVY 1

CTGGGTGGCAAGAGCTCCCGT LGGKSSR 1

ACTACCTCTCAGCATGGGCGG TTSQHGR 1

TTTTCTTCGGACCTGGGGCGG FSSDLGR 1

GGCCTCTGTGGTCGGGCGCCT GLCGRAP 1

GTCTTTCACCGCTGGTCTTCT VFHRWSS 1

AGGCAGAGGGGCGTTGGTGGG RQRGVGG 1

CCTTGTGCCAGCTGTAGTCTG PCASCSL 1

GAGTTCAGTTTCGTCTCCCGC EFSFVSR 1

GTGACGTTGGACGTTTATGGC VTLDVYG 1

TTCCGTGGGGTGTTCAAGAGG FRGVFKR 1

GGCGAGGTGCGGCTCTCTGAG GEVRLSE 1

GAGCTTCTCAATCTGCGGAAT ELLNLRN 1

AAGGGTGGTTGTCATTTCCAT KGGCHFH 1

TTGGTTGTCAGGCAGCAGCCG LVVRQQP 1

GGTTGCCGTGCTTGCTCTCCT GCRACSP 1

TCCGTGGCGAGGCTCTGTGCT SVARLCA 1

CGTTGTAGTTCGGTCAATGCG RCSSVNA 1

AAGCCGGACTCGCGGTATGGT KPDSRYG 1

GACATCCTTTGGCGTGGTTTG DILWRGL 1

GTGTGTCCGAGTAACGGCCTT VCPSNGL 1

TCTCGGTCCGTCTTGGAGTGC SRSVLEC 1

CGGTGGGCTCTCTATGCGTCG RWALYAS 1

CTGCCTATTACTGCGGTCGCT LPITAVA 1

CGCCCTGACGAGGGCTGTTCT RPDEGCS 1

CGGATGCGCGTGGATCTCCTT RMRVDLL 1

CTTCATAACCGTGTCTGGCCT LHNRVWP 1

TGGGCGTCGGAGTCGGTTGCG WASESVA 2

GCCAGCGCGATGTGCCCTAGT ASAMCPS 1

CTCATTTGCTCGGAGTGGTCG LICSEWS 1

AGTATCTTGGCGCCTCGTAGG SILAPRR 1

ATGTCGCTTGACTGGAGTAGT MSLDWSS 1

CGTCAGGGGGGGCTGACTTTG RQGGLTL 1

CCGTTCAACGGCACGTGCGGT PFNGTCG 1

TGGTGGCGCGCGTCCCGCGAG WWRASRE 1

TCTACCCGGTTCGTCTCGCCC STRFVSP 1

GCCGTTCTTATTGCGAAGCGT AVLIAKR 1

TTCCAGGACACGTGGTATGTG FQDTWYV 1

GGCCCTCGGAGTCGCGCGTGT GPRSRAC 1

CCTCTGGTCATGTCGGATCCG PLVMSDP 1

TCCGGTGAGTGGTACCAGCGT SGEWYQR 2

GGTTGCATGACGACTTTTGCG GCMTTFA 1

AGTCATGCTCAGAGCGCTATT SHAQSAI 3

GGGCATAAGCCTGCCGTTGGT GHKPAVG 1

AGCTGGGTGGTGTCCGTCGCT SWVVSVA 1

ATGTCGGGCGAGCAGGGCCGT MSGEQGR 1

GCCCGGCGCTTCTGGCACTGG ARRFWHW 1

GAGCATGTCGCGATGTCTTGG EHVAMSW 1

GGTGGGGAGCTTGTTGTCCGG GGELVVR 1

TTTTCCGGTTTGCTGGCCTGT FSGLLAC 1

GGCGTCTCGAGTGGTGGCCGG GVSSGGR 2

GTGGGCTCCTGTTCTGAGTCT VGSCSES 1

GGTTCGGTTATGCGTGCTTGC GSVMRAC 1

TGGCATGGCACTTGCAGCGGC WHGTCSG 1

CCTCGCGATTGGTTCCTTGCT PRDWFLA 1

GCTGGTCCTATGGCTGTGGCT AGPMAVA 1

TCCAGGGCCCATGCCCCTGGT SRAHAPG 1

TTGGTTAACAGGCAGCTGCGG LVNRQLR 1

GCGGATGGTTCGCTCGGTGCT ADGSLGA 2

GTCTTTTCTGGGTGGAGTGCT VFSGWSA 1

GGCGCGAGGGCCGTCCGGTGG GARAVRW 1

CTGCTGGAGGATGCGAACCGG LLEDANR 1

TCTCCGGACCATGACGATGTT SPDHDDV 1

CCCGCGCTGTCCCGCGTTAGG PALSRVR 1

TGCCTTCGGCACCTCGCCGTT CLRHLAV 1

AGGCTTTCCGGGGTCTGTTCG RLSGVCS 1

CCCAATGGCTGGCGGCAGCCT PNGWRQP 1

TGTAGGTCGGCTGAGTGGTCT CRSAEWS 1

TTTTGGCAGACGCGTGGGTCT FWQTRGS 1

GCGTGCTCGAGTCTGTGTTTC ACSSLCF 2

CCGGTTAGGGCGCATAGGCCG PVRAHRP 1

CTGACCGCGTACTGGTATTTG LTAYWYL 1

CATAATTATGGTTACGTGACT HNYGYVT 1

GCTCAGCAGGGCAAGTTTCAG AQQGKFQ 1

GTGATCTTGGGGGCTTCGCGT VILGASR 1

TGCACGCGCATCGCGCATCGG CTRIAHR 1

TCGCGGGCCCGCGATATTGTT SRARDIV 1

CGTACTCTGACCCACTTGCTG RTLTHLL 1

TGGAGGTTGAGTCTGGTTGGT WRLSLVG 4

TACGTGGCCTGGTACTTGCCT YVAWYLP 1

AATTGGGGCGGTTGGCGGTGG NWGGWRW 1

GTTACGCTGAGGGTTCTTCGT VTLRVLR 1

CCGTCCGGGAGGGTGTTGCCG PSGRVLP 1

TATAGCCTGTCCCACTCGCCT YSLSHSP 1

ATCGAGCATAATGTGGTTAGC IEHNVVS 3

CAGGTTCATGATTTTAAGGCT QVHDFKA 1

TTTTTCCCTGTGGCCTGGGGT FFPVAWG 1

TCCTGCCGCGGGCATTGCAAT SCRGHCN 1

CGCGTGCAGCTCTTTTGCTAT RVQLFCY 1

TGGCGGCTGCGGGGCCTCGGG WRLRGLG 1

CGGGTTACTTCGTGTTGCCGT RVTSCCR 1

TCGCAGCTTGAGGGGGGTTGT SQLEGGC 1

GACCTGATGCTGTGTATCGAT DLMLCID 1

AGTTTCGGTGGTTACAGTTCT SFGGYSS 1

CTCGTCGTCTCGCTGGGCGCT LVVSLGA 1

TTGGATGTCATGTGTTATAGG LDVMCYR 1

TTTGGTCTCATTGTCGGCGCT FGLIVGA 1

AGGTTGAGGCACACGGCGCCT RLRHTAP 1

TTTGACCGCTTGGGTACGTGG FDRLGTW 1

CTGCGGTTGATGTTGGCTGTT LRLMLAV 1

ATGCGGCTCAAGACGGCGGCT MRLKTAA 1

CATTGGCGCGCGTGCTGTCGG HWRACCR 1

GCCCAGTGTAGTTCGCGGGTT AQCSSRV 1

ACCTATTTCGCTGTTTGGTTT TYFAVWF 1

ATCTCTCCTAGCGCGGGGTGG ISPSAGW 1

CGTCATAGCAGGTGGGCGACT RHSRWAT 1

CTCCGCTCCCGTGTGGTCTGG LRSRVVW 1

GCTGGCTATTTGATGCTGTGG AGYLMLW 1

GTGGGTCTTACGTGCCGCTGT VGLTCRC 1

ATGTCTTTCTTGTGGTATCTT MSFLWYL 1

TTTCACTCTGGGGCCACTTGT FHSGATC 1

TCTTGGTACGCGGGCTTTAGT SWYAGFS 1

CTTCGGATCAGGCGGGCGTCG LRIRRAS 1

GTCCCTCAGACCTATTGCGAT VPQTYCD 1

GTTTTTCAGATCTGGAATTGT VFQIWNC 1

CGGGCCGTGAAGCATCTTTCT RAVKHLS 1

GTCCACCACTCCGTCCGTTCT VHHSVRS 1

AGCGATACGACGGTCTGGGGG SDTTVWG 1

GCGCCGCCCGGCGGCCCGGTT APPGGPV 1

GGTGGTGGGTCGGGGTCGCCG GGGSGSP 1

GCTTGGTGTGTCTACCCTGCG AWCVYPA 1

GACGCGGACGCGCTGAGGTCT DADALRS 1

GGGCGTGGTACCGATTGGAAG GRGTDWK 1

AATGCCTACAGTAAGGGGGGT NAYSKGG 2

GAGTTGCACTGGGCTCGGTGG ELHWARW 1

GAGTTTTGCGATTGCGCGTTC EFCDCAF 1

TTTGGCGTCCTCCTGTTTTCT FGVLLFS 1

ATGCGTGTGAGTTGTGACCTG MRVSCDL 1

CATGCTGAGACGCAGCTGGGG HAETQLG 1

CGTGGGGCTTGCTGGGGGCGC RGACWGR 1

CGGGCGTTTAGCGTCGTCGGG RAFSVVG 1

ACCAGTTCGGTGGTGTATCAT TSSVVYH 2

TTTTATATCTCTGAGAACTCC FYISENS 1

TGTCGGGAGGTCTGCGTCATT CREVCVI 1

GCTCGGCCCGGGTCCGCCAGG ARPGSAR 1

AAGGATAGCCCGGGTTTCTGG KDSPGFW 1

TGCTCGAGGATGTTGGCGCCT CSRMLAP 1

GTTCGCGATGTCGGCTTGGGT VRDVGLG 1

TCGTGGGGGCTCGTTGTCGTC SWGLVVV 1

TGGTCGGTTCGGTGCTTCCAT WSVRCFH 1

ATGGTCGGGTCCGCTAGTTCT MVGSASS 1

CGCGGTTCTCTGGTTGTGGCT RGSLVVA 1

GAGGTCGGGTGCGCCTGCACT EVGCACT 1

TGTGTTCGTGGGGATGTCTGG CVRGDVW 1

CATTCCTTGTGCACTGAGATC HSLCTEI 1

GTGGGCTATGCTTGTTCGAGT VGYACSS 1

TGCATTCACCGGGTGCGTCGT CIHRVRR 1

CAGAATTCCACTTCCTCGTTG QNSTSSL 1

CGTGTTCTGTGGGTCAGTCAG RVLWVSQ 1

TGTCCGTATGATGGCCGGTGT CPYDGRC 1

CACCAGTGGTGGCGCAGCAAC HQWWRSN 2

TCTGTGTGTTGTGTGCTTCAT SVCCVLH 1

GTGGGTGGGGAGTGCCGGACT VGGECRT 2

AGTGTCGTTCCTTCGTGGTGT SVVPSWC 1

TGGCGGCGTACTTCTCACAGG WRRTSHR 2

TGCTGCGACTGGTCGTCGGCT CCDWSSA 1

CCGGCGCGCAGTTTCGACGGT PARSFDG 1

GGCGGGGGGATCGGCTGGTGT GGGIGWC 2

GGCGGCGGTTCTATGGGGTCG GGGSMGS 1

CTGGTCAGTGTCGCGGGGTGT LVSVAGC 1

GTTTTTGACAGCTTGTCTTCC VFDSLSS 1

AGCCGGGTTAGCGACGTGGCT SRVSDVA 1

CAGCGGAGCGTTTGGTGTGTG QRSVWCV 2

GGCATTCTTCCGCGGGCTGTG GILPRAV 1

TCTTCGTACGCTAAGGAGCCG SSYAKEP 1

GCCTGCCGGCAGTACGGGGCG ACRQYGA 1

CGTGTCCTTTCCGGCGGGACT RVLSGGT 1

GTCCCCCCTCTCCGGAGGGCT VPPLRRA 1

GTGCCTCGTCCCTCCGGCCTG VPRPSGL 1

CGTTTGCCGGGGTATTATCCC RLPGYYP 1

AAGTTCCTGCAGCGGCTTCCG KFLQRLP 1

AGGCAGGCGAACGCGGTCCCG RQANAVP 1

CTGCGGGTGAGCGGTAGGCTG LRVSGRL 1

TCGAGCACCTCGCGGGTGCTG SSTSRVL 1

AATCCGGACACCACTTCTCGG NPDTTSR 1

GGGGGGCTGACCTTGCAGGTT GGLTLQV 1

GTTGTTCGCAGTGCGGCTGTG VVRSAAV 1

GTCGACGGGATTGGCGTGTCT VDGIGVS 1

TTGATCTCCAACTACCGCTGC LISNYRC 1

TGTATCAGGCAGCTCGCCGAG CIRQLAE 1

GGGCTCGGCGATCTGCCTCGT GLGDLPR 1

GGTTGTACTGGTGAGGGGGCT GCTGEGA 1

TGCTCGGGTCGGGGGACTGGG CSGRGTG 2

ACGGTGTGGGTCGGCGGCTGT TVWVGGC 1

CAGCGGGTGCGTAGCCCTGTG QRVRSPV 1

GAGGCTTGTTGTCGCTGTAGT EACCRCS 1

GTGCTCGAGGATGCTGTCCGG VLEDAVR 1

GTGGCGGATGCGAATGCTTCT VADANAS 1

CTGAGGGTTGCTCTCGACGAG LRVALDE 2

GATTCGGTCGCCGACGTCTAT DSVADVY 2

TTGTGCGAGTGTGATGGGGAT LCECDGD 1

ATCCTGCCTTTGTCTGCTGGT ILPLSAG 1

TCTGGGCGGGGGAGCCATGCG SGRGSHA 1

GCGGGCGAGGTTCATAAGGCG AGEVHKA 1

GGGCTCAGCTTTGATGGGCCG GLSFDGP 1

TACGTGCCCTGGCGCCGCTGT YVPWRRC 1

CGCGGTGGCACGGTGTCTGGG RGGTVSG 1

TCTGCCCGTCGTTTCAGGTCG SARRFRS 1

GTGAGGCGGGGTGATGAGTCG VRRGDES 1

CTTCGGGGCGACCGGCTGTTG LRGDRLL 1

GTGGCGTGCGTTTGCGTCCGG VACVCVR 1

TGCGTTCTGCGTAAGCTGCGG CVLRKLR 1

ATGTGTACGAAGCGTAGTCTT MCTKRSL 1

AGGGTGGCCTTCAACTGCCGG RVAFNCR 1

GCTCAGAGCCGGATCTGCAAT AQSRICN 1

GTCTTGGGTGCGTTCGGCCGG VLGAFGR 1

AGGGGCTTCACCTTGGGTGTT RGFTLGV 1

TGTGGTCTGTTGTGGTGGTAT CGLLWWY 1

ACCAGTGAGCGGCCTAAGCCT TSERPKP 2

CGGAAGCAGACGGAGTGGGTT RKQTEWV 1

CTCGGGGCTCTGAATTCGTCC LGALNSS 1

CGGGGGAGTGGCGCCGTTGGT RGSGAVG 1

TACCGGCGTACGTTGGCGAGG YRRTLAR 1

GGTTGGGTCGTCGTCTCTAGT GWVVVSS 1

GCGGTGCTTGGCTGCTTGTCT AVLGCLS 1

TGGAATATCTCCCGCTTCACC WNISRFT 1

CTCCGGGTGTGTCTGACTTGC LRVCLTC 1

CCGTCGGGGCGGTTCATTTCT PSGRFIS 1

CAGGGGCCTCCGGTTTGGAGT QGPPVWS 1

CAGCTTCGGCGCCTCTGCGGT QLRRLCG 1

TTTGACATGTGCATGCTCATT FDMCMLI 1

AAGTTGCGCATTCCGGCTTTG KLRIPAL 2

TTGCAGATTATTAGGTGTGGG LQIIRCG 1

GGTACGCGGCAGGCGATGACT GTRQAMT 1

CTCTACGGCCAGCCGCCGGAC LYGQPPD 1

TCTACGGTTTGCGTTCTGCGC STVCVLR 1

GGTTATACGTGCAGCGATAGG GYTCSDR 1

AGCCCCCTGCCGCGGCTCCTG SPLPRLL 1

CACGTGGGGTCGGACAATTTC HVGSDNF 1

ACCGATTTCGCCTTCGAGGGG TDFAFEG 1

CAGCCTGGTTTGTCGGACCCT QPGLSDP 2

GGGCGCTGCGGGCGGACTTCG GRCGRTS 1

TATACCCCCCTCAACAGTCCG YTPLNSP 1

TCTGTGTACGGCGATCCCAGT SVYGDPS 1

AGGCTGCCGGGCGGCCGTGCT RLPGGRA 1

GGTGGCTACTGTTCCGCCGGG GGYCSAG 1

TTTCGTCGCGGGTTTTTGCAT FRRGFLH 1

GGCTTGACCGCGTGGATGCTC GLTAWML 1

AGTGGGCTCAGCGACTTTCGG SGLSDFR 1

TGGGGCACTATTGCGAAGATG WGTIAKM 1

TCTTGGCGCGGCGATTTGACT SWRGDLT 1

GGGGATTGTGACTGGTTTAGT GDCDWFS 1

TGGTGGAATGTGGAGGGTTTT WWNVEGF 1

GTTTATCCCACCCTGGCGGCG VYPTLAA 1

TTGGGGCACTCGATGGGGCGT LGHSMGR 1

AGCGGTGGTTTGAGCGTTACT SGGLSVT 1

ATTGCCCTCCGGCGTTCGCGG IALRRSR 1

CGTGCGCTCGTGGACTGGTTT RALVDWF 2

CTGGGGTACTACGGGTCTATG LGYYGSM 2

GTCATTTCGAAGTGTCCTGCG VISKCPA 1

GAGCTCTTGTGGTGCTGTCTT ELLWCCL 1

GGTTGCGATCTCTCGGCGTTT GCDLSAF 1

CGGGCGGGCTGGTTGGTGGGG RAGWLVG 2

TTGCCTGCCACCACGCGGCCG LPATTRP 1

TTCGTGGAGGAGGCGTGGTGG FVEEAWW 1

TTTAAGCTTGAGGGGCTTGTT FKLEGLV 1

GTTTTGGAGGTGGTGAGCAGC VLEVVSS 1

TTGACTACCACGTTGATCAGG LTTTLIR 1

ACCTCCGATGGGGGCCGGACG TSDGGRT 1

AGTGAGGCCGGGGCGGCTGTG SEAGAAV 1

CAGGATTCGTATCAGAGCCGT QDSYQSR 1

CACCCGGGGGTGCTTCTCGTG HPGVLLV 1

GCGTGGCGTACTGTGCGGCGT AWRTVRR 1

TTTCGCGTTAGTGGTGCGTGG FRVSGAW 1

GGTTTTCACCGCCGCTGCCAG GFHRRCQ 1

ATGGCGAATTTGAAGCGGTGG MANLKRW 5

TATCGGGTGTGGTCTAGGGGT YRVWSRG 1

GTGGGTTGGTGGGTGTGGGCT VGWWVWA 3

GTTTCGGTGTACACGTTCTGG VSVYTFW 1

GTCGGCCTCCGGGGGGTTATT VGLRGVI 1

GTTATCTTCATGACTGCTCGC VIFMTAR 1

AATCGGGCTACGACTGGCTCT NRATTGS 1

AGGGCCTCTTGGAACAGTCTT RASWNSL 1

TGGATCGGCCATGAGACTGAC WIGHETD 1

GTTGTGCCTTCGTGCACCCCC VVPSCTP 2

TGTCGTGGTCGCAGGGCGTGT CRGRRAC 1

CGTCGGCACAGCGGGGTGGGT RRHSGVG 1

CATGTGGTGTGGGGTGGCAAG HVVWGGK 1

TCTTGGAATAGCGAGAGCCGT SWNSESR 2

GACGGCGGGTGCGTCTGTAAT DGGCVCN 1

ATTGCCTACGGCCAGTACAGG IAYGQYR 1

GTTGTGGTGAACGACACCGCT VVVNDTA 2

GCCGTGCTGAGTTTGGATTCG AVLSLDS 1

TTGGAGGAGAGGCGCATCGAT LEERRID 1

GGTGGGATCAGCCCGGCTAGC GGISPAS 1

TGCGGTTGTTGGTTTTGGCGT CGCWFWR 1

GATGGTTTGGCGGTGCGGAGG DGLAVRR 1

GGGAACCTGGCTCACCGTTCG GNLAHRS 1

GACCGCCTGTGGTGCACTCTG DRLWCTL 1

GAGGTCGCCGCCCGTCACCGG EVAARHR 1

GCCACTGGGGGCTGTCTGGAT ATGGCLD 1

GCGGCGCCTAGTCCCGCTATT AAPSPAI 1

TATAATACGCCGTGTGGCGCT YNTPCGA 1

GGTATCTTTACTCGGCCGCTT GIFTRPL 1

CTTGGTTCGCATGAGCAGCAG LGSHEQQ 2

GGGCGCAGGCTTGGCGTTAGG GRRLGVR 1

GGTGGCCTCACTGGGATCGTT GGLTGIV 1

AGGCCCCGGTATCCGACTAAG RPRYPTK 1

GGGTGTAGGATGAGGTCTTCG GCRMRSS 1

TCGCTTGTCCAGTACTCCAGG SLVQYSR 1

GGGTCGTTTATGTGGCTCTCT GSFMWLS 1

TCTCCGACTTGGTGGCGGCCT SPTWWRP 2

TCGTGGAGGTGTGGCCATTTG SWRCGHL 1

ACTTGGCGCCTGGGGCCTCTT TWRLGPL 1

CAGTGTTTGTGGGCGGAGGTG QCLWAEV 1

CGGCGCCCTATTGCTACGGAC RRPIATD 1

GTCAAGTTCAGCTGGCTCGTG VKFSWLV 1

TTGATTCGTGCCGGTGGTAGG LIRAGGR 2

CATCGCCCTGGGTGGGGTACT HRPGWGT 1

ATTAACTTGCCCTCGGGTGGG INLPSGG 1

GGCTCTCTTAATGTGCTGACT GSLNVLT 1

TTTGCGTGGTGGCCCGGCTGC FAWWPGC 1

CTTTCGTCGAGCTGGTGGTGT LSSSWWC 1

GTTAGCGACGTGATTTGTCTG VSDVICL 1

GAGCGCAGTTGTCCTTGGAGT ERSCPWS 1

AGCCCGGGGGTCTGTCGTTCT SPGVCRS 1

GTGCAGGGCCGGAGCGGTGAT VQGRSGD 1

GGGTCGCGGGGGTGCTCTTTG GSRGCSL 1

GCCGTCAGTAGTCCTAACGGC AVSSPNG 1

GTTGTCCCGAGTTTGGGTGTG VVPSLGV 1

CTGCGTCGTTGCGCGAACTTT LRRCANF 1

GGGCGTCGGTGGACTTATCCT GRRWTYP 1

GGCCCGTACTTCAACTGGCCG GPYFNWP 2

TTTAATTTCCGTCTGCGTTCG FNFRLRS 1

GACGGGGCGAGGATGCCGGTG DGARMPV 1

TCCGGGGGCGCCTCCAGGATT SGGASRI 1

GCGGTGGTGTTCCTCCGCATT AVVFLRI 1

CGGGTCGTGATCCATCGCATG RVVIHRM 1

GCTGGTGCGCCTGGTGCGCCT AGAPGAP 1

GAGGTTTCGCGGGGTCTCTGG EVSRGLW 1

AAGCTCCCCGCGACCTTCGCG KLPATFA 1

ACGCGCCTCGTGTACTGCGCG TRLVYCA 1

TACTGGGCCAGCCCTCTTTTC YWASPLF 1

AGCCGGCCGATGGGTTTGCCT SRPMGLP 1

ATCAGGCGGGACTTCAGGATG IRRDFRM 1

GCGAGCGTTGTCCACGCGCTT ASVVHAL 1

ATGGGGTACCGGAGGCTGACG MGYRRLT 1

GTTAGCGGGGTGTTGCACCTT VSGVLHL 1

GTCGAGCGCACGGCGATGCTT VERTAML 1

GGGCAGTTTACGACCTACGAG GQFTTYE 1

TGTGTGACGAAGAGGCCGGGG CVTKRPG 1

ACGGCTTGCGCCGGTGCCTGG TACAGAW 1

GACCTGCACTTTGGTATTTCT DLHFGIS 2

GAGGCGGCCCACAAGATGTGG EAAHKMW 1

ATTCTGCGTTTCCGGTGCTGC ILRFRCC 1

GAGTCGGTCGTGCCCAATGCT ESVVPNA 1

AAGGCGCACTGCGGCCGTCAT KAHCGRH 1

GTTCATGTTGCCATGGAGCGG VHVAMER 1

TGTTGTGGCCCCGTCATCGGT CCGPVIG 1

GTCCGTCGTTCGTCTGCTGCT VRRSSAA 1

CAGCTCCGCGTGTCGTACTCT QLRVSYS 1

TCGGTCCACACCAGCGGGTTT SVHTSGF 1

GCTGGTTTGTGTTGTTTGGCT AGLCCLA 1

GATGCGCTTAAGAATAGGGCT DALKNRA 2

CGGGTGGAGAGCGTCTGGGCG RVESVWA 1

CACGGCGAGATCGTTGGCACC HGEIVGT 1

GGCGGCGATGGTGCGCTGGCT GGDGALA 1

TACAAGGGGGCTAGGCCGGCG YKGARPA 1

TTGACGCGGGGGTGCCAGCTT LTRGCQL 1

GCCTCGCGCTATGTTTGGGGC ASRYVWG 1

ATCAACCACTTCCAGTTGACT INHFQLT 1

TGGACCAAGGATACCGCCTGT WTKDTAC 1

TCTATCCGCGGTCACTCCAGT SIRGHSS 1

CCGTGGAGCATGCTCGGTACC PWSMLGT 1

CATGGGTGCAGGGGGAGGTCG HGCRGRS 1

CTGTTTCACGCGGGGGGTACG LFHAGGT 1

ATGGCCGATAGGCGTGCTCTT MADRRAL 1

TCCTGTGCTGCGATGGTTGTG SCAAMVV 2

GGGAGCTACACCGTGCCCCCC GSYTVPP 1

GGGCTGGGCGCTCTCAAGTTG GLGALKL 1

GTTATGGGGACCGACGCGCCG VMGTDAP 1

TTCGTTCCTTTCTGGTTTTCT FVPFWFS 1

GTCTGGTGGCGGTTCGTTTTC VWWRFVF 1

GATCGGGACTCGCTTTTCCCC DRDSLFP 1

GTCAAGGTGCGCTTCGGGTCT VKVRFGS 1

TTGGTCCTCGACTCGAGGACC LVLDSRT 2

GAGGTGGGGACTGGGAGCGTG EVGTGSV 1

CACAGTTTGAGGCGGTGCAAG HSLRRCK 1

GGGCCGTGGGGCTGGTCTGGT GPWGWSG 1

GCCATTCTTAGTTGCGTTTGT AILSCVC 1

TGGGGGCTGAACTATAAGATC WGLNYKI 1

TTGCAGAGGGGGGCCAGGCGT LQRGARR 1

GGGGAGTGCAGTTGTGGGCGT GECSCGR 1

CGGGTGGGTTTCAGCTGGGAT RVGFSWD 1

GAGGTCGGTTTGAGGCGGGAT EVGLRRD 1

GACCCTGTCGCGTGGCGTGCT DPVAWRA 1

GACCGGCGTGTCTCGTGGCCT DRRVSWP 1

ATGAGCACGACGTTCTTCTCC MSTTFFS 1

AGGGGCTGGGGTTGCTGTCCG RGWGCCP 1

ACCGCGGCGTCTTCGGGGTCT TAASSGS 1

TGTCGCGGCCTGGGGCGTCTG CRGLGRL 1

GTGGTTAGCAGCATCTCTGAG VVSSISE 1

GTGCCTGATGTGTGTAGGCCT VPDVCRP 1

TACGGCACTGGCGTGTGCTCG YGTGVCS 1

ATGATTGGCCTCACGTTCGCT MIGLTFA 2

ATTGCGGCGGCTTTCCTTTCG IAAAFLS 1

GCCAAGTGGCCCATTCTGTCT AKWPILS 1

CCTATTGTCAGCACTCGTGTG PIVSTRV 1

TGGCCGTACTGGTACGTGCAG WPYWYVQ 1

CCGCCTCTGGAGTTTTCTCTT PPLEFSL 1

GTCGGGGGCGTCAGGGTTCGG VGGVRVR 1

AGGCCTTCGAGGCTTTTGAGC RPSRLLS 1

TGCTTTGAGGGTGGCTGGATT CFEGGWI 1

CTCTGCTCTGCGATCTCTGAG LCSAISE 1

TACTGGTGCTGGCAGAACTCG YWCWQNS 2

AGTGGTGTGGTCATGCGGAGT SGVVMRS 1

GGGTCGCGGGCTGTTGAGCCG GSRAVEP 1

GCTGAGCGCTCGGACGGTTCT AERSDGS 1

GCGTGCTTCAATTGCTGGGGG ACFNCWG 1

TTCGGCATGACTCGGGCCTTT FGMTRAF 1

CCGCGGCCCGAGATGCTTATT PRPEMLI 1

TACGCTCGCACTTGGTGCGCT YARTWCA 1

TATCGGCAGCTTCGGGGGCTG YRQLRGL 1

GAGAATGTGGGGGAGGACTTC ENVGEDF 1

TGTCAGCGGTATTCTGGGGAT CQRYSGD 1

AAGTGGCAGTATGGGTTGTCT KWQYGLS 1

TGGTATTCGCTCGGGTTTCTT WYSLGFL 1

TGGAGGGTCCAGTCTGACGGG WRVQSDG 1

CATACGGTCTCTAGTCGTCCT HTVSSRP 1

CGGGATGCGTGCGGGCCTCGT RDACGPR 1

TACAGTCCGTCCGCCGCGAGC YSPSAAS 1

GTGGTCCTTGTGGCCGGGCTG VVLVAGL 2

GAGGCGTCGGATTGCTCTAAT EASDCSN 1

GTGGGGAGCGACGTCTGCCTG VGSDVCL 1

TTCCGGGGCAGGCAGCCCCGT FRGRQPR 1

CAGCGGGCGAGTGTTGGTCTG QRASVGL 1

CGTCCTGCCGCGCGGGTCGGC RPAARVG 1

ACGCTGCATACTATTCGGCAT TLHTIRH 1

TGCTCGGGTTCCCTCATTACG CSGSLIT 1

CGGTCGTTGGGCTCGATCGGC RSLGSIG 1

AGGTGGCGCGGCCGGACGTTG RWRGRTL 1

CCGGGGCTCCTTGGCTTGCAG PGLLGLQ 1

GCGCTGGTCCCCTTCCTTATT ALVPFLI 1

GTTAGTCTTCCGGTTCGGGTG VSLPVRV 1

CACCGGGGCATGGTGCCCGTG HRGMVPV 1

AAGGTTGCCGTTGCTAATCGG KVAVANR 1

TACCCTTGGCGGATGAACTGG YPWRMNW 1

GTCTTTCTTCTCCGCATGGGG VFLLRMG 1

GCGCCCCGTTGCGGTGTTTTG APRCGVL 1

GATCTCGGGGCGCCGGGGACT DLGAPGT 1

AATCGCGAGACTCGCAGGTGT NRETRRC 1

TGCTCCATCGGGGATCCGGTT CSIGDPV 1

TGGTGGCCGGCGACTCAGATT WWPATQI 1

AAGGATCCGGCGGGCGCCGCG KDPAGAA 1

CGCTGTCGCCTCATGTGGCTT RCRLMWL 1

GGTGCGTTGTTGATGTGCGGG GALLMCG 1

GTGCGGTGGATTAAGCGGTTT VRWIKRF 1

TACGCTGGGTTGCGTGCGTGT YAGLRAC 1

GATTTTCATCGGCTGTGCGTT DFHRLCV 1

CATGCCGTGCCTCTTCTGGCT HAVPLLA 1

GGCTGTAGCTGCCATGGGGGT GCSCHGG 1

CTCGTTCTGATGGCCGGGACG LVLMAGT 1

AATTGCGCCTTGCCCGAGACT NCALPET 1

GTGTTGGTTGGTCCGCGTCCT VLVGPRP 1

GGGGCGGCCCAGTGTCGGTCC GAAQCRS 1

CGGGTTTGCGGCGGGCGGGGG RVCGGRG 1

GGGGCTTGTTGCCAGGGGGGG GACCQGG 1

CTTGGCGAGTGGAAGCAGCTG LGEWKQL 1

GGCATGGCCCTGTCCGAGGTT GMALSEV 1

CGTGCTGTTGAGAGGATCCTT RAVERIL 1

AAGGTTCGCGCGAGTCGGGGT KVRASRG 1

CATCGGCTGTACAGCTGCTGG HRLYSCW 1

GATGCGCATAGCTTGCTTCCG DAHSLLP 1

CTGCTGCGTGCTGTTGTGTCT LLRAVVS 1

CTGTGGACGGGTTGTTCGTAT LWTGCSY 1

AGTGCGTACACGTGCGGCCTG SAYTCGL 1

GGGCCTCCTGCGTTTATCGGT GPPAFIG 1

TGGGAGGGGAGTGGCGACTTG WEGSGDL 1

TGGGAGAGCGCTGGGCGCGAT WESAGRD 1

CGCATCCGGAGGCAGCTTGCG RIRRQLA 1

TTGTATATGTTTACTAGTCCG LYMFTSP 1

GACGAGTGGGGGGAGTCTGTT DEWGESV 1

CTGCTGGGTGGTATGGACGGC LLGGMDG 1

GTTTGGGGTCCCGACGCTAGG VWGPDAR 1

ACCCATCGTTCGCCGTGCGAT THRSPCD 1

TGGACTGGTGGCGCCCCGCTG WTGGAPL 1

GCTACCTACCCTTGGCCGCGG ATYPWPR 1

AGCTCTAGCCGGTCCATTCGG SSSRSIR 2

GCGCGCGACGTGTACCGTGTG ARDVYRV 1

TGGCTCGTGGTCAGCGTGTTT WLVVSVF 1

GTTCCGACTGCCAGGTCGACT VPTARST 1

GTCTGCGTGCTGCGGTTGTCG VCVLRLS 2

TCGCGGAAGGGCTTCGAGCGT SRKGFER 1

TATTTGGCTTTGAGTTCGAGT YLALSSS 1

CCGTCTGGGATCGTCACTAGT PSGIVTS 1

GGGCGGAGCGGGTGGTATGCT GRSGWYA 2

AGCGCGGGTTACGTTCTTTCC SAGYVLS 1

TGCCTCTCTGGGTCTTGCAGT CLSGSCS 1

CATCGCCAGTGGCGCGGGTGG HRQWRGW 1

GAGTCGCGGAGGGTGAGGCAT ESRRVRH 2

TTCTCCACCGCGTGCCGGCGT FSTACRR 1

TTGTCCTGCATGGTCGCGGTT LSCMVAV 1

GGGGTCCGTTACAAGTCCATG GVRYKSM 1

GGGCCTGGGGGGGCGTGCAGG GPGGACR 1

GTGCATGTCTGGCGTCTGGGG VHVWRLG 1

GCTCGCCCTGGGGACGTGGAG ARPGDVE 1

AGTAAGCGTCGTTTGATTGAT SKRRLID 1

GCTCTTCTTCAGCATGATCTG ALLQHDL 1

CTGTGCGGCTTGGTGCGGAAT LCGLVRN 1

GTTTGTGAGACTCCTGTGTCC VCETPVS 1

CTTACGGCTTCCCTGCCGTTG LTASLPL 1

AGGGCGGATGGCGAGATTAAG RADGEIK 2

TCTTGGGCTCGGGAGGCGTTT SWAREAF 1

CTGGGCGCCGTCCCTAGGCGG LGAVPRR 1

CTTGGGGAGTACCAGGGGGCT LGEYQGA 1

TGGTGGCGGTCCGTTGATGGG WWRSVDG 1

GCGCGGAGTTGGCTCACGCCC ARSWLTP 1

GACAGTGGGCGGTATTTTGCT DSGRYFA 1

CCTGAGTCGGGGTTGTCGCGT PESGLSR 1

GGTTCGGTCAGCGTTTCGCAG GSVSVSQ 2

TCTTTTGCTCCGCTGCGGCTG SFAPLRL 1

TGTGCTCGGAGTCTGAGTACT CARSLST 1

GCCAGGAAGTCCAGCCGGAAT ARKSSRN 1

GGGCGTCGGCACGGGGCCGGT GRRHGAG 1

GGGGTGGAGCCGCGCCGCCGG GVEPRRR 1

TTTATGCTGCCGGACGCTTCG FMLPDAS 1

ATCCCTTCCGCGTCCCGCGCG IPSASRA 2

TTGAGCTGGTGGCTCGTGCCT LSWWLVP 1

GCCGGGCATATGCTGGCCATG AGHMLAM 1

GCGCGCATGTGCTTTACCGGT ARMCFTG 1

GTCCTGCTCCATATGGGCAGG VLLHMGR 1

TACGCTGAGGTTCACAACGAT YAEVHND 1

TGGCGGGGGTTCCGTTTTAAG WRGFRFK 1

TGTCGCGCTAGGTATGCGTGG CRARYAW 1

GGCGTCCAGCGCGAGGGTAGT GVQREGS 1

GTTTCGTCTTGTACGGTTAAC VSSCTVN 1

TATCGGGTCGGGACCTGGCGG YRVGTWR 1

GATCCCCCGCTCACCATTCGC DPPLTIR 1

AGTTTTTTGCAGGCGGGGCTT SFLQAGL 1

TGGCAGGCCACGGAGGTGGTT WQATEVV 1

TATAGCGTTAGCTCCATTGCG YSVSSIA 1

TTGAATCGCTTTGAGTGGGAG LNRFEWE 1

GGTTTGTCGGACCATGGTCTT GLSDHGL 1

TTTGGTGCGTGTGGCGCTGTT FGACGAV 1

TTCAGGTTCGTTGTTTCCACC FRFVVST 1

GTTTTTCGCACGGCGCAGTCC VFRTAQS 1

CGGTTCCGGGAGGTTTTCGGG RFREVFG 1

GCTGGGACTCCGTTGCGTCGT AGTPLRR 2

TTGGGTCGGGTGGCGGACATT LGRVADI 1

CTGGATGGTATGGGCTCGTCG LDGMGSS 1

CTTTCCAAGAGGTGCGTTGGC LSKRCVG 1

GTGTTCTTGTGCCGTGCGGGT VFLCRAG 1

CCGCTGGGCTCGTGGGAGAGT PLGSWES 1

CGGCGGTTTATTAGGTTGTTT RRFIRLF 1

GTGGAGTGTGGGTGCCGTCTG VECGCRL 2

GCCGATTATTGGAGCTGCGTT ADYWSCV 1

CACTCGTTCTCGCGCGCGTCG HSFSRAS 1

TTGTGGTCGGCGGCTGCGGAT LWSAAAD 1

CACCTTTGCTGTGCTGGGCTG HLCCAGL 1

GCGCTGGGGCTCACCCGTGTG ALGLTRV 1

TATGCGTTTGGTTGCACGTGG YAFGCTW 1

TTCCACCACGCTATGGGTCTG FHHAMGL 1

CCGAGGCGTTGGACTCCGGTC PRRWTPV 1

GTGGCGCGGACTTACGGGAGG VARTYGR 1

GCGGGTGGGGGTTGCTTGATG AGGGCLM 2

AGCCTTTCGAGCCGCGGCCTC SLSSRGL 1

ATTGCGCAGGCCAGGGTCAGT IAQARVS 1

AACGAGGTCTGGGGTGTCTCG NEVWGVS 1

GCGTGCCGTGGTGCTAGCGGT ACRGASG 1

CCGGTGTCTTCGGGCCATACG PVSSGHT 1

GTGCGCCGTTGTATGGTGGTT VRRCMVV 1

TTTTACCGGACCAAGTCTCCT FYRTKSP 1

GGCGCTAGGTGCGCCCTCAGT GARCALS 1

GTGCGTGGGTTGCAGCGCGGT VRGLQRG 1

ACGTATTGCGATGGGGAGAGT TYCDGES 1

GGGGTTTTCGCTGCGCTGCCG GVFAALP 1

AGTCAGTTTAGGGCGGGCTCG SQFRAGS 1

TTCTGTGTGACGCTGGGTGAG FCVTLGE 1

TCCAGGTCCGAGCGCCAGGCT SRSERQA 1

TGGCGGTTCACGCGGGTGGCG WRFTRVA 1

CGGGCTCCTCCGCCGTTCCTT RAPPPFL 1

TTCAGGACTCTTGTCGGGCTG FRTLVGL 1

GAGAGCCGCGCTATCGCGTGG ESRAIAW 1

CCCGCTCCCGGTATGGTGCGG PAPGMVR 1

CTTGGCGCTCGGAATCTGTGT LGARNLC 3

AGTGGTGTCCGCGGTAGTCCT SGVRGSP 2

AGCGGGATCACGGGTCAGCGG SGITGQR 2

GCCCGCTGCCGGATGAGGCTC ARCRMRL 1

GTTGGCCATTGCACTCTCTGT VGHCTLC 1

GGGCCCGAGCTTGTCGCTTCT GPELVAS 1

TTCATGAGGGCGTGTGCTGAT FMRACAD 1

GCGCTCTGGTCTCCTTTGGTG ALWSPLV 1

GGCTCGCTCAGTGCTGGGGCG GSLSAGA 1

GCGCTCCTGGCGAATGACTGT ALLANDC 1

GAGAAGATTGGTCGGTACTTT EKIGRYF 1

GCTCTGGGCGGTGGTGGTTAT ALGGGGY 1

TTTCGCGAGCGGTGGGCGTGT FRERWAC 1

CGGTCGGATTTGGGTCGGGGT RSDLGRG 1

GTGGACCGCCGTGACGCGTGT VDRRDAC 1

TGGCCTAACCTTAGCAGTCCG WPNLSSP 1

GTGTGGTGCGGCGGGGCCTCC VWCGGAS 1

ACGTGCGGGACTGGTGTTTCC TCGTGVS 1

TGTGTGTCCTCTCCGGGGAAT CVSSPGN 1

GACTGCTTCGCGGACTGGTGT DCFADWC 1

GGCCGCTGCCCGGCGCTTGGC GRCPALG 1

TGGCCTCTCCTCAGCGGTGCT WPLLSGA 1

CCGTCGTGGTGGCGTTTGACT PSWWRLT 1

GACGGGAGCACGGATCAGCAG DGSTDQQ 1

TCGTGGGGCCGGCTGAAGTGG SWGRLKW 1

GACGATTCCGGTGCGATGAAT DDSGAMN 1

TATCGGGTTTATACCATGTGT YRVYTMC 1

GCGGGTTTGGGTGTGGAGGGG AGLGVEG 1

GATAATAATCTGTCGGTTCCT DNNLSVP 1

GGGAAGCGTGGTGATCTGGGT GKRGDLG 1

TCCCGCTTGCTGGCGTTGTGG SRLLALW 1

GTGGGCTGGGCGAACATCAGT VGWANIS 2

GGCCAGCCTAGTTTTCTTCAG GQPSFLQ 1

TGCCAGGGTAGGGCGCTTATT CQGRALI 1

GCGGAGGGCAGCAACGGCCTC AEGSNGL 3

ACGCTGCTCAAGAGGATGGAG TLLKRME 1

GCGCGGTTCGCGGTCGTCCTG ARFAVVL 1

GTGATTCGGAACAATTTCGGG VIRNNFG 1

CTGCCGCACAGTTGTGTCCGG LPHSCVR 2

GTGATGCGCTACCGCCTTGTT VMRYRLV 1

TCCGTGGCTCGCGGGTTGTCG SVARGLS 1

GATGCGATCAGTTATAAGTGC DAISYKC 1

TACCAGATTGGCTGGGTGCCT YQIGWVP 1

ACTAGGCGCAAGCTTAACTCT TRRKLNS 1

CATACGGAGAAGTACTCGTGT HTEKYSC 1

TCTGCGAGGATGGGCTATTGG SARMGYW 1

CGTTGGCGGGAGCGCGTTAAG RWRERVK 1

GGCCGTCTGTACTGCGCCGAG GRLYCAE 1

CATATGGTGCTCGCCGCGACG HMVLAAT 1

GCGTGTGAGCTGAGGAGGCAG ACELRRQ 1

AATCGGTTCGAGCGTTGGACC NRFERWT 1

GGGTTTGACCCGATGTGTCAT GFDPMCH 1

ACGCTCGTTCTCGGCGGGTGC TLVLGGC 1

GGCCGTCGGTGTCTTCGGGCT GRRCLRA 1

CACGAGCAGGACTCGGGGTCT HEQDSGS 1

TATTGTAGCTTCTTTCCTACG YCSFFPT 1

GGCCGTATTTCTCTCGGTAGT GRISLGS 1

AGTTGGACCGGCTGCACCAGT SWTGCTS 1

CCTGGTTCGAATGTCGAGCGG PGSNVER 1

AGTCTCATCTGGCAGATTACT SLIWQIT 1

GGGTCTGGGCGCACTCACACG GSGRTHT 1

CATGTGTGGATTTGGTGCGCT HVWIWCA 1

AATGGGTGTGGGGATCCGCCT NGCGDPP 1

TGCTGGTGGAGCGTCTCCGTC CWWSVSV 1

TGCCGGTGGTGTGTGGGGTTG CRWCVGL 1

GTGGGGCTTCTCAGGAGGGAT VGLLRRD 3

CGGATTTGGGGGGTCTGTCTT RIWGVCL 1

AAGTGTTTCAGGCACGCGATG KCFRHAM 1

TGGCGGGTGACGCGGATGTGG WRVTRMW 1

GCGTTGGCGATTGCGCGTTGT ALAIARC 1

CGGATGCTGTTCTGTGGGAAC RMLFCGN 1

TTGCACTATATGCGGGGCGTT LHYMRGV 1

TCGATTAACCACGGTGACCCT SINHGDP 1

CAGCGGGTGTTGGTCGGGCCT QRVLVGP 1

CGTAGGGTCGGGTTGGTCAGG RRVGLVR 1

ACGGTGAACATTGGGCGCTCG TVNIGRS 1

AGCTATGTGCGGTCCGGGTTT SYVRSGF 1

GGTCCTTGCTTCCGGGGGCCG GPCFRGP 1

GTTGGCCTCTCTATCCAGGCG VGLSIQA 1

GTCGGGGGTAGCTCTCGCAGT VGGSSRS 1

GCCGGGGCCGGTGGTGGGATT AGAGGGI 1

GGGGTTGCCAGGCTTGGGTCG GVARLGS 1

GGGGGCCACTGCAACCGTTTT GGHCNRF 2

GATCACGCGACTGCCAGGCAG DHATARQ 1

ATCGGTCGGAGTGCGTGTGCT IGRSACA 1

CTCGTTGGTTTTAATGGTTGG LVGFNGW 1

AACGCGGTCTCGGGGGATCCG NAVSGDP 1

GTCGATTCCTCCCTGGGGCTG VDSSLGL 1

TTCGCGCGTAGTGGGGCGTCC FARSGAS 1

GCGAACGTCTGTCGGCAGCAT ANVCRQH 1

TCGGTGTGCAGTTGCACTCTG SVCSCTL 1

TCGCTGTTGTGTAGTTGCTCT SLLCSCS 1

GCCTCCTCTGGCGCTTTGGGT ASSGALG 1

GATTCCGGCCACTGCGATACT DSGHCDT 1

CTCGTGATCACGTGGAATATC LVITWNI 2

GGGGACAAGCAGGCCGATGAC GDKQADD 1

TGTTGTCCTTTTGAGGCGACT CCPFEAT 1

GAGGCTTATTTCCAGGGCGCG EAYFQGA 1

TTGTGCCAGCTGTGTGGGGCG LCQLCGA 1

GCGGTGGAGGGCGGTTCTAAG AVEGGSK 1

TGTGGTAGTTGGAAGCGGGTT CGSWKRV 1

GGGCGGTTCGCCCGCAGTTAT GRFARSY 1

AGGGCGCTCGTTCCCGCCGAG RALVPAE 1

TGGGGCTCCGACCGGAACGCT WGSDRNA 1

TCGTTTGGGCTTCGGTCTGCG SFGLRSA 1

CCGGTGTGTACTCAGTGGATT PVCTQWI 1

TGGGGGTTGAGTTCGGAGCAT WGLSSEH 2

GTCCACGGCACGAACTTCTTG VHGTNFL 1

GTCCGCGTCCAGGTCGGCTCG VRVQVGS 1

GGGCCGGCTGTGCCGTTGTCT GPAVPLS 1

TCTTGCACGAGGTGGGCTGTG SCTRWAV 1

GTGCAGTATCGCTGGTACCCG VQYRWYP 1

GTCCGGTGGGGGTGTCTTGAG VRWGCLE 1

TTGATCCGGGCCGATTCGCCT LIRADSP 1

CTGGCGTCTCGGTGCAGTTTG LASRCSL 1

CAGGGTGCGACGAGTTGTCCC QGATSCP 1

CCTGGCCGCCGGACGAGCTTC PGRRTSF 1

AAGCGCGGTTTGCTGTCTTTG KRGLLSL 1

GGCGGGCCCAGTTGGATCTTT GGPSWIF 1

CGTAACCAGGAGTGCCTGGTG RNQECLV 1

ATCGGGAGCACGGATTATGCG IGSTDYA 1

TGCTGCTGTTGGCGGGGGCGC CCCWRGR 1

GGGCCTGGGCGGTGCGATCCT GPGRCDP 1

GTTTGTCTGATCATCCGTGCT VCLIIRA 1

GCGGGGAGGCTCTACGCCATT AGRLYAI 1

CGTGGGTCCTGCACCAGCGAT RGSCTSD 1

AATGAGGGTATTACTGTCGGG NEGITVG 1

TGGTGTGAGAGGTATTGGCTT WCERYWL 1

GCTTGGGGCGCGCGCCTCGTG AWGARLV 1

GTCTATCGGTCGCGCGACCGG VYRSRDR 1

GGGGCGTGTTCTGTCTACGCG GACSVYA 1

GTTGGTTATTGGCGGATGACC VGYWRMT 1

TGCATCGCCTGCCGGTTGTCG CIACRLS 1

TATTCTGACGGCCCTTTGCGG YSDGPLR 1

GCCCTGGTGATCACGTTGGGC ALVITLG 1

GTGCGCTGCGGTGCTCGTGCG VRCGARA 1

CTCAGTGGGACTTCCCGTCCT LSGTSRP 1

GCTAGGCAGGGGCGGTACGGG ARQGRYG 1

GGGCGTTGCCGGGCTACTGCT GRCRATA 1

CCCGAGTTGGTCAGCCATGTC PELVSHV 1

ATGCAGGGCGGTTTCCGGTTG MQGGFRL 1

CAGACGGGCGTGTCCAGGGGG QTGVSRG 1

TCCGGGCTTAGCGTGGGCGCT SGLSVGA 1

AGGAGTGTGGCGGGCGCGGTT RSVAGAV 1

TATAGTGCGACTGGGCTGGCG YSATGLA 1

GTTTGGGAGAATTTGGGTTTG VWENLGL 1

GCTTCGCGGTCGTCCTGCGGT ASRSSCG 1

AGTGCGCAGTGGTGGCTGAGG SAQWWLR 1

ACCACTGGTCGCTACATGTTT TTGRYMF 1

GCGGTCGGTTGGATCTATTAT AVGWIYY 1

ATGTTTCAGGCCGAGGTGTGG MFQAEVW 1

CTTAGTTATCATTACTGTCTG LSYHYCL 1

GGGAGTTGCACCTGGGTGGTT GSCTWVV 1

GCGGATGTTCTGGTTGATAAG ADVLVDK 1

CCTGAGGTGAAGCGGTTCGTT PEVKRFV 1

CCTTGGCAGAACGGGGCGACC PWQNGAT 1

TCGCCGTATTGTGGCCTTCCG SPYCGLP 1

TGGCGGATCCAGGCCTGTTCG WRIQACS 1

GGCTTGGCGGTCCGGCGCAAT GLAVRRN 1

GCGGCGGGTCTGTGCATGCTG AAGLCML 1

TATGGTTTGGACCTGCAGCTG YGLDLQL 1

GTTGCGCCGGCGGGGAACTCG VAPAGNS 1

CGGGTTCGGATCGGCCAGAGG RVRIGQR 1

GTCGGGGAGATTGGTAGCTGG VGEIGSW 1

AGGTGGTTCAGCCGCGATGCT RWFSRDA 1

GGGTCTACCCCGATGGGGGAT GSTPMGD 1

CTCAGCCTGTGGATGTCCGGG LSLWMSG 1

GTGATGCTTTATATCAGCGTC VMLYISV 1

GGCGCCCGGGCCATTAACGCG GARAINA 1

GTGGGTACCAGTCTGCTCTTG VGTSLLL 1

CCGGCGGCGAGTGAGAGGATT PAASERI 1

TCGACGAGCGAGTTGCTGCCG STSELLP 1

GCTTATAACTGGCTGCATCTT AYNWLHL 1

TTGATGTACAGTAATATGGCT LMYSNMA 1

TCTCGGGATCCTGAGTGGTCG SRDPEWS 1

GTGCGCGGGGCCCGTTATCGT VRGARYR 1

ATCTTTCATCCTGACATTGCT IFHPDIA 1

CGCTGGCCCTGCGTGGGCGCG RWPCVGA 1

TGTCAGTTCACCCAGCCTGGG CQFTQPG 1

TCTGTGCTGTACCGGGGGTCG SVLYRGS 1

GGCATTGTGAATTACTCGCAG GIVNYSQ 1

GATAGTTCGAATTCCGTTTGT DSSNSVC 1

GGTGTGGGCCTCCTGCAGCGT GVGLLQR 1

CTGGGTGGCGCGCCGACGGAT LGGAPTD 1

TGCCTGTCTGTCCCTTTCGGT CLSVPFG 1

CGGCTGTACAATATTTGTGTG RLYNICV 1

TGGCGCACTGTGTATCGTAAT WRTVYRN 1

GCTGCTGCTGGGAGCGATCCT AAAGSDP 1

TTTCAGATGGTCGGCGGGGTT FQMVGGV 1

AGCAGGTTCCATCTCCCCCAG SRFHLPQ 1

GCCGATGATCTGCATAGTCCC ADDLHSP 1

ATGGAGGAGATCTCTGGCTCG MEEISGS 1

GTTGGGCAGCTCGATCATGTG VGQLDHV 1

AGCAGCATGTCGGGCCACTTT SSMSGHF 1

TGGCAGTGTTTGAACATGTAT WQCLNMY 1

TACTGGTTGGGCGTGCGGATG YWLGVRM 1

GGTTACTGCGCGGGCGCGTGT GYCAGAC 1

TTGTTCTATACTGCGGCTCAT LFYTAAH 1

TCGGGCCGGCCTTACGATCGT SGRPYDR 1

GGTATTCACGGCCTGGGCTTT GIHGLGF 1

CTCGGGGTCCTTGTGGTGCCC LGVLVVP 1

GGGTCGGCGCGGACTCCGGGG GSARTPG 1

AACCGCCACTTCGAGGATTCT NRHFEDS 1

GGGCTGCGGCTCTTGGCTGAT GLRLLAD 1

TCCTCGTCGCGGTGGTGTGGG SSSRWCG 2

GCGGGCATTGGCACGGGCTTC AGIGTGF 1

TGTTGGGGGTGGAGCCGGAGT CWGWSRS 1

GGGAGGTCGGATAGCCGGTTG GRSDSRL 1

TCGGGGGACGTCGGGGGCGAG SGDVGGE 1

GGGCTGATTTGGTATATTTAC GLIWYIY 1

TGCAATGCTTCTGCGGTCGCT CNASAVA 1

CCTGCTGCCGAGTGGGAGTTC PAAEWEF 1

ATTTCCGCCGGGGTGGTGCGG ISAGVVR 1

ATCTTGTGTATTCGTAATAGT ILCIRNS 1

AAGCTCCTCGAGGGTCTCGAG KLLEGLE 2

TATCTCAAGCTGTGTAGGGGT YLKLCRG 1

GGGCCCTTGGGGGAGTCGCGG GPLGESR 1

TATCACCAGGGCGCCGGCCAT YHQGAGH 1

AAGGCGTGGCGCAGCGAGGTT KAWRSEV 1

GGGTATTCCAGGTGCTCCCGT GYSRCSR 1

GTGTGGGGTGTCCGCAGTGCT VWGVRSA 1

GCGCGGCGCTGGAGCGGGGTG ARRWSGV 1

TGGAAGCCGGTGCTCGCCAGT WKPVLAS 1

CCCCACTCGTGCGGTGGGCCG PHSCGGP 1

GCTCGCCTTGGTAAGCTTCCT ARLGKLP 1

TGGTGCGTTTGCCTTTCTGTT WCVCLSV 1

GACCGCCGGCTGCACCCTAGT DRRLHPS 1

TTTCTCTTGGGCACGCCGGCG FLLGTPA 1

TCCTGGTCTCTGATTCTTGGT SWSLILG 1

CTCTCGGGCGGGGTCCACCGG LSGGVHR 1

CTCCGGGAGACTGCCGACACT LRETADT 1

TGCCTCATCTCTTCGGAGCCG CLISSEP 1

CGGGATAGGAAGTTGAGGATT RDRKLRI 1

TTCGGTCGTCCGAGGGCTGGT FGRPRAG 1

CCCAGCTCCCTCTCTCGTGGT PSSLSRG 1

CGGGATGTTCGTGCTTTGTAC RDVRALY 1

GTCCGGAGGCACGGCGGGAGG VRRHGGR 1

GGTAGGATGTGCGACCTGGCT GRMCDLA 1

GGCGCGGGTCACGCGGCCGGC GAGHAAG 1

TCTATTCTTGTTCTTATGTAC SILVLMY 1

GATTATGAGGACTTTATCGTT DYEDFIV 1

GTTAACGTCCCGGTCCCGACT VNVPVPT 1

TACTCGCATTTGTCCTTTCCC YSHLSFP 2

GACGAGGGCTTGGTGAGCTCT DEGLVSS 1

AGGTTGCTCCGGAAGCTGACT RLLRKLT 1

CACCATCGCGCCACGGTTCCG HHRATVP 1

CTGCGTTTCGATGTGCTGTTT LRFDVLF 1

CTGTATACCGTCGCCCGGACT LYTVART 1

GGCGTTGATGTGGCTCCGGGT GVDVAPG 1

TGGCGTTGCGTTCAGCAGTAT WRCVQQY 1

CTCAGGCCGTGGATTTGGTGG LRPWIWW 1

CTGTCCGATGTTGGGTCGAGT LSDVGSS 1

TTCGGCTTGAGTGTCAGTACC FGLSVST 1

ATGGAGCGCTACTCTAGCAGT MERYSSS 1

GGTCGCGTCCAGCCGTACTGT GRVQPYC 1

ATCCGGTTTAATCTCAATCGG IRFNLNR 2

GACCGGCACTGCGTTTCTTGT DRHCVSC 2

GGGTTTCATGTTAGGCGTGCG GFHVRRA 1

GTGCCGCTGAGTCGGTCTTTG VPLSRSL 1

GCCATGGGCGTTTGGGGTGGT AMGVWGG 1

CTTTCCGATATGCCGCGGTAT LSDMPRY 1

CCGCGCCAGTGCCGTGGGACT PRQCRGT 1

TCTTGGTCCGCTTGCCTCGCG SWSACLA 1

GTTGTTGGGTTGATGTGGTGC VVGLMWC 1

TCGCACCTCACTTGTGTCCTG SHLTCVL 1

CAGCGTAGCAAGCGGACTGCT QRSKRTA 1

GTGCGGTCCTCGTTGGTCACG VRSSLVT 1

GTCTGGAGGATCGGCCACATG VWRIGHM 1

GACGTGGTGTGTTCGTGGCGG DVVCSWR 1

AGCGAGCATATCGGGTTTTGC SEHIGFC 1

TTGCGCTGGGCGGCGTATAGT LRWAAYS 2

GTGGTTGACCGTCGCCTGTGG VVDRRLW 1

ATCGCCCTTTCGGACTGTTAT IALSDCY 1

ATGAATTGGCTTGAGAATCGT MNWLENR 1

TTCCATGGCCGGCTGCCTGCT FHGRLPA 1

CAGCGCGGCAGCCCCATTCCT QRGSPIP 1

CGCGTCGCGCGGGTTCAGATT RVARVQI 1

TGGGGGGCGGTTGCTTCTCGT WGAVASR 1

CAGTTGGTCATTGACATTTCT QLVIDIS 1

GGGCTCGTGTCGGGTCACGAT GLVSGHD 1

CGTGGTCGTTCTGTGCGGACT RGRSVRT 1

CAGTGTCCTTGTGGGTCGGCG QCPCGSA 1

TATGGGGCGTGTGTTCGTACT YGACVRT 1

GGTACGGTTATCGGTCCTTGG GTVIGPW 1

ACTCGCTGCGCCCTTCGTCCG TRCALRP 1

TATGGTGAGCGGAGGCATTGC YGERRHC 1

GCCCAGCGCGTGTGGAACGGG AQRVWNG 1

TCGCACTCTGGCATTCTGATT SHSGILI 1

ACCGAGGGGCTCAGGTGCACT TEGLRCT 1

GAGGTTGTCCGCGCGGCTGTT EVVRAAV 1

GGGGTTGGCAGGGCTTGGGAG GVGRAWE 1

CAGTCCCTGTCGTGGTATTCT QSLSWYS 1

TATGCGATCATTCTTAAGCGT YAIILKR 1

GAGGTTCCTGGTGTCTTTAAT EVPGVFN 1

TCTACCTACTACCGTCTCCAG STYYRLQ 1

GCCCGGAGCTCGGTCGATTGT ARSSVDC 1

CGGTATCCCGAGTTGGCGACG RYPELAT 1

GTTGAGCGGAGTTCCATTACT VERSSIT 1

CGGCTCTCTAGGAGCCGCTAT RLSRSRY 1

AGGTGTTTGTCGAAGGTGGGT RCLSKVG 4

TCCATTGGCAGCGGCGCGCTG SIGSGAL 1

GCTACGGGCGAGTCCATGTAC ATGESMY 1

TTGCAGCGGTATCTGGTCAGC LQRYLVS 1

GATTGTATCTCGAGGTGTTGC DCISRCC 1

GCCTGGTCTAGGCTCAACGCC AWSRLNA 1

TTTATGCAGCTCGCCGTGGTG FMQLAVV 1

CACATGGATGGGAGCCCTTGT HMDGSPC 1

TCGGTTATTTGGGCGGAGCGG SVIWAER 1

TCCAAGGGGGTCACGGGCGTG SKGVTGV 1

GTGGTGGTGAACTTGCTTGTG VVVNLLV 1

CGTGTGAGCGACCTGTTGCGT RVSDLLR 1

TACATCCTGGCCCTCGGCGAG YILALGE 1

CGTGCGCTTGTTACTCTGGGT RALVTLG 1

GTTTCGGGCGAGTCGTGGACT VSGESWT 1

CAGGGCTCTATGGGGCAGTGG QGSMGQW 1

CCCGGTCTCGTCGCTACCGCG PGLVATA 1

GTTCATTCGCGGAATCTTTCG VHSRNLS 1

GTCCGTTCGCGTATCTGTATC VRSRICI 1

AGGTGGGGTATGTTGCGTCCC RWGMLRP 1

TATATCTGTTTGTGGTGGGCT YICLWWA 1

TTCAGCTCGCTCGCTATTGGC FSSLAIG 1

TTGGCTTATTTGCGGTACTTG LAYLRYL 1

TGCGCGCGGTATCGGCCTGTT CARYRPV 1

ATTTGCGAGTCGCCTGCCTTT ICESPAF 1

GCGGGGCGCGGTTTGCATATC AGRGLHI 2

GTGTGGCGGATGAATATCCGG VWRMNIR 1

CTCGGCAGGAGCAACCTCAGT LGRSNLS 1

ACTGATGAGGCCCGTGAGCCT TDEAREP 1

GCTGTGCTGACGGGTGGCTTT AVLTGGF 1

GGCAACGACGTTTGGCTGGAG GNDVWLE 1

GGGCGGGAGACGCTGCACGGT GRETLHG 1

GGTGACGGCTTGTTGGCGGTT GDGLLAV 1

CGGCAGTATTGCAGGGTCCAT RQYCRVH 1

AAGTGTCGGCGCCTCGCGATG KCRRLAM 1

ACCGTGCTCAGTGGGGGGTGC TVLSGGC 1

GGCCTGCGGTGGTTTGGCCTT GLRWFGL 1

TGGCGCAACGTCAGCAGGACG WRNVSRT 1

CAGGGTGACGAGAACTTCTGC QGDENFC 1

AGCGCGGCCTTCTGCGAGGAT SAAFCED 1

GGCGCGCAGAGGCGCATTAGG GAQRRIR 1

AATGTTGACTTTATCAAGCGG NVDFIKR 1

GGCTCCCTTTGGCGTTACGAC GSLWRYD 1

GCTCTGGCCTCTTCCGTGCCT ALASSVP 1

TGCTGCGTGCGTGCGGTGTTG CCVRAVL 1

GGTTACGTGATGCAGCGTACT GYVMQRT 1

GTGCTGTGCGAGGAGTTTGGT VLCEEFG 1

ATGACTCTCGTGGAGCACGGT MTLVEHG 1

TCTAGCGATTGTTGGTCCTCT SSDCWSS 1

GCCGTTAAGAACTACCTGATC AVKNYLI 1

ACCAGGTCCGTGGACGCGCAG TRSVDAQ 1

TGCTTTCGCTGCGTTTGGTCG CFRCVWS 2

TACCGTCGGTTGGATTCCAGT YRRLDSS 1

GGGCTGGTCAGGGGGTGGTGT GLVRGWC 1

GGCGGGCTGCGTGTGGGCTCT GGLRVGS 1

GGTTTCGGTAGGCTCCGGGTC GFGRLRV 1

TACGTCCGCCCGGGGGTGGAG YVRPGVE 1

AGCGATGTTCACAGCAACAGT SDVHSNS 1

GTGCGGGGGTATCGCATGAGG VRGYRMR 1

TCTGAGCCCAATTTCCACCAG SEPNFHQ 1

CGCGGTGCCAACTCTGTTTTC RGANSVF 1

GGTCGTTCTCACAGCGGCGGT GRSHSGG 1

AGTGACTCGCCGATCATTTTG SDSPIIL 1

CGGGATAGCTGCTGTTATGGG RDSCCYG 2

GACATTGACAGGTCGCTCGGT DIDRSLG 1

GTGTCGAGCCGCCATCTGGCT VSSRHLA 1

GTTGTGGTCACGCCTGTTACC VVVTPVT 1

GCTCGTCTGTTTTTCCGTACC ARLFFRT 1

TTTGTGCGCTTCGGGATTGTT FVRFGIV 1

GACCGTCCCGAGACCAATCAG DRPETNQ 1

GCCGCGCCTCCTGTTGTCAAG AAPPVVK 1

TGTTACTCGCGGTGCCGTGGT CYSRCRG 1

CGTGGTTGTGGTTCTTCTGGT RGCGSSG 2

CATATGGGCATTAGCGGCGGT HMGISGG 1

ACCTCGGGCACTAAGTCTGGT TSGTKSG 1

TCGCGGGTGAGTTTCGCTACG SRVSFAT 1

AGTTATGGCACCGACATGTTC SYGTDMF 1

TCCGGTGGTACTCCGTGGAGT SGGTPWS 1

GAGGGCTCGAGGTTTTCTCGT EGSRFSR 1

GTGGGGAGCACGTACGAGCCG VGSTYEP 1

AACCGGCTTAGGCGCCAGCCT NRLRRQP 4

GCTCTCGATCTGAGTTTCGGC ALDLSFG 1

TGTCTGCTGTTGTGTGATCCT CLLLCDP 1

ATGGGGGTTGGCCTCGAGAGC MGVGLES 1

CTGGTCAGTGACGTCTGCACG LVSDVCT 1

CGCTCCTCTTCGCACCGTGGT RSSSHRG 1

TTCCATGTTAGGCGGACTGGT FHVRRTG 1

TTGCGGATCATCGGTGTTCAG LRIIGVQ 1

GATGAGCCTGCCAGTATTCGG DEPASIR 1

GCTTCCCGTGGCTATGCGCTC ASRGYAL 1

TGCTTCGACAGGGGCCATGGT CFDRGHG 1

AGCTTTCGTGGGCGCCAGTCG SFRGRQS 1

TGCGGCGGGACTATGTACTCT CGGTMYS 1

GGCGTCTGGAGTCTTGTGCCG GVWSLVP 1

GTCCGCAGCCGCGTGGAGGCT VRSRVEA 1

CCCTGGTTCGCGCGGGAGACT PWFARET 1

GCGAGTGGTCGCCGGCTGCCC ASGRRLP 1

CCGGCTAATGTGGTGCTTGCG PANVVLA 1

ATGAGGCGCACTAACCTCTCT MRRTNLS 1

GGGTGTCCGTACTGGAATTCT GCPYWNS 1

CAGGTTCGCTCTCAGGCGTGT QVRSQAC 1

CCTGGGGGTCCCGTGGGCTAT PGGPVGY 1

GGCAACGGCGTTGTGGGGGCT GNGVVGA 1

GTGTGTACTAACTTCAATTAT VCTNFNY 1

CGCCATGCCAGGTGTCGTTTG RHARCRL 1

TTGGGGTGCATCCGCAGGGAT LGCIRRD 1

TTCCCGGTTATCCCTTATAGT FPVIPYS 1

TCGATTAGCCGCATCTCTGAT SISRISD 1

TGTGCGCGTTTGTCCTGGAGT CARLSWS 1

GTTTTCGCCCCCGAGAAGCTC VFAPEKL 1

GGCGCGGTTTACGGTATGGCT GAVYGMA 1

CGCGGTTGGGAGCGGATGCCT RGWERMP 1

TCTCGTGTTAGGGGCCTCGCT SRVRGLA 1

GTTCGTTCTGGTTCCATCGTT VRSGSIV 1

TATCAGCAGAGTGTCTCCGTC YQQSVSV 1

CATCACAGGAGTGTGCGGGTG HHRSVRV 1

TCTCGGAGCTTTGGGTCCACC SRSFGST 1

TGCGGCTACCAGCAGCGCTCG CGYQQRS 1

AGCCTGCTTGCGTGCTTGGCG SLLACLA 1

AGCGTGAACCGGGTGCGTCGT SVNRVRR 1

CGCCGCGGCACTCTGGGTCCT RRGTLGP 1

TGGGCTGCTCTCGTCCAGTCG WAALVQS 1

TGGCGGTGCTTGTCGAGGCGC WRCLSRR 1

CAGGGGTGTATTGAGTGCCGC QGCIECR 1

ATTAGTGGGAAGTCGAGGGGG ISGKSRG 1

GGCGAGCGGGGTGGTGTCTGC GERGGVC 1

CGGGGCTACGGCCGGTTGCTC RGYGRLL 1

TGTTGCTCTTGGGTGGCTAAT CCSWVAN 1

GTTCGCGGGAGCTTGGGGCGG VRGSLGR 1

GACTTTGTTACTACTTGGGGG DFVTTWG 1

CAGAGTGTCTTCGGCCGGTCT QSVFGRS 1

CGTAATTCGGCTTTGGTTGTG RNSALVV 1

ACGTTCACGTTGAGCAGGTCT TFTLSRS 1

AGGGAGGCGAGGCGCGCGTTG REARRAL 1

TCCCATGGTCGCGTCAGGATT SHGRVRI 1

GAGGCGGCGGTTTCGTGCGAG EAAVSCE 1

CCCGAGTGCAATCTTCGGCGG PECNLRR 1

GTTCAGTTCGTTGCTCTGAAG VQFVALK 1

CACACGTTTGCGGTCCGGGGT HTFAVRG 1

TGCATGTCGGCGTCTCGGAAC CMSASRN 1

CGTGGCGCCATCATCTTTCCT RGAIIFP 1

GCGCACGTTATCGATCTCAGG AHVIDLR 3

TTCGGTCTTAGCCGTTTTGCT FGLSRFA 1

CGGGAGGAGGTTTGCCGGCTT REEVCRL 1

GTGATGAGGTATCGCTGGCCG VMRYRWP 2

ACGCGGCTGTGGGGGCGTACG TRLWGRT 1

TTCGGCCTCCCGGAGGCGGGT FGLPEAG 1

TCGGATCATGTGTCCGGTTCG SDHVSGS 1

CGGGTGGGGAAGGCGCGGTAT RVGKARY 1

GCGGCTAGGTGTGTTTCTCAG AARCVSQ 1

CTCTCGAGCCGTGCTGAGGCT LSSRAEA 1

TCGCGGCGCCCGTGCGGTAGC SRRPCGS 3

GGCCGGCGTGAGGTGGTCGTG GRREVVV 1

TATAGCGGCAAGGCTGGTAGT YSGKAGS 1

TGCGATGGTACGGCGCGGGCG CDGTARA 1

GTGGATCCGACCCGCACTGTT VDPTRTV 1

AGTGGGGCTCGCGTCATTAGT SGARVIS 1

ATCCGTCTGGTTGGCGCGGGG IRLVGAG 1

ATCCACGCCTGCTCTGGCGAG IHACSGE 1

TTGACGGACAGGCGGCGGTGC LTDRRRC 1

GGCCGGAGCGCCCTCCTGGGT GRSALLG 2

TCGGTTCCGTACCGGGCGGGC SVPYRAG 1

GTGCCTTGTCCCCCTGTTGCG VPCPPVA 1

GCGTGTTCCGTTTTCTTCGGT ACSVFFG 1

CTTGTGGACAGGATTTTGGTG LVDRILV 3

TGTTTCATCCGCAGCTGGTCG CFIRSWS 1

GATGGTGATTCTGCGCAGCCG DGDSAQP 1

TTCCTCCACACTATTGAGGGT FLHTIEG 1

TCCGAGGGGAGTGGTGCTTAT SEGSGAY 1

GTCGAGCGGGATGGCGGGCGG VERDGGR 1

GTGACGTTCCGGAGTGTGGGG VTFRSVG 1

TTTCAGGATTGTCCCAGGTCT FQDCPRS 1

GGTTCTGTCTATCAGATGGCT GSVYQMA 1

CATCCTTATGGCAGGTTTCTC HPYGRFL 1

GCGCCTTGCTGTGTGAGTGGG APCCVSG 1

AGCGCCCGTAGGACCGCTCGG SARRTAR 1

TTGTTTGCGGGGATCCTTGTC LFAGILV 1

CGGCGGGCGCCCTTCGCGCCG RRAPFAP 1

TTGCGGCGCTGCTGCGAGCTG LRRCCEL 1

CGGTCTCGGCCCTCCGCTCCC RSRPSAP 1

ACCATCTGCTGCGTGGAGGGG TICCVEG 1

TTGCGGTTGGGCGCGGCCTTT LRLGAAF 1

GGTTGGTTGGGGTGGTGGCTG GWLGWWL 1

CAGGGCAGTACGCGCATGCGT QGSTRMR 2

TTCACCCTCCGCATGGTTAGT FTLRMVS 1

TTCGCCACTTGTTTGGTTTGC FATCLVC 1

GTGGTCCGCCTCTCGCCTTTG VVRLSPL 1

AACGCGGGGAGGATCGGGTTT NAGRIGF 1

CGGCACGTGGCGCTTAGCGAT RHVALSD 1

GAGACTGGGAGTACGGCTTAT ETGSTAY 1

CTCCCCTGCTTGCTGATGGGT LPCLLMG 2

AATTTGGAGTGGCGGGTTCTT NLEWRVL 1

CTTCCGCACGCGGCCGTGGAG LPHAAVE 1

GACGTTTCGTTCGCCACGAGT DVSFATS 1

TTGCGTAAGAGTAGCTTTAGT LRKSSFS 1

CCTAATTTCTGGCCGAGTCCG PNFWPSP 1

TGCATGCGGATTGATTGGGAT CMRIDWD 2

CGGGTGGGGGTCGGGATGTCC RVGVGMS 1

GTCGCTATGAGGGAGGCTTCG VAMREAS 1

TCTGGGCCTCGGCCGGTCTCG SGPRPVS 1

CGCAACGGGTGTGGGTCCGCG RNGCGSA 1

GCGGGTTCCAGCTCGCCGCTT AGSSSPL 1

AAGAGCGTGCTCGGTAGTCTT KSVLGSL 1

AGGCCCCTTCTCAGGATTTGG RPLLRIW 1

GCTCAGCTTAGCTCTGCCGGG AQLSSAG 1

GTGGGTCGCTGGATTTCGGGT VGRWISG 1

TCCCTGGCCGGCAATGCCAAT SLAGNAN 1

GGCGGTTGGCTTGCGGATTGT GGWLADC 1

GTCGGCTTCAGTTGCGGGACT VGFSCGT 1

GCGGGGGTGACGGCTTTTAAC AGVTAFN 1

GGGTCGTTGTTGGCCGGGGCG GSLLAGA 1

TTGGATCCGCTGGTCTGTGTT LDPLVCV 1

GTTTCCGCGCTCAATGGGCGG VSALNGR 1

CCTATGGGCAGTCGCGTGGCG PMGSRVA 1

TGGGGCGTCACGACTCGTCCG WGVTTRP 1

TGGTGTGCTGGTCGTGCTGCG WCAGRAA 1

GTCATCCGTATGAGGTCCTTG VIRMRSL 1

AATGAGACGTTTTTCGGTAGG NETFFGR 1

GCGGTGTGCAAGCGGCTTCGG AVCKRLR 2

TGGTTGGCGACGCGGGGCCGG WLATRGR 1

GTCCGGCACAGTTTCATTCAG VRHSFIQ 1

CTCCGGGGCGGCAGCCGGAGG LRGGSRR 1

GTGTTCACCATGGTTTGCCGC VFTMVCR 1

GGGCGGTTTAGCCGCTCTTGG GRFSRSW 1

GAGCCGGCGCTGCACGTTTGG EPALHVW 1

GGGTGTTGTGGCATTCCGTCG GCCGIPS 1

GGCCCTGGCGATATTCGTTCT GPGDIRS 1

GTGGCGGAGGGTTCCGGGAAC VAEGSGN 1

GATCGCGTCGGGATCCACACC DRVGIHT 1

CGGGAGGGCGGCAGGTACTCT REGGRYS 1

GGGCCGGAGGCGATTTGGAAT GPEAIWN 1

GAGGTTTTCTGCGACTTTTGT EVFCDFC 3

GACCGGCAGGTTGGCGCGGAG DRQVGAE 1

CTGGTCGTGAGGCTTAGTACG LVVRLST 1

GGGGCGACGGGTGCGAACCCT GATGANP 1

TCCGCTCTCCCTAGTTCGTGT SALPSSC 1

AGTTGTAGGTTCAGGCTCGGG SCRFRLG 1

TCCAGCGGGGTGGGCCCGACT SSGVGPT 1

GCCGTGTGGAGGGTCGCCATT AVWRVAI 1

GTCGAGCAGGGGACGGCCGGG VEQGTAG 1

TATTGGGTGTTGGCCGTTACT YWVLAVT 1

AGGGCGTGGAGTTTCGAGTTT RAWSFEF 1

GGTACCCGTCGCAGCGCCTAT GTRRSAY 1

GTTAGTCTTCCTTTGTCTTAC VSLPLSY 1

TGCGGGGAGTGGCGGTTGAGT CGEWRLS 1

AGTGACTGGCTGGATTCGGCT SDWLDSA 1

AGGCTCTGCGGGCCCTGCCAG RLCGPCQ 1

GTCTCCAGTTGCTCCACTGTT VSSCSTV 1

GGGATGGCGTCGGGCCTCAGG GMASGLR 1

GTTCGGGCGTGGGAGGAGCGG VRAWEER 1

TGCTGTGACATTGGGTGGGAC CCDIGWD 1

AACAATGGGTGGCGCTGTGCG NNGWRCA 1

TGCGAGGCGGTCGGTGCTCCT CEAVGAP 1

AGCTGGGTGGTGTATTGCGTT SWVVYCV 1

GTTCTGAGCGTCAGCACGCCT VLSVSTP 1

GTGTCCCCGCAGAATGTGTCT VSPQNVS 1

GTGGGTTGCTCGTGCGGGCGT VGCSCGR 1

AGCTGCAGCGCTGTCTGCTCC SCSAVCS 1

CGGGAGGAGGTCGGCTCGTAT REEVGSY 2

TTTGCGTCGGGCAGCCTTTCT FASGSLS 1

ATCCATCATAGGTTGGAGTGC IHHRLEC 1

GTGGCGAGGGCTAGCAGGTGG VARASRW 1

AGTGGGCTGTGCCACTGCCGC SGLCHCR 1

TTTGCTCTCTGGGCTTTGGGT FALWALG 1

TTTGACAGGAGTCTCGGGTCG FDRSLGS 1

GTCGCCCGTCTGCGCGGTGTT VARLRGV 1

TTGTTGTTGGTGGTGCGGGGT LLLVVRG 1

ATTCCCCAGGCTCTGTTCCTC IPQALFL 1

AGCCGGGGTCTGCCTATCGGG SRGLPIG 1

GGTCGGTTCCGCTGTTCGCCT GRFRCSP 1

GTTCCGCTCTGGTGGTTTTAT VPLWWFY 1

TGTCTTCTCGGCTGGTACGAT CLLGWYD 1

GTGAGTGAGACCGGGCCGGAT VSETGPD 1

TGTTCGCGCCATTGTTCGGCT CSRHCSA 1

AACATGCACCCGCTGTGCGTT NMHPLCV 1

ACGTTTCAGGTTCTTGTGGAT TFQVLVD 1

GGCGGTGCTCGGATTCCGGTG GGARIPV 1

CGGCGTGGGCAGTTGCTGTAC RRGQLLY 1

TGTCAGTGTGCGGAGAGCCGT CQCAESR 1

GCTTTCCGCACGAGCTGCAAT AFRTSCN 1

ACTAGTGGGGGCCGTGGCCGT TSGGRGR 1

AGTTTGCCCAGCGGCGGTCTT SLPSGGL 1

GGTGTTTGGCACCTGGGTCGT GVWHLGR 1

GACGGGTCTCAGCCCTGCACG DGSQPCT 1

CTGCAGATGGATAGCGAGCGG LQMDSER 1

GTGGCTGACTTCGTCAAGTTT VADFVKF 1

GCTGGCAGGGGCTGGTTGCAG AGRGWLQ 1

CTTGGGGATATGGTCAGGGCG LGDMVRA 1

TGGCTTGGCGGTTCGGTGAGT WLGGSVS 2

ATTCAGACCTGGCGCTTCTGG IQTWRFW 1

CAGTCCTTCACGATTCGCGGG QSFTIRG 2

CGCTGGTGTGGGGGTTGTCCT RWCGGCP 1

ACTCAGTGGCGGCGTTATGAT TQWRRYD 1

GGTCCGCGCGGGGCGCCCTAT GPRGAPY 1

ACGGATCGCTTGGTCGCGAAG TDRLVAK 1

TCCGTCGTCCTGGCCGAGACC SVVLAET 1

CTTTCGGAGTGGTCCTGGGTG LSEWSWV 1

CCCAGTCGTATTCCCATCTGC PSRIPIC 1

CACTTTGGGGCGGCCAATTCT HFGAANS 1

ATGGTCTACAGCTGTGGGTCC MVYSCGS 1

TATGTCGGCCGGAAGAGTTAT YVGRKSY 1

GTGCGGTTGTGGCATGACCTG VRLWHDL 1

CCTGCTCGGGGTGGCGGGTCG PARGGGS 1

GTTACTAAGGTGCTCATTCGT VTKVLIR 1

GGCTTTCGGAGGGAGTGGTTG GFRREWL 1

AGTCCCGTGTTGGACTATCAT SPVLDYH 1

AAGTCCACGGCGCAGTGCAGG KSTAQCR 1

TACGCTTTGTCGGGCGCGTGT YALSGAC 1

TCCCGGTGGGTCGGGAGGGGT SRWVGRG 1

GTGTTTCGCACTTTCGGGTAT VFRTFGY 1

TGTCGCGGGTTTGTCGATGTT CRGFVDV 1

AACTTCGAGTGGGGGCCTGTT NFEWGPV 1

TCTGGGACGTGCCAGGCTGTG SGTCQAV 1

TTTGACTACTTTTCTGGCGAT FDYFSGD 1

GGGACGGGGATCACGCGGCGG GTGITRR 1

CGGTGTCTCATTCGTCCGGAT RCLIRPD 1

GCTCTCTCGATCACTACTCCG ALSITTP 1

AATGTCGTGCTGCTCTGTGTT NVVLLCV 1

GGGCTGGTTCTCCCGGTCAAG GLVLPVK 1

GGGTACGCGGACTGCGTGCTC GYADCVL 2

GTTGGCCACGTTTCTCTCTCG VGHVSLS 1

GGTTGGGTTAGGCTGTCGCCT GWVRLSP 1

ACTAACTGTTACGGCGTTGGC TNCYGVG 1

TGTTCGGGCGTGGCCCTTCTT CSGVALL 1

GAGGGGCGCGACCACATGGCT EGRDHMA 1

TGCGAGCCTCAGTTCGATCAG CEPQFDQ 1

TGCCTGGGTTTTTCGGTTAAT CLGFSVN 1

ATGTTTGGCGCCTCGGATGGT MFGASDG 1

GCGCGTTACGTTGGGCATCCT ARYVGHP 1

CGGTGTCCGATCGGCATGCTG RCPIGML 1

CTGTTCTACGCGGGTTGGGAT LFYAGWD 1

TGGTCGCTCGGCTGGGACGTG WSLGWDV 2

GTTGGCGGCGCTAGGGGGGCG VGGARGA 1

GGTGCGGCGTGCAACACGTAT GAACNTY 1

CGGAGGTACTGCCTCTTGTTT RRYCLLF 1

CAGCTTCCCGCGCTGTTGAGT QLPALLS 2

TCTGGCCAGTTGCATATTGAC SGQLHID 1

TTCTTGGGCCACCGTTGTCGG FLGHRCR 1

TTGCCCATGCTCTCGTCCTTG LPMLSSL 1

CTCTGGCCCGGTGGCCTCAGT LWPGGLS 1

ATCCAGACCAGGAACAGCACC IQTRNST 1

CGTGGCCGGACTTGGTCTGAT RGRTWSD 1

GGCGATTTCGACGGTGAGATT GDFDGEI 1

GCGCTGAGGTGGTCGCTCCGG ALRWSLR 1

ATCACGGGTATGCACGTCGTC ITGMHVV 1

AGGTGGGAGTCGTTTGTTGGT RWESFVG 1

AATGGTCGTGCCGAGTATCCG NGRAEYP 1

CGGCGTCTCGGCATTATTAGC RRLGIIS 1

TGCTTTTGTTCTACGGTCGCT CFCSTVA 1

GGCTGGGAGGGCGCCGTTTGC GWEGAVC 2

TGTAATCTGGGCAGGCGCTGG CNLGRRW 1

GCTTCCCTTTTCTTGCTGGGG ASLFLLG 1

GCGCGGAGCCTGGTCTGTTAT ARSLVCY 1

TGTGGCCACGTCAGTGGGCCT CGHVSGP 1

TGTTGCCGCGGGTGGAGTAAT CCRGWSN 1

CTTAACTTCCGGCGGCATACG LNFRRHT 2

GGGTTGCTCGCTCTTGAGTCG GLLALES 1

CGGTGCCTCCGGCGGTGGCCG RCLRRWP 1

ATCTTCTCCGCCCGGGGGGAG IFSARGE 1

GCCATTCGGTCGAGTCGGCGG AIRSSRR 1

AGTAAGCCTAGGTCCGGCACG SKPRSGT 1

TATTCGCTCTCTCGCGACCTT YSLSRDL 1

GCGCTGCGGAAGGCCTGGGGG ALRKAWG 1

TGTCAGGGCTTTGGGCCTATT CQGFGPI 1

CTCCGGGGCACTAGGGCCCGC LRGTRAR 1

GTGTCGGAGGTGGTCGGCCCG VSEVVGP 1

ACGCTGAGCTCCAAGGGGAGT TLSSKGS 1

CTGGGTTTTCAGTGGCCGGGG LGFQWPG 1

TTCATGCATACGGTTAATTCG FMHTVNS 1

GGCGCTGCGTGCGCCACTGGT GAACATG 1

TACCGCGTTCTTCATCCGCTT YRVLHPL 1

TTGTGGGACGTGAACGGGAAT LWDVNGN 1

CTCGGTATGGGGCAGGCTTTG LGMGQAL 1

CGCGGTCCTACGATCAATTGT RGPTINC 1

TGGAGCGCGGGGCTGCGCCGG WSAGLRR 1

TGTCGTGAGCGGAAGGCGCCG CRERKAP 1

AGTATTTATCTTCCCCGGCGT SIYLPRR 1

CTTTGGAACAGGGGTCCTAAG LWNRGPK 1

GGGACTCGCGAGATCAGCTTG GTREISL 1

GGGTTGCCGATGTCCTCGGCG GLPMSSA 1

CACCTCTCTACCCGCGCGAAT HLSTRAN 2

TGGCTGATCGGTGTCCGCCGT WLIGVRR 1

GACCCCGTGGGGTGGATTATT DPVGWII 1

CATTTCAGCAGCGGCCTGGGC HFSSGLG 1

GGCGTGACCGCGGGCTGTCCG GVTAGCP 1

CTTCTGCATGTGAGGTATCTT LLHVRYL 1

TCCAACATCGTCGGGAGTTCG SNIVGSS 1

GCCGGTTCGAGTCCTCAGGGG AGSSPQG 2

CCGCTGTTCGGCCGGAGTCGG PLFGRSR 1

CGGTTGCTTGATCATTGGATG RLLDHWM 1

GGTATTCGGCACAGGCAGTCT GIRHRQS 1

CATGTGTACAGGTGGCGTCCG HVYRWRP 2

CAGTGGGTGAGGTCTGGCCTG QWVRSGL 1

GAGAAGTTGTGGTGGATTCGT EKLWWIR 1

GCGCCCGCCCCTCATATCATT APAPHII 1

TTCTATTTCTGGGTGTTCAAG FYFWVFK 1

CACGGGAGGAGTGAGAGTCGT HGRSESR 1

AGGCGGGACAACCGCGCCTCT RRDNRAS 1

TTTGCGGAGACGATGAGGGGT FAETMRG 1

GGGGAGCTCACGTTTGATCAG GELTFDQ 1

GCTGGCCTCGGCATCTGGTTT AGLGIWF 1

GGGGGCGCGCAGCAGGGTCAG GGAQQGQ 1

TACATGTCTAAGCCGCTCGAG YMSKPLE 1

TGCCCTCTGAGCTTTACTGCT CPLSFTA 1

GTTTGGCTTGCCCGGGGTACG VWLARGT 1

GTGATTTGCTCTAATAATATT VICSNNI 1

AGCGACACCAGTCGGCCTACG SDTSRPT 1

CCCACGAATGCTTTGATCGGT PTNALIG 1

TATCGTTGTTGTTTGGCGTGG YRCCLAW 1

TTTTGCTGCGGGTTTGGGACG FCCGFGT 1

CGTTTGCATGGGGGGCGCGGT RLHGGRG 1

CACTACCTGCTGCCGGCGCCG HYLLPAP 1

CATACCTACCGCTGTTCGTGT HTYRCSC 1

GGCGCGGCCTCTGTCCTTCTT GAASVLL 1

CGTGGTTGGACGCGGGCGGCT RGWTRAA 2

TGCCCGGCGGCGACGCATGAT CPAATHD 1

CCCGGGAACTTCACTGAGTCT PGNFTES 1

CCCTCGAGTCGTGCGACGGTT PSSRATV 1

GTTGACTCCGGTGCTATTTGT VDSGAIC 1

GGCCTTCGGGTTCGTGTGTCT GLRVRVS 1

AGGCGGAGTAAGATCAGTCCT RRSKISP 1

CCGTCTACGAGCAGCCTGTCG PSTSSLS 1

TGGCGCCTGAGGTTGGGCCAG WRLRLGQ 1

TTTCGGCTGAGTTCCATGGTG FRLSSMV 1

TGGACTGCTGTCTGGCGTCAT WTAVWRH 1

CCGAAGTTGTTGCGTCGCGGG PKLLRRG 1

TTCGTCTTTGAGCTGTATTGG FVFELYW 1

TGGATCTGCGGCTGGGGGTCG WICGWGS 1

CTGATTCACCATCGGTCCTCT LIHHRSS 1

GTCGGGAAGTTGCTGTCCCGT VGKLLSR 1

GCGGGGAACGGGTTTGCTTGG AGNGFAW 1

CAGAATCTTGGGTGGCTTCGC QNLGWLR 1

TCTTGCATCACCGCGAGGGTT SCITARV 1

TGGCGCTCTGGCACGAGGATT WRSGTRI 1

CTGGCGCATGGTTTCGTGAAT LAHGFVN 1

AGTCATGGTTCCTGGGCCGCG SHGSWAA 1

TTCACGTGTGGCTTCGGGAGG FTCGFGR 1

AGGGGCATTTGGGGCATGCGC RGIWGMR 1

GCGGATGCCAGTTGGGGCGGG ADASWGG 1

AAGCTTGGTACTTCGATCTAC KLGTSIY 1

CCCACGGGGCTGAACGGGACG PTGLNGT 1

CACTTCGGTCACACGGGTTCG HFGHTGS 1

GGTCGCTGCTGGTTTAACCCT GRCWFNP 1

AATGCTGCGGATTACATTCGC NAADYIR 1

GGTAAGAGGCTCGCTACGAGT GKRLATS 1

GACAGTTTCGAGTATATGGGG DSFEYMG 1

CCGTCGTGCGATCTGGCGGCG PSCDLAA 1

AACGGGGCTGGGGGTTATAGC NGAGGYS 1

GGGAGTGCTGTGGCCAGTGTG GSAVASV 1

AGCGTTGGGCAGCTCTGTTGG SVGQLCW 1

TTCCTCATTTTGCTTAGGAAG FLILLRK 1

CATGGGCAGCGGGGGCACGCT HGQRGHA 1

CCCGTGCCTCTGGCCGGGCTT PVPLAGL 1

TACTGTCGGGAGTACGGGTAT YCREYGY 1

GTGAGGTCCCCGGGTGTTCTC VRSPGVL 2

TCGGGTCGCTTTCGGGACCGG SGRFRDR 1

CGGCTTGGTTGCGGGCGGGAC RLGCGRD 1

GAGGGTCCGGGGGTGGCTCCT EGPGVAP 1

AACGTGAGTGTGTGCGAGTCG NVSVCES 1

TGGGTGGCCGTTCGCAGTTCT WVAVRSS 2

CTGAGGCCTGTGGTTGTGTCG LRPVVVS 1

CACGATTACAACGGTTCTGAG HDYNGSE 1

AGGTACTCGCTGGGTGGCCTG RYSLGGL 1

GTGGGGTATAGGCTGCCGCGG VGYRLPR 1

GGTCGCAGGCCCTCGAAGGTC GRRPSKV 1

GCCTTGAACTCCTGGAAGGAT ALNSWKD 1

GTGGGCAAGTTTAAGCACGCT VGKFKHA 1

ATCGGCGTCGCGCAGTTGATT IGVAQLI 2

AACACCGCGCCCCGCCTTGAT NTAPRLD 1

GCGTCTCTTTACAGCAGGTAT ASLYSRY 1

AGTCTCAGCGTGGCTAGCGGG SLSVASG 1

AGTCTGTCCGGTATTCGGGCG SLSGIRA 1

CGGCCGGCGACTTCGTTTCTT RPATSFL 1

ATTAGTGGCCACTCGGCGTGT ISGHSAC 1

CGGTGTCTTCTCTCGGGTGAG RCLLSGE 1

GTTTCCGTGGGGTCTGCGAGT VSVGSAS 1

GATCAGAGTTGCTTTGCTGGT DQSCFAG 1

GGGCCGGTTGAGGACGTGTTT GPVEDVF 2

TTCGCGAGTGCTGGGGTTCGC FASAGVR 1

GGGGGCATCATTTGTAAGTTT GGIICKF 2

CTGGTTTGGTGGTCGGTCACT LVWWSVT 1

TTTGGTCTCTCGCGTGGTGCT FGLSRGA 1

TATCTCTTTGGGGTCGGTTGG YLFGVGW 1

CGCATCGCGGGCACCGTGCCG RIAGTVP 2

GGCTGCTATCGTTGGGAGACG GCYRWET 1

GCCGTCGATGCGCGCCTCCCG AVDARLP 1

GCGATCGCGGCTCACCTCCGG AIAAHLR 1

GATGTGGAGTCGTTTCATGGT DVESFHG 1

CAGGGTATCTCGGCTGCTGCC QGISAAA 1

GCTGGTTCTGACTCTATTAGT AGSDSIS 6

AAGTATTTGGGGAGCCGCCGT KYLGSRR 1

GTCGATCTTGGCGGGATTCGT VDLGGIR 1

TTGAGTCCTAGCGAGTCTGGT LSPSESG 1

GTTTTGCGTGCGTCTAAGCTC VLRASKL 1

CCTAGCCGTAGTGCGCTTGGT PSRSALG 1

GAGTTGCTGAAGGCCTCGTAC ELLKASY 1

GTTGCCTTTATTTCCAGGTGG VAFISRW 1

GGCATCGGCCGTATCGGGCCT GIGRIGP 2

GTCGTGCTGCGCGACCTGTTT VVLRDLF 1

TTTCGGGTCGGCAAGTATATT FRVGKYI 1

CGGGGGATTAGGGAGAGGATT RGIRERI 1

TACATGTGTATTTCGGGGTCG YMCISGS 1

GAGTTTATCCAGTTTCTGGTT EFIQFLV 1

AGTATTGCGTTCAGTTCGTCT SIAFSSS 1

GAGGCTTGCGGGTTTCGTCAT EACGFRH 1

GTTGCGCCGCGTGGCTCGGGG VAPRGSG 1

GAGACCATCCCCTGTCTGGGG ETIPCLG 1

TTGGGTTGCCGCTGGGTGTGT LGCRWVC 1

AGGCTCCAGTCCCAGGCGAGT RLQSQAS 1

GTTTATTACGTCTGCTCGCCT VYYVCSP 1

GGTGCGCTGGAGGATCGTACC GALEDRT 1

CCTCGCGCCTCGGGTTTGGCG PRASGLA 1

GACGCTGCGGTCGGGCGCTAC DAAVGRY 1

GATCCTTCGGGTGTGCAGGTT DPSGVQV 1

CGCGGGGTTGACCCGCCGCTG RGVDPPL 2

CTCTCCCGCGTCTTCGCCGGG LSRVFAG 1

CTTCGTTGGCTCCCCGAGGCT LRWLPEA 1

GTGGAGGGCGTGCGCAACGCT VEGVRNA 1

CATTCCGTCGCTTCCTTGAAT HSVASLN 1

GATTTTCTCAGTAGCACGTGT DFLSSTC 1

CTTTCGCACCTGTCGGAGGGT LSHLSEG 1

CTGTTCTGGGTCTTGCTTGCG LFWVLLA 1

ATTCTGGTTATGAGGGTGCAG ILVMRVQ 1

CCTGTGGTCGAGGCTAATTCT PVVEANS 1

CGTGCTCAGTCTGTGGAGGTG RAQSVEV 1

GCTGGCGAGGGGTCGGAGCGG AGEGSER 1

CGCGAGTCCTGTGGTGGCGCG RESCGGA 1

AAGTCGGCGTGCCGCCTGTGC KSACRLC 1

CTGGAGCACCGCCGCGTGCTT LEHRRVL 1

GCGGGCCAGGTCCTGGTGGGT AGQVLVG 1

GGGCACCGGCATGGGACGGAG GHRHGTE 1

GCGCGCTGGTGCGTCGATCAT ARWCVDH 1

GATAGGCGGGCGCTGCAGGAG DRRALQE 1

CGCAGTGCCCCTGACGGTATT RSAPDGI 1

AGGAGTGCTGTTAGCCTTCCT RSAVSLP 1

TGCTTGGGTGAGGTGCATCCC CLGEVHP 1

GTGGTCTCGCTCCTTGTGGCG VVSLLVA 1

TTGTGGCTTGTCTTCCTCAGT LWLVFLS 1

ATCAGGCGCGGTCCCAGCTCT IRRGPSS 1

ATGGTTCTTCCCGGCGAGCCT MVLPGEP 1

GGTTTCGGGTCTCAGTGGCGT GFGSQWR 1

CATCAGTTCTTGAAGTTGCAG HQFLKLQ 1

TTTAGTTTTAGGAAGCATGGT FSFRKHG 1

TTGCCCCCGGTCGTGACTTTG LPPVVTL 1

CTTTGCTACAAGCCCTGGTCG LCYKPWS 1

TGGAAGGGTCGCTGGGCTCAG WKGRWAQ 1

AGTAAGGAGGGTGGTGGTTCT SKEGGGS 1

TTTGCCATGATTCTGGTGGCT FAMILVA 1

CTGAAGATGGCGGAGGTGGTG LKMAEVV 1

CGTAGGGGCGTGGGCTGGTCT RRGVGWS 1

ATTCGCTGTCCGTTCGCTACT IRCPFAT 1

TCGGGGCTTAACGTCGTCGCT SGLNVVA 1

GTTCGGCCCGACTGCGACGCT VRPDCDA 1

CGGGCCTCGCGTCTTGTCCGT RASRLVR 1

TTGTGTTGCAGGATTCGCATT LCCRIRI 1

TTGGGCGAGATTCATGGTTTG LGEIHGL 1

GGTGTTGGCGTGCAGCGGGCG GVGVQRA 2

TCTATGTGTAGGCGCGAGGCT SMCRREA 1

GTGCGGTGGATCGGGACTCTG VRWIGTL 1

CCGGCGGTGAGGTTGATTAGT PAVRLIS 1

GCGGAGCACGGGCATTATGCT AEHGHYA 1

CAGAGCTGGGGTTTGTGGCAT QSWGLWH 1

TTGGGTTGCGTTTGTCGTGAT LGCVCRD 1

TTGTTTCGCGTCACTGGGACT LFRVTGT 1

GGGCGCAGCGACACTGTGGAG GRSDTVE 1

GTGCTCCTGTTTGGCGAGGGG VLLFGEG 2

GTGGTGAGCCGCGAGAGCGCG VVSRESA 1

CGGCGTCGGATCGTGGAGGCT RRRIVEA 1

AAGATCATGGTGCGTAGCGAC KIMVRSD 1

CGCGTTTGGAGCGAGCTGCGG RVWSELR 1

GGCGTTGGCTCGGTTGGGAAG GVGSVGK 1

GTGGTTGTCAGCAACAACACT VVVSNNT 1

GCTCGGGTCGCGTTGGGGCAT ARVALGH 1

TACGATTGGTCGTACGATCGC YDWSYDR 1

AGGATGGTTATCCGCGGGTTT RMVIRGF 1

ATCCCGCTCGTCCTGATTACT IPLVLIT 1

TCTGTCTTGGGTGCTCTGGGG SVLGALG 1

AACTGCGTGGCGCGTGGGGAC NCVARGD 1

TCTTCGGGTGGGAGCGAGGGT SSGGSEG 1

GTCTGCTATGCCACGAAGATT VCYATKI 1

AGCGATTGTGGTGGGAGGTGT SDCGGRC 1

GTCGGGTACAGCGTGGAGGCG VGYSVEA 1

ACTTGTCTTACGTGTATGTTC TCLTCMF 1

GGGCTGGTGCGTCGGCTCATG GLVRRLM 1

GACTGGAGTATGGTGCTTGGT DWSMVLG 1

TCTCTGCTTCTCGCGGGTGTG SLLLAGV 1

TGGGAGGCGCGCCCTAGTTTT WEARPSF 2

TTCGAGGGTTACCTGGGCGGT FEGYLGG 1

ATGCTGCCGACGCTTCTGCGT MLPTLLR 1

GTGTGGCTCCGGGGCGGCACG VWLRGGT 1

CGTGGTTGCACGAACGAGTGG RGCTNEW 1

AGGTTCATGTATGCCGGTAAT RFMYAGN 1

TACTGCGCGTGTCCTGCGTCT YCACPAS 1

GTGAGTTTGGAGTCCTCTGGT VSLESSG 1

AGTGCTTGGAGGATCGCTGTT SAWRIAV 1

CTGAGCGTGGTTCCCTTTGAC LSVVPFD 1

TGCCGGGGGAGGCTGTGTAAC CRGRLCN 1

GTGTGCTGGGAGGGCTGTCTT VCWEGCL 1

GGCCAGTGTAGCCGCCTTGCG GQCSRLA 1

GGCATCTTGAGGTCGGAGCCG GILRSEP 1

TTTGTCGAGCGGGACAATGTG FVERDNV 1

CGGAGCTTTTCGGTTCTCCTT RSFSVLL 1

GGGCGGTTGATTGGGGGGTTT GRLIGGF 1

AACGTGGTTGTGCGGTGGGGC NVVVRWG 1

GGTGGGAAGGGGAAGGGTCCT GGKGKGP 1

TTTCTGAGGTGGTCGGCGGGG FLRWSAG 1

TCCCTCCGGAGGGTGGTGGGT SLRRVVG 1

GATCCGTTGAGGCGCGGCCAG DPLRRGQ 1

CTCGGCGCTGACAAGTGTCGT LGADKCR 1

GGCTGCTTGTGGGGCCGTTGT GCLWGRC 1

CACTTTGACAAGAGCTCGGGT HFDKSSG 1

CTGTTTGTTCCGGGCCCTGGG LFVPGPG 1

AAGGGGAGGGCGAGGTTCGCT KGRARFA 1

GTGATGGCCACTGGGCAGCGT VMATGQR 1

GTTTTGTCCAGGTGCGTTCCT VLSRCVP 1

GGTAGTGCGCGGGCCGTGCCG GSARAVP 1

ACGCGGAACAGCGTCAGTGGT TRNSVSG 2

TGTGACCGCGTCGCGTGGCCG CDRVAWP 1

GGCGCGGCGATTCGTATGGCG GAAIRMA 1

GGTGGGCGCAGTCGCATGTGG GGRSRMW 1

TGCCGTTTTAGGAGCCGGTTT CRFRSRF 1

GAGTATGAGTCTTTGATTAGT EYESLIS 1

CTGTGTCGGGGCTTCGCTCTT LCRGFAL 1

GGCGGTGGCATTAAGTGGCGT GGGIKWR 1

GTGGATCTTAGTGTGCGGGGT VDLSVRG 1

TGGTGTGGCGTCGGCGGCGTT WCGVGGV 1

CACTCTAGCAGGCGCTGGTCG HSSRRWS 1

ACTCATTCGTTTCCTTCTAGG THSFPSR 2

GAGTGCGTTACTTGGTGCGTG ECVTWCV 1

CTGCAGCTGGAGCGGAGGTCT LQLERRS 1

GATTTGGACGGTTGCGGTTCT DLDGCGS 1

CACGTGTCGCTGGCCTGTGTG HVSLACV 1

TATGACTTCGCCCCGGTGGGG YDFAPVG 1

CGCTTGACTACTGCTCGGGTT RLTTARV 1

GTCGCGCGCTTCGAGTATGGT VARFEYG 1

ATGCTCTGGCCTGCGTTCGCT MLWPAFA 1

CCGGGCCTCGGGCGCGCCAAT PGLGRAN 1

ATTGCTCTCGCGTTCGGGCGG IALAFGR 1

GTGTATCTTTTGTCTGAGAAT VYLLSEN 2

TGCGCCAACGCTAATGGGCCT CANANGP 1

GGCTGTGGGTTGAGTCCGCAC GCGLSPH 1

CGTATCTTCATTTGGGTCGCG RIFIWVA 1

GACTCTCCGTTCTGGTTGAGT DSPFWLS 1

GTTAGCGATGGGTGGTTGTCT VSDGWLS 1

AGTTGGCGCGGGCCCATGGGT SWRGPMG 1

CAGCGGTTGTACTCCGTGAGT QRLYSVS 1

GCGTTCTGCCGCGGGCAGAGG AFCRGQR 1

AACGTTCTTATGCGTGGTTGC NVLMRGC 1

CAGCGGCGGGTCTCCCTGGTG QRRVSLV 1

GGGGTCGCCCCGACGGTGACT GVAPTVT 1

CCGAGCATCGTGGGGCTTCTC PSIVGLL 1

TTGGGGATGCCTTGCCCGTTT LGMPCPF 1

TTCTTGTGGCTGTGCTCTTCT FLWLCSS 1

TGGCAGCGCATTAGCGTGGTT WQRISVV 1

CATGTTCCCGGTGTGGGGGCT HVPGVGA 1

AATCGCGGTATGGGGATTGCG NRGMGIA 1

GCCATTGATTGCTGCCATGAT AIDCCHD 1

GAGCCCGATGCTCGGTCGCAG EPDARSQ 1

CGGCACCTGTTGGGGCTCCCT RHLLGLP 2

TACGTTTATAACGGCGTGGGT YVYNGVG 1

TTGAGTGGCAAGGGGCTGGTT LSGKGLV 1

GGCGCTGGCTCTCTGCGCTGG GAGSLRW 3

CTGCGCAGCGGCTTTGGTCCT LRSGFGP 1

CCGTGCCGCTGCACCGCTATT PCRCTAI 1

GCTGCCAGGATCGGCGGGCGT AARIGGR 1

GATATCTCGAAGCTGAGTAGT DISKLSS 1

TTTTCCACCACGACGGTCCCT FSTTTVP 2

GGGGCCGAGAGGGTCGTCGGG GAERVVG 1

GGTGTCGCGGTTTGGCACATG GVAVWHM 1

TTGCACTCTACGCATTGTTGG LHSTHCW 3

GGTACTCGGACCCTTGTTGTT GTRTLVV 1

TGCTGTTCGGCGCGGTTTCCT CCSARFP 1

CCCGTCGCGGGCTACGATCAT PVAGYDH 1

TACAAGGTCGACCCTACGCCT YKVDPTP 1

CGGGCTTTCTGGGGGGTGAGG RAFWGVR 1

CTGAGGGCCGCCCATAGCTTT LRAAHSF 1

GGGTGTGGCTCGGGTTTGTGT GCGSGLC 1

TCGGTCTGTACTAGCGTTAGC SVCTSVS 1

GCTCGCCGGATGGCCGCTTGG ARRMAAW 1

AAGTCGTGTGTTTGCCTCCAG KSCVCLQ 1

TATGGCTCCCTTCTCCGGCTC YGSLLRL 2

CCCTGGTGCAGCACGTTTATG PWCSTFM 1

GCGCTGTTTAGCACCTGCTCG ALFSTCS 1

AGGCGGGATTTGGGTCATCTT RRDLGHL 1

TACAAGGGCGGGTACGTGGGT YKGGYVG 1

TGTGTGGTGCGTTGGTTCCGT CVVRWFR 1

ACGCCGCTCCTGATCCTTTCT TPLLILS 1

AAGCCCCGCAGCTCTGGTGGT KPRSSGG 1

CTGATTACGTTGTGGTATGAC LITLWYD 1

AATAAGGTGGTTTGTCATCGG NKVVCHR 1

CAGGTGATCCTGAGCAAGGTT QVILSKV 1

GGGCGGGGCGAGGTCCGTTTG GRGEVRL 1

GTCTGGCTGCTGTTCGTTCTG VWLLFVL 1

GTTCCGGGTGTTAACAACCAT VPGVNNH 1

GTTATCCGGAGCTCTCCGCGT VIRSSPR 1

CGTACTATGTTGGGGTGTGTT RTMLGCV 1

GGTGTGGCTGGGGACATGAAT GVAGDMN 1

CTGTGGAAGCCTCGTGCCTAT LWKPRAY 1

CTCGAGCCTTGGTTCGCGTGG LEPWFAW 1

CTGGCGAGGATCCACATTCCT LARIHIP 1

TTTCGTTTCCCTTGTGCGGCT FRFPCAA 1

TCTAGTGCGTGTTCGGCGTGG SSACSAW 1

TGTAACTGGAAGGTTGGGTGG CNWKVGW 1

ATCCAGCGCTTCGGTGGTTAT IQRFGGY 1

CATAAGGAGTGCAGGTGCCCT HKECRCP 1

GTTCCCGCGGCGAGCTGTGGT VPAASCG 1

TCTTCGGGGTGGGGGGCGTCT SSGWGAS 1

ACGATTCCGATCCTTCTGCCG TIPILLP 1

GGTGTGGCGATTCGCCCGCTT GVAIRPL 2

GGCTTTCGTCCGACGTGGGCG GFRPTWA 1

AGGGTCCTTACCATCAGCGCG RVLTISA 1

GATGTCGGGATCAACCTTACT DVGINLT 2

GGGCGGCGTGCGCCTCGTCCC GRRAPRP 1

CGCTCTTCCTTCCTTTGCGAC RSSFLCD 1

CACTGGGACGCGAAGCGGCCG HWDAKRP 1

GTGGGCTTCTCTTGCGCGGCT VGFSCAA 1

CCGCGGATTCGTGGTGTCGTG PRIRGVV 1

GGGAAGTTCAACAGGAGGTCT GKFNRRS 1

GCCAGCGGGGGCGAGGGGGGG ASGGEGG 1

GCGGTGGCGAGCAGTGGCTGT AVASSGC 1

CGCGGTATCTTGCAGACTCCT RGILQTP 1

CAGCCCGACTGCCGTACTCGG QPDCRTR 1

TCGTTTGCCGTTTCGGGGCTG SFAVSGL 1

CGCGCGGCGAGTCCCTTGGGG RAASPLG 1

ACCCGTGCGTGTGGCCTCTCT TRACGLS 1

TTGTGCGTCGGTGAGGCTCCG LCVGEAP 1

AGTCTGTTCATCCGCGACTGC SLFIRDC 2

GGGGTGAGCTTGATCTGCCCT GVSLICP 1

GCTATTGTCTTCACCGTCGTG AIVFTVV 1

CACGTGGAGGATGGCGAGCGG HVEDGER 1

AGGAGGTCCCCGTATTACGGG RRSPYYG 1

CAGAGGTTGCTTTGGGTTGTT QRLLWVV 1

CGGACGCTGCGTCCGCTGGGG RTLRPLG 1

AGTTACCTGGGTTCTGTCGGC SYLGSVG 1

CTTTGGCTTAGCGCCGTGTCG LWLSAVS 2

GCGGGCTCGTGGGTCAAGAAT AGSWVKN 1

TGGGCGCGTCTGAGTGGTCGT WARLSGR 1

AACCTCTTGTCCGCTAAGTGG NLLSAKW 1

CCGTATCCGCGCCTGTATTGC PYPRLYC 1

AGTATGCGGCTGGTCTCCGGC SMRLVSG 1

CTCTCCCACGATGCCTGGCCG LSHDAWP 2

CGTGCCTTTCACTGGGGTAAT RAFHWGN 1

GCGTCTCAGAGCCGTGCCGGT ASQSRAG 1

TCCCCTAGGCTGCGTCTTTGG SPRLRLW 1

TGTGTGGTCGTCTGTATTTCT CVVVCIS 1

CTGCCTCCGCCGGCCCGGAGG LPPPARR 1

CGTCTCCGGGGGTGGACGCCG RLRGWTP 1

GGGCATCCCAGGTCTCATGGG GHPRSHG 1

CGGGGGTGCGGTGACCGGTTG RGCGDRL 2

GGGCTTTTCATCGCCACCGGT GLFIATG 1

CGCCATGCGACCGGCTTTCCT RHATGFP 1

TGCGAGCGCGGTGAGGGGGTT CERGEGV 1

TCGGTTCTGCGCCTGAAGCGT SVLRLKR 2

ATGTCGCTCAGGTGGGTCCAG MSLRWVQ 1

CACATTACGTTGGCTAACCTG HITLANL 1

AATGGGCTGGTTCGCTCGGCG NGLVRSA 1

TCTTGGGGGACGGGTTTGTGT SWGTGLC 1

TACGGGCAGGATGGTACTCTT YGQDGTL 1

GTCCTGGTCTTCGCCGGGCCG VLVFAGP 1

GGGCGCGGCTTGGGCGGCCAT GRGLGGH 1

ATGCTGCCTCCTCATGAGGAT MLPPHED 1

GGGAAGGGCTTGTGGAGTGTG GKGLWSV 1

ATGACTGCGGTCGTCTGTTGT MTAVVCC 4

GTGCCTCTCAGTCGCTGTCGT VPLSRCR 1

GCGTACCGGGGGCTGTATTCT AYRGLYS 1

AGGAAGGAGAGGTGGGGGACG RKERWGT 1

TTGAATAGTCCGCTGCCCATT LNSPLPI 1

TCGGGCTTTAACGGGCGTTTT SGFNGRF 1

GACTACGGGACCGGTACGGAT DYGTGTD 1

AGTGACCACTGCCAGGCGAAC SDHCQAN 1

GGGTTTGCCGTTATTCTCGAG GFAVILE 2

TCGGAGGCGGGCTGTGGGGAG SEAGCGE 1

CGGGATTGGTTCAGGCTTACT RDWFRLT 1

CAGTGTTTCACCGGCCCGTTG QCFTGPL 1

GCTCGCATGGCCTGTCTGGTT ARMACLV 1

TTGCTCGCGCCCGCGGCTCGG LLAPAAR 1

GTTTGGACGGCCTTGACGTTT VWTALTF 1

GACTGGATGATGCGCAATCTT DWMMRNL 1

GGGGAGTGCGGGAGTACTCCT GECGSTP 1

TTTGGCCCTAAGAGGCGTGCG FGPKRRA 1

CGCGGGGATCTTAGGGGTCAC RGDLRGH 1

TTCTGGGGCGATCCCATGTGT FWGDPMC 2

TTCTACATCCTCCGGGGGATC FYILRGI 1

GATGCTGGTGCTGGCAGGTAT DAGAGRY 1

TCGCTGTGCAGGTGGCCTCGT SLCRWPR 1

TATCTGCACAGGCCCGAGCTT YLHRPEL 1

TTTCGTCGCCATGGGTGTTTC FRRHGCF 1

CGTGTGTGCACTGGGATTAAC RVCTGIN 1

CGTAGTGGTGCTCAGTCGTGT RSGAQSC 1

TGGCCGCGCTGCACTAGCCTT WPRCTSL 1

TCTGAGCTGGCTGGCTCCATC SELAGSI 1

GGGTCCCTGCGGCTGGATCCT GSLRLDP 1

AATCGCGAGTGTGGCTGGGAT NRECGWD 1

TACGGGGTGCAGAGGTGGTCG YGVQRWS 2

ATTCGCCTGCTCAGTTGCTTG IRLLSCL 1

CGTGTCGACTGCTGGGCCTAT RVDCWAY 1

CAGGTTTATTACTTGTTGTAT QVYYLLY 1

GGCTCGACGGTTAAGGTCATT GSTVKVI 1

GGTGTTGGGCCGCTGCTTGCG GVGPLLA 2

GTGCTTCTCAGCCACAGGAAG VLLSHRK 1

CGTGTGAGGACCCGGTTTGCG RVRTRFA 1

TACCAGTGCCGCCGTTGTGGT YQCRRCG 1

ATGATTGACAGCACGGTGTGT MIDSTVC 1

GGGCTGGCCGGGGTGTTGGTT GLAGVLV 1

AAGGCGCTCGTTTATCTCGTG KALVYLV 1

GTGTTGGGGCAGGAGGCTACT VLGQEAT 1

GTCATTATCGCTGCGCGGCGG VIIAARR 1

GTGTGGCCGTATACTGGTCTG VWPYTGL 1

GGGGTGACGAACCCGGTTTGC GVTNPVC 1

TTGCGCTTGCCTCGTTCTCTT LRLPRSL 2

AGCTGCGTGGTCAGCGCGAGT SCVVSAS 1

AATTATCTGTTCCGGTATGGT NYLFRYG 1

CCGTGTATGAATCCCCGCGCT PCMNPRA 1

TGGTGGAGCCCCAAGCCCTCT WWSPKPS 1

CGTAGCGGCCCGACCAACGGG RSGPTNG 1

CACAACCGGGTCGACGGGGGC HNRVDGG 1

AACCGGCCGTACCGGTGGCGT NRPYRWR 1

TTGCGCCAGAGCGCGGCGGCG LRQSAAA 1

TCGCTGCGGCGGGGCGTGCTT SLRRGVL 1

TGTGGCTTCGCTATCGACCTG CGFAIDL 1

TGTATCCGCTGGGACCCGCCG CIRWDPP 1

CCGAGGATCGCCGCCGTTCGT PRIAAVR 1

TGCGGGCTCTCCAGTAAGGCG CGLSSKA 1

GCCCGGCAGTGCGTCACGCTT ARQCVTL 1

ATCGGTTACTATGGCGGCCTT IGYYGGL 1

GCGCAGGATATGCCTTGGGTG AQDMPWV 1

CTGGAGGTCCCTGGGTCGCGT LEVPGSR 1

TGTGACGATTGCTGCAGCCCG CDDCCSP 1

CGGACTTACACCAACGTTATG RTYTNVM 1

TGCGTCCGGAGCGATTTTGTT CVRSDFV 1

CGGCAGTTTTGCTGCTTTACG RQFCCFT 1

TGCCTTGCGTGGCCGCTTCAT CLAWPLH 1

CGTTTTGCTCCGGGCTCTATG RFAPGSM 1

CTCGCTTTTGGGACGTTGCGG LAFGTLR 1

AAGCAGGGCAGTGAGGTGCTT KQGSEVL 1

ACGTATTTGTCGACGCGGGGT TYLSTRG 1

ATGGGCAAGGGCCGCAGGTTG MGKGRRL 1

GTTGGGATGGCCGGCAGCTGG VGMAGSW 1

TGTTGTCACGTTAGGTATAGC CCHVRYS 1

CCGGTGTTGCCGATTTCCCGT PVLPISR 1

GGTGGCTGTTGCACGAGGTAT GGCCTRY 2

ATGACCGCCGCCGCGTATGTT MTAAAYV 1

TCGTCTGGGAGCCGGGACCAT SSGSRDH 1

TGGGCCGTTAGCTGCTTTTGT WAVSCFC 1

GCTGTTGGTGCTCTCGCGTCT AVGALAS 1

GTGCTGGCGGCCCAGGTCTTG VLAAQVL 1

CCTGCTTCTCGGGTGCTTCGT PASRVLR 1

GGTACTCAGCTGGCCTGGGAG GTQLAWE 1

GGGTGCCGCTGGCTGAGGAGG GCRWLRR 1

AAGATGGTGCCTGGCCTCCGT KMVPGLR 1

TGGTGGTATTCGGGCATGTGC WWYSGMC 1

GTTTTGTGCAATTTCGGGTGT VLCNFGC 1

CGCATTTTTAGTGAGGTGAGG RIFSEVR 1

GGCGGGCTCACGGGCGTCCGT GGLTGVR 1

CACATTTCTGAGGAGCTTTGG HISEELW 1

GTGGTCGTCAAGGGTGCTAAT VVVKGAN 1

GCTTTTGATAGTGCTTCGCCT AFDSASP 1

AGGAGTTTCTTGGCCGATCTT RSFLADL 1

AGTCGCCTTACTGACGTCAGT SRLTDVS 1

CGGCGTGTGAATCGTGATCAG RRVNRDQ 1

GGCTCGCAGACGGAGCGCAAT GSQTERN 2

GGTCCTGTGGTTGAGTGGGTC GPVVEWV 1

TACGGCTGCTCTGATCTTTCT YGCSDLS 2

GCTGGGGTCGCGTTGGATGCG AGVALDA 1

TTTACTAGGTGGTGGCGGTGT FTRWWRC 1

ATGCGGATCGCGGACTTTCGT MRIADFR 1

GATCCGCCGTGGCATGGCCGT DPPWHGR 1

TGGCGTGAGGGGCACGGCCCG WREGHGP 1

GGTGCCGTGACGGTGGCCTTT GAVTVAF 2

GCGGTGGTTCGTAGTCTTCGT AVVRSLR 1

GTTGCGGTCCGGTTCCCGGCG VAVRFPA 1

TACCGGCTTCGTTACTCTTAT YRLRYSY 1

GTCGCGGTGAGCGTCCATTCT VAVSVHS 1

TGTTACGGGGCCTCTTGTGGG CYGASCG 1

CGTCAGTATACGGGTAAGCCT RQYTGKP 1

AGTGGCCTGGGGCTCTGGGCG SGLGLWA 1

CCGCTTTCCACGGTTACTACG PLSTVTT 1

TTCGGGGGCAGTGTCCTGCAG FGGSVLQ 1

TTGCTGCGTGAGGGCCCGGGT LLREGPG 2

TTCCTTACTATTAGGCAGGGC FLTIRQG 1

TTGTGCGACAGTGTGGCTAGT LCDSVAS 1

GTGCAGAGGGCCGGGTACGAT VQRAGYD 1

CAGCTGGAGCGCGTTTATGGT QLERVYG 1

TGCGAGCTCTACACCTATGTG CELYTYV 3

AGGGCCAGGTGTGTGATTGAC RARCVID 1

TGCGGGGGTTTGCCTGTCAAC CGGLPVN 1

TGTACGCTTTTTTACACTAGG CTLFYTR 1

TTCGGGCTGGGGTGCTCCTTG FGLGCSL 1

GGTAGTATCAGTCGCGACGGG GSISRDG 1

TGTTCGGAGGCCCTGAGTCCT CSEALSP 1

GAGACGGTGCCTAACGAGTGT ETVPNEC 1

TTTCGCGTCAGTGGTTTCAAG FRVSGFK 1

ATTAAGGATAACGAGCTGAGT IKDNELS 1

ACGGGCTTCGCGCGGGGGTCT TGFARGS 1

GAGGCTGAGCCGTTGTTCGAG EAEPLFE 1

GGGGGTCCGAGGTCCCCTTCT GGPRSPS 1

AAGTGGAGGGTCGCTACTGCT KWRVATA 1

ATTTCTAATCGGGCCGCGGTT ISNRAAV 1

GGGAGTTACGCGGCGGTGCGT GSYAAVR 1

CTGAGGGCCGTGGGTACTCTG LRAVGTL 2

CTTTGGACTCGCTGTGCGTGG LWTRCAW 1

TGCATGCCGGCGGCGTGGGCG CMPAAWA 1

TGCCGTCCGTGGGGCTCTTCC CRPWGSS 1

GCCACCTCTAATCTCACCGCT ATSNLTA 1

AGTTGGCCTAGGGTTGTTACG SWPRVVT 1

ACGAAGCAGCGGTACGCCGTT TKQRYAV 1

TACCTGAACGTGAGTAATATG YLNVSNM 1

CGGCATATCTCCTGGTATTCG RHISWYS 1

GTTTTGCGCTTCACTGCTTGT VLRFTAC 1

AGCTCGCTTTCGGAGCGGGTT SSLSERV 1

GGGGAGCGTCCGCCGCCGAGG GERPPPR 1

CTGTGGGTTAGCGTCTGTCAG LWVSVCQ 1

AAGGGGCGCTCCGGGGTCTTG KGRSGVL 1

TTCTGTCGCTCGGGGGATCGC FCRSGDR 1

TGTCGGTTTACGAGTGTGACT CRFTSVT 2

GGGGTCTGCATGCGGCCTCGT GVCMRPR 1

GGGAAGTCGCCTGCCATCAGG GKSPAIR 1

GTGTTGGCCATGGTCCGGGGT VLAMVRG 1

ACTCACTTCAGCGAGATGCGT THFSEMR 2

GTTCGTATGCGCTTTTTGACG VRMRFLT 1

GTGTCTGGCTTTTTGATTCTT VSGFLIL 1

ACTGGGATCAGGTCCTGCCAT TGIRSCH 1

GCCTGTTCCGTCCACGGGCAG ACSVHGQ 2

AGGGCGGTGGACGACAGCGAG RAVDDSE 1

CATCGCGGGGACGTGTGGGGT HRGDVWG 1

GGGATTTGGGCGCGGACGGGT GIWARTG 1

GCTCCTGCTGCGGTGCTGCGC APAAVLR 1

GCTCGGGCTTTGGTGTTGCGG ARALVLR 1

ATGAAGATTCAGAGGCTGTAT MKIQRLY 2

GTGAACGTCTGTGTTACGTGG VNVCVTW 1

CGGGAGAAGTGCTCTTGCGGG REKCSCG 1

CGCCAGGTTACGGCCCCTACT RQVTAPT 1

CATTTTTCCAGGGCGTTGGGG HFSRALG 1

GCTAGCTGGTGTGGGGTGCCT ASWCGVP 1

CCTGTGGTTTTTGCTTCGCGT PVVFASR 1

TTTTCTGTTGATGGTGAGTCT FSVDGES 1

CGGGACGCGCGGGTGTCGCAT RDARVSH 1

TCCAGGTGGAATCGTGGCTTG SRWNRGL 1

GTTAGGCTCATGGTCTTGCGT VRLMVLR 1

AGTGGGCCGAGGCATGCTCTG SGPRHAL 1

GGTGCGTTTTTCTTTTGGGAG GAFFFWE 1

CTTTCGGGCCTTCCCGTCCGT LSGLPVR 1

ACTCTTGAGTTTATCCTGCGC TLEFILR 1

TGTGGTGCCGGGGTGTCGATG CGAGVSM 1

GAGTGTACGTTCTCCGCGTAT ECTFSAY 1

AGGGTTCGCGGGAGCGGTCGT RVRGSGR 1

GGGGTTAGGATGTTCGGCAAG GVRMFGK 2

CTCTTCCCGAGCGCGGCGCCT LFPSAAP 1

ACTCTGTTGAGCGCCAGGCGC TLLSARR 1

CCGTGGGCTTATCGGGCCGCT PWAYRAA 1

CTTATCGTCGATGCCGTGCCT LIVDAVP 1

CCGAGCAGCATGCCTGTTAGG PSSMPVR 1

GTTGCCGGTACCGGTCGGAGG VAGTGRR 1

GGTCGTATCAGGTCCCCCTCT GRIRSPS 2

CGCTTGCGTCTGACGGCGTGG RLRLTAW 1

GTTGCTGAGTACATCTGGGTT VAEYIWV 1

GTCGACGTCAGGCCGTACATG VDVRPYM 1

GCGCATATTGTTATGGTCTTT AHIVMVF 1

AAGCCTGTTGGCACGGATGCG KPVGTDA 1

TGCTTCAGCAGGAGGTATAGT CFSRRYS 1

AAGTGTCATACTTCGATGCGG KCHTSMR 1

CGTCATGCTCTGGATCGTCCT RHALDRP 1

TGTCGCAGCCACGCTAGTGGT CRSHASG 1

GGCGACCGTACTTCGACTCTG GDRTSTL 1

CACGTCGACCTGTGGGAGCCG HVDLWEP 1

CACTGGCTCTTTGTTTGGAAC HWLFVWN 1

AGTGTGCTCGTGTTGGAGCCG SVLVLEP 1

TTTTCGCGTTTCAGGGTCTTG FSRFRVL 1

ACGGTTGACAACGCGGGGGTT TVDNAGV 1

CACCTGTTGCGGTCTGGGAGT HLLRSGS 2

AGGTTCGCCAGTGGTCTCTAT RFASGLY 1

GCCAGGCGCAGTGCCTATACG ARRSAYT 1

AGGAGGTTCTGGTTCTTTAGG RRFWFFR 1

ATGTCCTCGAGTGATGACCGG MSSSDDR 1

CATCGCCTCAGCCCCAGTATC HRLSPSI 2

GGCTACAACTATAGCGGTTGG GYNYSGW 1

GTCGTCGAGGGCTTTTCGCTT VVEGFSL 2

TTCCTGCGCGTTTGCGAGGAT FLRVCED 1

GCGCACGTGGGGCACGAGTCT AHVGHES 1

GCCTTTCGCAGGGAGGCTGGT AFRREAG 1

GTCGTCCATCGTGGGCCTCCT VVHRGPP 1

GGTCTTGCCTGGGTCGGCAGG GLAWVGR 1

GATGTGCATGGCGATGGGCTG DVHGDGL 1

GGCGTTTCGTTGGGTGTTGAG GVSLGVE 1

TTTGGCGATTGCATCTCGCAG FGDCISQ 1

GTCGGCAGCGCTTCTTGCGAT VGSASCD 1

GGGTGGCGCAGGTGCTCCTGC GWRRCSC 1

GTGCTTGGTCAGGATTGGCAG VLGQDWQ 1

CTTCATGCGTGTGCTGTTGGG LHACAVG 1

GGCATCATGATCTTCTATATT GIMIFYI 1

TGGGAGAGCCCGTACGGCAGG WESPYGR 1

GCTTGTTGCCCGTTCTCTAGC ACCPFSS 1

TTTGGCGGGAAGCTCGAGGTG FGGKLEV 1

CGCGCGTTGCAGTTGTGCCGG RALQLCR 1

GATGCGCGCGTGCTGGTGCTG DARVLVL 1

TATCTGGTTTTCTTCTCTTAT YLVFFSY 1

ACCCCTAGCCGGCTGGAGCGC TPSRLER 2

AATGGGACTCTCCTTGACTAT NGTLLDY 1

TGGATTCAGCGCGCGACTGCG WIQRATA 1

TATTTTGCGTGGACTAGTCTG YFAWTSL 1

CCTTGGTGGCTCTTGGCTCGT PWWLLAR 1

TGGAGCTGGTGCTTTAAGTCG WSWCFKS 1

CGCTACTCTTTTGCCCCGCCG RYSFAPP 1

ATTGGTCCGCGGCGCAGTTTC IGPRRSF 1

CCGAGTTGCGGGTATCTGGGT PSCGYLG 3

TTGGGGCTTACTGTCAGGAAC LGLTVRN 1

AGGCAGCGCCTGTGTTTGGAT RQRLCLD 1

GTCGACTCGCTGTGCGTGGGG VDSLCVG 1

GTCCGCTGCGGCTTGTGGGTC VRCGLWV 1

CTGGTGATCAGGTTTGAGGCT LVIRFEA 1

CGCCATATTCGGTATGCTGGG RHIRYAG 1

GGCGGCCCCGTCCTTCTGAGC GGPVLLS 1

CAGTTTTGGAGGACGCCGGGT QFWRTPG 1

GACGTCGGTTACGACGAGCGG DVGYDER 1

CGCCTCTCGTTTTCCTTTCAC RLSFSFH 1

TCTCGCGCTAGTCGTCTGAGT SRASRLS 1

TTGTATCTCGCGGTCGTCTAC LYLAVVY 1

GTTCGGATTCGGAGTGCGCAG VRIRSAQ 1

TCGCGGTCTTTCGCCGGCTCT SRSFAGS 1

GTTCGGCTTATGTTCCAGTCG VRLMFQS 1

CTTTCCGTCCCGCGCTCGAGT LSVPRSS 1

TTCGCGGTGGTTGTGGGTCGT FAVVVGR 1

GAGTTGGTGCGGGCTCGGATT ELVRARI 1

TGTACGAGGATGTTGGGCACG CTRMLGT 1

CAGTGTGTGTCCTTGGCCTGC QCVSLAC 1

TGGTGCTGTTCGAGCAGTTAT WCCSSSY 1

CATTTGGTGCACGGCTCGGCG HLVHGSA 1

TGGCATGTGCCTGTGTCGTGG WHVPVSW 1

AGCGTGCTCTTGCGGGGTGCT SVLLRGA 1

TGTCTGTGCTTGGCCAACTCT CLCLANS 1

GGGTTGTGCGTGAGTAACACT GLCVSNT 1

ATGTTGGTTCATGCCTTTTGG MLVHAFW 1

AGCATCTTTGTGGGGCGGCCT SIFVGRP 1

CGGGTGGCTGTCGTTGGGCCT RVAVVGP 1

TATTGGCGGGGCGATTGCACG YWRGDCT 1

GGCCGGTGTGTCCTGCACTCT GRCVLHS 1

CGGTGGCGCAGTTCGTTTTAT RWRSSFY 1

GCCGTCACTTTGTCTTGTGTC AVTLSCV 1

GGTGACTATCCTGTTTTGTCG GDYPVLS 3

GGCAAGTATTGGGACTGGTGC GKYWDWC 1

GGCCGGGTGCCTTATTCGAAT GRVPYSN 1

GTTGTCCGTCTCAGCATTCCT VVRLSIP 1

CAGACTAACGAGTCTAGGACT QTNESRT 1

CGTGACTACAGCGCCTGTTCG RDYSACS 1

GAGTGCAGGCTCCTGGGGAGT ECRLLGS 1

TTGCTGGTTAGGTACAGGCTT LLVRYRL 1

AGCAAGTGGTGGAGGGTGCTT SKWWRVL 2

GCGGCCTGGATTTGCGGCCAC AAWICGH 1

GTCAACGGTTCGGCGGACCCG VNGSADP 1

CAGGTTCTCGGGTTGAGGGCT QVLGLRA 1

ATGGGGCTGTCGTGGACGAGT MGLSWTS 1

ACCGGTCACATCGTCGTTCTT TGHIVVL 2

CTCCGTTGCTGTGGGTGGTTT LRCCGWF 1

TCTCGGCCTATCGTCAGTCGG SRPIVSR 1

CCCCCGCGTCCGCGGCTCCAT PPRPRLH 1

TTGGACTGCAGCTCTAAGCGG LDCSSKR 1

TTCCGCTCTTTTAGTGACGCT FRSFSDA 1

TCCCGTGGTCCCAGCTCCGGG SRGPSSG 1

TGTTTGTGCCGTACCGACGTT CLCRTDV 2

GGTATGGCTAGTGTTCCGGGC GMASVPG 1

ATGCGGTTCTTGGTTAATCGT MRFLVNR 1

CATGAGGTGGCCGCTTCCAGT HEVAASS 1

TATCTGATTGCCGGCGCTCCG YLIAGAP 1

CGCACCAAGCGGTCCGTTGCT RTKRSVA 1

GTTAAGGGTAGCAAGACCTTG VKGSKTL 1

TGCTACGTCACCCCTCTGGGT CYVTPLG 1

AGGCGTAGCGTGGGCGTGCGC RRSVGVR 1

GCGCGCGCCCGGAGGTGCCTG ARARRCL 1

CGCGTGGGTGAGGTGCCTCAT RVGEVPH 1

TTTCGTGTGTTTAGGGCGCGT FRVFRAR 1

GTTACTTTCCACCGGATGTGT VTFHRMC 2

GAGAGCGTCACTGCTGAGCTG ESVTAEL 1

CTGTGTGTCTGGTCGGGCCAT LCVWSGH 1

TCGAGCCCGAGTTTCTTTCGT SSPSFFR 2

TCCTCTCGGGCTACGGATCCT SSRATDP 1

GGGCAGAGTTCCTTGTGCTCG GQSSLCS 1

CAGGGGAGGGCCGCTGAGCTT QGRAAEL 1

GATTTCGGCACTCAGGACGTT DFGTQDV 1

CGGGGTCGGGCCGGTGTGTCT RGRAGVS 1

CAGGTCTTCGCGGTTGCCCGG QVFAVAR 1

ATTAGCGCGTGTAGCGCGCCT ISACSAP 1

CTTGTGCAGTCCGGCATGTGT LVQSGMC 1

GGTAACCACTGTGGGACTTGC GNHCGTC 1

GTGGAGGTGCTTTTGATCACT VEVLLIT 1

GCCCGCTTCCCTCGTGGTAAC ARFPRGN 1

CATGGTAGGACGTGTGTTGGG HGRTCVG 1

GCGTGTCTTAGGTGCCAGACG ACLRCQT 1

ATGCAGGTCATTCGGACGTCT MQVIRTS 1

TGCCGGGTGGAGGGCCGCATG CRVEGRM 1

GGGGTGCGGCCCTGGAGGGGG GVRPWRG 1

AGCCAGGGGGAGGAGTTGCTT SQGEELL 1

AGTTCGCGGAGGGCTTTGACG SSRRALT 1

GGGCTGTTCGGTTGCTGGTCT GLFGCWS 1

GGTTTGGAGTCCATGCACCCG GLESMHP 1

GGTAATTGCGGGGCCGCTGCG GNCGAAA 1

GACAGTGGGCCGCAGGCTCTT DSGPQAL 2

CGCGCGTCGGTGTGTTGGAGT RASVCWS 1

TACTCCGGTCGGTGCGGGGGT YSGRCGG 2

TACTTGGTGATGACGTGTATT YLVMTCI 1

TCGGTGATGGTGGAGGCCTGC SVMVEAC 1

GGTCAGGGTCCTCTTTGGAGC GQGPLWS 2

GGGCAGCTCTGCATGTGGGCT GQLCMWA 1

TTCGTCCCTGATCGCGCGGTG FVPDRAV 1

GTTCAGGTCGAGCGGTGTCAG VQVERCQ 1

GCCCTGGAGGGCCTCGCTCAG ALEGLAQ 1

CATAAGTGCAGGCGGCAGCTG HKCRRQL 1

TGGTATTGGCGCGGCGGGGCG WYWRGGA 1

TCGTGCCGCTGCCGGACCGCT SCRCRTA 1

GCTCTGGGGACGCTGGGTCGT ALGTLGR 1

GCGTTCCGGCCTTGCTTCCGT AFRPCFR 1

TGCGTTGTGGCTAGGCTCGGT CVVARLG 1

AACTACCGTCAGGTGTTGGGT NYRQVLG 2

ACGCGGTGCAATTACGTTCCG TRCNYVP 1

CAGTACCGGTACAGGTGGGGT QYRYRWG 1

TGCCTCGATTCTCGGTTTCTT CLDSRFL 1

CGTGGTCCGAGCTCTACTGGT RGPSSTG 1

TTGAGCTTTGTCGCTTCTGCT LSFVASA 1

CTGGCCAGCATGGTCCTGTCT LASMVLS 1

GTTCGGGTCTTGGGTGGTGTG VRVLGGV 1

GTGAGGGTCTCGGAGGGGCTG VRVSEGL 1

CGGAGGGAGGCGAAGTGGAGG RREAKWR 2

TGTTTGGGGAACAGGCGCTGT CLGNRRC 1

TTCTCCTCGTGGAAGCTGGGT FSSWKLG 1

GTCGTGGTCCCCGCTTGGGAC VVVPAWD 1

GTTATTTACGCGCGTATGCTC VIYARML 1

GGGGGGGGCCGGAGGTCGCTT GGGRRSL 1

TCGGGGAGTCGTGCTCAGCCT SGSRAQP 1

CGGGAGTGCCGTGGGCGCTCT RECRGRS 1

ATCCTCTCGCTCTGCTGTCGT ILSLCCR 1

GTGAAGTCGTCCGCTAGGGAT VKSSARD 1

GGCCAGGAGATTGGCGATCAT GQEIGDH 1

TATGGGCCTGATTTCTTGGCG YGPDFLA 1

CGGGGTCGTGTCACGGTGTGG RGRVTVW 1

GTGTTTGTTGCCGGTGACTGT VFVAGDC 1

CGTCAGGGTTTCGTCTTGTTT RQGFVLF 1

GCCGATCGCGAGTTTTCGCCC ADREFSP 1

GTCGGTTGGGTTCTCGTCAAT VGWVLVN 1

CTGTGTGAGCGCTTGGTCACG LCERLVT 1

TTGGGCTCGAGCTGCCGCTCG LGSSCRS 3

TTGAGGTTCGTCGCTCCTGGT LRFVAPG 1

AGGGCGCACGCGTTTTTCCAG RAHAFFQ 1

ATGTGGGTGAGGCCTCGGGTC MWVRPRV 1

CGCCGGTGTTGCTATGTGCTC RRCCYVL 1

TGCATGTACTGCCTGACGGCT CMYCLTA 1

TACGGTAGTTTGGAGTGTCGT YGSLECR 1

CGTGGTGGGAATCTCGATCAG RGGNLDQ 1

TGGGAGGTGAATGCGTCGTGG WEVNASW 1

CGCGATCACGAGGTCGCGCGC RDHEVAR 1

CGTTGGGATGTCATGTGCTCT RWDVMCS 1

GGGCGGGCCTACACTTTGCCG GRAYTLP 1

AGGCTTTGGCGCTGCGCGCGT RLWRCAR 1

TCGTACGGCTGGAGCGCGAGC SYGWSAS 1

CGCATCTGCGGCGGGTCTGCG RICGGSA 3

GTCAGCGATGTGTGCTTTCGT VSDVCFR 1

GATCGTGGCGCCCGTCAGCTG DRGARQL 1

TGGTACGCGGGGAGGGGTGTG WYAGRGV 1

CGGCGTGGTAGGTTGCATGGG RRGRLHG 1

AGGCGTTCGAGTTCGTACAGT RRSSSYS 2

CCGGCGTGTGGTAGGCTCGCT PACGRLA 1

GTGAGCCTCCAGCCCTGGTAT VSLQPWY 1

TTGTGCGCCCCGGATATTATG LCAPDIM 1

CGGTACTCCGGGCGCTTGAGT RYSGRLS 1

CGGCGGACGTGGTCCAGGGAG RRTWSRE 1

GATTATCTGTTCTTCTCTGCG DYLFFSA 1

GTCGAGCGGGTCTATGACTCT VERVYDS 1

GGCGTCCGTAAGCTGATTCTG GVRKLIL 2

GTGGTGCTCCGTGACTCGGCT VVLRDSA 1

GGCGCTCGGAGGCTTTCGGCT GARRLSA 1

TACGGTAACGGTTGCGAGTCG YGNGCES 1

CTCAACCGCGGTGTCGAGGTG LNRGVEV 1

TCGAGCTCTACGAGGAGTTGT SSSTRSC 1

GGTGACGTCGCGGCGAAGAGT GDVAAKS 1

TGCCTGCGGTGGTGCCCTTGT CLRWCPC 1

GTGGACCGGCAGGCTGCGTAT VDRQAAY 1

GTTCTGCGGCGGGGCTGGAGT VLRRGWS 1

GGTAGTTACCGGCGGCTCTCG GSYRRLS 3

CGTTTGGGTTGCATGCGCCGG RLGCMRR 1

TCGACGCCGGCTGGCGGTCTT STPAGGL 2

GAGTGCGTGCACATTGAGTAT ECVHIEY 1

CGCAGTAGCTCGGAGAATCAT RSSSENH 1

TTGCAGACGAGCCGCGTCGAG LQTSRVE 1

GGGCGTCTCCCTTACGTCAGG GRLPYVR 1

TTCGTTATGACGTCCGGTACG FVMTSGT 2

CGGAAGTGCAGTAGGGCTGAT RKCSRAD 1

CGCGACGGGATCGTCTGTCGT RDGIVCR 1

GTGCCCGCTGGTTTCCTTATT VPAGFLI 1

CCGCCTGGTTTCGGCTCGAGG PPGFGSR 1

AGCCTCATCTACAGCCTCAGG SLIYSLR 1

TTCGCGTTGTCGGTCGCTTGC FALSVAC 1

GGGCAGGACTACGAGGTGCTG GQDYEVL 1

TTGTATGCCACGGTTATTCGC LYATVIR 1

CACAGGCGTGATGTCACCAGG HRRDVTR 1

GAGGAGGGGTGCCTGGGGAGT EEGCLGS 1

TGCAGTTACAGCGCGGGTTAT CSYSAGY 1

GTCGTTTGGCAGAGGATGAGT VVWQRMS 1

GGGAAGTGGCCTGGCGGCCGT GKWPGGR 1

GGGCACGACGGGTCGCGTTTT GHDGSRF 1

CTGGCTCGCACTGGGAATCGG LARTGNR 1

GCGGCGGAGGTCTTTGGTTTG AAEVFGL 1

AGCCGCCTCACGTGGAACGTT SRLTWNV 1

AGGATGTACAGTCCCTGTCTT RMYSPCL 1

GAGGCGTTCCGCGCGCAGATC EAFRAQI 1

GCCAATGCTCAGCTCTGCCGT ANAQLCR 1

CGCGGCGGCCGGCCGGGTTCC RGGRPGS 1

CTGCAGCATAAGAGCTTGACG LQHKSLT 1

GTTCTCTGGTGCGGCACTTGG VLWCGTW 1

GCTTGTGCCTGTAACTTGGGC ACACNLG 1

CTCATTGTGGAGGGTCACGTT LIVEGHV 1

AGGAGTCATACTGTGGCTGCT RSHTVAA 1

GTCGACCGGAGCTTGTGGTGT VDRSLWC 1

GCGGGGATGGCGCGTCTTATC AGMARLI 1

TGCCCTGGGACTTCCTCCAGC CPGTSSS 1

ACCGTGTGGCCCTGCAGTGGT TVWPCSG 1

GGTGTGGGGCAGTTCGGGACG GVGQFGT 1

CAGTGGGAGAGGATGGGGCTT QWERMGL 1

TCTTGCGGGATGGACGTGGTT SCGMDVV 1

TGCGGTCGCTGGGGGCACATG CGRWGHM 1

TATGTGCAGTTGCGGTGGGCG YVQLRWA 1

CGGCGGGTCAGCTTTCAGGGG RRVSFQG 2

TGGGATCTCCGTCTCGGGCAG WDLRLGQ 1

GGGCAGTTGGAGTGCGGTGCT GQLECGA 1

CTGCCGCGCGCCATGGGTGGT LPRAMGG 2

CCTGGTGCTCACCTTCGGTGG PGAHLRW 1

CTTGGGGTCAATCACGCCGGT LGVNHAG 1

ACGGGTCGGGGTGCGGTGCTG TGRGAVL 1

TGTGGGTACGTGGTGCGCGCT CGYVVRA 1

CGTCCTCTCTGGGAGATGGCG RPLWEMA 1

GGGATGGTCTTCTATGTGGAC GMVFYVD 1

GTGTATTCTGCTTTGATTTCT VYSALIS 2

TTTGTTGGTCCGGGTTGGAGG FVGPGWR 1

GTTGCTCCGTTCCGTGTGTCG VAPFRVS 1

TTCCGGGTCGGGCTCGCGGCT FRVGLAA 1

GGTTATTCGAACTCTGTTCGT GYSNSVR 1

TGGAGGTTGAGTGTTGGCCAG WRLSVGQ 1

AGGGGTGTGCTGGCTTTGTGC RGVLALC 1

GCGGACGCCGGCGTCACTAGG ADAGVTR 1

TCGTGCGACGTGGGTGCCAGG SCDVGAR 1

GGCTGGCGCAGCGCGCCTCCG GWRSAPP 2

TCTGTCAGCTTTGCCTCTTGG SVSFASW 1

AGTAGTGGGCAGCTTGTCAAG SSGQLVK 1

GTCGTGGACTCGGACGAGTTT VVDSDEF 1

TGGGTGTGGAGCGATCGTGCG WVWSDRA 1

GAGTGGGTTATCCAGGCTGTC EWVIQAV 1

TGGCATTACCGGTCCGCGACG WHYRSAT 1

GCCATGCATGCCGATCGTGGG AMHADRG 1

GGCTGGAGGCTGGCGGGTTTT GWRLAGF 1

GAGGAGCGGCCCATGTGGAGG EERPMWR 1

AGCATGAACAGGTGGGTCCGC SMNRWVR 1

AATGGTGACAACGGGTGGATT NGDNGWI 1

CAGGCCACTTCCGCGCATGGT QATSAHG 1

TACTTCAAGAGCGGCAATCAT YFKSGNH 1

TTTCTCGTCTTTAGGGGGTCG FLVFRGS 1

GGTGGCTTGGCGTTCAGGACT GGLAFRT 1

CCTGCGGGCTGGCGCCGCTAT PAGWRRY 2

TGTGAGCTGTGTCTTGTCCTC CELCLVL 1

GCCCAGACGCTGGGTGTGGTT AQTLGVV 1

CTTCGGAACGTGATTGCCCCT LRNVIAP 1

GCGTCGGGGTTTAAGTATTCG ASGFKYS 1

CCCGAGGAGATGGGCGAGCAG PEEMGEQ 1

GGCGGTTGGTGTGTCGTGAAT GGWCVVN 1

GCGAGTCCCTCTGTCAGGCGT ASPSVRR 1

GTGTCGATGGACCGCGTCACT VSMDRVT 1

AGTAGGCGCCCTACTAGGGCT SRRPTRA 2

GTCCCCCGCTTGGACAGTGAG VPRLDSE 1

CGGCGGTCGTACACTTGGGTT RRSYTWV 1

TGTAGTGGTTACGAGCTCGGT CSGYELG 1

CCGACGCGGGTGCAGGCGGCT PTRVQAA 1

CGCCTGGGGAACTGTTTCTAT RLGNCFY 1

GCGCTTGTTTCCTCGCTTGGG ALVSSLG 1

TGCGTGGGTTCTTGGGATGCT CVGSWDA 1

TGCCTGCACTACCGGGACAAG CLHYRDK 1

CGGTGTTCCAGTGTGCGGTAT RCSSVRY 1

TTGTTTCTTAGCCGCTTGTGG LFLSRLW 1

GATCTTGATATTGAGGTTGGT DLDIEVG 1

TTTATGGTGAGGCTCGTTCGC FMVRLVR 1

CGGCTGCTCAGTGGCCTCGGT RLLSGLG 1

AACATTCGGATTATCACGCGT NIRIITR 1

AGCGGTTTGACCGTGGGCTCG SGLTVGS 1

CCTGCGATTGCGCTCGAGGAT PAIALED 1

CGGGTGCTCAGCGATAGGCGT RVLSDRR 1

GTGCATGTCGCTCGGCTGTTG VHVARLL 2

TCTCGTATGAGCTTTCCCGTG SRMSFPV 1

TGGAATTGCTGGAGGTATGCT WNCWRYA 1

TGGGGGCCCGGCACTCGTGTG WGPGTRV 1

TCGCTCCTCACCATTTGGCGT SLLTIWR 1

GTGGCGGTCTTCCTCTTTGCG VAVFLFA 1

TGGGGTATTCGGAGGCTCACG WGIRRLT 1

GCGTGCAGCCACCTCATTGTT ACSHLIV 1

GGGCATTGCTGCAAGGTTTCG GHCCKVS 1

GCGAACCACGCCGCTAACGTG ANHAANV 1

CATGTCGACGCGACTGCGCAT HVDATAH 1

GATGGCTATGCCTGGCTCCCT DGYAWLP 1

GTTATGTATGCGGTGGTGGTG VMYAVVV 1

TTTGATATGGTGGTCGGCTTG FDMVVGL 4

GGGGCCGTCCGGTTGAATGTG GAVRLNV 1

ACGTTGGGGCTCGCTAGGGGT TLGLARG 1

ACTCATGTCAGGTGGGCCGTT THVRWAV 1

GGCCGTTGGAGGCCCACCAAG GRWRPTK 1

CCGGGCTTGACTTTGGACCGT PGLTLDR 1

ACGCACGGGAGGAACGTGGTT THGRNVV 1

GACGTGGCCTGTTGCTTCCAG DVACCFQ 1

GGTTGGCGGAGCAAGTTGTTG GWRSKLL 1

CGTAGTTGGCTGCACGGCGCG RSWLHGA 1

TTGGTCAAGGTGCGGTCCCGG LVKVRSR 1

GGCGGCCACTGGTCTAGTATT GGHWSSI 1

TTGATTGTCGCGATTGGGGTT LIVAIGV 1

TGGAAGTGGCTGACGGAGGGT WKWLTEG 1

AGGCGGCCCTCTGCTCGGGTT RRPSARV 1

TGGTGCAACCGTTGGAGCACT WCNRWST 1

CTGGCGGCTGTTAAGTGGTTT LAAVKWF 1

AAGTCCTCGGGTCGCGCCGGG KSSGRAG 1

GGTGGTTGGGGTCGCGATCCT GGWGRDP 1

AGGGTGCGTTCTGTCTGCTTG RVRSVCL 2

AAGGACCTGTGCTCGCACCGC KDLCSHR 1

GGGATTCACGGGAGTGGTCCG GIHGSGP 1

TGTGGTTACCCGCTGGGGTGT CGYPLGC 2

GATGCGAGGAGCCCCCCGCGT DARSPPR 1

TTGTTGCAGTGTGCGGGCGTC LLQCAGV 1

CTTCAGCAGTTCACGGGGGCG LQQFTGA 1

GAGGGTGAGAGTACCGTGCTG EGESTVL 1

GTGGTCTGGAGGAATTCGCCG VVWRNSP 1

GCGCTGTGTCTCGTGTGGCCG ALCLVWP 1

GCGTTGTCGTCGTCCCTCACG ALSSSLT 2

AGTCCTAATCCCATGTCGGAT SPNPMSD 1

ACTGACCCTCATCGGACCAGT TDPHRTS 1

GTCGATCGGACCGGTGTGCAC VDRTGVH 1

GAGAGGGCGAGCCTCACGTCT ERASLTS 2

CTTGTGGAGAGGTTCCCTGTG LVERFPV 1

CTGGAGAACTATCTCCACGCG LENYLHA 1

CTTCGTGCTACGCAGCTCTGG LRATQLW 1

ACTCAGTACGAGGTTGTGGGT TQYEVVG 1

GGTAGCCGTATTGGTACTCAG GSRIGTQ 1

CCGCCCAACAGGTTTCAGTTG PPNRFQL 1

GGTGGCTTCGCGCCTAAGGTT GGFAPKV 1

CGGAGGTACGTCGGCAGGATT RRYVGRI 2

CTGGGGTGTGAGCGCATGAGG LGCERMR 1

GCGCGCGAGGCCGGCTGTCCT AREAGCP 1

GGGCTCAGCCTGAGGTCTCAG GLSLRSQ 1

GTCCTCGTGCGTCACCGTCCT VLVRHRP 2

TCGGCGTGTGGGCTCCTTGAC SACGLLD 2

CATTGGTGGGGTCGCTCTACG HWWGRST 1

TTGCGGCGGGGTCAGGCCGGG LRRGQAG 1

GGGGTTGGGGGCTGCTCGGCG GVGGCSA 1

GGTCCCGTGGGCAGGCCTATG GPVGRPM 1

GTGATGGCTGCGTTTTTTCCT VMAAFFP 1

GGGATCGGGTATAGGGCTGGG GIGYRAG 1

GGTGGGCGTTGCTGCTACGTG GGRCCYV 1

GGTTGTCGCTGGGAGGTGCCT GCRWEVP 1

GCGATGATGCGCTGCGCGCCT AMMRCAP 1

TTCGTCACGGAGGAGTCTATG FVTEESM 1

TATTTGTGCTGGTGCCGGATT YLCWCRI 1

CTCGGGGTCGGCTGCTGGTCT LGVGCWS 1

GCTAGGTCGGTGGGTCGGTGT ARSVGRC 1

TGGGTTGGTGACGTCGGCAAG WVGDVGK 1

GGCATCGTCGACCGTGTTGGC GIVDRVG 1

TGTTGGGTCAGGCAGTATCCT CWVRQYP 1

GATATCCGGGACGGCCTGGTG DIRDGLV 1

CTCGCGAATTGGTTTATGTCG LANWFMS 1

ACGCGGACCGGCCGCGTGTCT TRTGRVS 1

CAGACGGGGTTCTACGGCATG QTGFYGM 1

GGCAGGGTCACCAGTGTTGTG GRVTSVV 1

AGGGTTGCCATGTCGCGTGTG RVAMSRV 1

CCGTGGAGCAGCAAGGCTGTT PWSSKAV 1

AGGACCCGTGGGGCCGCGTGG RTRGAAW 1

CGTGTGCCGACCTTGGAGTGT RVPTLEC 1

GTTCGGTCCATGCGTTGGTGT VRSMRWC 2

CTTTGGTCGGCTCCTTATGCC LWSAPYA 1

TCTTGCCTGTGGCGGATGCCG SCLWRMP 1

CTGCAGGGGAGGGACGATAGG LQGRDDR 1

GGCAATCGTTCTCCGTGGTCT GNRSPWS 1

AAGGGGGGTCGTGTGTCTGTC KGGRVSV 1

CGGATCGTCTTTGTCAATTCT RIVFVNS 1

TTGCGGTCGACTGTTTCCGGG LRSTVSG 1

ACCCAGAACTTTGCCTGCGCT TQNFACA 1

CTCCTTTTTCCGGCCAATGTT LLFPANV 1

CTTAGCCGTACGGTCTGGAGG LSRTVWR 1

CGCGACTTGCCGGGGCGTCCT RDLPGRP 1

GCGCTGTTGTGGTGTGTTTGT ALLWCVC 1

CGGTACTCCATTGGTTGGTGT RYSIGWC 1

TGGGGGCATATGGGGCTTGCT WGHMGLA 1

TGTTGTTTCGTTTCGACGTGT CCFVSTC 1

GCTTACCAGGTTAACCTTCAC AYQVNLH 1

TTTCGGGCCCGGCTCAGGTCG FRARLRS 1

TGGCCGAGTATCAGGTGCCCT WPSIRCP 1

TGTGGTGAGGTTCTCTTGGTT CGEVLLV 1

GGGTTTCCTGGCGGTGAGGTC GFPGGEV 1

CGGACGCACGCTTCGGGGACT RTHASGT 1

GTTAGGGTCACTAACAGCATG VRVTNSM 1

GACTTCTGTTCGCGCGCGGTG DFCSRAV 1

AAGGTTGCTCCGAGCGGTGGT KVAPSGG 1

AGCGAGTGGGCCTTCAGGCGT SEWAFRR 1

GTCATGGAGGCGGCCTCGCGT VMEAASR 1

GTCGTTTTTAACTGCGAGGCG VVFNCEA 1

CGGAGGTTGTCGCGGTCTCTT RRLSRSL 1

GTGTTGCGGCGGGCTCCCGGG VLRRAPG 1

TTCCCGTTCAAGACGGACCCT FPFKTDP 1

CGGTGCTCTGTCTACGGGCGT RCSVYGR 2

AGTGTCCGGGGCCTTTTCGAG SVRGLFE 1

TGGGAGCAGCTTGGGGAGTGC WEQLGEC 1

GCGGATTTGTGCAGCGCGCCG ADLCSAP 1

GGGGGCGGCCAGAGGAGCTCG GGGQRSS 1

CGCTATGATGGGTGCTACCTT RYDGCYL 1

CTGGGGTTCGCGTCGAGGTCG LGFASRS 1

TGGCGGGGGAGCAAGCATTAT WRGSKHY 1

GCCCAGGACATGCATCGTCTC AQDMHRL 1

GTCACGTCGGGGTTGGTCACG VTSGLVT 1

TGGAGGGGCCCTCGCTGGGTT WRGPRWV 1

TGTTTCTTGCTGGCTGGCTCC CFLLAGS 1

AGGCCCATTGCGGTGCTTCAT RPIAVLH 1

CTGCTTGTGAGTCTCCGTGAT LLVSLRD 1

CCTCTTGGGTGGCCGGATCGG PLGWPDR 1

TTTGGGTACGTGGCGCTGCGG FGYVALR 1

CATGCGCCTTCTCATCGTAGT HAPSHRS 1

CGGAGGGTCAGCTGGGTCTCT RRVSWVS 1

GTGTTCGGGATGCGGAGGTCG VFGMRRS 1

CTGTGCGGGTTGCGCAAGTCT LCGLRKS 1

CACGGCGCGCGGTACTTTCCT HGARYFP 1

CAGGGCGACTCGGCCCTTCGC QGDSALR 1

CCGAGGCTCGGCCAGCTCTGC PRLGQLC 1

TCTGAGCCCATCTTGGGGCGC SEPILGR 1

CACATGAGGGTCTACAACTCG HMRVYNS 1

GTGATGAGTGATTGGTGGTGT VMSDWWC 1

TCTCAGGGGATCTTGGCGGCT SQGILAA 1

ATTGACGCGTTTATCTCTCGG IDAFISR 1

GTTGTGTTGTGCGACCTCGTT VVLCDLV 1

GGTTCGATGGGGCTTGGGAGC GSMGLGS 1

GGTTGTCCCCTTAGGACTGTT GCPLRTV 1

TATCGGGGCGGCGGTCATAGT YRGGGHS 1

CCGAAGGATGTTTGGTACGCT PKDVWYA 1

CCGGTGCTGGTTGGTACTGAT PVLVGTD 1

AGGCAGAACGGCTGGAGCCCC RQNGWSP 1

CGGCCCCGCTGCTTGCTGCCT RPRCLLP 1

GTCAGGTCCGCGTACGTTTGG VRSAYVW 1

ATGTTTATCGTGTTGGCTTGG MFIVLAW 1

GTCCCTGACTGCGCGAAGCGT VPDCAKR 1

GATCTGGCTTACGACGTGAGG DLAYDVR 1

TTGAGGAATTGGCATCGGGGC LRNWHRG 1

GCGGGGGACTGGGGCTATAAT AGDWGYN 1

GCCTCCTGGAGGAGCTGTTGG ASWRSCW 1

GGTGGGATTCCGGAGTTGCGG GGIPELR 1

TTCGTCATCACGGTCTCGGCT FVITVSA 1

CCCGAGTGCCAGGTGGATCCT PECQVDP 1

CATCGCCGGGTGACGAGCTTG HRRVTSL 1

AATGGGTGGGTGTTCCAGTGT NGWVFQC 1

AATGCCCCGAGCGTGGTGTCT NAPSVVS 2

ATTTGCCGCCGCGCGAGGCAT ICRRARH 4

CAGCACGTTAGGCTGCTTATG QHVRLLM 3

CTCGGTGCTTGTCCTCCGCAT LGACPPH 1

GTGGTTCTCGCGGGCTTTACG VVLAGFT 1

GTGCTCGACAGGTGGCAGGCG VLDRWQA 1

CCGTTGCGCGCGAGCGGGACG PLRASGT 1

AGTCAGGTTTACACTGAGTCG SQVYTES 1

GTTAGGGCCGCCGAGCTGAGT VRAAELS 2

GCTGGGGAGCGGTTCAGGAAG AGERFRK 1

CAGGCGCGTATTGTGAGGATG QARIVRM 1

TTGCAGCGGTGGCAGGTCACT LQRWQVT 1

GGTTGGCGGGTGTATGTCCGG GWRVYVR 1

GCGCGGACCGTCATCCGTTCT ARTVIRS 1

CTGGGCCGTGTCCATCACGCT LGRVHHA 1

ACGAGCGTGAACGAGCGGTCC TSVNERS 1

CATTGGAAGAATTTGGGTCTT HWKNLGL 1

TATCATGGGATCGTGCGTACG YHGIVRT 1

GCCGACGGGGTCGGGTGGTGT ADGVGWC 1

GGGCCGTGTGTGTTCTCTTAT GPCVFSY 1

CGGGCGTGCTGTGAGGCGGCT RACCEAA 1

CAGTGGCTTTTGGTCGCTGCG QWLLVAA 1

CCGTTGCTTCACCTGTTTCCT PLLHLFP 1

CAGTGGGGGTCTCTTGTCAGT QWGSLVS 1

GTGTCGGTCCATGGCGGTGCT VSVHGGA 1

GTCGTGTGCACCGATAATGCT VVCTDNA 1

TCTCGGATGGACGTCCCTTGG SRMDVPW 2

GTGGGGAGGTTGTTGGGTCGG VGRLLGR 1

AGGGCGCCGTATGTGCGTGTT RAPYVRV 1

GTGGTGCGGCGTGTCCCTCTG VVRRVPL 1

CTGCAGCCCGCGATCGCGCTT LQPAIAL 1

CGTGCCGCCTCCGGTCCTCCG RAASGPP 1

CGGCTTGCGACGTCGGCCCGT RLATSAR 2

GTCGGTCGGACCTCCAGCAGG VGRTSSR 1

GTTAAGCTGATGCCCAGGTTG VKLMPRL 1

GTCGGGTGGGTCTTGATCCAG VGWVLIQ 1

TATCGTCAGTTCCACTGGTCC YRQFHWS 1

AACAATGTTCGGACTGTGGCG NNVRTVA 1

CGGGTTGCGACCATTCGTCTG RVATIRL 2

TGTGAGGGTGACGTCACTGGT CEGDVTG 1

TTTCGGTCCTGGAAGGTGCTG FRSWKVL 1

GGCCATACGGTGTCCATTCAG GHTVSIQ 1

CGGTCGGGTGGTCGTATTGCT RSGGRIA 1

GGTAACGGCAGGACTCACTAT GNGRTHY 1

ATGGAGTGCAGGGTTTTGTTG MECRVLL 1

ACTAAGCAGCAGCGGCGGGAT TKQQRRD 1

GGTCGCGGCTCGTCGCTTGGT GRGSSLG 1

GGGATGGTTTCCTGTAGGGCT GMVSCRA 1

CAGAACACGATCAGCTCTAGG QNTISSR 1

ATCGGTAACTCTGACCCGCCC IGNSDPP 1

CATGGGCCCCGTGTCCATGCT HGPRVHA 1

TGGTATCCGAAGGAGCTTCGC WYPKELR 1

TGTCAGGGGATTGTGATTCGG CQGIVIR 1

GTCTGTGGCCGCGTCGTGCAT VCGRVVH 1

TCCCGCGTGCGCTCTCATGAT SRVRSHD 1

CTGTTCAATTTGCGCGCCCTT LFNLRAL 1

TACGCTAGCGTGCGGAATATT YASVRNI 1

GGGGTGATGCGGCTCGGCCGT GVMRLGR 1

ACCTCGTGCTCCCGCTTGCCT TSCSRLP 1

CAGGGGGTGGGCTCTACGGGC QGVGSTG 1

GGCGACTTCTTCGTGTTCGTT GDFFVFV 1

GGCGAGGGCTGGGCGCCTAGG GEGWAPR 1

TCGGGTTCCGGGGCGTGGTGT SGSGAWC 1

TGTGTCGGGCCTTTGGCTGGG CVGPLAG 1

GGCCGCTTGCGGTTGACTCGC GRLRLTR 1

CCCCGCGTCTGGTCGCCCATT PRVWSPI 1

CGGCTCCCGGCCTTCTGGGGT RLPAFWG 1

GTGTGTCGCTGGAACCCTTGG VCRWNPW 1

TGCAAGTGGGATTGTCCGGGG CKWDCPG 1

TTGAGTTGTGAGCTGGACATT LSCELDI 1

CTCTACGTGCCTCCCTTGGGG LYVPPLG 1

TGGGAGCTCGGTTGCTGGTGG WELGCWW 1

ATGGGGTGGAGTGTCTGGTTG MGWSVWL 1

TGGGAGCGTAGCGGCTCTCGC WERSGSR 2

CAGGGGACGGGCGACAAGGCG QGTGDKA 1

ATGCACTACAATGAGAGCCTT MHYNESL 1

TTCTGCGCGTGCGGCCTCGTT FCACGLV 1

AGGGTGGGGAGGTCCTGGGTT RVGRSWV 1

CTGGGGGTCTGTCCCGATCTG LGVCPDL 1

AGGTTCGCTAATGGGAGCGCC RFANGSA 1

TTTGAGGGCCTGTCGATGTGT FEGLSMC 1

GTTGATCGGACGGCGCGCTTG VDRTARL 1

CTTTGCGCTATGCACGTCCAC LCAMHVH 1

GGTCCGCGCGCGAGCAATGGG GPRASNG 1

GGGGAGAGGTTTGTCGACTCT GERFVDS 1

TGGACGGTTTGGTGGGTCTGT WTVWWVC 1

CAGCGCCGCACTGGGTGCTGG QRRTGCW 1

GCGCCGAAGTCTTTCATGTCT APKSFMS 1

GGCCGCGTGTGGGGCACTGTT GRVWGTV 1

GCTCCCGGGACCCACTGCATT APGTHCI 1

CGGGGTTGCTATGCCGTGCTG RGCYAVL 1

ATCGTTGGGGCCCTGGGGATG IVGALGM 1

GTCGGTGGGATCCGTGTTGCT VGGIRVA 1

GGGAGTGTCGTTTTTGACACT GSVVFDT 1

TGCCTCTGTAGTGCGAGGTTC CLCSARF 1

GATTGTTACACGTGGTCTGAG DCYTWSE 1

TGCCGCTGCCGTCCCTCTGAT CRCRPSD 1

GGGTGGCTGCGGTTGTGGGTG GWLRLWV 1

TGGATTGAGTCGGCCCACCTG WIESAHL 1

TGGGTCCAGTTCATGGTCTGT WVQFMVC 1

AATCATAATGCCGGGTTGGGC NHNAGLG 1

AACGTGGCGGGGTGCGGTAGT NVAGCGS 1

TCCTCTGGTACGGCCTACAGT SSGTAYS 1

CTTTCGGCGTTCCCGGTTCGG LSAFPVR 2

ACGCCCCGCGCTCGGTTCGCG TPRARFA 1

TACTCTCAGCGCTTTAGGCTC YSQRFRL 1

CGTGGGTCCTGTTCGGGCGGC RGSCSGG 1

GTGCAGGCCCGTAACGATGTT VQARNDV 1

CTTTACACGTCTGAGGCCAGG LYTSEAR 1

GATCTCCTCGGGAGGCGGTTG DLLGRRL 1

CACAGTTGTACGGGCGGTGAG HSCTGGE 1

ATGTGCTGCGTGTGCCGGTGG MCCVCRW 1

GTGCGTGTTCCTCGGTCGCGG VRVPRSR 1

TTCCTGGGCGGGGCTACTTAC FLGGATY 1

AAGCTGGCTAAGAGCGTTTAT KLAKSVY 1

AGGGTGAGGTGCTGTACGTGT RVRCCTC 1

TTGCGCGGGGAGTGCAATATT LRGECNI 1

TTGCTCACTCGGCCTTCGAGT LLTRPSS 1

CAGCCGCGTATTAATGGCGCT QPRINGA 1

CGGGGGCATGTCTTGAGCGGG RGHVLSG 1

GCGAAGCTGGACAGCGCGGAT AKLDSAD 1

CTGCGCTTCACTCTGAGTCCC LRFTLSP 1

TTTTTTGCGACCCATGGGTGG FFATHGW 1

GTCGCCGGCTGCGGTGGGGAG VAGCGGE 1

ACGGTTAGGTGGACGATGTAT TVRWTMY 1

CTTGGGCTCGGCGGGAAGGGG LGLGGKG 1

GTGAAGCTCTGGCCCTCTCGC VKLWPSR 1

TCGGTCGGCTTCCTTTTCCGG SVGFLFR 1

AGGCATTTTGCTACTCGTCCT RHFATRP 1

GAGGGTCCTTGGATCGGTGAG EGPWIGE 1

GATCGCGACGGGTGTGTGTCC DRDGCVS 2

GCCGCCGGTAGTCCGCATTCT AAGSPHS 2

TTGAGCGTTAGCGCTCTCTCT LSVSALS 1

TTGTCGTGGCGTCGGTGTCGG LSWRRCR 1

CTGGCGTGGCCGGTGGCTTGT LAWPVAC 1

GCCTCGTACACTAATAAGGCT ASYTNKA 1

GGTTGTCGCGCGGCGGCTGGG GCRAAAG 1

TGGCATGGTAAGTCGGTGTCG WHGKSVS 1

GGCGAGGCCCGCTATACCGCT GEARYTA 1

GAGTCGGGGGGCAGCCTGCCG ESGGSLP 1

TTGCTCGTTAGGCCCGGTGCG LLVRPGA 1

GTTGATACGGAGCATTCCAGG VDTEHSR 1

ACTGCGAACTTCTGGTTTAGC TANFWFS 1

AGTGATGCGATGTGTCATTGG SDAMCHW 1

CTGATCTATGCTGCGGGCCGG LIYAAGR 1

ACCACTGCGGCGATTCGGACT TTAAIRT 1

GTGGTCGAGAGCTCCAGGTTG VVESSRL 1

TACCAGGCTAAGGTCCGGCGT YQAKVRR 1

TTCCAGTTCCGGGTCCATCCT FQFRVHP 1

TGCCAGTTCTGGTCCTGCTCG CQFWSCS 1

GTTGGCGCTTGGGCTCGGCGC VGAWARR 1

CGGGCTCGCGAGAAGTTCGGT RAREKFG 1

TCTCTCTCTCCTCTGCAGCAT SLSPLQH 1

GTCATTGTTTGGTTCACCGGT VIVWFTG 1

CGGGCGGTTAGGATGCCGTGT RAVRMPC 1

AACAGGGGTGCTAGGGGCCGG NRGARGR 1

GCTTGGGGGCGGCCTGGTATC AWGRPGI 1

GTTTGGTGGAGGTCGTTGCTG VWWRSLL 3

CGCGCGCGGGTCTTGTGCTCT RARVLCS 1

AGGCCGTTGGTCCACACTTAT RPLVHTY 1

ATGGGCGCGGCGGACGTGCGT MGAADVR 1

ATCGTCGACCTCCACTTCGGT IVDLHFG 1

ACGGGTGCTGGGGCCGGTCCT TGAGAGP 1

CAGTCCTGGTGCTGGCTTCGG QSWCWLR 1

CGTAAGTGGGGTAGCGAGACC RKWGSET 1

ACGGCTCCGAGGTGCATTTCC TAPRCIS 1

CAGGCCCCCTGCCCTATTGTG QAPCPIV 1

TTGCTGTGGTCGATGCGTTGT LLWSMRC 1

GGTCAGGTCCATGACGGTTGT GQVHDGC 1

GTGGATCCCTTCGGCCTGCTT VDPFGLL 1

CGCGCTGGGGTGGGTCGGCGG RAGVGRR 1

CGGTTCCAGGAGCCGTGTAGG RFQEPCR 1

TATGGCGACCATTCTCTGTCT YGDHSLS 1

TTGGTCTGGGCTTGTATTAGT LVWACIS 1

TTGCTGGACGCTGGGGTGATT LLDAGVI 2

TTGACTCTGCGCCTCAATGGG LTLRLNG 1

GTTCTCATGTGGAGTCAGACT VLMWSQT 1

GTCGCTTGTAGTTCCCGGAGT VACSSRS 1

TGTGCGTACAGGATTTCGCTG CAYRISL 1

GCGGGCGACTGCGGTGGGTAT AGDCGGY 1

ATCATCCTTGGGGATACTTGT IILGDTC 1

GGGTTGGATTTGTGCTTGAGT GLDLCLS 2

CGTGCTCGTGATCGGTTTGGT RARDRFG 1

GTGGCGTGGAGCCGCTCTGAT VAWSRSD 1

GTCGGTCAGTTTGGGGCTGGG VGQFGAG 1

GTCGTGACTTGGACCTTGGTC VVTWTLV 1

GTCATTCCGACGGTCTGTGCG VIPTVCA 1

CTGATGCGCGTCTGCAACTCT LMRVCNS 1

CGCCTCTACGTTGTGCGCGGT RLYVVRG 1

CGCTTGCAGAGCCAGGGCGAG RLQSQGE 1

TATGCTCTCGGTGCTACCTTG YALGATL 1

GAGTACCCGCGCTTGAGCTTT EYPRLSF 1

TTTCAGAAGAGGCTGGCGCTG FQKRLAL 1

CGCCGGGAGACTCGGAGCTGG RRETRSW 1

GATGTGTTGTGGTCTCGCCCG DVLWSRP 1

GACTGGCTGGCGGGCTGGCAT DWLAGWH 1

GTTGCGCTCAGCTTGCTGCGT VALSLLR 1

ATCTTCTTTTGTGCCTGGGGT IFFCAWG 1

CAGTTTCTGGTTCAGTTGAAC QFLVQLN 1

GAGCTTTGGGGGGCCCGGGTC ELWGARV 1

GGTATCGTCGGTCGTGGCACT GIVGRGT 1

ACGTTCAGCGGGTCGCGCGTG TFSGSRV 1

TCGGCGAGCCGCGCTTTCCTT SASRAFL 1

TCTTCGACTGCTAGTAGTTAT SSTASSY 1

GGTGTGGACTTCTGTCCGATT GVDFCPI 2

GGTCGGTTTTCGAGGCAGTCG GRFSRQS 1

AAGGTGCATCTCCGGCCTCGT KVHLRPR 1

GTGGTGACCTGCTATCATAGT VVTCYHS 1

AGCGGCGAGCGCTGTTCTGTT SGERCSV 2

TGGCACACGAGCGTGGGTGCG WHTSVGA 1

CGGTGGAGGATGCTCGCGGCG RWRMLAA 1

ATCGTCGGGGCTGCGTACGTT IVGAAYV 1

GGCCGTCCGGATAGTTTCGCT GRPDSFA 1

AGGTGCCTCAACATGGTCATG RCLNMVM 1

GGTTGGCCGCAGCACGTCGTT GWPQHVV 1

TTTTATCCTAAGTTCACCAGT FYPKFTS 1

TTGCCGTGCTGTTGCGACGAG LPCCCDE 1

GAGGCTCCTGTGATCGATCGG EAPVIDR 1

TCCGTGTTGAGCGGTGCTAGG SVLSGAR 1

CCTCTGGCCAATAGTTCGCCT PLANSSP 1

GGGGAGCCCGGTATCCATGGT GEPGIHG 1

GTTTATACGACTCTGCAGCAG VYTTLQQ 1

ACTTTGGGCCGGGGCGTCCAG TLGRGVQ 1

CTTGGGATCACGAGGGGCCCG LGITRGP 1

GACCCGAGTCCGAGCCGCATT DPSPSRI 2

TTTCGCACGGGTTGGGCGCTC FRTGWAL 1

AGTGGCGCCGTGGACTGGCAG SGAVDWQ 1

TATACCGGGTTCAATGATCGT YTGFNDR 1

GGGCGCGATTCTATCGAGAGT GRDSIES 1

AACGGGGCGATGCTTCGGTTG NGAMLRL 2

GCGTTGAGGGCGTCCACCGCG ALRASTA 1

ACGCTCTGGGAGATTGTGACC TLWEIVT 2

TATCAGCGGAGCTATAGGTTG YQRSYRL 1

TTGGGGTGCAGCAATCGCTGC LGCSNRC 1

GGGCGCGTTGCTTCTGAGCAG GRVASEQ 1

GTTGTGGAGACTGGGGGGCAG VVETGGQ 2

GATCTGCGCGTGTTCTCGAAT DLRVFSN 1

TGGTTGTTCAGGTCCTCCGGG WLFRSSG 1

TATTGGCCGAAGCTGGTCGGC YWPKLVG 1

GCCGACCGTCGTGGGGACTCT ADRRGDS 1

TCTGTCTGGCGGGGGCTCGTT SVWRGLV 1

ATCGTGGTCGGCGGGCTCCTG IVVGGLL 1

ATGCGGTGTGCGGCGATGGCT MRCAAMA 1

GACCACGGGCTCGCGGGTGTT DHGLAGV 1

TGTTCTCTCGAGGGCGCTGGT CSLEGAG 1

GCGCGGAGTCTTCTGGCGTTG ARSLLAL 1

GTCTATGGGCGGGTCGAGCCT VYGRVEP 1

AGCGTCCGGAGTCGGATGACC SVRSRMT 1

GCGTATGGGTCCAGGTTGATT AYGSRLI 1

TGTCTTCGCGGTGGGTGCAAC CLRGGCN 1

TCTGGTCTCGGCCGCGACAGC SGLGRDS 1

GACCCTAGGCAGCCGTGTGAT DPRQPCD 1

GGTGGTAGCCGTGCTGCGCTG GGSRAAL 1

AGCATGGCGGTCCGCTGGCTC SMAVRWL 1

CATCGGGAGTATAGCGCCATG HREYSAM 2

GGGCGCTGGGCCGTCATTGCG GRWAVIA 1

GAGACCTGTATCGACCCGATT ETCIDPI 1

TCGAGGTCTAACCCGGGGCGG SRSNPGR 1

GTGTGTATCCCGCCGGTCCGT VCIPPVR 1

CGGGGTCTGTCGCAGTGCGAT RGLSQCD 1

CTCCTGCCCCGTCTCGGCGAT LLPRLGD 1

GGGTCCGGCACTGCGTGTTGT GSGTACC 1

TGTTTGTCCTCCTGGGGTTGT CLSSWGC 1

TGTTGGCAGATTGTCTGCCAG CWQIVCQ 1

CACGCGGTGACTCGTTGCAGT HAVTRCS 1

CGGAAGGGCAGCGCCCGTGCT RKGSARA 1

GTGCATAGTCTCTCGCTTACT VHSLSLT 1

TTCCGGCCGCTGTGGGGTACG FRPLWGT 1

GCTGCTGGCCGGATGGAGCAT AAGRMEH 1

GCGGGGGTTGCGGGCACCTTT AGVAGTF 2

GCTCGGGCCACCAGGCACGGG ARATRHG 1

TTCTGTGTGTTCCCCGGTTCC FCVFPGS 1

AGGGTGGGTCTGGTGGCGGAG RVGLVAE 1

GAGGCGTGGCGTTGGCTCCGT EAWRWLR 1

ACGGGCTTCATCGGCTTCCGT TGFIGFR 1

CTGGAGGTGCCCTTGACGAGT LEVPLTS 1

TCGATTGGCCATCATTGTTGT SIGHHCC 1

TGTTCTGGTGTGCGCACGGCT CSGVRTA 2

AGTGTTAATAGTCTCTGGCGT SVNSLWR 2

TATTCCTGGCTCGCTGTCGTT YSWLAVV 1

CATGTGCGTTTCTGCGGTAGG HVRFCGR 1

CGGACTAGCGCGCTCGCGCTT RTSALAL 1

GCGTGCGCCCGCGGGAGCGAC ACARGSD 1

GGGGAGTTCCTCAGGACCGAG GEFLRTE 1

ACTGACGGCTGTTGGACGCTT TDGCWTL 1

CGTGTGTATGATACTAATAGC RVYDTNS 1

GGGGTCCGTAGTAGTGGCCCT GVRSSGP 1

CCTGTCCAGTCGGCGCGGAGT PVQSARS 1

AGGCGGGCCGATCCTGGTTCG RRADPGS 1

GTTCCGGGCGGCGTCGTGCGT VPGGVVR 1

CGTGTGTGCAAGACGACCCGG RVCKTTR 1

GCTTTCTATTGTCTTAATCAT AFYCLNH 1

GCTGCTAATCTTGGCAGCGGC AANLGSG 1

GGTATGCGGCAGGGTTGTTGC GMRQGCC 1

GGTCCCAGTGACTGCCGTGCT GPSDCRA 1

GAGTTCAGCCAGTTTGTGCCC EFSQFVP 1

GTGAACTTCCTGTTCCGCTCG VNFLFRS 1

GGCATCCGGAGGTGCTGTCCG GIRRCCP 1

CCGCCTTCGCTCGACGGTCGC PPSLDGR 1

TGCGACGGGGACAACTCGGCG CDGDNSA 1

GTCTCGCGTGGGCATGTTGGG VSRGHVG 1

CCTGGCAAGGGGAGTCTTTGT PGKGSLC 1

GGTTGGGGTTATCGCTGGGAT GWGYRWD 1

TGGTCGCACTACCGGGTCATG WSHYRVM 1

CTCCTCCGCTCGGTTAGTATT LLRSVSI 1

ACCTCCACCACCGCGAGGGTT TSTTARV 1

ACGGGGCGGATGAGGGATGTG TGRMRDV 1

GTTAAGTCGGGGCACGTTTTG VKSGHVL 2

GCGCCGGTGGTCAGGCCTCAG APVVRPQ 1

AATGGGGACTTCGGCATGCGG NGDFGMR 1

AGGGGTGCGCTCGCTTGGGGC RGALAWG 1

GGTAGCGTTAACTGCGTGTCT GSVNCVS 1

CAGATTGGGCGCCCGAGTCAC QIGRPSH 1

AATGTTGCCACGCACACGATC NVATHTI 1

AGCGAGCCGGAGAGCCCGGAG SEPESPE 1

GACATTAGCAGTTGGCGGTCT DISSWRS 1

TTGCGTTTCGGGCGCCTGGCC LRFGRLA 1

GGGTCGGACTCCCGTATGGGG GSDSRMG 2

CTTGTGTCTCTGTGGCCTGAG LVSLWPE 1

ATGGATCTGGGGTCGAACCGT MDLGSNR 1

GATCGCCGTACCGGTTGGCGG DRRTGWR 1

GGTATTGGTCCCTGCTGTCAC GIGPCCH 1

GTCACTGAGATCCTTTGGTGG VTEILWW 1

CTGTCCGTCAGTAAGTCTATG LSVSKSM 1

TGTGACGCCGCCTGCTTTATG CDAACFM 1

TGGAAGCGGTGTGGGTGCGCT WKRCGCA 1

CGGGATCCCACGACCTCGCCT RDPTTSP 1

GGTCCTGTCAGGTTCCTCGCG GPVRFLA 1

AAGTCCGAGGCCTGCGTTAGG KSEACVR 1

ACTGCTACTTCGCCTACGGAT TATSPTD 2

TGGCGTCGCGGGGACGAGCCG WRRGDEP 1

TTCTTGACCAAGTCGCTGGTG FLTKSLV 1

AATGTGCGGTTTTGTAGTGGC NVRFCSG 1

CCGGGTGCCTTGGAGTTTCGG PGALEFR 1

GCTACGAGGACGGCGATTTCT ATRTAIS 1

AACACTGGGACCTTGGCCCTG NTGTLAL 1

GCTGGCTGCATGTCGCGGTCG AGCMSRS 2

GGGTGGAATCGTGGCTCTGTC GWNRGSV 1

ACGGACTGCACCTCGACCGTT TDCTSTV 1

CGGCGCGATCTGCGCCCGGTG RRDLRPV 1

GGGCTGCTTGCTGGCTTCCAG GLLAGFQ 1

CACGGTAGTGCGGGTTGGCTG HGSAGWL 1

CTGGTTGCCGTGTGGGTCCCT LVAVWVP 1

CGGTTGTGCAGCCAGGAGCAT RLCSQEH 1

GTGTGTTGGGCTGAGTGCCAG VCWAECQ 1

GGTTGCCGCTACAGCGTCACT GCRYSVT 1

ATTAGCCCCCTGACCAGGGCT ISPLTRA 1

ATGACTAATAACTTGTCCATT MTNNLSI 1

GCTCGCTTCTGCCTGACGTGT ARFCLTC 1

AGGTGGCTGCGGTGTTGCCTT RWLRCCL 1

TGCCTGGATCTGGACTATCGG CLDLDYR 2

AGTCCTTTCGTCGGTCCGGTG SPFVGPV 1

AAGCTCTGCGCCGGTGCGGAC KLCAGAD 1

TGGAGCGACAACCAGCGGTTC WSDNQRF 1

CTTTTCGTTAGGACCGGGTGT LFVRTGC 1

CGCTGGCACAACTGGGATTCG RWHNWDS 1

AGGCTTCGTGTGGAGCGTTGG RLRVERW 1

TGTCAGCGCAGCATGGAGTGT CQRSMEC 1

GAGGGTACTAGCCATGGTAGT EGTSHGS 1

GACTCCTTCTCGGACAGCTTT DSFSDSF 1

TACCCTGTCTTGCAGGGCGCG YPVLQGA 1

TTGTGGGTGTCGGGGTGGCCG LWVSGWP 1

GCCCCTGTTAGGGGGTCTCAG APVRGSQ 1

GGCTGTCACGCGGAGTGCCTC GCHAECL 1

CCGGGGACGTGGCTGTATCTG PGTWLYL 1

CAGTACCTGTGGTCGGGCCGT QYLWSGR 1

TTTGGGGTCTGGGGTGTGGTT FGVWGVV 1

AGTCCTGTGCAGTGCGAGCCG SPVQCEP 1

AAGCCTTGGCATCGCTCTAAG KPWHRSK 1

CGCGGCGCCCGCATGCAGCCG RGARMQP 1

TCTTCCTCCCTTAGCGTGACG SSSLSVT 1

CCGCGTGCTGGCTGGTATTGT PRAGWYC 1

CCGGGCTTGGCGAATTCGTGG PGLANSW 1

CTGTGGCGCATTGTCGGTCCG LWRIVGP 1

TTCATTATGACTTACACGGTG FIMTYTV 1

AAGCCGCACTGTTTCGTGACT KPHCFVT 1

CCGGAGATGCTGCGTGGCGTT PEMLRGV 1

CTTGGGCTGCGCCAGCTGTTG LGLRQLL 1

CGCGGTGTGATCGTGTTGGGT RGVIVLG 1

AGCCTCAGCAGGCTCTATCGT SLSRLYR 1

CTTATCGGCCGGCGCTGTGGT LIGRRCG 1

GTTGAGGCCACGGGCGGTAGT VEATGGS 1

ATCGCGCCGGTCTTGGGCTTG IAPVLGL 1

ACGCAGGGTCGGTTGTTCGTG TQGRLFV 1

TTTTCGGCGGTCGTCTGTCCG FSAVVCP 1

CAGCGTATGGGTTCGGCCCTT QRMGSAL 1

ACGTCGTACACCGCGCTGTGG TSYTALW 1

GTCCAGCTGGAGCTTGGCACC VQLELGT 1

GGTTTGAATGGCACGTCTGCT GLNGTSA 1

CTCGGTTTGGGCGGCCCTCCT LGLGGPP 1

ATTCCGGGGTGCTGTTCTCAG IPGCCSQ 1

TCCGTGTCGAGTGTGACTTTG SVSSVTL 1

TGTCGTCGGGGGTTCTGCGGC CRRGFCG 1

CCGCTGGTGCAGGGCTTTAGT PLVQGFS 1

TGGGCGTGCGGTTGGGGCGTC WACGWGV 1

GTGAGCGGGGAGAAGAACCCG VSGEKNP 1

CTTACCTTGCGCTGTCCGTTG LTLRCPL 1

CGCATCCGGAAGGGCCTTGTG RIRKGLV 1

GTCCAGGGCACCTCGCGTGTT VQGTSRV 1

TGGTGCGTTAGCGGGAGTTGG WCVSGSW 1

CGGCGGACGGTGTTCCGCACT RRTVFRT 1

ATGCAGTTGATGGGTCGCTTT MQLMGRF 1

CACCGCAGGCTTGGTGTCCGC HRRLGVR 1

TCTGGTGCGAGCGTGTTTCGG SGASVFR 1

ATTTGGATGGGTTCCGGTAGG IWMGSGR 1

CCTATTCATATCGCGGGGCAT PIHIAGH 1

TACTGTGGCTTGTGCGGGGGC YCGLCGG 1

GGGCTTCCGTGTGGGCTTGTG GLPCGLV 1

GGTGCCCAGCGTGCGGTTGTG GAQRAVV 1

CAGTGTGCTTGTCCCTTTAGT QCACPFS 2

GCGGTTTCTTTGATTTCCCGG AVSLISR 1

GCTCTTGGGGACCCGGAGCGT ALGDPER 1

GCTAGCCGGAGTGACCTGAGG ASRSDLR 1

TGGTACCGGTGGCGTGGCGCT WYRWRGA 1

CTGGTGTCCACGAGGCAGCAG LVSTRQQ 1

GCTAGGGGGCCCATGGTGGGG ARGPMVG 1

GTGGCTCGGGGGAAGGTCAGG VARGKVR 1

GGTGGCCAGTGCGCGGTTGCT GGQCAVA 1

CGTTTTGACTTCAGGCCGTCT RFDFRPS 1

CCGCCCAAGGGCAAGGACTCG PPKGKDS 1

GTGAGGCAGACGTGGCTTGTG VRQTWLV 1

CTGTATCTCGAGGGCTGGCCT LYLEGWP 1

CCTGGTGCTCTGGGGTGTGAG PGALGCE 1

GACGCGCCGGGTCTCTATGCT DAPGLYA 1

AGGCGGGATGTTCGTCACTCG RRDVRHS 1

AATTCGGGGTCTGCCGTGGGT NSGSAVG 1

TCCACCGAGAGTCTTACTGCT STESLTA 1

TTTAGGACGTGCTGGAAGACC FRTCWKT 1

GCGGGCTCGAGGTCTGGGCCT AGSRSGP 1

ATTTGGGGCTCTGGGACTCGG IWGSGTR 1

GGGATGGTGGTCTTGTATGTT GMVVLYV 1

GTTCATCAGGATGCGGGTGGT VHQDAGG 1

TTGGACTACTGCTGGTACAAT LDYCWYN 1

CATTATCGGGGTCAGCTGTTT HYRGQLF 1

TGTCCCGTTACCATGATCCGT CPVTMIR 2

CAGTTTCCTCAGACTGGGTTG QFPQTGL 1

GCGTTGGGGAGTCATTGCGCG ALGSHCA 1

GGGCCGTGTGTTGGGATGCTG GPCVGML 1

CCGATTTGGTCTTCGCAGACG PIWSSQT 1

CGGGGTAACTCCGAGAGGTTC RGNSERF 1

TCCTCCCGCTTGGGCGGTGGC SSRLGGG 1

CCTGTTCGGGCGCGGTTGGAC PVRARLD 1

GTTGCGGCCTGCTGCTTTGGG VAACCFG 1

TTCGTTGCGGTTGCCTGCCTT FVAVACL 1

TTGTCGACGTGTCGTTTCATC LSTCRFI 2

AATCCTCTGTCCTTGGCGCGG NPLSLAR 1

CCTCGGGGTGGCCGTGCGGGT PRGGRAG 1

AGCTGGGACGTGAAGAATCTG SWDVKNL 1

GTGGTTTTGCGCTCTACGCAT VVLRSTH 1

TCCCCGACCGGCGCGGGGTTT SPTGAGF 1

TTCGTGGGGTTCTTCGTGGCG FVGFFVA 1

GTCGTCAAGCAGGAGGTTGGC VVKQEVG 1

CCTGTGCGTCTGGAGCTTAAC PVRLELN 1

CCGTCTCCGAAGCAGCCTGTT PSPKQPV 1

CATGCTCTGGTCAGTGCGGGC HALVSAG 1

TTCGGGGTCTCGAGGGGTAGT FGVSRGS 1

TGGGCGCCCATCTGGTGTACT WAPIWCT 1

GCTTCGCGCCTTACCGCGCGG ASRLTAR 1

GTGTTTTGGGTGACCGAGGAT VFWVTED 1

GATTGCTTTTACAACGATATT DCFYNDI 1

AAGCGGGCCTGCTTCGCGACT KRACFAT 1

TTCCTCCGCGGGGTTGCTGTT FLRGVAV 1

GGGCTCCATACGGATGTGTGT GLHTDVC 1

TGTGCGTGGCTCGACGGGGCT CAWLDGA 1

TGGCCCATCGTGAGGAACGCG WPIVRNA 2

GCTCGGTACCAGTACATGTCT ARYQYMS 1

CTGTCCTTGCGGCAGAGCCCG LSLRQSP 1

TTCTGTGGCACGTGGTCGCCG FCGTWSP 1

GCGCGCATGGCTCGCCGTTGT ARMARRC 1

CGGTTCGTGTCTTGTTTTGGC RFVSCFG 1

CACTCTAGGGCTACTGAGGGT HSRATEG 1

GGGTCGCAGATCGCTATCACG GSQIAIT 1

GGGTTGAACACGAGGCCTCAT GLNTRPH 1

GTCGACGGGCATTGCTGGGCG VDGHCWA 1

AGGGGCTTTAGCCGCGCCGCT RGFSRAA 1

GCGTGGGTCATCCGGTCGGCT AWVIRSA 1

TCTTTTCAGGTGTTCGGTTTC SFQVFGF 2

GTCGGGTGCAGCTATCTCCCT VGCSYLP 1

CGTCAGCGGTCTTTCTGGAAG RQRSFWK 1

CGGGTTGTCCTCGCGTGCGGG RVVLACG 1

TGGGAGGTTCCGGTCGTCAAG WEVPVVK 1

CGGGTTTGCTGCTCTGAGCGG RVCCSER 1

GAGTGTTATCTTGGGATGGCG ECYLGMA 1

AACTACCCCCAGCGCCGCACC NYPQRRT 1

GGCTCGCTGAACGGGATGGGT GSLNGMG 1

CCCTGGATCAGTGGCCCCGGG PWISGPG 1

TCGGAGAGCCCGGGCTCTGAG SESPGSE 1

CGGGGTGGTGCTTGGGACGTT RGGAWDV 1

TTGGTGGCTCTGTACGCCAAG LVALYAK 1

TGGTGTGTCACCTACCGGGCG WCVTYRA 1

TTCCGCGAGTGGGGGTCCTGT FREWGSC 1

ACGCGCGTGTATCTTAGTCAG TRVYLSQ 1

GGCATCTGCAGTACGTTGCCT GICSTLP 1

GCTCGGCTCTGTTCCGGGCCC ARLCSGP 1

CTTGTCGCGCTTGGCGGTGGG LVALGGG 1

CATTCTGGGTTTGTCATTTCT HSGFVIS 3

CGCCGTGGGCCGGAGGAGCCT RRGPEEP 1

CTGGCCATTTACACCGGGTGG LAIYTGW 1

CGCAGGTTCGGGATTGGCCAT RRFGIGH 1

TGCTGGTGGTTCCCGCTCTAT CWWFPLY 1

CTGGGGATTGTCTTCCCCGGT LGIVFPG 1

TGCTGCTCGGCCCATCGGAGG CCSAHRR 1

GTGCGGTGCCCGCGCTTGAAG VRCPRLK 2

GCCGGTTTGAGTTCGCTCCAT AGLSSLH 1

CGGTGCACCGCCTTGTGGCTG RCTALWL 1

ACTGATTCCGCGTCCGCGTGG TDSASAW 1

AGGAGGGTGAGCTCTGTTGTG RRVSSVV 1

TATGGTGCTTTCGGTGCCTCG YGAFGAS 1

TACGCGGAGATGCGGGTTGTT YAEMRVV 1

GGCCAGCGGACGCAGACGCGT GQRTQTR 1

GCCTTGCTCATCACCGTCGCG ALLITVA 1

ACCTTGCTTACGAAGCTGCGG TLLTKLR 1

TGGTCCGTCACTTCCAGCATG WSVTSSM 1

TACATGGGGCCGGGGAATTAT YMGPGNY 1

TACGTGCTCTGCGGCTGGGTC YVLCGWV 1

CGTAGTCTGGACGGGCTCGTG RSLDGLV 1

GAGGGCGTCCGCGCCTGCCCT EGVRACP 1

CGCCGGCAGACCGATCGCTGG RRQTDRW 1

GGGCGCCTGAAGTCCACTTTG GRLKSTL 1

TTGGTCGCGGTGTGCACGATG LVAVCTM 2

GTTGCCACGTGCTGTAACCCG VATCCNP 1

ATGCTGCGGGGCGGCGGGCAT MLRGGGH 1

CATTGTCGGTGTTACGGGCAT HCRCYGH 1

AGTTTTCGCAGCGTCGAGTCT SFRSVES 1

TTGCCTGGCGAGTGGACTGCG LPGEWTA 1

CTCCTTGGCCTGCCGAGGTGT LLGLPRC 1

ACCTCGGCGCTTAGCGCTTCG TSALSAS 3

CGCGGGGACCACTTGTTTGGG RGDHLFG 1

CCTAGTCTGATCAGTTGTCAG PSLISCQ 1

TTGAACCGTGGGGGGCATACT LNRGGHT 1

GGGTTTGGCCGCGGGTGTCTT GFGRGCL 1

GGTGTGTTCGGTCTTGCGTCC GVFGLAS 1

CTTTTCCACGGCCGGCCGTAT LFHGRPY 1

GTCCGCATTTTCGCCTCTGGG VRIFASG 1

CTCCCGCCTGGTGCTATGGCC LPPGAMA 1

GTGGGGCTTGTTTGCGTCTAT VGLVCVY 1

CAGGGTACTGGGAATGCGGGG QGTGNAG 1

AGTCCGTTCGGGTGTGGTCGG SPFGCGR 1

GTTCTCGTGTGCTGCCTGCGG VLVCCLR 1

TTTAAGTTCCAGATTTTTGTC FKFQIFV 1

AGTACTCGCAATTGGGGGGAT STRNWGD 1

GTTCTTCGTAGTCGGCTGGTG VLRSRLV 1

GTTGTCATCCTGACGCAGTTT VVILTQF 1

ATCTTGGGTTGCGCTTTCCCG ILGCAFP 1

GTCGGGCTCGCCAACCGCTCG VGLANRS 3

GCCCCTAACGTCCTTTGGGCT APNVLWA 1

GGTGCTTCCAGGGTTTGTGAT GASRVCD 1

TGCGGCGGCCCGTCGGTGTCG CGGPSVS 1

TGGCGGATTCCTGCCTCTGGG WRIPASG 1

TTGTACGGGCCGATTGGCGAG LYGPIGE 1

GGGTCGGGCGAGGGGAATGCG GSGEGNA 1

TCCGTGCCCGGTGGGAGTTGT SVPGGSC 1

CAGACGTGTAGTTTGCTTACC QTCSLLT 1

GTGTGGCCCTGCCTGGGTCTG VWPCLGL 1

TGGGTGGTCGAGGCCATCGGT WVVEAIG 1

GAGAGTTCTAAGGTCCCGTGG ESSKVPW 1

TATGGGTCGGTGAGCATTGCT YGSVSIA 1

CGCGGCCATTTGGAGGATCTG RGHLEDL 1

TGTGCGTGTGCCGTGTACAAG CACAVYK 1

GTGTTGAAGCCGCCGACCGCT VLKPPTA 1

ATCCGCGGCGCTGTGATGGGG IRGAVMG 1

GTTTGGCGCCGGTCCGTTACT VWRRSVT 1

AGCTCGGTGGCCCCCGATTCG SSVAPDS 1

TGTTCGCGGCTGCCTACGCAG CSRLPTQ 1

GTTGGCCGCGAGGCCGGCGCT VGREAGA 1

GTGGGGTGTCGCCTTTCGGCT VGCRLSA 1

CGTCGGGGTAGCGGGTCTAAG RRGSGSK 1

GCCTTGGCGAGTGCTGACCAT ALASADH 1

TGTGCGGGCTCGAAGTTGAGT CAGSKLS 1

TCTGGCTGGCCCTTCGGTGCT SGWPFGA 1

TGTCAGTTGGAGTGCTGGCGT CQLECWR 1

TTGGGGGGTGTCATGAGGTCT LGGVMRS 1

CGCTTTAGTAGTAACTCGCCG RFSSNSP 1

TTGCAGCAGCACCACTGCTGT LQQHHCC 1

GAGCGTCGCACTGAGCTGTGC ERRTELC 1

CCGGCTATGCGTGTGAGCCGT PAMRVSR 1

GGTTGGGGGGCGGGTGCGCGC GWGAGAR 1

CTGTGCGAGACCTTTTCGGGG LCETFSG 1

TCCTGGTGCAGGCCCTGCCAT SWCRPCH 1

TGGCAGGTGAGTCCGCGGCGT WQVSPRR 1

CAGGAGCCCGTCTTCGTGTGG QEPVFVW 1

GCGGTTCGCATTCGGGCGCGG AVRIRAR 2

AATTTGCGGTTGGGTCGCCGT NLRLGRR 1

ATTGGTCTCGCCGGGGCTGTG IGLAGAV 1

AATCTGCGGACCCTGTTTGTG NLRTLFV 1

GGCGTTTGCGTGAGTTCGCTT GVCVSSL 1

TGGTGCGAGCTGAGTCTGGAT WCELSLD 2

GGTCTGGCCATGGCGCGGGGT GLAMARG 1

TGGTCGATCAACGGTGCGTAT WSINGAY 1

TGCGATATGAGTGCGCACGCT CDMSAHA 1

GGCCGGTATTCTTTTGGGAGT GRYSFGS 1

TATATCCGTCTGCGCCGCCCG YIRLRRP 1

AGCTGCGACAGGTCTATTAGT SCDRSIS 1

GCCGGGTTTGGCAGTTCTCAT AGFGSSH 1

CTTTGGGTGCTTTGGTTCTCG LWVLWFS 1

GGGTCTGCTAGCCGGTCGTCT GSASRSS 1

TGCGATTGCGCCCGTCGTTTT CDCARRF 1

GCCTCGCTTGACTTTAACCCG ASLDFNP 1

CGCTGTCGGGAGGTTCTGAGT RCREVLS 1

GGGCTGTGGAGTGGTGTGAGG GLWSGVR 1

GCCGGGGCTGCCGCCTGGCTT AGAAAWL 1

AGTCGTCACAGGGGTCTTGTT SRHRGLV 1

GGTCCCCCCTGCTTCAGCATC GPPCFSI 1

AGCGTGCGGTCCGGCACGGCG SVRSGTA 1

CGGCGTATGGGCGTCAGTCCT RRMGVSP 1

GGGCTTTTCATCTGGCGTGCT GLFIWRA 2

CGCCTGGATGGGCGGTGGCTT RLDGRWL 1

TGCGGGTGTGACCGGCTCTAT CGCDRLY 1

GCCGTCGAGCTGGTCGGCCAG AVELVGQ 1

AGCGGCGGGCCTCTGTTCTAC SGGPLFY 1

GCTGCGATTGAGGTGACGCGG AAIEVTR 1

GTCGTCCGCATGGGGCTTTTG VVRMGLL 1

GGTGTTCTCTATCCGTTCATC GVLYPFI 1

TCCCGGGGTAGTGTGGCGCCC SRGSVAP 1

CGGCCCTGCAGGGTCAGGTAT RPCRVRY 1

GTTGCCAGCGGGCCGTCTTCG VASGPSS 1

TTGCTGTCTAGGGTTTACGTC LLSRVYV 1

AGGTTGGGCGTTGGCACTAGC RLGVGTS 1

GATGGTGAGCTGGTGGGTGGC DGELVGG 1

GAGGATTACAGCGCGTGTCAG EDYSACQ 1

GCTGTCTTTGTGTGTAGGGAC AVFVCRD 1

TTTCAGCGGCGTGACGTGCAC FQRRDVH 1

TATCCGGCTAAGTCGAACTGT YPAKSNC 1

GCCTGGTTGGATGATGCGCGG AWLDDAR 1

GAGTCTCGGGGCCACTTTGGG ESRGHFG 1

TTGGGGATCATGTGTAAGGCG LGIMCKA 1

CGGCGGTGGCTTGGTTGGGGG RRWLGWG 1

ACCCCTTGGGTCCGGGTTATC TPWVRVI 1

GCTCGTGGCATCGGGTGCGTT ARGIGCV 1

GTTCGCACCGAGGGTGATTAT VRTEGDY 1

AGGTACTGTGCGTGCTTGGGT RYCACLG 1

CGGTTGGATGGGAGTTTGCGT RLDGSLR 1

GTTGGCAGTGGGACTTTGGGG VGSGTLG 1

GGGTCGCCGTGCGCCAGCCAT GSPCASH 1

TGTCGTGGGCGTGGCCGCCCG CRGRGRP 1

GCGCGTCCTGGCGAGGGCAGT ARPGEGS 1

GTTTGCTCGGTGCGTTGTCGT VCSVRCR 1

CGTTCCCGCGCCCATTGTCTT RSRAHCL 1

CTGCCTAGTCGCCTGGTGCCT LPSRLVP 1

CCGCCCTGGTTCCGCGTGACG PPWFRVT 2

CTCAGCCATAGTCCGCACAGG LSHSPHR 1

TTGGCCGTCTTTTCTCGCGAG LAVFSRE 1

CGGCAGCAGGTGGGTGGTGGC RQQVGGG 1

GCGGTGGTCGTGGCTAATGAT AVVVAND 1

CTTTTTCGCACGGGCGTGGGT LFRTGVG 1

GTTAGCTCGCGCCAGGGTCAT VSSRQGH 1

GTGGTTAGCGGCGTGTGCAGC VVSGVCS 1

ATGCTGGCGGTGGGCAATCGT MLAVGNR 1

GCCTTGTATCCCCCTTTTGCG ALYPPFA 1

GGCCGCATCAAGATTCCGTAT GRIKIPY 1

GTTGAGCGTGGCGGCGGCAGT VERGGGS 1

GGTGGCGCTATGAGCTCGGGT GGAMSSG 1

GCGACTGGCCCGGGTGCTTCG ATGPGAS 1

GCCTGGGTCCGGTTTAGGCCC AWVRFRP 1

GACTGTGGCTCCCGGCGGACT DCGSRRT 1

CCGCGCCCTATGTCGGTCGGC PRPMSVG 1

TCCGAGAGTCTCCGGCCCGTG SESLRPV 1

CACTTGCTGCAGTCCTCTGTT HLLQSSV 1

CCTGGTGTCGATGTGCATCGG PGVDVHR 1

CAGGGGTGGCCGAGCGAGGCT QGWPSEA 1

TCGTACAGCAACAGCGGGTCG SYSNSGS 1

GGTATCGTTTGCGGTTGTAAT GIVCGCN 1

CAGTCGCTGTGGTGCGCTGTG QSLWCAV 1

CTGCTTGGGCTGGCGGCTGGC LLGLAAG 1

TCGGCGGTGAGTGCCCGTAGT SAVSARS 1

AGTTATCAGGGTTTCGGTATC SYQGFGI 1

TACGAGTGGCGCGGGGTCGAT YEWRGVD 1

GGGATTAGCGCGGTCGTCCGG GISAVVR 1

TTTTGCGATATCGAGCTTCTG FCDIELL 1

GGGCACTGTAGTCGGATCCCT GHCSRIP 1

CAGAGTTGGCAGCGTGCTTGT QSWQRAC 1

CAGGTTCGGGGGTCTCCTCGT QVRGSPR 1

TGCACGGTCGCCCGCGTCGCT CTVARVA 1

CGCGTGCATATGAGGGAGCGT RVHMRER 1

CCTCGGAACTTCGGGGTGGTC PRNFGVV 1

TACTTTCGGAGCGCTTGTGAC YFRSACD 2

TGGGAGGCTAATTGGGATGCT WEANWDA 1

ACCGCTGTCATTTCTCCTTTG TAVISPL 1

CATGGTGGTGGTGGGTCGGCT HGGGGSA 1

GTGTTCTGTCGGTTCAGGGAT VFCRFRD 1

GGGGCCGCTTCGTTTTTGACT GAASFLT 1

GCGTGTGTTTATAGGACTATT ACVYRTI 1

ATTGTCTTGACGGACGGTGGT IVLTDGG 1

GGTCGTTTGAGGGAGTGTGCG GRLRECA 1

GTCTGTATCTTTAATAAGCGT VCIFNKR 1

ACCGCGTGGGTGGCCGTGGAC TAWVAVD 1

GTGATCACCTCTCGGCAGTTT VITSRQF 1

GGGCCGCACGTGGCCGGGTTT GPHVAGF 1

GTGATGAGTGGCCAGAACGCT VMSGQNA 1

GTGGCTCCGGGGGTCCTGCGT VAPGVLR 1

GTGGTGCTGGCTTTCTATACT VVLAFYT 1

AAGGGGATCAGGCTCGACGGG KGIRLDG 2

GTGGCGCCCTTCGAGAGTGCG VAPFESA 1

GGCTTGAGGACTTCCAAGCTT GLRTSKL 1

GGTAGCACGCGCGGTGGCCTT GSTRGGL 1

CGGTATGAGGTGCTTCAGGTT RYEVLQV 1

CGGGTCACCCGGGGCGAGGCT RVTRGEA 1

CCTTTGTGCGTGTTCGTGGTC PLCVFVV 1

TCGCAGTTGGGTTTTACCTCT SQLGFTS 1

TATCGCTGGTTGGGGCGCGGT YRWLGRG 1

GACGGCGCTTTTCGCTTGCAT DGAFRLH 1

CATAATTCGAGCTGCCGGTTG HNSSCRL 1

ACTAAGTCGTCTCGCAGGTGG TKSSRRW 1

CCCGTTGGTCTTATCTGTGCG PVGLICA 1

GGTTGGTGCTTTGTTGGTAAT GWCFVGN 1

CGCAGCAGTGGCAAGGCGCAT RSSGKAH 1

GTTGTCCTGTCGTCGCCGTCG VVLSSPS 3

GGGAAGGCCATCGAGCTCATT GKAIELI 1

CTCCGGCTTGCCATGCTTTAC LRLAMLY 1

GTGGGGAGCACGGGCAGCGCG VGSTGSA 1

CTTACGCGCTCCGACGGGTCG LTRSDGS 1

GCGTCTAGTAGCTGTGGTCGG ASSSCGR 1

GGCTCGGTGCGTAGCGAGATG GSVRSEM 1

CTGCTTGTGTTGTGCCTTGTG LLVLCLV 1

GGTATGGAGGCCGTTCTGTGG GMEAVLW 1

GGTTTCGCCATCGGTGCGTTG GFAIGAL 1

TGGTTGGGGACGGACCTGTAT WLGTDLY 1

TTTCACCTGTGGAACGCTGTG FHLWNAV 1

GATTGGGCCGCCATTTCCTTG DWAAISL 1

GTGGCTGTTCGTCGCAGTGGT VAVRRSG 1

TACCTGGTTCTTGCGGGTCGC YLVLAGR 1

CATAACGTCGGCTACGGGCAG HNVGYGQ 1

GGGGTCCGCCAGGTGGTCGTC GVRQVVV 1

TTCGGGGGTGGGTCGCTTGTT FGGGSLV 1

AACAGTCAGATGGTTGCGTTG NSQMVAL 1

CGTGTTCACGGTGAGGGGGAG RVHGEGE 1

CCGGCCGTTAGCCCTTTCTAT PAVSPFY 1

CACGTTTGGTATGTGTGCCTG HVWYVCL 1

TGGGAGCGCTGGTCGCTGTTG WERWSLL 1

CCCCCGAGGAGTACCCCGCGC PPRSTPR 1

CGGGACCTTTGGGTCCAGGCT RDLWVQA 1

CTGCGTGGCCCTCGGTTCGAT LRGPRFD 1

GTTGACCGCCCGATTCACAGG VDRPIHR 1

TTGATCTGGTGCATTTTGCGT LIWCILR 2

CAGCGGAACGACTCCGGTATG QRNDSGM 1

GCCCTCGCTTTGTCTCCCAAC ALALSPN 1

TGCAATAATTTGTCCAGGGGG CNNLSRG 1

CCTGCTGCGTGGGGTACGGCT PAAWGTA 1

CCGACTTGCAGCCACGGGGTT PTCSHGV 1

TTGCGTTTGACCAGGGGTTGT LRLTRGC 1

GGGCTCAACGGCTTCTACTAT GLNGFYY 1

GGGTGCACGGCCAGGGGTCCG GCTARGP 1

GGGCAGTGCGGCGGGATGGCT GQCGGMA 1

TTTTATGGTGGTAGCTCCCGG FYGGSSR 1

CGCGATGTCTCGGTCAAGCCG RDVSVKP 1

GGCTGCGACTCTGCGTTGACC GCDSALT 1

ATGCTGCTGGTTTACGACGAG MLLVYDE 1

CGGTCGCGCTGCGCCGGTCAG RSRCAGQ 1

TGGCCTGCCTCCGGCAGGGAG WPASGRE 1

CGTGGTGCGGAGGGCTCTTGG RGAEGSW 2

CTCGTGCACTCTTTTGGTGCT LVHSFGA 1

CGGTTGATCCAGCGGGGGCTT RLIQRGL 1

CTTGGGATCGGGTGTATGGTT LGIGCMV 1

GGGCTTAGCTGGGCTCACACT GLSWAHT 1

GCGCGTTTGAAGTTGGCGGTT ARLKLAV 1

GCCATCATCCGCCGTGTCGAT AIIRRVD 1

AGGATGGTGGTGTTGAATACT RMVVLNT 1

GTGCATTTGGTGCCTAGGCGT VHLVPRR 2

ATTGTCCGGACTCGCTTTGTG IVRTRFV 1

ATTTGGACTGTTCGTGTGGTG IWTVRVV 1

AAGCCGAGGCTGCTGGGCGCG KPRLLGA 1

GAGGGGCGCCTGGGTATTACT EGRLGIT 1

TCTCTGAACGGCACTGCCGTC SLNGTAV 1

ATCTTGGTTAGCTCCGCGACT ILVSSAT 1

TGGGTTTGTTGCCACTGGGAT WVCCHWD 1

GTCGGTCGCCAGCTCTGTTGT VGRQLCC 1

CACGGTTCCCAGGCTCGGGGT HGSQARG 1

GGCATCCCGGTCCACAATGGG GIPVHNG 1

CCGGGGTCGAGCACGACCAAT PGSSTTN 1

CCTATCATTACGAGGCACTAT PIITRHY 1

CGCTATCATAATGCTCGGGGG RYHNARG 1

AGTAGCCTGGGCGCCTCTCGG SSLGASR 1

ATTTCGGTGACCGCCACTATG ISVTATM 1

TCCCGGGTCAGGGGCGACCCG SRVRGDP 1

CGGTGGACCTGGTCTTTGCTT RWTWSLL 1

CATGAGCGGGCGAATGGGGGG HERANGG 1

TACTATCGCCCGGGGACTAGG YYRPGTR 1

ATGTCGGGGAGGCCGGATTGT MSGRPDC 1

ATCTGGGTCTTGGCCGTGTCG IWVLAVS 1

AATCCGGGGTCCGGGCTGTTG NPGSGLL 1

AGCGCCGTGAAGATGGTTCTC SAVKMVL 1

CAGGGGTGTAGTTGGAATCTT QGCSWNL 1

TTGTCGGTGTGCTTCATCTGC LSVCFIC 1

TGCGAGGGTTTTACCCTGCAT CEGFTLH 1

GCCCATCCGACCATCCTTGCT AHPTILA 1

GTGTGCCGCTCGTGCGCGCCC VCRSCAP 1

CTGCGGTTCAGCTGGTTGGGT LRFSWLG 1

GGTCGGTCGGCTGCTGGTCCT GRSAAGP 1

TTCTCGCGGAGGGCTGGGGGT FSRRAGG 1

GTCCAGCGGGTCGGGATTCAC VQRVGIH 1

AACCTGGGCAGTTCGTTCGGT NLGSSFG 1

ATTAAGCCCAGCGGCGGGCTG IKPSGGL 1

TTGGGTCAGGCCAGTTTGATT LGQASLI 1

CTCTTGATGCTTACCGGTACG LLMLTGT 1

TCGCTCCGGAAGGAGTCTCCG SLRKESP 1

CGGCGGTCGACCGTGGTTATT RRSTVVI 1

GGCTGGGTGCTCTCCGAGGGG GWVLSEG 1

CGGGGTCTTGGGAAGGTGATG RGLGKVM 1

ATGTCCGGCTTGGGGCTGTAT MSGLGLY 3

CGCGCGTACGTCGGTGCTCTG RAYVGAL 1

TCTCGCTTGGCTTCGAGGGAT SRLASRD 1

GACGCGGTTTCTTGCGCTTGC DAVSCAC 1

GGCTGTTTTAGCGACGATTAT GCFSDDY 1

CTGGAGTTCCTCCGCCCCAAG LEFLRPK 1

GTGGTGCGTCTGTCTCGCTTT VVRLSRF 1

GATGCGATCATGCGCCATCGG DAIMRHR 2

AATTTTTTCAGTAGGGTTTCG NFFSRVS 1

CGTGCTTGTATTGGTCTTAGG RACIGLR 1

AGGCGCCCTCCCAACTGCTTG RRPPNCL 1

GACAGGCTGAAGTGGCCTGGC DRLKWPG 1

GGTTTCGCGGAGTTCCGGTTG GFAEFRL 1

TCTTGGGTCCAGGCCGGGACT SWVQAGT 1

TTGTGTTGCAGGGAGCGTAGC LCCRERS 1

GGTCTTGAGGCTTTCATTAGT GLEAFIS 1

ATGGGGCGGACGCTGCTGGCT MGRTLLA 1

TTCCCGAACACGCGGGCTGAG FPNTRAE 1

GTGTCGGGGCGTTTGAGCATG VSGRLSM 1

TTGGCTGCGTCTGAGTCGTTT LAASESF 1

GGTTCCGTTTGGCTGCAGCGT GSVWLQR 2

CCGCGGCTGTTGACGCATCTT PRLLTHL 1

TTGAGGCCCTTGCTGAACGGC LRPLLNG 1

TTGGATACGTTTTGTCTGCTT LDTFCLL 1

GCTGTGTCGGGGAGGGGTGTT AVSGRGV 1

ATCCGGCCTCTGTTCCAGTCG IRPLFQS 1

TGTTTGAACACGCCCACGCGT CLNTPTR 1

AGCTGGTGGATCTGTAGCCCG SWWICSP 1

GTGCGGCGCTGGAGTTCTATT VRRWSSI 1

TGCTGTATGCGCTGGAGTCTT CCMRWSL 1

CAGGTGCTGAGGAAGGGCTGT QVLRKGC 1

CGGTGGAATCACTGTAGGGGG RWNHCRG 1

GACGGGGGGACGCGTGATGGT DGGTRDG 1

AAGGCTGCGTGCCTGGCCAGG KAACLAR 1

GGGTCCTTTCCGGCTCTCCGT GSFPALR 1

GGGGACTTGATCCTCCTTCTT GDLILLL 1

CGGCGCGGGGCGGGCTTGACT RRGAGLT 1

CGGTGCATGAACTCCGATGTG RCMNSDV 1

GACGGTGCCGGTCGCAAGGTT DGAGRKV 1

GACTATAATATGGGCACCCCG DYNMGTP 1

TCTGAGCACTCTGCTTGGCGT SEHSAWR 1

TCCCTGTGTGCTGCGGTTTCT SLCAAVS 1

CAGGCCCGCCGGGAGGCGTGC QARREAC 1

GCCGGGCGTCGCGGGCTGACT AGRRGLT 1

CGTGGGAGCGAGCAGATGTTT RGSEQMF 1

TGTCTCGCGGTTGGTAACCGC CLAVGNR 1

CCGAGGGCCGGTGCTGATACT PRAGADT 1

TCGGTCAGCAGCCTGTGGACT SVSSLWT 1

AGGGGCGACGGCGTGAGGCCT RGDGVRP 1

GAGGACGACGGGCTTCACGCG EDDGLHA 1

GTGTCCCCCACCATCTCCACT VSPTIST 1

TGGTTGGTCATTTCGTTCTAT WLVISFY 1

GTGAGGGGGACCTTGAGTGGT VRGTLSG 1

TCGCGTTTGGGCGGGTTTCAT SRLGGFH 1

GCGGAGTTCACGGGTGGTCTC AEFTGGL 1

GTGCACGGTGAGGCGGGTCCT VHGEAGP 2

TGTGAGGTCGGTTGGTATCTT CEVGWYL 1

CACGTGGGTTGTGGCATGCAG HVGCGMQ 1

GGTCTCGTCGCCTTCTCGACG GLVAFST 1

GGTCAGCGCGGCGGTGTCAGG GQRGGVR 1

AGGGGGACCGGCTGGTCTTAC RGTGWSY 1

CGCGCTCTGAGGGTCAAGTTT RALRVKF 1

GATCCCGGGCTGAGGGTGGTT DPGLRVV 4

GGGCTGGTTCGCGTGAGGGTG GLVRVRV 1

GGGGGGGGCTCCCGTGGGGTT GGGSRGV 1

GCGCGGGTTTGTTGTCCGGTT ARVCCPV 1

CCTTGGCTTCCGTGCGTTTGT PWLPCVC 2

CGGTGCCGTTCGGTCCGGTTG RCRSVRL 1

GTGACCCGCGGCAACACTGGG VTRGNTG 1

ATGGGGGGTGGTGCCAGGAGT MGGGARS 1

CGCGGGGCTGGGATCAGGTGT RGAGIRC 1

TTGCGGTTGCACGTGGTCTCT LRLHVVS 1

GCGTGGAAGTGCTTCGAGTGC AWKCFEC 1

GGGGGGCAGTGCGCGGGCTCT GGQCAGS 1

GCGTCTCGTAGCGTGGGCTGT ASRSVGC 1

GTTGTTGGTACGTGCGCCCTG VVGTCAL 1

TCTCTTTGTCCGAGTTGTTCG SLCPSCS 1

GGGCTGAGCAGGGACATGGGT GLSRDMG 1

GAGGGGTATGCCCCCGATTGT EGYAPDC 1

AGGGAGTGGGACCGCCGGACT REWDRRT 1

CTCGTTCTGTGGTGCTGTCCT LVLWCCP 1

CACGTTCCCACTCTGGGTGCT HVPTLGA 1

GCGTGGCACGGGGGGAGTCAT AWHGGSH 1

CGCTGGGGGGAGCGCGGGCGT RWGERGR 1

AGTCGTCCGAGGAAGGTCGGC SRPRKVG 1

GGGCCTCCTCGGGAGGTTCCT GPPREVP 1

GTGTTCGACTTGTCGAGCCAG VFDLSSQ 1

AGCGTGTATTGCCTCAGCGTT SVYCLSV 1

GTCTCTACTGTCTGCTTGGAT VSTVCLD 1

TTGCAGGATATTTTGTTCGGT LQDILFG 1

CCGTCGCGGTGTATGGCTACT PSRCMAT 1

GGGCTGGGTCGGGCCAGGTGT GLGRARC 1

ATGGGCACCTCGTTTTCCGAG MGTSFSE 1

CTCGGTTACAACTGTTTGATT LGYNCLI 2

GTCAGGGCGGTGTGCTCGGGG VRAVCSG 1

ACGCTGGCGCGGGCGCTCTAT TLARALY 1

CGGGCGGGGTCGGGGCGGGCT RAGSGRA 1

CACGCTCTGAGCGTTGGCTCT HALSVGS 1

TATTGCGGCGAGTTGCGCAGT YCGELRS 1

TGCGAGCTTAGTTTGGGTTGT CELSLGC 1

CTGCGTTTCTCCGTGTGTGTT LRFSVCV 1

GAGTGTCGCAGTGCGCTCGTG ECRSALV 1

TCTGACCCTGCGCATGGCGAT SDPAHGD 1

GTGTGTGTCTGGTTGGAGGGT VCVWLEG 2

GGTTGGGCGGATAAGCTGGGG GWADKLG 1

GTGAGGTACATGGGCGTGCTT VRYMGVL 1

CGCTGGCTCGCGGGTGCTCTG RWLAGAL 1

TTGAGCACTGGGAGTAACTCG LSTGSNS 1

GTGTACGTTTGCCTGCTTCTT VYVCLLL 1

AGTGGTATTGATGTCTTTGCG SGIDVFA 1

GCTGTCCGTATGTATCCCGTT AVRMYPV 1

ATGCCTCAGGCGGCGCCGTGT MPQAAPC 1

CTTCTGCTGTTCGCTATTTTT LLLFAIF 1

GATCTGCTGTTTTGGGTTGTG DLLFWVV 1

GAGGTGGATCGTGTGGTCAAT EVDRVVN 1

AAGCGGAGCCTGAGGTCGGGT KRSLRSG 1

TTCACCCGCCGGCTCGCTACG FTRRLAT 1

CTCGGTCGTCGGTGTACGATT LGRRCTI 1

GCCTATCGGTTGAGGTCTCGG AYRLRSR 1

GTGGGTCGTATTACGGCGTCT VGRITAS 1

TTCCCCTTTCGTGGGTTTAGT FPFRGFS 2

CCTGGGGCGGGCTTTATCACT PGAGFIT 1

GACCGGCTTGCCGTTTGTTGG DRLAVCW 1

TTGCACCCGGGGTCGCATGAG LHPGSHE 1

ATGGTTGGGAGGTTGTCGGTG MVGRLSV 1

TTGCTGAGGGAGCCCGGGGTT LLREPGV 1

GTCGTTCATTCCAATTTCGCG VVHSNFA 1

GGCGCTGTTCGCTGTTGGAGT GAVRCWS 1

GACCGTGGGCTCCGCGGGTCT DRGLRGS 1

CACCAGGTCCGCACGAAGCGG HQVRTKR 1

TGCGGGCGCGCTATCTATATT CGRAIYI 1

GCTTGCTGTACGTTGTGGGCG ACCTLWA 1

GGTTGTGGGTGGCACTGTGGT GCGWHCG 1

GGGGAGCATGCTGACCTGGGG GEHADLG 1

AGGGTTGTTCTCGGCCTGGGT RVVLGLG 1

GGGGCGTGGAGGGGTAGGATT GAWRGRI 1

TGGGTCTGTGTTAGGCGTGGT WVCVRRG 1

AACGGCAGCACTGTCTGGGGG NGSTVWG 1

TCCCCGGGCTTCAGCTTTAAG SPGFSFK 1

TTCAAGTCCTGGACCGGGCAC FKSWTGH 1

AACCGCGGTGCGCGTCTCACG NRGARLT 1

CAGTCTCAGAGGTGGTGGTGT QSQRWWC 1

GGGTGGCGGTTGCGCGGTCTT GWRLRGL 1

AGTCTCCGCGTGCACGGTTGT SLRVHGC 1

CGCGGTGTGAGGACTTTGCTC RGVRTLL 1

GAGGGGCCTTGTTGGGGGCCC EGPCWGP 1

CTGCTGCGGACGGTGCGTTGT LLRTVRC 1

GTCCGTAGCGGGCGGGGCCTC VRSGRGL 1

TGCTTGTATCAGCAGCGTGCT CLYQQRA 1

GAGATCGGCTTGGTCGCGGAT EIGLVAD 2

AATTTCTGGCGTGGGAACCGG NFWRGNR 1

GCTCGCAGGTTGTGTAATGGT ARRLCNG 1

CTGTCGGTGCATCATAAGGTT LSVHHKV 1

TTCTCTCCGCTGGGGGTTTAT FSPLGVY 1

ACTGTTCCCAGGTTGCGTTTT TVPRLRF 1

GTGCTGGGCAGCTGGACGTCG VLGSWTS 1

CCCGTTGTTGTGGGCAATGGT PVVVGNG 2

TACTGTCTGGCCCTGTGCGGC YCLALCG 1

GGGGCCGAGCGGAGGGGGACG GAERRGT 1

TATCGGTGGGCGAGCCTTGTG YRWASLV 1

GCCCTGACTTTTCGGGGTGTC ALTFRGV 1

GGTAGGCTTAACGGTATGTTT GRLNGMF 1

GGGACTTCGCGTCAGTTCCCG GTSRQFP 1

TCGTTTTTTTATATCGCGAGC SFFYIAS 1

GTTTCGTCTGATTTTAGGCGT VSSDFRR 1

CAGGTTGCTCCCGGGTCGTTG QVAPGSL 1

TTCGACGGGTCCGGGGGCTTC FDGSGGF 1

CCGAGCCGTCTTCCGCCTTAT PSRLPPY 1

AGCTTCGGGAGCTCCGATCCT SFGSSDP 1

GCGCTTCGGAGGGACTACGCG ALRRDYA 1

TGCTTGAACTCCACGGCGTTG CLNSTAL 1

CGCCTGAGCCTGGAGATTCCT RLSLEIP 1

GTGCGCACGGCGGCTTGGAAT VRTAAWN 1

CGGTGGCGGGCGACGGTTCAT RWRATVH 1

ACTGAGTTCATCGAGCAGTCG TEFIEQS 1

CGGCATCATAGTGATTGCGCG RHHSDCA 1

GCGCATGCTACGGGCGGGCAG AHATGGQ 1

GGGTCGCGCTCCATCTTGATT GSRSILI 2

TGGCACATCAGGTCGGTGCAG WHIRSVQ 1

GACCCGCTGTGGTCGTTTGCG DPLWSFA 1

CTCCGGCTTTGGGCTTCGGCT LRLWASA 1

CAGACGTGTTGTGCGGGGAAC QTCCAGN 5

TTTGGTGTGAATCACAATAAG FGVNHNK 1

AATATGCAGGACGGCTTTGCG NMQDGFA 1

CATCGGTCCACGAACGGTAGC HRSTNGS 1

TATTTCTGCAGCTCCCGGAGT YFCSSRS 1

TGGTGCGGGGCGCTCGGGCGG WCGALGR 2

GGGTGTTACTTCTACGGTTTC GCYFYGF 1

CTCCGGTGTGCCTGCAATTGC LRCACNC 1

GAGGAGGAGCGTTCGGTTGGT EEERSVG 1

GTTTATAGCATTTATCTGGTG VYSIYLV 1

AGTCTGCGCTGGCCGCCTCCG SLRWPPP 1

GGGCGCTATGATATCGAGTCG GRYDIES 1

GATGGGGTCTCTCCCTGTCAG DGVSPCQ 1

GTCGGGAGCCGCTGCAACGAG VGSRCNE 1

GGGACTGGCTCCAATGTGCGT GTGSNVR 1

CTTCGGGTCGGTCCGTCGTGT LRVGPSC 1

GGCCTTCAGCAGGAGGTTGCT GLQQEVA 1

TGCTACTCGGGCTCGACGGTG CYSGSTV 1

CGTTTGTCCCGGAGTGATCGC RLSRSDR 1

CCGGTTTCCGGCAGCAGCTTC PVSGSSF 1

TACAGCTTGTTGCTCGGGACG YSLLLGT 1

GGTGTGGGCCTGTGGATTGCG GVGLWIA 1

TATCTTGCGTCGGGTGTTCTC YLASGVL 1

GCTGAGTGTTTCAGTTACGCG AECFSYA 1

GTGTCCTGCATCTGCTTCGGG VSCICFG 1

TCCGGTTGTTCGGGCGATGCT SGCSGDA 1

GAGGTGGTTGTGACGGCCGCG EVVVTAA 1

TTTAGTCAGGTGGACGGTGGT FSQVDGG 1

GATACCTACGTGGTTGTCGGT DTYVVVG 1

TTGGCGGTCATCTGCAATAGT LAVICNS 1

TCCGAGGACGTCCCTGCGCGT SEDVPAR 1

GATTGGCCGTCTGTGGATGGT DWPSVDG 1

GTCCATCGTCCGGCCTGTTGT VHRPACC 1

GGCGACGAGAAGCGCTCCGGT GDEKRSG 1

CGCGTGGTGATCGATTCGCCT RVVIDSP 1

AATAAGCCTGGCGTGCTGTCT NKPGVLS 1

TACCGCTGGCCGGTTAGCTTT YRWPVSF 1

CTGCGGGTCGTTGGGCCCCCT LRVVGPP 1

TTGGCCCGCTGGGAGCTCAGT LARWELS 1

TCTTTCAGCTCGTTTTTCTTC SFSSFFF 1

TGTCTGACTTTGTACTTGGGG CLTLYLG 1

GGGGTCAGGAGTGGGGGTGCT GVRSGGA 1

CGGGTTGCCTCGTCCTGTAGT RVASSCS 1

TATGTCGGGTTCTGCTGCTTT YVGFCCF 2

GTTGGGTCCCAGGTGGTTCGG VGSQVVR 1

GCTTCGGGTAGCGATTTTAGT ASGSDFS 3

CTGTGGGTGATTGGGCAGCCG LWVIGQP 1

GCGTGGCTGGCCTCGCGGGTC AWLASRV 1

AGGTATCATTCTTGGGGGGTT RYHSWGV 1

GATGCGCTCCTCTGTATTACG DALLCIT 1

TCGCGGGCGATGTTTGCTGGT SRAMFAG 1

GAGAGTCGGGGCGTGCGCTTG ESRGVRL 1

CTCATGCGTAGCTTCATGGCG LMRSFMA 2

AGGTCTGAGGCCGCGCTGGTT RSEAALV 1

CACTGGGAGATGGACCCTACG HWEMDPT 1

TGTCCGAACACCGTGTGCGTG CPNTVCV 1

TTCTGGCTCGGGGGTTTGTCG FWLGGLS 1

AAGCAGCCGAGGCGCCCTAGG KQPRRPR 1

TTGATCGCGATGGCGTTGGTG LIAMALV 2

GTTTATACGGCGGGGCGCGGG VYTAGRG 1

AATCGCCAGATCATGCGTCGG NRQIMRR 1

CCCGAGCGGGGGCCCCTCGTG PERGPLV 1

GGCGCTGTGAACGTGGTGGGG GAVNVVG 1

CGGCACCAGGACGCGACTACG RHQDATT 1

CTCATTGTCAGGTCCCGCTCT LIVRSRS 1

GGTCGTCTTACGCCGAACTGT GRLTPNC 1

TCGCAGTGGGCTCTCAAGGCT SQWALKA 1

CTCAATCAGCGTTCTACTACG LNQRSTT 1

GTCAGCCGGCGCATGCTTGGT VSRRMLG 1

CCTGTGGCTGTCCAGCGCCTG PVAVQRL 1

GGGGGGGTGAATCGTGCGGTC GGVNRAV 1

TTCACCCGCATGCGTGGCTGT FTRMRGC 1

GACGATGGGTGGTCGGACTAC DDGWSDY 1

TGGACTTTCACTGACATGGCT WTFTDMA 2

GACAATCCCTCCTTGGATCTG DNPSLDL 1

GCGCTCATCAGGGTTGATAGT ALIRVDS 1

AATGCCGCGGCGCGCACGCGT NAAARTR 1

GATTTCCTCTTCTCCCTCTCG DFLFSLS 1

TTCTTGTGCTCTGTTAGCGGG FLCSVSG 1

ACTCCTTGCGGCTCTAATTGT TPCGSNC 1

CTGGGGAGGGGGTCGGGTTTG LGRGSGL 1

GGTATTCCTATCTGGCACAGT GIPIWHS 1

TGTGCGGGGCGCGAGGAGGTT CAGREEV 1

ATCTTGCAGCGGTCGTCCTTG ILQRSSL 1

CGGGGGATCTTGGCCAGGTTT RGILARF 1

TTCGATCCCGGCTACCTGCTG FDPGYLL 1

CTGCGTGAGGAGAAGCAGCTG LREEKQL 1

TTCGGTATTGACTTGACGCCT FGIDLTP 1

GACGGTGTGCGGTATTCTCCT DGVRYSP 1

GTCGTCGTTTCCGGGATTCCT VVVSGIP 1

ACGGTGCACGAGTCCGCCGTG TVHESAV 1

GCCGGCGGCCGGTTTTGTTCT AGGRFCS 1

CACGTGTGGGGCTTGATGTTT HVWGLMF 1

TGTGGTGCGGTCTTGTTTGCT CGAVLFA 1

AACGAGCTCTGTGAGCCCACT NELCEPT 1

GGCGGTCGCGCCTATCGTTAT GGRAYRY 1

GGCCTCTTCTTGCGGCGGCCT GLFLRRP 1

TCTTTCACCAACTGCTCCGGT SFTNCSG 1

ATGTTCCTGGTGGATGTTCTG MFLVDVL 1

GTCGGTGCTAGCGACGCTGTG VGASDAV 1

CGGCGTTTTCAGCTCTTCTCG RRFQLFS 1

TGGAAGGACTGGGTGTCCCAT WKDWVSH 1

CGGGCGCTTCTTGGTACCGCG RALLGTA 1

CATTCGCCTACGGTTAGGTTT HSPTVRF 1

GTGGGCCGGTTTGCGAGGAGT VGRFARS 1

CCGATCATCACGTGCGGCCTG PIITCGL 1

AGTCTGTGGCTTGGGGTGGTG SLWLGVV 1

GTGGAGGTGTCTGGTGGTCCT VEVSGGP 1

GGCGTCAGGAGCTGGTTGGTG GVRSWLV 1

TACGAGTCTGTTGCCTGTGGG YESVACG 2

TTGCGCCAGCCCCGCGGCGCG LRQPRGA 2

CGCAACGACCAGGTCAAGGAT RNDQVKD 1

CGCTCGCACCTGGGCGTCGTT RSHLGVV 1

TGCAGGGTGTTCGTGCCTTAC CRVFVPY 1

CTTTGGAGCGGCACGCGGGGT LWSGTRG 1

CGCCCTGCGGAGCCCCGGTCG RPAEPRS 1

CCGGCTCTCGTGCACGACTGG PALVHDW 1

TTTATTGTCGTGGGCGGCGGT FIVVGGG 2

TGCTACGGCAGGGGGGCGCGT CYGRGAR 1

GAGTATGGGAAGTGGGATCGG EYGKWDR 1

AGTCCTGTTTGCGTCTCTTGC SPVCVSC 1

CGTCGGTATTCCGCGATCCCG RRYSAIP 1

ATCACCAACGCGCTGGATATT ITNALDI 1

TGGGTGGCCGGGTGTCGTGCG WVAGCRA 2

ATGGCTGCGCGCTCGCCGCTT MAARSPL 1

AACCGGCGGATTTGTTGGACT NRRICWT 1

GAGGAGTGTTGGATTGGTGGT EECWIGG 2

GGGAGGTCGAGGACGTCTTCG GRSRTSS 1

TCGGGGTGCTCGGCTTCTCTG SGCSASL 1

TCGCAGTACGGGCTGGGGGTT SQYGLGV 1

GGGCTGATGGCGGCGACTCTT GLMAATL 2

TGTCGGGTCTTGAGGTCCTCG CRVLRSS 1

TCCCTCGAGTCGATGCTGTTT SLESMLF 1

TGCAACCACCGGCCGGTTGGT CNHRPVG 1

GGCTTCCGCGTGCGCTGGGAT GFRVRWD 1

ACGAATCGCGGTGGTTACTTT TNRGGYF 1

GCGGTGGCTTTGGATCGGTGT AVALDRC 2

GTTCGGGTGAACGAGCTTGCG VRVNELA 1

ATCGTTAGGGTGGGGCGCGCG IVRVGRA 1

CGCGGGCCCATGAAGACTCAG RGPMKTQ 1

GGCCCTAGCAGGATGAACCGC GPSRMNR 2

GCTGCGGGGTCCAGGACGGTG AAGSRTV 1

GTGTGGGACATCAGTGACTGC VWDISDC 1

TCCTCGCTCAAGTGCCAGCAG SSLKCQQ 1

TCTGGGTCGGTGCTGTTGCAG SGSVLLQ 1

CGGGCGGCTTGTTTTGCCAGT RAACFAS 1

AGGTATAGGGCTCGCATTAGT RYRARIS 1

CGGTGGGACGGTCAGAGCTGG RWDGQSW 1

GGGCCTAGGAAGGTTGCGTTG GPRKVAL 1

TCCTCGGTGCCCGATAAGCTG SSVPDKL 1

TGGCTTCCTGAGGCTCGTTGT WLPEARC 1

AGCCCGCAGTATTCCTGTGAT SPQYSCD 1

CTTCTGGTCGCCTGCGAGAGT LLVACES 2

ACCTTGGGCTTGGCCCGTGGT TLGLARG 1

CGCCTCCGTCGGGGGGATAGT RLRRGDS 1

TCGCACCGTCGCTGCTGCGTG SHRRCCV 1

GAGCGGCGCTCGGGGTCGGCT ERRSGSA 1

GTGGTCGGCATGAAGGAGGCT VVGMKEA 1

ACGATGCTGCAGGTCCTCGAC TMLQVLD 1

TGGGAGGAGATGCGTAACATT WEEMRNI 1

AAGTTCGTGCGCAGGTTCGCG KFVRRFA 1

TGGACTGCCTCGCATGGCCGT WTASHGR 1

GGTGAGCCGCTCGCTTCGGTT GEPLASV 1

AGTAGTGATTTCCACAGTTCG SSDFHSS 1

TATGACGTTCTCGGTTCCATG YDVLGSM 1

AACTCCACCTCGTTGAGTCCT NSTSLSP 1

GCGTGCGTCCCGGCGGTTACT ACVPAVT 1

GTGGGCCTCGAGGCTGCGACT VGLEAAT 1

GCGCTCAACAACGGGGGGAAG ALNNGGK 1

ACGTACATCGGGGGTCTGGTG TYIGGLV 1

GTGGCGTCTTGGTTCGGTGAG VASWFGE 1

TGTCGTGGTGGTTGTTGCCCT CRGGCCP 1

ACCTCTCCCGTCAAGGGTCCT TSPVKGP 1

TTTGTGCAGTCTCGTACCGTT FVQSRTV 1

AGGGGCTCGAGGGTCGGTCAT RGSRVGH 1

CTGAGGGGTATCCAGACCGGT LRGIQTG 1

AGGAGGGGTAGCTGCCTCAGT RRGSCLS 1

GGCTGTTTGATCGCGGCCTTC GCLIAAF 1

GTTAGCGCTAGCGGTGTGGAT VSASGVD 1

AGCATGCGTAGTGGTCCGCCT SMRSGPP 1

GGTGGGTTTGGCCGGTGGCCG GGFGRWP 1

CACGAGCTCCGCGGGGGTTGT HELRGGC 1

GAGGGCCAGGTTTGCTGGATG EGQVCWM 1

CCGCACCTTTTTTGGTCCCTG PHLFWSL 1

AACCGGTGGAGGATCGACGGT NRWRIDG 1

CGGCGCGGCAAGTCGCTTCAG RRGKSLQ 1

CAGGTGGTCAGTTATGCTGCG QVVSYAA 1

AGCCAGTCCGCGGTGTCGGTT SQSAVSV 2

CCGGGCGTCTCGGTGGCGGGT PGVSVAG 3

ATTGGCTCGATTCGCTCGCGG IGSIRSR 1

TGCTCTCGTGCCGGCTCCGGC CSRAGSG 1

GGCAGCTTCCCCCGCGGGTAT GSFPRGY 1

GTTAACGGGCGGGGCGACTCG VNGRGDS 1

CAGGTTTGCTGTGACGAGGCT QVCCDEA 1

CAGAATCGCGACGGGCGCGGG QNRDGRG 1

TTGATGGTGGCCCTGAATCAT LMVALNH 2

GCTGGGGGCCTCAGGTCCGTT AGGLRSV 1

CATGGCGTTGATAATCTTTCT HGVDNLS 1

CGGGTGCGGGTTACCCTTTGT RVRVTLC 1

TATGGCCCGGGTGGCGGCTGT YGPGGGC 1

TCGGCGGGCTGGCGTCAGAAT SAGWRQN 1

GCTCCCCGGACTGCGCGGCGT APRTARR 1

GTCATCAGGACCTGGAGCGCT VIRTWSA 1

GCCTGCGGGATTAATGGCTGC ACGINGC 1

GATAGTCGGCGGAATCGGGGT DSRRNRG 1

CCGATGTATAGCCAGGGGTGG PMYSQGW 1

CGCTACGAGGGGACCGCGCTT RYEGTAL 1

TCGAGCCATCGGTGTGGTAAT SSHRCGN 1

GGCCGCGGGCACCTGGTCTGG GRGHLVW 1

TGGGGTGACCGTCAGGCTGGT WGDRQAG 1

ATTTTCTGTGGGTTCCACGCG IFCGFHA 1

GGGAATCACGCCAGTACGGCG GNHASTA 1

GTGTTTTCCCCCGGCGATGGT VFSPGDG 1

TTTACTGATCTGACTCTCCTT FTDLTLL 1

CTGGGCTCGGGGGGTGATTTC LGSGGDF 1

GTGGACATCTGTTGCATGCCG VDICCMP 1

GGGTGCTCCACTTTGTCCAGT GCSTLSS 1

TGGCACCCCAGGCGTGCGGTG WHPRRAV 1

GGTTACGCGAGCGCGAAGATT GYASAKI 1

CTCGGCACTCCGGCGCGTGTG LGTPARV 1

TCGTTGCCTCTCGAGGTGCGT SLPLEVR 1

CCTAGGGTTGACACTGACGGT PRVDTDG 1

GGGTGCCACAACCGGTCCCAG GCHNRSQ 1

CTCTGGGAGGGCGTCGACGTT LWEGVDV 1

CATCTTTATGATCTGCCGAGT HLYDLPS 1

TGGCGCTGGCCTGGTTGTTAT WRWPGCY 1

CACTTCATCAGTTTCGTGTGT HFISFVC 1

CTGCCGGTTGGTCATTCGTAT LPVGHSY 1

TGCGTTTGTGGGCGCCCGCGG CVCGRPR 1

AGGGGTGACCGGGGCAATAGG RGDRGNR 1

TATGGCCGCCGCTTGTTGGGG YGRRLLG 2

AGGCATTCGGAGGCGGATGGC RHSEADG 2

GGGTTGGTGTGGAGCCTCAAC GLVWSLN 1

GCGTGCCTCCGTTGGTGGCCC ACLRWWP 1

GCGTGGAGCAAGCATTGCGGT AWSKHCG 1

CGTGGTGCTGCGGTCTTGGTG RGAAVLV 1

GCCGGGTTCGGGTCTGATACT AGFGSDT 1

GGTGGGGACTGGAGCATCTGG GGDWSIW 1

TCCTATGCTCCCGGCAGTAGT SYAPGSS 1

TGGCACGCCTCGCGCAGGGTT WHASRRV 1

TGTGGTATGCCGAGCCTGCAT CGMPSLH 2

TGTCGTGTTCTCAGCTTGTGT CRVLSLC 1

GGGATCGTCGAGTGCTATCAG GIVECYQ 1

CGTGCGTGGAGCAGCTGCATG RAWSSCM 1

CACTTCAGCGCGGGTCTGCGC HFSAGLR 1

ATTGGTGTTTTTATGTGTATG IGVFMCM 1

GGGGCTCTCGATGGGGCGGCG GALDGAA 1

TTCCAGGCGGGCTTCGCCGGG FQAGFAG 2

TTCACTCCGTATACCGTGCCT FTPYTVP 1

GGTTACTGCCATGCGGAGGCT GYCHAEA 1

CTGGCGGCGTGGCGGCATCCG LAAWRHP 1

GTGGCGGCCCTCCTTGAGTCG VAALLES 1

CGTGCTCGGGACTCGGGTGGG RARDSGG 1

TTCGTGACGCCCAGTCGGATG FVTPSRM 1

GAGAGTTTTGCTTGGCGGGGG ESFAWRG 1

GGGCTCGTTGACTGCACCGTT GLVDCTV 1

GCGAGGGAGGATCTTGTGGGG AREDLVG 1

CAGGGGAAGACGGGCCTTGAG QGKTGLE 1

CACTGGTCGAGCTACTTTCCT HWSSYFP 1

CACCGTCTGAGTAACTCGCGT HRLSNSR 1

GGGGCCGTGCGCCTCTTCAAC GAVRLFN 2

AGGGCGGGTGTCGTGCTTAAC RAGVVLN 1

GTGTCTTCTCTCGATGGCGGC VSSLDGG 1

CGTGCTGTCGCTTGCGGCCAG RAVACGQ 2

CCGGAGGTGCACGATGCCCTG PEVHDAL 1

CTCTCTCATAGGCCGAGCCTT LSHRPSL 1

TTTATGGGTGGGATCTACCGG FMGGIYR 1

CGTGGCGGGCTGGGCGCTACT RGGLGAT 1

CGGGATGCCCGGCTTGTGCGG RDARLVR 1

TTGTTGACGATCACTTGGGGG LLTITWG 1

ATTAGGGATAGTACGTGGGTT IRDSTWV 1

TCGGGCCGTATTGACGGCGCT SGRIDGA 1

TGGCAGGGCGGGTTGGCGTCT WQGGLAS 1

GGTAGGGGGGAGTTGTTGACC GRGELLT 1

GAGCTCGGTGCCACTCTCTAT ELGATLY 1

GGGGTGCCTTGCATGCCTAGG GVPCMPR 1

AAGTGTCGTGGGTGCATGTTG KCRGCML 1

ACTGTGCGCGATTCGAAGTCT TVRDSKS 1

AATCTTGGCACTGTGGTCCGC NLGTVVR 1

CCCTTCGCGGAGGACAGCGTT PFAEDSV 1

AGCGCTAGGGTGAGGTGTCTT SARVRCL 1

TTTTCCGTCAGTTGCATGCGG FSVSCMR 1

GCGATGGGCGACATTTACCGG AMGDIYR 1

GTCCGGATGAGTAGGTGTGGG VRMSRCG 1

CTGGTGGTCACGCTGTTGAGG LVVTLLR 1

CTTTGGTTGGTGTGTGGGCGT LWLVCGR 1

AGTTCCAGTGTCTTCGCTACG SSSVFAT 1

CAGGCTGTGTGTGGCAGGTCT QAVCGRS 1

CTGGTTCTGGACCACGTGCCC LVLDHVP 1

CGCCACTGTGTCGATTGCTGG RHCVDCW 1

CATGTGTTCGGTCGCGAGGAT HVFGRED 2

TATGTTGGTTTGGACTGTATT YVGLDCI 1

TTTTGTCGGGGTCACGCGAAG FCRGHAK 1

GTTGGCATGTTGTGGTCTCTT VGMLWSL 1

AGGTTCGCGGTGACCTATAAC RFAVTYN 1

GATGGGATGAGCTTTTATGGG DGMSFYG 1

GGGTTTCTTGCGGCCGAGGCT GFLAAEA 1

CTTGGCCATAGCATCCGGAGG LGHSIRR 1

GATGGCGTGAGCTCCCGCAGT DGVSSRS 1

TGTAATAGCGGCGCTACTATG CNSGATM 1

GTCGCGCGCCGGGGTTGCCCT VARRGCP 1

TGCGCCGACCGGTTTAGTATC CADRFSI 1

ATGGTCTCGGTTAGGGTCTCT MVSVRVS 2

CCTGGTCAGGGGGGCTGCTCT PGQGGCS 1

CGCTCTTTCATTGCTGATAGT RSFIADS 1

ACTGCGGGGAGCTGTAAGGTT TAGSCKV 1

ATCCTGATTTACGTCCGGTTG ILIYVRL 1

CGCGGGGCTTGCGTCACTGGT RGACVTG 1

AAGGGGGTTCTCCGCGAGCAT KGVLREH 1

GTCCGCGGGACTGAGGGTGCG VRGTEGA 1

TTGTGCTGCAGCTCGCGGTCG LCCSSRS 1

ATGTATTGCTTCGGCCTGGTG MYCFGLV 2

TCCTGTCAGGTCGTCGACGGC SCQVVDG 1

TGTCTTTGGTGTCTGAGTAAG CLWCLSK 1

GCCGATTACAGTATTCGTTGT ADYSIRC 2

CGGAGTTGGTTGATCAGTGTT RSWLISV 1

AGGCGTCACTCCTCCTTGGTG RRHSSLV 1

CGTCCTAAGAGGAACCTCGCG RPKRNLA 1

TGGCTTGCCAGCCACCGTTGG WLASHRW 1

GTGGCCCGCGCGCCTCGTGCT VARAPRA 1

TCGGGCATCTTTAAGGCCCAT SGIFKAH 1

GTGTCCTGCAGGGTCTCTCGC VSCRVSR 1

GGTGGCGTGTCGATGTTTATG GGVSMFM 1

TCTAGTTTCAGGCTGGATGCT SSFRLDA 1

CGCGTGGCTTGGGTGCAGCTC RVAWVQL 1

CACACTACGTTTATCTCTATT HTTFISI 1

TACGGGGTTGGTGGGACCTGG YGVGGTW 1

TTCGATCAGCGGTTGCGGTGT FDQRLRC 1

GTCTTGCGTTTCTGTTGTAAG VLRFCCK 1

GGTAGGCTGGGGCTCGGTTAT GRLGLGY 1

GAGCACTTCTGGCGCTGTGTG EHFWRCV 1

GGGGGCTGGGTTTGCTCGCGT GGWVCSR 1

AGGATTGCCGGTTGCCATAGG RIAGCHR 1

TCGCGCCGGTCGATGTGCGGG SRRSMCG 1

GTCGTCCTGCCTGTCAGTCCC VVLPVSP 1

GTGGCTAAGCTGAATCGCAAG VAKLNRK 1

GGCGGGCTGGGTCGGACGCCT GGLGRTP 1

GATAGGTCGTGGTTCTGCTTT DRSWFCF 1

CTTCAGGTGTGCTGTTCGCTC LQVCCSL 1

GTGCTGCGGAGGACTAAGGTT VLRRTKV 1

TTGTGGCTGGGCAGCCTGACT LWLGSLT 1

TCCAATTTGGGCGGGTTGCTT SNLGGLL 1

GGGCATTGTTATAATCATAGG GHCYNHR 1

TCCCTGAAGTGCCAGGTCGAG SLKCQVE 1

CACGTCGTTTTTTTCCTTTCT HVVFFLS 1

AACGGTCCGGGCCGTAGCCCT NGPGRSP 1

TATGCCGTCAGTGCGCACTGT YAVSAHC 1

TTTTGCCTTGTCTACCCTACG FCLVYPT 1

CCCAGCCTCGGTTGGTGGGGT PSLGWWG 1

TGGAGCCAGGAGTTCAGGTTC WSQEFRF 1

CTTTTCACGATCAGGTCTCGT LFTIRSR 1

GGGTTGAACCCCTGCGTGTGT GLNPCVC 1

GGTCGTTGGGGGGCGGTGTGG GRWGAVW 1

CACCTGTACAGGCAGAAGCTT HLYRQKL 3

GGCAGCCGGCCTCGGGCTGCT GSRPRAA 1

GTTTGCACCGGGATCATCACG VCTGIIT 1

GGCGGGAGCAGCGGCCGCGCT GGSSGRA 1

AATGTCCATAGTCTCGGCGCT NVHSLGA 1

AGCCGTTTCCCCCGCGCCTTT SRFPRAF 1

AAGTTTTGGAGCGAGGCCCGT KFWSEAR 1

GGGCTCTATAAGGTCTGGACT GLYKVWT 1

TTGGTTTTTGCTTGCGTGGTG LVFACVV 1

ACTGGGGGCTATGGGTGGATC TGGYGWI 1

CGGACGCGCGTTACGAAGTAT RTRVTKY 2

AAGTTGACCTGGCTTCGCAAT KLTWLRN 1

CTTAGTGTGCCTATGACGCTG LSVPMTL 1

GAGTCTTACCAGCCCGTCTGT ESYQPVC 1

GATCTTTGCAGGTGCAATCCG DLCRCNP 1

ATGTATGGGGGGACTTGCTTT MYGGTCF 1

CGGGGCTTGGGTCGGGTGAGT RGLGRVS 1

GGTGTCAACGGCGTTGCTTGC GVNGVAC 1

GCGCAGAGTATTACCGTGCGC AQSITVR 1

GGCATGCACGACAGTGGTAAG GMHDSGK 1

TCTTACTCTCGCGTGCTGCGT SYSRVLR 1

AGTTTTTCGAGCGCTAAGTGG SFSSAKW 1

TACCAGGATTGGCTTGACGTT YQDWLDV 2

TCGCAGACTTGGCGGGCCATT SQTWRAI 1

TGCAATAGGAGCGTCGTGTGT CNRSVVC 1

GCCGCGGGGAGGTTCTGTATT AAGRFCI 1

CGCTGGGTGAACTCGAAGTGG RWVNSKW 1

GTTCGGCGTACGTTTGAGTTG VRRTFEL 3

TTCGTTTCTTGGCGGGGCCGT FVSWRGR 1

AGCAGGGGGAAGCATGCTCGT SRGKHAR 1

GGGATCCCTTGGCCTCGTTGG GIPWPRW 2

GTGCTGCTTGGTTCCTTGGGG VLLGSLG 2

ATTTTCTCGTACATGCAGTGC IFSYMQC 1

GGGTTCATGGATAGGAGGTCT GFMDRRS 1

CCGGCCCATTCTGCCCAGCCT PAHSAQP 1

GGGGCGGGCTGTCGTTGGCGG GAGCRWR 1

CGTAGCTCCCCGGGGGTCCAT RSSPGVH 1

CTCGGGAGCGGCTGTGCTGAT LGSGCAD 1

CATTGGGTGAGCCTTGGCCAG HWVSLGQ 1

TCGGCGCACAGGCTGGAGTTT SAHRLEF 1

CGGGGCGTTCATGTCTGGTCG RGVHVWS 1

TTGGGTGATGTTTCGACTTCG LGDVSTS 1

GCGATTCAGTTCTGTCATCTG AIQFCHL 1

ATTGATTCTAGGGATCCTGTT IDSRDPV 1

CTGGTGAATATCGGTGGTGAG LVNIGGE 1

TTGGTGTCGGTGCCTTCGAGG LVSVPSR 1

TCCCGTGCGCACTTCGTTGAT SRAHFVD 1

CTCCTGATTACGCGCTGTCTT LLITRCL 1

TGTATTTGGGATGCTGAGCGG CIWDAER 1

ACGGTGTGGCCGCAGATCAGT TVWPQIS 1

CTGTATTGTGGTGGCGATGGG LYCGGDG 1

TACTGCTTCGCGTCCGCGCAT YCFASAH 1

ATGTTTATTTCTGCCTGCTGG MFISACW 1

ACTTGCTGCTTGCCGGTCGGG TCCLPVG 1

CTTTACCGTCGCTGGATGGGG LYRRWMG 1

GTTCACGCCACTTGCTCTTCT VHATCSS 1

TTGTGGCGGATTCACGCTTTC LWRIHAF 1

GCCGGGGAGGGGCTCTGTACG AGEGLCT 1

GGTCCGAGGAACAGGGCCAGT GPRNRAS 1

GGCGGTCGGACGTACGTGACT GGRTYVT 1

TGTCTTCAGTGGCCTTGTCGC CLQWPCR 1

GGGAGGCCGTGGAACGGGGCG GRPWNGA 1

TTGGTGTCCGAGCTTAGGCCG LVSELRP 1

CATTACCGCTGCTTGCTTTGT HYRCLLC 1

GATGGCTACGCTCTGCCCGTG DGYALPV 1

CACCAGAAGCGGGTTAGGTGG HQKRVRW 1

TGTCACTTGCGCTGGCTTGCT CHLRWLA 1

TCTTGGCCCAGTTGCAATGAG SWPSCNE 1

TCTCGCAAGCCCGACTTCTCC SRKPDFS 2

TGGTTCCTGGAGGCCGGGTGT WFLEAGC 1

GTGAATGCTCGCGTGAGGAAC VNARVRN 1

GCTGAGCGGCGCGCTTTGCCT AERRALP 1

CTTGACTGTGAGGGTTATGTG LDCEGYV 1

GCCGTGTGGCGCAGCATGTCC AVWRSMS 1

GCGCGGGGCGCGCGGGTTCGT ARGARVR 1

GCGCAGTCTGCGATGCGCTGC AQSAMRC 1

GTTCTGGGCGGGAGCAGGGTT VLGGSRV 1

TCTGACGACAGTTTTCGGGCT SDDSFRA 1

GGGAGGATGGCGCGGGTCTCG GRMARVS 1

GGCTGGGCCATCTTTATGGCG GWAIFMA 2

TTTGTGCGCAGCTCGGTTGCG FVRSSVA 1

TGTGGGCCGTTCACGGTTAGT CGPFTVS 1

CCTAGGTGTGCGCAGTGTGGT PRCAQCG 1

TGTGGGCCTTGTACGGAGCCT CGPCTEP 1

TCTATTCGGCGTGTCGTGGGG SIRRVVG 1

TCTCTGTTCGAGCTTTCTACC SLFELST 1

GACGACCGCATTACGTATGTT DDRITYV 1

TCGCTTCGCGGTTGTATCTCG SLRGCIS 2

TTGTCGGGCAGGGAGCGTCCG LSGRERP 1

ACCGCCCACGTGCTCAGGCCG TAHVLRP 1

TTCAGGCACTGTCCTTGCCGC FRHCPCR 1

GTGAGCCGCGATGTGTGCGCG VSRDVCA 1

GCGATCATTGGGTGTGAGTCT AIIGCES 1

GCGGGGAGCGGGCCTCCTCGT AGSGPPR 1

ATCGTGTGTCAGGCGATGGCT IVCQAMA 1

GACAGCCGGATCCTCGGGTCG DSRILGS 2

TTTGCCATGAATTCTCGGTGT FAMNSRC 2

CCTTTTGACTGTAGCGCGGTT PFDCSAV 1

TCTGCGGCTAACTGCTGGGCT SAANCWA 1

CGTCGTCCCGTTGCGCCTTGG RRPVAPW 1

AGGGCCGGCTGCTTGATCGTT RAGCLIV 1

AGCGGGGGGACTATCTGGCTG SGGTIWL 1

GGCTGCGTCGTGTGGGTGCAT GCVVWVH 1

CCGGGCCGCGAGGCCGTGGCG PGREAVA 1

TATATGGCTTATGGCGGCGAG YMAYGGE 1

CATCGTGTCAGCCCTTGTCCG HRVSPCP 1

GCTTCGGTGACGGGCAGGTCT ASVTGRS 1

ATCATCGCCACTGTTCCTTCT IIATVPS 1

CATGGCGGCGGGTGGCTCGGG HGGGWLG 1

CTGTCTTCCGCGGACGGGCGG LSSADGR 1

GCGCTGCACTATAATGCTTGG ALHYNAW 1

GCGGTGGTTTACATGCTCGGG AVVYMLG 1

GGCTCTGGTATTAACTCTACG GSGINST 1

GAGCGGGAGCTGGGGTGGTTC ERELGWF 1

AGTGTCCGTAACACTGGGGAG SVRNTGE 1

TGTGATTTCTTGATGTTGTCG CDFLMLS 1

CTCCTCACGAGTGACGTGCGT LLTSDVR 1

TCGTGTTCGTTCCGCACTAGT SCSFRTS 2

AGGGCCGGGCTGAGCGTTCCT RAGLSVP 1

GTCTTCTTGGGGGTTTTGTGG VFLGVLW 1

CGCTGCGCCAGTTATACGCGT RCASYTR 1

TTGTTCGCGACTGCCCGTGCG LFATARA 1

CTGGGCTCGGTTAATGTTATC LGSVNVI 1

ACGTACCAGAGTGGGCGCCGT TYQSGRR 1

CCCGCGTCCGTGGGGCGGATT PASVGRI 1

AGCAGTTTTCCGAGTTTCCCT SSFPSFP 1

TACAAGTGCCTGACTGACCGT YKCLTDR 1

GTTTGTACCGCCCTCCATCTC VCTALHL 1

TTCGAGGACTTCAGGGAGGTG FEDFREV 1

TGTCTTCGCTGGGGCTGCCTT CLRWGCL 1

GGCCCGCTGACGGGCGACATG GPLTGDM 1

TGCGGGCTCGCGCGTATCGAC CGLARID 1

GCGCATTTGAGGGCTCGCGGT AHLRARG 1

CTCACGGCGGCTCGTGCTGTT LTAARAV 1

ACGATTGGGGCGAGGTCGTCC TIGARSS 1

CGCTTTACCGGGTGCAGTCAT RFTGCSH 1

GTCGATCGCGGGGTGCGTGGG VDRGVRG 1

GGTAAGCACAGTTGCACCTCC GKHSCTS 1

TTCAGTTTGGTGCAGTGTGGC FSLVQCG 1

CAGGTTTGCGGTAAGTTTACG QVCGKFT 1

ACCGCCCTCAGGTTGGGTGTG TALRLGV 1

GTTGTCCTGATTAAGGGTGGG VVLIKGG 1

CGTATGAGTGATCTCGGGGGG RMSDLGG 2

TGCAGCGCGCTGGTTGTGCGC CSALVVR 1

CTGATTCTCTGGCACGCGCAT LILWHAH 1

GGGTGTCTGGGCTGCCTTTTG GCLGCLL 1

CCGCGCGCGCACAGCTGTAGG PRAHSCR 1

GGCGTGTGTGGGCAGAGGATG GVCGQRM 1

ACGTGCGGCGCGTGCGGGCGG TCGACGR 1

TCCGAGGGCGTCGTTCTTTTG SEGVVLL 1

CCGCAGTTCGAGCTTAGGGTG PQFELRV 2

GTTGCTAGTTGCGCTAGGCGG VASCARR 1

GGCCTCGTGGCGGGGCATCGG GLVAGHR 1

CATGATCGGCTGGCCGGTGTT HDRLAGV 1

TGCTCCGTTGCCCACTTGGAC CSVAHLD 1

CAGGTGTTTAGCTACTTTGTG QVFSYFV 1

AGTCGGCTTCTCAGCCGTGGT SRLLSRG 1

CGTGACCCCCGCTTCAAGACG RDPRFKT 1

GTGGACGGGTGCGCGCTCGGC VDGCALG 1

CAGGCGCGGAGCAGGAGGATT QARSRRI 1

CTCCGCCGGATGTCCACGTGG LRRMSTW 1

TTCTGGCTGACTTCGCAGGAG FWLTSQE 1

TCTACCCACAGGCGGGACACG STHRRDT 1

GCTGATCTTGTTCATGCGTCT ADLVHAS 1

AGGAGCTGGAGGGCCGTCCCG RSWRAVP 1

GTGTCGTTTTACCCGGGTACT VSFYPGT 2

ATGCGGAATGTTGTGCCCCCG MRNVVPP 1

AGCTGTCGGGCGCAGTGTGCG SCRAQCA 1

TTTCACGTTTGGGATTGGGAT FHVWDWD 1

TTGCCCGGGAGGTGCGGTTCT LPGRCGS 1

CTGAGGGTCCTTGGTCCTCTG LRVLGPL 1

GGGGCCGGCGTCTCTAGCAAT GAGVSSN 1

TGCCATCTGTGGGGTTTTCAG CHLWGFQ 1

TGCGGTTCGAGGCTCGCCTTG CGSRLAL 1

GCGGGGATTGAGGCTAGTGTT AGIEASV 1

CAGTGGTTGAGGGTCAGTTGC QWLRVSC 1

GGGAATCGCTGGGTCGGTGTT GNRWVGV 1

GAGCGTGATGCTGTGGACGTT ERDAVDV 1

TTCGGGTTCCCGTTGCGGTTT FGFPLRF 1

TGGTCTAGTGGCTTCAGGTGG WSSGFRW 1

GTGGCTGACAGCGGGGTGACC VADSGVT 1

CCCGGGAGCTTGATGGTTACG PGSLMVT 2

TCCCGGTGTGCTGGCCCCAAG SRCAGPK 1

TCCAGTGAGAGGTCGGTTTTT SSERSVF 1

TCTGGGCGCCAGCGGATTCTT SGRQRIL 1

TGCTTTCTGTGCGGCGGGCGG CFLCGGR 1

ACCTATCGCACCGGTGAGTAT TYRTGEY 1

AGGCTTGAGCGTATGATGCAG RLERMMQ 1

ACTGGCTTCAGCGGGCTGGTG TGFSGLV 1

AATTTCGTCGCGATGCTCGCT NFVAMLA 1

CGCGTGGTCCGCCATCGTCTC RVVRHRL 1

CGGGCGCTCGAGACTGGTTCT RALETGS 1

AGGAGGATGACCGGTTGGGCT RRMTGWA 1

GTCCCGGGGGACAGGCTCTTG VPGDRLL 1

GGGTATATCCAGGGCGCGGGG GYIQGAG 1

GCTGGTCGCAGCCGCCGGCTT AGRSRRL 1

GGGAGGCAGGCGGCGCGCTTT GRQAARF 1

GTGGAGTTCTGGCACTCGCCG VEFWHSP 1

CGGCGGTGTGCTGGCTGCGCT RRCAGCA 1

CAGACGCGGAAGGGCGTCCGT QTRKGVR 1

GACCGGAGGGTGTATGATGTT DRRVYDV 1

AGGCGGGTGATGACGAGTACT RRVMTST 1

AGCATCTGGGCCGTCCATGAC SIWAVHD 1

TTCTGGTTTTTCGTGGGGCGT FWFFVGR 1

GCGAAGGTGTCCAACGCCGGT AKVSNAG 1

GATGAGCCGTCGTGGGTCCAG DEPSWVQ 1

GGGTGGGACATCCTTCATTCG GWDILHS 1

TGGGTCGAGGTCGTTATGGGC WVEVVMG 2

GCCGAGCCCAGCTTCTCCTTT AEPSFSF 1

CACGTTACTAGCAGGGGCATT HVTSRGI 1

TGGGCCGTGGGCGGCGTCGTT WAVGGVV 1

GATTCGTCCGCTGTTGAGCCT DSSAVEP 1

GATGGCTACGACGGGGTGGGG DGYDGVG 1

TCCAACTGCATGGGCCGCGGT SNCMGRG 1

GGCTTGGCGTGGCCGTGTGCG GLAWPCA 1

TGTGATGGCCTGTGGCAGTCT CDGLWQS 2

GATAGGGTCATTGGGGGCTCT DRVIGGS 1

TGCACGCAGAGCTGCGGGGGT CTQSCGG 1

ATGGGCTTGAGGGGCCTTCGG MGLRGLR 1

TCCGGTTCGCCGTGGCGGTTT SGSPWRF 2

TTCTCCTGGAAGAAGCGGTTT FSWKKRF 1

GGGCAGGTGTGCACTCTGGTC GQVCTLV 1

TTGGGTGTCACGCGTACTTCG LGVTRTS 1

AAGTCGGCGCCTTGTCATCCG KSAPCHP 1

TGGCTTATCTGTGGCCGGCGT WLICGRR 1

TCGCGGTCCACGGGCGCTTAC SRSTGAY 1

ATTTGTCTGTGTCCGCGCGTG ICLCPRV 1

CTGTGGTCCTGGTCCGCTCCT LWSWSAP 1

ACGTGTAGCAAGCGTAGTCGG TCSKRSR 1

AGGGAGAATCTGTTCTGCGTG RENLFCV 1

CTCGCTTGCAGCTTTAGCCGG LACSFSR 1

TTGGAGCGCGGCAGCGTCTGG LERGSVW 1

ACCTCGTCTGGGATGCGGGAG TSSGMRE 1

TGCATCTATAGTGGGAAGTCG CIYSGKS 1

AACAGGCTCGGTCGCGGGCGG NRLGRGR 1

TGCGGGCCGGTGGGGGAGCGG CGPVGER 2

GGTTGGGCCCCCTCGAATGTG GWAPSNV 1

GGTTGGGTTAGGAGTGCGGTT GWVRSAV 2

TGCCGCTTCCCCCGGTGTGGG CRFPRCG 1

CTTGGTTTCACGGTTGAGAGC LGFTVES 1

CTTAGCCCTGGTTCTCCCGAC LSPGSPD 1

GACTGCGCGAGTTGCGCCCTT DCASCAL 1

CAGTTCGAGTCCTGCGTGACT QFESCVT 1

CGGGGTGCCCGTCGGCACGTG RGARRHV 1

TTCGGCTGCCTCCGCTTGAGT FGCLRLS 1

ACCGGTGGTCCCATGGCGCTG TGGPMAL 2

TTGTTGGCCACGGGCTCGGAT LLATGSD 1

TTCCGTGCGAGGCGCTTGTTT FRARRLF 1

CGTCGGCTGCTCATGGTCTTT RRLLMVF 1

GGGGCGCCTGTCTGGGCCAAG GAPVWAK 1

GACTTGGGGCGTTGGCTCCTG DLGRWLL 1

GCCAAGAGGCGGGAGGACGTG AKRREDV 1

CTCCATCGTCCTCGGCGGCCT LHRPRRP 1

GGGTTTTTCGTGTGCTGCCGT GFFVCCR 1

ATCATCCCCGGGGTTGCTGCT IIPGVAA 1

CGGCGTCCGGATGTGTTGCGT RRPDVLR 2

TTCCAGCTGTTGCTGGTGGCG FQLLLVA 1

TTGATCCCGCCTGCTTATGGT LIPPAYG 1

CAGGGGGCTCGGCTGGTGTCG QGARLVS 3

TGGTTTGGGATTAGCACCCGG WFGISTR 1

GCGGGTGTGACCCGCGGCCTT AGVTRGL 1

GTCTTGTTCGCCGCGGCTGAC VLFAAAD 1

CCTGGTTCTAGTTGTGTGCTG PGSSCVL 1

ATGGGTCGGGGCGTGCTGAGT MGRGVLS 1

GGCAGCGCGGTGGGGGCGCTG GSAVGAL 1

TTCGTTCTCTCTGAGTGGCAT FVLSEWH 1

GATAGGGACTTGAGGGGTGAG DRDLRGE 1

TGCTGGTGCCTCACTAGCCAT CWCLTSH 2

CCGGTTCTGATTGTGATTCGC PVLIVIR 1

GTGGAGCCTTGGTGGATCGAT VEPWWID 2

TCTCACTACGTGACTTGCGTG SHYVTCV 1

TGGGCCCTTGGTCAGTGCTCG WALGQCS 1

GCGGGGCGGTTCTACGGGGCT AGRFYGA 1

GCCATGCTCAGTTATGGGAGC AMLSYGS 1

GATCAGTCGATGTGCGTTCCG DQSMCVP 1

GCGGTCAGTTGGTGGCGTCAG AVSWWRQ 1

CCCCTTGAGGACGTCTTTCTG PLEDVFL 1

GGGAGGAGCTCGGCGGCGCTT GRSSAAL 1

CTTGAGGAGCTCTCTGGCTGG LEELSGW 1

TCCCTGCGCGGGGCCGTCTCG SLRGAVS 1

TTGTTGATTACGTATCGGTGT LLITYRC 1

CGGCAGCGTACTGGCATTCCT RQRTGIP 1

GTCCTCCAGCTCTGGAGTCTG VLQLWSL 1

TACGGTGAGGTGCGGGGTTCC YGEVRGS 1

GGTTCGCTTAAGTCGTTCGAG GSLKSFE 1

CGTTTCTGGATTATGTATTAC RFWIMYY 1

ACGCGGCAGCGGAATTCGCTT TRQRNSL 2

GTGGTTGCTCTTCTCTCTTGT VVALLSC 1

CAGCAGTTTAGCGCTGGGAGT QQFSAGS 1

GGCAGTTCCTGCTTGCGCAGC GSSCLRS 1

GTCGGTATGACTGAGAGTTGG VGMTESW 2

CCGGCGGGTCCCTTGGCGCAG PAGPLAQ 1

TACCCGCGCTCGAGGCTGCCG YPRSRLP 1

ATTATGTCCATGCACATGCAT IMSMHMH 1

GGGTTTTCCTCGAGGTCCTTC GFSSRSF 1

AGGGTCTCCGACGTGTGTCTT RVSDVCL 1

GTCGCTTCTACTGCCTGGCCG VASTAWP 1

TACGATCGGGTGCACGGCCGT YDRVHGR 1

GTGTTCTCCTTCGATATTGTT VFSFDIV 1

CCCGTTACTTCGGTCGCCGGT PVTSVAG 1

TCTTTTCACTTGGTCTCGGTC SFHLVSV 1

ATTCGGTCGATGGTGGCTGAG IRSMVAE 3

AGGGCGGATCTCGTCCGTCCG RADLVRP 1

GGGTTTCATCGGATGTGTCGG GFHRMCR 1

GGCGTGGACAGCCGCAAGCCT GVDSRKP 1

TGGGGTAACTGCGGTCATCCT WGNCGHP 1

ACCGCGCGTGCTGGGCTTCGT TARAGLR 2

CAGTTGCGCACGGCGTTGCCT QLRTALP 1

GCGAGGAATATCTGCGATGGT ARNICDG 1

AGGGTGGAGAGCTTCTACGCT RVESFYA 1

CGTGTTCTTCTGCGGACGCGT RVLLRTR 1

TTGGTCCCCGCGCCGATTGTT LVPAPIV 1

ATGGAGAGGGGGTCCTTGGGG MERGSLG 1

AGGTATTGGGGGTGGGCGATG RYWGWAM 2

CGCTACCCGGTGAGCCGGTTC RYPVSRF 1

GCCGTCCTGAACAGCAGGTGT AVLNSRC 1

GTTGTGGTCGACGCGAGCCGT VVVDASR 1

GTCGCCAGGTGCTCCGGCTCT VARCSGS 1

CATGGCGTCTATGCGTGCAAC HGVYACN 1

CTTATGGGCAGCCTGTTGTTG LMGSLLL 1

GCGGCCCAGCATAGCTGTCGC AAQHSCR 1

TGTTGTCTGGTGTTGGTGTGC CCLVLVC 1

AGTGTGCGCGAGCTGGAGCTT SVRELEL 4

TTCCTGGTGCGGTGCTGTTCT FLVRCCS 1

TTTCGCGGTTGTAGTGGGTGC FRGCSGC 2

CTGCGGACGAGGTGGAGTCGG LRTRWSR 1

GGTGTTCGTAGGTGGAGGTAT GVRRWRY 1

ATCTGGGGGGCGTTGTGCTTT IWGALCF 1

GTTGAGGGTCGTTGTTCGGTG VEGRCSV 1

GTCGGGCCCATGGAGGTCGTT VGPMEVV 1

GGCCCTCTCTTGGACCAGTGT GPLLDQC 1

AATTCTGGCGGCAGGGAGTTT NSGGREF 1

AAGTATTTGTCGTTCGTGCAT KYLSFVH 2

CCTGTGTGTACTCCGTCCACT PVCTPST 1

GTTTTCGGCGCTTTTGGCGTT VFGAFGV 1

GGCCGGGGGAGTGTTGCGCTT GRGSVAL 1

GTGCCGAGGTGCGTGAGCGGG VPRCVSG 1

TCGTGGGGTAGGGTGGCTCCT SWGRVAP 1

CAGCGCCACAGCCTGTTTAGT QRHSLFS 1

CGGCGTGCTTCGTGGCAGAAT RRASWQN 1

GGTAGGAGTACGCTCGTGCTT GRSTLVL 1

ATTGTTGGGGAGCGCGAGCCC IVGEREP 2

TGCGGTGCGTTCAACCATGGT CGAFNHG 1

GAGGGCTGTTCTCGGATTTCT EGCSRIS 1

GCCCCTCCCAAGTGGCGTACT APPKWRT 1

CTTGTTAAGACCGGGTGCGGT LVKTGCG 1

TGTCGGGGCCACTTCTTGTGT CRGHFLC 1

TGTGCGCCGGTGCAGTTGTCT CAPVQLS 2

GTGGGCCGCACGGCCGGGCGC VGRTAGR 1

TGGGAGGGCAAGGGTAGGTGG WEGKGRW 1

TGCCGTGAGGCGCGGCGCCGT CREARRR 1

CGGTATACTAGTGGGATGCAG RYTSGMQ 1

TTTGAGGCTGCGAGGAGTCCG FEAARSP 1

GCCAACCACTCTAGCAGTGAG ANHSSSE 1

TGCCCCTCGAGCAGGGGCTGC CPSSRGC 1

TTCGGCCCGCGCGCGTGGTCT FGPRAWS 1

CCCGACCTTTCTACCATGCCG PDLSTMP 1

CTTGTGCATCGCTGGGGGGTT LVHRWGV 1

ACTGTGCAGCTCGGGATTACT TVQLGIT 1

ATCGATCCGTCCAGCGCTTTC IDPSSAF 1

GGGGCGCGCTACGGCCGTTTG GARYGRL 1

TTTGTGCCGATTCCGCCGCTT FVPIPPL 1

TCGGTTAATAGGTTGTCTGCG SVNRLSA 1

GCGCAGTCGTTCCTCGGCAGT AQSFLGS 1

TGGTACTTGACCAGTAACGTT WYLTSNV 1

CCTGTGCGGCTCGGGGCGGCT PVRLGAA 1

GTTGGTGGCGGCTGGGGTCCT VGGGWGP 1

TTTTGGGAGATCAGTTATATT FWEISYI 1

ATGTGCTCCGTCTTCGGCGAG MCSVFGE 1

TCCACTAACCTTTCGTCGGTG STNLSSV 1

CGGCCGACTTATTATTCGGTT RPTYYSV 2

GTTCTGGTTAGCACGGGCACG VLVSTGT 1

AGCAGCGTTGTCTATCGCCAT SSVVYRH 1

GGGGCGCTGGTTGTCGTGGGG GALVVVG 1

CGGGCGCGTCTCAACCCCATT RARLNPI 1

TACGGGGCTATCAGTCTCAGT YGAISLS 1

ATTCTGCACCAGATGCATGGT ILHQMHG 1

GAGGGTCCGTTCATCCCGAAG EGPFIPK 1

TACGGTCCGACCGACAGCGGT YGPTDSG 1

GTCGGCGTGATTGCCGGTCTT VGVIAGL 1

AGGCGGACGGTGGGGGCTCTT RRTVGAL 1

GATTTTTGCGTTCTGTGCGAG DFCVLCE 1

TTCTATGAGCGGGTGGAGACG FYERVET 1

GGCGGGAAGGTCGCGCCGCCC GGKVAPP 2

CGCGTCCAGAGCCCTTGCAAC RVQSPCN 1

TGGGTCCACTGGAGCTGGTCG WVHWSWS 1

CGGTCCTCTATTCGCGTGCTG RSSIRVL 1

GGCGTCGGGGAGCTTCGGATG GVGELRM 1

GGTGTGGGCCTCTCGACTCGG GVGLSTR 1

GTTTTGCGCACTAAGATCGCT VLRTKIA 1

GGCAGGGATAGTGGGCTGTGC GRDSGLC 1

GCCGGGGCGGAGGCGCTGGGT AGAEALG 1

ACGTTGTGTGCTGGCCGCTGG TLCAGRW 1

ACGTCGGGGAGGGGCTCGATG TSGRGSM 1

CCCGTTGGTGGGGCCATTCCT PVGGAIP 1

TCGCTCCTGGGGTGTTATTTG SLLGCYL 1

GGCGTGTCCAGCGTGATTCGC GVSSVIR 2

TCGTGCTGGAGGGAGGGTGAT SCWREGD 1

GGCCGGACGCTGAGTATGGAT GRTLSMD 1

TTCGTTGGCGTGCCGATGGGT FVGVPMG 3

CGGGCGCAGTCGGGCAGGGAG RAQSGRE 1

GATGTGTGGCGCGTTCTCTCT DVWRVLS 1

AAGTGTTTTAGCAGCTTGTCG KCFSSLS 1

CTCCGGAACCGCCTGTGTCGG LRNRLCR 1

ACCCGTGTGTGCAGCGGGAGG TRVCSGR 1

CGTGTGGCTTTGGTCGTGAGT RVALVVS 1

GTTCGGGGTAGCGGTCACCTC VRGSGHL 2

AGGAACACCGAGTGCCGCTCT RNTECRS 1

GTTCTGGGCAGTCGCAGTTCT VLGSRSS 1

CGGTGTAGGCCGTGGGTCGGC RCRPWVG 1

TGTCCGGACATGGCGCTTTCT CPDMALS 1

TCGTATGTCGCGCATGCGTGG SYVAHAW 1

GCTTGCTGCCCGGGGGTCCTG ACCPGVL 1

CGCGCTGTCGTTGAGAGGCCG RAVVERP 1

AGTGCCTATAGGGTGTTTCGG SAYRVFR 1

GTTGCGTCGCGGCCTGCTGTG VASRPAV 1

CTTCTTTCCCGTAATCGTTAT LLSRNRY 1

TTGGCCAGCAGGCCGCTTGGC LASRPLG 1

TGCTGCCACCGGGCTTTGGAT CCHRALD 1

GGCCAGGGCGATATTGCTTGT GQGDIAC 1

CCCTACTCGTTGGGCGATCTG PYSLGDL 1

GGCGGGGGCGCGAGGGCGGTT GGGARAV 1

CTTGGCAGTCCGGGGCTCTCG LGSPGLS 1

ACGGGCGATGTCATGTGTCGG TGDVMCR 1

CACCTCTGCTACCCGAACCTG HLCYPNL 1

TGTAAGGTGTGCAGCCGTCCT CKVCSRP 1

CGGTGCTGTATGGCCTGGAGG RCCMAWR 1

CCGGTCGTCGTTCGCCAGACC PVVVRQT 1

TTGTGTACGCAGTGCTATTAT LCTQCYY 2

GGGTTCCTTTACACTTTGCGT GFLYTLR 1

AGGGTGGGGTTTTACATGAGT RVGFYMS 1

TTGAATGAGTGGTCGCTTCGG LNEWSLR 1

GCTGGCGCCTCGCAGTGGCGT AGASQWR 1

TTCAAGCGCCGGCTCACGACG FKRRLTT 1

GATGGTGGTAGTAGGGGGTAT DGGSRGY 1

CCGCAGCCCGTGGTCAGTTGG PQPVVSW 1

ATGGTCGGTTCTATGCCTGTG MVGSMPV 2

GTGGGCCCTAGGATCTGGCCG VGPRIWP 1

TCTCGGAGCTGGAGGGTGAAG SRSWRVK 1

CGCGGGCTGGTGCTTTCGCCG RGLVLSP 1

AGCGTTAATCTGGTGCTTGGT SVNLVLG 1

TCCATCTGGTTCAACACGCTG SIWFNTL 1

CATGCGCTGGGCGGTGAGAGT HALGGES 1

ATCCCGGATATCGCGGGTGGT IPDIAGG 1

ATGTACCTGACGCTGGCTGGG MYLTLAG 1

GGGGGCCTCGGTCGCTTGTCT GGLGRLS 1

GAGCTCCCGCGCGAGTTTAGT ELPREFS 1

TGGACGCTGAGCTTCTCGAGG WTLSFSR 1

GAGACCTCCCGTCTCTGCAAT ETSRLCN 2

AGTCGTAAGCTGGGTTCCACT SRKLGST 1

GAGGGGCAGACTAGTAAGACT EGQTSKT 1

AGCTTTATGTGGCCGTGCCCG SFMWPCP 1

GCGTGGCTGAGGACTCTGCAT AWLRTLH 1

TTCCGCGCCGGCGCTCTTTCT FRAGALS 1

TTTTACCGCAACGGGCTCTGT FYRNGLC 1

TGCAGGTACCTGGTCCGCCAC CRYLVRH 1

TGGAGCCGTCTTGCCTCGCAT WSRLASH 1

CGCCCCTGTGGGGAGACCCTT RPCGETL 1

TCCAATGGCAGCCGCGGCTAT SNGSRGY 1

ATTTATGGCATTGTGGTGGTC IYGIVVV 1

GTCGTGCAGCTGGCCGGTAGT VVQLAGS 2

GGCGACTCCAACGAGGTCGAG GDSNEVE 1

GCTCCGGGCATGTGCGGCGCG APGMCGA 1

AGGAATATCCGTTCCGGTTTG RNIRSGL 1

AATGGGGCCATCCGCGATGTT NGAIRDV 1

TTCGGGATGAGTGGCAACCGG FGMSGNR 1

TGTAAGCGGGGGTCCTGCTGT CKRGSCC 1

GTCTGCTTCCGGTCGGATTCT VCFRSDS 1

GAGACTTCTCGGGCTTCGGGC ETSRASG 1

GGCTGCAATGAGACTGAGCGT GCNETER 1

GGCCGCGATCACAGGGCTGTT GRDHRAV 1

TTGCACTGGACGAGGTCTTGG LHWTRSW 1

GGGGCTCACACGTGGGTTAGT GAHTWVS 2

CGGGCGATCGCTGATGAGTTG RAIADEL 1

TGGTGGTTCACTTTTCTCTTT WWFTFLF 1

CGGATCCGCTGGCGCACTGTT RIRWRTV 1

ACTTGTTTGGCTGGGTTGAAT TCLAGLN 1

TCTGCTTTCTACGGTTGTTGG SAFYGCW 1

GCTGTTTTCCGGTTCGATAAT AVFRFDN 1

TGGTGGATCCTGTATTGGTCT WWILYWS 1

CAGTGGAGGCAGGCTCAGCCT QWRQAQP 1

GGGAGGGATCCCTGTAGGCTT GRDPCRL 1

CCGATCCTCACGGTTGGGGGC PILTVGG 1

CTGGTTCATAGCTTTCATGTT LVHSFHV 1

GGTGGCCAGAGTGGGTTCCCG GGQSGFP 1

GATGAGCGCTGTCAGGGGCAT DERCQGH 1

GGTATTTTGCAGCCTGGTCTG GILQPGL 1

TGGGAGGGTAGGACGGGGAAT WEGRTGN 1

TTGAGTTACAGTGCGGTGCCG LSYSAVP 1

GTTCCTACGACCCATCGGCAC VPTTHRH 1

GTCTATCCGGACGCGTCGCGG VYPDASR 1

TCCCAGCCGCGTAAGTCGCCT SQPRKSP 1

CACACGGATTGTCTCTTCTAT HTDCLFY 1

AGCGGCTGGATCAGGCGCGAT SGWIRRD 1

GTTTGCTATTCGTGCGAGACT VCYSCET 1

GTCCGGAGTTGGGCGGTCCAG VRSWAVQ 1

GGCGTTCGGCCGGTCGGCCGT GVRPVGR 1

TACCGGATGTGGAGGCTGCTT YRMWRLL 1

ACGGGTGATGGCCTGTGCCCC TGDGLCP 1

CGCGGTTGCAGGGTGCGGGCG RGCRVRA 2

CCCGGGCGCTCCAGGAATTCG PGRSRNS 1

AGGGGGAAGGGGGATGGCACG RGKGDGT 1

GAGTTCTATAAGTTTTTGATG EFYKFLM 2

TTTAGGACGGTCCGGCTTGAC FRTVRLD 1

TGTTGGGTTGTTGCCATGGCT CWVVAMA 1

ATGGGCCCCTGGATGGAGCGG MGPWMER 1

GTGAAGGACGCTCGGCTTGGT VKDARLG 1

CTTGTCAGGGTGGTGGTCGTT LVRVVVV 1

CTCCCCTGCGAGCGCGTGTCC LPCERVS 1

AAGAACAGCGTGCGGCTCTGG KNSVRLW 1

AATTGGCAGGTGTACCTTTGT NWQVYLC 1

GGTGGGGGCGTGGGGCGTCAC GGGVGRH 1

AGGAGGTTCAACAGGGTGTGT RRFNRVC 1

GCCGTGCTGTATCTCTCGGTT AVLYLSV 1

GTTCCTATCATTGGGGGCCGG VPIIGGR 1

CACAGTTCTTGGGTCAGTCAT HSSWVSH 1

TCGCCGCTCCTTATTATGGTG SPLLIMV 1

TGCAGCGGCGGCTGGCCGCGG CSGGWPR 2

ACGAACGGGATGTTCGTGGAT TNGMFVD 1

GGCGTTCTGACTCGCAACCTT GVLTRNL 1

GGGGGGATTTATGGCGGGCTT GGIYGGL 1

AGTCTTCAGCGCTCGAGCATT SLQRSSI 1

ATGGACGCTAGCACCGTTCCG MDASTVP 1

CCGCCGCGGGAGGTGGTTAGT PPREVVS 1

CTGGGCATGTGGAGCAGTAGG LGMWSSR 1

TGGGAGGATAGCGAGATTTCT WEDSEIS 2

CGGGTGGTGAATCTCCACCTG RVVNLHL 1

CGCTGCGTGTATGGCTTTCCT RCVYGFP 1

ACGGAGTCCAGTTACACGTCT TESSYTS 1

TGTGTGGTTATGTCGGAGGAT CVVMSED 1

ATTGCTGCCTGGGTCCTGAGT IAAWVLS 2

CGTGGGGACAATGCGGGTACG RGDNAGT 1

CGTGTTCACGGGCCTTTGAGT RVHGPLS 1

ATTCCGTTGAGGGTGAGGGAC IPLRVRD 1

GGTCGCAAGCGGGGTCTCAAC GRKRGLN 1

GAGGTTACGGTGTATACCGTT EVTVYTV 1

TATCGGTCGGTCATGCCTTCG YRSVMPS 1

TATTCGCGGGCGGCCGCGTCT YSRAAAS 1

AATCTTGGGCCGGAGTTCAGT NLGPEFS 1

CTGAGTCTTGCGTTTACTTAT LSLAFTY 3

CCCTCTACGGTTTGGAGTTGT PSTVWSC 1

GTTATCGCTGCGGATATGATT VIAADMI 2

CAGAGGGACTCTGGCCCCTAT QRDSGPY 1

GTCTGTTTTCAGCGGCTGACT VCFQRLT 1

CGGTGGCACTGCGCTAGGTTG RWHCARL 1

GGCAACTCGCTCGGGGACTGG GNSLGDW 1

GAGAGTCTTCGTATGCGGTGG ESLRMRW 1

GTGTTCGGTCCTCGCGTGGGC VFGPRVG 1

TGGTTTTATGCCGGCTCGGAG WFYAGSE 1

TTTTTGAGTGGGTTCGGGGCT FLSGFGA 1

GAGATGCCGACTGCTTTGGGT EMPTALG 2

TTCTACTGGAGGGCCAGTGTG FYWRASV 1

GTCGTTACGTTGGAGGGTTCT VVTLEGS 1

TCGTGCGGCGCTGGGCGTTGG SCGAGRW 1

TGCCGTGCTAGGGACCGGCCT CRARDRP 1

ACCCCGGTGTCGGTTTCTCTT TPVSVSL 1

CCTGGTGAGGGGATGACTGCT PGEGMTA 1

TTGGTTTTGGGTATGAGCGAT LVLGMSD 1

GCTGTGGAGATTGAGCTGCTG AVEIELL 1

CTGTCGTGTGCGGAGACTTCT LSCAETS 1

GTGGGTGACAAGTGTGGTGTT VGDKCGV 1

GCGGACTTCAGGGGCCTTTTG ADFRGLL 1

TCTGGCGCGGCTGGTAAGGGT SGAAGKG 1

CTCAACGCGGAGAATGTTCCT LNAENVP 1

GTGTCTACTTGTATGTATCTT VSTCMYL 2

CGGCGCGTCTGCGGCCTGGAT RRVCGLD 1

GTTGATTTCTGGCGCGAGTTT VDFWREF 2

GGGGTCAAGTGTGTCGAGACG GVKCVET 1

TTGGCCGTCCCTGGGAGCGGC LAVPGSG 1

TGCAGTAGGATCGGTTGCTTG CSRIGCL 1

CCCATCAAGGCTGCGGTTTGG PIKAAVW 1

GTCTGGAAGGGCGTGGCGTTT VWKGVAF 1

ATCTTCTGCGGGTGGTGCACT IFCGWCT 1

GGTTTGTGGCTGGTTCCGTGC GLWLVPC 1

CCGTTCGACAGGGACGGTGGT PFDRDGG 1

AGTGGGATGCGTCGTACGGGG SGMRRTG 1

CGCGAGTTCAGGTCTAGGGAT REFRSRD 1

GTTTCTACCGACTTTGCCGTT VSTDFAV 1

CCCCTTAATAGCGGCCACAGG PLNSGHR 1

GTGCGGCTGAGGTTCGAGTCG VRLRFES 1

CGCGGGCTTGCGGTGATGAGC RGLAVMS 1

GCGAGCGGTCTGGCGGTGTTT ASGLAVF 1

GTGGTTCCGTCGGACGTTCTT VVPSDVL 1

CGTCTGTGGCGTCGTCTCACT RLWRRLT 1

GATGACCTCCTTAGTCGCAAT DDLLSRN 1

TATCTTTTGCTGGGTTTTTAT YLLLGFY 1

AAGTTGGCGATGTGGCAGGTC KLAMWQV 1

GCTTGCAGTCTGAGGCCCTCC ACSLRPS 1

GTCATTAAGAGGGGGGCGGCT VIKRGAA 1

GTTTTGAGCGGCGCCACCCTT VLSGATL 1

CCGTTGGTCGCTGTCGGGTTG PLVAVGL 1

GGGAGTGGTCTCGAGCGTGTG GSGLERV 1

GTCAGCTTCTTCTGCAAGCGG VSFFCKR 1

GGCGGGAATGGGCAGCAGTGG GGNGQQW 1

CTTGGGGCGTCTGTTTCTCGT LGASVSR 1

AGCCCGTGGAGTACCCGGAGG SPWSTRR 1

CGCAGGGGGGTTATCGCGGCT RRGVIAA 1

GAGTCTGGGGCTAGGGCTCTG ESGARAL 1

GGGGGCAACTGTGAGGACGGG GGNCEDG 1

GAGGCGCTCGGCGGTGTCGCG EALGGVA 1

TACCCGTGCGGTTTCTGGCTT YPCGFWL 1

GAGGGGGTGAGCGATCAGGAT EGVSDQD 1

GTCGGGGTGCGTAACACGTCC VGVRNTS 1

ATCATGCAGTACAGTCCGCCT IMQYSPP 1

GGCGTCGCGATGTTCGCGCGG GVAMFAR 1

ATTCCGGTTTCGAACGATGCG IPVSNDA 1

GCGGCGTGGGGCGAGGATGAG AAWGEDE 1

TGGCATTTGTGTCGGGAGGCG WHLCREA 1

CTCGAGCACATGCTGTGTTGT LEHMLCC 1

GCTTGGTCCAGCCGGCGGTAT AWSSRRY 1

TGTATGCATACTGGCGCGCTT CMHTGAL 1

CAGGGGCAGTTCGCCTGGGAC QGQFAWD 1

TCGGGGCGGATCTTTGTGCAG SGRIFVQ 1

CAGGTTCGGGTCTGCCGGTAT QVRVCRY 1

CGGGGGTTCAACGCGTGGACT RGFNAWT 1

GGTCGTCGCAGCGTTGGGGAT GRRSVGD 1

CTGCAGGTTTGGTTCGCCAAT LQVWFAN 1

GTCCGCTCTTTCCATAGTTAT VRSFHSY 1

GGTCCGTGCGGGCTGCGGTGC GPCGLRC 1

GCCGGCATCAGCTTGGGGCAC AGISLGH 1

GGCAGGTGTCACGCCTCGATC GRCHASI 1

GTCCGGTTGGTCGAGTTCACT VRLVEFT 1

TCTCCGATCGTGGGGGCTTGC SPIVGAC 1

GAGCCTTACGGCTTCATTCGT EPYGFIR 1

GCCGTGGGCCCGCGTCATCAT AVGPRHH 1

TTTGACTTGGCGCGTGTGACG FDLARVT 1

CACTTCCGGAAGGGCGCGAGT HFRKGAS 1

ATGCAGCGGAGGCCGCTGGAC MQRRPLD 1

CTGGCGCCCCTTTTTCGTCGG LAPLFRR 1

ATGTCGCTCGCCGACGGCGCG MSLADGA 1

TTGGTTAGCGGGGAGGGTTCG LVSGEGS 1

GGCAACGAGTCCAGCCGCGCT GNESSRA 1

CAGGGGCTCACGGTGATGGGG QGLTVMG 1

GACTGGGCCAGGCTTCAGTCG DWARLQS 1

ATGGTTGTGGCCACGATGGCT MVVATMA 1

TCGTGGGAGCGCGGCGGGCTT SWERGGL 1

GTGGGCGGTCCGGGTGGTGAT VGGPGGD 1

TCTTGCATTCAGCTCTGGCTT SCIQLWL 1

GTTCTGTGGTGTCAGGTGTGT VLWCQVC 1

ATCGGGGCTGGTGATTGTGTT IGAGDCV 1

CATGAGCACAGCCTTTTGGCG HEHSLLA 1

GCTGGGTTTCTGGTCGTTTGT AGFLVVC 2

GTCGTCCAGTTTATCTGCCTG VVQFICL 1

TCCGATCGGCTTGCTACCCCG SDRLATP 1

TGTAGGCGCGGTCACGTTTGG CRRGHVW 2

GCTAGTGTCGTTTACGCTCCT ASVVYAP 2

TCTTTGATTCTGTTCTATAGT SLILFYS 1

GCTGTTGAGTGGTGGCTCGTC AVEWWLV 1

AGCCAGAGGATCACTGGCTGT SQRITGC 1

CGTCTCTTTTATCGTTGCTGT RLFYRCC 1

CTGGCTTCTCGGGTGTCGCGG LASRVSR 1

TCGCTTAGGGTTGGTAACCGT SLRVGNR 1

CGCGCGGCGGTCCGGTCTTTG RAAVRSL 1

CGCGTGTTGCGCGGCGGGCAT RVLRGGH 1

GGTCGCATGATCGTCTGCGGG GRMIVCG 1

CTCGGGGCGAGGCGTGAGAGT LGARRES 1

TCGTTGGTGGGTTCTGAGCCG SLVGSEP 1

GGGTTTGAGTCGTGCAGTTTC GFESCSF 1

GTCCTGCGTTCCATGTCTGCG VLRSMSA 1

CGCGAGGTGGACCCGAAGCTT REVDPKL 1

GGCCGGTTCACGGTTCGCGGT GRFTVRG 1

TTTAGGTTGAGGTGGTGCGGT FRLRWCG 1

GATGAGATGGGGTCCGGCGCT DEMGSGA 1

AGTAAGCATACTAGGGGGGCG SKHTRGA 1

CTCGGCCTTGCTGCGCACCCC LGLAAHP 1

TTGGCCTTCTTCCGTGATGGT LAFFRDG 1

CGTTGCATCGGCTGCCAGGGG RCIGCQG 1

CTGAGTCGCGGGTTGGTGCTG LSRGLVL 1

GCTGGTGTTGTGCTGTTGCGG AGVVLLR 1

GCGCGGCGTCTCCGGTTCGTT ARRLRFV 2

CCGCGCCGCTGGTCCTTTTCT PRRWSFS 1

CGGGAGGTGTGTCGGTTCGGT REVCRFG 1

CGCGGTTGTCCGTGGTTGTGG RGCPWLW 1

TGGTGGGCGGTCCGGGGTCAT WWAVRGH 1

CACTCGCGGCTGTTCTATCTG HSRLFYL 1

AACGTGCGGACCGAGCGTAGT NVRTERS 1

ATTGACCGGAGTAGTAGTATG IDRSSSM 1

ATTGACAATTTGTATGGGTGT IDNLYGC 1

CGGCTTGAGACCGCGCGGCCG RLETARP 1

TCCCTGAGTTTCGTGCCGGAG SLSFVPE 1

ACCGACGTGAGTCGCTGGGGT TDVSRWG 1

AGGTGCTTTTGGGAGCCGAGT RCFWEPS 1

CATTGGGCGTGTCACAGGAGT HWACHRS 1

GGTGTTTGCAGCGGCGGTAGT GVCSGGS 1

GTGTGCCAGCAGTCTCTTCGG VCQQSLR 1

CGGTCGGTGCGCTGGTCGCTC RSVRWSL 1

AACGCTCATTACCATCGCGTT NAHYHRV 1

GAGGATGTGACGGCTCGGGGT EDVTARG 1

AGGCTCTATCGTGCGGATTGT RLYRADC 1

CCTGCGGTGGGGGCGAGGTCG PAVGARS 1

TTGTTGGTTTGGAGGCGGCCT LLVWRRP 1

AGGCGTTTGTGCGCCTGCCAT RRLCACH 1

TACAGTGGTCTCAATTCCTGT YSGLNSC 1

GGCAGCAGCAGGCACGGGCCT GSSRHGP 1

CGGTGGGAGTTGTCGATGGAT RWELSMD 1

TTCTTCCGCTCGCATGTGGAT FFRSHVD 1

CGGTATCTCGGGTCCGGGCCT RYLGSGP 1

TCTTCTCGGAGCCTGTTTGAG SSRSLFE 1

TTTCTTCAGAGCGTCCGGTGT FLQSVRC 1

TTGTACCGGGCCAAGTATCTG LYRAKYL 1

GAGCGCCGTGGCTTGCCGTTG ERRGLPL 1

GTTCCTTGTGTGAGCGTCAGT VPCVSVS 1

AACTCCGCGGTGTCTTGTCAT NSAVSCH 1

GTCCTGATGGGTCTCATTCGG VLMGLIR 1

GCGAAGCTCGATGGGGAGTCG AKLDGES 1

AGCTGGGGTATGGCCACGCTT SWGMATL 1

CAGCACTGTTATCGTTGTCTC QHCYRCL 1

CGCGTGGATGTGCTGGCTCTT RVDVLAL 1

GCGACTTGGATCGGGACTATT ATWIGTI 1

CTGTGTGATGGTCCTTGGTCT LCDGPWS 1

GGGGTTAGTGCCATTGAGACT GVSAIET 1

CATCTCTGTTTCCGCGGTATG HLCFRGM 1

TTGCTTGCTCGGGTGTGCGCT LLARVCA 1

TTGTGGTCGGCGGGCTGTTGT LWSAGCC 2

TTTCTGCTCATCTGGTTGAAT FLLIWLN 1

GTGGTGAACAGTATTGAGAGT VVNSIES 1

ACGGTGCGGTCCCGGTGTGGG TVRSRCG 1

TCGTTGCTGTGTAGTGATACT SLLCSDT 1

GGCTTTTGGCGGCTGCCTTTC GFWRLPF 1

TGCGCCGCCTGTTTGGAGGTT CAACLEV 1

TTCTCCGCCAGTACCAGCGTT FSASTSV 1

TCCTCTAACAGGGCGGACTCC SSNRADS 1

TTGCGGTGGTTGGTGCGCAAC LRWLVRN 1

TGGCAGGAGGCGCCCCCCAGC WQEAPPS 1

GCGCGTAGGACTGATACGCGG ARRTDTR 1

AGCATGGATAGGGCCCGCCGT SMDRARR 1

TCGTTTGCCACGAGCTACCGT SFATSYR 1

CTTATCTGCGAGCTCGCTTTG LICELAL 1

CCTGGTTTTCGTTTTCATTAT PGFRFHY 1

GTTGCTTGCTCGGCCCACCCT VACSAHP 1

CTTGAGCGCGGGTTGTGTAGT LERGLCS 1

CGGGGGGACTGCCCTAAGTGT RGDCPKC 1

GTGTACCGGGAGGGGTTTAAT VYREGFN 1

CAGGCGCGGCGGACGGGGGGG QARRTGG 1

CTTGTGGCTCGGAGTTGTCGC LVARSCR 1

GGCGACCTCAGGCGGGTTGTG GDLRRVV 1

TGGCTGGGGAGCGCGAGCGCT WLGSASA 1

AGTGTGCTCGTCTCGCTGGCG SVLVSLA 1

TGCGTCGTGGCTTTGGCTATG CVVALAM 1

CACTGTTCGGGTGGCGTGGCG HCSGGVA 1

GACTGGCGGAGTGTTGGTCCG DWRSVGP 1

GCGCTCGCGTGGGGCCGGCGG ALAWGRR 1

TGCACTTTTTGTTTGTTGTGG CTFCLLW 1

CTCGGGCTCTCCCACTCTGCG LGLSHSA 2

GGTGCCGGGGTTGTTCTCTTC GAGVVLF 1

GGGTGCACTAAGTGCACCCAG GCTKCTQ 1

CTCGGTTTGCCTGTGCCGTGT LGLPVPC 1

ACCTGGCGTCTCATGGCCAGG TWRLMAR 1

GAGTGGAGTGGCGCGGTGGTG EWSGAVV 3

AGCTCGCTGCCGCCGCTTCCG SSLPPLP 2

GCGGGCTTTTTCGGGTGTGTT AGFFGCV 1

GTGGTCGCGCGGCACGGGTGG VVARHGW 1

CGTCGGCAGTATGGCGCTGGG RRQYGAG 1

TCTCGCATTCGTGTGACTTCG SRIRVTS 1

GAGGGTTTCACTGTGCGTTGG EGFTVRW 1

CAGATTTGGATGATCGGGTCT QIWMIGS 1

TCTCATGCGTATAGTACTGGT SHAYSTG 1

ATGCTCCGGTTGTGTTTCCCT MLRLCFP 2

GGTGGTGTGGCGTTGCTGTGC GGVALLC 1

CAGGTGGGCCCGAGCACTATG QVGPSTM 1

ACTCATTGTAGGGGCTCTGAG THCRGSE 1

GGTGACTTGTGTACCGTGGCG GDLCTVA 1

GGCCGTACGTCTTTCGGTCGG GRTSFGR 1

GCTTTGCTTCAGGTGATGTCT ALLQVMS 1

GGTGGCGCGCGGGGCAGGGTG GGARGRV 1

CGGTCTTGGAACGCTGTTAGT RSWNAVS 1

ATTGAGGTGGCTTACGGTGTG IEVAYGV 1

TGTGACCTCACGGCCGCCGCG CDLTAAA 1

TGGGAGGTCCATGTCGGGTTC WEVHVGF 1

AAGCCGAGGGTGGCGAAGCGT KPRVAKR 1

GCGGTCGGCCCGCGGTTGGCT AVGPRLA 1

TCTCCCGGGGTTGGCATCCAT SPGVGIH 1

GGCTGGCGGTATAGCTCTAAT GWRYSSN 1

TGCACGGCCGGCAGCGCTTGT CTAGSAC 1

AGTCGCGACTGTGGGGACACG SRDCGDT 2

GCGTCCGCGTCTGGCGTGCTT ASASGVL 1

CAGGTTTTCTGGTGCGATAAC QVFWCDN 1

AATATCTCTCGGATCGACACG NISRIDT 2

TTGGGCTTGGCTTGCGGGTCG LGLACGS 1

ATTAGTGATCTCCGGGGTTAT ISDLRGY 1

CAGCACGAGCCTGTCACCGTT QHEPVTV 1

GTCCGCCTCCGTTCTTATACG VRLRSYT 1

CTGATCGACGATTGGCGGAGT LIDDWRS 1

GGGTGGGTGAGGGTCCTGTTT GWVRVLF 2

TCTCGGTTGAGTGCTGGGTCG SRLSAGS 1

GTTGGCGACGCTGGCGCTTGT VGDAGAC 2

TTGCGTGCCAAGGGCTTGCCG LRAKGLP 1

TTGCTGAACGCCGCCGTCCAG LLNAAVQ 1

GTCTGTGCGATGGCGTGGTCT VCAMAWS 1

CTGGGGAAGGGCACCGGGCAT LGKGTGH 1

GCGGCCCCGTCTTATGTGGTG AAPSYVV 1

CGTCCTAATATCACTACGCGG RPNITTR 1

CAGAGGCGCTGGGGCTTTACG QRRWGFT 1

GACGTCGGTCAGCTCTCGGCG DVGQLSA 1

GCCTCTCGGTACATTGCGGCG ASRYIAA 1

GGCCGGGGCAGTATTCACGAG GRGSIHE 1

TCCCGGGCGGCGGTGCAGACT SRAAVQT 1

GTGGTGCGTCTGTGCTACTGT VVRLCYC 1

CGGTGTTTCTGTGCGGCCGCT RCFCAAA 1

GGTCGCTCCGCCGTGATTCAG GRSAVIQ 1

GACTGGGCGACCTTGGTTGCT DWATLVA 1

GTTGGTGATTGGTCGCCTGGT VGDWSPG 1

CGCAAGGAGAGTCTGTTGGGG RKESLLG 10

AGCAATGCGTACTGTCTGGCT SNAYCLA 1

ACTGGCCGGCCGAGTTGGTTT TGRPSWF 1

TCGTGGAGGTTCTTTGTGCGT SWRFFVR 1

GCGCGGTTGGTGGTCTGGCTT ARLVVWL 1

CCCTTTGTGGATGCGCGGAAG PFVDARK 1

TTGAGTGGCAACCCGTTGGCG LSGNPLA 1

ATGGACCGCGCTAGGCGGCGT MDRARRR 1

GTCAGGGTGGGCTCGAAGCTG VRVGSKL 1

TGTTGCGGTATGTGGTTGGCG CCGMWLA 1

CCCCAGGTGAGCGTCCCGGAT PQVSVPD 1

GCTTTGCTTGAGTGCTCTGGT ALLECSG 1

TGGGGCGGTGCGAGTTGGCTT WGGASWL 1

CTGTATTTCGCGCTCTCGAAT LYFALSN 1

GTTGAGGGCGCCTCGGGTTAT VEGASGY 1

GGCGGCTATACCCTCCCGTCC GGYTLPS 1

TTTGTCGTTAGGCCTAGGAGC FVVRPRS 1

CCGCTGACGACGAATCAGGGG PLTTNQG 1

GGCGCGGTTAGCTCTGTGATT GAVSSVI 1

GACTGTGTGTTTGGGTGGCGC DCVFGWR 1

GTTTTGACTGCGAAGTGCGCG VLTAKCA 1

GAGATGGCCGGGCGGGGTCGG EMAGRGR 1

GCGGATGGGAAGGAGTTCGTT ADGKEFV 1

GTGTCTCTCAGCGGGGGGCTT VSLSGGL 1

TCGGGCGGTTCGTGGATCAGG SGGSWIR 1

GTTAGGTGGAGCTGCCATTGT VRWSCHC 1

GTTCGCCGGGCTTTTGAGTGG VRRAFEW 1

GCCGGGGAGGAGTGCGATTGT AGEECDC 1

GGTTGCCCCAGGCGCTCGTTG GCPRRSL 1

TCGCCGGGCTGGGGCTCTGGT SPGWGSG 1

AAGTCGACCCGCGAGGGCAGT KSTREGS 1

TTGCTTACGTGGTGGAACTCT LLTWWNS 1

TGTCCGTGGAGCCTCGCGCAT CPWSLAH 1

GTTAATGAGGAGGGGCGTTGG VNEEGRW 1

GCGGTGTTGGTGCGGGTTCCG AVLVRVP 1

TCTGACCTGCAGCATCTGAGT SDLQHLS 1

TGCTTTGGCGGGTGCAAGCTT CFGGCKL 1

TTGTCGCTGGGGAGGGCGCAG LSLGRAQ 2

CTCCTCCCGACGCGGGGTGGG LLPTRGG 1

GTGCAGAGGAGTCGTTCCCAT VQRSRSH 1

GCGCCGGCCCTCATCGGGACG APALIGT 1

TTCACGGTCAGTGGGTTGCTC FTVSGLL 1

ATCCTGGTCTTGCTCAGCAAT ILVLLSN 2

CCGCATGTCAGGATCAGTAGT PHVRISS 1

TGGGGCTGCGGCGAGCGCGGT WGCGERG 1

CGGATTCCCCCTTACGGGCAT RIPPYGH 4

GGGTCGTCGGTCGCCGTCCTT GSSVAVL 1

TTGTGGTGGTTGCTCCCTTCG LWWLLPS 2

GCTTTCCGGGTGCTTCACATG AFRVLHM 1

AGGTGGCGTATGGTTGCGGGT RWRMVAG 1

TACTCGCTGTTTTGTCCGTTT YSLFCPF 1

ATCAGTCGGCGCAGTTGGATT ISRRSWI 1

GCTAAGACTTCCGGGTTGTTG AKTSGLL 1

AACGTCGACAGGACTTGCGTT NVDRTCV 2

GGCATGTGTCTTAGTACCGGG GMCLSTG 1

GCCGTGTGTGATCTCCAGGTT AVCDLQV 1

GCTGGCAAGGATGGGTGCGGG AGKDGCG 1

GTGTGGTCCGAGGTCGCGGCG VWSEVAA 1

ATGGGTTTCGCGAAGTTCTTT MGFAKFF 1

GGGTGCCCGCACCTCCACATT GCPHLHI 1

CTTTGCGGTCCGTGGAGCGTT LCGPWSV 1

GAGGTGGATTGGGGTTGCCTG EVDWGCL 1

GCCGGGCCGCGCGTGTGTTAC AGPRVCY 1

GTTCATCAGCTCGGCTTGGGT VHQLGLG 1

TTCGACCTCCGTGCCGCTTCG FDLRAAS 1

GGGTCGGCGAGCTGTACTTGT GSASCTC 1

TGGAGCGGCGCCTGGTGGAGG WSGAWWR 1

TTCGATTGGGCGTGCGGTCGG FDWACGR 1

TCTGGGCGGTCGCGCCTGCGT SGRSRLR 2

GTTCCTGCGCTTGGCTCCGCT VPALGSA 1

TTCAGTCTTAGCTCTGCCGTG FSLSSAV 1

GAGTGGGTCGTTGTGGTCTTG EWVVVVL 1

CCTGGCAGGTCGGCTACCCAG PGRSATQ 1

TGGATTGCCAGGTACACTTGC WIARYTC 1

ATGGTCGTTTATATGAATTGT MVVYMNC 1

CTGTGGGCGGCGAGCGGTGTG LWAASGV 1

GGCATTCGCAGGGGGCGCTGT GIRRGRC 1

GATCCGGTGTCGTGTAGCCAT DPVSCSH 1

GGTATTTTCACCATCTTTATC GIFTIFI 1

GCGTTGGGCTGGAGTGAGGGC ALGWSEG 1

GATTGGGGCACGAAGGGGTGG DWGTKGW 1

CACCGTGCTTTCGTGGCTGCG HRAFVAA 1

GTGGTTCGGACGAGGTTGCCT VVRTRLP 1

GGGGCGGGGCTTCATTGGACT GAGLHWT 1

CTCGAGGTGTCGAGTTCGCGT LEVSSSR 1

GCCAATTTGGGGGAGGTCGTC ANLGEVV 1

GTGTGTATCGTCGGGGTGCTC VCIVGVL 1

CATCGTAGCGTCGAGGTGACT HRSVEVT 1

GGGCCGAGGCTTGTTGTCGGG GPRLVVG 1

GTTCAGTGGGTTATGGCGACG VQWVMAT 1

ATGGTGGTGGAGGGTCGTGCT MVVEGRA 1

GATATCTGCAATTGGCGCGCT DICNWRA 1

ACGCGGCCGTCGGTCGCTCCG TRPSVAP 1

TTTATGGACGTGAGCAGCTCT FMDVSSS 1

ACCTCGATTATGACCGCTGCT TSIMTAA 2

CCCGCGGCTTGTGACCAGAGG PAACDQR 1

CATCGGTCGAGGGCCTGTGGT HRSRACG 1

CTTGGCTTGTCTGCGCTGGCG LGLSALA 1

GGCCGCACTAGGGACTTGGGG GRTRDLG 1

GTGTTGGCTTCCGGCGGCGCG VLASGGA 1

ATTTATCTCAGCGCCTCTTCT IYLSASS 1

GTTCGCAGCTCTTGGGAGATG VRSSWEM 1

GGCCACGGTTGCTTCGCTTGT GHGCFAC 1

GTGTTTGGTCGCATGAACTGT VFGRMNC 1

CGCACTCGGCTTTGCCGCTAT RTRLCRY 1

GTGGTCGCTTACTGGCGGCTG VVAYWRL 2

TGCGGTTCTCAGTGCTCCATT CGSQCSI 1

GGGTGGGGTACCTTGTCCCAC GWGTLSH 1

TGTTCGCGCAGCCAGAGTTCT CSRSQSS 1

GGTCGGACCTCCGTCCTCCAT GRTSVLH 1

GTCTTCGCGGTGCGTGGTGCG VFAVRGA 1

GTGGTGGCGGGGACGGCCGGT VVAGTAG 1

CGGGTTTGCACCACCTGGTCT RVCTTWS 1

TCCCGGATGAGTGCCAGTGCG SRMSASA 1

ATGTGGCGGAGTTGGTCGAAG MWRSWSK 1

TTGTGTTGCGTGCTGTATGTT LCCVLYV 1

GAGGACAGCAGGTGTGGGCCT EDSRCGP 1

GACGTTAAGGCCGCGCACGCT DVKAAHA 1

TACGTCGTTTGGGGGAGTTGC YVVWGSC 1

GGCAGTATGCGGGGTCGCTGC GSMRGRC 1

ACGAGGGTCACGGGGCGTCTG TRVTGRL 1

CCTGCCCTGGGGCTTGCGGCT PALGLAA 1

GTGGCGTTCGGGTGCCGCCCT VAFGCRP 1

CAGGTCTTTGCTCTGAGCCTG QVFALSL 1

TATCGGTTTGGCAGGGGTCTC YRFGRGL 1

GTTATCTGCGAGGTGACTGCG VICEVTA 2

CTGGGGACCTCTTACACCATG LGTSYTM 1

CGCTCTTGTTTGACCGCGATT RSCLTAI 1

GGGACCCTGGCTCGGCTTTAT GTLARLY 1

AAGCTGGGCAGGCACGTTGTG KLGRHVV 1

TGGAGCCATGGGTGTGGCTGT WSHGCGC 1

ATCCCGCGCGTTGTCTGTGGG IPRVVCG 1

GAGCCCTGTCTCCATAGGCGG EPCLHRR 1

CAGCAGGGGACCCTTGGTTTT QQGTLGF 2

TGTGGGCATAGCGCCTGGGAG CGHSAWE 1

TGGCGCCGTTGGAGCTGCGGG WRRWSCG 1

GTTGCTCACGGGCGGCGCGGT VAHGRRG 2

CCTGTCATGGCTGTCGAGTGG PVMAVEW 1

TGGGGTTTCGGCCGGCGGCAT WGFGRRH 1

GGCCGGTGCGCGCTGAGGCTT GRCALRL 1

TTGGGGTCCATCAGGTGTCGG LGSIRCR 1

TGTCGGGCGACCTTCAGGTGC CRATFRC 1

CTGGTCTGCTCCTCCCAGTTT LVCSSQF 1

TTTGGCTACTCTTGGGGGCCG FGYSWGP 1

GCTTGTAGCATGGTCATGGTT ACSMVMV 1

GTGGCGCGCCCCCTGATTGCG VARPLIA 1

ATTAAGCATTATCACAATTCT IKHYHNS 1

CGTACCGGCAGGACCCGTCTG RTGRTRL 1

AATTGTCCGCTGGCCGCGTCG NCPLAAS 1

CACTTGTACTCTCTCCTGGAT HLYSLLD 1

CACCGGCTGTTGGTGTACGAT HRLLVYD 1

AACGCGTTCGTGTCGTCGTTT NAFVSSF 1

TCCAAGCAGCGTGGGTCTCCC SKQRGSP 1

CATACGATGGCGTGGGTTAGT HTMAWVS 1

GGCCAGGTTTTGGACCGCGGC GQVLDRG 1

GGCTCCACCGGGGGGTTCCGT GSTGGFR 1

AGGCGTTGCTCGTTCTCGCTT RRCSFSL 2

CCGGCCGTTGACCGCGCGTTG PAVDRAL 1

CCGCAGCGCCGTCAGCTTGGT PQRRQLG 1

CGGCTGGGCATTGTTGGGTAC RLGIVGY 1

TCGCTCGAGTGGTACTATAAG SLEWYYK 1

CCGGTGTACCTGGAGTGGCGT PVYLEWR 1

TGGCGGCCTGACAGTATGTGT WRPDSMC 1

CGGTCGGACATTGTGTGTCGT RSDIVCR 1

GATGTGGTCGACGCGAACGCG DVVDANA 1

TCCTTGGACTTGGCGAGTGGC SLDLASG 2

TTGTGTGATTATGATCATTGG LCDYDHW 1

TATACGGGGAGGGAGGTTGGT YTGREVG 1

TACGCTGTTTGGCATTTGCCG YAVWHLP 2

TTCCAGTTTCGCTGCAGGATT FQFRCRI 1

GTGCAGACTGTTTGGGGTTCT VQTVWGS 1

CTCTGCGCCGACGCGGTGATG LCADAVM 1

TATGCCGCTCCTCCTGGTGAG YAAPPGE 1

TGCCGTGCCTGTTTCAACATT CRACFNI 1

AAGAACATTGAGAATTCCACG KNIENST 1

TGGCCGCATGGGACCTTTATT WPHGTFI 1

AATGGGACTCGTTTGTGGCCT NGTRLWP 1

GCGGGTTTTGACCCTCGCGTG AGFDPRV 1

GTGCGTTCGTTGAGGGTCTGT VRSLRVC 1

GGGGTGGTGACTCTGGCGTAT GVVTLAY 1

TACGTCGGGTGCGATGGCACG YVGCDGT 1

TGGGGTATGTCCTCCGTCGTT WGMSSVV 2

TACTCCGAGGTGGGCCGGGCT YSEVGRA 1

TGCCCCCGGATTTGGCGTCCT CPRIWRP 1

CCTGTGCAGGCGCTCGCTGCT PVQALAA 1

AACCTTCACAGCAGCTGCTTT NLHSSCF 1

GCTTTGTGCATCCTCCGGCTG ALCILRL 3

AGGGGGAGCACCCGCTGCCAT RGSTRCH 1

TACAAGTGTTCCTGTTTTCAG YKCSCFQ 1

TTTAGGGGCGGCACCGTGTCT FRGGTVS 1

GTTTTCCTCAGTGGTGCCCGT VFLSGAR 1

ATTGCGCTGGTGAATGCTATC IALVNAI 1

GATGGTTGGTTCTATCTCTCT DGWFYLS 1

GGCGGCGGGGTGGTCCGGGCG GGGVVRA 1

GTTAACCGCAGGGGCTTCATT VNRRGFI 1

GGGCGGCACTTTTCGGCCCGG GRHFSAR 1

CGTCAGCACGGGAGTTCCCTG RQHGSSL 1

TGGCGGTGGGAGTATGAGTCG WRWEYES 1

AGGCTGCGCCCGGATGTTCCT RLRPDVP 2

ATCGCTGTGGGCGCTGTGATG IAVGAVM 1

CCGGGGAGCCTTTCTTGGCAG PGSLSWQ 1

CCTAGGACGCGGTGCACGGCT PRTRCTA 1

GTCTGTCACTCTCGTTTGAGT VCHSRLS 1

CGCGACCTCAGGCTCACGTGG RDLRLTW 1

TATCGGCCGGCTTACGGTGGG YRPAYGG 1

TCGTTGCGCCAGAGCTGTCAT SLRQSCH 1

CCTCGGGTCGAGGCGCGGTGG PRVEARW 1

CGGCTGGATAGGTCGAGTGGC RLDRSSG 1

AGCTTTGTCACCTCCCTCCCT SFVTSLP 1

CAGGTTCTCAGGAGGGAGGTT QVLRREV 1

TATGGGGGGAGTCGCATCATT YGGSRII 1

CAGTCTTACAACTTTGTGTCG QSYNFVS 1

CCGTGTAGGAAGTGCTCCTTG PCRKCSL 1

TCCACTGCCCTGGGTCGGCGT STALGRR 1

GGGGTCCGTTCGTGGTTTCCG GVRSWFP 1

GCGACGCAGACCGAGGGTATG ATQTEGM 1

GTGCGCCGCAGCTATCATTAT VRRSYHY 1

AACGTTTTTTGCCTCTGTTGT NVFCLCC 1

GTGGGGGTCTGGGGTTTGAGT VGVWGLS 1

GCCCATGAGGTTGTCGGGCTC AHEVVGL 1

GGCACCGTCGATCGTGTTGTT GTVDRVV 1

GATGCTGTGTGTAATGCGGGC DAVCNAG 1

GGTTGGTCTATGGCTGCTCCC GWSMAAP 1

ACCTTGTGTTCGAAGAGGGTC TLCSKRV 1

CCGCTGAACAACGGGTCCGTT PLNNGSV 1

GGCCATTATAGGATCGAGGTC GHYRIEV 1

GGTGTTCATGCCTGCCGTGAT GVHACRD 1

CGTGTCGCGCGCCTGATCGAC RVARLID 1

GCCGGCTGTGACAGCTTGTGG AGCDSLW 1

GTGGGCGTCGAGTCTGTGTCC VGVESVS 1

GTGCTGGGCTTCCGGAAGAAC VLGFRKN 1

ATCCGCGCGGCTGGGGGTTGG IRAAGGW 1

ATGATTATTTTTCGCCGGCCG MIIFRRP 1

TCGTCGTGGTTGCGCGGTAGT SSWLRGS 1

GTGTGCTGGATGGCTGTTCTG VCWMAVL 1

ATGAGGGTCTTGCGGGACGAG MRVLRDE 1

TGGTGCTTCCTCGGCTCTGCG WCFLGSA 3

TTTAGGGAGGCGGATAACCCT FREADNP 1

CAGACCGTTTCCTCGCTCTCC QTVSSLS 1

CCTCATCGTGTCGCCGGGAAG PHRVAGK 1

ATGATGGATATTGTCTGGTCG MMDIVWS 1

TCTTATCGGGCCAGGGGGTGG SYRARGW 1

GTCTTTGAGGGTCAGAGGGCT VFEGQRA 1

AGGCCGTCTTCCTTGTTGGAG RPSSLLE 1

GAGCAGGGTGTGTATTTTCGT EQGVYFR 1

GGCATTCACGTCTCCATTAAT GIHVSIN 1

CAGCCGCACAGCTTGGGCGAG QPHSLGE 1

AAGGTTAATGCCGGGTGGTTT KVNAGWF 1

GATAGGTGGCAGGCCTCTGCG DRWQASA 1

GCGGCCCGTGTGCCCTCGAGT AARVPSS 1

GGTGAGCGGGTCCAGCGTCAC GERVQRH 1

GCCCGTCTGGATGTGCCTAAT ARLDVPN 1

GGTCGCCCGTCTTGCAGCTCG GRPSCSS 1

GTTTTGCGCACGCGGGGCAGT VLRTRGS 1

GCCTGTTTGACCGCGGCCTGG ACLTAAW 1

GGGGTGGTTCCGTGGGATGTG GVVPWDV 1

TTCGAGGTGTCTTTGTGTGCT FEVSLCA 1

TGGTCGGCGATTCGTGGGCCT WSAIRGP 2

TCGACGCCTGCGGTGTGGATT STPAVWI 1

GCTCCTCAGCGTCGCCATTCC APQRRHS 1

GCGGTGCCCGCCACCGTCGTG AVPATVV 2

GCTTTGATCATGCGCCCCGCG ALIMRPA 1

GTGGGCCTCCGCGGTGTGCGT VGLRGVR 1

TGTCGGGTCACTCCTCACCTT CRVTPHL 1

ACTTGGCCGTGGGGCGGGTGG TWPWGGW 1

TCGGTCAAGATCTGGTGGCAT SVKIWWH 1

AGTGGTCGTCACGTCGGGAAG SGRHVGK 1

GACGGTTTCCTCTATGTGGAG DGFLYVE 1

GGGCGTTTGCCGCAGGCCTGT GRLPQAC 1

TTTGCTGGCCTTAGGTGTGCG FAGLRCA 1

CTGGACGCCGCTCTCAGTGCG LDAALSA 1

GGCAATTGGCTGAGGCGGCCT GNWLRRP 1

GTTGCGCTCGATCTGGACTCG VALDLDS 1

GTGCGTGTCCCGTGCGGGGGG VRVPCGG 1

TTGGTGTGGGAGAGGTTTCAG LVWERFQ 1

TGGGGGTATCCGGACTGGTGG WGYPDWW 1

GATGGGCGCTCGGCGGTTCCG DGRSAVP 1

CGGCGGGGCTGGATCGGGTTG RRGWIGL 1

TTCCCCCCCGCTGCGGGTACC FPPAAGT 1

AGTGCTTTGAGCGCGTACGAG SALSAYE 1

CGCTTCGGTTTCCGGTGGCTT RFGFRWL 1

CATACCTACGTGCTCGTGCGT HTYVLVR 1

CAGCTCGCTCAGAGCTCTGAG QLAQSSE 1

GATCGCCGCGGCGTTGGGGTT DRRGVGV 1

GATTGCCAGTCTAGTTGCGCT DCQSSCA 1

TGGATGGCCGGTTCGGGTGCT WMAGSGA 1

AGGATCGTCTGGGCGTCCGTG RIVWASV 1

AGGCCCATTATGGGGTCGGTT RPIMGSV 1

CTGCGCCGCTTTAGCAGCCGG LRRFSSR 1

TATCTTAGGATGGTGCGCCGG YLRMVRR 1

CGGTGGCTGTCGCAGTCCGAT RWLSQSD 1

GGCGGGAACAGCCTGGCTCCG GGNSLAP 1

ACCCAGAACATGGGTTGGCAG TQNMGWQ 1

ATGTACAGCACGAATGGGAGT MYSTNGS 1

GCTGGGCTGGGCTGCGTCTTT AGLGCVF 1

TGCCGGCGCCAGGGGTTCTCG CRRQGFS 1

GGGGTCGCGGATGCGACTGGC GVADATG 1

TTGATTATCCGGTTGGATCAC LIIRLDH 1

TGTCTCGCTAGTAGTATTGAT CLASSID 1

TGCCAGGTTAGCGTCTTCGCT CQVSVFA 1

AAGCGCCACGCCAACCGTGGC KRHANRG 1

TGTTATCATGGCTGTCGCGAC CYHGCRD 1

GTCTCTCGGGGTGGGTACCGC VSRGGYR 1

CGTGGTGTCGACGGCGTGTTT RGVDGVF 1

CTCGACACTAGGTGCGTGGGT LDTRCVG 1

CGGCGGTGCTTGAGCTTTCAG RRCLSFQ 2

CGGCGTGCCTCCTTCTCCGAT RRASFSD 1

ATGGGCCGGCCGAATGTTGGT MGRPNVG 1

TTGCAGCAGACTTTCTGCGGT LQQTFCG 1

GTCCCTTATTCCGACACGGTT VPYSDTV 1

ACCGGCGCTCGTCTCCATTGT TGARLHC 1

TTTGCTGTCACTAGCTTCGGT FAVTSFG 2

CGCGCTGATTGCAACTTCGAT RADCNFD 1

TGGCCGGGTAACGAGTTGTGT WPGNELC 1

AGCGATAGGTGTGCTAGTAAT SDRCASN 1

CTCGCCGTCAGTGTGCCGACT LAVSVPT 1

TCTTTGCTCTACCTCCACTGT SLLYLHC 1

CCGCCCATTGGCGGTTGGGGG PPIGGWG 1

CGGAGTGGGGCTCGTGGCTGG RSGARGW 1

GGGTCGGAGAATAGTTGGATT GSENSWI 1

CGCTCCCGCGGTGACCCGTCT RSRGDPS 1

GCCATTGAGACGGACGGGGCG AIETDGA 1

TTGCTTTGCCTGTGTTCTGGC LLCLCSG 1

TCCGTGACCAGCGACCGTGGC SVTSDRG 1

TGCGCGCGGGCTGGTTTCGCT CARAGFA 1

GTTGGTCGCGGGGCCCTTCAT VGRGALH 2

CTGCGGCTGCAGCGTGAGGGG LRLQREG 1

GATCTTCCGTCGAGTGGTTCG DLPSSGS 1

TCTTTTGGGCCTGAGTCTGCT SFGPESA 1

AGGGAGGCCGAGGGCTCGGGC REAEGSG 2

AGTTCTGGGCTTCGGGTCTCT SSGLRVS 1

GTGTCTAGTCTCTGGGTCGGT VSSLWVG 1

CGCGCGTACACGGGTTACAGC RAYTGYS 1

TCTATGGAGAGCTCGCTCTGT SMESSLC 1

GTGCTGTACATGTATCTGCTG VLYMYLL 1

GGTTTGACGTCCTGCGAGGTT GLTSCEV 1

CGTCCGTACCTGAGGATTGCT RPYLRIA 1

CGCTGTTTCCGGTGCAGCGTT RCFRCSV 1

GGTTGGCCTCCGTTGTATAGG GWPPLYR 1

TGGCCGGGGTTGTACTCGTCG WPGLYSS 1

GAGTTTCGTGGTCCGAGGGCT EFRGPRA 1

GCTCGTGACTATGCCGAGAGT ARDYAES 5

GGTGTTTGCGCGGGCGGTCTT GVCAGGL 1

GGCCCGTGCGAGACCGTTGGG GPCETVG 1

GAGGTCAAGGTGCCCAGTTTT EVKVPSF 1

AGGTGTAACGAGTCGCGGACT RCNESRT 1

TGGGACGGCATGTCGCCGGCT WDGMSPA 1

AATCTGCGTGGTCGCCGGAAC NLRGRRN 1

TACGCTGGTTGGGTGGGTGAG YAGWVGE 1

CGTGTTAACTTCGCCTTGTGC RVNFALC 1

AGGGACTTCGCCGTGGATGAG RDFAVDE 2

CGGGGTCTCCGGTGCCTGCAT RGLRCLH 1

GTCGGTCGCCATTGCTTCGCT VGRHCFA 1

GCGGCGGTGGCTTGCTTTGTG AAVACFV 1

CTCCGGACGAGTAGTGACGTT LRTSSDV 1

AGGGTGGTGAGGTGTGTCTGG RVVRCVW 1

TCCTTGGGTCTGTACGGTGGC SLGLYGG 1

GGTGTGAGCGGCGTCTCGGCT GVSGVSA 1

TGGGGGCGTGCGTCGGGTGCG WGRASGA 1

GGTGCGTCCAGGCCTTGGTGC GASRPWC 1

CTGAGCAAGAGGATTTATCTT LSKRIYL 1

TACGTGGCGTCCTGGCTGGAC YVASWLD 1

GGCTTCACGTTCGGGGATGTT GFTFGDV 1

GACTTTGGCGCCGCGGGGCCT DFGAAGP 1

CGCAGGGTGATTTCTGGCTCT RRVISGS 1

GCGTTGACGCCTCGCGTGCCC ALTPRVP 2

CGCCACCAGCTGAGGGAGCAG RHQLREQ 3

CTGGCTAGCCGCGGGCGCACT LASRGRT 1

TGGGGGTCGCGTGCGTTTTGG WGSRAFW 1

CCGGTGGTCAGGATGGTTAGG PVVRMVR 1

CGGATGGAGTGGAACAAGCCT RMEWNKP 1

GGGTACTCGAACGGGTTTGGG GYSNGFG 1

CGTTTGGCTTATCTTGGGGCT RLAYLGA 1

AAGGGCTCCGCTAACTTTCGG KGSANFR 1

GATCCGCTCAGCTTCTTGCAC DPLSFLH 2

ATGGTCTCGATCAGCTGGGTT MVSISWV 1

CACGGTCGTTGGGCCCGTTCT HGRWARS 1

GTGGCCACTAAGTTCTCCCCG VATKFSP 2

ATGCTGCTGCGTGAGTTTGCT MLLREFA 1

GGCATGCGTCCTTGTCGGTTT GMRPCRF 1

GCGGGCCGCGACGTGTTTCGG AGRDVFR 1

GGGCATGGGGTCGTGTATTCT GHGVVYS 1

GACCATTATACCTCGAGCAGG DHYTSSR 1

TGCATTGTGGCCAGTCTCGCT CIVASLA 1

TTTCCCACGGCGCGCCAGTGT FPTARQC 1

GCTTGCCTCGGGCGGTCGCGT ACLGRSR 1

GGTCCTTCCACGGGGTGGGCG GPSTGWA 1

GGCTCTGATGTTGGGCTGGAG GSDVGLE 1

GGTGTGTGTAACTGGAGGTGT GVCNWRC 1

ATCGACTCGAGCGGTCATTCT IDSSGHS 1

AAGGGCGAGAGTGACTGTGGT KGESDCG 1

CGTTGTAGTGTGATGGGCCGT RCSVMGR 1

GGTGCGCGGTGGTACGGCTTC GARWYGF 1

CCTCCTGCGGGTAATTTGCAC PPAGNLH 2

CGTCGCGCCGCTCTGTGCTTG RRAALCL 1

CGGGGTTATTCGCCGTGGAAT RGYSPWN 1

GCTGGGCTCGCTTCCATTGAT AGLASID 1

GTCGGTGTTCTGCTTCTCAGC VGVLLLS 1

CCGTGGATGCCTGGGCACCCG PWMPGHP 1

TTCTGGGTGGAGCTGTATCGG FWVELYR 1

GCTGCTGGGGGGATGCTCGTG AAGGMLV 1

GGCATGTTCCTTGTGTGGGGT GMFLVWG 1

TCGGTTCAGGTGCATCTTCGG SVQVHLR 1

TCTCAGCGTATCAGCTGTGGT SQRISCG 1

GGCAGTGTTCCGTGGGAGCCT GSVPWEP 1

CTGCGGTGCCAGGTGCGCCAT LRCQVRH 1

GGGGTCCGGCCTTGTGCTTCC GVRPCAS 1

GGTTGGTGCGCGGGGGGCGTC GWCAGGV 1

ATGGGCATCTTTGGGGTGATG MGIFGVM 1

ATCGATCCGTGGCTCGTTCCG IDPWLVP 1

GTGGAGCCGGCTATCGTGATT VEPAIVI 4

GAGTGGGCGACCTGCATGAAG EWATCMK 1

CCTGGTGACAGGACTCTGGCT PGDRTLA 1

ATCAGGGGCTACGTTTTGGGT IRGYVLG 1

GACATCCTCGGTGATCATGCG DILGDHA 1

GAGTCTAAGGGGACTCACTGT ESKGTHC 1

ATGAATCGTGGCGTCGATTCT MNRGVDS 1

CATTGTAAGGCGCGGAGTGGC HCKARSG 1

GGGTGCGCCCCTCTTGGTCGC GCAPLGR 1

AAGCTGCAGGTTCCGGGTCTT KLQVPGL 1

CCGCTTGTTCGCCGGTTGGCT PLVRRLA 1

ATTCGTGTGGTGTCGGATGGC IRVVSDG 1

AGCATGCCGCAGTTGTTTGGT SMPQLFG 1

GGTGACTGCGGGGGGGTGTCG GDCGGVS 1

TGGTTCGTCGGTTTGTTTGAC WFVGLFD 2

CGTAGGGCTTGGGCTCGTTCG RRAWARS 1

GTCTCTGAGAGGGGCGCGTAT VSERGAY 1

CTGGTGAAGGCGGTCCAGACT LVKAVQT 1

CGCCCTAACATCGGGCTCAAT RPNIGLN 1

CCCGCGTGGTCCAGCGTGGTT PAWSSVV 1

CTCCTTGCCGCGGGTCAGGGT LLAAGQG 1

CTTCGGTGGGTGATTGGCAAT LRWVIGN 1

GGGCAGGTCACGACCGTGGCT GQVTTVA 1

TGCCCGCGGTCTTTGCGCGGT CPRSLRG 1

ATCGGCGCGTGCGTGTGCTAT IGACVCY 1

CGGCAGAGGGCTAGCCCCTAT RQRASPY 1

CATGTGGGCAGGTTCTTTAAG HVGRFFK 1

GATGTGCCGTGGGACAGTGCT DVPWDSA 1

CTCCCTGCTATGTGGTTTTGT LPAMWFC 1

CTGTTCGAGCGTGTGGGGAGC LFERVGS 1

TGGGAGCGGTCGGGGGTGGGT WERSGVG 1

AATCACGCCGAGCTCCGCTGG NHAELRW 1

CCTTCGAGTGTGTCCGGTCTT PSSVSGL 1

CATGGTCGGCTGGGCCCGAGC HGRLGPS 1

TATCCCGCTAGGACGTCGGCT YPARTSA 1

ATGACGGCCATCTTCTGGCTC MTAIFWL 2

CAGTTCTCTGCGGTCGGGACT QFSAVGT 1

AGCAAGTATGCTCCTCTCATG SKYAPLM 1

AAGGATCAGAGTGAGGCCGCT KDQSEAA 1

CTTGGGGTCCGTCTCTGCGGT LGVRLCG 1

GACGCGAGCTCGACGGCGCTG DASSTAL 2

CAGCGGCCGGCGCGTTGGTGT QRPARWC 2

GAGTCGGGCGGCGGGTGCGCT ESGGGCA 1

GTTGTGTCGCCGGTGACCCTT VVSPVTL 1

TGTTCTAGCGGCACGAATACC CSSGTNT 1

AGTTCGTGCCCTGGCTCCTCT SSCPGSS 1

AGGTCCAGCAGGGGCAATCGT RSSRGNR 1

TCTGCGGTCCGGCCTAGGAGG SAVRPRR 1

CTTGTTGGGGTTCTGCTTTGG LVGVLLW 2

AAGTCGTCGCTCCTCACTCTG KSSLLTL 1

TGCCCCTCCAATCGGTTCTAT CPSNRFY 1

AGGAGCTGTACTAGGGGCACT RSCTRGT 1

TGCCATGGTGAGCCTGATCGT CHGEPDR 3

ATTAGTTACATTTGCATTCTG ISYICIL 1

AGTCCTGGCGGGCAGAATGCG SPGGQNA 1

CTTGTGTGGATCAACGTGAGT LVWINVS 1

GTTGAGGAGTTGCGGCGTAGG VEELRRR 1

AGCGTTGGCACGTGCGTTGTT SVGTCVV 1

GCCTGCGGCATCAACCTGTGT ACGINLC 1

GTTGGGTGGATGGTGCGGCTT VGWMVRL 1

TACGGCCGGGGTATCAACTGG YGRGINW 2

GGCGGGAGCCCGAACCAGCGG GGSPNQR 1

AATGTGATGAGTTCGCGGAAG NVMSSRK 1

TTTTACTGGACTGGGAGCGGT FYWTGSG 1

AGTTTGTACCCTGGGCCTTTC SLYPGPF 1

AAGAGTTGCAGGCCGACGACG KSCRPTT 1

GTGCGGCCGGCTGATAGGCCT VRPADRP 1

TGTGGGCCTGTGGTCGCGCCT CGPVVAP 1

TGCGCGAAGCATGTCTACTGC CAKHVYC 1

TGCGATCGGAGGGGGTATTGG CDRRGYW 1

ATTCGCGGTGGGTTCGCTTCC IRGGFAS 1

TCGGGCGTCTGTGCGTTGTTT SGVCALF 2

GTTCGCAAGAGCCAGTACGGT VRKSQYG 2

CGGTGGTCTTGTTTGTCGCCC RWSCLSP 1

GCCCGCTTGAGTGGCTCGTAT ARLSGSY 1

TTGGCGTCCTTGGACGTGTGG LASLDVW 1

AATCGCTCCTTTTATGTTCTG NRSFYVL 1

GTGTGCACCACGTTTTGTGGG VCTTFCG 1

GGGACCGCGCCGCCGCCGTCC GTAPPPS 1

GGTGGGGGCAGCACGGAGGCT GGGSTEA 1

GGGCAGGCGAGTGTTGGCTGG GQASVGW 1

TTGCGCGGCAGTACTCTGGTT LRGSTLV 1

TCGCTCCGTGCGATCTCTAAT SLRAISN 1

CGTGCGGACTTGTTCCCTGCG RADLFPA 1

TCGCGTATGAGTGGCTGTGTT SRMSGCV 1

ATCCGGTATCCCATTGGCCTT IRYPIGL 1

TCGGGTTCTCCCGGTAGGCAC SGSPGRH 1

GGGTGGCAGAGTACTTGGACT GWQSTWT 1

GGCAAGCCGGGCGTTCATATT GKPGVHI 1

GGCCGCGGCTGTGCCTGTTCG GRGCACS 1

GAGGTGCACGACCAGAACATG EVHDQNM 2

TTTCGTGTGACCGGCCGGAGT FRVTGRS 1

TTTCAGGATTACAGCCAGTGT FQDYSQC 1

GGTGGGGATCGTTGGGGGCTC GGDRWGL 1

CTCGTGGGGAGGGCGCATACG LVGRAHT 1

GTCGGGGTGAACCTGTTCGTG VGVNLFV 1

CCGGTCCCGAGGGACTTGCGT PVPRDLR 1

ACTCTCTACGAGGGGTTCCCC TLYEGFP 1

TGTGGGGTGCTGGTGTGGAAT CGVLVWN 1

TCTCAGTGGTTTCTTGCGCGG SQWFLAR 1

GCGCAGTCCAAGGTGAGCGCG AQSKVSA 1

GTCAAGTGGGCTTTCCGTAGG VKWAFRR 1

TGTTGTGAGCCGGAGAAGCAG CCEPEKQ 1

CATCGTTGTTTGCGCGCTCCG HRCLRAP 1

CGGTGCCGGGGGAAGGGCCCG RCRGKGP 1

CAGTTTCCCACCTGCACGCCT QFPTCTP 1

TGCGGCGAGGTTGGCCACGCG CGEVGHA 2

TGTAAGACTCTCGGCCGGTGC CKTLGRC 1

CAGCGTAGCGGGGTTTGGGAT QRSGVWD 1

CTCGAGGGGAGGTTGCCTCGG LEGRLPR 1

GGTCTGACGTGTGTGTACGCT GLTCVYA 1

CCGGCGGAGCTCAGTGGCGCT PAELSGA 1

GGTTGGTTGCGGTATCGTGGT GWLRYRG 1

TGGAGCGTGGCCGCTGTTGCT WSVAAVA 1

AATGTGGCGTCCCGCACTTGT NVASRTC 1

GTCAGTACCGCGACGGATGGC VSTATDG 2

CAGCCGAGTCGCGGCTTTGTG QPSRGFV 2

GGGGTGCCGAAGCACGCCGTG GVPKHAV 1

ATGTACGAGGTCCGCACTCCC MYEVRTP 2

GGCGGCGAGTCGTCTTCGGCG GGESSSA 1

ACGTACGACAGTCTGGTGACT TYDSLVT 1

ATCAGGTACCCGAGTTATGCT IRYPSYA 1

AACTTCTGCGGTTGGCTTCTG NFCGWLL 1

AATAGCATCCGGCTTGGTGGG NSIRLGG 1

CACTACTCCACTAAGGGGAAG HYSTKGK 1

GTGGGCAAGGGCCGTGACATG VGKGRDM 2

TTGGTCACGACTGGCTCGCTG LVTTGSL 1

TGGATGGCGCCGGTCGGTGGG WMAPVGG 1

AATGGGTGGTCGTCGTATGAC NGWSSYD 1

TGGCCGTGTAGGACCGGGAGC WPCRTGS 1

CAGGTGTGTAGTACGACGCTG QVCSTTL 1

AACAGGTTGTTCCGGTGGCAG NRLFRWQ 1

GTCAAGGGGAACGGGATGGGT VKGNGMG 1

TCCGGTCGCCTCTACTTTCAT SGRLYFH 1

GTCCCGGTCGCGGTTGTTTGG VPVAVVW 1

GGGCTGCGGTACAGGCTGGGG GLRYRLG 1

GGCAATTGGTCTGGGGCTCTT GNWSGAL 1

GATGGCGGGACCGTGAAGGAT DGGTVKD 1

CATGTCAGCCCTTTCAGCTTT HVSPFSF 3

ACGGCGGTCAGCCGTCCGTAT TAVSRPY 2

ATGCATTGGTCGGCGGAGGTT MHWSAEV 3

CTGGATGGCTGCAACACTCCT LDGCNTP 1

ATTGCGCACTGCCTGGTCTCG IAHCLVS 1

GCGAGTGACGTCACCCCGCGT ASDVTPR 2

CACGCGGATGTGAGCTGTTAT HADVSCY 1

GTGGTGCCGGCGTGTGTTCCT VVPACVP 1

CTGGTTCGTAGGCCGCCTCCG LVRRPPP 1

ACGTTTTGTATGGTCGGCGGG TFCMVGG 1

CTGGATTTGCATCTCTCCGCT LDLHLSA 1

TTGATGCGCATTATCTATATT LMRIIYI 1

GCGTCGTGGTCTAGTTTGCTG ASWSSLL 1

AATGGGGGGCGGTGCGCGATG NGGRCAM 1

TTTGGTCGCTCGATTGATCAT FGRSIDH 1

ATGCTGTCGAGGGCCGCGCGG MLSRAAR 1

TCTCTTGGCCGGCCGGTCCTT SLGRPVL 1

GTGAGTGCGCCTGGGCATCGT VSAPGHR 2

GTGCGGTTTCCTTGGTTGCCG VRFPWLP 2

GCCAGTCAGCTGCGGGAGGGG ASQLREG 1

TCTATTCCGTGTAGTCGGGGT SIPCSRG 1

GACCGTGAGGGCGTGCAGGGT DREGVQG 1

GCGCAGTACCGCCGCTTTGGT AQYRRFG 1

TCGTGCTACCGGGAGTACTCT SCYREYS 1

CGCTCGCTTGGGGGGGCCCGG RSLGGAR 1

AAGCGGCTGCGGGTCATTGAT KRLRVID 1

GTTGCGGATCCGTTCAGCACG VADPFST 1

ACGGCGGCCAGCTTCGGCATT TAASFGI 1

CGGGTGCTCAAGGCCTCGAGT RVLKASS 1

AAGTTTGACAGCGACTTCGGG KFDSDFG 1

CGTTACTGGGGTCGCTTCGCT RYWGRFA 1

GAGGGGGACGCTGGGTATATG EGDAGYM 1

CAGGGGGTGAGGGGCCAGCCG QGVRGQP 1

CGTCGGTTGTGTTGGGGGCCT RRLCWGP 1

GTTGGTCGGTCTAGTGGCAAT VGRSSGN 1

TTGGCCGGTCTTTATGTTTGG LAGLYVW 1

GGGAGGTGCAGGATGGCGTCG GRCRMAS 1

ATGCCGTTCTCCAGCGACAAG MPFSSDK 1

TGGTTTCGGATTATTGGTTCT WFRIIGS 1

TCGAGGAACTGTTTGGCGTGT SRNCLAC 1

GTGGTTGAGGAGAGCTGGGCT VVEESWA 1

GTTAATCGGCGTGACTGTACG VNRRDCT 1

TACTATGCGGACGAGTGTATC YYADECI 2

GATGACCGCCCGGTCCTCAGT DDRPVLS 2

GTTGGCGAGAGGCCGTTGGTT VGERPLV 1

CCTCAGCCTTGTCTCGCTCGT PQPCLAR 2

AGTCTGAGCGCGTCCGGGCCG SLSASGP 1

TACTGTCGTGCCGCGAGCCAG YCRAASQ 1

ACGTGGCCCTACGTCGTGGCT TWPYVVA 1

CTCAGCAGCGAGGCCAGTTTG LSSEASL 1

AGGCGGTCCATTCGCGATTGG RRSIRDW 1

CGCGGTCGCATGTTTTGCGTT RGRMFCV 1

GGTACGGAGGCTATCGTCGCT GTEAIVA 1

TGGCCTCGTGGCAACGGCCAT WPRGNGH 1

GTCGTGGTTTCCAACGATCTG VVVSNDL 1

GGCGGTGATTGGAGGGCTCAG GGDWRAQ 1

CGGTGCATTACCTCGTGTCCT RCITSCP 1

AACAGGAGGGGGACGCGGTCT NRRGTRS 1

TGCAGCCGCGCCGTCGGGACT CSRAVGT 1

AGGGTGGCCGCGGTGGGTCGT RVAAVGR 1

TCCACTCTCAAGCCGGGCAAG STLKPGK 1

GAGGCGTTCCGCGTCCGGATG EAFRVRM 1

TCCCACGAGCCTGGCCGTGGG SHEPGRG 1

AGTCTCTCGGCTTGTACGGCT SLSACTA 1

GATCCTCTGGTCGACGGCCGT DPLVDGR 1

AGTAGTGTGGGTTTGGCCTTC SSVGLAF 1

GTTAGCTTCGCCCATCCGGGT VSFAHPG 1

GGGTACCTTAGTGATAATAAT GYLSDNN 2

CTGGTGTGGTGGCGGATGTCT LVWWRMS 1

CTTTGGCGCTTTGGGTTGATC LWRFGLI 1

TGCTCGGACTGTTGCTGCGGG CSDCCCG 1

GTGCTGGCCTACTCTCGTTTT VLAYSRF 1

CTGCTGACCAGCCGGGTGCAT LLTSRVH 1

TGGTCTCCTCGTGGGGCGAAG WSPRGAK 1

TTGCAGTCGCACCGTGTTGGG LQSHRVG 1

GTCCCCGAGTGCGGGAGGTCG VPECGRS 1

CAGTGTTGGATTCTCAGCGCG QCWILSA 1

GTCTATTTGTGTATCGTCTCT VYLCIVS 1

CGTGTTCCTGGGGCTGCTATT RVPGAAI 1

TGCGGCGCGCTTCGTCGCGGT CGALRRG 1

GTGTCCCAGATCGACCGCCAG VSQIDRQ 2

CGGCAGTCGACTGAGTGCCCC RQSTECP 1

CTTGGGGGCGAGTTCAATGCT LGGEFNA 2

TCTGTGTTTATCGGCGCGGCT SVFIGAA 1

CGCCGGCGCGCGTGGGAGACT RRRAWET 1

CAGGTGTCGCTGGGGCGGTCG QVSLGRS 1

CTCTACTCCGCCGTTGTTCCT LYSAVVP 1

ACGCCGAGTTATTATTGGTGG TPSYYWW 1

TCGGACCGGTGGCTCGAGCGT SDRWLER 2

GCGTCCCTTGGTTCCAGCCGG ASLGSSR 1

CGGAGCGGGTACGTGCTTAAC RSGYVLN 1

GTGGTGTTGCCTCCGTCTAAG VVLPPSK 1

GAGCTGAGTCAGGACGCGGGG ELSQDAG 1

TGGTCGATTCGGATTTTTGCT WSIRIFA 1

TGGCTCTCGAGTGCTATGGCT WLSSAMA 1

TCGGCCGCGAGGAGCCGTCCT SAARSRP 1

ATTCGTGCTCGGTTTCGCTAC IRARFRY 1

ATGGTGGGCATTTATTGGATT MVGIYWI 1

AATGGGAGTGGGCGGTGTACG NGSGRCT 1

AGTGCGCGCCGGGCCGGTGGT SARRAGG 1

AGGATGGTCACGGCGTGGTCT RMVTAWS 1

AAGCTGAATATGCTGCTGTGT KLNMLLC 1

GGGTCTCCGAGCTATGGGGCG GSPSYGA 1

TGGACGTTGGTGTGGATGCTT WTLVWML 1

GCCCCGGTGCGTGCTAAGGCT APVRAKA 1

TATGTCAGGGCGGCGCGCCGG YVRAARR 1

CGTGGGCTTCCTGGCTGGCGT RGLPGWR 1

GACCGTCAGAGCGACATGTCT DRQSDMS 2

GGTACGATCAAGACCACCGCT GTIKTTA 1

GGGGATCAGAATCTGCAGGGT GDQNLQG 1

GATTGGGCTTGTTCCGAGAGG DWACSER 1

TCGCTGAACTATGGCGTCCAG SLNYGVQ 2

GCGGCGGACCGGTCGCGGAAT AADRSRN 1

TCTGACGAGAGCCCGTTGTAT SDESPLY 1

TCCGAGTTTTCTCCGGCTTCT SEFSPAS 1

ATTGTTTCTCCGCCGGACCCG IVSPPDP 1

TCGGTTAGCCTGCCCATGACT SVSLPMT 1

GACTGGTGCATTACTAGTCTG DWCITSL 1

TACCAGCGTGCGAGCGTCAGG YQRASVR 1

TGGCCCGGGAGCCCTCGCTGT WPGSPRC 1

TCGAAGTGGAGCGTGTTCGGG SKWSVFG 1

CATTGGCCCGCCTGCATTCCC HWPACIP 1

CTGGGCGGTTGCGCTTGTCCG LGGCACP 1

CTGGAGATCTGCGCGGTGAGT LEICAVS 1

ATCCTGCGCGCTGGGGGTCAT ILRAGGH 1

TGTTCGCGGGAGTGGAGGCAT CSREWRH 1

TTGGACGTGGGGTGGTTGTCC LDVGWLS 1

TGTCCCGTCTGCTGCCAGAGG CPVCCQR 1

GCTTGTAGGCTGTTGAACCCG ACRLLNP 1

TGTGCGCTGAGCCTTATGTAT CALSLMY 1

GATGGTGAGGGTGGCCTGAAG DGEGGLK 1

AGTGCGATCGACTGGGGGGAG SAIDWGE 1

CGTTGTGTGGGGTTTCCGTCG RCVGFPS 1

AGGAGGGTGTTCGATCGTGTG RRVFDRV 1

CTGCTCAACACGCGTGGTCGT LLNTRGR 1

GGCGTTCCGCTTGAGGGCCGT GVPLEGR 1

TCCTACCGGTGTCTTTGGCGT SYRCLWR 1

CGTGACCTCGACGCGTCTCAT RDLDASH 1

CGCACTTTCCTCTCGGTTCAT RTFLSVH 1

TTGCGCGTGAAGAGTTTCCAT LRVKSFH 1

CTGGTGGGCTCGGACTTTGCG LVGSDFA 1

TGGCGGACCTCGCATGCGACT WRTSHAT 1

TGGTGGTCGGCGGCGGGGCTG WWSAAGL 1

CGGTACTTGAAGTTGCGTCCT RYLKLRP 1

CGGCTTCACAGTGGCGTTCGT RLHSGVR 1

CTCACCAGGTTCAGTAATTAC LTRFSNY 1

GGGCTCTCCGTGGTGTCCACT GLSVVST 1

CCCTTGCTTAACGGTGACAGG PLLNGDR 1

TCGGAGGTCACCTGGATTCTT SEVTWIL 1

TCTGTTGTCAGGAGTAATCGG SVVRSNR 1

GTCAGCGGCTCGTGGGCGTTC VSGSWAF 1

GTTTACAGTTGCGGCCAGCCG VYSCGQP 1

AACCACTGGGGCTCGATTTCT NHWGSIS 1

ATGGTTATGGTGATTGGGCTG MVMVIGL 1

GTGGTTGCCAGCGGGTGGTGT VVASGWC 1

TTCGTGCGGGTCTTCGAGGTT FVRVFEV 1

ATCGGCACGAAGGTCGTCGTG IGTKVVV 1

GTCGACTGCGGTAGTAAGTGT VDCGSKC 1

TTGCAGTTCTCTCTGTCGGGT LQFSLSG 1

ACCACGTGCACTAGGGGTACT TTCTRGT 1

CATTGCGTCGGCGCCATCTTG HCVGAIL 1

TGGAAGGGGATGGGCCTGTTT WKGMGLF 1

GAGTCGTTGTTTGGCTTGCCG ESLFGLP 1

CTCGATGGGTCTGCTGAGTTG LDGSAEL 1

TGGTTCTTTCCGTCCGTCGTT WFFPSVV 1

TTCGTTCGTAGGCTCCGGACT FVRRLRT 1

TTGCTCGTTAGGGTTGCTAAT LLVRVAN 2

TCGTTTAACGAGATCGCCGTC SFNEIAV 1

TGTGGGAGCCTGCCGAGTGTC CGSLPSV 3

GTTATTCTGGGGCTTGTTTCT VILGLVS 1

CTGTACTTGCGGTGCCCCATT LYLRCPI 1

GGGGAGGGGAAGGAGAGCGAG GEGKESE 1

GTGTGCGGCAGTCTTACGTGT VCGSLTC 1

GAGGCTCCGGTGTATGTTCCT EAPVYVP 1

AGGCTCTGGGCTTGTGTGGGC RLWACVG 2

GTTGTTATCTCCCCTGCTGGG VVISPAG 1

AGCCTGCTTGGGGTTTTGGCT SLLGVLA 1

CGCATTCCCTGTTCTTGTCCG RIPCSCP 1

GAGGCGTGTATGATCCGGCTG EACMIRL 1

GATTTTAAGGTGTTGTCCTGG DFKVLSW 2

TTTGAGATTCGGGATGCGCCT FEIRDAP 1

GGCTCCATGGGCACTTGTACT GSMGTCT 1

GCGGATGGCTGGGACATCTGC ADGWDIC 1

GCCGAGGATAGGATGCTGGCG AEDRMLA 1

GGTGGTGGGATCGGCATGTTT GGGIGMF 1

CGCTCTGTCTTGCACGGGCTG RSVLHGL 2

ATCCGGTGCTGGCGGAGCTGG IRCWRSW 1

AGGGCTTTCCGGTTCAAGTGT RAFRFKC 1

GGTCCGCGGGTGCGGTTTCGG GPRVRFR 1

AAGGAGCGCACGATCGTTCAT KERTIVH 2

ATGCAGCGCGGGCACGGGAAT MQRGHGN 1

CGTGACACTAGGGGCAAGAGG RDTRGKR 1

GCCGGGGCCGCGGTTAGTTGC AGAAVSC 1

ACGATGGCGTGGATTTTGTGG TMAWILW 1

GATTTCGAGGGGGTGTTCCTG DFEGVFL 1

GGTGGGCTTCGTCTTGTTGCT GGLRLVA 1

GAGTTTGAGACGCGCTTTTTG EFETRFL 2

CCGCGGCCGTTCGTCTTGGTG PRPFVLV 1

GTCCAGGAGGAGATGGGCATG VQEEMGM 1

GATCTGAACATGATCAGGTAT DLNMIRY 1

GCGCTTTCTAGTGTCCCCGCG ALSSVPA 1

TTGCGTGCTCAGTTCGGCCGG LRAQFGR 1

GTGCCCCGGGCGACCGTCGTT VPRATVV 1

GCGCGGCAGAAGGGTACCGAT ARQKGTD 1

AGGACTAATTCCGATCGGCGT RTNSDRR 1

AATATGCATGGGTGTGGGCTG NMHGCGL 1

GCTTGTGTGGGGGAGGGGTGT ACVGEGC 1

AGGTGCGTCAGGATGGGCGCG RCVRMGA 1

GAGACCTCCACTTCGGGGGCG ETSTSGA 1

TCGTTTTGGTCTGGGGGTTGT SFWSGGC 1

CGTCTTGAGTCGTACGATCGT RLESYDR 2

CCTCTGGCTAGCGCTCTTAAT PLASALN 2

GTGATTGGTAGTGAGTCGTAT VIGSESY 1

GATTGGCAGTTGATCCACGCG DWQLIHA 2

GGCGTTAATTGGTTGCCGCCC GVNWLPP 1

CAGTCTTGTGCGTCCTGCGGT QSCASCG 1

CGGAGCGAGCACGTTCTTCCG RSEHVLP 1

CGTCCGGGGGCGGGCTTTCGT RPGAGFR 1

GCTTTGCTTAGTTATATCTGG ALLSYIW 1

GGGCAGCTGGTTCCCCGTTGT GQLVPRC 1

GCTGATGTGATGTACTGCGCT ADVMYCA 1

GCCCGGCGTGCGCGTTCCGAT ARRARSD 1

CCCTCTCGCAGTAAGGTCTCT PSRSKVS 1

GCGAGCCGTCTTGGCTCGACG ASRLGST 1

CTGTGCACTAGGCAGCTGGAT LCTRQLD 1

TCCTACGCTCTGCCGAGTCGG SYALPSR 1

CAGGGCGCCTGGGTCCTTGGC QGAWVLG 1

TGGTGGCACCGGAAGTTGTCT WWHRKLS 1

CGCGTGTGGTGCGGTAATTCT RVWCGNS 1

TTGGGTCCTGCTCGGAGTTCG LGPARSS 1

AGTTGTGTTATTTTGTCGGAG SCVILSE 1

CAGGCTGCTGGCTTGTTCAGG QAAGLFR 1

GGGGCTGCGTGGTCGTCGAAG GAAWSSK 1

CGCCGGGAGCGTTTCAATGAG RRERFNE 1

TTTCAGGTTAGCATGAGCTTT FQVSMSF 1

CGTGTGGAGTCCTCCGTTCAG RVESSVQ 1

TTGTACGCCAGTGGCATGGGC LYASGMG 1

CGCCATTTGTTGGCGCACAAT RHLLAHN 1

GGTAGTAGCAGTCGTCACCTT GSSSRHL 1

GCGTGCTATAGGCCGGAGAGT ACYRPES 1

CACGGCTGTGCTAGGGAGAGG HGCARER 1

AGTCATACCAGGTTGTGTTAT SHTRLCY 1

ACTACCAAGCGGTCGTTGCTG TTKRSLL 1

TGGCGCCGGGTGAATCGCCTG WRRVNRL 1

GGTAGCGGGAATAGCGGCCAG GSGNSGQ 1

GTCATTGCCGTTCACGGTTGG VIAVHGW 1

CTCAACGGCGTGGCCATTGAC LNGVAID 1

CGGTCGCTGTCGGCTAGGTTT RSLSARF 4

GAGGGCGGCGGCGGTGTGGTG EGGGGVV 1

GCCTTGCATGCGCTCGGCCGC ALHALGR 1

ATTCGGAATAGTGCCCGGTTG IRNSARL 1

ACTGCTGGTCCTGATCTTTCT TAGPDLS 1

GTTGGTCGTCAGGGGCACCCG VGRQGHP 1

ACGTTGTGGAAGACGGGCCGT TLWKTGR 2

GGCGTGCGTTGCCGCCCTAAC GVRCRPN 1

TGTCGCCCGACCTGCGCGCGG CRPTCAR 1

AGTAGCCCGGTGTGGTGTTGG SSPVWCW 1

TGGGGTGTCGGCGGCCCGTGC WGVGGPC 2

GTTTTCGGGCACCGCTGTGCG VFGHRCA 1

TGCAGGCCCAGGAGTGACCCT CRPRSDP 1

GCCGTGATGCGGCCCGGTTCT AVMRPGS 1

CACTGGGTCCGTGTTGGTTCC HWVRVGS 1

AGCTTCCAGCGGAGCAGCACT SFQRSST 1

CGGGCGAAGAGCTGTTGTTGT RAKSCCC 1

AACGCCGCCAGCCGCGACTCG NAASRDS 1

TTTGTCGTGCGTGGGCCGTCG FVVRGPS 1

TGGGAGTGCAGCGGCTCCTCG WECSGSS 1

GGGCACCTCATTGGGGATAGT GHLIGDS 1

CTCTTCTACGCGTTTGTGGTG LFYAFVV 1

ACTCCGTACATCCACGGCCCT TPYIHGP 1

GGCAGGCGGACCTATCTTCTT GRRTYLL 1

TGGTGTCGGGTGCAGAGCTGT WCRVQSC 1

CGCCCCTGCGTTGCGGGCATT RPCVAGI 1

GCCCTGGGTCTGGACGTTGGT ALGLDVG 1

CAGGATGTCTCGGGGTCGGTT QDVSGSV 1

AATGAGTTCGGTAGGGTTGGG NEFGRVG 2

CCTCTCCGTATGGGGTATGCT PLRMGYA 1

CCTGTGGGGGTTGGGCAGCTG PVGVGQL 1

GTGGAGGCTCGGTGGGGTCGG VEARWGR 1

TGGTTTAGGGGGGGCCTGCCG WFRGGLP 1

TTCCAGCGCTACTGTGCTGAT FQRYCAD 1

TTGGTTGTCCGTGATGGCGCG LVVRDGA 1

TCCGACAGGCCGATCCGGACG SDRPIRT 1

TCCGTCGGTTCGTGGGCTATG SVGSWAM 1

GCGTTTCCGGTCTCTGTTGTG AFPVSVV 1

AACTTTTACCGCCTGGACATG NFYRLDM 1

AGCAATATGACCGTCAAGTGT SNMTVKC 1

TGCCCGGACGTCGGGGGGTCG CPDVGGS 1

TGTCGTTTGGTTAGCGACACC CRLVSDT 1

TTGAGTCGCCGCATGTATGGT LSRRMYG 1

AGCAGCGGTGAGGTCAACTCT SSGEVNS 1

AGGTATCGTACCAGTTGGTCT RYRTSWS 1

CTTTTGATCACGTCTCACCAG LLITSHQ 1

GTTCGCTTTAGGGTCTCTCAT VRFRVSH 2

TTTCTGCGCATGTCTGCCAGG FLRMSAR 1

CTTTACCTTAACAGGCGGGGG LYLNRRG 1

CTGCGCCGCGGGGCGGTCTCG LRRGAVS 1

CCGATTTCGATCCGGCGGATC PISIRRI 1

TGCAACATCCTGCCGGTGGCG CNILPVA 1

GATTACCATTATGTGGCCCAC DYHYVAH 2

CCGTGCTGTAGCGCTAACACT PCCSANT 1

CTGGATTTGACTGGGTTCGCG LDLTGFA 1

CGCAGTGACGTTCGCGTTTTC RSDVRVF 1

TGCTGGCAGGGGGTCCACTCG CWQGVHS 1

AACGACTATGCTCTCGGGGGT NDYALGG 1

AGTATCAGGTGTCTCCAGGAC SIRCLQD 1

CGCTGGCGGAGGGAGCTGGGC RWRRELG 1

GCTTTCGGTGCCTGTAGTGGT AFGACSG 1

CGTCTCCTCATTTGGGTTCTT RLLIWVL 2

ATGGGGCCGGTGGAGCGTCTG MGPVERL 1

TGTCTTCGTCGGGTGTGGCGG CLRRVWR 1

TTTTCCGGGTTGGCGGTGGTG FSGLAVV 1

GGGTACGTGGTTTCTCCCGCT GYVVSPA 1

ATTATCGTCTCGTGGTTTAGT IIVSWFS 1

GGCCGTCTGCGGCGGGGGGTG GRLRRGV 1

TGCGCGGTCTGGATGGTCCTG CAVWMVL 1

ACGCGGGATCGGATTGGGATT TRDRIGI 1

GTTGATGGCTTCGGGAGCCGT VDGFGSR 1

GGTTACTCCACCTTGAGCGAG GYSTLSE 1

GTCCTGTGGCTTGCGCTGCGG VLWLALR 1

GTTTGCTACAGGCGGCCGGGG VCYRRPG 1

ACCGGCCCGGCGTACTTCTTG TGPAYFL 1

TTGTTTCATGCGAGGAAGGGG LFHARKG 1

GCCCGGGTCGTGCTTCTCTTG ARVVLLL 1

CAGCCGCGTGCCTGGGCGGAT QPRAWAD 1

CGGGGCGGGGGCCGGCTGGGT RGGGRLG 1

GTCATGGCTGGTTCTCATCGG VMAGSHR 1

CTCATGCTGATGAGCCATCCG LMLMSHP 1

TCTCAGCGGGCTCGCGTGAGG SQRARVR 1

TGCTGTTTCACCTCTGGGCCT CCFTSGP 1

TGGTCTCGGCGGGCCGCTTCG WSRRAAS 1

TTGCTTTGTGTCCCCGCTTGC LLCVPAC 1

GTTTTCATGGTCGGGGTCTTT VFMVGVF 1

CACCTTTGGACGGGCCGGGGC HLWTGRG 1

GATCTGGATCTCGCTTCTGCT DLDLASA 1

CGGGCCGGGGGTTACGCCGTG RAGGYAV 1

GGGGGTCAGCCCAATTTCTGG GGQPNFW 1

TTGTCGTGCGCGCGCGTCGTT LSCARVV 1

TACGTGCCCGGTCGTGCGTTC YVPGRAF 1

TTTGATCTCGGGAGGGCGCGT FDLGRAR 1

GCTGCCATCATGTGGGGTGGT AAIMWGG 2

TTCCCCGGGGGCTCGCTTGAG FPGGSLE 1

GTCAGTTGCCGGGGGCTCGGT VSCRGLG 1

GGCATGGAGGCGTTGAACGTC GMEALNV 1

CGGGGGCATAGTTACAACGCG RGHSYNA 1

GGGTGTTCCAGGGATTCCGGT GCSRDSG 1

ATGGTGCGCGGGTGGGGCGAT MVRGWGD 2

AAGTTCCGGAGCGCGGCGGCG KFRSAAA 1

GAGCCGCGGGTCGGCTCCGAG EPRVGSE 1

GATTTTGGCGCGTGGGATCCT DFGAWDP 1

ATCGGCGCGACGCGGGTGGCT IGATRVA 1

GACTTTGTGCGCCGCCTGATT DFVRRLI 1

CGGTGCCTGCTCGGTAGTGGT RCLLGSG 1

TCGCAGTGGGTGCTTAGCCAT SQWVLSH 1

AGGGGCCAGGCGCTCTCTGAG RGQALSE 4

GGTGCCAGCGGGAGGCTTCGT GASGRLR 1

GGCCTTCGGTCTCTGCATGGG GLRSLHG 1

ATTATGTGCGCCGTGTGGTGT IMCAVWC 1

GGTTCGTTGATGCGTCTGGTG GSLMRLV 1

CAGATTGACTGGGGCGTCACT QIDWGVT 1

AGGGGCGGCTCCACGCAGCAT RGGSTQH 1

GCGCACCCGGGGCGTTTGCTT AHPGRLL 1

TACACGCTGAGGCGCGGTCAG YTLRRGQ 1

AAGGTGCTTTGGTTCAAGAGG KVLWFKR 1

AATCAGCGGTGGGGCCATCCG NQRWGHP 2

ATCTTTCCCAGTACCGGTGCT IFPSTGA 1

CGCTCGAGCTGGTGTTTGGTG RSSWCLV 1

GGTTGGCGTGCCGCTTCGGGT GWRAASG 1

GAGCGCACCTCTTGCATGTGT ERTSCMC 1

TGTTACTACGATGTCGGTACC CYYDVGT 1

GGCGAGGGGGGCGGCTGTATT GEGGGCI 1

CAGGCGTTGGAGAACTGCAGT QALENCS 1

TCTGTGCGCGGGCTGTGCCAG SVRGLCQ 1

TTGGGGAGGCGCGCCACTACT LGRRATT 1

CTGCAGATCGCTTGGTGGAAT LQIAWWN 1

TGGAGGCTCATTTTGCACAGG WRLILHR 1

TGGATGTTCTCGTGTATGCCG WMFSCMP 1

TTGGGCCGCGGTGGTCTTCCT LGRGGLP 1

CAGCGCGGCGATGGTGCCGGC QRGDGAG 1

CAGCCGTTGCGTATGCCGAGT QPLRMPS 1

GCTCGCCTCAGCGCCTGCGTT ARLSACV 1

AGTCAGTTTAGGATCTCGCGT SQFRISR 1

TTCGTGCGCTGGTTGGTTGCG FVRWLVA 1

TTCTGGCGGTGGATGTCCCGT FWRWMSR 1

CGCATTAGGAGCGGGGTGTAT RIRSGVY 1

CTGGATTTCAGCGTCGGTGAT LDFSVGD 1

AAGCGCTGTTCGACCTGGAGT KRCSTWS 1

TACCGCGATACGCGCGGTCCT YRDTRGP 1

CCCTATGATTGCAGGTGGTGG PYDCRWW 1

CTCCGTCGGTCCGGGTTTGGG LRRSGFG 1

GACAGCGGGAGTTCGCAGGGC DSGSSQG 1

GAGACCGACATGGACTACTGT ETDMDYC 1

AAGTGGGTGTGGGTGTTGCTG KWVWVLL 1

AGGCGGTGCTATTGCATCTGT RRCYCIC 1

TGTATCGACTACTGCGCGCTT CIDYCAL 2

CGTTGTCAGTGTTTGGACGTT RCQCLDV 1

GGCCGGGGGTCTGCGGGCGGG GRGSAGG 1

GCTTGGTGGCTGGTGGGTAAC AWWLVGN 1

TGGTTGGCCAGTGTTCGGCTG WLASVRL 2

GTTCGCCTCCTGTCCGCTGGG VRLLSAG 1

GTCGTTGTCTTTTTTGCTCCT VVVFFAP 1

GGTAAGTATTGCTGTGGCAAG GKYCCGK 1

GAGCGCGGGGGCCTGATGCCG ERGGLMP 5

ATTAGTGCCGTTGAGGGGACT ISAVEGT 1

GTGCGCGGCGCGTGTAGGGGC VRGACRG 1

GGGTTTGGCCGGCTTCCTGGT GFGRLPG 1

ACCTTTGCTTTGGAGCGCGCG TFALERA 1

GCTCCGGAGGACCGTTGCGGT APEDRCG 1

AGCGCTTGCGCGGCGATTAGT SACAAIS 1

GGGCAGATCCTTGGCGGGTTT GQILGGF 2

TTTAAGCGTGGGGAGAGGGCC FKRGERA 1

ATCTATACGAGGGCGGGGGTT IYTRAGV 1

GATGTTCGTTTTGACGGGTGC DVRFDGC 1

GCGCGGGGGGAGGTGCTTGCG ARGEVLA 1

GATTCTGCTCGTCACGGGTTC DSARHGF 1

TATCGTGAGTTTTGGACGTGT YREFWTC 1

TTGTGTACCGCGCCCGGCTTT LCTAPGF 2

GCGAAGCGGTACCTGTATCAT AKRYLYH 2

TCGCGGCTCTGGCTTTATGCT SRLWLYA 1

GACTGTATGTTGCCCGCGACG DCMLPAT 1

CATGTCCACAATCGGGGTCAT HVHNRGH 1

TGTAGGGATCTGAAGGTGCTG CRDLKVL 1

TTGCCGCTCCTGCAGTGTATG LPLLQCM 1

AGTGTCCTCAGGGGCGAGCGT SVLRGER 1

ACGGGTCGGATGTGGCAGGAT TGRMWQD 1

GGGCACTCGTATATCCCTGCT GHSYIPA 1

CTCGCGGAGAGGGTCGGGCCT LAERVGP 1

GGTCCCCTCGGCCGCAGGCTT GPLGRRL 1

GGGTACGTTACGTTCCAGCCT GYVTFQP 1

GTTGTTGGGAGGTTTCGGCGG VVGRFRR 1

GGCTCGGGGCGGACCGGGAAG GSGRTGK 1

GGGCGTTGGTGGCTCATCACT GRWWLIT 1

GCGCAGATCCATACCTCGCGT AQIHTSR 1

CAGGTTTTCTGCTGTTGCGGG QVFCCCG 1

CATGTGCGGGCGGGTTCTCGG HVRAGSR 1

CGGATTCAGATTGCGGTCGTT RIQIAVV 1

GAGGAGCTTGCGTTGGCTGTC EELALAV 1

ATCATCGTTGGTTCCTTGTAC IIVGSLY 1

TGGGACAGGTCGATCCTGGAG WDRSILE 1

GGGTATCGTGGCAGCTGTCTG GYRGSCL 1

CTGGAGAGGGTGCCGAACGGT LERVPNG 1

CCTCTCCCTTGTGGTGAGGTT PLPCGEV 1

AGGCCCTTTGGGTTGCCTGTT RPFGLPV 1

CGGATCTCGACCCAGGATGGT RISTQDG 1

CAGATCGCTCTGCCTTGCGGT QIALPCG 1

AGGTCGGGGCTGGTCTCGGCT RSGLVSA 1

CGTTGGGACAGCGGTACGGTT RWDSGTV 1

AGTCGGGCCGCGAGCGGGAGG SRAASGR 1

GCCGGCACTAAGGCTACGTGT AGTKATC 1

GTGCTTGGTCGCATGCTCACG VLGRMLT 1

GAGCGGCCCAGTGTGAGGCCT ERPSVRP 1

CCGGTCCTGATTCTCGGCCGT PVLILGR 1

AGTCGGGACGCGCTGCTGGCG SRDALLA 1

TGTAGGCCGAGCCATACCCGG CRPSHTR 1

AGGGCTTGTGGCGGTCTCGGT RACGGLG 1

CCGGACGTGGCGATCAGTGGT PDVAISG 1

TTCGAGAACTCGATTGTGCCT FENSIVP 1

AGGCGGTCGCTGCCCTGTCAT RRSLPCH 1

GATGGCCGCATCGGGGCTCAT DGRIGAH 1

CGGTGGGAGGTCTTGTGTTGT RWEVLCC 2

TTGTTTCGGCTTTACTGTTGT LFRLYCC 1

AAGTGCGCTTCTGAGGGCCGT KCASEGR 1

GCTGAGGGTCTGGACCGTTGT AEGLDRC 1

AGCGCCGCCCCCGTGTGTAGC SAAPVCS 1

GTGCACCTCATTGTGCGGCGT VHLIVRR 1

TCCTTTGGCGTCGAGGTGGTG SFGVEVV 1

TCGTGGCGGTCCGGCCGTTCT SWRSGRS 1

GTGACGTTCTCCAACTTCTTG VTFSNFL 1

GATGTCGTGGCGGCCTGTAGG DVVAACR 1

CTCATTTTTTGCCGGCCTTGC LIFCRPC 1

TCGAACCTCTCTTTTTCGGGC SNLSFSG 1

TCCGGGGTCTGGTGGTTTGTG SGVWWFV 1

TGGCCGCATCGTTCTAGGGGT WPHRSRG 1

TCTTCGCGGGTGCGCGGTTTG SSRVRGL 1

ATCCTTGGCCTCAAGTGTCGG ILGLKCR 1

GCTTGCGGGCTGCGCGTCGTG ACGLRVV 1

GCGCAGGATTTGGGCGCGAAG AQDLGAK 1

AATTACTTGGGTGCGTGTATG NYLGACM 1

GAGTGTGGGCGTGTTTGGAGT ECGRVWS 1

GTCGGCTGCGGCTTGGGGCTG VGCGLGL 1

CCCATGTCGCACGGCTTCCCG PMSHGFP 1

TCGGCTGGTCACTGGATGGGG SAGHWMG 4

ATGGGCTACTGTGGGTGCGGT MGYCGCG 1

ATGTTGGTGCTCTGGTTCCGG MLVLWFR 1

CTGCAGTATCGGCGCTCGTCG LQYRRSS 1

TGGCGCCACGGCGAGTTTCCC WRHGEFP 1

TCGCAGCGCCTTTGGTTGCTT SQRLWLL 1

ACGAGCTGCACCTATGCTTTT TSCTYAF 1

CACGTCCAGGTTATCGCGTGT HVQVIAC 1

GGTTGGCGGGTGTTCCGGGAT GWRVFRD 1

TGTGCGGAGTTGAGCGATTCC CAELSDS 1

GCGCTCTACATGCTCCAGTGT ALYMLQC 1

TTTAGGTCGCTCGAGGTCTTC FRSLEVF 1

GATGCTCTGGTCATCTCGGCT DALVISA 1

CACTGCTCGGGGAGTGATCAT HCSGSDH 1

AGGCGGCAGTCGGAGCGGCCG RRQSERP 1

CGGGGTAGCCGCGTGAGCCGT RGSRVSR 1

GTGGGGGCCAAGAGGATCTCG VGAKRIS 1

CATCTCAGTCTGGTCCGTGCG HLSLVRA 1

GAGAATCGGCAGTGTAGTTCG ENRQCSS 1

TATCCCGGGTGGCGGGCCGGT YPGWRAG 1

CGCCGGTGCCGTGTCGCGGGC RRCRVAG 1

CGTCGGCATGGGTGTGCGGGT RRHGCAG 1

TCCTCCGGCAGGAATGGGAGT SSGRNGS 1

GTTCCTACGAGGGTGTGGGAC VPTRVWD 1

TACGTGAGTGTCGTGGATCCC YVSVVDP 1

TGTAATGGTAGGTTGCGGCAT CNGRLRH 1

GTCCGGTTGGGTTCGTGCGTT VRLGSCV 1

TCGTGTGTTGGCGGTGGTGGT SCVGGGG 1

CTCGGCCCGCCCTGCAGGGCG LGPPCRA 1

GTTGCGGCTCTGGGCCCGGAT VAALGPD 1

GTCCATGGTGCCTGTGTTTGG VHGACVW 1

TGTAGCTCTACGACGGAGGGT CSSTTEG 1

TATCCGCTGGTTCCTGAGACT YPLVPET 1

TATAGTGTGGTGTTGGCCGCG YSVVLAA 1

GCGTGTTTCAACTTGAGGCGT ACFNLRR 1

CACCTCTACTTCAGCGATTAT HLYFSDY 1

GTGTCGCTCAGGCGTGGTCTT VSLRRGL 1

TTCGGGTGCTACTCGGCCACT FGCYSAT 1

TGGTCGAGCATCGTTTGTTGT WSSIVCC 2

TTCCCTAGCAGCTATCTCCCG FPSSYLP 1

GGTCGGTGTGTTTGGTACGTC GRCVWYV 1

GGCGAGAACAGGACGGAGTGG GENRTEW 1

CCTGTTCTCAGGAGGTCTGTG PVLRRSV 1

AGCTTTGGCGCCGGCGCTCGG SFGAGAR 1

CGTGAGTCTACTTCCCGTCGC RESTSRR 1

GGGATCATGCGGTGCGCTGGC GIMRCAG 1

GAGTGTTTCATTTCGTGCTAT ECFISCY 1

TCTCTGTATCCGCCGGCTGAT SLYPPAD 1

AGTAGTTGCTATGGTTGCCCT SSCYGCP 1

TGCCGTTCCGAGTGTGTCGTT CRSECVV 1

CACGCTCGCGTGGGGTGGCTC HARVGWL 1

CGCTGGCGCAGGGCCAATTTT RWRRANF 1

TGCTGGGACATCGAGATCGCT CWDIEIA 1

AATACTAAGGTCAGGTTTCGT NTKVRFR 1

GGGTTTGTCGTCGCGCTCTTT GFVVALF 1

GGTCTGCTGCGCGGCTCTGGG GLLRGSG 1

GGCCTGGGCTGGACTGGCTCC GLGWTGS 1

GGTTGCTCGAGTGACGACCGT GCSSDDR 2

CGGCACACTTTGAACAGCCGG RHTLNSR 1

GGGGTCTGCACCGTGTGTCGT GVCTVCR 1

GCTGAGCGCAGGGAGAATGAC AERREND 1

GCTGCTCCTTCCGCTAGGTCT AAPSARS 1

GCGCTGGCTCCGGGCCGTTGG ALAPGRW 1

AGGGTTCCTAACGTTCGCCTG RVPNVRL 2

ACCGTCTGGGAGGTGAGCATG TVWEVSM 1

GCCCGGCGCACTACGATGACT ARRTTMT 1

GAGGTGCGTAGTGAGGACCTC EVRSEDL 1

GAGAAGACTGGTGCCGTGGAT EKTGAVD 1

TTGGGCATGCAGTCGCTCTGT LGMQSLC 1

AGTCAGCTCAGTACGGATCGT SQLSTDR 1

TGGCCCCTCGCGAAGTGGGTG WPLAKWV 1

CACGGCGGGTACGGCCCTGCT HGGYGPA 1

AGTTTCCAGGGGCTTTCCTAT SFQGLSY 1

CCTATTATCTGGGATCTGGAG PIIWDLE 1

GGTGCCGGTAGTAGGGAGGGT GAGSREG 1

TGTGTCCGCCGCAGCTGGTTT CVRRSWF 1

CTGTATGGCACCAGGGTCACC LYGTRVT 1

TGCTGGTATTGTACGGTTGTC CWYCTVV 1

TATTGCGTCGGCGTTGACTTG YCVGVDL 1

AGTCCCGCGGTGTGGTTTCTT SPAVWFL 1

AGTCGTTACTGGTGCTGTGCT SRYWCCA 1

AGGCGGCCCGGGTGCGGCAGT RRPGCGS 1

GAGGTGGTGCGGTGTGGGCTT EVVRCGL 1

GAGGCCGGCGGGCGCGCTAGT EAGGRAS 1

GGTTCTAAGTCCGTGATGGTG GSKSVMV 3

TATAGTTCCCCCGCCGCTGGT YSSPAAG 1

TGCTTGCGCTGGGTGTTCGTG CLRWVFV 1

GAGGTGGTTGCCCACTTCTGC EVVAHFC 1

TTGCTGGAGACCCTGTTGCCG LLETLLP 1

GTGGATCCGGTGGTCTACCGT VDPVVYR 1

GGGTTGCGGATTAAGCAGGTT GLRIKQV 1

CGCGGGCAGCCGCTTATGTCG RGQPLMS 1

AATCTCTATCTGCGCGGTTAC NLYLRGY 1

TCGGGGCACCCCATTCTTAGG SGHPILR 1

CTGCGGTGGATCCAGCGCCGG LRWIQRR 2

TTTTTCAGGCGCGTCGTGGGT FFRRVVG 1

CAGCGGCATGGTAATCCTTTG QRHGNPL 1

ATTTTGCGGGGCCTCTTGGCT ILRGLLA 1

TTCAGTGCGCCGGCTTGGAGT FSAPAWS 1

TCTTGTCTGAGGTGGGAGGCG SCLRWEA 1

GTTCCGCTGGGGCTTGTGACC VPLGLVT 1

TGCAGTCTCTGTTGGGGGCGC CSLCWGR 2

CCGCCGGTCATGCATCCTCTC PPVMHPL 1

GGGATCGTTAAGCGGCTCCGG GIVKRLR 1

GCCCCTCCTTCCTTCGGGAAG APPSFGK 1

TTCTCGCTCATGGCGGGTGAC FSLMAGD 1

TGTGGCCTGTCGGTTTTCGGT CGLSVFG 1

CGCTCCGGCGAGGGGCATCGG RSGEGHR 1

CGTTCCCTGGATGTGGGGCTG RSLDVGL 1

GCGCATCGGGAGGCGTCGCAC AHREASH 1

GGGTGCGCCTATGTGGTCAAT GCAYVVN 2

GATTGGCGGCGGATGGCGTCG DWRRMAS 1

GCGATCGTGCGTCTTAAGGTT AIVRLKV 1

AGGTTTACGCCGCGGCCCGCT RFTPRPA 1

TGGGCTCTCACGGCTGCCACT WALTAAT 1

AGGATGAGCATCCACGGCGAG RMSIHGE 1

CTCCGGGGTCGGTCGTGCTGG LRGRSCW 1

GTTGGCAGTGCTTCGCACCGG VGSASHR 2

GCCGTTGCGTGTCACTGTAAC AVACHCN 1

GTCGTGGCCGGGGTCGGTGAT VVAGVGD 1

TTGATGAACAGTAGGATCATG LMNSRIM 1

CTGCAGATCGTGTCCGCTTGC LQIVSAC 1

GGCCTGGCTACCGTTTGGGGT GLATVWG 1

CATCGTCTGCGTGTGCCGGCG HRLRVPA 1

GTTTCGCATGGTGGCGTGGAC VSHGGVD 1

ACTCGGCGCGATCGGTCCGAT TRRDRSD 1

CAGCGCGTGGTTTGGACGAGG QRVVWTR 1

GACCCTCCCGGGGTCGAGCGT DPPGVER 1

ATCCGCTTCGTGTGGGGTTCG IRFVWGS 1

ATCCCGGTTAGCAGCGTGGCG IPVSSVA 1

CGGATCGAGCGCACTCGGACG RIERTRT 1

CGGCCCAGGGTGGGGTCCAAG RPRVGSK 1

ATGTCTGGCTCGTCTGTGTGG MSGSSVW 2

GGGAGTAGCAACGGGAGTCGT GSSNGSR 1

CGTCCGTGTCCGAGGCAGCTG RPCPRQL 1

GCTCGTCGCGCCCGCGCCACT ARRARAT 1

CCGGCCGGCATGTGGGTTTGT PAGMWVC 1

ATCTGTGTGTTGCCGGGGCGG ICVLPGR 1

GCTCCCCTGCTGAGCGGGTCG APLLSGS 1

GGCAGCTACAGTTGCGTTGCT GSYSCVA 1

ATTGGTCCGTCGTGGGCCCAG IGPSWAQ 1

TATACTTGGCGTGAGAGGAGG YTWRERR 1

GGGCCGTACTGGTGGTGGTGC GPYWWWC 1

ATCCGTAACTGCCGGCCCCGG IRNCRPR 1

TTCTGGACTATTATGCGGCTT FWTIMRL 1

CTTACTAGGAGCAGCTGTCGT LTRSSCR 1

CTCGATTTGCGGTCGTTGCTT LDLRSLL 1

TTGGTTCTCTATCGTTCTTTC LVLYRSF 1

GCTCGTGGCGGGTGGTGTCGT ARGGWCR 1

ATTGGTCAGTGCTGCGCCGCT IGQCCAA 2

TCCTCGGGGGTGGGCGCCCGG SSGVGAR 1

GCGGGTGGTCGGGCCCGCACT AGGRART 1

TTCGCTTCGCCGAGCTCCCAT FASPSSH 1

TCGCAGTTTAGTGCTCGTTGG SQFSARW 1

ACCAACAGCCGCGTGGATACT TNSRVDT 2

GCGGGGAGTACGTTCTTCTTC AGSTFFF 1

GGTGGCCGGCGGATCCGCCCG GGRRIRP 1

AAGCTCGGCGGTGGCAATGTG KLGGGNV 1

ATTCGCCGCTCGGTGGGCGCG IRRSVGA 1

TGGACGTTCGGGATCGTCTTG WTFGIVL 1

TGGGACTATTTTCACATCCCT WDYFHIP 1

TTGCGGGTGTTGTACGGTCTT LRVLYGL 1

TCCGGGAGCACTTGCGACCGT SGSTCDR 1

ACCGGTTTTACTAGTTACAGG TGFTSYR 1

GCGCGTGTGTGCGAGGTCACT ARVCEVT 1

GTGGCGGCGGGTAAGAATGTG VAAGKNV 1

AAGTGCCTTTGCATCTCGCTT KCLCISL 1

TGGCTGCTTAGTACGCAGCGG WLLSTQR 1

TTGGTGCTGCATGCCGGTCAT LVLHAGH 1

TGCGTGCTGGCCGCCCAGATG CVLAAQM 1

CACTGTAGGATGATCTCGGGG HCRMISG 1

GAGCTTCTTATGAGGGTTCAG ELLMRVQ 1

GTCAGGCGCGCCGTTTGCTTG VRRAVCL 1

GGCTGGGCTCGCTTGTCCACG GWARLST 1

GTTTGCGACATGCGGCGGCTT VCDMRRL 1

ATTTACGGCGCTGGTCTTAAG IYGAGLK 1

TCCGGCAGCGGGGGGGCGGTT SGSGGAV 1

AACGGGAGTCCGCGGCTGGCG NGSPRLA 3

GCGCGGGCCAGTTCCAGGGTT ARASSRV 1

ATGCTGGACTTTGGTGTGGAT MLDFGVD 1

GTTCACTCTACCGCGGGTGCT VHSTAGA 1

GCCCGGGCTATCGGCTCTGTG ARAIGSV 1

GGGGAGAATAGCTGTACGGTG GENSCTV 2

GGCGTGCCCGTTGCTGGGGTT GVPVAGV 1

AACGGGCATAGTTGTGCGGCT NGHSCAA 1

CGGAGGGGTTGGCAGGTTGCT RRGWQVA 1

CTCGTGTCGTATCCCCATCCG LVSYPHP 1

GGGCCCAGGCGGTGGAATTAT GPRRWNY 1

AGGCTGGAGTTTCGGCTTATG RLEFRLM 1

GTGCGCAAGATCGCTTTCTGC VRKIAFC 1

AATAGCGTGATGCGCTGGAGT NSVMRWS 1

GGTCATGTGAGGGCCTTCCTG GHVRAFL 1

GTTTCCATTGTTGGTAATCAT VSIVGNH 1

TTGGTCGTGTATACGGTCGGT LVVYTVG 1

AAGTATCTGCGGTGTTCTGGC KYLRCSG 1

AACACTGGCGGTTTGTTTTGG NTGGLFW 1

CATGTGCGGAGGACCGATCTG HVRRTDL 1

GATCGTACGGGTGGGGGGAGG DRTGGGR 1

CGTATCTCCTCGACCAAGAGT RISSTKS 1

TGCTTCCGGATGCTTTGGCTT CFRMLWL 1

CCGCACGTCAGCAGCGGGTTG PHVSSGL 2

TACGGGCAGTTCAAGAAGTGT YGQFKKC 1

TGCGGTGGCTGTATGTTGGCT CGGCMLA 1

TACAGGCGCGCTGAGCTCAGG YRRAELR 2

GGCAGGGGCTCGGGCCCGGGG GRGSGPG 1

CCGGTTGTGAACCAGTTGTCT PVVNQLS 1

TTGGTGCTTTGGTTTATGACG LVLWFMT 1

TTCTACCGTTGGCTGGCTGAG FYRWLAE 1

GTTGGCTGGTTGGTCTGCGCT VGWLVCA 1

TACAGGGCCCCTTCCGCCCGT YRAPSAR 1

GTCATCATCGGTTGTCAGTCT VIIGCQS 1

GAGTGCCACCAGGACGGTCGG ECHQDGR 1

GAGGTTCTGTACAGGCTCTGT EVLYRLC 1

CATGTTGTCAGGCGCCATAGT HVVRRHS 1

TCCCATCGTCTCATCGGTGGT SHRLIGG 2

CTGTGGCAGCTGCGGTATCGT LWQLRYR 2

TTGAACGACGGGCGGCTCTTC LNDGRLF 1

TGCAACGCGACCGAGTTGGGC CNATELG 1

GCGAGCTGGCCTACGGATTAT ASWPTDY 1

TGGAGGGCCGAGGCTAGCGCG WRAEASA 1

GCGGGCGAGATTCGCTTGAGC AGEIRLS 1

GACTGGATGTTGGATTGTGCT DWMLDCA 1

TGTCGTTTCTCCATCAGGTGG CRFSIRW 1

AAGCGTACGTCGTGTGAGGCT KRTSCEA 1

GCGACCTGTTGCTTTTTTCGC ATCCFFR 1

TATTTGCTCGTCTGGACTCAG YLLVWTQ 1

CCCGGGGGGTCTAGTCGTCGG PGGSSRR 1

GAGGGCAGGGTCCTGTGTTCG EGRVLCS 1

ATGAAGGCTGTTATCTCTGCT MKAVISA 1

GTGCGGACTGTTATTCGTAAG VRTVIRK 1

GGCTGCTACGGGCTGCATATG GCYGLHM 1

CTTGTGAGTGGGCGTGATGGG LVSGRDG 1

GGTCTTCACAATGTTGGGTAT GLHNVGY 1

GGTCACACGGCGCACCACGTG GHTAHHV 1

TGCGATCGCGCTCGGAGGCTT CDRARRL 1

TGTGCGCGGTGTAATGATCGT CARCNDR 1

AAGGTGCTCTTTGGCCCTTTG KVLFGPL 1

TTGCCGTTTGGCTTGGGTGAT LPFGLGD 1

CATGTGCATTTGTCGCTGCCG HVHLSLP 1

CGTCGTAGCCGGGTGGCTACT RRSRVAT 2

GGGTGCGATGCTGTGGTTAGT GCDAVVS 1

TGCGCGTGCCTCTGTTGGTAT CACLCWY 1

TTTGCCGGTCGTTATGGTGTG FAGRYGV 1

AACCGCCCCTCGCGGCAGCTT NRPSRQL 1

TGTGATCCTGACTGCCGTTGT CDPDCRC 1

GATCGTCGTCCCGGCGACGTC DRRPGDV 1

TTGGCGCGCTTTTTGTTCTCC LARFLFS 1

GTTATTGGCTTTCGCGAGCGC VIGFRER 1

TCTAGCGCGTCGCGGCACGAT SSASRHD 1

CCGCTGAGCTGTAGCGACTAT PLSCSDY 1

AACGCGGCTCAGATCAGCATC NAAQISI 1

GACGGCGATTTGCAGGGCGGT DGDLQGG 1

CTTCTGTCTGGTGATTCCAGT LLSGDSS 1

TTCGATCGGGGGTGGGTTGAC FDRGWVD 1

GCTGATGGCGGGAGCGTGCGG ADGGSVR 1

GGGGCTCGCTGGCTGGCTAAG GARWLAK 1

GCCTGGCGGAGTTGTCGGGGT AWRSCRG 1

CGCGTTGCTGGCGGTTCTCAG RVAGGSQ 1

GTTTCTGTGCGGTTGCGGTCT VSVRLRS 1

GCCCGGTGTTGGTCCCTGGCG ARCWSLA 1

AGGGTGGAGCGTGACTTTAGT RVERDFS 1

CACGTTTACGCTGTCGATAGT HVYAVDS 1

CTCCAGGGTAGGGATAGGCGC LQGRDRR 1

GGGCAGTCCGGTCTTTGGTGG GQSGLWW 1

CTGGGCCGCGAGGTGGGTGCT LGREVGA 1

GGCATCCGGACGTGCTGTTAT GIRTCCY 1

ATTCGGCGGGCGGCCTCCCGC IRRAASR 1

TTGTGTATCTGCGTCTTCTCG LCICVFS 1

TCGGCCTTGGGCGGCGCGGCG SALGGAA 1

GGTGTTTTCTGTCCGTGGTCG GVFCPWS 1

GTGTTTTCGGAGGGGCCGCTC VFSEGPL 1

GTGCATGGTTGTGGGCGGCGT VHGCGRR 1

CTTTATCTCGGCGCTGCGGGT LYLGAAG 1

TGCAGGCCGGCCAGCGCCGCT CRPASAA 2

ATTGATTGGAGGGGTATGGTG IDWRGMV 1

GGCGTCGCTAGTGTTTGCTGT GVASVCC 1

ATCACCGTTCTGGGGGCTCGG ITVLGAR 1

ATGGACGGTCTCTGCGTCTGT MDGLCVC 1

GGGATGGGCAAGCTTCCGGCT GMGKLPA 1

AACTCCCTCTTGCCGTTGGCT NSLLPLA 1

TTTCCTTTGGCCGTCTATTGT FPLAVYC 1

GGTAGGGGCCTGGGGCGCAAT GRGLGRN 1

GCTAGTGTGGTTGCGCTGGCG ASVVALA 1

TATAGTGAGCTCGTGGTTCCG YSELVVP 1

CGGCTGCTCCGTCAGCTCTCC RLLRQLS 1

GGGAATGTGATTTTTTGTTTG GNVIFCL 1

TGTTTGTGCAGGCCTGGGACG CLCRPGT 1

GACGGCGGGAAGAATCTTCAT DGGKNLH 1

CTTTTGCTGATTCAGAGTTGG LLLIQSW 2

CTGCTTGGCATGTGCCATGGG LLGMCHG 1

GGCTCTTGCGGTTCTGAGGCT GSCGSEA 1

GATCGGTTGTGCATTCGTTCG DRLCIRS 1

CCGCGCGGTTGTCGGGCTGGT PRGCRAG 2

GTCAATCGGTCTTGTACGATG VNRSCTM 1

CACAACAGCCAGTGCTTCATT HNSQCFI 1

TATTGGGCGGTGGCGCTGCAG YWAVALQ 2

GACCTCTGCACTGCGCAGGTG DLCTAQV 1

GTGCTTTGGGGCGCGTGGTTG VLWGAWL 1

GCGACTTTGTCTGTGAGGTCG ATLSVRS 1

CACGATTCTAGGTTGGATGGC HDSRLDG 1

CATGTGCTGGTGAGTCTGAAC HVLVSLN 1

ATGGTCCCCACTTACGTCGCG MVPTYVA 1

TGTACCTGGACGGTCGCTCCT CTWTVAP 1

ATGGTGCTGATGCTGCCGACT MVLMLPT 1

GCGGAGTTCAGGCGGTCGGTT AEFRRSV 1

GGGTGGCTCCGGCCCTGTGAT GWLRPCD 2

CCTCGTCCCGGGCCTCTGGCT PRPGPLA 1

TGTAGGTCCTCGTCCTTGGAT CRSSSLD 1

CAGATCTCCCGGTGGGCTGAG QISRWAE 1

CTTGGGCCGCTCACTCGTTGG LGPLTRW 1

GTGGCTCTTAGGGTCGCGACT VALRVAT 1

GCCGGGTCTCGCGCTGTCGTG AGSRAVV 1

AGCACGACTTCGGTCGGTCCG STTSVGP 1

CCGGACGGGAATGTTTGGCAC PDGNVWH 1

ATTCTGGAGTCGCGCGCGTGT ILESRAC 1

CTCCTGATTATTATGGGGCTC LLIIMGL 1

TCTTGCGTGCATAGGCCGACT SCVHRPT 1

CTGGGGCGTAGTCTGCGGGTT LGRSLRV 1

CACACGTGTTGTCAGTTGCCG HTCCQLP 1

CCCAGCGGGAACTTGCACCTG PSGNLHL 1

CTTGAGCTCGGGGGGCATCCG LELGGHP 1

TGTGATCGGTGCCTTCCTGTG CDRCLPV 1

GGCGAGGCGCCCGAGTTGGCG GEAPELA 1

TGGTCCCAGTCGCTGATGTTC WSQSLMF 2

GTCCTTCTCTCGGCCGTGCCT VLLSAVP 1

CCCCGGGGGAATGTCCAGCCG PRGNVQP 1

CCTGTTCTGAGCGCCCTGGGG PVLSALG 1

ACCGGGGTGCTCGGGGCGCAT TGVLGAH 1

CTTGCTGTCTCTTGTGGGCTG LAVSCGL 1

TTCAGTTGTATGGCGCGCTAT FSCMARY 1

GGGCGGTTGCTGTTCGTTAAG GRLLFVK 1

TGCGTTTGCAGCGGTTTGGAG CVCSGLE 1

GTTTTCCACAGTCAGGCTGCG VFHSQAA 1

AGCGATGGGCGGTTCGATATT SDGRFDI 1

GGGAATCCTGGCTGCGCCAGG GNPGCAR 1

AGTATTGGCATGGCGGTGTGT SIGMAVC 1

GGCATTTCTTTGTGTTATCAT GISLCYH 1

GGTACGAGGGCTGGCAGGACT GTRAGRT 1

CTTCTCTTCCCTGCGAAGTCT LLFPAKS 1

GGGCTTGTGGATCTCGCTTCG GLVDLAS 1

CTTGGTAGCGGGATTAACCGG LGSGINR 3

TCGGGTTCGTGGCTGTGTGAT SGSWLCD 2

AGTGACGTGATCGGTTGTAGC SDVIGCS 2

ATGCTGGTTGTCAGCTGCCGG MLVVSCR 1

AGGCTCGACCCTGCGTCGGGT RLDPASG 1

GTTGCCTGGCAGGCTTATTGT VAWQAYC 1

ATGCGGGAGTGCTGCTTGCTT MRECCLL 1

CTTGCCAGGCCGGGGGCTCCG LARPGAP 1

GGCTTGGCGCGGGATGGCAAT GLARDGN 1

TGCATGGCGTCGGGGGTGCAT CMASGVH 1

AGGATGGCCACGCCCGTGCAG RMATPVQ 3

TTGCTGGTTGGGAGCTGGTTG LLVGSWL 1

GACGCGATCAGTGTGGAGACG DAISVET 1

CGGAAGCTTCGGAGTAGTAAG RKLRSSK 1

GTCTACCAGATTGCGTGCCTG VYQIACL 1

ATTGGCCTGACGACCTCGGGT IGLTTSG 1

GATTGGCAGAACTTTGCCTGT DWQNFAC 1

CATTCGGGCGGCAGTAATTAT HSGGSNY 1

AGTCGGTGGCCGCTCTCGGCG SRWPLSA 1

TGGGGTCGGAGTGGCGATCTT WGRSGDL 2

CCCCAGTTGTCTAACCGTATT PQLSNRI 1

CTCGGGATTAGCGCCTGGTGT LGISAWC 1

TGGGTTTCCGCTGCGAGCAGC WVSAASS 1

GTTTTGGACCGCGGCTGGCCT VLDRGWP 1

GGTTCGAACGGGTGGGCTATG GSNGWAM 1

TCTACCGGGTTGCCTCTGCAG STGLPLQ 1

ATTCGCGGCTATGCGAGTTGT IRGYASC 1

ATGTGGGTCGGGGCGGTGACT MWVGAVT 1

AGCTTCAGTATCGACCGCGGG SFSIDRG 1

CGCCTTATGATTATGCACGCG RLMIMHA 1

ATCGGCGTTGGCGTTGCGAAG IGVGVAK 1

GCGGGGTGTCGCAAGTTCCTT AGCRKFL 1

TTGTTTCCTGGTGTTGCGGTG LFPGVAV 1

TTGTGTGTGAACGGTGTTGGG LCVNGVG 1

GGTGCGTGTGCTCTTCTCTCG GACALLS 1

CGGAGTCGCTCGGGCTCTGAT RSRSGSD 1

TTTAATGGGTATGAGGGCGGT FNGYEGG 1

TTGCTGAGCAGGCCTGTCTTG LLSRPVL 1

AGCGCGTGTCTGCGCGGTCAT SACLRGH 1

GAGGCGGTCGTCGGGAGTGAG EAVVGSE 1

ATCTCTCGCGGCCGTGCGTAC ISRGRAY 1

CCTGGTAAGTTCTGCCCTGTT PGKFCPV 1

TTGTCTCTGGTTCTCGCGCTG LSLVLAL 1

GAGCGTGGGAGGGAGGATTGG ERGREDW 1

GTTGGTTTGCCGAGTGGGGAT VGLPSGD 1

TTGCTGAGCTGTTACCCGCTT LLSCYPL 1

CGGCACTGCAGGATCTCGAGT RHCRISS 1

TTGCACTGGGGCGTCGGTAGT LHWGVGS 1

ACCCCCCGCGTCGTGATGGGT TPRVVMG 1

TACCTGCGCTCTGGCGTGTTG YLRSGVL 1

TCTGGTGTCCGCTTTGCTGTT SGVRFAV 1

GCTGGGAGGAAGCTGCAGTGT AGRKLQC 1

GGGGGTGGGGGGTTGCTGCGG GGGGLLR 1

TGCCGGTGGAGTTGGCGGCCG CRWSWRP 1

GTTGATATCGCGTTCAGGACG VDIAFRT 2

CAGTGTAGTGTGAGTTGCACG QCSVSCT 1

GTTGGCGGGGTGGTGCAGCAG VGGVVQQ 1

TCGTTTGTGGCCGTCAGCGCG SFVAVSA 1

GGGGTGCGTACTTCCGGGCTG GVRTSGL 1

GGGTTTCGCTTCGTTCACGTT GFRFVHV 2

GTGAGGGGGACTCGGGTTGCT VRGTRVA 1

TCTCGGCGTTGCGCTGAGTCT SRRCAES 1

GGGTGTCGTAAGGAGCGTATT GCRKERI 2

CGCTGGCGCGCTAACATTTGG RWRANIW 1

TATGGGCGGAACAGCCGTCGG YGRNSRR 1

GCGGAGCGTTGCAACTGGCCG AERCNWP 1

GGTGCCAGCCGCTGGTATCGT GASRWYR 1

AGCCTTCGTTTGTTGGTGGGT SLRLLVG 1

GTGGAGTGGTGGGAGTGGCAG VEWWEWQ 1

GGTATGCAGGCGATCCAGGGC GMQAIQG 1

AAGGTTTACATCGCCGCGTCG KVYIAAS 1

ACGTCTTTCGCTCCCGCGTGG TSFAPAW 1

TGTCTGTTGCCCCTGAGGCGT CLLPLRR 2

TGGCTTGAGTGGCAGGCTAAG WLEWQAK 1

CTGCTTACGGCTTGCGCTGGT LLTACAG 1

CGTGACCGGGGGCGTAGTGCT RDRGRSA 1

TCCGCTCCCGTCGTTTCTCTT SAPVVSL 2

TGCGTGGTGATCCCCAATTGG CVVIPNW 1

GCGCGTTATTGGAATCCGAGT ARYWNPS 1

CAGGAGGCCCCTGAGGGCGAC QEAPEGD 1

CTCGTGCGCAGGGGCTATCGG LVRRGYR 1

CGGCACTCGTCCAAGAAGAGG RHSSKKR 2

GAGCGCAAGGGGGACTTCGGG ERKGDFG 1

AAGGTCACCAAGGTCTGTATG KVTKVCM 3

CACCGTCATATGGATTCGGGG HRHMDSG 1

GTTTCTGTTGGCTGGACGATG VSVGWTM 1

CCTTTGCTTGACGAGGCGGCT PLLDEAA 1

CCGGCTCCGAAGGGTGGGGCG PAPKGGA 1

GACTGTATCCGGCGGAGTTAC DCIRRSY 1

CTGTCTAACAGTTTGCGCCAT LSNSLRH 1

GCGCGCCAGCTTCGGGGGCTT ARQLRGL 1

CTTATGCATCTGGCCTGCCCT LMHLACP 1

GGGGCTGGCCATGGGTGTTCG GAGHGCS 1

AGGTGCTTTTGCGCGGCCTCT RCFCAAS 2

GTCGTGCGTGGTCTCGCGTCT VVRGLAS 1

GTGTACGGTAAGGACGAGTCG VYGKDES 4

GCTGGGTCTTTGGCTGGCTTG AGSLAGL 1

GAGGAGGGCGCCGCTCCTTGT EEGAAPC 1

CATTGCCGGGGTAACCCTTGG HCRGNPW 1

CGGCGTGACTGGTTGGGGTGG RRDWLGW 1

TGGGGCCGCTCGTCCGTTGTT WGRSSVV 2

AGCTCGGCTGCGGAGGTCGTT SSAAEVV 1

CTTCGGGATGCTGTGTTGTGT LRDAVLC 2

ACGATCAGGAGCGATGGGCGG TIRSDGR 1

ATGTCGGGTCAGCGCCGGTTC MSGQRRF 1

GCGTCCTTGAGGAGGTGTCCT ASLRRCP 1

CCGGGCGTCAACAGTGGTGGG PGVNSGG 1

AGTGCCCACGTCTGCAGGCGT SAHVCRR 1

TGTCAGTCGGCCTCCATCCCT CQSASIP 1

CTCGGGAGTGAGAGCTATAGC LGSESYS 1

AGGGGGTGCTATGTCGACGGT RGCYVDG 2

TGGCTGCGTATTATTTGGAAG WLRIIWK 1

TGTACCCGCTCGAGCGTGGGC CTRSSVG 1

AGGTCGAGTCGTTGGAACTCT RSSRWNS 2

TGGTGGTTGGCTGGCGAGAGG WWLAGER 1

TGGCGCCGTGCCGTGAGGTGG WRRAVRW 1

CGGACTCCCATGGTTCTTTCG RTPMVLS 1

TGGGAGGCTGCCTGGAACGCT WEAAWNA 1

AGCCGGGACTCGCGTGAGTAT SRDSREY 1

GGTGTTGATATTGGCGACGTT GVDIGDV 1

GTTCATAGTAAGGCGGTGGCG VHSKAVA 1

GATTTGACCGTCATGTTTCAG DLTVMFQ 1

AGGCTGTTCGGTGCCGGTCTG RLFGAGL 1

GACCCCGAGGGCGTTGGCGCT DPEGVGA 1

TCTCGGTCGACCACCGCTTCT SRSTTAS 1

GCTTGCGTGAGGACCTGTTAT ACVRTCY 1

TTTCTGATCCTGGGTGGCTCT FLILGGS 1

GAGCAGGAGTCGTCCGCCTCG EQESSAS 1

CCGTGGGCGGAGACCGAGGGT PWAETEG 1

CCGACGGGTTCGTGCGAGCAT PTGSCEH 1

TGGGGGAAGAGGTTGCGGGGG WGKRLRG 1

GAGCAGGTTTGTGTCGTGCGG EQVCVVR 1

TATCGGGCTGCCGTCCCTGAT YRAAVPD 1

AGTGTTCGGCTCGCCGTTCCT SVRLAVP 2

CTGCCGCGTTGGTTGAATCGT LPRWLNR 1

GATCGTTGTGGTGGGTCGTTG DRCGGSL 1

TTGGCTACTCCGCCGCCGAGG LATPPPR 1

GACGGGCAGTTGCTCAAGTGG DGQLLKW 1

GTCTGGATCCTTGTCAGTTAT VWILVSY 1

GCTTTCTCGGGTGTCGCTTTG AFSGVAL 2

AAGTTCCTGATGTCGCTGCCT KFLMSLP 1

TCTTGTCTGCTCGCCGGTGCG SCLLAGA 1

CATGGTATGTATGCGGGCCCT HGMYAGP 1

GGTTGGGAGGATGCCCGTAGT GWEDARS 1

GGGTTGATCGTGCGTGCGGCG GLIVRAA 1

CTGACTACCGGTGTGCACTGG LTTGVHW 1

GCTACGGATGGCGGTGATTCT ATDGGDS 1

CCGAACCCTGGGCAGTACGCT PNPGQYA 1

GTCTGTGATAGCAGCGAGTAT VCDSSEY 1

ATTATGGATTCCCTGGGTAGG IMDSLGR 1

ATGCCCAGGCGCGAGGGGACT MPRREGT 4

TCGTGTGATCCGGCTTGTGTG SCDPACV 1

AAGCTGACTGCTACTATTCGT KLTATIR 1

GTTTTGGCGTGGACTCTCACG VLAWTLT 1

TACGGGGACTGGAGCCAGTGT YGDWSQC 1

GTCCCGTCGCTCTGCTGTTTT VPSLCCF 1

TGGGGGCGGCGTGTCTTGTCT WGRRVLS 1

CGCCGTGTTCGCTGCGAGGGT RRVRCEG 1

CCTCGCCGCGCCGCGATGGTC PRRAAMV 1

CAGGGCTTGTCCTTTTGGCCT QGLSFWP 1

GAGTCTCCGAGGCAGTGGCGT ESPRQWR 1

CGGGCGTTGGCGGGCTGGGGG RALAGWG 1

CATCTGGCCAACTGGGTTCGG HLANWVR 1

GAGGTGTCCATTTGGTTCAAT EVSIWFN 1

TGTTATAATCGGTCCCTTAGG CYNRSLR 1

CTGCTCCTCTCGCAGCCCATT LLLSQPI 1

GGCTGCAGGTCGAACGGCCCT GCRSNGP 1

GGGCTGGCCATCTGGTTCCGC GLAIWFR 1

GGCCGTGCGTTCGTCGCTGTC GRAFVAV 1

GCGGTTGGTGGTAGCGGGTCG AVGGSGS 2

CGGTTCTTTGACCGGGCTCGT RFFDRAR 1

GTGATGCTCTTGGGCTTTCGG VMLLGFR 1

GCTTTGTCGGACTTGCTGTTT ALSDLLF 1

CGGGCTTGTATTAGGTGCTCG RACIRCS 1

TTCTGCCGTAGGGGGCTCCCG FCRRGLP 1

GCGGGCCTCAGCGGTGGTTGT AGLSGGC 1

CGCGCTCACGGCCCTGTTCCT RAHGPVP 1

TTTTGGAGGAAGCTGAGCTGC FWRKLSC 1

GCGAAGGAGGGCCCTCGCTTT AKEGPRF 1

TTTCGCGACTCGGGTTGGACG FRDSGWT 1

GGGCTCCTCTACGGGGGGCCT GLLYGGP 1

GTTTGCTGCTGGGGGTGTTTG VCCWGCL 1

ACGTATTGGCAGGGGAGGTAT TYWQGRY 1

CGGGCGCCGAGGGAGGACGTG RAPREDV 1

TCGGCTCGCTGTCGGGCTAGG SARCRAR 1

CGGTGCCGCGTGATTACCGCG RCRVITA 1

GCGGCTTTCTTGCTCCTGGTC AAFLLLV 1

CCCGGTCGGCTGGCCCTGCGT PGRLALR 1

TTTGGGCGGAGTTGTATGTTT FGRSCMF 1

TTGAGCGTTACGGGGGCGCAT LSVTGAH 1

GGTTGTTGGTCTCCGTACTGC GCWSPYC 1

TGCAGCTCTGCCAAGTGTACG CSSAKCT 1

CATCCGGTGTTTGCTCGGGGT HPVFARG 1

AGGCAGGCGGGCCGCCGTGTG RQAGRRV 1

GCGGTCCTTGTCCCTGGGGAG AVLVPGE 1

GGCGCGGTCCGGGGCGAGTCT GAVRGES 1

GGCCTTGTGCCGGGGAGCCCT GLVPGSP 1

CTCGGTTTTCGTCAGAGCGTG LGFRQSV 1

GCCTGGAAGCGCGTGACGTTG AWKRVTL 1

TGTGTGGACGGCAGTCAGTGG CVDGSQW 1

GTTTTGACTACTGAGAGCCGG VLTTESR 1

TTCTTCGTGCGTTGGGCGCAG FFVRWAQ 1

TTGTCCCCGTTTCGCCATCGT LSPFRHR 1

GTTCGGCTGAGGCCTCAGGAG VRLRPQE 1

GCGAGGACCACTAGTTGGCCT ARTTSWP 1

CAGGGTCGGGTGGACTACAAT QGRVDYN 1

TGTCGCTGGATCAGTGGTAGT CRWISGS 1

CATGTGTGTAACGACGAGGGT HVCNDEG 1

GTTCAGCTGAGCTTGGTCGCG VQLSLVA 1

AATCTTCATTGGGCGTTGAGT NLHWALS 1

GTGTGGACCACTCGCGGCTGG VWTTRGW 1

TCCCGGGCGAGGTGGGCGGTC SRARWAV 1

ACTGCCCTGCGGAATAGGCCT TALRNRP 1

TCGACCCTGACCAGTCGGCGC STLTSRR 1

GGGCGTTTCTGGCGGGGCTCT GRFWRGS 3

GTTGCGATCGTCGGGGCGGGT VAIVGAG 1

AGCAAGAGCGTCCTGGGGACT SKSVLGT 1

GGCTTGTGGCGCCGCTCCTGT GLWRRSC 1

GGGCGGGTGTTTAGGGTTGCT GRVFRVA 1

TCGCTGCAGCGGCAGGACCGG SLQRQDR 1

GCTCGGATGGGTCGCTTCGCT ARMGRFA 1

GCGTCGTTCCTGCGCATCTGG ASFLRIW 1

GTGCGTGGGGGGGTCTGTGCT VRGGVCA 1

CGCTGTTGGTTGTGTGGCGTT RCWLCGV 1

GCGGATGCTCTTAACTTCCGT ADALNFR 1

CTTACGTGCATCGTTACGGGT LTCIVTG 1

AGGGATGAGCGTTCGTGGGAT RDERSWD 1

CGCGGCGGGGGGCCCAAGTCT RGGGPKS 1

AAGATTGGCTTGACTTTTAAT KIGLTFN 1

TGGAGGCGTTCTGGTGCTACT WRRSGAT 1

ACCGGTCTTCAGCTCGAGCTT TGLQLEL 1

TGGGCCCGTGATTCCTGTAGT WARDSCS 1

CTCACTGTTGACGTCGCGAGT LTVDVAS 1

CATTGGGATTTCGCGACGGAG HWDFATE 2

TTTCGGCACTCGGCTGTTGGC FRHSAVG 1

TGGCGTCTCGAGAAGCTCCGT WRLEKLR 3

GGGGCTTACGGCAGGTATTCT GAYGRYS 1

AGGTTGCCTTCGCAGTATAGT RLPSQYS 1

AGTCTCGGTGCTGTGCCCATT SLGAVPI 1

CCGAGTGTTGGCATGGCCTGT PSVGMAC 1

GCGGCGCGTTACTGCCGCTTC AARYCRF 1

TTTTGTAACGCCCGGCGGGGT FCNARRG 1

TACCGGGTGAACATCTTGGTT YRVNILV 1

GTGCCGCTGTGCCCCCGGTTG VPLCPRL 1

GGGCCCGGCGATACTAGCTGT GPGDTSC 1

GCCAGGGAGCTCGCGCGTCCT ARELARP 1

TGTATCGGGCTGGGCGTTCGT CIGLGVR 1

ATCTGTCCGTGGGAGGCGTTC ICPWEAF 1

GTTGGCGCCTTCGCGGGTGTT VGAFAGV 1

GGGGGTGGGGCTGTTCTCTGT GGGAVLC 1

GAGGTGTTGTGGGACCGGGTT EVLWDRV 1

CCCCGGCCGGGGTTGGTGAGT PRPGLVS 1

ATTCATCGGGTTGGGTTGGCT IHRVGLA 1

GATGCTGACCGTACCTTCTGT DADRTFC 2

TTTAGGCATCGGTATATTCCG FRHRYIP 2

AGGAGCCGGTGTAGCGGCCGT RSRCSGR 1

TCGTTGGCGACTGACAGGGCT SLATDRA 1

CGGGGCCAGGCCTCCTGGGGT RGQASWG 2

GTCTTTGGGTCTAGCGCCGCT VFGSSAA 1

TATCATTATCTCGTCGCGTAT YHYLVAY 2

TGGTGCCTTTACAAGCGTGGT WCLYKRG 1

TGTCCGACTAGGCTTCAGCGG CPTRLQR 1

TGCGTGATTATTCGGTGGTCT CVIIRWS 1

ATGCGGCGCAGCTTCTGCATG MRRSFCM 1

TTTAGTGGCCGGTTGTGTCGC FSGRLCR 1

TGTTTTGCGGGTGGCCGCGGG CFAGGRG 1

TTGACGGTTAAGAAGACGCTT LTVKKTL 1

GTTAACTCCCGTCTCATTAGG VNSRLIR 1

CCTGCTCTGGTCCGGGAGCCT PALVREP 1

AGGCTGCATATCGGGACGACT RLHIGTT 1

CGGGACCCTATCGAGGTTATG RDPIEVM 1

AGCAGTCTTAGGTGGCCTCTT SSLRWPL 1

AACCGCCGTGGCCGGCCTGCG NRRGRPA 1

TCTTCGCCCCGGGAGGACGGG SSPREDG 1

TTGCGGGCTGCGTATGTCCTG LRAAYVL 1

GTGGGCCTTTTCGTCTCGGCG VGLFVSA 1

AATGATATCCTGGGCGGCGGG NDILGGG 1

CAGGGGAATTCCGGCAGCCGT QGNSGSR 1

GGCATTCGGTCCTGTATGAGG GIRSCMR 1

GGCTTGGCGTCGTTCCATCCT GLASFHP 2

CGTCGGCCCTACAGCTCCGCG RRPYSSA 1

CGGCTTAGGTTTTGGTCGGCT RLRFWSA 1

TACAGGGGGCTGGATGACGGG YRGLDDG 1

CGTTGTTGTTACGGTAGGTGG RCCYGRW 1

TGTGCGGCGTCGTACCGGTTG CAASYRL 3

ACCTTCCAGTCTATCGCTCGT TFQSIAR 1

TACGACCGTCCGCCGGGTCTG YDRPPGL 1

GGGTGCCTGTCCCGGAGTGCT GCLSRSA 1

AGCTATCGCTGGTTCGGTTGG SYRWFGW 1

TTGTGTCATTGGGACATTCAT LCHWDIH 1

GGTACGGGGAAGGTGGCGGGT GTGKVAG 1

ATGCGTGGTCTCGCTGCTTCG MRGLAAS 1

GAGGTCGGTCCGCCCGTGTAT EVGPPVY 1

GGCCGGATGTTCTCGGGCCAG GRMFSGQ 1

GGCGATTCGGGGGTGGTCCTG GDSGVVL 1

GTCTCGCTGGGGGAGCGTGGT VSLGERG 2

TTCCGGTGGGTGCTCGCGCAG FRWVLAQ 1

GGGATGAAGCTGGGCGCCAGT GMKLGAS 1

CGGCGGGCGACGGTCTGGGAT RRATVWD 1

CAGGCTAAGGTGTTGATGTCT QAKVLMS 1

ATCTGTAGGCCCTGCGCCATG ICRPCAM 1

CGGGACGTTTGGGCGAGGTCC RDVWARS 1

TGGGCGCTCTCGGCGCGTGTT WALSARV 1

CCGCTCCTGCCGGCCGTCTTT PLLPAVF 1

AGCGAGTTCCACGTCTCGGGT SEFHVSG 1

TCCTGTCGGACTTCCAGTAGT SCRTSSS 1

CGTAGTAGGACGTCCAATTCG RSRTSNS 1

GGTCTCGTGCTCTTCACCAAC GLVLFTN 1

TCTCGCAGGGTGAGCCTCTTC SRRVSLF 1

AAGGTGCGTCTCGGGACTCTG KVRLGTL 1

TGCGCTGTGTTTTGTTTCCCT CAVFCFP 1

ACTGTTTGCGGTCTTTCGGAG TVCGLSE 2

CTTAGGGCGTTTATGTGCCCT LRAFMCP 1

CTCGTGCCTAAGTGCGAGTGG LVPKCEW 1

GCGAACCACTGGATCACTACT ANHWITT 1

GGGGCCGTGGTGAGCCTTCAG GAVVSLQ 1

TTTAGGGTGTTCTGGCTTTCT FRVFWLS 1

TCTTGCGTGTTGGCGGGTGCT SCVLAGA 1

GCGTTGGAGGGTAAGGAGTTG ALEGKEL 1

GTGAACGGCTCCAGGTGTGAT VNGSRCD 1

GTGCACAGGTCTACTGTGCGT VHRSTVR 1

GTTGCTAGCTGCCTCAGTACT VASCLST 1

AGGCCCACGGCTATGGCCATT RPTAMAI 1

ATTCGGCGCGACGTGGGCGCG IRRDVGA 1

CCTCTGGTCACCCTGAGGGTG PLVTLRV 1

GGCCTGAAGCGCGCTAGGACT GLKRART 1

CACTGGTATTTCATGTGCGGG HWYFMCG 1

GTTATCGCGTTTGGCAGTCGT VIAFGSR 1

CCGACCTGCCTGGTCCCTCGT PTCLVPR 1

CTGAGTGCTAAGCAGGGCTGT LSAKQGC 1

CAGGTTCAGTCCATCGCTGGT QVQSIAG 1

GCGATCCCGGGTGGTAGCTTT AIPGGSF 1

CATGAGCGCAGCGACGACTGT HERSDDC 1

CTTCCTAGCCTCTGGGTTCGC LPSLWVR 2

GTCGTGGGCGGGGCCTTGGCG VVGGALA 1

GGGGCGCCCAGCACCGTGCCG GAPSTVP 1

CGTCGGTGTGAGGAGGCCAGG RRCEEAR 1

GTGTCGCGGACTAGCGGCGAG VSRTSGE 1

AGCGCGATGCTGAATTATGCT SAMLNYA 1

ATGGAGCACCGCTGCGTGGTT MEHRCVV 1

AAGATCGTGAGCACTGGGTTG KIVSTGL 1

GAGGTGATCGGTGGTGGTTTG EVIGGGL 2

GGCTGTCGCTTCCGTGCCGAG GCRFRAE 1

CGCCACTGCGCCCGTGTGAAG RHCARVK 1

TGTGCTGGCGGCTGTTTCGAT CAGGCFD 1

GGTAGCGCGGGCGAGGCGCCG GSAGEAP 1

GACGTCGGCCTGGTCCGGCAG DVGLVRQ 1

GGTGCGATTAGTCGGTGTATC GAISRCI 1

GGGCGGTTCTGGCCCGCTTAC GRFWPAY 1

CGTGGGTGGCTCCTGGGTGGC RGWLLGG 1

GTTGTTTTGATGGGGTTTCGT VVLMGFR 1

GGGTGGCTGAGCCTCACGCCC GWLSLTP 1

GGTTGCGCCATTGACGGTACC GCAIDGT 1

CGCGGTCGTGTTGGTGGTCAT RGRVGGH 1

AGTTGTATGCAGCAGGTTGGT SCMQQVG 1

GTTTGGGGCTGCGTTTTTTGG VWGCVFW 1

GCGTTCACCCGCGGCGCGCGG AFTRGAR 1

CGGGCGCTCGATTTGTCGGCG RALDLSA 1

CCGTTGCAGCCGGATTGTGAT PLQPDCD 1

CGGCATCTGGATAGGGGCCGG RHLDRGR 1

CGGTCCCTCTCCTCGATCCTT RSLSSIL 1

AGGTCGTGTTGCTCGGTCAGG RSCCSVR 1

CCCACCGTCATGGCCGGCTAT PTVMAGY 1

ATGGAGTCGAACCGTGATGGT MESNRDG 1

TTGGGTGTCGGTGGTCGGCTT LGVGGRL 1

TGGCGGGCTTCCAAGGGGGGT WRASKGG 1

GACCCGGATATCGGCTGTCGT DPDIGCR 1

AGTTTGATCGGTGACAGGGTC SLIGDRV 1

CGCAGTGCCTCCACCGAGCGG RSASTER 1

CGGTTCGGGTTCTCGTGCGTG RFGFSCV 1

CGTTTCACCCGGTACTTTGCG RFTRYFA 2

AGTCGCGAGCGTGTGCGGGTG SRERVRV 1

AACCTCTGCCATTCGGAGTTT NLCHSEF 2

GTGGCTGCGGATAGCCTTTCG VAADSLS 1

CTTTACATTCGCGGTGTGTAT LYIRGVY 1

ACGTGGAGGGTTCTCATGTGG TWRVLMW 1

GCCCTGCGCATCACCGGCAGG ALRITGR 1

CTGGAGAGCAGGGCGGTGTTG LESRAVL 1

GTGAGGGGTTGGCATCCTTTG VRGWHPL 1

GCCGGTTATGTTTCGCTGGCT AGYVSLA 1

GTGGTGTTGGAGTGGGCTGAG VVLEWAE 3

TGGGAGGAGGACGGTGTGGGG WEEDGVG 1

GGGCCGCGCAAGCGGGTGCGT GPRKRVR 1

TGGGAGCTCGGGAGGCCCGAT WELGRPD 1

GATGCCAGGGAGTTCAGTATT DAREFSI 1

GGCATCGCGGCGATGACTGCT GIAAMTA 1

GCGTGGAGGCGTGCTTACTGT AWRRAYC 1

GTGGTTTGGTTGGTCTGCGGT VVWLVCG 1

GTCAGTTGCTCGGTCATGCTT VSCSVML 1

TGGGGCAAGATGGCCTCCGCT WGKMASA 1

CGGCGGGCGAGTTGTCCGTGT RRASCPC 1

TGCCTCTGGACGCGGAAGTCT CLWTRKS 1

TATAGTCCTCGCATCGGTGCG YSPRIGA 1

ATTAGGGTGAAGGTGCGTGAG IRVKVRE 1

TTCTGCTGTGGGCCCGGGACT FCCGPGT 1

GGCGGTCCTAACGGGAGGCGG GGPNGRR 2

CTCTGCTGCAGCGTGGCGTTG LCCSVAL 1

AGCGGTCGGCGTGGCAAGAAT SGRRGKN 1

GTGATCCAGGGTAGCGGCCGG VIQGSGR 1

GGCGCGCTCGGGCCCCCTGCG GALGPPA 2

GGTGTTCACCGTTGGGTGCGC GVHRWVR 1

TCCGTCCGCTTCTGGTTGGTT SVRFWLV 1

CCGCGCGAGCAGGGGCTGGCG PREQGLA 1

GGTCATCAGCCTCGCAGCGTT GHQPRSV 1

GTGCGTCGTACGGTGGTCCAT VRRTVVH 1

AGGCCTCGGCGTGTGGAGCGT RPRRVER 1

GGTGTGCAGGTGTCTTGGTCG GVQVSWS 1

GGGTGCTGCGCCGGCTCCCGG GCCAGSR 1

TCGGATGGTGCGCTCGTGTGT SDGALVC 1

TGCTTGCGCACGGTGTTCTGT CLRTVFC 2

GTGCGGCGCGGCAGGATCTGT VRRGRIC 1

GCGGCGTCCCGCTTCTGGCGC AASRFWR 1

TCCGGCGGCGTTTGGTCGGGC SGGVWSG 1

GAGGCTCGCGCGGGTCACGCT EARAGHA 1

GGGCTGGGTGCTAGGGGCTCT GLGARGS 2

AAGGTTCGCGGGTTCTTCTCG KVRGFFS 1

GTTTCTCACAGCTCCACCTCT VSHSSTS 1

TTTCAGGCCCAGTCGTTGGCT FQAQSLA 1

GTGCCTGTCTGGACTGTCCTC VPVWTVL 1

CACCGGGGCGTCTCGAGCGCT HRGVSSA 1

TCTGGTGTGCGGTGGGGGTCC SGVRWGS 1

GTTTGCGGCGGTAGCCAGATG VCGGSQM 1

TGCCGCCCCAGTTGCCTGAAC CRPSCLN 2

TCGGCGCAGCAGCGTCGTTGG SAQQRRW 1

TGTGGGTATTGCTTTATTGCT CGYCFIA 1

TCTCAGGTGCCCTCCACGTTG SQVPSTL 1

ATGCTGGGTGCCGGCCTCTAT MLGAGLY 2

TGCGGGCGCATTGATTGTGCT CGRIDCA 1

CCCGATGTCCGTCGTTACTCG PDVRRYS 1

CATTTTAGTAGGGGGGCGGTG HFSRGAV 1

TTGGGCGGCAATGGGGGCGAG LGGNGGE 1

ATCGGGCTTCGTGACTGTGGG IGLRDCG 1

CGTGCTCGTTTCAGCGACCAG RARFSDQ 3

ATGGTGTCGTGCTCTCCGGTT MVSCSPV 1

TCGCGGCGCCCGCTTGCTCCT SRRPLAP 1

AGGTGTGTTAGGCACTACGGT RCVRHYG 1

CTTCTTTCGGAGCTGATGGTG LLSELMV 2

CGTGCCTTGGCGATTACTCGG RALAITR 1

GCCCATTTGTTGGCGCTCCTG AHLLALL 1

CGTGGCGAGCACGACGAGATG RGEHDEM 2

TGGCGCTGGTTTCAGTTGCGT WRWFQLR 1

GTGCGCTTGGGCAACGGTCAT VRLGNGH 1

TGCGGGTGCTGTTGGACGCGG CGCCWTR 1

CTCGCTCGCAGTACTATGTGT LARSTMC 1

CAGCGGAACGGTAGGATTTCG QRNGRIS 1

ACCGCCGTCGCTGCGGTTCGT TAVAAVR 1

GACCTGTCGGGTATGCGGGGT DLSGMRG 2

TGGCGCGCGAGGGACCCTTGG WRARDPW 1

AGTCGGGTTGGCAGGGGGAAT SRVGRGN 1

AGCGTTTCTAGCAGGCGGAGT SVSSRRS 1

TGGGCGCTCGGTCCCAACGTG WALGPNV 1

AGTTTCTCCGAGATGCTGGGT SFSEMLG 1

TGCGCGGGTTACCGCTTGCCG CAGYRLP 1

GGTGGCGGTGCGTGGTACCAT GGGAWYH 1

CGGCGTGTCTGGGACTCGGCT RRVWDSA 1

GAGGAGCACAGCAACTGCCGT EEHSNCR 1

TCTTGTTCCACTGGTAGTCGT SCSTGSR 1

CGTGGGTGTCGTGGTCTGGCG RGCRGLA 2

TGGTGGAATTGGGACGTTAGC WWNWDVS 1

GCTAGGTGGGGGGTCGAGGGC ARWGVEG 1

AGTGGTTACTACTCCGTTAGT SGYYSVS 1

TGGGCCTGGTCTCGTAGTACC WAWSRST 1

TTCGGGACGTGTTACTTGATG FGTCYLM 1

TTGGCCGGCAGCCCCAAGGTT LAGSPKV 1

CTGAAGGTCTGCCAGGCTCGT LKVCQAR 1

GGTGGCGGGCACGGGCACTCC GGGHGHS 1

GCCTCGAGCGGTGTCGTTCCT ASSGVVP 1

CGGCGTCTCTCGTTGTTGGTT RRLSLLV 1

TTGCCTGTTTTGCGTAATCCG LPVLRNP 1

TACTGGCCGTGGATGAGCGAG YWPWMSE 2

GAGCTCTGTAGCCGTTGCTGT ELCSRCC 1

GCCTCTTCGAGTAGGAGCGGC ASSSRSG 1

GCCTGCGCGCGGTGTGCTCCG ACARCAP 1

CACTGCGTGATGGTGCACGGG HCVMVHG 1

ACCGGCACCACGGGCCTCGGT TGTTGLG 1

GGGAACTGTCGTGTGGTGTCT GNCRVVS 1

GACTTCGGTAGCTTGGTCTGG DFGSLVW 1

GGTCGTAGCGTTTGCGACGTG GRSVCDV 1

AAGGCGAACAGGGGGAGTGCT KANRGSA 1

GGGAGGAGGAGCAAGGGGGCG GRRSKGA 1

GCGCGGACTGCTGGGAGGGCG ARTAGRA 1

ATGGTGCGTCGGAGCTCGCTT MVRRSSL 1

GTGCGTCATATGTGTCGCGGT VRHMCRG 1

GGCGTGGGCAAGCACGGGAAT GVGKHGN 1

GAGGCGTCCGTTACCGACTTT EASVTDF 1

GTCCTTCGGGGTCGGACGACT VLRGRTT 1

GGTCGTACCCACGTCGGGGGT GRTHVGG 1

CCCTGGGCTATGTGGCTGGCG PWAMWLA 1

GTCGTGGGGCGTGTCGCTCCT VVGRVAP 2

GGCTGTTTCGGGCATTGCACT GCFGHCT 1

GACCAGCACAATGTCTTCCCT DQHNVFP 2

CGCGCGCGTTGTGACCTGCAT RARCDLH 1

AGCCGTGGGAGCGGGGCGCAT SRGSGAH 1

GCGGTGGTTGAGTGTTTCGCG AVVECFA 1

AGGTGCTCTGGCAAGTCTTAT RCSGKSY 1

GACTTGATCCCTATCGGTGGT DLIPIGG 1

CTGGGCGACGCGCTCAAGTGT LGDALKC 1

GTCCTCGTTAGCCTGCCGTCG VLVSLPS 1

TCCAAGAGCAGCTGGCAGGAT SKSSWQD 1

CATGCCCGGGCGAGTCTTGCT HARASLA 1

GCGTTGAGTAATCGCGTGACC ALSNRVT 1

ACGGTCCGGAGGGTGGCGGCT TVRRVAA 1

GCGAGTCTCTGCTGTTTGGCG ASLCCLA 1

ATGAGCGCCCGTGCCGATTCC MSARADS 1

CAGTGGGCGGTCTTTCTCGCT QWAVFLA 1

CTTTATTTGCGGGAGTCGGCT LYLRESA 1

TCTCGGTCCACGTGTTCTAAT SRSTCSN 1

GGTACGTATTATTCCGGTTGT GTYYSGC 1

GGGCGTAGTGAGGGGCGGCCG GRSEGRP 1

GCGCGCGTCTTGGTTCACTTT ARVLVHF 1

TGGAGGCTGTTTCTGCGGGGT WRLFLRG 1

TATATGTTGCCCTGGGGGAGT YMLPWGS 1

TTCGGTTTGAGGCTCTGGCTG FGLRLWL 1

CTGTTCTCGCCTCTCCGGAGC LFSPLRS 1

GGCTTGTACGGTGGCAGCCCG GLYGGSP 1

GGGCAGCGCGAGGGTGGGATG GQREGGM 1

AGGTTGAGCCAGGATTGCAAT RLSQDCN 3

CTCCGTTTCGATATGCCTTCT LRFDMPS 1

ACGGAGGTCTGCCTGGTTGCG TEVCLVA 1

CCTTGTCTGGTCAATGAGGTG PCLVNEV 1

GGTGAGACGAGCCGTGTTGCG GETSRVA 1

GTTAGGGGCATCGTCAGTGTT VRGIVSV 1

GGGGCTGGCGTGAGTCGGCCT GAGVSRP 1

TCGGGCTGTTTTCGGTACTGG SGCFRYW 1

GCTGCCGCGGGTGAGGTTTCG AAAGEVS 1

ATTGTGGAGAGGGCGGAGACG IVERAET 1

GGTGTGAGCGGTTTTTACGCT GVSGFYA 1

TCCTGGTTCCGCGTTATGGGG SWFRVMG 1

TTCGTTGCCGCGACGGGGCAT FVAATGH 1

TTCAGTCGCGCGCCGAAGGCT FSRAPKA 1

TGCTGGGCTTGCGGCGCCATT CWACGAI 1

TTTTCGAGTGGCTGGGCTCAC FSSGWAH 1

CGCGCGGTCGTGAGTACGCCT RAVVSTP 1

GTTTATTGTGCGGCGAGTCCG VYCAASP 1

GAGGTCTCCGCGGTGTGCGAT EVSAVCD 1

AGTGTTCTTCGGTGCTTCCGG SVLRCFR 1

GTGTGTTCTGATTTTTTCAAT VCSDFFN 1

TGGGCTCGGGCCGCGGGGTAC WARAAGY 1

TGCATGAAGCCGGTTTGCGGC CMKPVCG 1

ACGTGTGTGGGGTGCTTTGGC TCVGCFG 1

GAGCTTGGGTGCGGCCTGTGT ELGCGLC 1

GTTGGGTTGTCGCGTGCTCGG VGLSRAR 1

ATCCGGGTTAATCGGAACTGT IRVNRNC 1

GACCCCGTCTACGGGCCGCGT DPVYGPR 1

GGCGGGCGCATTGGGGCTTTG GGRIGAL 1

AGGCGGCAGGTTGTGCATTGT RRQVVHC 1

ATGTACCGCAGGCCCGTCGTT MYRRPVV 1

ACGCGTCGCGTTGTCATCTAT TRRVVIY 1

TTGTTGATCTGCAGCTGTGCT LLICSCA 1

TGCTGCGGTATTACCTATACG CCGITYT 3

GTCAGTTGCCCTCTGTTGCTT VSCPLLL 1

TCGCACAGCCCGGTCATTCCT SHSPVIP 2

TATCCTGTTCGGATCACTCTT YPVRITL 1

GCTTTGGTCGTGCAGGAGAGT ALVVQES 1

GCGAGTCTCAGTTCTTGTAGT ASLSSCS 1

AGTCGCGTTCCGGGCTGCCCT SRVPGCP 1

GAGCGTTCTACTGCGCGTTTT ERSTARF 1

CCGAGGGGCTGGGGCTATGCT PRGWGYA 1

AATGTTTACGTGGTGGTGAGT NVYVVVS 1

CCGGGTTGGAGCATTTGCAGT PGWSICS 2

GTGAGTCCCATGCGCGAGTTT VSPMREF 1

AGTCGGACTAGGAGCTTGTGG SRTRSLW 1

GGGCAGCGGGAGTTTGACACG GQREFDT 1

GGGGTCTTCTCGGGTATGTTG GVFSGML 1

GTGGAGTGCTGGGTCACTGGG VECWVTG 1

TACTGTCGGAGGTTTGTTAGG YCRRFVR 1

GGGTGCGCGATGCAGCTCGTC GCAMQLV 1

GCGCACGCGCGGCTCCCCGGG AHARLPG 1

TGGCTTAGTGCGCACACCACG WLSAHTT 1

GCGAGTAGTCGGCGCTGTAGT ASSRRCS 1

CATTGGGGTCATAGGAGCCGG HWGHRSR 3

CCCTGGGAGCGGAGTGTGGCG PWERSVA 1

CGCCCTGAGGCCTTTCATTGT RPEAFHC 1

CATATTAAGGCGTGGCAGGGG HIKAWQG 1

CCTGTCCGGAATGTTGTCGGG PVRNVVG 2

TTTTATGACACGCCTGTTTTT FYDTPVF 1

TTGTGGCGGATCTGCTTGCCG LWRICLP 1

CTGGAGACCCGCGCCTGTAGT LETRACS 1

GGGAGGGGTACCATTCGGGGC GRGTIRG 1

TACTGGTCGCGGGCCTGCCAG YWSRACQ 1

ATCGTCGTCACGGGGGACGGT IVVTGDG 1

GATGAGGGGCTTTGCCGGCCG DEGLCRP 1

GAGTTCGAGTACTCGTTTTGT EFEYSFC 1

CCTAGGCAGTTGGGGTGCTCT PRQLGCS 1

AACGAGGTGCGGGGCTGCGAT NEVRGCD 1

GTTGTCATTCTTCGGTACAGG VVILRYR 1

CGTTCTGGTAGCTTGATTTGG RSGSLIW 1

ATTCGTGTGCGGGACTTGTGT IRVRDLC 1

TCGTACCGGCTGGAGGAGCAG SYRLEEQ 1

CGTGGTGTTCGCCTGTTTTGG RGVRLFW 1

GTCCTCTGTGTGCCGAAGACG VLCVPKT 1

GTCACTCAGGCCCTGTCTGTT VTQALSV 2

TTCGGCGAGGCCGCTGAGATG FGEAAEM 1

CGGCGTGGCGGCGAGAATGGG RRGGENG 1

TCCGTGGCCGGGGGGCCGCAG SVAGGPQ 1

TGCGGTATGAATGCCGGCGCG CGMNAGA 1

CTGTACGCTTTCCTTTACTTT LYAFLYF 1

CTTCGGGGTGTGCTTGGTGAC LRGVLGD 2

GGGTCCTGGGCGAATATCTTC GSWANIF 1

GGCCCGATGGGGTGGAATACG GPMGWNT 1

CGTTGTAAGCAGGGCATTGCG RCKQGIA 1

GCTCTTGTTACCAGGTCTTTC ALVTRSF 1

CTCATGCTTGTGCTCGAGCGT LMLVLER 1

TGCGGTTCGGCCTTGCGGGTG CGSALRV 1

GCGAATTTGAGCGCCGGTGCG ANLSAGA 1

GTTGTCAAGCACGCTTTGTCG VVKHALS 1

AATCGTCATTGCATTCCGGGC NRHCIPG 1

GGGAGTAACGCTTTTACGGGC GSNAFTG 1

GTCATTGGGTTGGACAACTAC VIGLDNY 1

GCTAGCGCTCGGTCGGATTTG ASARSDL 1

GCCTCCGAGCGCCTGTTTGGT ASERLFG 1

GTTCTGTGCGTGGGGCGGATT VLCVGRI 1

GGTCGGCGCTGGACGCGGGCT GRRWTRA 1

CAGCAGCAGACTTACATGACG QQQTYMT 1

GGTTTTAGGGGCTGTCGGATG GFRGCRM 1

CTCCGCGGTTGGGGTTGGCCC LRGWGWP 1

AAGCAGTCGGGGAACGGGACT KQSGNGT 1

GTGTTCATTATGCGGCGGTGG VFIMRRW 1

CGTTTGTACCGCATTCCGGGT RLYRIPG 1

CCGCACGTGGCCGTCACGCGC PHVAVTR 2

TGGGAGCTCACCGGCGGGGGT WELTGGG 1

AGTTGGTCGACGGAGGGGGCG SWSTEGA 1

GTGGGTACGATTTGGGGTGCG VGTIWGA 1

GATGGGCTGTCTGATTCGAGT DGLSDSS 2

AGTGGTCTGGTGCCTCGCCTG SGLVPRL 1

CAGCGGCTGCTTGATCCGATT QRLLDPI 1

GAGGCGCGCAAGAGCTTCTAT EARKSFY 1

TCTGTGTGCAGGACCACGAAT SVCRTTN 1

CATGTTTTGGCTCCTCGCGGT HVLAPRG 1

GGGGGCGTCAGTACCAAGAGG GGVSTKR 1

TGCGTGTCTTTTCGTTCTACT CVSFRST 1

AAGGTGCGGACTTGGGTCGAG KVRTWVE 1

TCGGGCCTGCCGAGCGGTACG SGLPSGT 1

TGGTTTGTTGTTGGGCTGGCG WFVVGLA 1

CACCTTGTGTTGTTCCCGTCG HLVLFPS 1

GGCCCCCTGGCTTTTCGCTCT GPLAFRS 1

TGTGGTTGGGTGCATTTTTGC CGWVHFC 1

GCCAGTTGCTTTGTCGACGTG ASCFVDV 1

CGTGTGCGTTGGTGGGAGGCT RVRWWEA 1

GATTTGGAGTTGAGGGCGAGT DLELRAS 1

TGCTGGTTTGAGTGCGGGATG CWFECGM 1

GGTTGTGACTGTGATAAGACG GCDCDKT 1

TACGCCTGGCCGGGCTCTTGG YAWPGSW 1

ATGCGCCCGACGTCGGGGCCT MRPTSGP 1

GATCGGGGGATGTATCGCGAT DRGMYRD 1

TTGTGCTGGATCTGCATCACG LCWICIT 1

TGCCACGTTAATTGCCTCCCG CHVNCLP 1

GGCCGCCCTGAGAGTTGTGGG GRPESCG 1

CGTGCCCCTTGGGTCGTCGAG RAPWVVE 1

TTTCGCGACCGTGCGGGCCGG FRDRAGR 1

ATGGACCTGCTGGTCTTCAGT MDLLVFS 1

TTGCATTCTACGTTCAGGTCG LHSTFRS 1

TGCGAGCCTGGGTTTTCGGTT CEPGFSV 1

GCGTGTAGCCTGACCGCCGTG ACSLTAV 1

AAGCTCTTGATGCATAACTGT KLLMHNC 1

ATCGTCGGCAGGCGCTCGGGC IVGRRSG 1

GCTATCAGGTGTGAGTGCCAC AIRCECH 1

GTTAGCGCTAAGTTCACCTCT VSAKFTS 1

CCGGAGCGGTTCCCGGGTAGC PERFPGS 1

TACCCCATCTGCAGTGGCGTG YPICSGV 1

GCCGGTTGCTGGGCGGGCGGT AGCWAGG 1

ATGCGGCGGCACCTCCCTGCT MRRHLPA 1

TTTCAGGTCCTGTGGTCTAGG FQVLWSR 1

CCTGGGGCTACGTCCGGGCCG PGATSGP 1

TACTGTCTCTCGTGTGTGAGT YCLSCVS 1

GTTATCCGTCCGGGCTGCGGC VIRPGCG 1

GGCGGGGGGGCGCGCGTTACC GGGARVT 1

TCCGGGGAGATGGTGCGGATT SGEMVRI 1

CACAGTTCGGCGCCTAGGTGG HSSAPRW 1

CCCTATGCCGTTGACGTTCGT PYAVDVR 1

CTGTCGAGCCGCAGTCGCCTT LSSRSRL 1

GGTGTGCGGAAGATCACCCCT GVRKITP 1

TTTTACGCGGTCCGCCTCACC FYAVRLT 1

ATTCGCATTGTTGACATGAGT IRIVDMS 1

GTTTTGCTGGCTACCTATCGG VLLATYR 1

ACTGTCCAGGCCTGGCTCACT TVQAWLT 1

GATCTTGTGTGTCGGGGCTGG DLVCRGW 1

GGGTGCCAGAGTCGTCGGGTT GCQSRRV 1

GGGTCGACGGGTATGATTGCT GSTGMIA 1

TCGTGTACCTATGCGTGCACG SCTYACT 2

TCTCAGCGCTCTAGGTGTTCG SQRSRCS 1

AACGGTATTCGCGACAGTCAT NGIRDSH 1

GTTTTGGAGTCGTACGAGTTG VLESYEL 2

GTCGGGCATATGGAGGACTTG VGHMEDL 1

CTGTCCTGCGCTGCTAGGTGT LSCAARC 1

GCCCGCCAGGTTGATCGCCCG ARQVDRP 1

TCGTCGCAGGCTGTTTGCTCC SSQAVCS 1

ATGTGCGGGATGTACAGTTGT MCGMYSC 2

TGGTTGCCGCGGACTTGTTCT WLPRTCS 2

TCCCTGGTTGAGCGCTGTCTT SLVERCL 1

TTGGGTTGGCTGTCCGGGCGG LGWLSGR 1

TGGCGGTTTCTTGTGTATTGG WRFLVYW 1

TGGGACAGCCGGTGTTGGGGT WDSRCWG 1

GACGGTCTCGGGCTGAGTATC DGLGLSI 1

GCTCGCTTGTGCGGGCTCTCG ARLCGLS 1

GTGAAGGCGTTTTTGCTGCTG VKAFLLL 1

TTGTCGTTCACGTACCTGCGG LSFTYLR 1

TGGCTTCGCGGTCGGCTTTTT WLRGRLF 1

ATCCACAAGACTTGCCGTGTT IHKTCRV 1

GTGTTTCGGGTCTTCAAGGTT VFRVFKV 1

CCGGGTCGCTTGAGTGGGTCG PGRLSGS 1

TACTTCATGATTGTCCACGCT YFMIVHA 1

GCGCATGTGAATCTCGCGTCT AHVNLAS 1

GATGCTGTCACGCGGGGTCCG DAVTRGP 1

CATCTCCGCACGCCGGACCTT HLRTPDL 1

TCTGGCGATCAGCGTGTGTCC SGDQRVS 1

GCCGATGGCCCGGTGAGGGTC ADGPVRV 1

TGGGAGGTTGGCTGTAGGCTG WEVGCRL 1

TTGCTTACGGACGGCAGCCCT LLTDGSP 1

GTGTCGGGCAGTTGTGAGCTC VSGSCEL 1

GTTCTGGCTTCGGGTTTGACT VLASGLT 2

GGGTCCGTGCCGCTTTATGGT GSVPLYG 1

GGGCTTGCTTCGGTTGACCGG GLASVDR 1

TTGCCGCTTACGGGCATTGTG LPLTGIV 1

GTGGCGTTCAGCAAGGCGTTT VAFSKAF 2

GACGTTACCCCGCGCGGCATT DVTPRGI 1

GCGAGCGTCATGCTCGTCTGT ASVMLVC 1

CGCGGTATCGGCGAGAGGGCG RGIGERA 1

GGCCTGTTGGTCGACATCGTG GLLVDIV 1

TATGCTGGCGGGAGCAAGATG YAGGSKM 1

AGCCCGAACACGCTCGGGTTG SPNTLGL 1

AGTCGGAGTGAGTGGTGGCAT SRSEWWH 1

TTGCGTATGACTTGGTCGGTT LRMTWSV 1

GAGGGGCCTACGGGTTTCCCG EGPTGFP 1

CTTTTGAATAACGTGGACGTT LLNNVDV 1

CGGTCTCGCGCCAGGCCGGTG RSRARPV 1

TATAGGTACCAGACGAATCTG YRYQTNL 1

GGGCGTGATCGGGCTGCGACT GRDRAAT 1

TGTTTGATTGCGGTGAGCCGG CLIAVSR 1

GGTGCGTTTAACTGGGAGAAG GAFNWEK 1

GCTCGCCACGGGTCTGTCATG ARHGSVM 1

TTCAAGTGTATTTGTCCTCGG FKCICPR 1

TACTGCGGTATCTGGTGCACT YCGIWCT 1

GTCAGTCCGAAGGGTAGTAGG VSPKGSR 1

AGTTTTATCTGGGGCGTGCGG SFIWGVR 1

AAGGGGCGTGGCCTGGGTCCG KGRGLGP 2

GGCGGTCGCAGGCATCCGGTT GGRRHPV 1

TCTTGTGTGGTGGTGGTCGCG SCVVVVA 1

CACCTCAGCGGCCTGGGTTGT HLSGLGC 1

TTGGGCACTAAGATTAGCGCT LGTKISA 1

AAGTGGATCTTGACCGGCCGT KWILTGR 1

GGGGGCGGGGGCAGGGATCGG GGGGRDR 1

CTCGAGGGTACGTGTCACGCT LEGTCHA 1

AGGCCGGGTGGTATTAGTTGG RPGGISW 1

GTGGCGGGTAGTTACCGCGGG VAGSYRG 1

GAGAGCGCCGACTTGCTTTCT ESADLLS 1

GAGGTTCGGGGTTGCGCTCTT EVRGCAL 1

TGCTGGCGTAGCTGCTCGGGG CWRSCSG 1

GAGCTGCGCAGGTTCAGCCTT ELRRFSL 1

CACAGGTCTCTTCTCGTGCGC HRSLLVR 1

ACGCCCCATTGGGCTAGGGTG TPHWARV 1

CACGCGCGCGGCGCCGCTATT HARGAAI 1

GTTGCTGGCGGCGGGCCGTTT VAGGGPF 1

TGGTCCTGGCGTTCCAATTTT WSWRSNF 1

CTCCGGGGCCGGTGCAATTCT LRGRCNS 1

TCTCGCAGTCGGTTGAATCGG SRSRLNR 1

CGTAGGAGCGGCGGGATGAAG RRSGGMK 1

TCGAGGGTGGTCGCGTGTGCG SRVVACA 1

GTGGGTCTGGCCATCGTCTTT VGLAIVF 1

CGGTTGATGCTCACTACTCGT RLMLTTR 1

CACTCGGATCGCGGTCCGGAT HSDRGPD 1

CCGGTGGAGGAGTCGTACTTC PVEESYF 1

GAGGTCGGCGCCAGCAGTGAT EVGASSD 1

TCGGATGTGAAGACCGATCCG SDVKTDP 1

CCCAATAAGGCGGCTAGTTCT PNKAASS 1

GCGGGCTTTGGTACGCGTCTC AGFGTRL 1

GTTTTTAGTTTTTCCTGTCTG VFSFSCL 1

CAGAGCTCGCTGCCGGCTTCT QSSLPAS 1

TCTGGGTGCAGGCTCGCGAGT SGCRLAS 1

CGCTTTGGGCACGGCAGCGGG RFGHGSG 1

AGGGGTCCGTGTCGCGACCCG RGPCRDP 1

AATTCGACTCGGCGTGGGCAG NSTRRGQ 1

GGGTGCGTCTGCAGCTTTACC GCVCSFT 1

GGTCTCAATGAGCGGTCCGCG GLNERSA 1

GAGGAGGCCGCCTGTGGTGTG EEAACGV 1

TGGAGCGGCTCCATTGGTCCT WSGSIGP 1

GCTCTCGTCACTCGGCGGGCG ALVTRRA 1

GCGGAGCAGGCTTGGGTCCTG AEQAWVL 1

GGCTGTGGGATGTACTTCAGT GCGMYFS 1

TGGCTGCGCGGCTGCTGCATT WLRGCCI 1

AGTGCCGTCACTTGTGGGGTG SAVTCGV 2

ACGGCCGGGCAGCTCAGGCGT TAGQLRR 1

CTGTGGGTTCCGGTGTATACT LWVPVYT 1

CCGGCCTTGGGGTACTTTCCT PALGYFP 1

GCGGAGTCGGGGAGCCGGCGT AESGSRR 1

CCTGTCTTCGCGGCCCTTGCG PVFAALA 1

CTTGCTAAGGTGCATGTGGGT LAKVHVG 1

AAGTCGGCGCGGAGGTACGTG KSARRYV 1

TCCAAGCTGTGGTGTGTGAGC SKLWCVS 1

GGGAGGCCCATGGTCGCGTGG GRPMVAW 1

TTTTGGTCGGCGGGTCAGCGG FWSAGQR 1

TTGGGCTCCGCGTGGCGTCTT LGSAWRL 1

CCGGCGGTCAGTTCCAAGACT PAVSSKT 1

TGTCATCCCGATGTGGGCGGT CHPDVGG 2

CTTGTTAACTCTCGCCTTGCT LVNSRLA 1

CGGGTGTTGGGGATGAGTGCT RVLGMSA 1

GGGTTTGCCGATGATCGTGGG GFADDRG 2

TTGTTGACCAGTGAGCTCTCG LLTSELS 1

TCGATCCTTAACGGCAGGTGT SILNGRC 1

TGGGTGGCGGTTGGCAGCGTT WVAVGSV 1

TCTTCCGGGACTTTGCCTTCT SSGTLPS 1

ACTTTGATCGGTGGTGAGAGC TLIGGES 1

GCCTTTTTTTCGGGGAATCGG AFFSGNR 1

CCTTTTGGCCTGGTCGGTGTG PFGLVGV 2

TGGTGCTTTGAGGACAGCCGT WCFEDSR 1

GCTGTTCCCGAGCTGCGTAGT AVPELRS 1

TGGGCTAGGGTGGTCGTGACT WARVVVT 1

GTTTGCGTGGCCCGGTATAGG VCVARYR 1

TCGTCGCATATTCTTTGTCGG SSHILCR 1

AGGTTGTACGGGGACGCGGGT RLYGDAG 1

GCCGAGTCGTGCCCTTTTCGC AESCPFR 4

TTCCCTTACTGGGGGTTGCGC FPYWGLR 1

CGGCAGGGCTTCAACTTTCCG RQGFNFP 1

CTTTTTCGCTTTTACCGCTTG LFRFYRL 1

TTTGGTCGGAGCGAGGCCGTT FGRSEAV 1

GTTTGTGATTGCTGCGGCTTG VCDCCGL 2

TTCCGCGTTAGCCCGGGGCAC FRVSPGH 1

TCCAATGCGTGGAGCCGGACG SNAWSRT 1

AGGGACGCGTCTTGCACGTGT RDASCTC 1

TGTTGCACTTTTAGGATGGGT CCTFRMG 1

TGCTTCTGGTTTCTTTGCCAC CFWFLCH 1

GGGTCTCAGAACGCTTTTGTT GSQNAFV 1

GATTTGGTCTGTTTCGGTTGT DLVCFGC 1

TTGGGGGCTATTCACGGTCTT LGAIHGL 1

GCTGGTAACACGAGTCTGCGT AGNTSLR 1

GGGAAGGTCGTGGGTGTCGAG GKVVGVE 1

GCGCTCCTCCGGGGTTGTGCT ALLRGCA 3

CTCCTGTGGTCCTCGGCGTCT LLWSSAS 1

GCGCCTCGCGGCGGCAGTGGC APRGGSG 1

TTTCTCCGGATCCGCGGTGCG FLRIRGA 1

TTTGGTTTCTTCGGCGGGCCT FGFFGGP 1

TTCAAGTGCTTCTTGTCTTTT FKCFLSF 1

CTTGCGGCGTGTTGCGGGACG LAACCGT 1

ATCTGTAGCGATGCGTGCCGG ICSDACR 1

CGGCTCTGTTCGTTGGATTGG RLCSLDW 1

TGCACTTGGCGGGTCGATCTG CTWRVDL 1

TTGGTCATGCTTGGGATTCCT LVMLGIP 1

AGGGGGGCCCTCGTGCATATT RGALVHI 1

GTGGGCGCGCTCGTCGCTTAC VGALVAY 1

GCCACGCGCCGTCTTAAGTAT ATRRLKY 1

AAGTGGCGCAACGTCTATTTG KWRNVYL 1

CGCCGCCAGCCGCGCGGGGGT RRQPRGG 2

CGGGATTCCGGGTCGAATTTC RDSGSNF 1

TTCGGTCTTTCTCAGGCGAAG FGLSQAK 1

TCGATTAGTTCCGCGCGGGTG SISSARV 1

GGCAGGTGGACGACGGGTATT GRWTTGI 2

GCGGGTCCGTGCGACATGCAT AGPCDMH 2

GGGGTTCTCCGGGGGACTGTG GVLRGTV 1

TTGGTGGAGAGGTTTGGCGGT LVERFGG 1

TGGGGGGTCGAGGGGGCGGGT WGVEGAG 1

ATTTTGGACAGCAGGTGCGGT ILDSRCG 1

GTCACTCGGACGGCTCTCGGT VTRTALG 1

CTCTGGGGTAATATGATGTTT LWGNMMF 1

GGGTGCTATAGGTGTTGGCCT GCYRCWP 1

TCCTACGCCCGGTTGGATTCG SYARLDS 1

TGGCTTGGTAATGCGGCGTCG WLGNAAS 1

TGTCGCGCTGGGTGGCATGCG CRAGWHA 1

CGGAGCCACTCCGTGGAGGTG RSHSVEV 2

TGGTGTCCCCGTGTGTTGACG WCPRVLT 1

GCGGGTCTGAGCGCGGACGGT AGLSADG 1

AACTCTGGCAGGCCTCGCCTG NSGRPRL 2

ATGGCCCTCCCGCAGGGTGCT MALPQGA 1

GGTGGCCTCAGGCCTCGCCTC GGLRPRL 1

GACGCTTTGACGTGCGATTTG DALTCDL 1

GGGCGGCAGGGGTGGTGCATT GRQGWCI 1

TGCGAGCTCGGTAAGGTGAGT CELGKVS 1

TCCAAGAGGTGTGTGGTGGCG SKRCVVA 1

CTCTGGAATCTCTCCCCGAGT LWNLSPS 1

TCCCCGTTGTCCCCCGTCGGT SPLSPVG 1

CGCATCCTGAGTGCGAGTTAT RILSASY 1

CTCTCGTGCTTCTGTCCTCAT LSCFCPH 1

CAGTTGGTCTCGAGCGCTTGC QLVSSAC 1

CGGCGGGTCGATTGGGTTTCG RRVDWVS 3

GGTGTCAGTAAGCGTGCGCTG GVSKRAL 1

GCCCGTCAGTTGGCCGTGCTT ARQLAVL 1

TGTCGTGCTTCTGGCCAGTTT CRASGQF 1

TATTTTGCCAGCGCCAGGCGG YFASARR 1

CACTTCGTTTCGACCTTGGCG HFVSTLA 1

ATGGTCGGCGTCCCGGCTGTT MVGVPAV 1

ACCTCGGATTGCGGCTTTGCG TSDCGFA 1

GCGGCGGTCTCGGGCGCTGTT AAVSGAV 1

GGTCAGAAGTGGCGGGTGGAT GQKWRVD 1

TTTAGGCTGCCGAAGTATGGG FRLPKYG 1

ATCTCGCAGGACGCTGGGCGG ISQDAGR 1

CATGGTACGCCTACCGGCAGT HGTPTGS 1

ACTTGGGGGTCGGGCCGCGGG TWGSGRG 1

AGGGGCGGGACGCGGGGTAGG RGGTRGR 1

CTTTGGTCTCGCCTGGTGTTT LWSRLVF 1

CGCTCGTGCAGCGGTTGGAGG RSCSGWR 1

TGTGTGTTTGCCCAGTACGGT CVFAQYG 1

AGTAGTCTCCGCACGGGGCTG SSLRTGL 1

GGCTCTTGGCTGTTGGGCGCG GSWLLGA 1

AGGCTTCTTAAGCGGTGGTGG RLLKRWW 1

GAGGTCAGGGTTCCGAGTACT EVRVPST 1

TTCGGTGTGCTGCGGTTCCGT FGVLRFR 1

TGCGGTGATTGTCGTGCTGGT CGDCRAG 1

GATTGCCCCATGGTGAGTCGG DCPMVSR 1

GAGCCTTCGAGGACTCGGCTT EPSRTRL 1

TCCGATGTTGCGTTGGTCAGG SDVALVR 1

GTTCTCGGGCCTCCGGCCGCG VLGPPAA 1

CGGCGGGTTCGCCTCCCGATG RRVRLPM 1

TATTCCAGTACGGTCCCGGAT YSSTVPD 1

CCGTTGGGCTTGGTGCCCTAT PLGLVPY 1

CGGGCCGCCCATTCGGAGAGC RAAHSES 1

TCTTCGGGTGCCGTTTCGGGT SSGAVSG 1

GTGGAGTGTTGTCAGCTGGAC VECCQLD 1

CGGTTGAGCGCGGGCCTCTCG RLSAGLS 1

TTTTTGGCTCTCCGCGCCGCT FLALRAA 1

CGGCGTTGCGTTAAGGCCCTT RRCVKAL 1

TCTCGCGTCAAGTGGGGCTTT SRVKWGF 1

GACCACTGTTGGTATTGTCCT DHCWYCP 1

GCTGATTTTCGGCGCTGGCTG ADFRRWL 1

GGGATTTATAGGTCTGTTACT GIYRSVT 1

ATCCACCACATGGATCTGGAG IHHMDLE 1

GCGGCTCCGACTGCCAGGAGG AAPTARR 1

AGTCCCTTTTTGAGGTATAGT SPFLRYS 1

GGCCACCCCTTCGCCCCTGCG GHPFAPA 1

CAGGGTATTGCCCTCGGCGGT QGIALGG 1

GTGGAGGATCGCACCGGTTTG VEDRTGL 1

GCGGTGCCCCCTCGTGGGCGC AVPPRGR 4

GTGTTGGCCGGCTGGAGCGGG VLAGWSG 1

CTGGTCATCTCGGTCGTGGGT LVISVVG 1

GCGGGCCATAGCTGGGGCCAG AGHSWGQ 1

AGCGGTACGTCGTTCACGTCT SGTSFTS 1

TTGGCGGACAGGACCAACGCG LADRTNA 1

GTGGGGCGGATGGTTAGCGGT VGRMVSG 1

TTCTCTGGCGCGGGCTTGCGG FSGAGLR 1

TCGAGCGCGGCCACCTACAGG SSAATYR 1

GAGGGTGAGGAGCGGTTGTCT EGEERLS 1

GTGGGTCGTAGCCGGTGGGGC VGRSRWG 1

TGCGGCTACGAGCTGGACGAT CGYELDD 1

TATATTGTTTGGTCCGGGCGT YIVWSGR 2

AGCCGTTTGGGTCACGTCCCT SRLGHVP 1

CGCGACATTACCGTGGGCGCG RDITVGA 1

AACAGTTATTGGTGTCACGCG NSYWCHA 1

GGGCGGCCTTTGTTCGACCCT GRPLFDP 1

GTGTGTCGGAGGGAGTGGTCT VCRREWS 1

CCGCGGGCTCGCGAGTTGCCT PRARELP 1

GATGGTGGCCGGTGCAAGTTG DGGRCKL 1

GAGTTTCTTGCCCATGAGTGG EFLAHEW 1

GCCGGGCGGTGCGGTCCTAAG AGRCGPK 1

GCGTTTCAGCTTTTCCGTAGT AFQLFRS 1

TTCTGCCCGGGGTACCTCGCT FCPGYLA 1

AGCTACGGCGCGCGCGGGACG SYGARGT 1

CGGGCTTACAGGCGTGCTTCG RAYRRAS 1

CGGGTGACGGGGCGCGCGAGG RVTGRAR 1

TGGCGGGGGAACAATCTTGCG WRGNNLA 1

GGTTGGCGCGAGTGGTGTCGT GWREWCR 1

TGGACCTTGGCTGACGAGGTT WTLADEV 1

CTCGCCGCTCTCGAGTTGTGT LAALELC 1

CATTTGTCGCGTGGCTGTTAT HLSRGCY 1

GTTGCGTGGATGCTCTCCCGG VAWMLSR 1

GCCTGCATTTCCGGGAGGCTT ACISGRL 1

ACTCAGCAGTGCATCTTTCCT TQQCIFP 1

ACGGCGGGCACCGGCTACGAG TAGTGYE 1

CCTGACTCGTGCTGGACGGGT PDSCWTG 1

TCGGATTTTATTACCAGTCTT SDFITSL 1

GTCTACTCCTGGTTTTGGGAG VYSWFWE 1

GTGCGGCACTCTACCCCTACT VRHSTPT 1

TGTCTGCGGAACGGCCGCCAG CLRNGRQ 1

CGTAGTCTGGATGTTATGGTC RSLDVMV 1

TCGTTCAGCTGTCTTTTCCCT SFSCLFP 1

TCTGGGGGTGTTCGCGTGGCT SGGVRVA 1

AATGTTAGCGGGCCGGTGCGG NVSGPVR 1

TACTCGCATCGGTATGTGTGG YSHRYVW 1

TTGGTGTTCCGCCGCCATCCG LVFRRHP 1

GGTGCTCGCAAGGCTCGGCCT GARKARP 1

CGGGCGGTGATCCGTGTCTGC RAVIRVC 1

TTCCAGTTTCGTCTCTGCGTC FQFRLCV 1

AGCAATATGGGGGCTAGGTTC SNMGARF 1

GTGAGCTATGCCCACGGCCTC VSYAHGL 1

CATTGTGAGCGGCACCCGACT HCERHPT 1

CTGCTCAGTGCGGGTGTTCCT LLSAGVP 1

CGCCTTTGCATGGGTTACGGC RLCMGYG 1

GGTTTTCTCCATATCCGGGTG GFLHIRV 1

TATAGGCAGCTCTTCTCGCAG YRQLFSQ 1

GCGGGGTTTGGTATCGGCGCT AGFGIGA 3

GTGGTCCACAGGAGCATTCGG VVHRSIR 1

GCTTTTACCCTTATCACCTGC AFTLITC 1

GTTGGGGGTTTGGACCTGATC VGGLDLI 1

TGGCTCTGGAAGTGGTGGTGT WLWKWWC 1

CCTCATTTTAGGCGTCACGTG PHFRRHV 1

CGGTGTTCGTATTGCAAGGCC RCSYCKA 1

TGCGTTGGGATGAATCGTCCT CVGMNRP 1

ACTCGGGTGGGTGTCGTGCCG TRVGVVP 1

GGTCGGACTTATCCCCAGTCT GRTYPQS 2

GAGGTCAAGATGGAGGGTTGG EVKMEGW 1

AATGGTGGGATTTCCCATTCG NGGISHS 1

GCCCACAGTGGCAGCGGTATG AHSGSGM 2

ATCCATTTCCGGTTGAGGCAT IHFRLRH 1

CACCGGCGCGCGTGTGCCCGT HRRACAR 1

GGTGGGGACAGCGCTGTCGGT GGDSAVG 1

CGTGGGCGGACGCCGTATGGT RGRTPYG 1

CGGTACTGTTGTGAGGGGAGG RYCCEGR 1

CGGAGGACGGTGTATTGGCCT RRTVYWP 1

TATTCGCCGGGCTGGGTGGTG YSPGWVV 1

CGCCCCGCTAACTTGGGTTGT RPANLGC 1

CGTGGCAGCGTTCCCGAGGCT RGSVPEA 1

AGGTCTTGCACTTGCGGCCCG RSCTCGP 1

TCCGTCGTCCCCTGGCTGTGT SVVPWLC 1

ACTTCGCGGTGCTTGCGCTGC TSRCLRC 1

CCTACTCGGGGGCCGATGCGC PTRGPMR 1

CTCGGTTCTAGGTGCGCTCCT LGSRCAP 1

GCGTTGCCCTGGTATGCTCTT ALPWYAL 1

TATGTGGTGGCTCGGGTGCGC YVVARVR 1

TTCATGCTGCCGGTGGGTAGT FMLPVGS 1

ATTGTGCGGCTGACCGGGTAT IVRLTGY 1

GCGGTGAGTTTCACTTTTGTG AVSFTFV 1

TTGATGGGTCAGGCTAGCGCT LMGQASA 1

GACGGCGAGCGTCACAATCGT DGERHNR 1

TCTACCGACAGGGTCGTGGTT STDRVVV 1

CGGTCTAATAGGGGGCTCGGT RSNRGLG 1

TATGTGCTGAGCAGGCCCGCT YVLSRPA 1

GAGAGCGGCACGGTGGTGCAT ESGTVVH 1

CTGGTGTGGAATAAGAGCGAG LVWNKSE 1

AGGCTTGGCGTGGCGTTCAGC RLGVAFS 1

ATCGTCGTTAACTTTGTCCGT IVVNFVR 1

TGCGGGTGCCGGGAGTTGATC CGCRELI 2

GCTCAGGGTTGTTTCCAGCGG AQGCFQR 1

TATGTCCTCTGTATCTGCCGT YVLCICR 1

GGTCAGAAGTGGAAGTACTCG GQKWKYS 1

AGTCCCATGAGTGTGAGTGGG SPMSVSG 1

TGTATCGGGGTGCCGGATGGC CIGVPDG 1

GTGATGGTTGAGTATGCTCGG VMVEYAR 2

GGGCATCCCAGGATCGTGCCT GHPRIVP 1

CGTCGGGGTTGCCGCGTCTGC RRGCRVC 1

GTCTGGCAGCTCGAGCAGGCT VWQLEQA 1

CTCGGGGGGGTCGGCAGTGTG LGGVGSV 1

GCTGCCTACAATCGTTCTGGT AAYNRSG 1

CGGATGGTTCGGAGCGGCGTC RMVRSGV 1

TGTTGCCTGACTCCCGGGAAC CCLTPGN 1

GGCTCCTTCAGCTGCTCTGGC GSFSCSG 1

TCCAGGGCCTGGCGCAAGGCG SRAWRKA 3

GTCAACACTGCTGGCCTCCAG VNTAGLQ 1

ATGACGCTCTACGAGCTGCGG MTLYELR 1

TGGAGTGATTACCGGATGCAT WSDYRMH 1

TGCCGTACTGGGGCTGGGATG CRTGAGM 1

GATAAGTGCCCTTGCTTCGAT DKCPCFD 1

TTTTGGGGGGATTTTGACACT FWGDFDT 1

GAGCATGCTGCTCCGGAGTCC EHAAPES 1

GTGCGGAAGGCGCGCTGCCGT VRKARCR 1

GGGTGGTGCAGCGCGCTGTTT GWCSALF 1

TACGTCTCGTCCTATTCGGTT YVSSYSV 1

TGGCGCGGTATTCGGAGTGCT WRGIRSA 1

CTCCGGTGCGTGCTTCGCACT LRCVLRT 1

GGTTGCTCGTGGGAGATGCCT GCSWEMP 1

ATTCTCAATTGCAGCGCGGAG ILNCSAE 1

TTCACTTTTAGTTTCGAGTGG FTFSFEW 1

ACTGCCGGTCGTCTGGTCGCG TAGRLVA 1

GCGGTGGCTATCGGTGCGTGG AVAIGAW 1

GTGTCGGCTAGTTTGTGGACG VSASLWT 1

GGCTCCAACAATGGCGCTGCG GSNNGAA 1

ATGACGCATGCTGTCAGTGAC MTHAVSD 1

CGGTGCAAGATCGGGTATTGT RCKIGYC 1

GTTCACGTCCGCAACCACTCT VHVRNHS 1

TGTCAGCTCAGTGTCACGGCG CQLSVTA 1

TTTGCTGGCCGCTTGGAGTCG FAGRLES 1

CTCATGGGGCGTGCGTATCGT LMGRAYR 4

CAGCGGAGCGTCTGGTGTGAG QRSVWCE 1

CTCGGCTTCGGGACGCGGCCG LGFGTRP 1

GGTCACTGCTGCTTGGAGGTT GHCCLEV 1

GAGCAGCACCTCCCTCGTAGG EQHLPRR 1

CCTCTTAGGCGTGGGTTCAGT PLRRGFS 1

TACGCCGTGTGCCCTTGGTGT YAVCPWC 2

GGTGCGACGCAGTGCAGGGGT GATQCRG 2

TGGGTTCTGCTCAGTTGGCCC WVLLSWP 1

GCTCATAGTAAGATGGGTTGT AHSKMGC 1

CGCGCCAGCGGCCGTGGGGCT RASGRGA 3

GATCGGGGGGCCGTCTGGGTG DRGAVWV 1

GTGCGCCCTGCGGCGGACCAG VRPAADQ 1

TTTTCGCCTTGTCTGGTTTTC FSPCLVF 1

TTTGCCTGGGTGAGTAGTCGG FAWVSSR 1

TGGGTTTGGAGCATCTGTCGG WVWSICR 1

GGGGTTGACGGGTGGCATTCT GVDGWHS 1

TTGCGGAGTATCAGCTGGTCG LRSISWS 1

TCGGGCGCCAGCTCGCGTTGG SGASSRW 1

GAGGATTGTGTGGATCTGGGG EDCVDLG 1

GACGGCGCCTGGGCGAAGTCG DGAWAKS 1

GCGGTGTCCGCTAGTGCTTGT AVSASAC 1

GTGTTGCTTCAGGGTCTCTGT VLLQGLC 1

GCCTCCGAGAGGACTTGTGGT ASERTCG 1

GTGTTGCGGAGCCTCCTTTCT VLRSLLS 1

ACGGCCTCCTTGCATCTGGTT TASLHLV 1

TTCACCGACACGTGCTGCAGG FTDTCCR 1

GGTAGCGGCAGGTGCTGGTCG GSGRCWS 1

GTCGGTTTCCTTCTGAAGGGC VGFLLKG 1

TTTCTCTCGAATTCCGTCGTT FLSNSVV 1

GTCGTCCAGATGGCTGGTCTG VVQMAGL 1

GGCTCTGAGCGGGATGAGCGT GSERDER 1

AGGACGTGCGCGGTCTGGCAC RTCAVWH 1

GGCAGCCTTGAGTTGTCTTCT GSLELSS 1

AGCTTGGGCTCGAGGCGCACG SLGSRRT 1

TGCGGGTCTCGGCGCTGTGTT CGSRRCV 1

GGGCGTAGGTGGGTCCAGGGT GRRWVQG 1

AAGGGGTGTACTGCTGGTCGT KGCTAGR 1

TCTCATCCCGCCCGGGTTGCT SHPARVA 2

GACTGCCACCCGTGCAGGTTT DCHPCRF 1

CCGGATGTTTCGGCGAATGCT PDVSANA 2

ATTGATGCGCTCTTGCTGCAC IDALLLH 2

CCGAGGCCGCGCGCTGAGACT PRPRAET 1

TCGCGCATTCGGGGTGGGCGG SRIRGGR 1

CGTTCTCCGGACTATGGGGCT RSPDYGA 1

TATAGGTACGGGTTGGGTGCG YRYGLGA 1

CAGTTGGTCGATAGTCCTCCG QLVDSPP 1

CATAACCACAGCGACTTGTCG HNHSDLS 1

AGGGGCGACGTGATGGGCGTT RGDVMGV 1

GGGCTGGTTACTGACTTCATT GLVTDFI 1

TCTGATTCGCGGTTCGTTAGG SDSRFVR 1

AGTAAGCTTAGCACGATCCCG SKLSTIP 1

GCGTTGTTCGAGGCCCTGTCG ALFEALS 1

TGGCGTTGTCGCGCCGACGTG WRCRADV 1

CGTCTGAGCTGTGGCCCTATT RLSCGPI 1

AGTAGGGTCATCGAGTGTCCT SRVIECP 1

GTTAGCGTGCGTGCTTCGCGG VSVRASR 1

CACCCCTTCTATCGGGAGCCC HPFYREP 1

GGCAAGTTGACGAGGGTGGGT GKLTRVG 1

TGCATCGAGGTGTCGCTTGTC CIEVSLV 1

CGCGTGATGAGCCTGAGGCGG RVMSLRR 1

TGCCCGGTTATCGATTTCACG CPVIDFT 1

CGGTTCGCTCCTAGCGGTGCG RFAPSGA 2

GTCGTTCGGTCTATTTTTCTT VVRSIFL 1

GTCTTGGCCTGCGTGCCTTTG VLACVPL 1

GGGCGCCAGGATGCGGGGCTT GRQDAGL 1

TTCCGTCTCCGGTACGTGACC FRLRYVT 1

CGGGGGGCTGTTTCCCCTAAC RGAVSPN 2

GAGAGTTGGGCGACGAGTTGT ESWATSC 1

CTCGGGCGTCCTGGGTACCTT LGRPGYL 2

CACAGCGGTGCGAGGACGCGT HSGARTR 1

CGGTCCGTGGTGCATATCGAG RSVVHIE 2

TTGAGTGGCGTCGTCTCCGCT LSGVVSA 1

CGGGGTATGTCGTTCGCCCTT RGMSFAL 1

GTGGTGGATGGGAGCCGTGTT VVDGSRV 1

TCCCAGTGGGGGCACAATCAG SQWGHNQ 1

CGTGCTCCGACGCGCGTTCCG RAPTRVP 1

CCGTTGAGCACCACTTCGCCT PLSTTSP 1

GTGGGCTCTTACGTCACTCGT VGSYVTR 1

CCGCATATGTTCCTTGGCCAG PHMFLGQ 2

GTCGTGATGGTCATCGGGAGT VVMVIGS 1

AGTTCTCATAGTTTCCACATG SSHSFHM 1

GTCCTTAGTCTCCTCAATGGT VLSLLNG 1

GCCGCCGTTTTGTTTCGCCTC AAVLFRL 1

GCTTTGGTCACGGTCGCGCTT ALVTVAL 1

CAGTGTTTGCAGTATTCCGGT QCLQYSG 2

GACTTCAGCCATCGCTGTGTT DFSHRCV 1

AGGCATTATAGGACCGGTGGT RHYRTGG 1

ACCGTGGCCTTTCTTCTGGGT TVAFLLG 1

GGGATGGTTGGGGCGCTGAGT GMVGALS 1

CCGTTGCAGTTGTGCGAGCCT PLQLCEP 1

GATATTTGCGTTTGCACCCCT DICVCTP 1

TTGGGTGAGTTGCCGAGGGTT LGELPRV 1

GCCTTTGCTGACGACGTCCGG AFADDVR 1

GTTTGCGGCACCGCGGAGTCT VCGTAES 1

TGCCACTTTTGCGCTGGCCGT CHFCAGR 1

GCTGCCGCGGCGGGCGCGGAG AAAAGAE 1

GTCGGCACCAAGGGCGAGTTT VGTKGEF 1

GCTAGGGAGCATCTCCATCGT AREHLHR 1

ATCGTCCGGGCGGACTACGGC IVRADYG 1

TTGCGTCTGAAGCTGCTGAGG LRLKLLR 1

GGGGTCGTTCGCGCGGTTTCG GVVRAVS 1

AATCGTAGCATGCCCCGGGTT NRSMPRV 1

GGGTGTCGGACTCGTTTGAGT GCRTRLS 2

ATGCGTTACCTGGTCCAGAGT MRYLVQS 1

GCGCGTCACCTTGAGCCGCCT ARHLEPP 1

AATCGCCGCGGCTTCGGGGCT NRRGFGA 1

GACCGTAACAAGGCGCTGACT DRNKALT 1

AACGCGCTGCGGGGCTGGAGT NALRGWS 1

CTGCAGATTGTGCGGTACCCG LQIVRYP 1

TGTGCCTACCCGATGTGGTGT CAYPMWC 1

AGGCGCTCCGCGGGCAATTGC RRSAGNC 1

GTCAGCATCACGGCTTCTGGG VSITASG 1

TGCGAGCGGATCTGGTTTGTT CERIWFV 1

CTCAGCAGCTACCGTGTTTTG LSSYRVL 1

TTGCACGGCTCGGCGGCTGGT LHGSAAG 1

TCGAGCGGCATGGGCGTTTCT SSGMGVS 1

TGGGGGAGCGGTGTTTTCAAT WGSGVFN 1

TCCGAGCGTGGGGAGTTCGCG SERGEFA 1

AGGCGGGACATGCGCGACCAC RRDMRDH 1

GTGCGGCAGGCCGGCTGTTCG VRQAGCS 1

AGTGTGCCCAATGCTGCGGCG SVPNAAA 1

TTGCTCCCCGGCAGCGGTCCT LLPGSGP 1

TGGGACGGCTTTTGCTGTGGC WDGFCCG 1

GGTAGGTTGTGGGAGTGTCAG GRLWECQ 1

TGGATTGAGTGTGTCGTTCGG WIECVVR 1

TACATGACGTGTTGCATGCGG YMTCCMR 1

ACTGTCGACGGCCGCCTCATT TVDGRLI 1

ACTACCTCTTTCGGTGGCCCG TTSFGGP 1

GAGTCTGTTTTCGTCTGCAGT ESVFVCS 1

GGGCGGTGCTTTGGGTGGCGT GRCFGWR 1

AAGCTCGGGGCGGGGTGTTCT KLGAGCS 1

TGTGACCTCAACCGGCAGGCG CDLNRQA 1

GTTCAGGTTTGGAGCTCGGTG VQVWSSV 1

TGCAGGGCGATCGCTGCGGCT CRAIAAA 1

ATGGGCGTGTGTCGGGGTGCT MGVCRGA 1

GAGCTCACCTATTGCGCGCGT ELTYCAR 2

CCCCGGCGTAGTTGCCGGGCG PRRSCRA 1

GTCCGGAGTAGGATTGTCGTT VRSRIVV 2

TCCGAGAAGCTCGTCGAGGGT SEKLVEG 1

GGGTACGGGGGTCCCACGAGT GYGGPTS 1

CGCCGGGAGCACTCTATCACG RREHSIT 1

CGCCTGGTGAGTGCTCTGCAG RLVSALQ 1

TGGCCTTCGTCGCCCTCGTCG WPSSPSS 1

CTTAGTGATCGTTCGCGGCAT LSDRSRH 1

TTGGGGGGTCGGAGGCATAGT LGGRRHS 1

ACTTCGCCGACTAGGTATGGG TSPTRYG 1

CAGCTTTATCCCTGTCGTCAG QLYPCRQ 1

GTTGTCCGCCTCATTGATTTG VVRLIDL 1

AATTACACTAGCGACGCTGCT NYTSDAA 1

TTTGCGGAGCCCTCCATCGGT FAEPSIG 1

CATCCCACGCTCTTTGTGCCC HPTLFVP 1

GAGGTCTTGCCCTCGACTTTG EVLPSTL 1

TATGCGTGTTGCTTCCACCTT YACCFHL 2

ATGCTGGTGATCGGCGTGCTC MLVIGVL 1

ATCTACTGGGTGACCGAGGTT IYWVTEV 1

CTGCGGGACTCCGGGTCGTTT LRDSGSF 1

CGGCTTACCGACGACGAGTTT RLTDDEF 1

GGGTATCGTAGGGAGGCGCGT GYRREAR 1

CGGCCCCGCGTTCAGGTCACT RPRVQVT 1

TCGAGTGGGTCCTCTGTGTTT SSGSSVF 1

TCTTTGTTGGGGAGCGATCCT SLLGSDP 1

GGCAGCCAGTGGCGGGCGCAT GSQWRAH 1

TATTTGAGCCGCGGTGAGCGG YLSRGER 2

AATGCTCGGACGTTCTATTTT NARTFYF 1

CCGGCGGCTTACAAGAGTACT PAAYKST 1

GTCCGCGTGAGGCTCCGGTAT VRVRLRY 1

TTTCCGCACGGTGCGCTGACG FPHGALT 1

AGTAGGTACCGGTTGTTTGGT SRYRLFG 2

TATCGCGTGAGGGCGCGGTGG YRVRARW 1

GACTCCTGCCGGGTGACTCCG DSCRVTP 1

TGCAAGCCCATGTGGAACTGC CKPMWNC 1

TGGTGTTGGGGCGGGGAGGCT WCWGGEA 1

GCCCAGCCTAGCCTGGCTTCT AQPSLAS 1

GAGCGGGCTAGTGCCACCTTG ERASATL 1

GCTAGGCTCGGGGTCTTCAAC ARLGVFN 1

TTCGGGTCTATGCTGAATCTG FGSMLNL 1

GCTTGGGTCGTGATGCTCAAT AWVVMLN 1

TGGTCGTGTCGCGGTGTTTCG WSCRGVS 1

AGGGTCACCAGGGCCGTGTCG RVTRAVS 3

GCCAGGGCGTGCAGCGCTCCT ARACSAP 1

TGGGGTGTTGAGTGCCCGACG WGVECPT 1

GTCACTTCGTGCGTTGGGAGG VTSCVGR 1

AGCTTGAATGCTCTTGGGCAT SLNALGH 1

CATCTCCTGGTCGGGAGGAAC HLLVGRN 1

CTCCGTTGGTGGACCTGGAGT LRWWTWS 1

ATTGGCCGGTCCGGTAATGAT IGRSGND 1

ATCCAGGAGACGCTCTTCCTT IQETLFL 1

TGTCTGACTTTCCTCCTGAGG CLTFLLR 1

GGTGCGGTGAACCCTGTTGAG GAVNPVE 1

GCGGGGCGTTGTTTCAGTAAG AGRCFSK 1

ACCGCTGGGCTCATGGAGACC TAGLMET 3

GGGCTTGAGGCGTGCGTTGCG GLEACVA 1

CTCGGCATGCGGCAGTACTGT LGMRQYC 1

ACCTCGTGGCGGAACGCGTCT TSWRNAS 1

TCGCGGCACGGGGTCATCGCG SRHGVIA 1

AGCTTCCCGCGTTGTAGTTGT SFPRCSC 1

GGGAGTGGCTGCAATGTGGTT GSGCNVV 2

GTCCTGCGGGTGTTTCCTCGT VLRVFPR 1

ACTGGGCGGTGTCACCTCAGT TGRCHLS 1

TATCAGCTCAGTGTGAGTTGT YQLSVSC 1

GGGCGGACTATGTGGTGTGTT GRTMWCV 1

TTGTCCGTCATCGTCTTCCCG LSVIVFP 1

TGCAGCCGTCGGGAGCGCCTT CSRRERL 1

AGTGGCAGCCGGGATTGCGGT SGSRDCG 1

CGGCGCGGCAAGATGGTGGCT RRGKMVA 3

CTGCGGGGCTTTTACACTTTG LRGFYTL 1

TCTATGGTGGAGAGGGCCATT SMVERAI 1

GTCGGTAATAAGGGGGTGGAG VGNKGVE 1

CGTCTCGACGGCGGCGACTCT RLDGGDS 1

GGGCTCAGCTACAAGTTTCAG GLSYKFQ 1

GCCTATGCCGCGCCGATGCCT AYAAPMP 1

TTCGTTGGCAGGTGGTCTTAC FVGRWSY 1

ACTTGGAACATGATCGGGGCG TWNMIGA 1

GTTATCCCGTCGCCGGTGGTG VIPSPVV 1

GGTGGGTCCAGCGGCAAGCGT GGSSGKR 1

TCGCGCGACACCGTGTCGCTG SRDTVSL 2

TGCGGCTGCAAGGGCGGGTTT CGCKGGF 1

GTGAAGTGCCGCTGGCTTAGC VKCRWLS 1

TCGTCGCTTCGTAACATTCAT SSLRNIH 1

GAGTGGCCGAGTCGGATTGCT EWPSRIA 1

GGCGATGTGGGTGCCCTGGAT GDVGALD 1

GCGCTGATGGTTCTCGCTTAT ALMVLAY 1

GACTTGTATCACGCCGTGTTG DLYHAVL 1

CCGTGTTGGGACTGCAGGTGT PCWDCRC 1

AGTCACCAGGTGGGCCAGGTT SHQVGQV 1

AAGGTCACCGGGCCGTCGGAT KVTGPSD 1

TGGAGGGCCTCCAGCGCGCGT WRASSAR 1

GATCTCCGCTCTAGGACGCTT DLRSRTL 2

AATTGCGGCGGCGGTGTGTCT NCGGGVS 1

TTGTTGGAGAATATCGCGTTC LLENIAF 1

ATGGGCTGGGTCGTTGATGGG MGWVVDG 2

GTGCAGCTTTCGTCTGTCCGT VQLSSVR 1

TCTGGTAGCAACCGCCAGTGT SGSNRQC 1

TCGACCTGGAGGAGGGGCGGG STWRRGG 1

CGGGACTCTCCCTCGGATGCT RDSPSDA 1

GCGTGTGGTCCGATGAAGGTT ACGPMKV 1

GGGTGCCGGTTTGCGCTGTCT GCRFALS 1

GGGCGGTGTCTGCCGGGCCTG GRCLPGL 1

AGCCGGCTGACGCGCGGGCTC SRLTRGL 1

AATTCTGGGACTTGCGCTACT NSGTCAT 2

CTCGTGCCTCTTGGTGATGGG LVPLGDG 1

TTTTGCTGCTACCCTATGGAT FCCYPMD 1

CATCAGCAGCTGGGCGACGTT HQQLGDV 1

GTCATGGGTGAGTCGGACTCC VMGESDS 1

ATTCGGAGTACGTCTAGGCAT IRSTSRH 1

TGTTACAACTGCACCACGAGT CYNCTTS 1

TGGGGGCTCTCTATGGTTACT WGLSMVT 1

AAGATTCGCGTCTGTCTGGGT KIRVCLG 1

TGTAGTGTTCGGTTTGGGTAT CSVRFGY 1

TGTCTTCTGGGCAATATGCCT CLLGNMP 2

TTCAGGCGGTCCGGTTACCCT FRRSGYP 1

GTGGACCGCCGGAGCTCGTCG VDRRSSS 1

CCTAGCTCGCGTTGGTGCCCG PSSRWCP 1

TTGCGTAGCATGTGGGGCGTG LRSMWGV 1

CGGGGGTCTAGTCTCGGGGCG RGSSLGA 1

CACGCTGCGGGGTTTTGCCCT HAAGFCP 1

CGGTTCGTGAGGGTTTTGGTG RFVRVLV 1

CCTCTTCCTCCCATCACTTCG PLPPITS 1

CCTTCCTGGTGGGTGGATACT PSWWVDT 1

GTTTGGTCGAAGAATTCGGCG VWSKNSA 1

GGGTGTTTGTTTTGCTACGGG GCLFCYG 1

GGTTCCTTGCATCGGATCATG GSLHRIM 2

TTTCGTACCTTGGGCTGTTCG FRTLGCS 1

GCTCTGCGTTCGCCGCCGCAT ALRSPPH 1

ACGGTCATGCTGGTGGTTCCG TVMLVVP 1

CAGTTGTATGAGTTGACTGTT QLYELTV 1

CCTAATATTGAGGTGAGCGCG PNIEVSA 1

TGGTATGTGTCGTGCGCTCTG WYVSCAL 1

CCGGTTTGGGATGCGGCCCTG PVWDAAL 1

CGGGGGTCCGTGTTCCGTTTG RGSVFRL 1

TGGGGCTGTCGCATCGATTGT WGCRIDC 1

AAGCGTCATCTCGGCGGGCGG KRHLGGR 1

ATCTTCCAGGTTGCCCACAGT IFQVAHS 1

AGCCAGTCGCATCGCCGGCTT SQSHRRL 1

CCCGTTCGGAACTCTCATTTC PVRNSHF 1

TGGTCCTTCCAGTTGACTCCC WSFQLTP 1

GTGAAGTTCATTTCTGGTAGC VKFISGS 1

AGGGTCGACTTTTTGTCGCCT RVDFLSP 1

GTTCTGCTCTCTTTTTATATT VLLSFYI 1

AGCAACGCGTTTTGTTGGGGT SNAFCWG 1

CGCCGCTGGTCGCCGCAGCCG RRWSPQP 1

TGGCAGAATTGGACCCTTGCT WQNWTLA 1

GGCGGTCCGCCCGGGGTCTCG GGPPGVS 1

GGCCTGAACAGCGTTGATCCC GLNSVDP 1

GCCTCTCTTTGCGCTCTGACG ASLCALT 1

CCCGCGCAGTCTAGCAGGGGT PAQSSRG 1

TCGCAGCCCGCGGCCCCTCTT SQPAAPL 1

TACAGCGTCGGGTGGAGCGTG YSVGWSV 1

GGGTCGATGACTGAGGGGTAT GSMTEGY 1

GGTGGGAGTCGGCGCGTCGTG GGSRRVV 1

CGCCTGCGTGCGGAGCTTCTT RLRAELL 1

ATTGTTTACGGGGGGCCGCTT IVYGGPL 1

ATCGAGCGGATCGATGTGGTT IERIDVV 1

AGTTATGCGAGTGGTCGCGTT SYASGRV 1

CACGTGTGGTTCCGGCAGAGG HVWFRQR 1

AGGGCTGGCTGGGGTGCGAGT RAGWGAS 1

GTTCTGGGTACTGGGCCTATC VLGTGPI 1

TATCCGTGGGGTGGCCTCGCT YPWGGLA 1

TTCCGCGGGAAGTCTCAGCCT FRGKSQP 2

GATTTCCGCGTCAGCATGCGT DFRVSMR 1

GTGGCGCAGGACGGCCCTTGT VAQDGPC 1

TTTTGGGTGTCCTTGAGTGAG FWVSLSE 1

ATGGTTGGTGTCAGCAGGTGC MVGVSRC 1

CGGGAGCGGATGGGCCCGCGT RERMGPR 1

CGCCGCTTGGCGAATAATCCG RRLANNP 1

GTCACTTGGATCTACGCTCTG VTWIYAL 1

GTGGAGAACCTCTGGCTGTAT VENLWLY 1

CATGTGGTTCGGTGCCGGTCT HVVRCRS 1

CACCCCGACGCCCGGGGGGAC HPDARGD 1

CGGTGGACCTTGATGTGGGTG RWTLMWV 1

AACTGGCGGCTTAGGGCGGAT NWRLRAD 1

CCTCGTGCTTATAGTTTTCTG PRAYSFL 1

GGCCCCCACGCTTCGGTTAGC GPHASVS 1

ACTCGTGACAGCTATTGTGGG TRDSYCG 1

TCTGTGCTCATGACTACGGCG SVLMTTA 1

TGGTGCCTCTGGTACCGCTGT WCLWYRC 2

GTCCGGCGCGGGGATGTTTGT VRRGDVC 2

CGTGACTGCGGGTTGAGGGCG RDCGLRA 1

GGGAACACGGCGGTCCCGCTT GNTAVPL 1

CACAGGATCATCACGGTTCTT HRIITVL 1

GGTTTCAGTGGCTCCAAGACG GFSGSKT 2

CGGTTCAAGCAGCTCAACACG RFKQLNT 1

GGTGGGAGCAGCATTGGTCTT GGSSIGL 1

GGCTATTTCAGTCGTGGCAAC GYFSRGN 1

TTTGTCCCCGCGTCCACGTGG FVPASTW 1

CAGGGGGCTATCGTTACCATT QGAIVTI 1

TACCCGCGGTTTGACGAGTGG YPRFDEW 1

TGTGGTAATAAGAGGTGGGGG CGNKRWG 3

CATTGCTATCGTCGTCCGCGG HCYRRPR 1

CGCGCCGTCTCCGGTGGGTCT RAVSGGS 1

GCGCAGCGGCCGGGCATTAGT AQRPGIS 1

GTTGCTCGGACTGGCAGGAGT VARTGRS 1

TCGGCCCTCGCCGTTTGGGGT SALAVWG 1

TATGAGCGCGGTCCGCTTGCT YERGPLA 1

CTCGGGGGCTCGGACACGGCT LGGSDTA 2

CCGCGTAGCGGCCGCGTTGCT PRSGRVA 1

TTTGTCACGGATAGCGGTGAT FVTDSGD 1

AGGCCCTGCCGCCTGACTATT RPCRLTI 1

AGCCTTGATATGGTGGTTACT SLDMVVT 1

TTTCATACTTCCCGGAGCGAG FHTSRSE 1

AGCGCTTACTCTCGGATGCTT SAYSRML 1

ATTTCCAGGAGCGCGGTTACT ISRSAVT 1

TTCTCCTTGGTCGACTGGTCG FSLVDWS 1

CAGTGCGGGGCGCTGGGCCCT QCGALGP 1

GGCGGTGCGCGCAACATGTCG GGARNMS 1

TCGGAGGGGAGTAGCGGGGAG SEGSSGE 1

CGCATGTACAGGTATTCGCAG RMYRYSQ 1

AGGTTTCGCGGGGAGGCGGCT RFRGEAA 1

CGTCTGGTTAGTCGTAAGGTG RLVSRKV 1

AGTAGCGATGTGAGCCGGCCC SSDVSRP 1

CGGGTGCTCTCGTTCCCTCAC RVLSFPH 1

TGTGTCAGGGAGTTCTCGTTT CVREFSF 2

GAGCCGCAGTGGGTCGTGGGT EPQWVVG 1

TCGTGGAGGGGCCTTCGGAGG SWRGLRR 1

GTTCTTGTGGTCTCCGGGAGC VLVVSGS 1

CTCGCGCCTTGCGTGTGTAGG LAPCVCR 1

CAGGAGTTCGTTTGTCCTGTT QEFVCPV 1

TGCGGCTCGCTGACGGACCAG CGSLTDQ 1

TTGGGTGTGCGCTGGCTTTCT LGVRWLS 1

GCGGGCTCCAGGCGTTTTTCT AGSRRFS 1

TTGCGGGTCGGTGGCGAGTCT LRVGGES 1

CCTGGCGTGGGGTTGCCTCTG PGVGLPL 1

GAGGAGGAGCGTGGCATTTCG EEERGIS 1

TTGTCGTTGATCTTCCGGCGG LSLIFRR 1

TGCGGCGAGCTCACGGCCCTT CGELTAL 1

CGCCACCGGTATATGTACTGT RHRYMYC 1

GGGCATTGTGGCGGCCACGTT GHCGGHV 1

CCTTTGAGTACGTTCGGCCGT PLSTFGR 1

GACTTGCGCTGGGTGGTCAGT DLRWVVS 2

CGCCTGCCGAACTGTCGCATG RLPNCRM 1

CCGGAGAAGCGCCGGAGTGGG PEKRRSG 1

TATTTTCTGAAGCTCTGGCGG YFLKLWR 1

GATCGGTGCATTACGGGTCAG DRCITGQ 1

CGCGTCCGTTTTGGTATGGGC RVRFGMG 1

TACTCGCGTTGCGGTGCGGGG YSRCGAG 1

AGTGGGGCGAGTGTTATTGCG SGASVIA 1

GAGTACGTTAGCGGCTGTGTG EYVSGCV 1

TCTTGCCTCAGTTATCGGCAT SCLSYRH 1

TGGGGCTGTAGGACCGGCTGG WGCRTGW 1

ATGGGCGGCGTTTGTGAGCCG MGGVCEP 1

CACGGCGCCAGTCTGGTGCCG HGASLVP 1

GCTAGCCTCTCCATGGCGAGG ASLSMAR 1

GGCGTTCAGGCTAGGATGTAC GVQARMY 1

GATTGCTACTTCGAGACGGAG DCYFETE 1

CACGGGCGCTGTGGCGGTCAC HGRCGGH 2

GTCGCGTTCTGGCGTGCTATT VAFWRAI 1

TGGGTTCACGTCCCCTACCAG WVHVPYQ 1

GGGTGTTTGCCTGCCACTGGT GCLPATG 1

GCGCGCGAGAACGATCATGCT ARENDHA 1

TACCGGCGTTGGCAGCACGCG YRRWQHA 1

CTCTGCGGGGATGTCCAGCGC LCGDVQR 1

ATTCTGTATATGGGGTATTGG ILYMGYW 1

AGGTGTCAGAGGTGCTGGCTT RCQRCWL 1

CTCTGGAGCACCGTTCACACT LWSTVHT 1

GCGGCGAACAGTGCGTGGGAT AANSAWD 2

GACCGGCAGATGCGTATGGTT DRQMRMV 1

ACGTTGACTGCCTGCTCTCCC TLTACSP 1

TTTACGGGCACCGCGCATCCC FTGTAHP 1

TCCGATCCCAACGAGATCATT SDPNEII 1

GTGGCCTGCCTCGAGAGGGCG VACLERA 1

GAGTTTCCGAGGTACGCGACT EFPRYAT 1

TGTGTCTGTGAGGTTTTGGAG CVCEVLE 1

TGCGGCTCTTGGTGTGGTACT CGSWCGT 1

CAGCGGCCCGTTCGTATCAGG QRPVRIR 1

GGCCGGTGCTTGCTGAGCACT GRCLLST 1

AGGGACGTTGAGCTGTCTGCG RDVELSA 3

CAGCGGCCTTGCTGCCTCTCG QRPCCLS 1

CTTAATGGTTATTCGCCGCCT LNGYSPP 1

GCCAGTTTTAGTTCGGGTCCG ASFSSGP 1

CACTCTGTGTGGTGCGGTCTT HSVWCGL 1

CGTGCGCTCGTCTGCGGGTCG RALVCGS 2

ATCGTCTGGGGCGGTTGGCTG IVWGGWL 1

AAGGGCGACGATGGGTGTGCG KGDDGCA 1

CGGGTTCGTTGCGCCAAGGCT RVRCAKA 1

GTTTACTCCGATGTCTGGTGT VYSDVWC 1

ATTGTGGCTGGGTCGGTTGCG IVAGSVA 1

CTGATCGGGAGGGTGGTCCCG LIGRVVP 1

GTCCTGCGCAGTGGCGTTTGT VLRSGVC 1

TGGGCGCCGTTTGGTGTGGCG WAPFGVA 1

GCTGTGGTCAGCTCCGCTGGG AVVSSAG 2

TCGCGCTCGGGTGAGTGGTGT SRSGEWC 1

GAGACGGGCTCTGCGACGCGG ETGSATR 1

GGTACCAGTGCGGCTCTGTGT GTSAALC 2

TGCTGGGGGACCTACAGGAAT CWGTYRN 1

GTGATCCATAGTCTCAAGCGG VIHSLKR 2

TTCCGTGCGGGGGCGCAGCTG FRAGAQL 1

GGGCGGAGGCTCTTCTGCCCT GRRLFCP 1

GGTAGTGTGAGCAGCGGGACG GSVSSGT 1

GGCTACGTTGACAGCGACCCG GYVDSDP 1

TCTAGGTGGCAGGCGTTCATC SRWQAFI 1

ATGTGGCTCCCTCGGTCTGTT MWLPRSV 1

AAGGCGTTCAGCGACTGGTCG KAFSDWS 1

ATCCTTATTTGGTTGTGGACG ILIWLWT 2

TTTCTGCTCTTCGGCGGTGAG FLLFGGE 3

AGTGCTTGCAGTAGCTCGCCG SACSSSP 1

TCCGGGCCGATTGGCACGGAT SGPIGTD 1

GGGATCGAGGCCGGTTTGACG GIEAGLT 1

GGGTCGCTCCTCCTTAGTTTG GSLLLSL 1

CCGTGCCTGGGTATTGATTGC PCLGIDC 1

ATTGCGGGCGACGGCACGTGT IAGDGTC 1

TATGGCTGTCGTGCCACGCTT YGCRATL 1

GCCAGGGTCAGGAGGACGACT ARVRRTT 1

CGGTTCCGCTGGGTGGTGTTT RFRWVVF 1

AGGGTCGAGTCGGACGTGATG RVESDVM 1

CGCGATAAGCGGACTCTCTAT RDKRTLY 1

GGGCGGAGGCTCCTCTGGAGT GRRLLWS 1

CCCGAGTGGGCGGGGTCTGGG PEWAGSG 1

ATGCATTGCAGGTCTAGTGGT MHCRSSG 1

GGGGATCGCAGCGTTAGGTCT GDRSVRS 1

ATCGGTGGTGGGCGCACTGCG IGGGRTA 1

TGGTCGGGTGCCTGTCATTGG WSGACHW 1

ATTCGGTATTTGCTGGACCAG IRYLLDQ 1

GATCATTGTGAGGCGATTGGT DHCEAIG 1

GCTTTGGACTGCGTTCAGCAC ALDCVQH 1

GCCGACTTTACCGGCAGTGAT ADFTGSD 1

GTCCCTAGTGTTGGCGGGAGG VPSVGGR 1

CGCGGTGGGCCTTGCGCGTGC RGGPCAC 1

AGTTTTTACTGGGGCAGTGTT SFYWGSV 1

GAGCCCTTCCGCTCGATTTGT EPFRSIC 1

GGGTATCGCAGCGGCATCAGG GYRSGIR 1

AGTCTTACGGGTTTCCACAGC SLTGFHS 1

AGTGGTAGTTTTGTCGATACG SGSFVDT 1

GTCTCGCTCCTGATCTGGACT VSLLIWT 1

GTGCATGCTAGTGTGCCCCAG VHASVPQ 1

TTGGCGAACATGGTCCATACT LANMVHT 1

CTGCGGCGGGTCTACGCGGAC LRRVYAD 1

CCCTGGTGCGACTTCTGGGCT PWCDFWA 1

CGTATCTGGATGGGGATCTCG RIWMGIS 1

GCGGCCGCTCATCACCGTTCG AAAHHRS 2

ACGCGTAACACGGTGGGTTCT TRNTVGS 1

GTTGCTCCTCGGATCTCGCCC VAPRISP 2

TGGCTGTGTTACCGCTCGAAC WLCYRSN 1

AAGACGCGGGTTTTTCCGAAG KTRVFPK 1

ATGATCAGCGTGGCCGGCTTG MISVAGL 2

CCGGGTGGCGGGAGTGGTCTG PGGGSGL 1

TGTGACTTTCGCATGCTTAGG CDFRMLR 1

GGCGCGGGCAGGTCGGGGATC GAGRSGI 1

GCTGTGTTTACCGATTCTATC AVFTDSI 1

GGCGCTGACGTGAGGCGGGGT GADVRRG 1

CGCCAGGGGCGGTTGAGTCGT RQGRLSR 1

GTCTATGGCGTCGGTGGGTAC VYGVGGY 1

TTTTGGGCGGACCGGTACTGG FWADRYW 1

GTTCGGGATTTCCCTGTTCGT VRDFPVR 1

CGGCTTGCCGGCGTGTGGAGT RLAGVWS 1

CGTTCTTTTAAGCCTTTGTCT RSFKPLS 1

CTCGGCGACGTGGTGGTGGTG LGDVVVV 1

AGGGCTCTGTGCGGCGGCCGC RALCGGR 1

GTGCGGCTGCGGGGTTTCTAT VRLRGFY 2

TGTGGCCAGTGGCAGGACTGT CGQWQDC 1

GTCCGGTGGGCGTTCGGTTCT VRWAFGS 1

AATGAGGGCAGTGACGTCTCC NEGSDVS 1

GGGCCTCGCGCCCGGGGCGCT GPRARGA 1

TGTTTCCAGTTCGGGCGTGCT CFQFGRA 1

TGGGTGATTCAGGCGCTTGCT WVIQALA 1

AACCGCCAGGTGTTGCGCATG NRQVLRM 1

GAGGTGCATACTTTTGTGGGT EVHTFVG 1

TCGGTGTGTAGTGGCGTTCCG SVCSGVP 1

GCGGCGCTCGGCTTGTTTCGG AALGLFR 1

CCGTTCCATATTTGTTTTACT PFHICFT 1

TCTAGCTTCAACTGGACGTAT SSFNWTY 1

TTCTCTTCTTTGAGTCTTGCG FSSLSLA 1

TCGGCTAACTTGGGCGGTGCG SANLGGA 1

TACTTGATTTCGTGCCCTATG YLISCPM 1

TCGGCCATGGCTTCTCCCCGG SAMASPR 1

TCGAGCAGCACGCTGACTTTT SSSTLTF 1

ATCTCGTCGGTTGGCCAGGGG ISSVGQG 1

GGGGCGCGTCGGGCTGGGTGT GARRAGC 1

GGGGCGGAGAGCGTCGTGGAT GAESVVD 1

TGGCTCTTGCGCTGGACTCGT WLLRWTR 1

TTTTGGGGGTCGGCGCCTGAG FWGSAPE 1

GGGCGGGCTGGCGGGCCTCAC GRAGGPH 1

CCGACGTGGTGCACTGTGCCT PTWCTVP 1

AGGGAGCCGCGGCCGGCCTCC REPRPAS 1

GCCTGGCACGATTTCGGTGGT AWHDFGG 1

CTCTTGGGTCCGCTGAGGCGT LLGPLRR 1

TTCGTGCCTAGTTTGTGCGAC FVPSLCD 1

AGCGAGGTGGCTCGCAGCTTC SEVARSF 3

ACGCACTTTCGTCGTTCCGGG THFRRSG 1

CTCCTCCGCATTGTTGTTCCG LLRIVVP 1

TGTGTGAACTGTGGTAAGTCT CVNCGKS 1

TCGGTGCGTGCGAAGGCGCGG SVRAKAR 1

AATTCCGGTCCGGTCGCTTGT NSGPVAC 1

CGCGCGCGCGGTGTCTTGAAT RARGVLN 1

GGCTGGTTTCGGATGTGCCTG GWFRMCL 1

GGTTTTCATTGTGGTTTGTTG GFHCGLL 1

AGCTACGAGGTCACTTTTTGT SYEVTFC 1

ATCGTGCGCGTGTCCGTGCGT IVRVSVR 1

GTTGAGAACCTGCTGTCCCCG VENLLSP 1

AGTGCTCAGTGGCGTCTCGCG SAQWRLA 1

GGGTTGGCCATGTGGACCGTT GLAMWTV 1

ACCGGCTGGAGCTCCGGCAGT TGWSSGS 1

GGGAGCTACTGTGCTGCCTAC GSYCAAY 1

CGCTGGGGTCTCCAGGTGATT RWGLQVI 2

TCCGCCTGTGCGCTGCGGTGT SACALRC 1

GTGGCGCACCACTCGCAGAAT VAHHSQN 2

AGCCAGAACCTGTACTGGCTC SQNLYWL 1

AGTTTGCAGACCACCTATGTC SLQTTYV 1

AACCAGGTGGCTCCCGCGGCT NQVAPAA 1

GTTCTGTCTAACAGCTGGCGT VLSNSWR 1

GTGCCGGACCCGTTCTTTATG VPDPFFM 1

GGCCGTTGTTTGAAGGAGCCG GRCLKEP 1

GGGACCTGGGTGAGTGGCATG GTWVSGM 1

GCGGTTGTGTCGTCGGGCCGT AVVSSGR 1

GGGTTGGGGGCGTGGTCGCGT GLGAWSR 1

ATTGGTGCCAGGGAGTGGTCG IGAREWS 1

AAGGAGGGTCCTACGCTTGGG KEGPTLG 1

AAGGTCCGCCTCCGGTGGATG KVRLRWM 1

CCGCTCCTTGCCGCGACCTGT PLLAATC 1

GTTCTGTCTCCTCGGGCGCAG VLSPRAQ 1

CCGCAGCGCAGCTGGCTGTAT PQRSWLY 1

ATGGGCGTCTCGCGCAGGTGG MGVSRRW 1

GTGGTCGTTACGTCGGACTGG VVVTSDW 1

CCGAGGCCGCCGGTGTTCCGT PRPPVFR 1

ACCGGGATTTACCTCGGCGCT TGIYLGA 1

ATGCCGGAGAGCTGCCGTCAT MPESCRH 1

GTGGCTAACAGGGCCCGCGTT VANRARV 1

GTCGCGAGCCTGCTGCGGCGC VASLLRR 1

CTTTCGGTGGGTCGGCAGCTG LSVGRQL 1

GTTCGGAACATGGGGTGCGCT VRNMGCA 1

TGGCCTGTCAGTATGGGCCCT WPVSMGP 1

CTGTCGGCGGTGCGCGTGCGC LSAVRVR 1

TCCACTTTGAATTTCAAGCTT STLNFKL 1

CCCGCGCCCGCTAGCACTTGG PAPASTW 3

TGTCTGCGGCCCCGTTCTTCG CLRPRSS 1

GGCCGGTGCTGGTTGAGTAGT GRCWLSS 1

AGCCGGGTGGTGGAGTTTCGT SRVVEFR 1

TGGGTCATCCGGAGTTGTTCC WVIRSCS 1

GGGTGCGCGGTTTCGTGGCAG GCAVSWQ 2

AGCTCCTGGCAGGGCAGTTGT SSWQGSC 1

GGTCCTCAGTTGGGGCCTAGG GPQLGPR 1

TGGATGCCTCCTTACCGGTGT WMPPYRC 1

TGCCTTCCTTATTGTAACGAC CLPYCND 3

TTTTGCGACACCGTGGATTCT FCDTVDS 1

GTCAGGTATAGCCGCGGTTGG VRYSRGW 1

ATTTCTAATTGGTGCTACCCT ISNWCYP 1

GTTTGGGGCAGGCCCGGCGCT VWGRPGA 1

GGCTTTACGACCGCTGGCCGG GFTTAGR 1

CGGTCGTGGTGGGTTCGTAGT RSWWVRS 1

TGTCCGTTGATCGTCTCGACG CPLIVST 1

TGTAGGCTTGAGGTTGCTTCT CRLEVAS 1

CGGCGTTTGAAGGCCTGCAAT RRLKACN 1

GTCGCGTTCACTTTGGTCGGG VAFTLVG 1

GCCCGCCAGGCCGCGTCCGGT ARQAASG 1

CGCCCTGAGTCTATGCCGGGT RPESMPG 1

CCGGTTTGGTTGAAGCTGATT PVWLKLI 1

ATGCGTCACTGTTGTTCTGGG MRHCCSG 1

TATTCCCTGTCGTGCATGGGC YSLSCMG 1

CTGCCGATGGAGCAGATCCCC LPMEQIP 1

CGGATGCTGCCGTTCAGGAAG RMLPFRK 1

AGGTGCTTGCTGGCGTGTAAT RCLLACN 1

GTGGGGGAGTGTGGCGCGATT VGECGAI 1

CAGCCGGTCATCTTTGTTCCT QPVIFVP 1

GTCCCGCTTACCGTTCACTAT VPLTVHY 1

GTTATGTCGCCTAATTACCAG VMSPNYQ 1

ATGGTGGTCGGGCTCTTTGGT MVVGLFG 1

CCGATGCTGAACCCGCGGCCT PMLNPRP 1

GTTGTCCACTGGGAGGATTTG VVHWEDL 1

GTTTGGTGGCACCGGCAGCTC VWWHRQL 1

ATCGCCGGTGACAGGGGCCCT IAGDRGP 1

GGGGCTTTCGTGAGGGGGATT GAFVRGI 1

TTGTTGAGCTCGCAGTATCGT LLSSQYR 1

AGGGTGTTCACGCCGATGTTT RVFTPMF 1

ACGGGGCGTTGGCTGAAGAGT TGRWLKS 1

CGTGGGGCGTGCTTCGTGTCG RGACFVS 1

GTTTTGCAGTCTGTGGGGTCT VLQSVGS 1

CCGGCGACCTTCAGCGGGTTG PATFSGL 1

AAGGCTGAGCCGTGCCGGGCT KAEPCRA 1

ATCGGGCGCTTGTGCCGGGCG IGRLCRA 1

GCCTGTATCTCCAACTCTCCG ACISNSP 1

GGCCAGAGGTGTGGGGCTGGT GQRCGAG 1

GCGCTGGACGGCAGTCAGTGT ALDGSQC 1

GGTCTTGCGCATGCGACCGCG GLAHATA 1

GCGTCGACTCATGGCATGGAC ASTHGMD 1

GCGTTGCTTTGCAAGGGCCAT ALLCKGH 1

GTCGCGCGCTCGGGCACGCTG VARSGTL 1

GTTCGGCTGCGCGAGTGGTTG VRLREWL 1

GCCGTGTCCAGGGCCGGCTGT AVSRAGC 2

CCCATTGTGGAGGGTGCTGCT PIVEGAA 1

CCGCAGCGGAACGATGGCACG PQRNDGT 1

GTTTATGCCATCAGCCTGGTT VYAISLV 1

CTGTTGGTCTCGACGATCACT LLVSTIT 1

AGGTATGTCGTGGGTGCCGGT RYVVGAG 1

GTCGTCCTGTGCTTGATGCCC VVLCLMP 2

GTTCAGGGCTCTCACAGGTCG VQGSHRS 1

GGGGTGTCGCGGGTGAAGTAT GVSRVKY 1

TATAAGCTGACCTCTGGCACG YKLTSGT 1

CTCGCTCAGTCTGTCTGGCGG LAQSVWR 1

GAGCGCAGTCTGGATTCTAAC ERSLDSN 1

CTGGTCCACAGTAACGTCGGT LVHSNVG 1

AGTGGGGACGGGGGCCAGTTG SGDGGQL 1

CCGCCGTGGGTGCTGGTCCAT PPWVLVH 1

TCTGCCATTTGCGGCAGTGGG SAICGSG 1

GGGGGGCGTGGTAGTTGGGCG GGRGSWA 1

CTTATCGGCCTGGTCAAGTCT LIGLVKS 1

TCTATGGTGAATGACGTGGTG SMVNDVV 1

CATCGGTTTAACTGCTGCAGT HRFNCCS 1

ACTTGTTTCTCTTTGCGTCTG TCFSLRL 1

GGCTGCTCTGCTGCCCGTTCG GCSAARS 1

TCGCGCTTGTGCCATAGCGGG SRLCHSG 1

GTCCGGTGCCTCGGTGTCTGG VRCLGVW 1

TGTCGCTTGAGTCTTGATGCC CRLSLDA 1

CCCGTTTGGACGTACGGGCCG PVWTYGP 1

AACCAGAACGCTCAGCTCTCG NQNAQLS 1

GGTCCGCCGCCTAAGTTTATG GPPPKFM 1

ATCGCCGCGAGGATCGGCTTG IAARIGL 1

GTCGGGATTGGCCGGTGCGGG VGIGRCG 1

CGCCGGGGCGCTGACTGTAGC RRGADCS 1

ACCGCCTTGCTGTGCGTGGCG TALLCVA 1

TGGCCTTGTAGTGACGCTGAT WPCSDAD 1

ATGGTTCGCAGTCTCTGCGAG MVRSLCE 1

TATGGGTCTCTCGTCGGCCCT YGSLVGP 2

GGCACTCCCAGCGCGTGGGCT GTPSAWA 1

GGGGGGCTCATGGCTCGCTGT GGLMARC 1

TACTGGCCCTCCTGTTACGAG YWPSCYE 1

GTGAGGAGGAGTGTTAGTCGT VRRSVSR 1

TGCGGGCTCGGGCTCGGTTGT CGLGLGC 1

TGTCAGACGCGGTCGCTGCCG CQTRSLP 1

CATCGGAAGGCGTGGTTTCTG HRKAWFL 1

ATTCGGGAGTGTCAGTACTGT IRECQYC 1

AGCGGTCCCTGGGCGGAGATT SGPWAEI 1

CTTTTTGCGCTGGCGCTTTGT LFALALC 1

GTGAGTAAGCCGGACTACAGT VSKPDYS 1

TCTTGCCTCAGTCCGGGGTGT SCLSPGC 1

CTCTGCCTTGTTTGCGACCCT LCLVCDP 1

ATGCGGCGGGGGCTGCAGCCG MRRGLQP 1

CGGCTCTCTAGTTTTATTCCT RLSSFIP 1

ATGCCGGAGCTTACCGGTCGG MPELTGR 1

GCCCTGGGTTGGAGTAGTTCG ALGWSSS 1

TCCAGGTTTAGGCAGAGGGTT SRFRQRV 1

TTTAGGGTGGTGTATCTTGGT FRVVYLG 1

TTGATTAACCGCGGCTCGCGT LINRGSR 1

GTGGGTCGGCACGAGGCTAGG VGRHEAR 1

GCGAGTATCTTTAGGGTTCCT ASIFRVP 1

AACTTGCGGAAGGTTGCCAGT NLRKVAS 1

CCTGGGCGCTGTGCGGGTTGT PGRCAGC 1

GGGGTCTGTGTTGTCGGGTGT GVCVVGC 1

TCCGGGGCTATCCGGCTGGTG SGAIRLV 1

AGTGATATGGTTGAGGCGGCG SDMVEAA 2

TACTCCGAGGACTCCCTTGCG YSEDSLA 1

GTCGTGTATAACCCGGAGAGG VVYNPER 1

GAGGTGAACTCCGGTAGTCCT EVNSGSP 1

TCTCCGTGCATCAATATGCGT SPCINMR 1

GCCTTGTGTTCCGGGTCGCTT ALCSGSL 2

TGCAATCGTCTCCGTCGGGAT CNRLRRD 1

TTGCTTAGGCACGTGCATGCG LLRHVHA 1

GCTCTGGCGCGGGTTGTCACT ALARVVT 1

GAGAGGACTCTGCGCGGGCTT ERTLRGL 1

GAGTTGCGGATCAGGCACAAG ELRIRHK 1

GCCCGGTGCATTCGGTTTGGT ARCIRFG 1

GGCGGCGGGGCCCTTCTGGGT GGGALLG 1

GGCGAGTTGCTGCTTCTTGTT GELLLLV 1

GTTCGTGGCAGGAGTAATCAG VRGRSNQ 1

CCCCACCCGCGTCACGTGCGT PHPRHVR 1

CAGCGTATCTGTTGCCACATG QRICCHM 1

AACAGCGAGTCGCACGTCGTG NSESHVV 1

GACGTCAGGAGTGGCGTTAAG DVRSGVK 1

TATCGCTTCGAGTGGCCTTGG YRFEWPW 1

AAGGGGTGTCGGTGCAGGGTT KGCRCRV 1

CACTGCGTGGAGCCCTGCCTG HCVEPCL 1

GGTGTCTGCCGCTACTGTCCG GVCRYCP 1

TGCCCCGGGCGCTGGTTTGCT CPGRWFA 1

TGTGGGCCTTCTCCCCCTGTG CGPSPPV 1

CGTGCGCGGCTCGGCAAGTTT RARLGKF 1

ATTAGCTTCCACGGCGCCAGC ISFHGAS 1

CTGGTTCAGTCTGCCTCGTGT LVQSASC 1

GGCTTCAGCGCGCTGCCCAAG GFSALPK 1

ACGAGTTTGTACTTGGGTTGT TSLYLGC 1

TGTTATCTCTGCAGCTATCTG CYLCSYL 1

GTGCTTCGGAGCGGTCCGTGT VLRSGPC 1

CTGCACGGGCCGGCTCACCTG LHGPAHL 1

AATGGGAACCGTGTCTTCAGT NGNRVFS 1

GCGGTCAGGGTGCGGTTTTTG AVRVRFL 1

TCCGTTTCCGCGTGTAGCTGG SVSACSW 1

GTTCGTGGTGCCTGGTGGCCC VRGAWWP 1

TGGCAGTACATGAGGTTTGCG WQYMRFA 1

CTCAATCAGCGCTCGTGGATT LNQRSWI 1

GTGGAGGGTACCTTGGACTCT VEGTLDS 1

CTCCTGCCGCTGCGTGTCTCG LLPLRVS 1

TGTCGGGGGAGGTGGTTCTAT CRGRWFY 1

GGCGGGTTGGTTATGCGGTAT GGLVMRY 1

TGTGGTCTGTTTGGGTTGCCG CGLFGLP 1

GAGATGGTGTATTTCTGTTGT EMVYFCC 1

TATCGGCCGACGCGGATGAGG YRPTRMR 1

CCTTCGCGTGGGAGGACTTGT PSRGRTC 1

GGCGGCGCCTCCGGGGGCAGG GGASGGR 1

TCCAGTACCTCGCACTTTGGT SSTSHFG 2

AGCGTGTGTTGTTCTATTTCG SVCCSIS 1

CCTCTTAGCATGGAGGCTATT PLSMEAI 1

ATGCATTTCGGGGCGCAGCGT MHFGAQR 1

CGTTGGATCGCGTCGTGCCGG RWIASCR 1

TCGTTGGAGGTTTGCGTTCAG SLEVCVQ 1

AGGGTGGGGCGTAACCCGCGT RVGRNPR 1

GGCCGGTTGTGCCAGCGTCGT GRLCQRR 1

GCGTTCGGCTGCCGGGCTGTC AFGCRAV 1

AGGGCTGCGAATTTGAAGTGG RAANLKW 1

CTCAGGGGCCCGGAGTTGCTG LRGPELL 1

CCGCGCCCGATGTTGTGGTTG PRPMLWL 1

CATGTTGCCGATGTCTGTCGT HVADVCR 1

TCTAAGGGTAATAGCAGGGGT SKGNSRG 1

AGGCTGAACACGCGTGACCGC RLNTRDR 1

ATGCCCAATCGGGCCGTTGCG MPNRAVA 1

CGGAATGGCGAGCTGTGCCCG RNGELCP 1

TGCTGGCGGCGGCTTTATTCT CWRRLYS 1

TGCGGGGTGGGGCTTGTGTTG CGVGLVL 1

CGGTACTATGCGGTCGCCCGG RYYAVAR 1

ATGGAGGGCTTGAGTACTGTT MEGLSTV 1

CACTGTGCCGATTGGTGTTTG HCADWCL 1

AGTGGGTTCAGGGCTATTGCG SGFRAIA 2

GTTCACCGCCTCTCGCTGCCT VHRLSLP 1

AGTGTCTGCTCGCCGCTTACT SVCSPLT 1

TGGAGGCAGGTGCTGGGCCGG WRQVLGR 1

CGGCTTGTCGAGAGGGCGATG RLVERAM 1

GTGCGGGCGAGCAGCAAGGGC VRASSKG 1

AGCGCCCGGCGTTCGACGGAC SARRSTD 1

GACGGTCGGGTCTCCAGGGCT DGRVSRA 1

GCGGTGCTCTATTTGGTTCTC AVLYLVL 1

CGCCCCGCTTGCGTGTGGGGT RPACVWG 1

CTGGACCTGAGGTGCTTGATT LDLRCLI 1

CGGCGTGACTCGGTCAGCCAG RRDSVSQ 1

GCGGGTTCGAACATTGGCTCC AGSNIGS 1

CAGGATCTCGCTTTCTGTTTT QDLAFCF 1

CGGCGGAGTTCGTGCTCCTCT RRSSCSS 1

TTGCGCGAGGATTTGTGTCGC LREDLCR 1

TGGAGCGAGAGTCGCGGCGCT WSESRGA 1

GTGCGCGTCAGTGGCCGCCAG VRVSGRQ 1

GCGGGCTGGAGTGCCTCGGAT AGWSASD 1

CGCAACGTCGATACGCACGCG RNVDTHA 1

ATGCTGGAGTACATCATCATT MLEYIII 1

TGCCGGTGGCAGTGGTCCCGG CRWQWSR 1

GCCGCGCTGAGTAGGCTGCGT AALSRLR 1

GTTCACTGGATGTGCGGGACG VHWMCGT 1

TCGTGCATCTATAGGAAGGTT SCIYRKV 1

GTGTGTGAGGGCGGGTTCGTT VCEGGFV 1

TGGGCCAATAGCAGTGAGCTT WANSSEL 1

TCGCAGGATAGCGATGACTCT SQDSDDS 1

CCGGTCAGCAGTGCCCGTACT PVSSART 1

TTGAGTCGGTGTTGTGTTGAT LSRCCVD 1

TATATTTGTCGGTGCGGTCGG YICRCGR 1

GTGCTGGGTCGTTCTGGGTGC VLGRSGC 2

CGGGGGCGGGACCGGGGTGCG RGRDRGA 1

GTGGGTGAGGGTGAGAGCAAT VGEGESN 1

GGGTTGTGGCCCGGGGATCTT GLWPGDL 1

TCTCTTCCGTCCGCGCCGGAT SLPSAPD 1

CCTGGGTTGTTCGCTGGCTTT PGLFAGF 1

GCGGGCGTCTCTGACGGGATG AGVSDGM 1

GTTGCCAGGCTGCGGTGGCTT VARLRWL 1

ATGGTTACGTTGCTTGTTGTC MVTLLVV 1

CTGACCTATATCCTCTCGAGG LTYILSR 2

GTGTGTGCGCGTTGCCTTGTT VCARCLV 1

GGGCGCTCCGTTGGCGCCATG GRSVGAM 1

CGTTTGCCTGTGGGTAGGGTT RLPVGRV 1

CTCCGGCGCTGGGGGGATTTT LRRWGDF 1

GCTGACGCGCTGCTTTTGAGC ADALLLS 1

AGCGCGCTGAGGTTGTGGGCG SALRLWA 1

CGTGTTATGTTGCGGGTTATT RVMLRVI 1

GGTCGGAAGTGCATGGGGTGT GRKCMGC 2

GATAGCTGGAGTCTCTGCTGG DSWSLCW 1

GTTACCATCTTGCTCGCGGAC VTILLAD 1

TTGCTCTTGAGGGACCGGCAT LLLRDRH 1

TTGCTCCAGGGCGGCCTTCTT LLQGGLL 1

TCCAAGGGCGACGTGGACGAT SKGDVDD 1

CCTGCCCAGCGGAGCGGCAAT PAQRSGN 1

TACAGTAATACTAGTTGGTCC YSNTSWS 2

CTGGTCCTCGTCAGTAGTATT LVLVSSI 1

GTGCTGACCCATTCCTCTTGG VLTHSSW 1

TGTGTGGAGCAGCACCGCGTT CVEQHRV 1

TGGCTTTATGGGCATTGTGCG WLYGHCA 1

GTTCAGGGTGGGGTTTGTTGT VQGGVCC 1

GTGGGCGGCGTTGGTCCCACG VGGVGPT 1

CGTGTGTGGTCGACCGACTCT RVWSTDS 1

TGTCGGTCGTCGGGCGCTGCT CRSSGAA 2

CCGCGTCGCGCGTCGGCGAGG PRRASAR 1

CGTCGGATTGAGAGGGCTGCT RRIERAA 1

TTCCTGGCTGGTTACCTTGTT FLAGYLV 1

GGTCGCGCGTCTGGTGCGAAT GRASGAN 1

TCTCACCTTGCGCCGCTCCAT SHLAPLH 1

CGCAGCATCTGGAGCGGGTTT RSIWSGF 1

GTTATTACCAAGCGGGTGAGG VITKRVR 2

TCCGCGGGGACGGGCGTTACT SAGTGVT 1

TTGGCGCCTTCCCTGGCGACT LAPSLAT 1

TCGTGGTTGGCTGAGCGTTGT SWLAERC 1

CGTGAGGCTCAGATTTGTACT REAQICT 1

GTCCGGGTTGTCTATGCTGGT VRVVYAG 1

TGCCGTCTCGGGGCGGTGTGG CRLGAVW 1

GGTTGTGTCTCTTCCGGGCTT GCVSSGL 1

TCTAATGTGAGCGGTTCTGCT SNVSGSA 1

GGTCCTCACAGTGGGCCTGCT GPHSGPA 1

AGCTTGAATGGGGCCAACGTT SLNGANV 1

TTGTACGGTAGCTGTTGTATG LYGSCCM 1

TATCGTATCGTTTGGGCTTAT YRIVWAY 1

TCTCCGGGCTTTCAGTTGAGG SPGFQLR 1

TTGGGGTTTAAGCTTGTCGGG LGFKLVG 1

GGGCTCTGTGTGTCCTCGTGT GLCVSSC 2

TCCTTGTGGGTGAATCAGGCG SLWVNQA 1

CGGAACGCGTGGCCGAGGGCT RNAWPRA 1

TGTCCCTCCACTGTGGTCGCT CPSTVVA 1

GTCCTTACGGGTGTGTCGATT VLTGVSI 1

ATGTTTGCCACTCGCGCCCTC MFATRAL 1

ATGCTCGACATCGCGCTGATT MLDIALI 2

ACCGTCCGCTGGTGGCAGCTG TVRWWQL 2

CATCGGGAGAAGCGCATTTCT HREKRIS 1

TTGCGCTGGCGGGGCATTCGT LRWRGIR 1

CACGTGGTGGGTCACCAGGTG HVVGHQV 2

CTTTGGTACGGGAGGGCGAGG LWYGRAR 1

GTTGTGTGGCTCTGTATCTGC VVWLCIC 1

GGGTGCGGCTACCTGGCCTAC GCGYLAY 1

ATCTACTCTCCCCAGGTGCGT IYSPQVR 1

AGTGATCGTGGGCTGACTCGG SDRGLTR 1

TCCCTGCCTGTGTCCACGGGG SLPVSTG 1

GTTTGCTATGGGGTTAGGGGG VCYGVRG 1

GTGCTGACTCTCGCTTCGTTT VLTLASF 1

GAGTGGCTCAGGATGGAGGTT EWLRMEV 1

ATCCACCCCAGCGGGGGTGAG IHPSGGE 1

ACTCTGCAGGTCGTGTACGAG TLQVVYE 1

ACGTTCGGCCTTGCGTGTCAG TFGLACQ 1

CCCGGGAAGTTCGTGAACTGC PGKFVNC 1

AGCCGCGTCAGGACGCTCCGT SRVRTLR 1

GTGTCCCCCGACTGGGAGTCG VSPDWES 1

CTTGTGCGCTATGCGGCTATG LVRYAAM 1

TTCGTTCCGTTGGCGGTCTGC FVPLAVC 2

TGTTTCGTTGGTACCCTTGCG CFVGTLA 1

AGGTTCTCCTCGCTGTCTGAG RFSSLSE 1

GGCATGTACGTGGCCGTTTTT GMYVAVF 1

CCGCTTGGGCTTGCGAGCTGT PLGLASC 1

CGGGTGATGATGTTCAGGTCT RVMMFRS 1

AAGGTCGGGGACTCCGCTAGG KVGDSAR 1

CTCTTCATCCTGGGCGCCACT LFILGAT 1

GAGTTCAGTTGCTGCTTCCTT EFSCCFL 1

AGGGACGGCGTCCGTCGCACG RDGVRRT 1

CCGTCTGGCGCGTGGGTGTTT PSGAWVF 1

CGGTCCAAGCGCGGCGAGGTT RSKRGEV 1

GTGGAGTATGGGGGGTGCGCT VEYGGCA 1

ATCGGCCCTCACATTTGGACG IGPHIWT 1

TGGATGATGACTTATCTTTCT WMMTYLS 1

GAGATTTGCAGTGACCTGGAG EICSDLE 1

CAGGCCAACCATCGCCCCGCT QANHRPA 1

TGCCGGTGCAGCGCTTCCCTT CRCSASL 2

CTGGCGTGCACCTCTTGTTTT LACTSCF 2

TGGCCTGTGAGGGACAGTGGT WPVRDSG 2

GACATGTCGCGGAGCACGCCG DMSRSTP 1

GAGGGCGGGCAGCCGCAGCCG EGGQPQP 1

GTCATTAAGATCCTTGGTTCT VIKILGS 1

CGGTCGTGCGTCTCTGACGCG RSCVSDA 1

TGGCTGTTCGGCAGCGGTCGC WLFGSGR 1

ACCGGTTGTCCCCGGGACGTG TGCPRDV 1

CATTGTGTTGTTTTGAGCATC HCVVLSI 1

AAGGCTCGGGGTGTCGCGCTG KARGVAL 1

GCGCCCCTCAGGGTTTTTAGT APLRVFS 1

TGCCGTATTGGGGATAGGCGT CRIGDRR 1

GTCATTAATGGGGGTGCCGAC VINGGAD 1

GCGAGTTACAGGCTCTTGGTG ASYRLLV 1

AGGGCGACCATTGCTCGGGAG RATIARE 2

AACCGGAGGGCCCTGTCTCTT NRRALSL 1

GTGGAGGTGATGTCCGCTGCT VEVMSAA 1

GTCCGCGTCGCTTCTGGGGGG VRVASGG 1

GCCGGGCGGAGTGCTTCTGGG AGRSASG 1

TGGGCTAGCGTTTATACGCCG WASVYTP 1

CATAGGCCTTTTAGGCGGGCG HRPFRRA 1

TCTGGGTGGGGTCTCTACTGG SGWGLYW 1

AAGTGTGAGCACTACGCGGTT KCEHYAV 1

GTTGGTCCTCTTGTGTGGTCG VGPLVWS 1

GCGGTGGGGCGTCTGAATGCG AVGRLNA 1

TTGTTTGACGCGGCCGGGTCT LFDAAGS 1

ATCCAGAGGTTCCGCGGGTAT IQRFRGY 1

GGCGGGGGGCTTCGCGTTTTG GGGLRVL 1

TGGAGTCGGGAGTTGCGCGCT WSRELRA 1

AGCTTGGTCGCCGTCGGGAGG SLVAVGR 1

TGGTATCGGCCGCGCTTGATT WYRPRLI 1

AGCAGCCGCGTCGCGATGCTG SSRVAML 1

GTCCCGTTGCTCTCCTATCGT VPLLSYR 1

TACTGGACGGCGGGGGGGGCT YWTAGGA 1

TGTCCCGCTTATTGGTCTGGC CPAYWSG 1

GCGCAGTGCAGCAACGAGCTG AQCSNEL 1

CTGGGCCGGACCTTCTCGAAG LGRTFSK 1

TCGGATCGGCATAATGTCAAT SDRHNVN 1

CGTGATGAGGGGAACGAGGCC RDEGNEA 1

ATGTCGGTTACCAGGCACGAC MSVTRHD 1

CTTGACGGCCGGAGGCGCCTT LDGRRRL 1

TTGCGGTGGAAGTCCTTGCGT LRWKSLR 1

GAGCATGCGAGGAGGCTGGTT EHARRLV 1

GCGCTCGCCGTCAGGGGCAAC ALAVRGN 1

GGTCTTCCTCGGGCGTTGCGG GLPRALR 1

GAGCGGGGTCTCTTGTTGCCT ERGLLLP 1

GCGCACACGGGCTGGCTGGGT AHTGWLG 1

CCGCCCGAGATGGTTTGGTCG PPEMVWS 1

GCGTTTGATACGAGTAGGGTG AFDTSRV 1

CACGCGCTTTGGAGTCCGGCG HALWSPA 1

ATTCCGTGCTCGCTGCTCCTG IPCSLLL 2

TTGCTGTCCTGGTTCCGTGGG LLSWFRG 1

GGCGTCGGGGGGTATGAGTCG GVGGYES 1

GTGGTCGGCGGTTGTCCTTCT VVGGCPS 1

GTGCGTCACCGTGTCAGGGGG VRHRVRG 1

GCTTTTCCGAGGGGGGCGATG AFPRGAM 1

GCGCTGGTCCGTGTGGGCGTC ALVRVGV 1

GTCCGGGAGACTGCTGGTGCG VRETAGA 1

ATTAGCCGCGTGGGTACCGCT ISRVGTA 1

ATTCACCCCAGGGCGTCGATT IHPRASI 1

GATGGGTGCAGGGGCGCCGGG DGCRGAG 1

GCGCTTGGTCACCGCTGGAAT ALGHRWN 1

GGCGTGGCCAGCCGGTATCTT GVASRYL 1

CTCAAGGTGGGTTTCAGTGTT LKVGFSV 1

GTGCAGTTCTGGTCGACCGCG VQFWSTA 2

GGCGAGCAGTCTGGCTGGCAG GEQSGWQ 1

TTCGGCTGCATTCCGATTTGT FGCIPIC 1

ACCTCCAGGAGGAGTGTGTGG TSRRSVW 1

CGTGTCTCGTCGCTGTCGAGT RVSSLSS 1

GGGCTCTGGTGCCACGATCTC GLWCHDL 1

CCTGGGCTCTGCTGCTCCATT PGLCCSI 1

GTGAAGTGCAGCGTGAGGCTG VKCSVRL 1

GGTTGTGGGCGGGGTCGGCTC GCGRGRL 1

AATGGGCCTGGCCACATGCTT NGPGHML 1

GCTAGTTTCGTCGGGCGCATG ASFVGRM 1

TCGCTCTCTCTTCGCTTGTTC SLSLRLF 1

TGTGGGCGCGGGACTTTTCGG CGRGTFR 1

TTTCAGGCCAAGGGGCTTGCT FQAKGLA 1

CTCGCGTTGGTGCGCGGTCCG LALVRGP 1

TTCTGGGGCGTGCCGGGGTGC FWGVPGC 1

TGGAGGCGTGAGTGTGTCGTT WRRECVV 1

CTTAGCGACAGGCGTTGGGGT LSDRRWG 1

GTTGACACCATGAGGACGGAG VDTMRTE 1

TGGATTAAGCAGCGCACGTCT WIKQRTS 1

GAGGGGCCTCGTCTTCGTCCG EGPRLRP 1

ATCACCAGTCCGTCCAGTCGT ITSPSSR 1

GTTTTGCGCAGCTCCTGGTGC VLRSSWC 1

TCCCTTGCTATGGGCTACTGT SLAMGYC 1

GATTCTGCTACGTGCTGTCGG DSATCCR 1

GGTGGTCATGTGTTTAGGCCG GGHVFRP 1

CTCTCGGATAACCCGTGGCGT LSDNPWR 2

AGTGGGTTCTTGTGGCAGGTT SGFLWQV 1

CTCGGTTCCGTCCAGCCGATG LGSVQPM 1

AGCGGCTCGAGTTGCACTAGT SGSSCTS 1

GATGTCCAGCAGTCTGGGAGT DVQQSGS 1

TTGGGGGTTTCGGGTCCTGTG LGVSGPV 1

CGGTGTCTGCCGGCCGCCATT RCLPAAI 1

GCTCTTTACACGCGTAAGCGT ALYTRKR 1

GGCCTTAAGGCCTCTTGTACG GLKASCT 1

TTTAGCTGGGTGCGCTTGCAT FSWVRLH 1

CGGCTGGACAATCTCGCCGGT RLDNLAG 1

CTTGATTTGATGTTCGATTGT LDLMFDC 1

TCGCTTCACTGGGCTCGCGCC SLHWARA 1

GCGGCGGGGAGGCACAATGAG AAGRHNE 1

TGGAGCGCTCGTGGCAATTGT WSARGNC 2

ACGGACGGCCAGGTGGAGCCG TDGQVEP 1

GGGCTGTCCTGGTGCGCCGGC GLSWCAG 1

ACGCGGGCCTCCGACTGTTCC TRASDCS 1

CGGGGGCGGGTTATGAGTTAT RGRVMSY 1

GAGAATGAGAGTGCCCTGTAT ENESALY 1

TGGGGGCTCAGTACGGTCAGC WGLSTVS 1

TCGCATGCGCTGCCGTGGCTT SHALPWL 2

GCGGGGGAGCCGTGCAGTCAT AGEPCSH 1

TGCGGGTGTACGGTCGTGTGT CGCTVVC 1

CAGCTGAGTAGCCGGATCCGT QLSSRIR 1

TGCGTGGGTTTGGCTATTTCG CVGLAIS 1

TTCTACTGGTGCTTTGCTCCG FYWCFAP 1

AGGTTGAGTAGCAAGGGCGCT RLSSKGA 1

GCGACGTTTACTCTGGGTTAC ATFTLGY 1

TTCTCGGACCCTCTCTTTAGC FSDPLFS 1

TGCAACCGCGGGCATGTTACC CNRGHVT 1

GAGCGTCCTGCCGAGTTTCGT ERPAEFR 3

GCGGGCTTCATGAACTGGGGC AGFMNWG 1

GCGGAGGCGTTGATCGTTTTT AEALIVF 1

TACTCGTACAAGGGGCGTTTT YSYKGRF 1

GTTTGGGCCGAGAGCCTGGCG VWAESLA 1

TTGTTTTGCGCCGATGTTTCG LFCADVS 2

TTGGACCGGAGGGTCGGTCAG LDRRVGQ 1

TACTCGAGGGAGGAGTCCAAC YSREESN 1

TTGGTTTCGCAGTCGAGTCAG LVSQSSQ 1

ATGAGCGGGGACGAGGTGGGT MSGDEVG 1

GTGCGTTTGGTTCGTGGGAGT VRLVRGS 1

AAGGGGGACCCGCGCTACGCT KGDPRYA 1

CGTTCCGTTAGCATCGAGGCG RSVSIEA 2

GCCCTGGACCGCGAGCGTTAT ALDRERY 1

ATCGTGCGGATTCCCGTGATT IVRIPVI 1

TGTTACAGGAGTCTTAGCTGT CYRSLSC 1

TATCTGCATGACAGCTGGTGT YLHDSWC 1

GAGCCGTGCAGCTGCTTCCTT EPCSCFL 1

CGGGGTTGGGCCGGCAGGAAT RGWAGRN 1

CGCTTTCCGAGGCTGCGGGAG RFPRLRE 1

GGCTATTGCGGCTTCATCCAC GYCGFIH 1

TGCGGGTCGCTGCGTACTGCT CGSLRTA 1

TTTCAGAGGAGTTGTTGCCCT FQRSCCP 1

TTTCACTTTCTGTGTATTGTT FHFLCIV 1

GTGGAGTTGGTGAGGTGCGTC VELVRCV 1

TGGGTCATGCTTAGGATGGCT WVMLRMA 1

ACCGTGAGGGGTGACGCTCAT TVRGDAH 2

TGGCACAGCTGCGAGTCGTAT WHSCESY 1

CTGCTCGATACGGACATCCAG LLDTDIQ 1

TTTGTGTTTAATCACAATCGT FVFNHNR 1

CTGCGTGATCTGGTGGCGTCG LRDLVAS 1

GGTCTCCAGTTTGCCTACCTG GLQFAYL 1

GGCGTCACCCTTCGCGAGGCT GVTLREA 1

GTTGCTTCTCTCGGGGCCATG VASLGAM 1

GGGGATGACCACTGGTCGCGT GDDHWSR 1

GTCGAGGATGGTGTGTGGAGT VEDGVWS 1

GGGCGTCTCCCTGTGTTTCCG GRLPVFP 1

TCTCGGCCGACGCCGAGCTGT SRPTPSC 1

GTTCCTGATACCCGTGCGAGG VPDTRAR 1

CCCGAGCATTTCTTCGAGCCT PEHFFEP 1

GCCTGGCACGAGTGTCGTTTC AWHECRF 1

TCGTTGGGGGCCGGCATCGGT SLGAGIG 1

GGGCGGCTCGCGGCCGATTTG GRLAADL 1

GCGTGGTTCTGCGGCCTTCAG AWFCGLQ 1

TTGCAGGGCTGCACTCGCCGC LQGCTRR 1

GACTGCGGCTCTCTGCGCTGT DCGSLRC 1

GTTCTCTATTTTAAGCGTAAG VLYFKRK 1

TACCGGAACCTGAAGCTTACT YRNLKLT 1

ATGTGGGATGGGACTGGGCCT MWDGTGP 1

CTCTGGCTTATTGAGTGGTCT LWLIEWS 1

AGGAGCCCCGTGAGTAGGGAC RSPVSRD 1

TGGGACTTGGGTCACCAGGCT WDLGHQA 1

AACACCGCGAACAGGCATCCT NTANRHP 1

GGTGGTGGGTGGTTTAGGCGT GGGWFRR 1

AGCTGCCATAGTACGGATGTT SCHSTDV 1

GGCTGGTGCCGCTCGTTGAGT GWCRSLS 1

GGCGAGGGTCGGAGGGCGAGT GEGRRAS 1

TACGAGCTGCATATGTGGTTG YELHMWL 1

ATTATCCGCTGGTCTGGGAAC IIRWSGN 1

ACCGCGGATGCCGGGTGCTGT TADAGCC 1

TACTCGCTGGTGGGTGATCAT YSLVGDH 2

CTGACCCGGAATTCCAACGGC LTRNSNG 1

GCGAGGCTTACCGTTAGTCGT ARLTVSR 1

GACTTGGGCAGCGCCTGGGCT DLGSAWA 1

ATCAGGGGCCGTATTTGTCGG IRGRICR 1

ATGCGCTTCTGTGTCAGGGGT MRFCVRG 1

GGTTGGCTGGACGTGCGCTTC GWLDVRF 1

GCGGCGCCCACTGGCGCTCCT AAPTGAP 1

CTTGGCCTGTTGCAGCTGGAC LGLLQLD 1

TTGAGTGGGCACCTCCTCGCG LSGHLLA 1

CCGGCGGACAGCGATCGGCTT PADSDRL 1

TCTCGGGCCAAGTGGTTCGTG SRAKWFV 1

TTGCCGTTCATCTTGTTCTTT LPFILFF 1

CTCGGTTCTCGTTTCCTGCTC LGSRFLL 1

AGTCCGGGCTCGGACCGGACT SPGSDRT 1

ACCTTGGAGGATCCCAGCGAT TLEDPSD 1

CCGGGGCTGTGTACCGGGTGC PGLCTGC 1

GTGGCGGATTGCTGCAGGGGG VADCCRG 1

GGTCGCCTTAGCATGTATGGT GRLSMYG 1

TTTGCTGTCGTTTATTATATT FAVVYYI 2

AGGGGCAACTTCGTGCTCGCT RGNFVLA 1

GCGGGTGTGTTGAGCGAGGAT AGVLSED 1

CAGGTCGGGAGCTGCGGGTCT QVGSCGS 1

GTTGGGCCCACCTGTGTGTCG VGPTCVS 1

TACGACGCCAGGAGTCGGTCT YDARSRS 1

CAGCATTACTTGGAGATGTTT QHYLEMF 1

GGGGGCCGGCTGGGGCCGAAC GGRLGPN 1

GTGCGGGATTGGAGCGCTATT VRDWSAI 1

AGGGTTGTCGTCAGGCACGTG RVVVRHV 1

GACGGGCTGATTGGCGGTCCT DGLIGGP 1

AGGGGCGTGGGTTTCGCTACG RGVGFAT 1

AACCTTACCGTCGGCCTCCGT NLTVGLR 1

CCGCTTAGCCTGATGAGGTCG PLSLMRS 1

TGGCCCCTCCGCATGGTTCCG WPLRMVP 1

GGCTCCTCTTCCCGGCTGTTG GSSSRLL 1

CACTGCGTGTGGTCTTTTAAT HCVWSFN 1

TCTTGGGTGTTTGACTTTATG SWVFDFM 1

TTGGTCGGCAATTGCATCATT LVGNCII 1

ACCAACGGCAGGCATCGCTGT TNGRHRC 1

GAGGTGCGCGTCCGCTTCTGG EVRVRFW 1

TGTCGGCGGTGCTCGAGTTTT CRRCSSF 1

GTGCATCGGTGTGAGGAGCAG VHRCEEQ 1

CTCGGCAACAACGTCCTGAGG LGNNVLR 1

GGGCTTCTCAGGAGGTTTATT GLLRRFI 1

TTGCGCGAGAGTCACGATACG LRESHDT 1

GCCGCGTGCCATGTCGGTGGT AACHVGG 1

AGGGTGTACGGTACGGTGGTC RVYGTVV 1

TGTTGTCCGTCTGCGTGCTGG CCPSACW 1

TTGTGTACCGTCTCTCGGGGT LCTVSRG 1

TGCGGTTTTAGGAACACTGGG CGFRNTG 1

GGTGAGCACTCCTTGTCCATG GEHSLSM 1

GTGGTCCGTCCTAGGGCGCGG VVRPRAR 1

CTGGGGCGGATCGAGGTGCCG LGRIEVP 1

GTGTACCGCAGGTTTGGGAGG VYRRFGR 1

GTGTCGTTTGAGTCTCTGCTG VSFESLL 1

CTGGGCGGGTGCCCGAGTCGG LGGCPSR 1

ATGTCGTTGTGGGCCGCGTCG MSLWAAS 1

CAGAGCGCTGACAGCGTCCCT QSADSVP 1

TTTTATTTCCACGTCGTCATG FYFHVVM 1

GATCTCGGCATTTGCGTTCGT DLGICVR 2

ATCCTTCGTGGGGTGATGCCT ILRGVMP 1

CGCAGTGGCCTGTGTAGGTGT RSGLCRC 1

GGCGGCTTCAGGTGCAGGTGG GGFRCRW 1

TTTGACGCGCCGCGTAGCCAG FDAPRSQ 1

ATGTGCAACGTCAGCTCGGAC MCNVSSD 1

TCTTGGCGGGGCCGCGATGGG SWRGRDG 1

TGCTCGACCGCGAGCGGTGGC CSTASGG 1

CACTGCCTCTGGGAGATGTTG HCLWEML 1

TTGCCGGCGGGGGCCGACCTT LPAGADL 1

GAGCACCGGACGTATCCCCGG EHRTYPR 1

GGTTTGCCTATTTTGTGCCGG GLPILCR 1

CGTTATGGGCCTGCCGGGTCG RYGPAGS 1

CGCGGGCGGGGCCGGGCCGTT RGRGRAV 1

ATTAACAACTGGGGCCAGGGG INNWGQG 1

TGCACGGCTTGGAAGCTCTTT CTAWKLF 1

GTTTGGATCAAGGATTTTAAG VWIKDFK 1

GGTGTCGGCGGGTTGGTTCGC GVGGLVR 1

GCCCGCGCCCAGGAGTACGTT ARAQEYV 1

TCGAAGCGGACCGTCGGCTAT SKRTVGY 1

CGTAGGCTCGTTACGAGTCGC RRLVTSR 1

GGGCAGGTCCACAGGGCGGGC GQVHRAG 1

GTTTACCGTATTCTCCGCGCT VYRILRA 1

CCCCAGTCCCATCTCTGGGCG PQSHLWA 1

AGCGCTGCTGGTTTGGGTTTG SAAGLGL 1

GTGCCGTCGTCCGGGGATGCT VPSSGDA 1

CCCGGTTGCAGGCTTATCTGG PGCRLIW 1

TGTCTGCCGCGTTGTACCAAG CLPRCTK 1

TGTACGGCTCAGCCCCCTTAT CTAQPPY 1

GGTGGTCTGAGCTATCGTCCT GGLSYRP 1

TGTGTCGCCGGCGGCACCGTT CVAGGTV 1

TTGGGCGCTGCCGTGTTTCTT LGAAVFL 1

GGTCTTCGTGGGCCGGGCTCT GLRGPGS 1

CCCATTGGTTGGGTGTACCAT PIGWVYH 1

TTGCGGGCTTCCCCGCGCGCG LRASPRA 1

CATATGCTTTGCCATTCGTTT HMLCHSF 1

TTGGCTGGTTTGTCCTCCAGT LAGLSSS 1

TCCATTCTTTGCTCGCAGGAG SILCSQE 2

TCGTGGCAGACTATGTGCGTG SWQTMCV 1

TTCTGGGAGGGTATGCGGGTT FWEGMRV 1

CTCTCCTTTGGCGATGCGCTC LSFGDAL 1

GAGTGTCTGTGGTTCTTGCGC ECLWFLR 1

TGGGCCCAGGTTGCTGCTTTG WAQVAAL 1

ATGGCCGCGAAGAGCGCCCGC MAAKSAR 1

CCCTGGCAGAGCAGCAACAGG PWQSSNR 1

GGGCGGGTTCACTACTTTGTT GRVHYFV 1

TGGGTTATCGCGTGGGACCGT WVIAWDR 1

GTCCGGGGTTCTACGAGTATT VRGSTSI 1

GGCGACATTGGCAGGCTCGCG GDIGRLA 1

ATGGCGTTGGTCGTTCTGGTG MALVVLV 1

TGCTGGGGGTCTGCCAAGCGT CWGSAKR 1

AGTCGCACTCGGCCGATTACT SRTRPIT 1

GTGAGGCTTATTGTCGACCCG VRLIVDP 1

CTTGGTATGGTGGTCCTGTTG LGMVVLL 1

GGTCGTTTCGGCCTCGGGGAT GRFGLGD 1

CTCCTCGGCCACAAGCTGCCC LLGHKLP 1

TTCCCTCCCTCGGCCTACCGG FPPSAYR 1

GTCCGGCTCAAGGCTGCTGGT VRLKAAG 1

GGGTTGGGGGATGGGAGCGCG GLGDGSA 1

AGTGTGTCTAGGGACAGGCCT SVSRDRP 1

TCGTCCCTGAGCTTGGTGGGT SSLSLVG 1

GGTCTTTACACGGAGGCGTTT GLYTEAF 1

AGTGAGTTCGGCGCGAACGCG SEFGANA 2

GGTGTGCCTCAGTGCATTGAG GVPQCIE 1

TTTTTGCCGGAGCCCGCCTAT FLPEPAY 1

GCCCAGAGTAGTGCGTGGGCT AQSSAWA 1

CAGGGGATTACGGGTGGTTGT QGITGGC 1

TATGCGGACTGGCTCATTCGC YADWLIR 1

GGGGTTTGGTGCTATGTGTAT GVWCYVY 1

GCTGGTTGCCTCCAGTCGGTT AGCLQSV 1

AGCTTGTTCTATCAGGTTTGT SLFYQVC 1

CACCGCGTTCTTGACCGTTTG HRVLDRL 1

GGGGCTTGCGTGTTTGATTCT GACVFDS 1

AACGAGCGCTCGGTTTATTTG NERSVYL 1

GTCGGGCTGTCGAAGGTGATG VGLSKVM 1

GTGACCGGCAGTCTGTATCGG VTGSLYR 1

GTCTGGCGGCATGGGAGGGGG VWRHGRG 1

TATCTCGCCACGCGTAGCGTT YLATRSV 1

TATACCGGGAGTCGCGGTAGT YTGSRGS 1

GGTCGTAGGGTTGAGTCCGCG GRRVESA 1

CAGCCGGTCCCTGCGCGGGTT QPVPARV 1

CAGTTCGGTGGCGGGGGCCCT QFGGGGP 1

TCGATTGTCAACTTGGTTCCG SIVNLVP 1

GCTGTTGAGACGTTTGCTCCG AVETFAP 1

TGGGGTCTGCTTTGGTATCCC WGLLWYP 1

CTCAAGTGCACGCGGTCGCTG LKCTRSL 1

TTCGGTCCCCGTGACTATCGT FGPRDYR 1

CATGGTGGGTGGAGGAACCGT HGGWRNR 1

CGGCGGGTGGACGCTTTGGTG RRVDALV 1

GGCTGTATGTTCTTGCAGTGC GCMFLQC 1

TATGTGGTCAAGTGGAGTGGT YVVKWSG 1

TCCCGCGCCATTTACCATGCG SRAIYHA 1

AATGTGGGGGGGTGCGCGTCT NVGGCAS 1

CGCCCTGTTCACGGGATGTCT RPVHGMS 1

CCGCGCTGGTCTGACATGTGT PRWSDMC 1

GCTAGTCAGTACGGTGTGAGC ASQYGVS 1

GTGTGTCAGGTTTGCAGCCTC VCQVCSL 1

CGGGGCTTGTGGTTCCAGTCT RGLWFQS 1

TACTGGGCCCAGTTGCCTCGG YWAQLPR 1

GAGAGCGTCGAGGCGATGCTC ESVEAML 1

CATTCGGATGGGATCCGTGGT HSDGIRG 1

TTTCTTGGTATGGGCCGTGGT FLGMGRG 1

CGCCTGTCCAGTGCCGAGTGT RLSSAEC 1

TATTACTGGTGGACCCCGATT YYWWTPI 1

GTGCGGTTTAAGGTGGGGCTG VRFKVGL 1

GTGGTCACTGAGTTCGTCATT VVTEFVI 1

GATTCGGGGGGCTTGGACCCG DSGGLDP 1

CATCGTCGCAGTGCGATTGCG HRRSAIA 1

CTGTATATCCTTATGTGGGAG LYILMWE 1

GTGGGGCTGATTCCGGGTTTG VGLIPGL 1

GCTTTTCGCAGCAAGTGTAAG AFRSKCK 1

TCGAAGCAGGACGGCGTCTTC SKQDGVF 1

TTCGAGGCCGGCGCGTTGGAG FEAGALE 1

GCGTGTGTCGCGTGGGTCCTT ACVAWVL 1

ATTGTCGGGTTCGCGCGTACT IVGFART 1

GGTTCTTATAGGTCTGGTAAC GSYRSGN 1

GACTTCCGCGGTGCGGGGTGT DFRGAGC 1

TGGGATTCCTCTCCGAGCACC WDSSPST 1

ATTGGCCGCGGGTCCGGTACC IGRGSGT 1

TCGCGCATGCGGGCCAGCCAG SRMRASQ 1

GATCGTAGCTGGATGCCCGTG DRSWMPV 1

CCGCTGACGTACTCGGAGCAT PLTYSEH 2

CGCGTTGGGGCTCTTCTGCGG RVGALLR 1

CCGTTGTTGTCGGATCGGCTG PLLSDRL 1

GAGGCGTTGCCTGGGGTTAAG EALPGVK 1

GTCGTGCCTCGCGTCGATTTG VVPRVDL 1

GTGCTTCGGGTTGTTTTGTAT VLRVVLY 1

GCGCACGTTAGGGCTGTGAGT AHVRAVS 1

GGGTTTGGTCGGCCGTTTCAG GFGRPFQ 1

GTTGTGTATGTTGCGTTCCCT VVYVAFP 1

CCCGTTATCTTGTCGGGGTAT PVILSGY 1

TCTCGCTTTTGCCTCGGCCCG SRFCLGP 1

TTGTGCGAGAGGGACCTGCGG LCERDLR 1

TGGTATAGTCTGATTGGCGCT WYSLIGA 1

GCCAAGGACTGGGTCAGCCTT AKDWVSL 1

CTGACCGGGACCGTCATTCCC LTGTVIP 1

CTCTACGGGACCGGCTCTCGG LYGTGSR 1

GGTGCGCGCACCGTGGTGCAC GARTVVH 1

CGCTATGGGCGCTGGGTGCGG RYGRWVR 2

GTGAGCAAGGTTCCGCGGCTG VSKVPRL 1

TTGTACAGGAGTGTCCTTTCG LYRSVLS 1

GCCGGGTCGCGGTTTGGGTTG AGSRFGL 1

AAGGGTCGGGTGCTCCTGCAT KGRVLLH 1

GGCCCGGTGTTGGGGAGCCAG GPVLGSQ 2

CTCCACTTGGACCGGGAGTAT LHLDREY 1

GAGTTCCGCAGGCTTCGCTCG EFRRLRS 2

GAGCGTTGCGGGCAGTGTGAG ERCGQCE 1

TGCTCTGTTCGGGCTGTTGGG CSVRAVG 1

CGTGGGGTTATCTCCAGGGGT RGVISRG 1

CGGGTTCCTCATATTCCTAAG RVPHIPK 1

GGGTGTTGCAGGAGCTCTAAT GCCRSSN 1

CCTTTTTGCGGTGGGGCGGTT PFCGGAV 1

TTCCTGGGTTACAGGAGTGAG FLGYRSE 1

CTCATGGACCCCGGGTTGGTG LMDPGLV 1

TTTCTGTTGTTGTGCTGTCAG FLLLCCQ 1

TTGGGCCGCCTCGGCTATATG LGRLGYM 1

TCTCGTATCGCGGTCCTCTCT SRIAVLS 1

GTTTGTGTCAGCTGGAGTGGC VCVSWSG 1

GTGCGGAGCTGCTGGTACCGC VRSCWYR 1

GCCCAGGGTCATGGGCAGTCG AQGHGQS 2

TCGCGTAAGTGCTATAAGTAT SRKCYKY 1

TACGATCGGTGTTGCAGGAGG YDRCCRR 1

GGCAGTGTTTCGAAGATGTTT GSVSKMF 1

TCTAAGGTTCTCGAGATTGGT SKVLEIG 2

TGGCAGTTGAGCGGCCTGCAG WQLSGLQ 1

TGGTGTGCGTTCACCATGCTG WCAFTML 1

TCCCTTCGCGCCCCCTCGACG SLRAPST 1

TGGAAGCTGGATGTGGTCCGT WKLDVVR 1

GAGTCGTGGGACTGCGCGCCG ESWDCAP 1

ACTAGTTCGCGGCGCGTTGCT TSSRRVA 1

TCGTGGAGTATCTGGTGGGCT SWSIWWA 1

TGCGTCTCCTCGGCGACGCAT CVSSATH 1

GAGTGCACTGGGCCGAGTTGT ECTGPSC 1

ATGCTCCGGGCCGGTGGTCTC MLRAGGL 2

CCTTGGTCGGTGATCCTTACG PWSVILT 1

AGTTGTCTCGTGCTCCAGAGG SCLVLQR 1

AGCTTGCGCAGGACCGGTGCT SLRRTGA 1

GGGGGGAATTGTCTCGATGCT GGNCLDA 1

GTGGCGCCGCCGGACCTTTCG VAPPDLS 1

GTGGGGAACTCTAGGTGTCCG VGNSRCP 1

TACCACGTGTCGCCGTGCTGT YHVSPCC 1

GGCCGCTGCACGCTCTACGGG GRCTLYG 1

TGCTCCATCGGGTGCTGTGTT CSIGCCV 1

AGGCGTTGCTTGGGCAGGGAG RRCLGRE 1

ATTTGCGGCCAGAGGGAGCGC ICGQRER 1

GATCTGGCGAGTGATATGCCG DLASDMP 2

ATGGTCGGCTACGCTAGCGGG MVGYASG 1

TTGCAGCTCACGTCGGTGATG LQLTSVM 1

CACCTCTCTGCCGACCGCAAC HLSADRN 1

GGGCGCGACAGGGAGGAGTCT GRDREES 1

CATCGGTACCCGACGTCTGCT HRYPTSA 1

GCGCGGGCTTTGGATGCGACT ARALDAT 1

GCGGCGCTGAGGGTGATGTTT AALRVMF 1

AAGTTGTCGGGCTCGCTCTTT KLSGSLF 1

TGTTACTTCCTTGGGCGGCCT CYFLGRP 1

CGCCAGGGGGGGAACGCTGAT RQGGNAD 1

CTGTCTTTTCGCCTCCAGCTT LSFRLQL 1

TTTAGTTGGTTTCGGCCGTTG FSWFRPL 1

CTCAAGGTCCACGTGGGGTTG LKVHVGL 1

CGGGGCTGGGGCAATGTCGGT RGWGNVG 1

GGGACGCAGAGGTCTCGGGCT GTQRSRA 1

GTTGTTCGGGCGGGGTGGCGT VVRAGWR 1

TTGGGGCCGACTGGCCTTGCG LGPTGLA 2

TGTTGCTTCTTTGGCGGTAGT CCFFGGS 1

GATCTGGCTTCTACCGTCGGT DLASTVG 1

TCTTCTACGACTGGCTTGGTG SSTTGLV 1

CCCAGCCGGGGTCGGTCGCCT PSRGRSP 1

GCGGCTCGGACCGCGATTTGT AARTAIC 1

GGGGGTGGCTTTGCGGTGAAT GGGFAVN 1

AGTTGTGTCAGGACTCGTACC SCVRTRT 1

GGTGATGTGAAGGGCGCTGCG GDVKGAA 1

TTTAGCGAGCACTCGGTCCCT FSEHSVP 1

GTTACTACGCGCCTGATGACT VTTRLMT 1

CCTGGGAGGCTCTTGTTCTCG PGRLLFS 2

GACGGGGAGTGTAGGGAGCCT DGECREP 1

CGCGTGGTCGGCTCCAGGGTT RVVGSRV 1

TTTTACTACGTTATGAGGCTT FYYVMRL 1

CCTTTTACTGGGCGGATGTTC PFTGRMF 1

TGCATCGTGGCTTCTGTGGCT CIVASVA 1

ATCTGCGGTTGGTTCCCGAGT ICGWFPS 1

TGTAAGGGCTACAGCGGCTGT CKGYSGC 1

CCTGTTTGCTGCAGCGATGCG PVCCSDA 1

CAGGCGGAGTGCTGCGATTAT QAECCDY 1

CTTTGGTCTACTCTGCTTCTT LWSTLLL 1

GATCGGGGTCCGTCTGTTTCT DRGPSVS 1

AGGGCTGGTAGTCTGGGGCTT RAGSLGL 1

GTTGGCTCTTCGGATCTTGGT VGSSDLG 1

GCGCTGGGGATCTTTCGTAGG ALGIFRR 1

GGCGCGCGGGGTTTGCAGCGT GARGLQR 2

AACTTCCCTAAGGCCGATTTT NFPKADF 1

AACCTGCTCGGCGTTGTTCTG NLLGVVL 1

TATCGCTGCCGTGCCGGGAGG YRCRAGR 1

GGGGTCGACAGTTGTTGTACG GVDSCCT 1

CACGGTCGGGACCACCGGGTT HGRDHRV 1

TGTGTTCGGTGTGATCCCAAT CVRCDPN 1

CCGGGGGGCGACGTGTCCCCG PGGDVSP 1

GAGATGATCGAGGTGCCGCCG EMIEVPP 1

TGGTGGAGCCTCGCGCATTCT WWSLAHS 1

GTTTTGGTGGGTTCGTCGGGC VLVGSSG 1

CGGGTCGCGTGCGGGGGCATT RVACGGI 1

TCTGAGTCTAAGCTGGCGGGC SESKLAG 1

GAGTGCGAGTGGAGCGGTTCT ECEWSGS 1

GCTCCGGGTGTCGTCGACCTG APGVVDL 1

ATGCGCGCGGGGTCCCTTCCT MRAGSLP 1

TTGAGTAACTCGTGGCTTACT LSNSWLT 1

TGGCTCGGTTTGTTGGGGGGT WLGLLGG 1

CTCCCGTTCTCTGGGTATCTG LPFSGYL 1

GGCTGCTACGCCAGGGCGACG GCYARAT 1

CTTGCCTTGACTCAGAGGAGT LALTQRS 1

GGGAGCGCCGAGACGGTTCGG GSAETVR 1

ATGTGGTATGGGGTGGCTCTT MWYGVAL 1

GTGCGGAAGCATGTCATGTCT VRKHVMS 1

AGTGCTTCGTACGCGCTGTTC SASYALF 1

TATGGTGAGTCGGAGTATTAT YGESEYY 1

TCGGGGAGGGACAGTTCCGCG SGRDSSA 1

GTTGGGTCTGGTCGGCAGATT VGSGRQI 1

TTGGCGCACTTTGACTGCTTT LAHFDCF 1

CGGCGCGTCCGCACGGAGAAT RRVRTEN 1

GATCTTAAGTCGGCGTTCCTG DLKSAFL 1

TGGGCCGGGACGCTCGAGGTT WAGTLEV 1

ATGTCTGCGAGGAGGGAGTGG MSARREW 1

GTGGCCGTCTGGCTGGGTCGT VAVWLGR 1

ATCAAGTCGGTCTACGGGGGG IKSVYGG 1

AGGCTGCGGATGGGTCTCCGG RLRMGLR 1

TTGGCCTGCGGGGTGGTTTGT LACGVVC 1

CGTTCTGCCGTGTATGCGAAC RSAVYAN 1

GGCTGGATTCGCTTTGGTTGC GWIRFGC 1

GTTGGGCAGCACCGCGGTACC VGQHRGT 2

GTCTGGGAGCGCGGCGTGCGG VWERGVR 1

GGGCGGTACAGCACCGGCCGG GRYSTGR 1

GTTCGGGTGGTTCCCACGGCT VRVVPTA 1

GGTCAGCCCGGGAGGTTGCGT GQPGRLR 1

GTCAGGTCCGCTTACGTCGAG VRSAYVE 1

CAGCTCGGTCACCCGGTTATG QLGHPVM 1

TGGCTCTGCAACAGCTGGAGT WLCNSWS 1

TTGTGGCGGGAGCTTGCTTCG LWRELAS 1

TCTTGTCGGTGTTTCTGGGAT SCRCFWD 1

TGTTCGTCGGGCCATGGCTCC CSSGHGS 1

CCGGGCTGGTCTGTGGAGCGT PGWSVER 1

GTTGTTCATAGGGCCAGGGGT VVHRARG 1

ACGACGTTGTTGCGCTCGGGC TTLLRSG 1

CCGCCGATCTGGGTCGTCTGT PPIWVVC 1

GTCTGCGAGTCGCTCGTCTGG VCESLVW 1

GGCGGTGACAGGTCCAAGCTC GGDRSKL 1

TCGGTTCGGCGTGGCGTTCGG SVRRGVR 1

GGGTTGGGGTCTCCTATTGGT GLGSPIG 1

GGCCACCGTATCGCGCACGGT GHRIAHG 1

CGGCGGTGGGACCATCCGGAT RRWDHPD 1

GACGGGTTTACCGGCACGGTT DGFTGTV 1

AGGGCGGACGACTGCAGGGCG RADDCRA 1

ACGCAGATTGCGTTGGACGGT TQIALDG 1

TCGGTCCGGTCCAGCTTCGCT SVRSSFA 1

GCGGAGGGGCTGGTTCTGGGC AEGLVLG 1

GGGGGTGGGTCCCTCATTCGG GGGSLIR 1

GGGTTTCAGGAGACGGGCTTT GFQETGF 1

TGGGACCTTGGGGTCGCGCAG WDLGVAQ 1

GTTAGGTTCGTCATTATTGCT VRFVIIA 1

CGTATGCGGGCTTATGGTGTG RMRAYGV 1

GGGGCCGCCTGGTGCATTTGG GAAWCIW 1

GCGTTGCTGTCCAATCTTAGG ALLSNLR 1

GGGAGGTTCGGGTCGGTTTCT GRFGSVS 1

GGGGCCAAGCGGTGCGGTTCT GAKRCGS 1

TACAGCCCCGAGGATCTCCCG YSPEDLP 1

ATGGACGCTAGTTGCGGCCCG MDASCGP 1

TTGGCTGCCGTCATCCAGGGT LAAVIQG 1

TTGCAGAGTTGCCAGACTTGT LQSCQTC 1

TACTTCGAGATCTCCTACCCT YFEISYP 2

CAGCTGCCGCTTTGTAACGTT QLPLCNV 2

TATCTGCCCCTGTGCCTGATC YLPLCLI 1

GGCCCGCTGAGGGGGGTTCGG GPLRGVR 1

TGTTTTATGCGGTGGCCCGTG CFMRWPV 1

CGTTCTAGCGTGCTCGGCGTG RSSVLGV 1

TGCGCGATCTCGTCTGTGTCG CAISSVS 1

TGGGGGCGGTTTCGCGTGCGT WGRFRVR 1

TTTGGGTTGGTGACGGTGAGT FGLVTVS 1

GGGCGTAGGTATCGGGTGCAG GRRYRVQ 1

GACGGCGTGCTTATTGTGCTG DGVLIVL 1

CTGTTTACGCGTTTGCAGAGT LFTRLQS 1

GTGCCGCGGCTCCTCGATGGT VPRLLDG 1

GAGATGCACGGGTTGCGCCGG EMHGLRR 1

AGGTGCGTCGTCTCCGTTGGT RCVVSVG 3

CAGCCCGAGGTGTTCGTTCGC QPEVFVR 1

GCGCGCGACCATGGGACGCTT ARDHGTL 1

ATCAATGACAGGCTGGGTGCG INDRLGA 1

CCCGTGAGGTATGCTGTTGAT PVRYAVD 1

TGGGCGGGCTCCGGTGGGCTG WAGSGGL 1

TGTGGGCGGGTGGAGCACGTT CGRVEHV 1

ATGCTCATCAGCCTCAGTCCT MLISLSP 1

TATCGTGGGGTCCTTGCCGCG YRGVLAA 1

GTTACTATGGGCAGCCGCTCT VTMGSRS 1

TCGGGGATGATGGCTGCTCTT SGMMAAL 1

GCGATTCCTATGCGTGAGGCG AIPMREA 1

AGTTCCACCAGCTTTTGGCAT SSTSFWH 1

GCGCTCCGTTCGTGCGCGAGG ALRSCAR 1

TTCAATCGCTCGTGCGTGGTG FNRSCVV 1

AAGGAGAGTCCTGCGTGCTCG KESPACS 1

TTGCATGGCTCTGGGATCGTT LHGSGIV 2

CAGAGGCTGGTTTCGATGCTG QRLVSML 1

CTGATCGCGAGCAGGCTGACC LIASRLT 1

CACGTGCTGTCTGCGATCTTG HVLSAIL 1

ACCGTTGGCACCGAGAGTGTG TVGTESV 2

TGTAGTACGCGCGCTCGTACC CSTRART 3

TCCCGGTTGTACGCTCCGCCT SRLYAPP 1

TACGGTAGGAGGATTCGGTGC YGRRIRC 1

AGGCCGCGCGGTTGGAGGGTT RPRGWRV 1

TCTGGCTTCGCTAAGCATTTT SGFAKHF 1

ACTACGATTGGCCGTCGTGAC TTIGRRD 1

AGTGCGATCTGCAGGGAGTCT SAICRES 1

AGCCGGATTAGCGTCGCTCCT SRISVAP 2

CGTCCGGCGCGGTGTCTCCGT RPARCLR 1

CGTTGGGCTAGGCCCTCCTCT RWARPSS 1

GGTTCCAAGCGTCTGGGTAAG GSKRLGK 1

GAGGCGGGCCTGATTCGGCTT EAGLIRL 1

GGGTTGATTAGTCGGGCCGCG GLISRAA 1

ATGTTGGTTTTCGCGTGTGTT MLVFACV 1

CTCCCGGTGGTGGGGCACGGC LPVVGHG 1

GAGACCCTTAGCGTCCCTCCG ETLSVPP 1

TTGTCCCGTGGGAGGTTGCGG LSRGRLR 1

TCGGGCCCGACTAGCAATTGG SGPTSNW 1

CTCGCTGATTGCATTTCGGCG LADCISA 1

TCCTCCTGGACGTGTATTCGT SSWTCIR 1

CGCGCGATCCCCTTTCTCTCT RAIPFLS 1

GGTCGCAAGGTTAGCAGGTCC GRKVSRS 1

CACTGCGACCGCTGTGGGGCT HCDRCGA 1

GGGCGGCGCTGCCGGGACTCG GRRCRDS 1

TGCATCAGCGAGTTCGGGCGG CISEFGR 1

TATGGGGTTCTTCTCGTGACT YGVLLVT 1

TCCGTCGCTATCGCGCAGCTT SVAIAQL 1

GAGTCCGTTCTGCGGCCTCGG ESVLRPR 1

TGGGGTTCTTGTGACGTGTCT WGSCDVS 1

GATCTCGGCATTGTCGCGGAT DLGIVAD 1

AATCTTAAGAGGGGGGGTCGT NLKRGGR 1

TGCCGCGTTTACCAGTTCACT CRVYQFT 1

CACGGGGTTATGGTTGTGAAT HGVMVVN 1

GGGGGGCATCCGGGTTTTGCT GGHPGFA 1

ATGCTTGAGTCGGTCTACCCT MLESVYP 1

GTGCACGTTTGCGTCGATGGG VHVCVDG 1

AAGCCCGGGACTATGCGTCGG KPGTMRR 1

CGGTGGCGGAGGGTTGTGGGT RWRRVVG 1

GGTTATACGAACCGCGGTCGG GYTNRGR 1

CAGGCGTTCGCGTTGAAGTGC QAFALKC 1

GATTGGGACGTCGCCCAGGTC DWDVAQV 1

GAGGCGCGCTCGTTGTTCTGC EARSLFC 1

CAGCTGTTCACGGTCTTTGAG QLFTVFE 1

TGCGGGCAGCAGCGTGAGGGC CGQQREG 1

TGTCTGGTGTGTCGCAGGCCT CLVCRRP 1

GTGAGGTTCTGGGATTCGCTC VRFWDSL 1

TTCTCTAGGACTGGTCTTCTT FSRTGLL 1

ACGAGGGGCTCTGTGGAGAGG TRGSVER 1

GAGGTCTCGAACGCGCCTGCT EVSNAPA 1

CTGGTCGTCACGGGGTTTCTG LVVTGFL 1

TTTCGGGGGTCCGGTGACGGG FRGSGDG 1

GGCGCCAGGGGTGGCGCGCGT GARGGAR 1

TATCGGGTTCGGGCCTATTCT YRVRAYS 1

AGCGGGGACAGCGCGCGCGCG SGDSARA 1

TCGTGGCTTTCGACCAAGTGT SWLSTKC 1

TTGTGCGTGAGCAGGAAGAGG LCVSRKR 2

GTCAGGTCGCTCGAGTGTTGT VRSLECC 1

CCTCTTGTCTTGGCCTCGAGT PLVLASS 1

TTGCGGCTCTACTCTGGGAGT LRLYSGS 1

GCGGCCAACTCGGGCATTCCT AANSGIP 1

GGGGTCTGCAGCGGCCATGTG GVCSGHV 1

GACTTGGATCTGGGCGGCGCT DLDLGGA 1

TTGTCGGCCGGTACCGAGGCG LSAGTEA 1

ACGCTGGTTATTCTTTGTTGT TLVILCC 1

TGTGTTAGCTTGCGCCATGTT CVSLRHV 2

TCGGACCTGCTCCGTATGGCG SDLLRMA 1

TGTATGCGGAGCATCCGCCGG CMRSIRR 2

ACGTCCTGCGTGTGCTTGCCT TSCVCLP 2

TATTTCAACGTTGAGAGCGCG YFNVESA 1

CTCTCTGGGAGGTGGGCGTCT LSGRWAS 1

ATCCGTGCTTACGGCCGCGGT IRAYGRG 1

AATCGCGGCAGTCCGTGGTTC NRGSPWF 1

ATGTACCCTCCGTGGCGTTTG MYPPWRL 1

TCTCGGGTTAGCACGACGTTG SRVSTTL 1

GGGCTGCGGAATTGGCTGCGG GLRNWLR 1

CGGCTCATTAAGGTGGCGGTG RLIKVAV 1

GGGCGCAGCTTTGGCTGGGAT GRSFGWD 1

GGGGACTACTCGCCGCCGCGG GDYSPPR 1

AACCCGTCTGTCTTCGCGGTT NPSVFAV 1

GTTGTGGCGTCCGTCAGCCTT VVASVSL 1

TGGGAGCTTATGGCCGACCGG WELMADR 1

TGTCGGTATTCTATCCGGGTT CRYSIRV 1

TACATGTCGGGCGATGGCCGT YMSGDGR 1

CTCGGGGTGATGTCCGCTATG LGVMSAM 1

TTCGTCGAGAGGCGGGCTCTT FVERRAL 1

GGGCGTTGCAGCTGTGGTTCT GRCSCGS 1

TCTATCGTGCAGTTGGGTAAT SIVQLGN 1

GCGCAGGGGGGTGTTATGGTT AQGGVMV 1

CGTGTTCGTACCGGTTTTAGG RVRTGFR 2

TGGTATGTGCATATTATGTTT WYVHIMF 1

TGTGTCAGCGTCGGGTTCGTG CVSVGFV 1

CCGCTGCAGCGGTGCGGTGAG PLQRCGE 1

CCGACCCCTTGTCCCGGGCGG PTPCPGR 1

GTGTGTAACTCTGGCCGTGTC VCNSGRV 1

GTTCTCGATCTGCGGTCGGCG VLDLRSA 1

TGGGGGCAGATGCAGTGTCGG WGQMQCR 1

AGGGATGTGGCGCACGGCGCT RDVAHGA 1

GCCTACCTGTTGGCTCGCCGG AYLLARR 1

CTTTGCCTCAACCAGCTCGGT LCLNQLG 1

GCTGAGGATATCGGGGCTCCG AEDIGAP 1

TGGGCCGCTTGGTGCCTGGGC WAAWCLG 1

TTGGATCTCACTGACCATGGG LDLTDHG 2

CGTGCCCTCCTCCTCCTTTTC RALLLLF 1

TGGACGCTTTCGGAGGTTGGG WTLSEVG 1

AATGTGAGGATTTGCTCTCAG NVRICSQ 1

ACCGCGAATGCGGCCCAGGCG TANAAQA 1

AAGTCTCGTTGTCGGGAGCCT KSRCREP 1

GACGTGCGGGGTGATAGGCTT DVRGDRL 1

ATGGCCTCGTGCAGTGGTCTG MASCSGL 1

TGGCTTTCTCACATCAATAGG WLSHINR 1

GTCGGCACGGTCGACATGAAG VGTVDMK 1

GTGTACGGCGTCTTCCTGGGG VYGVFLG 1

GAGTCTGTTAGCTGGGCTAGC ESVSWAS 1

GGGTGGCAGCGTGCGTGGTGT GWQRAWC 1

CGGCTCTTTCCTTTGGTGTCT RLFPLVS 1

GCGTCTGGTTCTTTGTGCCCT ASGSLCP 1

TGGCACGCGTGTCGGGTGATT WHACRVI 1

CTCGGGCGTTACGCGGATCCG LGRYADP 1

GGGAGGCTTTCGAACTGTTTT GRLSNCF 1

TGGGGTGGCAGCTATCGTGGG WGGSYRG 1

TCTGAGTATCGCGGCAGTAGT SEYRGSS 1

ACCGTGCAGTGGATTAGCGGG TVQWISG 1

CCCGGGGAGCGTGTGTACTGG PGERVYW 1

GGGCTGTGCCACCTGGGTCCC GLCHLGP 1

GCTAGGGAGAGGTGGGACAGT ARERWDS 1

CGTACGCTGTCGAGGGGTCGT RTLSRGR 1

GGCTTGGGCTACTGGGGTTGT GLGYWGC 1

CTGCTGGGGCAGGGTACGCAT LLGQGTH 1

TCGGCTTGGGCCGATGAGTAT SAWADEY 1

AGCCGTGTGCCGTACCCGAAC SRVPYPN 1

AAGCAGCGGCGCTGGTCCTCC KQRRWSS 1

TTGGCTGTTTTCTGGGTGTCG LAVFWVS 1

AAGTTGTCCGGCGAGTGTTCT KLSGECS 1

CGCGCGTGGTTGCTGAGTGAT RAWLLSD 1

GGGGTTCGGCACGTTGCCACG GVRHVAT 1

TGTGTGCGGGGTGCTGGTGAT CVRGAGD 1

GGCTTTGGGAATGCTGGTCTT GFGNAGL 3

ACCAACGGGTGCGCCCGGCTG TNGCARL 1

GAGCATTCTCATTTTATGTGG EHSHFMW 1

GCGCGGGCCGGTCGGAGGGAT ARAGRRD 1

TTGTGCGCGGTTCGGTATCGC LCAVRYR 1

GTGGGGATGTTTATCGCTGCT VGMFIAA 1

TCCCTTTGTGCCTCGGTTCCC SLCASVP 1

AGTCCGCGGTGCAGCTGGTCT SPRCSWS 1

TTGGTGCTGGCCGTCTGGCTG LVLAVWL 1

AAGGCTCGGAGTTCGCGGTCG KARSSRS 1

AGGGGCTCTGCTGTGCGGGGT RGSAVRG 1

ATCGATCTTAGCTTCACCGAT IDLSFTD 1

TTTGTCGGCCGCTCGGTGGGG FVGRSVG 1

CCGCGCGGCCTCGGCGTGTTG PRGLGVL 1

GGCTGGAGCGGCCTCGTGGCT GWSGLVA 1

CTGTATCTGATCCTGGGGTTC LYLILGF 1

TTGCGGGAGTCGCGTTCTCAG LRESRSQ 1

CGGCCTGTGCCTGCGTGCGCG RPVPACA 1

TGCATGACCTTCCTGGAGAAT CMTFLEN 1

CGGGCGTATGTGCTTAAGCGT RAYVLKR 1

TTGTGCGGGACGATGAGTTAC LCGTMSY 2

TGCCAGGCCGGGTGGCTCAGT CQAGWLS 1

GTGTGTGCGTGTAGCTTCGCG VCACSFA 1

CGTTCCGGGAGGGGCGATAAT RSGRGDN 1

CAGGGTAGCGGGGGCACTTGG QGSGGTW 1

CTTCTCATGGGCTCTTTTCCT LLMGSFP 1

GTTATTCGGTCGGACGATTCT VIRSDDS 1

ACGGGGGATAGTTGCTTGGGT TGDSCLG 1

CTCCCGCACAGTCGGACGTCT LPHSRTS 1

TACCACTGCAGCGACGCTCAT YHCSDAH 1

CTGTTGGATCAGTGCAAGGCG LLDQCKA 1

TACATCTACTGCACTCATGGG YIYCTHG 1

CCGGGGCGTTTTGGCCCGTCG PGRFGPS 1

GTGCGGCAGCGGAAGGCGTCT VRQRKAS 1

TGCGGGTGCACGTTGATTTCG CGCTLIS 1

CCCCACTGGACTTGGGAGGCT PHWTWEA 1

CGGGGGGTCAGTTCCGAGCTC RGVSSEL 1

GGGTCCCTCTGTCGGGCGCGT GSLCRAR 2

TATACTAACGGGTTTCTCTGT YTNGFLC 1

GACGTTGACAGCAACAGCTCG DVDSNSS 1

GCTAGTTTCCAGTGTGCTCTG ASFQCAL 1

GGGGTGGGTCATTATCTGCTG GVGHYLL 2

CGTACGGTCCCGGCGCTCAGG RTVPALR 1

ATCGGTGCTCTGCTCTCGCCC IGALLSP 1

ATTTCGTCCAGCATTGCGAGT ISSSIAS 1

CAGGTGGTTAACTGGTACCAT QVVNWYH 2

CCTCGTGTCAGCACGCTTGCT PRVSTLA 2

CGCTCTTGTTTTCACTGCGAG RSCFHCE 1

GTTTTGGGGCGCGGTCCTCGT VLGRGPR 1

TGCATCCGGGCTTTGTGTCCG CIRALCP 1

TTTCGTAGCGGGGGCACTTGC FRSGGTC 1

GCCCGCCAGCTGCGTGTTTCG ARQLRVS 1

CCCGGCTCTCTTAGTGTCTGT PGSLSVC 1

GTCGTGAAGGACGGGGTCTTG VVKDGVL 1

GGTCTGCCCATTCCTCGTACT GLPIPRT 1

GATGAGTATATGACCGATTGG DEYMTDW 2

CGGTGTGTCGTTTTGTGGTAT RCVVLWY 1

GGTGGTTCCTCGGCCGAGGAT GGSSAED 1

GCCATGTGGCTGGTGATGAAG AMWLVMK 1

GAGTACACTCCGTCGGTGGGT EYTPSVG 1

TTGTGGCGGCCCGTGATGCCT LWRPVMP 1

TGTTCCTTGTTGGAGTGCTAT CSLLECY 1

ACCATGCCGAGGTGGTCTAAT TMPRWSN 1

TTCCTCCTGCGGCGCGGCATG FLLRRGM 1

GACGGGTACACGGGTGTCGGT DGYTGVG 1

GATCTCTTTGTGCTCGCTGCG DLFVLAA 1

CGCGCGTGGTTCGTCATTAGG RAWFVIR 1

CTTTCTCGCAAGTACAGGTCC LSRKYRS 1

GTCCGTGCTAGCGTCCTGGAT VRASVLD 1

CCGGCGGGCATTTGGGGCCGT PAGIWGR 1

GAGGGCTGCCGCTTTCTTCGT EGCRFLR 1

GGGGCTTTGATCTGGGCCGTT GALIWAV 1

TGCCGGCCCTGTTACCCGTTG CRPCYPL 1

GGGGCGTCGTTTTGCGTGCCG GASFCVP 1

CCTCTCAGGGAGGGCTCTTGG PLREGSW 1

TACATGATCGTCGGCGGTGCG YMIVGGA 2

GCCGGCCACTGTGCTTGTTCT AGHCACS 1

TCGGAGAGTGGTCCGTGGGAT SESGPWD 1

TCTCAGAGCGGGTGGCGCACG SQSGWRT 1

TCGCGTCCGGGCTTCATTCGT SRPGFIR 1

GGTGGGCCCATCGGGGCGGCT GGPIGAA 1

ATGAGTACCTGGTGGTGGATT MSTWWWI 1

TCTCTGAACGTGGAGCTGACT SLNVELT 1

CGGACGGCTCTCACCTTCAAC RTALTFN 1

TGCTTCTGCGCCGGGTTTGTG CFCAGFV 1

CTTTTGCGGGCGACTTGTCAT LLRATCH 1

TGGTGTGATGCGGCTTGGATT WCDAAWI 1

CGTAAGCTCGCGCGGTGTACT RKLARCT 1

GCCTCCGGGAGGTGTTGGGCT ASGRCWA 1

GGCTCCATTAATGAGTTTGTG GSINEFV 1

CTCGATATTTGGGAGGTGTTG LDIWEVL 1

AGTTTCGGTAGGTGGTTTCCT SFGRWFP 1

CATTGGGCGAATGGGTTCAAG HWANGFK 1

TGCGGTGGGAGTCGCTACGGT CGGSRYG 1

AAGGAGGGGGACGGCTCGTGG KEGDGSW 1

CGGTATTCCGGGGTGGAGGTG RYSGVEV 1

GCCAGGGACAACGAGCGGCCG ARDNERP 1

TTTCGGCGCACTATGAGCACT FRRTMST 1

GCTGGCACCGAGCAGAAGCGC AGTEQKR 1

TCGCGGACCGAGAGCGCGGAC SRTESAD 1

ACTCTCGGTCCCAGCGCTGTG TLGPSAV 1

CCTCAGCGCTGCTGGAAGTGG PQRCWKW 1

TTGAGGTATCCGCATCAGATG LRYPHQM 1

TGCTGCTTCGCGAAGCGGGCT CCFAKRA 1

ATGAGCCATTCCGTGGGGTGG MSHSVGW 1

TTGTTTGTCACTGTCTGGGTT LFVTVWV 1

CTCGCTAGTTCGGGCTATTAT LASSGYY 1

AGGAGTGCTTCCGGTTTGGGC RSASGLG 1

GGGTGGTATCCTAATTCCCGG GWYPNSR 2

GTCTCCAGCCGTGAGCGCTTG VSSRERL 1

TCTGCGGTGGCCGTCACCATT SAVAVTI 1

CGGTCTGTGACGGCGCCTTGG RSVTAPW 1

AGTCGCACGTGTAGTACCCGT SRTCSTR 1

TGCCCCGCTAAGTCTCGTTTT CPAKSRF 1

GAGGCTGGCTGTTGTTGTGCT EAGCCCA 1

GTTATCCACGCGTGCGGTGTT VIHACGV 1

GCTGCGCTCGCCTGGGTGAGT AALAWVS 1

CGGCCCGGCGTTCCGCACGAG RPGVPHE 1

GGTCTCCTCTCGGACTGGCCT GLLSDWP 1

GTCGAGTCGTCCTCGAGGAGG VESSSRR 1

TGCGGTCAGCGCTCGTTTAGC CGQRSFS 1

AATCGGTGTGGGAATCAGCGC NRCGNQR 1

TTCAAGAGTGCGGCCCGTCGT FKSAARR 1

GTTTTTGTTAGGGCTTTGGAT VFVRALD 1

GGTGAGGCCGGTCCGGAGCGT GEAGPER 1

TGGCGTTTCGCGTTCATGGGT WRFAFMG 1

AGTCTCGGGCTCGAGCCGGGT SLGLEPG 1

GTGGAGCTTACTGGTGGTGTT VELTGGV 1

CTCGTTATTGACAGGGCGCTC LVIDRAL 1

GTCCTTGCGCCGTTCATGTCT VLAPFMS 1

CGCACGTGTAATATTTGTGTG RTCNICV 1

TCTAGCGTGGCGCAGTCCGTC SSVAQSV 2

TGTGCTCCCGTCCTCTGTGGT CAPVLCG 1

CGTTGGTGTCTCGGCGCGCCG RWCLGAP 1

GGGGCTGCTGGTGCGGGGACG GAAGAGT 1

CTCCCTGAGGGCATGTTCCAG LPEGMFQ 1

ACTCGCCGTACTCGGTATTGT TRRTRYC 1

CCTGTTGTGAGGGGGCATTGT PVVRGHC 1

CACTGCGGTGGCTGTAACTGT HCGGCNC 1

GCGCCTAGGAAGTCCGCTGAT APRKSAD 1

CATTCGCCGCTGAAGCTTCCG HSPLKLP 1

CTTCTTAAGGCCGTCAGCAGT LLKAVSS 1

ATTGAGCTTTTGAGCTGCCCG IELLSCP 1

TGCGTTTTGTATTCTGTGAGT CVLYSVS 1

GTGTACCGGGGCAGTTTTCCG VYRGSFP 2

TGTGGTAGTAGCGCCGGTCGG CGSSAGR 1

TCGAAGCGGTGTACGTTCTGG SKRCTFW 1

TGGTTTGTCTTGTGCGCGGCT WFVLCAA 1

TGGCTGGTGTGCCGGTGGGTT WLVCRWV 2

TCCAGGTGCAGCGAGGTTACT SRCSEVT 1

AGGTGGGTGAAGGCTGGGCTT RWVKAGL 1

GTCGATTGCATTTGTGTCAGT VDCICVS 1

AGTGGCCCTGTGCTGATTCCT SGPVLIP 1

AGTGAGGGTCTTGGTGGTATT SEGLGGI 1

CTGATTCGTCGTGGCGTGAGC LIRRGVS 1

CGCCAGCGTGAGTTTCGGCGT RQREFRR 1

GTGGGTTGCAGGTCTGACGGG VGCRSDG 1

CAGGGCGCTCTGGGTCTCCTT QGALGLL 2

GCGGCGGGCAATTGCGTTCGT AAGNCVR 1

GCCGTGCATAAGGGCTGCTCT AVHKGCS 1

TCGGCGGCGCCGCTGCGTCAT SAAPLRH 1

TGGTCTCGTCGCGTTTTCAAG WSRRVFK 1

GTCGGCTTGATGGGGATCGCT VGLMGIA 1

TTTTGGACTCAGTTGCAGCGT FWTQLQR 1

GTTATCAATGGTCCGTTGCCT VINGPLP 1

ATCAAGTTCTTTACTGTGGCG IKFFTVA 1

GATCGTGTGAGTTCCAGTCGG DRVSSSR 1

TCTACTGACTTTCAGCTGGTT STDFQLV 1

CACCTGGTCCCTAACATGGCT HLVPNMA 1

GAGGATGTGCTGTCGGTCTCG EDVLSVS 1

TGTCGGCGGGCCTGCTGGCCT CRRACWP 1

TCGATTTCCGGGTTCGTGCCT SISGFVP 2

TGTGGGCGCCTGGATAGTTTG CGRLDSL 1

AGTGGGATGCGCCTCGTGGGT SGMRLVG 1

ACGGGTTTGACGCGGTCCATT TGLTRSI 1

TGCGGGCGGAGGGCGCGGATT CGRRARI 1

GGTCGTACCGATCCGCATCCT GRTDPHP 1

GCGGTGGCTAATCAGGTTGCG AVANQVA 1

CAGCCGGACATGGTCATGCCG QPDMVMP 2

GTCACGTTCTGGATCCAGCGC VTFWIQR 1

CCGGTGCCCAGTATCTCGCGT PVPSISR 1

GTTCTGACTGGGACTTGCTCC VLTGTCS 1

CAGTACAATTGCGGGGGTGCG QYNCGGA 1

TCTATGGCGTGGAGGACGTGT SMAWRTC 1

GAGAGTATTAGGGTCCTGCAG ESIRVLQ 1

TGGCGCGCGCGGGTGTATCTT WRARVYL 1

ATCGCTGATCCCTGGGGGGTG IADPWGV 1

TTTAGGTGGGGTGGCGTGCAG FRWGGVQ 1

TCCGCGCTCGTTCGCTCGATC SALVRSI 1

CACCGGTATGTGGTTGGGCTT HRYVVGL 1

CAGTTGGCGTTCACGGCGTCT QLAFTAS 2

GTTGGTTCGGACGGGTGTCCG VGSDGCP 1

GGCCGTGGGGGTCGGTCGAAT GRGGRSN 1

AAGCTGCACTTGGCCGGTGGG KLHLAGG 1

GATTCTCGGCACGGCGGGTCT DSRHGGS 1

GTTCCCTTCAGCAGCGTCGCT VPFSSVA 2

GTGGTTCCGAGTTATGGGCTG VVPSYGL 1

TGGTTGCAGCGTCTCCGTCGT WLQRLRR 1

GTGGACCCCGGTTGGGCGCGT VDPGWAR 1

TGTTGTGGGTGCCTCTTGCCC CCGCLLP 1

GATGGCGGGGTGCCGCTGCGT DGGVPLR 1

GTCAAGCACGTGGAGCGCACG VKHVERT 1

GATGAGCGGAGGCTGGGTGCT DERRLGA 1

TCCCATGTCGGGTTTTTCACG SHVGFFT 1

GCGTTGTTGGGCTCCTGGCGG ALLGSWR 1

TGGCGTCGGAGCCTTCTTTAT WRRSLLY 1

ACGCATGTTACTGGCGGTCGG THVTGGR 1

TGCTTTTTCTGCCTGGAGAGC CFFCLES 1

GGGGTGAGCAAGTTGATGCTT GVSKLML 1

TATTATGGGCTTCCGAGTCGT YYGLPSR 1

CATAGCCGTGGGTACTTTGTT HSRGYFV 1

ACCTACGATATCCGCGCGAAT TYDIRAN 1

CTCTTGCTTTTTGGCTGGTTT LLLFGWF 1

CTGCGCTCTTGTCCTCCTAAT LRSCPPN 1

AGCATCGCGACTTCTCATGGC SIATSHG 1

CCGCGCGACGTGTTTGACCAG PRDVFDQ 1

TCGGTTCATTGCTACGTCTCG SVHCYVS 1

GCCTATGTTGGCTGGCATGTG AYVGWHV 1

TGCCGCGAGGCTGGTCTGTCG CREAGLS 1

TTGCCGAGGTGCTACGATTCT LPRCYDS 1

CTGTTCTGTACGGGGCTGCGG LFCTGLR 1

ATTCGGTCGCTGTGTAGGCCT IRSLCRP 2

TGCCCGAGCAGGCTGTGTGAT CPSRLCD 1

TGCGGGGCCAGGAGCGCGGTT CGARSAV 1

TCGTCGGCGTTCTTGAGGGAG SSAFLRE 1

TGGGGGCCGCGCGGTAAGTAT WGPRGKY 1

GGCGTTACCAGGACTTTGGAT GVTRTLD 1

GGGTGCATCTGGGACAGTGGG GCIWDSG 1

CTTTATAGGCTTGGTGATCCG LYRLGDP 1

GGTGTGCTCACTGTCGTGTGT GVLTVVC 1

GGGCTGTCTAGTCCTGATGTT GLSSPDV 1

GTTGCTACCAGGCGGCTTCGT VATRRLR 1

GTGCACCGGGCTGCCGTTGTT VHRAAVV 1

GTTTTGGGCGTTCGCTACCTC VLGVRYL 1

AACTGTCGGTCTGGCAGCTCT NCRSGSS 3

GCCTGGGGCGTCACGTTGGGG AWGVTLG 1

CTGTTCGCTGGCAGGGGTCCT LFAGRGP 1

TACAGTGGCAGGCGGTATAAG YSGRRYK 1

TTCCTGCACACGGGCGTTAGC FLHTGVS 1

TACAGGCCGATTCAGTTGGCT YRPIQLA 1

AGTTCGGTTCCGGCTGGCTCT SSVPAGS 1

TGCAGGCTGTACTTCAGTGTT CRLYFSV 1

TTCATTCACCGCGGGGGCGCG FIHRGGA 1

TGGGCTCACGTCGTGGGGGTT WAHVVGV 2

CAGTACAGTCGTCGGCCCGCG QYSRRPA 1

GTGTTGCGCGTTACCACTTCT VLRVTTS 1

ATTATGAGCCAGCTTCCTGGG IMSQLPG 1

GCCTGCGGCGCGCTGGGCTCT ACGALGS 1

TGTCAGACGGCGTGGCTGGGG CQTAWLG 1

CGGTTCTGGCTTCATCTGGTG RFWLHLV 1

CGCGGGACGTTGCTGAAGTGT RGTLLKC 1

TGCTTTCTCATCGGTCTCCCG CFLIGLP 1

GCGCTTAGCGGTGGCCGGAGT ALSGGRS 1

AACGCTTATTGTGGGCTTTGG NAYCGLW 1

GCTGAGTACACTTATGAGACT AEYTYET 1

GTGCGTCCTAGTGGCGGTAGC VRPSGGS 1

TTGCTCAGTCGGACCGCTTTT LLSRTAF 1

AACCGGGCGTTCAGGGTTTCC NRAFRVS 1

TGCTGGGCTGCCACGCGGTTG CWAATRL 1

GAGGGTTTGAACTATTTTTCG EGLNYFS 1

ACGTATCAGTCTGGCGCGTGG TYQSGAW 1

TATCGCGCGTTTTACTTGCCG YRAFYLP 1

TGCGTTCGCTGTAGCCGGGTT CVRCSRV 1

GGTCCCCACTTGTCGGCGTCG GPHLSAS 1

GCTGATTTTTTGGTGGGGGAG ADFLVGE 1

CTGGCGGAGGCGCATAGGGCG LAEAHRA 1

TGTTTTGACTGCGCGCGCGTG CFDCARV 1

CAGGGGAGCGTTAGGTATGAG QGSVRYE 1

ATGGCCAAGGCCTGGCTGCTG MAKAWLL 1

TTCGGCAGGACGCTTTCTCAT FGRTLSH 1

GGGGATCACCCTCGCAGCTTC GDHPRSF 1

GTCGGGCGGTCCCAGGGCCGC VGRSQGR 1

CCCGAGCGGGGCTGTTGGCCC PERGCWP 1

CACTACTGTGCTAGCTGCGCG HYCASCA 1

CGGGGCGCGACCAGGCTTCGG RGATRLR 1

AATAGTCAGCTTGATGTCTTT NSQLDVF 1

AGCTGCAATTGGGGCCAGCTT SCNWGQL 1

GGCGACGCTCACGCTCGCGGT GDAHARG 1

TTGGGTTCGACCGGGTTTATT LGSTGFI 1

TATTTGCGCACGAGTTGGTAC YLRTSWY 1

CAGCGGAGCTCGTCCAGTTGT QRSSSSC 1

TATGTGCCGCTTGACGGTGAT YVPLDGD 1

ATCTTCGACTCTACGCGGGGG IFDSTRG 2

GTCTCGGACAGTGTCCGGGCG VSDSVRA 1

TCTACCGTCTCCCGTGCCCGT STVSRAR 1

GGGTCGTTCCGTCGTAGCGGG GSFRRSG 1

GTTGCGTCGCTTGGGCTCTTG VASLGLL 2

GTGCGCGGCAGCTGCCATTTG VRGSCHL 1

TCGTCTGAGATGATGTGGCGC SSEMMWR 1

AACAAGCCCTTCTCCCTGCCT NKPFSLP 1

TTGCGCGGGGTCTTGGTTCGC LRGVLVR 1

CAGTTGATGAACGCCAGGCTC QLMNARL 1

CGGGCCCATAAGAGGGAGCTT RAHKREL 1

GTGTGGTGGTGTGTCTTGGGG VWWCVLG 1

AATCGCCGGTATCGGCTTGGT NRRYRLG 1

ACTCTTAGGAGGGATTTGGAG TLRRDLE 1

TACTTGGGGATGGAGGACGCT YLGMEDA 1

AACGCGTTCCCTAATGTTAGG NAFPNVR 1

CTCCATAGCGTTGCCGTCGGT LHSVAVG 1

TTGCCTCTTTGCGCCCTGATT LPLCALI 1

GCTGTGCTCATGGTTCCGGCG AVLMVPA 1

CGCCGCCCGTGCGGGTTTGGT RRPCGFG 1

GCGGTCAGCGTTAGGTATGTT AVSVRYV 1

TGTCTGGCCGTGGGTAGCATG CLAVGSM 1

GGGCTGATGGTGGTCATGGCG GLMVVMA 2

TTCTTTTGTGTTGGTCAGACT FFCVGQT 1

CGCTGCCTTCTGCCGGTGAGT RCLLPVS 1

GATTGTGGTTTGAACGACTCT DCGLNDS 1

TTCAGGGCGAGCAGTGGGGCT FRASSGA 1

CTTAAGGTGTTCGGGCTCGGT LKVFGLG 1

CTGCGGATGCCGAGCGGGATT LRMPSGI 1

GCTTGGGTCTCGCTGGGTTTG AWVSLGL 1

GGGCAGTTGGCGTCGCCGGGT GQLASPG 1

CCCACCCAGTTGGGGTGCCTC PTQLGCL 1

TGGTCGCATTTGTACTCTGAT WSHLYSD 1

TTTAGGACGAGTGCCGGTTGG FRTSAGW 1

TCTGATTGGACTTGCCGGTGG SDWTCRW 2

ACTCTGCTTCACTCCGCTCCG TLLHSAP 1

TGCGATGGGACGGGCGGTTGC CDGTGGC 1

GCCGACCTTTCGTATTGTCAG ADLSYCQ 1

CAGCAGGTTGTCCCGCGTCGG QQVVPRR 2

AGTTACTGGAGCCGTTCGATT SYWSRSI 1

TATCTGATTTCGTACGCCTTT YLISYAF 1

CGCCGGGGCTGGAAGTGGTGG RRGWKWW 1

GACCGGGATTCCAACCGGGTG DRDSNRV 1

GGTAATCCGAGCAGGCCGGCT GNPSRPA 1

GTCGGCGGGATTGTTGGCGGG VGGIVGG 1

GTTGATCCTATCCAGGGTGCT VDPIQGA 1

GGGCGGGATACGCAGTCGTAC GRDTQSY 1

GTCCAGGCTCCTCGCAAGTCT VQAPRKS 1

TGTCTGCACGCGAGGGTTATG CLHARVM 1

GGGTCCTTGGACATTGCTCGT GSLDIAR 1

GTCCGTGCCCGGTGTATCCGC VRARCIR 1

TGGCAGGGGCCCTGTGACGGT WQGPCDG 1

CTGTCGCCCAGTATCCCGTTC LSPSIPF 1

GTCAGGGGGACGCGGCACCCG VRGTRHP 1

GTGTCCCAGTGGGCGACGCAT VSQWATH 1

GACGGTACCAAGTCCGCCACG DGTKSAT 1

CAGAGCTTGCGGTTCGTTGGT QSLRFVG 1

ATGGGGGCTAGGCACTACGCT MGARHYA 1

TGGGATTGGGTCACGATGCCG WDWVTMP 1

GGGTGCTGCCCCTCTGCTCGT GCCPSAR 1

TTCCATGTCATCCCTAGCTGG FHVIPSW 1

TCCCGTTGCGGCGGGGCTTGG SRCGGAW 1

GGGACTCGGAGCCATGATCGG GTRSHDR 1

CCGGTTTCGTCGGCGGCCAGG PVSSAAR 2

TGCTGGTGCAGTCCTCGTGAG CWCSPRE 1

CGCGCTGCTAAGCACTGGGTT RAAKHWV 1

CGTGGTTTGCCGGTCGGCTAT RGLPVGY 1

TGTCGTAGGACGAGGGCGCTC CRRTRAL 1

CGCTTGGCTGGGATCAACGTT RLAGINV 1

CTGGATATCTCGACTTGGGCG LDISTWA 1

GATGTCTCTAAGTCGGCCGGG DVSKSAG 3

AGGTTGTACCGTGGCAGTCGG RLYRGSR 1

ACGGTTCGTGACGCGAGGGCC TVRDARA 2

CAGAGCCGGAAGAGCACCCTT QSRKSTL 1

AGGAGTAGTCGGTTGCCCAGT RSSRLPS 2

AGCTGGTTCGGTGGGCTGGCG SWFGGLA 1

CTTACGCATTCCATGGCTTCT LTHSMAS 1

TTTCTGTGGTTGCAGCGGATT FLWLQRI 1

GTCGCGTACATCAGTTTTCCG VAYISFP 1

CTTCTGAGCGGCACCGAGGGG LLSGTEG 1

GTGGACAGGGCCGTCTATAGG VDRAVYR 1

TGGCCGAGTATTGACGAGTTT WPSIDEF 1

AGGGTTGCGGAGATTGCGCGT RVAEIAR 1

GGTAGGTGCGTCACCCGGCGC GRCVTRR 1

TGGCGGCTGATGGGTGCCGCT WRLMGAA 1

AGGGTTGGCGACACCTGTCTT RVGDTCL 1

TGCGTGAGCTGTCGCTGGTAT CVSCRWY 1

AGGTGTGTCTTCATGTACACT RCVFMYT 1

TGTTTCTATGGGTGGCGGGGG CFYGWRG 1

GTCGGGCATACGTTCCGGGCG VGHTFRA 1

TTTCCTGCCAACCTGGAGTCG FPANLES 1

GGCGTTTGGATCTGCACCCGT GVWICTR 1

GCTCTGCTGGACGGCCCTCGC ALLDGPR 4

CTCGGGTTGGTTTGTCGTGGG LGLVCRG 1

GCCTCGAATTCCGGGCTGCCC ASNSGLP 2

GGGTTTATGTTCCGCGGTACG GFMFRGT 1

TTTGGGCTGTGCATCGTGGGT FGLCIVG 1

TATGCGTGCTGGCTTCGTCAG YACWLRQ 1

TTGCGTTCTGGGCGTGTGACT LRSGRVT 1

ATTGTGCTGCCTCAGTGGTTT IVLPQWF 1

AGCTGGGCTAAGCGGCTGTAT SWAKRLY 1

TCGGCGGCGTTCTCTAATTAC SAAFSNY 3

TTCTTGGTGTCCGCTGAGCCG FLVSAEP 1

ATCTTGATCACGCGTATCGTG ILITRIV 1

CTGCGCGTTTGCGGCGTTAGG LRVCGVR 1

TTGGAGCGCGGGGGCCGCTTG LERGGRL 1

GGGGGCGCGAAGGGTGACTCT GGAKGDS 1

GTGGTGCCGGCTGTCCGGGCT VVPAVRA 1

CGGTCCCTCAGCAGCCCGGGG RSLSSPG 1

CCTGATGTGAGGCTTGCTTTG PDVRLAL 1

ACTCAGGTTGCGAGGAGGGTT TQVARRV 1

GCGCGGTGGTCGGCGTTGTGT ARWSALC 1

GCCGCTGCTGGGTGCATGTCG AAAGCMS 1

GACTTGGGGACTGCGCTCAGG DLGTALR 1

TTCTATATGTTGGCTCTGACT FYMLALT 1

AAGTGGGACAACAGTCTGAGG KWDNSLR 1

CGGCGGGAGTTGTATTGCCTT RRELYCL 1

GAGCACGGGGTTTGCTCTCCG EHGVCSP 1

CTTTGGGCGGCCGGTGGTCGT LWAAGGR 2

TGGTATGTCCTCCTGGTGATT WYVLLVI 1

TTGTTTCATTATGTTAATCCG LFHYVNP 1

AGTGGGGTGGAGGATGCGAGG SGVEDAR 1

GTGTTGGCCTCGGCCGATATT VLASADI 1

GCTGGGCTGGCGGCCCGCGTG AGLAARV 1

TATTCCGTGTGGGCCAGGTCG YSVWARS 1

AGGTTTTGCATCCACGGGCGC RFCIHGR 2

GGCGTCCTCGGGGTCCTGTGT GVLGVLC 1

GATCGCATCACGGGTACGCAG DRITGTQ 1

TTCCAGTGGTTCTCCGTGGCT FQWFSVA 1

GTGGGGAGTGTTCGGGGGGCT VGSVRGA 1

AATCATGTCAAGCCCTGTGGT NHVKPCG 1

GTGAGCCGCGGGTCGCTGGGT VSRGSLG 1

GTGCGGCCGATGGGGAGTCGT VRPMGSR 1

GAGGGCTTGGGGCACAAGACT EGLGHKT 1

GGCTTCTCGAGGCTCTACGTG GFSRLYV 1

TACTGGGCGTCTAGTTTGAGG YWASSLR 1

GGTCGCTGCTTGGTTCTGCAG GRCLVLQ 1

ACGAAGGTTCGGGACGATCTT TKVRDDL 1

CATCGTAGCTGGAGCTCGCTT HRSWSSL 1

GACTGTAGGTCTCCCGGGTCG DCRSPGS 1

TTGAGGCAGCTCTCTGATGAG LRQLSDE 1

GCCGTGGTGCGGGCGTCGACT AVVRAST 1

CAGTGTCTTGGGTTGGATCCT QCLGLDP 1

GCTGTCGACAAGGCCTCTTAC AVDKASY 1

AGCGTGGGTCGTGTTCTTTCT SVGRVLS 1

TCGCGCCAGATTATGATGAGT SRQIMMS 1

GACTACGTCTGGGAGTGGGGT DYVWEWG 1

CAGGGGTGGTTGACCAGCTTT QGWLTSF 1

TACGAGGGGCCCGCTAGCTCT YEGPASS 1

GGGTTCCGGCGGGGCGGTAAT GFRRGGN 1

TGTCCCGGCGACTTGCCCAGT CPGDLPS 1

TGGCGGGGTGAGGTTATTAAG WRGEVIK 2

TATGATGCGTTGTTTTATTTG YDALFYL 1

GTTAGGCATGAGAGTGAGACG VRHESET 1

ATTTCCATGCATGGCAGGTTT ISMHGRF 1

GGGAGCGGGGGTTTTGGGACT GSGGFGT 1

AAGGGGGCTGGTGTTCGGTCG KGAGVRS 1

GGTTTGCGCCGCTGGCTGCGT GLRRWLR 1

CGGGTGGATTACTCGCTCCCG RVDYSLP 1

TGCGCGGTTTGCGGGGGGTCT CAVCGGS 1

TCGCGTCGGTCGCTGTGCCGT SRRSLCR 1

TTCTGTGAGTCGGTTAGTACT FCESVST 1

AGGATGCCGGCTGTTGCGTTC RMPAVAF 1

GGCGCTGACGGGTTGGGCTGG GADGLGW 1

CTCGAGCGGTTGGCCAGGGTG LERLARV 1

TTCCATTGCGGTAGTCGGTCC FHCGSRS 1

AGCAGTGATTGTGGGGCTGGC SSDCGAG 1

TACGAGTGCTGGAGCCCGGAG YECWSPE 1

AACGGCGACCACCGCCGGACT NGDHRRT 1

TGGGTGCTGGCCGGCGGTGAG WVLAGGE 1

TTTACCGGCCCGCGTCACTTG FTGPRHL 1

TATGTGAGTTGTTCCCCCGGC YVSCSPG 1

TGGGTCGTGTGCTTCCAGTAT WVVCFQY 1

CAGCAGAGGGGGCTGTATAGC QQRGLYS 1

TTCAGCCACTCGCATCCGAGT FSHSHPS 1

GGGTGCATGGCGAGCCGTGGT GCMASRG 1

GCGGGGCTCACTGTCGGGGCG AGLTVGA 1

CTCCGGGGGTTCTGGTGCTAT LRGFWCY 1

GGGAAGGCCGTCGGCGGGCTC GKAVGGL 2

TCGCCTAAGAGGGCCGGTTAT SPKRAGY 1

CCTGTTCGCCCGAGCAGCCTG PVRPSSL 1

TGTAGGGATATGTTGCTGGGC CRDMLLG 1

CTTGTCTGCGCTTGGGCGTGT LVCAWAC 1

GGTGTTTGCGGTCATCAGCTT GVCGHQL 1

GAGTGGCGGCAGGTTTTGACT EWRQVLT 1

GTGCAGATGATGTGCAATAGT VQMMCNS 1

CTGCTCCACGTGGCCCAGAAG LLHVAQK 1

CGTGGTTGGGACATGCTGAAG RGWDMLK 1

AGTCTGAGGAGCGGCTGGGCT SLRSGWA 1

GGCGGGGGCCCGTGTGTTCAC GGGPCVH 1

CAGCGCCAGCGCAGTACGGGG QRQRSTG 1

GCGCCGCGCCCGCGGAGGAGT APRPRRS 1

CGTGTGTGTCTTAGCAAGGAT RVCLSKD 1

TCGAGGCGGCTTTTGGCCAGT SRRLLAS 1

CGTTATGATCCGGATAGTGGG RYDPDSG 3

AATTGCGGCGCTGGCCTGAGT NCGAGLS 1

TTTGTGGTGAAGTGGTCGATT FVVKWSI 1

ATTGTCCTCTGTGCGGTTGCT IVLCAVA 1

CGCATGCCGAGGTTCCTGCGC RMPRFLR 1

CCGCACCAGCATCGTGCCATT PHQHRAI 1

GGCGACTGCAGGTCGGGGCGT GDCRSGR 1

CATCTCTTTCTTGGTGCGCTG HLFLGAL 1

ACCGACGGGAATCCCGATCGT TDGNPDR 1

CGGGCCCAGGTGCGGGTTAGG RAQVRVR 1

GAGAGTGCGGTGAGTTCCTTG ESAVSSL 1

CAGCAGCCCACTTCGCGTGTC QQPTSRV 1

TGTACGGTGGACCGGTTGGTT CTVDRLV 1

GGCGCGGTTTTCCCGTGGTTG GAVFPWL 1

TATGGGTATGGCCCTGGTGGT YGYGPGG 1

TGGGCGTCGCGCGCCGCTCTT WASRAAL 1

GGCTGGGCTGACGCCGAGGTT GWADAEV 1

GGTATTTTCAGCATGGCGCGT GIFSMAR 1

TGGTCTTGTTCTGGCTTCTAT WSCSGFY 2

TGCTTGGCTCCGGTGGGCGTT CLAPVGV 1

GAGTCCGCGACGTTCAGCGTT ESATFSV 1

ATCGTCCTGTTTCGTGTTTAC IVLFRVY 1

ACTAGGATCCCTTGCCTCGTT TRIPCLV 1

TGCAAGCCTCCCTGGGTCGAT CKPPWVD 1

CACGTCAGGCCGCGCTTGGTT HVRPRLV 1

CCTGCGCATATGATTTACCGT PAHMIYR 1

CACCGTCGGGGGGATAGGTGG HRRGDRW 1

TGGTCCGTCGGGTCTCACCGG WSVGSHR 1

GTGTGGCTGAACACGTTGTCG VWLNTLS 1

CGTACTGGTGATCTGGTGTCC RTGDLVS 1

TCGTACCTGGTCCAGAAGCGC SYLVQKR 1

GGTGGCCGGAGCGGTGTGTGC GGRSGVC 1

ATTTCGGGCTCCAGGAGCCGT ISGSRSR 1

ATGTCTTGTTGCGTGAGTCCT MSCCVSP 1

CCGTCTCGCTGTGCGTTTATC PSRCAFI 1

GTCTCTTGGCTTGTTGTGGAT VSWLVVD 1

GGCCGGAATGTCGCGCTGTAT GRNVALY 1

CTTAGGGAGCGGCGGATTACT LRERRIT 1

GTGGCTCGCGGTTTTTACGTT VARGFYV 1

GTGTTGGCGCTTTTTGACTGC VLALFDC 1

CGCCGCGGTGGGCTGAATCTT RRGGLNL 1

TCGCCTCCGGCTTGGTATCGT SPPAWYR 1

TTGTTGGTGCCTAGCCCTTAT LLVPSPY 1

TTGGGTGGCGCTCGTAGGCCT LGGARRP 1

CTGTCCCGCAATATCTTGGTG LSRNILV 1

TGCGTGCATTGTCCTGCGAAT CVHCPAN 1

CACACTGTTCGCTGCGCTCCT HTVRCAP 1

GGTGCGCGGTGGCGCGTCGTT GARWRVV 1

CGGTATTATGCTTGGTTCCGG RYYAWFR 1

CGCTTTGGTTTGTACACTGAG RFGLYTE 1

TTTTGGGTTAGGACCGAGCGC FWVRTER 1

TCGACTATGGTCATTAACCGG STMVINR 1

GATGATGGGCCGGACAATCTT DDGPDNL 1

GTCTTTTGTAGGTGTGTCGCT VFCRCVA 1

CTCTTGCGGCGGATTCCCCAT LLRRIPH 1

GGTGAGTGGTTTGAGTCGGCT GEWFESA 1

CGCTGTGGCAAGTGTGGGAGG RCGKCGR 2

CTTCGTCGCAGTAACCTCGTT LRRSNLV 1

GCTGTTTTTATGACTCGTCAG AVFMTRQ 1

GGGCGGAGTACTATCTTTCTC GRSTIFL 3

CTGTCGTCCATGAGCGCGCGC LSSMSAR 1

TGGGCTGCTGCGATCGCGAGT WAAAIAS 2

AGGTTTCCCCAGAGTTGCGAG RFPQSCE 1

GTTGGTTCGAGGGGCTTCCTT VGSRGFL 1

CGCCTTCCCCGTGAGTTCGAT RLPREFD 1

GACACTAACTTGTGTACTCAT DTNLCTH 1

CTTCTGGGGATCTGGGTTCCT LLGIWVP 1

AAGTTCCTCGTGAGGCGGAAG KFLVRRK 1

GAGGTGAAGGCCGACTGGGGT EVKADWG 1

CATGCGGGGGAGGAGGCGGCT HAGEEAA 1

TTGACTGCGAATGCCGACTCT LTANADS 1

GAGTCCACGTGGCGGGTGCGT ESTWRVR 1

CTCTGGGTTATTTTCTCGTGT LWVIFSC 1

GTTAGCGGCAGGCTCAGGCCG VSGRLRP 1

CTGGTCTGGCATTACCACTAT LVWHYHY 1

TGCACCGGGGACGTTAATTGG CTGDVNW 1

AGTGTCGTGCGCGTGCACAAT SVVRVHN 1

GGTTTTGTGATCAGGTCGCGG GFVIRSR 1

AGGTCGGTGGCGTATTGTTTT RSVAYCF 1

TCCAAGCTCTATCACGAGTGT SKLYHEC 1

GCGGCTATGGGCATCACCTTT AAMGITF 1

TCGCACGGCCGCACTTTTAAG SHGRTFK 1

AGGTGGATCAATAGGACTTCT RWINRTS 1

TGCCGGAGGGTGCTTTGGGGC CRRVLWG 1

TGCGGGCAGAGGGCCGTTCGG CGQRAVR 1

ACGGAGCGGGGGCTTCGGTTG TERGLRL 1

CTTCTCTGTGTGGCGTTGGTG LLCVALV 1

GCTATGCTCACTTCTGAGAGG AMLTSER 1

GGGGTGGCCCGGTTCACCCAG GVARFTQ 1

ATTTGGGTCGGGTGGTGCAGG IWVGWCR 1

GGGCGGTATTTGCGGTGGTGG GRYLRWW 1

CTCGTCCCTTGTTGGACCGAT LVPCWTD 1

GCGCGCTTCAGTGTGGTCGGG ARFSVVG 1

CGTCTCAGCTCTTGGATGTGT RLSSWMC 1

CGTTCCGCGAGTAGCGTGAGG RSASSVR 1

ACGGTGCGCGTTGGGTTGCTT TVRVGLL 1

GGCATGCGCGAGGACATCGCT GMREDIA 1

GACGGTGGCGAGCTTGTTCAG DGGELVQ 2

GCTGCCGAGTGCGGGGACCTT AAECGDL 1

TTGGTGCAGTCCAGCGTTGTC LVQSSVV 1

GTCAGGAGCTATATGGTTCCG VRSYMVP 1

GACGCCACTCTCCGGGTCAAT DATLRVN 1

GTCGTGCTCACTCAGGCCGTG VVLTQAV 1

CTGCGCGCTAATCCGCACCGT LRANPHR 1

GGTGGGCGCTCCTTCGTCATT GGRSFVI 1

CTGCAGGGGTGGAGGGCGGTT LQGWRAV 1

GGTCCGAATTCCGTTGATGGG GPNSVDG 1

TGCTTCAACGTGACGTGCACG CFNVTCT 1

TCTCGGGTGGTCGTGGGCTCG SRVVVGS 1

GTTTGGGTCCATAGGAAGAGG VWVHRKR 1

GTTGGTTTGGCGAGGGATGCT VGLARDA 1

GAGCGGCTCAGGCTTCGGCTG ERLRLRL 1

CTCGCGCTGCTTATGGTGAGC LALLMVS 1

TTGCCCGAGCTCTGTTGTTGG LPELCCW 4

GGTCGGAACCTCATTTCCGAT GRNLISD 1

CCTTACTTGACGATTCTCTTG PYLTILL 1

AACGGGGTGGAGATGCAGCTT NGVEMQL 1

GTCGTGCGGCTGGCCTGGCCT VVRLAWP 2

CATCAGGGCTCGTTGTGGGTG HQGSLWV 1

GTGGAGCAGTATTGGGACCGC VEQYWDR 1

TTTCTGCACACGGGTGGCGGT FLHTGGG 1

AAGAGGATTGGTAGTTGGGCG KRIGSWA 1

AACCGCCTGGTGGCCTTGGAC NRLVALD 1

TCGTGGCGCTGCTGTCCGCGG SWRCCPR 1

ATGAGGCTTGAGGTTACCGGT MRLEVTG 2

TGGGAGCTCGGGCTGACGCTG WELGLTL 1

CACTGCGGGTGGGGTGAGATG HCGWGEM 2

CTGTTGGACGGCCACTGCGTT LLDGHCV 1

GGTAATGACATGGGGTGCAAT GNDMGCN 1

TTCCTGATCGTGGTGCAGAGT FLIVVQS 1

CGGCACGATGTGATGGTCGTT RHDVMVV 1

TTCTCTTATGTCCGTGTTGTT FSYVRVV 1

TATGGGCGCTGCGATTCTGCT YGRCDSA 2

TGGAGGATGTCGCGGGTCCCC WRMSRVP 1

TCTGACCGCTGGTGGGATTCT SDRWWDS 1

CGGATCTCTCTCGTTTGTGGG RISLVCG 1

GGTCTCGGTAGTGGCCTTTCC GLGSGLS 1

TCGTACGCGTACGGTCTGCCG SYAYGLP 1

TACGAGTGGTGGTGTCCTCGG YEWWCPR 1

GTGGACGCGGCGAGCTCTCGT VDAASSR 1

TTCCGGTCCGGTTTTGGCATT FRSGFGI 1

CGGAGCGGTACTGACGCGCTT RSGTDAL 1

GCGCCGCGTATGGAGAGCCGG APRMESR 1

GTGCATCCTACGGTGGTCAAC VHPTVVN 1

GGTGCCGGGTCGCGGTCTCCG GAGSRSP 1

GTCGCTCTTTATTTGTCGCAT VALYLSH 1

CTGGGGCTGTGTAGCTTCAGT LGLCSFS 1

AACTATCGGACGCTGAGGCGT NYRTLRR 1

GGCCCGCGTTCGTCCGATCTG GPRSSDL 1

CGTTCTCACCGTGTGGGGTCG RSHRVGS 1

CTCGGTGCCACGTCTGGGGTC LGATSGV 1

TCTGACTCCACTGAGGCCGAC SDSTEAD 1

GAGATCGCCGCCCGGGGTTGG EIAARGW 1

ATTTTCAGGGTCGTCTGGCGT IFRVVWR 2

GCTTACCACGTTTGTCACTCG AYHVCHS 1

TTCGTTGACATTCTCGCGGGT FVDILAG 1

CGTGATCGCAATCCGGTGCCG RDRNPVP 1

ACCATCCAGTGCAGCGCGCTG TIQCSAL 1

CGGTACGTCGACGCGTCGTAT RYVDASY 1

TACTATGCCGTTCCCCTTGTG YYAVPLV 1

TGGAAGATCCGGCACCGCGAT WKIRHRD 2

TACGTGTATCTCTTTACTTTG YVYLFTL 1

GAGCTCGCCCAGATGATTAGG ELAQMIR 1

CAGAGGCCGAGTGCCGTCCTC QRPSAVL 1

TTGTTCCGCGCTCTGGGCAAG LFRALGK 1

CTCAATCGCGCTGGTCGGAGG LNRAGRR 1

GGCCACCCGAGCGCCGTGGGT GHPSAVG 1

TCGCGGCCGGGTTCCGGGTTT SRPGSGF 1

CTTAACGGCCGCGTCCTTTAT LNGRVLY 1

ATTTATAGGGGTCAGAGGGGG IYRGQRG 1

TGGCTTACCCTCGACGGTCTG WLTLDGL 1

AGGTCGTGGATGCGCTGTGCG RSWMRCA 1

GCGTACACCGGTCCTCAGTAT AYTGPQY 1

GAGTTTATCAGTGCTGGGCGC EFISAGR 1

CTTCTGGCCTACTCGCCGGGG LLAYSPG 1

GTTCACTGCGTTGTGTTTGGT VHCVVFG 1

GAGTGCGGGTGCAGCAATATC ECGCSNI 1

TCGGTGTGTTGGAACTCGCGG SVCWNSR 1

AGCATCGGCCGCGTCAGCCGT SIGRVSR 1

TGCGGCCACAGGAAGGTGTGT CGHRKVC 1

GTCATGGCCGCGTGGTGGACG VMAAWWT 1

TTGGGTCTCTTGAACGCCTTG LGLLNAL 1

TCGATGGTGATCTGCGTGGAC SMVICVD 1

TGCGAGTGCTGCGGGAAGAGT CECCGKS 1

TGGCCCGACCCGTTTGTGAAT WPDPFVN 1

CTTCGTCCCAGTTACCTTGGT LRPSYLG 1

TTGGTGAGGTTTTGCCTCTGT LVRFCLC 1

TTGAAGTTGGCGGAGGCTGGT LKLAEAG 1

GTGCCTGTGCGGGGTGAGCCT VPVRGEP 1

GGCCTTGCCAAGGTGCTGGAT GLAKVLD 1

GTCGTGGGTCAGGCGCGGGGT VVGQARG 1

TGGAGGACTCGGGTCGGCTAT WRTRVGY 3

CTTAGGAGTTGTGCGGGTGTG LRSCAGV 1

AGGGCGGCTATGAAGCGGGCG RAAMKRA 1

GCCTACAGTGTGTACTCGTCG AYSVYSS 1

TTTTTCCGCAAGCAGTGGCCG FFRKQWP 1

CTGGCGTTGTCTAATTCTGCG LALSNSA 1

GGCCCCTACAGCAGCCACCGT GPYSSHR 1

TTGTGGGTGGTGGCCGTTGCT LWVVAVA 1

TCCGGTGAGTTGAGTGCGAGG SGELSAR 1

TCTCGGATCATTAATGATGGG SRIINDG 3

GCCAGCGCTGTCGCCGGGCCT ASAVAGP 1

TCGCTTGGGTACGTTAGCGCT SLGYVSA 1

GGCAATGGGCCGTGCTCCTCT GNGPCSS 1

TTTATGCGTGCGAAGCAGAGG FMRAKQR 1

AATATGGTGTGGGCGAACGTC NMVWANV 1

GATGCTGGGGCCCAGGCGTGT DAGAQAC 1

TGTGGTGTCCGTCCGGTTGCT CGVRPVA 1

CGTGGGTTGGCCAACATGCGT RGLANMR 1

GCTCGTTTCCCTGTGCTGCGT ARFPVLR 3

TGGAGGCGCAATCGCCCCGTG WRRNRPV 1

ACGGAGTCCGAGGTTGATTCG TESEVDS 1

TTTTCCTCTGGGCCGTGTTAT FSSGPCY 1

TCCGAGGGGTTGCGCTGGACT SEGLRWT 1

TGCCGGTGCACGGCCGAGCGT CRCTAER 1

TGTGAGTGGACCTGCCGTAGT CEWTCRS 1

GTCGAGGCGTGCGTCCTCGCG VEACVLA 3

ACTCGCTGCTGTGGTACCGGG TRCCGTG 1

GCTGTTTTCCCTGTCCCTGCT AVFPVPA 1

TGGCGGGCCCCGCGTAATTTT WRAPRNF 1

GAGCGTCCTAGGTCCCCGGAT ERPRSPD 1

ACGTGCTTGGTCATCTGCCAG TCLVICQ 1

GGGGAGTGTGGCGGGGTCTGG GECGGVW 1

CGGTGTTTTATGTACGGGTTT RCFMYGF 1

GGGCAGGACAGGGTCGACGCG GQDRVDA 1

AAGACTCTGACCAATTGTTCG KTLTNCS 1

GAGGGGCGGGTCGAGGTTCAT EGRVEVH 1

GTCACTGGGTGTCGCGCCTGT VTGCRAC 1

CGGGGGTCGATCGTGGCGGGG RGSIVAG 1

AGCGACGGTTGTGGGTCCGGT SDGCGSG 1

GTGATGGCTAGTGGTCCTGTT VMASGPV 1

TCTCTGGTCGTCGCCCAGGCG SLVVAQA 1

CGCCAGCTCGCGTCGAGTGGC RQLASSG 1

TCGTGGCCGACTCGTTGGTCG SWPTRWS 1

GATCACCTGACGCGTCGTCGC DHLTRRR 2

GCCGATTATGGCGCGAGGACC ADYGART 1

GACATCCCGTATTTTTGCTGT DIPYFCC 1

CAGCGTTCGACGGAGGGGCTG QRSTEGL 1

GCGTCCGGCTGGGCTGTTTAT ASGWAVY 1

GTCGCCGTCATGCATCTCCCT VAVMHLP 1

ACCTACAGCGCGAAGATTAGT TYSAKIS 1

TGGACCTGCGCGAGCACGAGG WTCASTR 1

GTCTCTGTGAGCTATGGTGCT VSVSYGA 1

CGGGTGCATGCCCGGTCGGTT RVHARSV 1

GTGCACGTCGCTTTGGCCCGG VHVALAR 1

GCTGGCAAGTTGCTTGTGCTT AGKLLVL 1

TTTGAGAGTCCGCCGCTTGCG FESPPLA 1

GGCGGTGTTGACGCTGCTCCG GGVDAAP 1

GCTCACCGTTGCTCGTTTGTG AHRCSFV 1

TGTTTGGCTTCGCGGGCCCGC CLASRAR 1

AATCTGAGCCATAAGGTTGCG NLSHKVA 1

CACTTGCTGGTTGCCGGGCTG HLLVAGL 1

AGCCGGTGCTGCTGGAAGGCG SRCCWKA 2

TGTGGGCTGGACTTTAGCAGG CGLDFSR 1

TTGGTGCAGAGGCAGGTTTGT LVQRQVC 2

GCTGGCCATCCGAGGTTGGGG AGHPRLG 1

CACTCTTCTTCCCGCGGGATT HSSSRGI 1

CTTTCGCACCCTGCCTGTAGT LSHPACS 1

CGGGTCGGGAGGGTCATTTTG RVGRVIL 1

GTGCTGTCTGGGGTTATTATT VLSGVII 1

GTTCGTCCTAGGGTTGGGCGG VRPRVGR 1

TTTGCTGCGTGCGCGCGGGGC FAACARG 1

GTCCTGCATTGGGGGTGCTGC VLHWGCC 1

AATTCTCCGACGTTCCCGAGT NSPTFPS 1

ATGGTTGTCTATCGCTGTCAC MVVYRCH 1

TGCTGTGGCCAGGTCAGTTGT CCGQVSC 1

GTTGTGCTGCTCAGCGTGTCG VVLLSVS 1

TGTTCGCTCGACGGCTGGCAG CSLDGWQ 2

GATCTGGGCGAGCGCAGGCCT DLGERRP 1

TGCCGGTCCACGCTTTCGATT CRSTLSI 1

CGGCGCCTTGGTCGGGTGAAT RRLGRVN 1

CCTGGGCGGTATAGCGTCCTT PGRYSVL 1

CGCGAGGGTACGCTGGCTGGC REGTLAG 1

TCGCCCGGGTGGGGTGGCGGT SPGWGGG 1

GGTCACGTCGAGGTTGGTTTT GHVEVGF 1

GGCGGGCGTCATGGCGGCGAT GGRHGGD 1

TACAAGCGTTGGCCGTTTGTT YKRWPFV 1

GACGGGGGCTTTAAGGTTACG DGGFKVT 1

GTGAATCCTTTGATGTTGTCC VNPLMLS 1

TCCGGTTTCTGGCACCCTTGT SGFWHPC 2

GGGGAGTTTTGGTCGCTTGGT GEFWSLG 1

GTCCGGTCTGGGGCTGGGAAT VRSGAGN 1

GCCCCCCGCTCTTGGGGGTTT APRSWGF 1

AGCTGGAACACTAGCGAGGTC SWNTSEV 1

GCTGAGTTGGTGATGGTCTGT AELVMVC 1

GGGTATTCCACTCTCGCCCCG GYSTLAP 1

CATCGCATGAGTCGGAGGTTG HRMSRRL 1

GTTGTTCGCTCGGCGGGCTCT VVRSAGS 1

GGCACTCGGCGGCCTAGCAGC GTRRPSS 1

CGTGCTTGCTTTGGCACGGCT RACFGTA 1

CGGGATAATCGCAGCGGGCGC RDNRSGR 1

GGCGATACGGCGGCCCCTCGC GDTAAPR 1

AGCGGGGCGGCTATTGGTCCT SGAAIGP 1

AGGAGGGGGACCTGGAGTAGC RRGTWSS 1

CTGTGGGACTTTTGCTGTCAT LWDFCCH 1

CGGCTCAGGAGTAGGTTTCCC RLRSRFP 1

CTCTGTGGCGCGTGCGCGAGG LCGACAR 1

GACGCTTGCTGCGGCTTGGCT DACCGLA 2

GGTGGCCTTGGGTTCCAGGTC GGLGFQV 2

GCGCCCGTCTGCGGGGCCTGC APVCGAC 1

GTTTCTTGGCTCATTCCGACT VSWLIPT 1

GGTGCTCCTCCGGGTGGTGCG GAPPGGA 1

GTCTCGGAGGTGGTCGCTTGG VSEVVAW 1

CCGCAGCGCGTGACGGTGCCG PQRVTVP 2

GGTACGGCGATGAATACGACT GTAMNTT 1

ATGTTGGGTGGGTCTCGTTCT MLGGSRS 1

TCCTGGGTGAGGCGTAACGGT SWVRRNG 1

GGCGTTTTTTCGGCGTTTCGG GVFSAFR 2

GGTGTTCAGGGGCGGGCCGCG GVQGRAA 1

TCGCGCTACACGTGGCCCGCG SRYTWPA 1

GTTCTCCGCGGGAACGGGATG VLRGNGM 2

CATACGGGCGGTGGCAGTTGT HTGGGSC 1

CTCAGTTGGGGTTGTAACGTT LSWGCNV 1

CATGATTCGGACTGGGTTGGG HDSDWVG 1

TGTCGTGGTACGCTGTTGGCT CRGTLLA 1

TACAAGGACTTCTACACGTTG YKDFYTL 1

GGCTATTTTGCGGGGTGGGTC GYFAGWV 1

AGCGGTTCGTGCTGCGGCGAT SGSCCGD 1

TCGCCCGAGCGTTTGAGCTTG SPERLSL 1

GTCTGTGGCTGTGTCATCTGT VCGCVIC 1

GTCCGGAAGTCGGTTTGGCCG VRKSVWP 1

CCGTACACGAGCGATAGGAAC PYTSDRN 1

AAGGATGGCGCGTGGGCTGCG KDGAWAA 1

ATGCCGGCGAAGTGCGCCGGT MPAKCAG 3

ATGCGGCGCCTCGACAGGTAC MRRLDRY 1

CCGTCGATGGGCGCTAATGTT PSMGANV 1

GGTCCGGGTGCGGTCTTGGCG GPGAVLA 1

GTCGCCTGGCTGTACGGTTCT VAWLYGS 1

TCTAGGCTGAGTCGGTGGATG SRLSRWM 1

TTGCTGCGGAGTGCCTCTGGT LLRSASG 1

GACGGCTTGATGCTGCGCGGT DGLMLRG 1

TGCAGCGGTTACGGCGAGAAG CSGYGEK 1

CTGTGCAGCAGGATTGTTTGC LCSRIVC 1

GGCCGTGGCACGGAGCGGTTT GRGTERF 1

GAGCGCTGCGGGTTGGACGTT ERCGLDV 1

GCGGGGGGCGTGTTCTTGCGG AGGVFLR 1

TTCTCTACCAGCGTGAGCGGG FSTSVSG 1

CCCGGTTTCTCGGAGTTGCGT PGFSELR 1

GAGTCCCTCGAGGTGGTCTCC ESLEVVS 1

ATTTTCTGTGCGAACCGTCGG IFCANRR 1

ATTGTGCTGGTCAGTCTGCGG IVLVSLR 2

CCTTGTCTTAGCGATGTGGTT PCLSDVV 1

GCCCGGTACAATGGTAGCCTG ARYNGSL 2

GGCTCGCACGGGCTCTTTATT GSHGLFI 1

AATAGTGTTGACGTGGGCTCT NSVDVGS 1

CACTGTTTTAGTTTCTTTTCG HCFSFFS 1

ATTAGGATCACGGGGCGTGCT IRITGRA 1

GTCTTTCTGCGGCTGCTTAGG VFLRLLR 1

TTTGTGCTTTATAATGCCTGG FVLYNAW 1

AGCTTGGGCACGTGCGGGAGT SLGTCGS 1

CTGTCGGAGGCCATGTATGTT LSEAMYV 1

GTGCTTTCTGAGCATGGCCTG VLSEHGL 1

GAGCATTGCGGCTCGTTTCGT EHCGSFR 1

CAGGAGGCGGTGGGCAGGGAC QEAVGRD 1

CACCCTCATGGCGAGGTTGCG HPHGEVA 1

TTTGGCTCCGGGGCCGTCGCT FGSGAVA 1

GGGCTTGAGTGCTGCTGGGTG GLECCWV 1

GAGTTTATCTGGCTTCTGCTG EFIWLLL 1

GTTTTCGACGGCCACGGGACG VFDGHGT 2

ATCGTGGGTGGCAGCAGCTTG IVGGSSL 1

GTTAAGCGCCGCTTCACGGGG VKRRFTG 1

GAGGAGGGTTTGTGCCTCTGT EEGLCLC 1

GGCGGCCGGATCGCGTATCCT GGRIAYP 1

GTCTTGGGCCTTATGGTGGCG VLGLMVA 1

TGTGGCAGCTGGAGCTGTGGT CGSWSCG 1

CCCGGTCGCGGGCTGGGTCTT PGRGLGL 1

CCGCGCGAGGTTCTTGCGCCG PREVLAP 1

ATGGGGTTCGGTAGCATGTCC MGFGSMS 1

GGTATGCGCGGGTGCGATCGC GMRGCDR 1

GTTGGGCACGCGGAGCAGGAG VGHAEQE 1

ATGGCTGGGATGGTCACGACT MAGMVTT 1

CTTACCTGTAGGCTGTGGCCG LTCRLWP 1

TGGGACGGTGATATCAGCTGG WDGDISW 1

TACCAGCGCGACTCGTGCGCT YQRDSCA 1

AGTGAGTGCGGGGGCGGTTCT SECGGGS 1

GCGAGGTATCGGAGCTCTAGT ARYRSSS 1

CTGAGCAGGCTCCAGCACTTT LSRLQHF 1

GACGTGCGCCTGCAGCCGCGG DVRLQPR 1

GCTACCGTTGTCGCGAGGAGT ATVVARS 1

CTCACTGTCGCTTTGCCGGCT LTVALPA 1

TCCCTGGTGCGGTCCGCCGGT SLVRSAG 2

GGGAGCGTCGGGATGTTTTGT GSVGMFC 1

GTCGGGGGTGGTCTGTCTTGT VGGGLSC 2

GGCTTGATCATCTCTTTGTCT GLIISLS 1

GGGGTCCCTGGCTGGTGCCGG GVPGWCR 1

CAGGGGCTCAGGGTCCTGCGT QGLRVLR 1

GCCTTTGGCACCGTTGCGCAT AFGTVAH 1

CTGCGCAGGGCGGAGCGGTGT LRRAERC 1

CTCGCGCTTCCGTTGTGCCGG LALPLCR 1

AGGCTCGGGTGGCCCTCCGAT RLGWPSD 1

GAGCCTAACTCCAGTCCGGGT EPNSSPG 1

GTTCGTATTATGGCGCTGACC VRIMALT 1

CTCGGGCGTGATGGGCTTTAT LGRDGLY 1

GACGGGTGTTGCGTGTCTGTT DGCCVSV 1

GGGGTTCCGGGGAAGACTCAT GVPGKTH 1

GGTGATGGCGCCGAGGTGACT GDGAEVT 1

GGGCGGGGCCGCTTGGAGGAC GRGRLED 1

TGCTACGGCCCGTCGCACCAG CYGPSHQ 1

GTGTACGGTACGTGCTGTGAT VYGTCCD 1

CTTTCGTGCTGGGAGCAGTCG LSCWEQS 1

GGGGCGTCTCACGTCGAGATT GASHVEI 1

CACTTGGACGATCTGGTGCGG HLDDLVR 1

TTGGAGGCGGAGGTGTTCGGT LEAEVFG 1

TCTTCGGCCCGTGGTAGTGCT SSARGSA 1

GTTTTCCCCCGTTGGGTGCGT VFPRWVR 1

GTCCTCCAGACCAGCAGGCGC VLQTSRR 1

GGTACCCCTGGGACCCCTCCT GTPGTPP 1

GCCGCGGCCTTCAACCTCGTG AAAFNLV 1

GTCCCTTTGGCGGTTCGCACG VPLAVRT 1

GTTTGGATCCGGCCTACCGAT VWIRPTD 1

ATCGATCCGTTCGGTCACCCG IDPFGHP 1

ATGCGCAGGCTTTTGGACTTG MRRLLDL 1

ATGGGTGGGCATGGGTGGGGG MGGHGWG 2

GTGTACTGGATTGGTGTGTGG VYWIGVW 1

AGGGCTGGCTGCCGCGCGCAG RAGCRAQ 1

GCTCTTATTAAGGCCCCGAGT ALIKAPS 1

GTGCGGTTTGAGTTCCTTGGG VRFEFLG 1

TCTCTGACGCTCTCGAGTTGT SLTLSSC 1

TCGAGTAGGTCGCCCGGGCCC SSRSPGP 1

GTGTGTCGTTGCATCGGTGTC VCRCIGV 1

TATCCGCCCGTTGTTCTTTCT YPPVVLS 1

GCGGTGAACGAGTCCGGCGCT AVNESGA 1

TCTTTGGACCTCCAGCGTTTT SLDLQRF 1

TCGTTTATCGGGGCGTACTCT SFIGAYS 2

TCGGAGGTGCTTTACAGCAGT SEVLYSS 1

GCGCGCTGTAGCATTCTGGTG ARCSILV 2

GCGATTGGTGGCGACGGGCTT AIGGDGL 1

AGGAGTGTTCAGAGCCTCGTG RSVQSLV 1

AGCGCCAGCACTCGCCTTGTT SASTRLV 1

AGGTTGCCTACGCGGGTCAAG RLPTRVK 1

GTGCATTATACTCGGCTCCTG VHYTRLL 1

ATCGGTAGTCCCAATGCTGCG IGSPNAA 1

CTCGTGCAGCCCTCGGGGTGC LVQPSGC 2

GCGCCTTTCATGAAGTCGTTT APFMKSF 1

GCGTGTGGCTACATTGGGGAG ACGYIGE 1

CGCCTCGGTCGTGAGTCCCTC RLGRESL 1

TATTGGACCGCCGTCTGCTAT YWTAVCY 2

CGGAATGGGATTACGTGGGGT RNGITWG 2

GGCTGTAGCGACAGCTGCACT GCSDSCT 2

ACGGTTGGCTCTGGGTTGGCG TVGSGLA 1

ATGAGCGGCGCTCGGCGTGTG MSGARRV 1

TTGGTCTCTGCGGCTTTGTCG LVSAALS 1

CCTGGTCTTTTTATGGGGCAG PGLFMGQ 1

AACAGTCACTTTTCCGGTACG NSHFSGT 1

GGGGAGTTCGGGCCCCATCGG GEFGPHR 1

AAGGGGGACTGCGGGGTTAAT KGDCGVN 1

CTTGGTGGCGTCTGGAATGTT LGGVWNV 1

GTGCAGGGTCTCTGTTTTAGG VQGLCFR 1

CGCGCGACGGACGAGTATGAT RATDEYD 1

CTCTGTGTCTACAACGGTTGT LCVYNGC 1

TGTTGTGCGTCTGACAATGTG CCASDNV 1

TCTAGCCGTCGTCCCTCCCGG SSRRPSR 1

ATTGTTCGCTGTGTGTTGGGT IVRCVLG 3

GTTACTGGTCGCACCGCGATC VTGRTAI 1

TTCAGCGCTGTGGGCGTTGTG FSAVGVV 1

CGTGTTTACGCGGTTCTTCAG RVYAVLQ 1

CTGTTGTTCGGCTCCCTGCGG LLFGSLR 1

AAGGCCCATGGGCCGCAGCAG KAHGPQQ 2

AGTTTGTGTTTCTGCTGTCTT SLCFCCL 1

CTGGGTCGGTTCGTCCCTATT LGRFVPI 1

AGTGGTTATAGCATTTTCCCT SGYSIFP 1

AGTAGGTTGAAGTTGGGCCTT SRLKLGL 1

TTCTACTGTGCCTCCGGTCCT FYCASGP 1

GTCTGTTATCTCCGCGGCCGG VCYLRGR 1

GCGGGGCGGATTTTTAGCGGT AGRIFSG 1

AAGCATTGTCTTTTGGTGCCG KHCLLVP 1

GGCGGGCGGTGCCGGGTCTAT GGRCRVY 1

ATGGATCTTGCGCGTTTGGCT MDLARLA 1

AGGAGCTTTGCTTCCACTGGG RSFASTG 1

GTGAGGCGCCTTGTGGCTCTT VRRLVAL 1

GGGAGGCTCCCCTCCAGGTTT GRLPSRF 1

GATTGCCCCGGGCAGGTGATG DCPGQVM 1

TGCTGCGGCGTTCGTCGTTGC CCGVRRC 1

TTTAGTGCTAGGCTTTGTAGT FSARLCS 1

AAGTTCTTGGTTGCCGGGCGG KFLVAGR 1

CCTGAGAGGCGGTACGGGTCT PERRYGS 1

GCCTCCTGGAATGCGAGTCGC ASWNASR 1

GAGCTCGTTGTGATGGTGCAG ELVVMVQ 1

ATGAGGTGGCCGGGTCGGATT MRWPGRI 1

TCTAGGCTCGGGCTGGCTAGT SRLGLAS 1

ATCGGGGTCGGTTGCCTGCCG IGVGCLP 2

CAGGGTTATGGCTTCGGTATT QGYGFGI 1

TTGATCCGCTCTCGTTCTGGT LIRSRSG 1

ATGTGGCTGGCTTGGCGGAAT MWLAWRN 1

CCGTGGCGGGCGTTGGCGCGT PWRALAR 1

GGTTTCCGGGAGTGCGCGCAG GFRECAQ 1

CACGGGTCTGACTGGCTTCGG HGSDWLR 2

ATCTCTGTCCCGAACGGCGGT ISVPNGG 1

GATCGTGTGGGCTTCCTGGTG DRVGFLV 1

GTGCGCTGGCTGGCCCTCCCG VRWLALP 1

GTTGAGCGCCACGGCGCTCGT VERHGAR 1

ATCATTGGGTTCTCCAGCACT IIGFSST 1

CATCGTCACGGGATCCGGCTC HRHGIRL 1

TACGGCTCGGTTGAGTCCACT YGSVEST 4

GGGGGTTTGATTCTCGCGGTG GGLILAV 1

ATGGGTGTGGAGACTTGTGGT MGVETCG 1

AACTGTCGCGCGAGGCGGCCC NCRARRP 1

CGGTTCTTTGGGGAGTTGAAT RFFGELN 1

TGTGCGGAGCTCGTTTGTGTT CAELVCV 1

GATGCGGCGAGGGGGGCTCGG DAARGAR 1

CTTCAGAACAGCACCTCTACT LQNSTST 1

GACCCGCGGGCGCGCAGCGCT DPRARSA 1

GATGGCCGCGAGAGGGAGGTT DGREREV 1

CTGTACGGGTCTTGGCTGGGG LYGSWLG 1

AGCGTCGGTATCAATTACGGG SVGINYG 1

TGGCGCCCCACCGTCGTGTTT WRPTVVF 1

CGGAATTCCCCGATCACGGAG RNSPITE 1

GGTATCACCTTGCCCGATGGT GITLPDG 1

GACGCTGTGGGTTTGAATTAT DAVGLNY 1

TCTCCGGCTAGCGCGATCCGC SPASAIR 1

AACGATGGGTGTCTCTCCTCG NDGCLSS 1

TTGCTTCGGAGGGTGTTCCAG LLRRVFQ 1

GGGCAGGAGTGCATGTGCGGC GQECMCG 1

AGGGAGTGTGTTCCTTCTTGG RECVPSW 2

TTGCGGGCCCAGGTGGGGCTC LRAQVGL 1

ACTCTGGACATCTGCTTGCGT TLDICLR 1

AGCCCCATCCACGGCTTGCCT SPIHGLP 1

GCGAGGGGGCAGACTTATTGG ARGQTYW 1

CCGTGTCTGAGGGGGTCCGGC PCLRGSG 1

CGGTTGTATTGGCGCACGTGG RLYWRTW 1

TTGAAGAACTCGCGGGTTTTG LKNSRVL 1

TGGATGGGCCGGACGGTGTCG WMGRTVS 1

TGTTTCCTTAGGAATGCGAGT CFLRNAS 1

CTCCTGCCCAAGGGTATGCGG LLPKGMR 1

AGCCGGTTCGTGGCTGTGCGG SRFVAVR 1

CCGCGCAGTAAGGGGTCCAGG PRSKGSR 1

TCTTGGTTGGGCTGCCGTAAT SWLGCRN 2

GGCAACCTTCTCTGGGTCATT GNLLWVI 1

CATCGGCGGGTTAGGTTCTGG HRRVRFW 1

TATCGGCGCGTTGAGGTGGGG YRRVEVG 1

AGCAGCCAGAGCCGGCATAAT SSQSRHN 1

ATGCGCGGTGGCGGGAGCCTT MRGGGSL 1

GGGTCCGGCATGAGCCGGATT GSGMSRI 1

GGTCTCGAGTTTAGCCGCGGG GLEFSRG 1

TCGCGGGCTGGCTGTAAGCGT SRAGCKR 1

AGCAGGCATCCCAGTGGCGTG SRHPSGV 1

GGGGCGCGGCGTTTCACGGTC GARRFTV 1

TACCTCTTGGGCCTGTCGGCG YLLGLSA 1

GGGGGGGTGGCGATGCGGCCT GGVAMRP 1

CCGCGGACCTTGTACGGTCCC PRTLYGP 1

GAGGCGTGCTTGCTGGATGCG EACLLDA 1

GTCGATGACGGGGTTTTGTTG VDDGVLL 1

AGCGACAACTGGCTCCGCTCT SDNWLRS 1

CGCGGGTACTCCGGGGACTGG RGYSGDW 1

GTTCGTCGGTTCACCTTCACG VRRFTFT 2

GATTTGTACCGGTGTTGCGGT DLYRCCG 1

TTGATGAGTACCTCGTCCAAG LMSTSSK 1

TGTTATAATTACAGGTATTTG CYNYRYL 1

AGCGTTACCAACGAGATGATC SVTNEMI 1

TGGGTGGCCAGCGGTAAGCCG WVASGKP 1

TACGTGAGTGGTCACGGTCTG YVSGHGL 1

TGCGTCCGCAGCGGCGCGCGG CVRSGAR 1

GACCCGATCTGCTGGACGTGG DPICWTW 1

TGGTGCAGGGGGATTTTGAAG WCRGILK 1

CTGTGGTGCAGGCTGGCTAAT LWCRLAN 1

GGGAGGCACATTCCGTCGATC GRHIPSI 1

GATAGGAAGTTCCATGTTTCC DRKFHVS 8

GTGTCCTACATTTTCGGCCCT VSYIFGP 1

TGCCAGGCGTTCAGCACTCGG CQAFSTR 1

TGGTCGGACTCTGGGTCGTAC WSDSGSY 1

CTCCAGAGGATTCTGATGTTT LQRILMF 1

TACAGTGGCAGCCGCGGGACC YSGSRGT 1

AGCTCGCATAGTGGCGTTCTG SSHSGVL 1

CCCCCGTTCATTGCTCAGTCC PPFIAQS 1

AATGTGGACTGCAACGTGTGT NVDCNVC 1

TTGTCCACTTGCCTGTATCTT LSTCLYL 1

TCGTGCGCCGTCACGGACTGG SCAVTDW 1

GTGTGTGCTTTTTGTTCGGCG VCAFCSA 1

GGTTGGCTCAGGAGCGGCTGT GWLRSGC 1

GCCGAGCGTCGGTGGGGGGTT AERRWGV 1

TCCGGCACGAGCATTTCTGGG SGTSISG 1

CTCAACAGCGCGGGCGCGTTC LNSAGAF 1

CCGGGTTTCCTCTTTACTGGT PGFLFTG 1

ACGGACCATCCCTGCCAGTCG TDHPCQS 1

GTTGGGTCGGCGTGGGTTGTG VGSAWVV 1

CACGAGTGGTCGCGGAACTGC HEWSRNC 1

GTTTCCTTTTGTTTCCTGGCG VSFCFLA 1

TCTAGTATCAAGGGGGGTTTG SSIKGGL 3

GGCTACAACACTTTTAGCGGG GYNTFSG 1

CATGCGTTTGACTTTGCTGCT HAFDFAA 1

TTCAGTATTGACCCCATGGAC FSIDPMD 1

TCGATGGTTACTAGTGCTGGT SMVTSAG 1

CACGTTGGGTGTGTCAGGGGT HVGCVRG 1

TGGCGGCGGTGGCGGCACCAT WRRWRHH 1

CAGAGGGCGGCGGCCCAGTTT QRAAAQF 1

ACGTGGCCCATGCGCCGGAGT TWPMRRS 1

CGTTGTCGCTGCGCTAGTGCT RCRCASA 1

TTGTCCTACGTCACCGGTGGG LSYVTGG 4

TGCTCGCTGCGGTCGGAGTCT CSLRSES 1

GTTGCTGATTTGCTGAGGGAT VADLLRD 1

AGGGCGGCGTGTTATGGCGCG RAACYGA 1

ACGTATGCGAATCGCGTTTCC TYANRVS 1

TGCTGGTTCTCGCTGCGCCGT CWFSLRR 1

GCGCGCTGGTTCGACGCTTTT ARWFDAF 3

AATGGGGTCAGCTTCGAGTTG NGVSFEL 2

GCCCCCGAGCGCTCTTCGGAT APERSSD 1

TGGGGTCGCGTCGGCTCCCCT WGRVGSP 1

TTGTGTGACTCTCGCCAGTTG LCDSRQL 1

AGTGTGGGTCGGTGGATCTCT SVGRWIS 1

TTCAGGCAGCTGAGGAGGCTT FRQLRRL 1

GTGGACGTCGGGGTGTCGTCG VDVGVSS 1

CGCGGCATCTGGTGCAAGTGT RGIWCKC 1

AAGGATGATCTGCTGGGGACC KDDLLGT 1

TCTGCGGGCGGCTCCATCGAC SAGGSID 1

CAGTCGTATCGTGTCCTGCCT QSYRVLP 1

CGCCGGAGCTCGGTCTTGAAT RRSSVLN 1

TGCCCCGACGATAGCCGCCGG CPDDSRR 1

TTCGCTGGGTCTGGGAAGTTG FAGSGKL 1

CCGCTGAGTCCCTTGGCGACT PLSPLAT 1

GTTAGGGCCTGTCATGGGCCT VRACHGP 1

ACGCCTCATGAGATGCGTGAG TPHEMRE 1

TACGTGCGGCTGCCTGCGTTG YVRLPAL 1

GACTCTAGGTTCCACTCGCGG DSRFHSR 4

GGGAACGTCATGAGGGCGTTC GNVMRAF 1

AGGCCGCAGTTGGGGACGGCG RPQLGTA 2

TTCGAGGTCTGGGTTTGGTGT FEVWVWC 1

CCGTTTGACATTGTGCTGGCG PFDIVLA 1

TTGGTCGCTGCGTTCGTCATC LVAAFVI 3

CGGTACAACCAGGCGGTGCGG RYNQAVR 1

CGGTGGCCGGTTTGCGCGCGT RWPVCAR 2

CTGCGGGCTCGCGCTATTCAG LRARAIQ 1

TGTCGCTGCGGGGCCGAGAAG CRCGAEK 1

AGGGACCAGCCTTCCCAGGCT RDQPSQA 1

CACTGCTGTCAGTGCGGGCCT HCCQCGP 1

ATGGTCTTGCTCGGGTCTCTT MVLLGSL 1

GGTTTTCGGTGGGTCAGTTGT GFRWVSC 1

GGTGCGCTCCGCTATGTCCTC GALRYVL 1

CCGGGGGTTCCGTGGCTCACT PGVPWLT 1

GTGTACAGCCCGGTCTGCTGG VYSPVCW 1

GCGTCTTTTTTCGGGTGGGAC ASFFGWD 1

ACGGCCTGCGCGTGCGGGACG TACACGT 1

CCTAGGTGTAAGGGCTATTGG PRCKGYW 1

CTCGTGTCTTTTGCGTATACT LVSFAYT 1

GGCCGGCTGCCGTTGTGGGAT GRLPLWD 1

TCCAGGGGTGGGCGCACGCCC SRGGRTP 1

ATGGGGGCCAGTGTTTCGTGT MGASVSC 1

CTTGGCGCGAGGGATCGCTTT LGARDRF 3

TCGACGCTCAGGCCTGGCGCT STLRPGA 1

GAGCTTACCACTCGCGATCTT ELTTRDL 1

TGCGCTGTCATCCGTTGGCGG CAVIRWR 2

CGGGGTTGCACTCTGGCGGCG RGCTLAA 2

ACTGTCATGCCGGGTGTGGTT TVMPGVV 1

CTTGTGTGTGTGGGCGAGCCG LVCVGEP 1

GGGCCCGGCACGCGTTTGGTC GPGTRLV 1

ATTAGGGGCGCTGTGCTCGGG IRGAVLG 1

GCCGGCCAGTGGCGCCTGTAT AGQWRLY 1

GGTAAGGGCAATAGCGTCGGG GKGNSVG 1

GGGCGCCGCGCTTACGCTACT GRRAYAT 1

AGGACTGGGCTTTGGCATCCG RTGLWHP 1

CTCAATCGGGTCAAGCCGTTT LNRVKPF 1

CGGTTCGTCACCGCCCGGTGC RFVTARC 1

CGTTGGGACGCGCAGGATCGG RWDAQDR 1

GAGAAGCGGGTTGGCCCGTCC EKRVGPS 1

GTTTGGGGCCGGGGCCGCGGC VWGRGRG 1

GCGGGTGTGATTGTGGCCACG AGVIVAT 1

CGGGTGGGTTTGGTCACGTGG RVGLVTW 1

TATGTTGTCCGCAGGGTCAGG YVVRRVR 1

CACGGGATCTTCTTCCGTGTC HGIFFRV 1

TTTAGGCGTCACTCTAGGATT FRRHSRI 1

GTGGCGTTTGGGGGCTGGCTT VAFGGWL 1

AGCGGGGCGACGCGCGTTCAT SGATRVH 1

TGTGACATCATCGGTAGTGCT CDIIGSA 1

GGCGCCTGTGCCCTCGCTTGT GACALAC 2

TACCGCCACCTTGTCCGTGCG YRHLVRA 1

TGGGCTGCTTGGTGTTACCAT WAAWCYH 1

ACGCCGGGCCGCTTTGGTTGT TPGRFGC 1

TGGGGTTATTCTCCGCCTGAG WGYSPPE 1

TTCTATTATATGGTCGATGAT FYYMVDD 1

CGTAGGCAGAACTCGAGGTGT RRQNSRC 2

TTCTACTATATGGATGCCGGT FYYMDAG 1

TTCTGCATTGACATGGACGTC FCIDMDV 1

CTGTTTAGCATGGGGACGTAC LFSMGTY 1

CTGCATGACGGGTGCATTATC LHDGCII 1

CTCCGTGTCGCTGTTCCCGAT LRVAVPD 1

GCGCCGGTTATCGCGGCCGAT APVIAAD 1

CAGTTCGCTCAGTGTGCCAGC QFAQCAS 1

GATTGCGTCGGCGCCCTTAGT DCVGALS 1

TCCCGGTCGGCTGGCGCCAGG SRSAGAR 1

GGGCCGATTCCGGCTGAGCCG GPIPAEP 1

TCGAGGGCCACGTGCCTCCGT SRATCLR 1

GTTCTTAGTGGGGTGGGGATT VLSGVGI 1

AACATGTTTACTCTCACCGGT NMFTLTG 1

ATTACGTGGAGGGTCTCCCGT ITWRVSR 1

CTGGAGCGGTGGTACTCTCAT LERWYSH 1

CCGGTCGTGCCGGTGTTCACC PVVPVFT 1

TGGGCGCTGCCTGGGGTTCGT WALPGVR 1

CAGTGTCTGCGCGGCGTGGCG QCLRGVA 1

TTGTCTCGCAGCGATGAGCAG LSRSDEQ 1

GCGCGGCTCGGCTGGGTGTCT ARLGWVS 1

GCCGTGAGTACGCTCGCGAAC AVSTLAN 1

GGCTCGGTGGTTGCGGTGATT GSVVAVI 1

AGCAGCCAGGCGTGTCCTATG SSQACPM 1

ATGGGGTGTTTGACGAACTCG MGCLTNS 2

GGGCGCCGTGTGTGGCTGTGC GRRVWLC 1

AGTCTGCGGATGTGGCAGTGT SLRMWQC 1

GGGCGGGGTGCTGTCTGCGAT GRGAVCD 2

GAGCTGGCGTCCGGCTACGGG ELASGYG 1

GTCGGTCGTGTGCGTGCCAGC VGRVRAS 2

GTCTCGTCCAGGTTGAACACG VSSRLNT 3

GTGAGGCTCGCTTCTAGTGGC VRLASSG 1

ACGGCGGAGCGTGAGTTCGGT TAEREFG 1

GGGCGGGTGGTGTGCCGTCTT GRVVCRL 1

GGGTGGACTCGGGGCAACGTG GWTRGNV 1

AGCGGTTGGCGCGACAGCGTT SGWRDSV 1

GGTCTTTGTCTGGTCTGCATT GLCLVCI 1

CGGGTGCGGGGTCTCAGGGGT RVRGLRG 1

GACATCTTCAATCATCTGCTG DIFNHLL 1

CTCACTCGTTGGGATGTTGGT LTRWDVG 1

GTGCTCAGCCGTGGCCTCTGC VLSRGLC 1

GGCGACCGCGGCTGGGACCGT GDRGWDR 1

ATGACGTCTTGCCCTAGGAGG MTSCPRR 1

GTGGACTGCCCGCCGGGGTCG VDCPPGS 1

TATAACCGTGGTAGGCGGGTT YNRGRRV 1

TACCGGCTTCTGGCTACGTCT YRLLATS 1

GCGCGGAGGGTCGTGTGGCTT ARRVVWL 1

GTTCATTTGCCCGCTTTTCTG VHLPAFL 1

AGGTCTTACGCTAGCGCGGTT RSYASAV 1

CGGCCCGGCTACCTGGCTAGC RPGYLAS 1

GGTGACAGTTTTATCAGTGCT GDSFISA 1

GAGCACGTGATGCAGGGTTCC EHVMQGS 2

GGGAACAACATGAACGGTCGC GNNMNGR 1

GTGGGGCTTCAGGAGTGTTTG VGLQECL 1

AACACCCGCTCGGAGGAGTAT NTRSEEY 1

TTGGCCGACGGTGGCGGGCTC LADGGGL 1

CAGCAGCGGAGTGATGTGCTT QQRSDVL 1

ATCGACGGCGGCGCCCGTGAT IDGGARD 1

CTTGGGGCGGTCAACCTTCTG LGAVNLL 1

TCTGCGGTGACCCCCGTCCTT SAVTPVL 1

TATCAGGCGGTGGATTTCTCG YQAVDFS 2

CTGACCTACGGCTGTGGTTGT LTYGCGC 1

CAGCTCGTCTACGGTGTTGTG QLVYGVV 1

CACAGCGGCTCGGTGAAGCTG HSGSVKL 1

TGGGTCCTCAGGTTCGAGCGG WVLRFER 1

GCGTGTGCGTCTGTGCTCATT ACASVLI 1

CAGGTTCCGTGCTCCGTCCAG QVPCSVQ 1

CCGACCGAGTTTTTGCTGTTG PTEFLLL 1

GCTAATGTTTGGGGCGGGCCG ANVWGGP 1

TGGATGCACTGCAGGTGGTGT WMHCRWC 1

TTGATCTTCACTGCGCTGGTG LIFTALV 1

AGTTTCGTCTACGGCTCGTGT SFVYGSC 1

GTCGGTGTCTTGAAGCTGAGT VGVLKLS 2

AACGTGTTGTTGAGGGACGCT NVLLRDA 1

GGCGTTGCTGGTAGGAACGCT GVAGRNA 1

TCGCTGGGCTCGGCCTTGGCG SLGSALA 1

GCTTTCAGCAGTTTCGAGTCT AFSSFES 2

TTCGTTCACATCCGCCCTCCG FVHIRPP 1

CTCAGCACTAAGTTGTGGGGC LSTKLWG 1

GACCGGAAGTACTGGTGTAAG DRKYWCK 1

TCCAGCGCGAGTCATTATGAG SSASHYE 1

TGTCGTTCGGGTGGCTGTTCT CRSGGCS 1

GACGGGGGCTTGGTGTTCCCT DGGLVFP 1

CGGTTGCGGTCGGGGGGTTAT RLRSGGY 1

TTGGTTGCTGGGCCTCCCTAT LVAGPPY 1

CGTCGCGGGCTGGACACTGTT RRGLDTV 4

GAGTGGCGTACTCAGTCGTTG EWRTQSL 1

TTTGCTGGGAAGGCCGACCAT FAGKADH 1

CTTATGGGCTTCTCCGTGTGG LMGFSVW 2

TTGCTGATGCTTTCGCCGGGG LLMLSPG 1

TACTTGTTGATTGTGTATCGT YLLIVYR 1

TTGGGGTACAGCAGCTGGGAT LGYSSWD 1

CGCGTCTTGAAGAGTTCTGGG RVLKSSG 2

CCCCGTTCTAGCGCTCTGCAC PRSSALH 1

CGGCCCGACAGCGCGTTGCCG RPDSALP 1

CACTCTCCCAGGCGGATGCGT HSPRRMR 2

GTGCAGGTCTCTCGTGATGTC VQVSRDV 1

CCCCGGTGCTGTCGCGCGTCG PRCCRAS 1

GATGGCCGTGCCGCGGGCAAT DGRAAGN 1

CATGGGCACAGCGGCGGTGAC HGHSGGD 1

TTGCTGGCGAGGGCTTTTCCT LLARAFP 1

GTGGGGTGGGCGAGTAACCGG VGWASNR 1

GTTCGGTGTCCGTCGTCTAGC VRCPSSS 1

ACTGCGCCGGAGTTGCTCGAT TAPELLD 1

TGCTACCAGTGCTGCGAGCTT CYQCCEL 2

GCCAAGCCCAATGCCTGCTGT AKPNACC 2

TCTATGGAGCGCGGCCTCCAT SMERGLH 1

TGGGCGTCCAATAGCGATGGT WASNSDG 1

TTTGGTCTTCCCGTGCGGTCG FGLPVRS 1

GGGTGGGCCAACTTTCTCGGC GWANFLG 1

TGGGGGATGGATGTGACTCTG WGMDVTL 1

CGGATCGGGTTCGGTTTGCTT RIGFGLL 1

TGGGATGCGGGGTTGACCACT WDAGLTT 1

TTGCGTCTGAGTTTCGCGTAC LRLSFAY 1

GACCGCGTCGAGGGTGGTGCC DRVEGGA 1

TGGGGTACCATTTGCATGTGT WGTICMC 1

GTGAATACCTTTGCGCTTACG VNTFALT 1

GTTGCTCGCAGGATCCTCGGT VARRILG 1

GCCGTCTTTCACTCCCGCCGG AVFHSRR 2

GCTTCTGGGTGCCGGTGGCCC ASGCRWP 1

GCCTGTGGCTGTTTGGGTCTG ACGCLGL 2

GTGATCGAGATCGCGTCGTGT VIEIASC 1

TTGCTCGCCGCGTTCTGTGGT LLAAFCG 1

ACCGGTAACGTTGGGTGGAGT TGNVGWS 1

GTTCTGCTCTTGGGCAGCGAC VLLLGSD 1

GATGTCGTGCATTACTTGCTT DVVHYLL 1

CGCGATTGCCGTCAGCATGAG RDCRQHE 1

GATAGCTGGCGCCGGCGGTAT DSWRRRY 1

GGTGTGCGGGACCTCAGCGTG GVRDLSV 1

GCGCATCGCAGGCATCTCTGG AHRRHLW 1

GGCGCTATTGTCTCCCGCCCT GAIVSRP 1

GCCCCGGGGCCTACCGGCCAT APGPTGH 1

TCGCAGAAGAAGCGGAGTCCT SQKKRSP 2

GCGCTTGACGTTCACATGTCG ALDVHMS 1

TGGCGCAGGGTCAGCTACCTT WRRVSYL 1

TTGAGCCACAGTGCTTGGTCG LSHSAWS 1

AGGTGTAAGTTGCAGTGGAGG RCKLQWR 1

TTTAGGTCCGAGCTCTACAGT FRSELYS 1

TTGCAGATGGTTTGTGTTCTT LQMVCVL 1

AGTAAGGGCGAGCCGCGCGTG SKGEPRV 1

TGTATTAGCATCCTCGGGTCT CISILGS 1

CGGCTTGGCGCCGCTTTTCTT RLGAAFL 3

AAGCTGAACAATTGGTGCTCT KLNNWCS 1

TGGAGGGACTTGCAGGGGAGC WRDLQGS 1

CGCCTGTTGCTCTGCTCCTGT RLLLCSC 1

TGGGCGGTTTACATTCTTTCT WAVYILS 1

GAGCGGCTCAAGGTCCTTCCG ERLKVLP 1

TTTAGCTGGGTCTTCCAGCTG FSWVFQL 1

GATGGCTGCTGTGAGATTTTG DGCCEIL 1

CTCCCGGATCAGCTGCTGTTT LPDQLLF 1

GGGGCCCGCTTGTGCGCTACT GARLCAT 1

TGCCTCCTCTTCCGCAGCACG CLLFRST 1

TATCGTCGCTTCTGGCGGTGG YRRFWRW 1

TTGATGGTGCTCCAGGTCGAC LMVLQVD 1

TCGGGCTCTGGTTCGAGGGCG SGSGSRA 1

TTTCTGGTTGGCTGCTTGGCG FLVGCLA 1

TTTGTTGCTGCGGGGCGGAGT FVAAGRS 1

TGGCCGCGCGCGGCGGTGGGT WPRAAVG 1

TCTTCTAAGGCTGTTGGCTGT SSKAVGC 1

ATTCTCTCGGCCGGGTGGCTT ILSAGWL 1

TGGGTTGGCAGGAGTTGTTGC WVGRSCC 1

ATGAAGCGTCTGGAGGTTAGT MKRLEVS 1

CGGCGCGTCAGTCAGGTTCTG RRVSQVL 1

AATGGGGACGTGGTGAGGGGT NGDVVRG 1

GCGATCCCGGTGCAGGTCAGT AIPVQVS 1

GGGACGAGGACGAGTGAGGTG GTRTSEV 1

CTGGTCTTTGCTGGCTACGGT LVFAGYG 1

GGGTTTACCGTGCGCTGGGTT GFTVRWV 2

GTGTGCGGCTCGGCTAGCCGG VCGSASR 1

GCCCGGCGGTGTCGTGTCCCT ARRCRVP 1

CACTTCGTGGCTGGGGTGGTG HFVAGVV 1

GTTGGGGACTCTGCCGAGCTT VGDSAEL 1

TGGCTGGGCGGTGGGCACTGT WLGGGHC 2

GCGACTGTGAGCGATTGTGCT ATVSDCA 1

TTGGCTACTGCCCTCTGGGGT LATALWG 1

CTGGGTCGTTCGCCTCGCCGC LGRSPRR 1

GGGCTCCGTGGGTGGGTGGTT GLRGWVV 1

TTTGGGTCTAAGTGCTCGATT FGSKCSI 2

CCGCACCGTGAGCGGCTTCCG PHRERLP 1

GTGCGCGTGGGGGCGAGGCCG VRVGARP 1

GTCCTTGTGTCTTTCATCGCT VLVSFIA 2

TTTGGGCGCGGGGTTTGCTTG FGRGVCL 1

CGTTGTAATGAGTCTATGGCT RCNESMA 1

TTTGGTCGCGGGCGGGTGCCT FGRGRVP 2

CTGTGTGCGGTGGGGGCCTGG LCAVGAW 1

TTTTGGTCGGGGCGGGATTCC FWSGRDS 1

TTGCAGGTGTGGGGCGCTCGT LQVWGAR 1

GTGTGTTTTGCGCGCCGGTCT VCFARRS 1

ATGCAGAGGAGCGGTTATGAG MQRSGYE 1

CAGCGGAGCATGTACTGTCGT QRSMYCR 1

CGGTTGCATCATGGGTTTCTG RLHHGFL 1

CGTTACCTGTGGTTTCTGGGT RYLWFLG 1

GTTGAGGTGGGCCGTCGCTCC VEVGRRS 1

GTTGCCAGGTTTCTCGTCGGT VARFLVG 1

CACGGGTCTCTCGTGGGTATG HGSLVGM 1

CTGGGTGGCACTTCTGTCAAG LGGTSVK 1

TCTTTGGGTGCGGGGGAGTGG SLGAGEW 1

GTGGCGGACCCTTGCGCTTGT VADPCAC 1

CTTTCTGTGAGCGACTGGCTC LSVSDWL 2

TCGCGGGCGCGGATCGGGCCT SRARIGP 1

CGGCGCATCTGCGGGAGCTAT RRICGSY 2

GTTAACAATAGCCGCAGGTGT VNNSRRC 1

GCGGGGAACAGTAAGAGGAGG AGNSKRR 1

TGTGTTCGTCGGTGGGTGCGT CVRRWVR 2

GTGTGGCATGGTGCGTATTCT VWHGAYS 1

GAGTTTGACGTTTTGCAGCTT EFDVLQL 1

AGCACGTATTGGCGGCCGGTT STYWRPV 1

GCCGCTATCTCGGGGGGGGTT AAISGGV 1

CGCTGCATCGGGGCGTCGACT RCIGAST 1

CGGCTCCATTCCAGTGCCGCT RLHSSAA 1

ATGCGGATGTGTTTTCCGCTG MRMCFPL 1

AAGTCTCTCTGCGACGGCTGG KSLCDGW 1

GTGAGGGGCTGTTTGCTGGGT VRGCLLG 1

CATTCGGACCTCTACGGGCGG HSDLYGR 1

GCGTTGATTGTCGGTCGCCAT ALIVGRH 3

TTTCGTGTTTGCTTTGTTCCT FRVCFVP 1

CTGATTCGTAATGATTCCTGT LIRNDSC 2

GGTTTGCTGAGGCGTGGCTGG GLLRRGW 1

GAGGTCACGTGCCACACCGAT EVTCHTD 1

GGTCTCTTCGTGGTGGCTCGG GLFVVAR 1

CAGTTCGGCTGTAATGGGTTG QFGCNGL 1

CTGGTGCGCAAGGGGCGTGTG LVRKGRV 1

TTGCTGGAGGTCTGGCTGGAG LLEVWLE 1

GAGTGCCCCGGTACCGGTGTC ECPGTGV 1

ATTCATCGCACCCGTAAGTCG IHRTRKS 1

CTCCAGCTTGGGCGCGCGCCG LQLGRAP 1

TCCCTCGCGCTTAAGAGTCGT SLALKSR 1

TGGCTGGAGAGCAGGCGGGCT WLESRRA 1

TCCTCCGGGTGGCCTGCCTCT SSGWPAS 1

GGCGGGAGGTACTTTGTGCAT GGRYFVH 1

CGGTTGCTCCAGTCGAGTGAT RLLQSSD 1

TCGTGCGGGAGCTCCAGGAGG SCGSSRR 1

TGGCCCGAGTGCTGCCCGGCG WPECCPA 1

GAGAGGCATCTGCCTTCTAAT ERHLPSN 1

GGTGGTAGGAGTCATGCGGCT GGRSHAA 1

GGGCTCGTTTGCCCGGCGGCT GLVCPAA 1

GGTGTGCATGCGAGCTCGCTG GVHASSL 1

TCGAAGGTTTTCTCTACTCCG SKVFSTP 1

CGGGCGGGGGCGCTGATGTCG RAGALMS 1

TGCGGTGTGGAGAAGAGGAGT CGVEKRS 1

AGGGTGGTCTTGAGTAGGGGG RVVLSRG 1

AGGCACGTGGGGAGCGTGCAT RHVGSVH 1

CTCCAGGCTAGGCCTCGTGGG LQARPRG 1

TTGGAGATGGTCTGCGTTCTT LEMVCVL 1

TCGGGGCTGCACCTCCCGGGG SGLHLPG 2

GACTTGAGCGCGAGCGTCCCT DLSASVP 1

CCTATTCTTGAGGCGGGCGTT PILEAGV 1

GAGTCGGTGGCGATTTACACT ESVAIYT 1

ATGCTGCACTCCTGCATGGCT MLHSCMA 1

TACCGTGATAGTACCGGTTGT YRDSTGC 1

TTCAACTGCAACTGCATCATG FNCNCIM 1

TCTTTGTTGAGGGCTGGTTCT SLLRAGS 3

GCCTTGGTGATGTGTAAGACG ALVMCKT 1

CGTGGGTTGAGCATGGAGAGT RGLSMES 1

TGTGTTGGCTCTCGTGCTAAC CVGSRAN 1

GTTTATTGGGGCTCCCCGTCG VYWGSPS 1

GCTGGTCATGGCGGCGCCTTG AGHGGAL 1

TGGTTGCCCAGTAGGCGGTTG WLPSRRL 1

GGCAGCGTGTGTAGCGCGCTG GSVCSAL 1

ACGCATCCCCTCGGCGGTGTT THPLGGV 1

GTGGGCACGCACATGCGCTGG VGTHMRW 1

GTGTTTGTGAGGTGCCGGTAT VFVRCRY 1

CAGCGGGGGCTCAGTCGTGGT QRGLSRG 1

TTGGTCTGTACTAGCGTGTAT LVCTSVY 1

TCTGGCCAGAGTCGCCGCTCG SGQSRRS 1

CGGAGTATCCGCGGTGGTATT RSIRGGI 1

TTTGTTCTGAGGGCGCTCCAG FVLRALQ 1

GGCGTCGGGCGGAAGTTCGCG GVGRKFA 3

ACGTTTGAGCCGAAGTGGGAG TFEPKWE 1

CGCGCGCTGACGGTTAATGGT RALTVNG 1

GTGGTGGACATGTTCGGTCGT VVDMFGR 1

TTCACTGTGCGCGCGGGTGTT FTVRAGV 1

CCGAACAAGGTTTTCGAGCAG PNKVFEQ 1

CCGTGGCCCATCATCCGCGTT PWPIIRV 3

GTGGCGACGAACGCGAATACT VATNANT 1

ATGGAGCACATGCGCCGGTCT MEHMRRS 1

CGTTATGAGGACGGTGCCTGT RYEDGAC 1

CCCGAGCCTATTGCGCCTAGG PEPIAPR 1

CGGTATCGGATTCGCGGTCTT RYRIRGL 2

ATGTATCTCTCGAACGGTCCT MYLSNGP 1

ACGCAGGACAAGTACATGAGC TQDKYMS 1

GGGTTGATCTCGCACAAGCAG GLISHKQ 1

CAGATCATTAGCATGGCCGCG QIISMAA 1

AGCTCGCAGGCTAGGGTTCCT SSQARVP 1

GACTCTAACGTTGCTGCTCGT DSNVAAR 1

ACTGAGCGGACCGCTGCTGGG TERTAAG 1

AGTGGCAGGCCCAGTGGCGGT SGRPSGG 1

CTGCTCCCGGATGACCGTAGT LLPDDRS 1

GAGTGTGGGAACCAGTGTTCT ECGNQCS 2

TCCTCGGTCGCTGCTTTTCCG SSVAAFP 1

GTCGGGCTTTCGGTTAGCTGG VGLSVSW 1

CAGTCGCTCCTCCACTTGCTC QSLLHLL 2

TGTGCGTCCGTGTGTACTTAT CASVCTY 1

TTGCCTGGCCGGATTTGGTGT LPGRIWC 1

GCTGGGAGTCAGAGGGTGGTC AGSQRVV 1

TATGGTGTCATCTGGAGCTGC YGVIWSC 1

TACGGGGGCGCGGACACGCAT YGGADTH 1

GTGGCGCGGGACGCTATGTCT VARDAMS 1

GTTTGGATTAGGTGCGAGCCG VWIRCEP 1

CTGTTGCTCTGCTGCCGCAGT LLLCCRS 1

CGGCTGCGGACCGTTTTGACC RLRTVLT 1

AGGGGGCTCAGGCGTTGCTTG RGLRRCL 1

CGGGCTCTGGGGAATGATCGG RALGNDR 1

GGCGGCAGTGTTGGGTTGTGT GGSVGLC 1

GCCGTCATTGGGCGGGGTCCG AVIGRGP 1

GGGAGCCGCGATCGGCTGTTT GSRDRLF 1

GAGTGCGGGATCCGTTATTCT ECGIRYS 1

TACGACTCGTCCGGGGGCTGT YDSSGGC 1

TGGGGTCATGCGGTTGTCCTT WGHAVVL 1

CGTTGGCGGTATTGTGCTGTG RWRYCAV 1

GCGTTTCTGTCTTTTCAGGTG AFLSFQV 1

GACTTCGGTTTGGGGATCCAT DFGLGIH 1

CCTGTGCGCCTGGGTAGCGAG PVRLGSE 1

TGGTTTGAGCGCTTGGGGCAG WFERLGQ 1

CGTAGTACCGCGGGCGGTCGT RSTAGGR 1

TGGAATTCCACCTACGGGGTT WNSTYGV 1

AAGCGGCTGCGGTTGGGCTGT KRLRLGC 2

ATGAGCGGGCGGGCTCGGTGC MSGRARC 1

GATCCCATTGGGCCTGGTTGG DPIGPGW 1

GGGGGGACTGTGATCCAGCTG GGTVIQL 1

TATATTGTCTGGGTGGAGCGG YIVWVER 1

GGCCTCGGCAGCATCTGCAGC GLGSICS 1

TCCGGGCTTGGCAGCCGTTTG SGLGSRL 1

GCTGTTTGGTGGCGGGCTCCG AVWWRAP 1

CGTGACGTCATTTTGTTCGCT RDVILFA 1

ACGATGGTCCTTGTGAATGCG TMVLVNA 1

GCGTTGTCTTTGTTTGCCCAT ALSLFAH 1

CAGTGCCCCGCGGCGACTCCC QCPAATP 1

GAGTGTTTGGCTAATGCCGGT ECLANAG 2

GGTGAGGAGGCCCCTTGCTCT GEEAPCS 1

GTGCGCATGGTGTGTACGTCG VRMVCTS 1

TCTATGCTGGCGCGCGGTTGC SMLARGC 1

TATTATCTTTGGGTTGTCAGC YYLWVVS 1

CGTCTCGCCTGCGATGGGCCT RLACDGP 1

GTTGTTAGTACCCAGGCGTGT VVSTQAC 1

GCTCGTGTGACGTGCATCCGT ARVTCIR 1

GTTGTCCCCATGCGTTCCCGT VVPMRSR 1

TATCTGGCTAAGGCGCCCCAG YLAKAPQ 2

TTGTTGTCGGATAGTTTGCGG LLSDSLR 2

CGTTGGGTGTTTGTCCTCGCG RWVFVLA 1

ATGTTCAGGTTGGCTACGTGG MFRLATW 1

TGGTTCTGGTGCTGGGTTTGG WFWCWVW 1

TCGCTTGGGATTCTCTTTCTT SLGILFL 1

CCGGCCGTGAGCGTGGTGGGT PAVSVVG 1

CACATGAGCGGCGTGCGCAGG HMSGVRR 1

AGTGCTTACGATTTCTTGCCC SAYDFLP 1

AGGGATGGTAGTGCGCCCATT RDGSAPI 1

GTCGCGCGTCTTGTGAGGCCG VARLVRP 1

GGTGACAACATGCGGCCCGAT GDNMRPD 1

ATGCGTCGGCCGGCGTGGAGT MRRPAWS 1

TTTGGTTGCAGGTCGGAGTGT FGCRSEC 1

CTGGGTCCTGGCCTCTCGCTT LGPGLSL 1

TGGCACCCCAATTGCCGGTAT WHPNCRY 1

ATGCACGGTTGGGATTCGCGT MHGWDSR 1

ACGGGTCTCTCCTGGAGCGCG TGLSWSA 1

CGTGGTGAGTGTCGGGATCTT RGECRDL 1

GGGGCGCCGATTAGTTGCTTG GAPISCL 1

CTTACCCGCGACGTGAGTCGC LTRDVSR 1

CACGCGCGCTCTCCCGCGTAT HARSPAY 1

CGTGTCCTGCCCACGGATGTG RVLPTDV 2

TTCTCTCGCTCGTTTGTTAGT FSRSFVS 1

GACATGTTGCTCGTTAGGGGT DMLLVRG 1

TCTCTTAGCCGCAAGGAGTTG SLSRKEL 1

GTGGGTTGCAGCGGCGGGTTT VGCSGGF 1

AGGTGTGTGGAGTTGGTTCAG RCVELVQ 1

CCGCTGAGGGCCGCTATTCAC PLRAAIH 1

CGCTCGCTTTATTCTCGGCGC RSLYSRR 1

TTGATTTTGGGTTTTTTTCAT LILGFFH 1

CAGTGTGGCGCGATGCGTGGT QCGAMRG 1

CGGCCTGTGCGTGTGGGCAGT RPVRVGS 1

CGTGGTGCGTTCTCGTATCTG RGAFSYL 1

ATCGCTCTCGGCGGCTGGAAT IALGGWN 1

CTTTACGGGTGCGGCTGTCCG LYGCGCP 1

AAGAGGCAGGGCGTTAGTAGG KRQGVSR 1

GTGGCTCTTGCTCGGGGTAGG VALARGR 1

CCGATGGGTGGCGCCTGGTAT PMGGAWY 1

TTCATGTGGTACTACTGTATT FMWYYCI 1

GCTGAGGCGTTTCGCCTCGTC AEAFRLV 1

ATGTCTGGGCGCGGCAGTCGT MSGRGSR 1

CCGTGGGCGGTGCCCGAGCGG PWAVPER 1

CCGCTGTGGCGTCGCTCTATG PLWRRSM 1

GGGGTTGTCGTCTGGCGCGCT GVVVWRA 1

GCCGGGCGGAGGCTGCCTGTT AGRRLPV 1

CTGTATTTCGCTCGGGCCGTG LYFARAV 1

TGTTCGCGGGCTGCCTCGTTC CSRAASF 1

ATGTGCTGGACGGCCTGTTCG MCWTACS 1

TTGAGCCTTCGCTTTGACTGG LSLRFDW 1

ATTCAGCGCGAGGGCGTGCCT IQREGVP 1

TTTCCTCATCGCGGGCAGCTC FPHRGQL 1

AAGCAGATCGGGCGGCTGCTT KQIGRLL 1

GTGACTTGTAGCCGCGTCGGG VTCSRVG 1

AGTCGGATCAAGATTTGCACT SRIKICT 1

TTTTGCGAGGCGGAGGACAAG FCEAEDK 1

TGGGCGCGGTTTAAGCGTACG WARFKRT 1

GGCGTCCAGCCTGGGCGTCGT GVQPGRR 1

CACGGTCTGGTGATGGGCCAG HGLVMGQ 1

GGGTATAGCCGGGCGGCGCCG GYSRAAP 2

TACACGCAGGTCGTGGATGCT YTQVVDA 1

CGCTGGCTGCTGTTTCGCGAG RWLLFRE 1

TCTGCGCGGTGGACGTGTGCC SARWTCA 1

TGGGCTAAGGCGGGCGCTGAG WAKAGAE 1

GATGAGCGGTGTGAGAAGGTT DERCEKV 1

GCCGGGGAGAGGAATGCCAAG AGERNAK 1

AGCATGTCCGTTTGTAGTCCG SMSVCSP 1

GTGTCGTGCGCGCTTGGCCGG VSCALGR 1

GACTGTCGCCAGTGTGTTGGG DCRQCVG 1

GGGGAGGGTTCCGCCGTGTCT GEGSAVS 1

GAGTTCAATATGGCTCTTCCC EFNMALP 1

GGGCGGAGCACGCTTGTGGGG GRSTLVG 1

GGGGTGCGGACTCATGGTGCG GVRTHGA 1

CGGGTGGACTCGTTGGATCTT RVDSLDL 1

GTCCGGCTTTTGCGCTGCCCT VRLLRCP 1

CCCAATCGGGTCGGTAGGGTG PNRVGRV 1

CGTTGTCTGCCGGTCACGACG RCLPVTT 1

GTCCCCGGTGATGGGAAGAGT VPGDGKS 2

TGTAGGGACCTGGCTGGCCGG CRDLAGR 2

AATCCGCCGGTTGGGATGTAT NPPVGMY 3

TACCATTTGATGCTGTCTCAT YHLMLSH 1

TGCCGGGGCTGCCGTGAGCGT CRGCRER 1

GCTGCCGCGCAGATCCGGCAG AAAQIRQ 1

CTGCAGGTTTGGGGTTCTGTT LQVWGSV 1

GCGAGCGTCAGGCTTGCCGGT ASVRLAG 1

GCCAGGCACCGCGCCTGGCAT ARHRAWH 1

ATTCGGAGGATGCGCCACGCT IRRMRHA 1

CTTTACTGGAGCGAGGCTGTT LYWSEAV 1

CAGGGGCTCGTGGGCTACCTT QGLVGYL 1

CTTGAGTTGGACAGGTCCGCG LELDRSA 1

CGCAGCTCCGATTGTTTTGGT RSSDCFG 1

GGTGACGATTGTGTGAGGGGG GDDCVRG 1

GAGCGTTTGTCTATGCGTGTT ERLSMRV 1

GTGCTGTGCATCATCCCGGAG VLCIIPE 1

CCGACTCAGATCAGCATTCCT PTQISIP 1

CGGGCTTTTCCTCTCTCTGGT RAFPLSG 1

CGGGTTCGTAGGGATCCTCTT RVRRDPL 1

GCGCTGGCCGTGTCTCCTTCG ALAVSPS 1

ACGCTTGGCGCTTGCACCCGG TLGACTR 1

AGGGTTGTCTATCGCCGCGGC RVVYRRG 1

ATCTTGCTTTCGTACTTGCTT ILLSYLL 2

TGTGTTTCTTGGATGGCGGCT CVSWMAA 1

GTGCCGCCGCTGGGGGAGGCG VPPLGEA 1

CGGGTCGTGGATTGCGCTTGG RVVDCAW 1

CTCCAGTTCTCGCCTCCTTAT LQFSPPY 1

GCGAGTATTTATCAGTTCTCT ASIYQFS 1

GTGTCCAGTACTCATTCGTGC VSSTHSC 1

GGGCTCGGTTCGGACAGGGCT GLGSDRA 1

CTGGTGAGTCCCTACTCCGAT LVSPYSD 1

CGTTGTGTGGGCTGCGTCCCG RCVGCVP 1

CCGCGTCAGGCGGCCGGTCAG PRQAAGQ 1

GTCTGGAATGTGGCTCTCTCT VWNVALS 1

CTGCATCTCTCTGGTCGGCCC LHLSGRP 1

CTTACTCGGAGTTGGCGCCTT LTRSWRL 1

ATGTGGCCGGGTGGGATGGGG MWPGGMG 1

TGGCGTGCTCTGAGCGTGTCT WRALSVS 1

TCTGGGGGCCCGCCCCAGGGT SGGPPQG 1

CCGGTGGCCGCGCTCATTGAG PVAALIE 1

GACATCACGGGTTGGCTCTGC DITGWLC 1

ATCTGCCACGTTCTGCGGACC ICHVLRT 1

CTGCGGAGTACCTTGCGGATT LRSTLRI 1

GTCGATGCGTCTGGGGCTTGT VDASGAC 1

CGGTTGCCTGGTGTCTGCTGT RLPGVCC 1

TTTTGCCATAATTCTGTTGCT FCHNSVA 1

TTCTTCCTGAGGAGCGTTGGT FFLRSVG 1

GCGGCTTGCCACTGTCGGTTT AACHCRF 1

AGTGTGGAGGTGTGCTTTCCT SVEVCFP 1

GTTAAGGGCTGGGACGTGAGT VKGWDVS 1

CACTTCGTTCCCAAGCCGCCT HFVPKPP 1

CACCGTCGCTGGTGGGATTTT HRRWWDF 1

GGGTCCTTGGGCGTGTATTTC GSLGVYF 1

GACGGGCCCTGCTGCTTCCAG DGPCCFQ 1

GGGGTTGGGCGGTTTGCGTTT GVGRFAF 1

AGGCCTCTTCCGGGCAATTTT RPLPGNF 1

GGGCGCGGGTCGACCCGCATG GRGSTRM 1

GGGCTTGTTCTGTGGTGTACT GLVLWCT 1

GCTGCCTCCCGTAATCTCTGG AASRNLW 1

GAGAAGTTTGGGCTGCTTCAG EKFGLLQ 1

CACGTGATTCCCCTCGGCGTT HVIPLGV 1

GGGCGGCGCACCGTTGACATG GRRTVDM 1

TGTGTTGTGACGCTGTGGCTG CVVTLWL 1

AGGGTCTTGTGGGATCTCAAG RVLWDLK 1

GACCAGTTGTACATCCCGCCG DQLYIPP 1

TTTTTGGCGTGGACTGCGCCC FLAWTAP 1

TGTGTCCTGCTGGCTGTTCTG CVLLAVL 1

TGTTGCCCGACTTGCAGGCGT CCPTCRR 1

CGCGTGTTCCATTTCGTGTGG RVFHFVW 1

GTGCGCGGGTCGGCCAGGTGT VRGSARC 1

TTTTCGCGGAAGAGGTTGGGG FSRKRLG 1

GTGGCTTGTTGGTCGAGTGTC VACWSSV 1

GAGGGCAAGCGGGCCACCGAG EGKRATE 1

TTCTTCTGTGCCTACGTTGTT FFCAYVV 1

GCGTTGGTCCTTTTTGTTTTG ALVLFVL 1

AGGGGGGGCCTGGCGGGTGTG RGGLAGV 1

GGGTGGGACGTCGAGCACTGG GWDVEHW 1

CGGCGGTTCCTTAGTAGCTGG RRFLSSW 1

GGGGATCGGTCGAAGGTTTGG GDRSKVW 2

CACTGTCGGGGGCGCATCCGC HCRGRIR 1

GCGACTGTGCCCCTTGCCCGG ATVPLAR 1

TGGACTCAGGCCCTGGCCGGC WTQALAG 1

GCGAGGTGCATGGATTCTATC ARCMDSI 1

CGGAGTTGGTTTGTTCTCATC RSWFVLI 1

CTCGCGTCGTCGGGGGGTTGT LASSGGC 1

CATAGTGGTACGTACGAGGCT HSGTYEA 1

GGTTTGCTTGGCGCGGTGCAG GLLGAVQ 1

TACTGTCTCTCGTTTAAGAGG YCLSFKR 2

TGGAATGTTTGCGGGGATGGC WNVCGDG 1

GCGGTTCGCCGCGAGCTGATT AVRRELI 3

GAGCGCCCTGGCTCGAGGTAT ERPGSRY 1

CCTCTTGAGAGCGGGCGGTCC PLESGRS 1

AGTTGGCTTCGGTGTCGCCCC SWLRCRP 1

GGGTTGAACAACGCGGAGAGT GLNNAES 1

TGCAGCTATAGCGGGATTTAT CSYSGIY 1

CGCCGGGAGATGTGGCGCCTG RREMWRL 1

AGTGGTGCGCGCCGCGTGTCG SGARRVS 1

TGGGAGGTCGGTGAGAGGTGT WEVGERC 1

CTTTCGTTTGCTGGTACTTGT LSFAGTC 1

CGGGGCGCTGCTAACAAGTGG RGAANKW 1

CATTTGCTGCGCGCGAGGCTG HLLRARL 1

GTGTGCTGTGGTCCTGACTCG VCCGPDS 1

TTGGCCGAGCTGTTCGAGGTG LAELFEV 1

GTTGGCGTGTTTGTTCTGTCT VGVFVLS 1

AATTTGTTCGACAGTTGTAGT NLFDSCS 1

GCTCTGAATAGCATGGCTATT ALNSMAI 1

AGCTGGTCCAGCGTCGATACG SWSSVDT 1

ATTATCCCCTGCCTCCCGGGT IIPCLPG 1

GCTCGCGAGATCTTGTCCAGG AREILSR 1

CGCTCCCGTGCCGTCGACGGT RSRAVDG 2

GGGGAGCACGTGAGGTGGCGT GEHVRWR 1

TCTTATGAGATCGACCTGTTC SYEIDLF 1

GAGCGTCGTCTTGCTCACTGG ERRLAHW 1

GTGGTGATTAAGTTGCTGCGG VVIKLLR 1

GACCCGGATAGGCGCAGGGAT DPDRRRD 1

GGTTACACGGAGTGTAGTGGG GYTECSG 1

GGTCCGGTTGTCGCCAAGGGT GPVVAKG 1

TGGGTCTGCTGGCAGGGCGCG WVCWQGA 2

GTCTGGCTCAAGGGGGACTCC VWLKGDS 1

TTCTGGAGGGTCAATACGGCG FWRVNTA 1

CGCAGTGGGAGGGTCGTTCAG RSGRVVQ 1

GAGTTTAGCTGGGCTCCTGCT EFSWAPA 2

AGTCTTGCGACGGGGATCGTT SLATGIV 1

GGGGCCAGCATTGGGGGCTTG GASIGGL 1

GTCAACTTTCATCGCGTGACG VNFHRVT 1

GCCGGGATGCTCTGGGGTCGG AGMLWGR 2

TGGACTGATCTTCATTGTAGG WTDLHCR 1

AGGTTCCATGGGTCTGTCACG RFHGSVT 1

CTGCAGCACTATGCCTCTTGG LQHYASW 2

GAGGCGCATAGCTCGATTGAT EAHSSID 1

TACTTCCAGTCCAGGGCCTGG YFQSRAW 1

GTCCGGTGCTTCGGTGTCCGG VRCFGVR 1

GGTCTGCGGCGCGAGGGCCAT GLRREGH 1

AAGGCGTTCTGGGATTGTCTT KAFWDCL 1

TGCGTTACCGTCCTGGACGTT CVTVLDV 1

CGGAGGAATAGCAATCAGTAC RRNSNQY 1

GCTACTGATACGTCTGGGAGT ATDTSGS 1

TGGACGCTTCTTGCGGGTCCG WTLLAGP 1

TGCCGGGGCTTCGCTCTGCTG CRGFALL 1

TCTTTCCGTCTCTGTAGCAGG SFRLCSR 1

ATGTGTTGCTGGGCGTGGGCT MCCWAWA 1

GATGCCAACTCCCGCATTTGT DANSRIC 1

TATTCTGCGAACGCTTGGTCT YSANAWS 1

TCGAATCGTATCGTGGGCTGG SNRIVGW 1

AGTGGGCGTTTGCTTCCTCCG SGRLLPP 1

AATTGTCACGGTCTGTTGGGT NCHGLLG 1

TCCCCTGCCCTTGTGATTCCT SPALVIP 1

GGTTTCCTCGTTCGGGAGGTC GFLVREV 1

AACTTGCAGGCGTGCGTTGGT NLQACVG 1

GTTGCCAGCTGGAGTTGGGTT VASWSWV 1

CCGCTTGCGTGGTGGTGTTCT PLAWWCS 1

CGGCTCGCGGGTGCGGTGCCT RLAGAVP 1

TCGCGTCTTCCCGAGCTTCTC SRLPELL 1

CCCTGGCCTAGTTCGCTGGTC PWPSSLV 1

ACCGAGGCCCTCTGGGGCGAG TEALWGE 1

GGCTTTCGCCAGTTGCGGACC GFRQLRT 1

GTGCGGGAGTGGCCGCCGTGG VREWPPW 2

GTCCCTCTGACCTATCCGGCG VPLTYPA 1

ATCTTGCCGAGCAACTTGTCT ILPSNLS 1

GCTCGCCGGTCCTGCATGAAG ARRSCMK 1

ACCGCGGGCGGCTGCTGGCGT TAGGCWR 1

CACCGTCCTGTGAGCTTGAGT HRPVSLS 1

GGTCGCGGCAGTGGCCTTTAT GRGSGLY 1

GGGTGTCTCGTGAGCTGCGAT GCLVSCD 1

AGCCCTCCTTCGGGCGGGCCG SPPSGGP 1

GTGAAGAGTACGACCGTTATC VKSTTVI 1

CTTGCCTCGGGGGGCGTTGAG LASGGVE 1

GCGGCGTGCTCTCTCTGTATT AACSLCI 1

GGCAGCCTGGTCTTCTGGGTG GSLVFWV 1

GCTATTTGCAGTTCGGACGAT AICSSDD 1

CTGTGTAATTGTTGGTGCTAC LCNCWCY 1

GCCGTGCATACGTTCGAGGCG AVHTFEA 1

GGCCGGCTGCAGTGCGGCCAT GRLQCGH 1

CGGGTGGGGATTGCGTCCAGG RVGIASR 1

GTCCTTTGGCGGTGCGAGACT VLWRCET 1

TGGGGCCTGTCGGTGCCCAGT WGLSVPS 1

GTCCACTCGATTCTTGGCTGT VHSILGC 1

ATTACCCCCAGTTTCGTGGTG ITPSFVV 1

GCTAGCGACCTGTGTCGGCAT ASDLCRH 1

TATGTTAGGAGGCAGTCTTGG YVRRQSW 2

TTTCGGCACCTGCGCGCTCGT FRHLRAR 1

GGGTGGGTCGATTGCAGTCGG GWVDCSR 1

GGCTTGGACCCTAGCTGGCAT GLDPSWH 1

GGCGTCATTCTGGGCATGGTC GVILGMV 1

AACAGTTCGCCTTTCCCGCAG NSSPFPQ 1

ATCGTTGTTGTGGCTCGGGCT IVVVARA 1

GCGATGGGTGCCCGTGGTAAT AMGARGN 1

GTGCATGGCGTGGCGGCGTCT VHGVAAS 1

CGGTATAACCTTAAGTATTCT RYNLKYS 1

CGCAAGGGCGGTCTCGTCGGC RKGGLVG 1

AACCAGTTGATCGCGCTGCGG NQLIALR 1

TCGCTCAAGCTTCGCGGCGCG SLKLRGA 1

GTGTTCTACGCCGGCTTTAGC VFYAGFS 3

GACTCGTGCGTGGCCGGTGCT DSCVAGA 1

CACGTGGCGCACGCCTTGGCT HVAHALA 1

GTTTCTACGCACGGTGGGCTG VSTHGGL 1

GCCGGGTGCGATAGGAGTGGT AGCDRSG 1

TCTGCGGAGAGGCGTAAGAGT SAERRKS 1

TCTTCCGCGAGGCGCATTGGG SSARRIG 1

CGCTACGCCTTTACTACCCTT RYAFTTL 1

GGTCGGCATTCGCGTCGTTCT GRHSRRS 1

CGGCACAGGTTCCATGGGTGG RHRFHGW 1

ATCGGTCCCACCTTCGCCCCT IGPTFAP 1

GAGAGTCCTAGTTTGGAGCCT ESPSLEP 1

ATGCTGCCTATGGGTTGTACT MLPMGCT 1

TGCCTGAGCAGGGGCCTTCAT CLSRGLH 1

TCTTGGTCGCCGCGTTGGTGG SWSPRWW 1

GGCGGGTGTAGCCTCATTATG GGCSLIM 1

TGTCGTTGCTACGACGGGGTT CRCYDGV 1

GCTATTGGTCTGACGAAGTGT AIGLTKC 1

ACCCTTCTCATGTGGATGGGT TLLMWMG 1

CGGACGATGGATGTGGGCTTT RTMDVGF 1

CGGTTGTGCTTGTGCCGCCAT RLCLCRH 1

CTTGTGCTCATGTGTGGCCGG LVLMCGR 1

TGTCTCGCGTACCGGCCGCGT CLAYRPR 1

CTTATGACCTTGGCTCGGGGG LMTLARG 1

GGTCCTATGCCTCAGTGGGTG GPMPQWV 1

GGCTGGAGTCAGTCTATTAAT GWSQSIN 1

CCTTTGGTGTTCGGCGGCCGT PLVFGGR 1

TCGTGCGACAGCAGCCCGGGT SCDSSPG 2

GGTCTGAGTGTGAGCGCTATT GLSVSAI 1

CGGGCCGTGAGTGCGAGGGTG RAVSARV 1

GGTATGTCGCCTGTGAACTGT GMSPVNC 1

AACCTCAAGGTTGTTGCGGTG NLKVVAV 1

TTGCGCCGCTGGGTCACTGTT LRRWVTV 1

TCGGAGCTTAGCTGTGGGCGT SELSCGR 1

TTGGTCGGCGCGACCGTGTCG LVGATVS 1

TTTACGCTCAATTTGCGTACT FTLNLRT 1

CTCCTCGTGGAGGAGGTTCCC LLVEEVP 1

TGTGACGACGCTATGATGGGT CDDAMMG 1

GAGCGTCACGGCGGGAGGATG ERHGGRM 1

CGGAGCTACGCTTGGGCGCGG RSYAWAR 2

CCTACGGTTTGTTGCGAGGTG PTVCCEV 1

ATGCTGCCCTTTAGTGGGCGG MLPFSGR 1

AATTGTCCTTCCAAGCCGGCG NCPSKPA 1

GTCTCGCGTCCGCGCCTGCGT VSRPRLR 1

TTTCAGCGCAGCTTCAGGTGT FQRSFRC 1

CCGTATGGCGAGGGCCCTGAT PYGEGPD 1

AGCGGGCATAGCTGGTCCTTT SGHSWSF 1

AACTCCGTGGCGGCGTTCAGG NSVAAFR 1

TTGACTCTGAGGATCGACGCT LTLRIDA 1

GACGGCAGGGCGCTTCCTGTT DGRALPV 1

GGTCCTCGCGGCGCTAGGATG GPRGARM 1

GCGTGGGAGAGGTGTAGCTGT AWERCSC 1

ACGTACGACCGCCCGGATCGC TYDRPDR 1

CTTCGTCGGTGGCGGCCGTTG LRRWRPL 1

CTTTTGGACACGTATGGGCAT LLDTYGH 1

GTCCGGCGGAGCGCGGGCCGT VRRSAGR 1

GTGCAGCGCGCGGAGGGCGGG VQRAEGG 1

CCTCTGATCGTCAGGAGTGTT PLIVRSV 1

CGCGGGGTTCCCCGCCGCGAT RGVPRRD 1

TATTGCGATGACGTGGTGCAG YCDDVVQ 1

CTCGTTGTTAGGGCGGACCCG LVVRADP 1

TGGAAGCTTTACATCTCGCCT WKLYISP 1

AGCGGGTGGAGCGCTACTCTT SGWSATL 1

TTGGGGCTCGATCCGGTTCCT LGLDPVP 2

GTTAAGCGTATCTCGAGTGGC VKRISSG 1

AGGAGGTGTGGGCAGTCGAGT RRCGQSS 1

TTGTTGGCGCGTGAGCTCGAC LLARELD 1

CATATGGAGCGGGGCGCGTTT HMERGAF 1

AGTATTGCGTTGAGTTCCCCT SIALSSP 1

GTCGCGCGCGCTGTTCGCTGT VARAVRC 1

TGCCAGTTGCCGTATGATTGT CQLPYDC 1

ATGGGGTTCGGGCGCATTCGC MGFGRIR 1

CGCCGTTCGATCATGTTGAGT RRSIMLS 2

GTGCCGTACGACCTCGACGTC VPYDLDV 1

CTGTGGAAGTCTACCGAGTTC LWKSTEF 1

GGCGCGTGTGTGGTCGGGAGT GACVVGS 1

TGCTCGTTGGTCGGTAATGTT CSLVGNV 1

ATGCTTCCCCAGGCCCGGAGT MLPQARS 1

GATCCTCACCGTGGTGCGTCG DPHRGAS 1

AGTGGTCACGGTACTCGTGTG SGHGTRV 1

ATGAGCAGCGGGCGTGGCCCG MSSGRGP 1

TCGCTTCCTCATAGTGGTGGT SLPHSGG 1

TGCCCCGACGTTCGCGATCTT CPDVRDL 1

CGGGCTGTTAAGACGTGGTTG RAVKTWL 1

TCCCAGGCTTTCGGGTCCGGT SQAFGSG 1

TTTAGTGTGGGCTTGTGGCAG FSVGLWQ 2

TTGTCCTTGCGGCAGCGCTGG LSLRQRW 2

AGTGCGGTTAGGCGGGGTGCT SAVRRGA 1

GTCTGGGCCAGGGTGGACCTT VWARVDL 1

CTCCTCAAGGTGGCCCAGGCT LLKVAQA 1

GTCTGGGCGTCCGCCACTTCG VWASATS 1

GCGGGGCGTTCGGCGGGGGTT AGRSAGV 1

ACCGTGTCGCCGGGGAAGCGG TVSPGKR 1

AGCGGGATTATCAGTAGTGAC SGIISSD 1

GTGGTTCGCCCTGTGGCTAGG VVRPVAR 1

AGCCGGGGCAGCTTGGATGGT SRGSLDG 1

TTGTTCGACGTCGTCAGGCGG LFDVVRR 1

GGTGACAGTGTCGCTTGCCGT GDSVACR 1

TGCGTCGGGACCCCGGAGGTT CVGTPEV 1

GGGCTGCTGAGTACGGTGCGT GLLSTVR 1

GGGGCGTTGTGGTCCTCCGCT GALWSSA 1

TGGGGTACGTGTTCTGGGGCT WGTCSGA 1

GCCGCGAGCACTAATGAGAGT AASTNES 1

ATGTCGTTCAGGCCGGACCAC MSFRPDH 1

ATCATCACGCTGGAGTGCACT IITLECT 1

GCTCGGGGCGCGTGTGAGGGT ARGACEG 1

CTGACGCAGATCCAGCTTTAT LTQIQLY 1

TGGGGCTGTATCGTCACTCAG WGCIVTQ 1

GCTGTGATTGTTTCGTATCTG AVIVSYL 2

GATATGTGCTTCGGGGTGGCG DMCFGVA 1

TTTGGGTCCACGATCGTCCAT FGSTIVH 1

AGGCGCTGCGGGGCGCGCCCT RRCGARP 1

TGGTTGTCGCCGGCCGATCGT WLSPADR 1

GGGGGCGCGGTTGCGGTGGGT GGAVAVG 1

CCCGTCAGGTTTGGCCACGCG PVRFGHA 1

TTTTCGTATGGGTCCAGTCGG FSYGSSR 1

AAGAAGCAGTGGTGCGAGTTG KKQWCEL 1

CGGTGTGCCAGCCGGATTCGT RCASRIR 1

CAGGGCCCGATTACTTTCGCG QGPITFA 1

GCGCAGGTGAGGGGCCGGATT AQVRGRI 1

GTTTCGGAGGGTTGCTGGGGT VSEGCWG 1

CTGTTGTTGGTCACCTCCATG LLLVTSM 1

CGCGCGGTTAGCGTCGGGTTG RAVSVGL 1

AGCTCTAAGGGGAACTGCGGT SSKGNCG 1

ACCTACAGGGATTTGGCGCGT TYRDLAR 1

TTCCCTCTCGGCGATCACATT FPLGDHI 1

CTTCCGCGCTGCTTTATCGCG LPRCFIA 1

TTCCAGCGCTTCACTGGCTAT FQRFTGY 1

TGGTCGGGGTTTTGCGACGTG WSGFCDV 1

GTGTGGATGAGCGCTCTTCCG VWMSALP 1

GTCTGGTTCCAGATCGCGGCT VWFQIAA 1

CTCGCGGGGAATTTGGTGTGC LAGNLVC 1

CTCGTGTTTCCCGAGTTTCAC LVFPEFH 1

GCGTTGTGCAGGCGCGGCATT ALCRRGI 1

GGGTCCTTCAAGTTTTGTCAC GSFKFCH 1

GTGGTGTCGAGGCACAGCCCT VVSRHSP 1

GGCCGGTGTAGGTGGTATGCG GRCRWYA 1

ATCTTCGGCACTGAGGCGTAT IFGTEAY 1

GGTAGGCCGATCGTGTGTCTT GRPIVCL 1

CCTGGCTTCTACTCGGCTTCT PGFYSAS 1

GGCGCTCAGAAGATGGATCTT GAQKMDL 1

GTGAGCTGCGCGCTCGCGCGT VSCALAR 1

AATGCCGGGGCGCAGTGGCCC NAGAQWP 1

AAGGTGCAGATGCTGATGTTG KVQMLML 2

GGGTGGCGCTTGCGTTTGCGG GWRLRLR 1

GTTGGCTGGCTTGGCCAGAGC VGWLGQS 1

GTGTGCCACGGCCTGTATCCT VCHGLYP 2

ATGAGCAGTAGCAGCGCCGAT MSSSSAD 1

CTTCAGCGTGGCAGCGTGCGG LQRGSVR 1

GGGCATCCGCTGCTCCGGAAC GHPLLRN 1

GTTGGGCAGTGCATTTGTCGT VGQCICR 1

GGTCCTTTGTGCTCTGCCTCT GPLCSAS 2

GAGTGTCGGAGCGGGTGGGAT ECRSGWD 1

GTTGATTACGCCTGCGTCCAG VDYACVQ 1

ATGGAGGTCCCGTTTCGGTCT MEVPFRS 1

GTCTCGGTGCTGCTCTGTTGT VSVLLCC 1

TACTTGTCGGCCTGTCCTTTG YLSACPL 1

GGGCTGGAGGCCAGTACTCAC GLEASTH 1

CTGCATCGCGCGTGCCGTTTG LHRACRL 1

GCCGGGCGCTTCGGCCAGTAT AGRFGQY 1

CTGCACCGGGTTCTCTGGGAG LHRVLWE 1

TCTACCTATCCGGGCGCGGAT STYPGAD 1

ACTTGTAACGGTTGCATCACG TCNGCIT 1

CGCAACATCAGCTTGGGTCTG RNISLGL 1

GTCGGCCGGGGCATCCTCTTG VGRGILL 1

TTTGGGACTCGGCTTTTGCGT FGTRLLR 1

TGCCGGGTTGGTGGTGATCCG CRVGGDP 1

CGCTGGCCGTGGTCTTATACG RWPWSYT 1

AGGTTTGAGGGCGGCTGTCCT RFEGGCP 1

CGCGGGAATTCTGAGTGGGGT RGNSEWG 1

CATTGGAATGGGGACTGTCTG HWNGDCL 2

TCTTGGCGGATGCTGCAGTGG SWRMLQW 1

ACGTTGATTAGTTCCCGCGGC TLISSRG 1

ATGCTGGGCCGGTGCTTCTGT MLGRCFC 1

TCTCCCACGTCGGATGGGCCG SPTSDGP 1

GGGGAGGCGCTTGTGTTGCCG GEALVLP 1

CATTTGGATGTGGGCTCGTCG HLDVGSS 1

TTTTTTGAGGGCAATGTGCAT FFEGNVH 1

TATACGGGGCCCGGCTTGCAG YTGPGLQ 1

GGGAGGTCCCCTAACCCTCAG GRSPNPQ 1

AGTGAGTGCTCGGCTTTCGCT SECSAFA 1

TCTGTGCTGATGGTCCGGACG SVLMVRT 1

TTTCCGTTCGAGTCTACGCGT FPFESTR 1

GATGGGACTATTTGCGGGGTT DGTICGV 1

CGGGCGTTCTCTTTGGGTGAT RAFSLGD 1

CTTAGCGGCTGGCTCGGGTTT LSGWLGF 1

GTTATTCGGGCCGAGGATTCG VIRAEDS 1

GCCGATAACGCTATCTCGCCC ADNAISP 1

AATAGGGGCCAGTGCAGTGGT NRGQCSG 1

GGGTTGGTGTGCGAGTGGATT GLVCEWI 1

TCCTTGGGGGCTACGCTCTCC SLGATLS 1

TTCCCGGAGGTGCTGGTGGCG FPEVLVA 2

GTCCTTGGGACGTATCGCGAT VLGTYRD 1

GCTCTGCTGACTGCTACGCTG ALLTATL 1

CTCAGGTACCATTGCTTTGAG LRYHCFE 1

GATCTGGTTCGGCACTGGGCT DLVRHWA 1

GTGCATCCTAAGCCCCGGACT VHPKPRT 1

TACTGGGACGAGTCCGGTGAG YWDESGE 1

GTTGGCAAGAGGGCCACCGCG VGKRATA 1

AGCTCTCTCGACTATGTTCAT SSLDYVH 1

CGGTGGGCCGGGAAGGGTGGT RWAGKGG 1

CGTTGGATCGCTTTGTGGCAT RWIALWH 1

GTCGCTCATATGTGCGGGGCT VAHMCGA 1

ATGGACCGCGGGGCCGAGGCG MDRGAEA 1

GCGCGGCCGTTCGTCGCTAAT ARPFVAN 1

TCCCGCTCTGCTACGGACCAT SRSATDH 1

GCTCCTTGGACTGTGGATCTC APWTVDL 1

GTGCACCACAAGGTGGTTTCG VHHKVVS 1

TTCTGTTGGTGCGGGGAGCGG FCWCGER 1

CATAGTTTGTTGCGGCGTGGT HSLLRRG 1

GCGGGCTGGGTTGTCCGGGAT AGWVVRD 1

GGTGAGGCGCCTGGCTTCTCT GEAPGFS 1

GGGTGTGCTCTGCGCGTCCAT GCALRVH 1

AACCGTTGCCGTCGGCCTCCT NRCRRPP 1

GGGATGCGTAGTGCGCTTCCG GMRSALP 1

TGTCCCAGCTACCACTGCTTT CPSYHCF 1

CCGTCTCTTAGTAATTCTATT PSLSNSI 1

TCGATGGAGGTGGATTGGTTG SMEVDWL 5

GCGCATGACTGGCCTCACTTC AHDWPHF 1

GGGTCGGCTAGGGCTTCTGTG GSARASV 1

ACCGGGAAGTGGGGCTCGCGT TGKWGSR 1

GGGGGGGTCGCCCTTTTGCTG GGVALLL 1

GGCGCGGGGAGGTTCGCCGTG GAGRFAV 1

GAGGGGAATTGTTGGTCGCAT EGNCWSH 3

GTTAGCGCGGGGTACGTTCGG VSAGYVR 1

GGCGGCGTCTGTGTGATGACG GGVCVMT 1

GGCGGGGCGCCTGACTTGGGT GGAPDLG 1

GCGGGTGTTGTTTCGCTTTGT AGVVSLC 1

AGGTGGCAGATCCGTGGGCCG RWQIRGP 1

GCTGCTCCTTGGTGCTGTCTT AAPWCCL 1

CGTGGCTGGCGCCCCTACCGT RGWRPYR 1

TTGGTTCGGAGTGGCGAGTCG LVRSGES 1

CCGGCGTGTAAGGAGTGTCCG PACKECP 1

TGGGCCAGGGCTCCCGCTTTC WARAPAF 1

AGCCTGAGGGAGTTGGGTTGT SLRELGC 1

TGGCATGGCAGGCTGGGTCGC WHGRLGR 1

CTTCGGCCGCTGTGGGATTTG LRPLWDL 1

ATCAACTGTGTGCCCGGCGGT INCVPGG 1

TTGGTGCTTGTCCGCAACGAT LVLVRND 1

CATATGGCGCCGCCGCATACT HMAPPHT 1

AGTCTTGGGTGCTACGCCGCT SLGCYAA 1

GTCGGGGCTGGCAACTCCGGC VGAGNSG 1

GGTTTTAACGTTGCGGAGCGG GFNVAER 1

GCGCGGATGCTGCACGCTGTT ARMLHAV 1

CGGAGGGGTGATTGGTGGTCT RRGDWWS 1

TGTGGCTGCCTGTGCTGCGAT CGCLCCD 1

CGGGTCCCGGCGGGCATCTCT RVPAGIS 1

GCGGCCGATATGGGTTGCCCG AADMGCP 1

TTGTGTTTTATTCCCGAGTCG LCFIPES 1

TGTGCGTGGCGGGCCCTTCCG CAWRALP 1

ATGGACGTTAGTATGGCGAGG MDVSMAR 1

GTGAGCGTGCAGACCAGGGTG VSVQTRV 1

GAGCGCATCATGAGTTCTACG ERIMSST 1

TCGGTGGATCTCGAGGGGCTT SVDLEGL 1

CTCAGTATGCATCCCATCGTG LSMHPIV 1

ATTTACGAGATCACCAGGCTT IYEITRL 1

GTCCAGCATAGCAGGAAGTCT VQHSRKS 1

AGTGTGTCGGCTGTCGTGCAT SVSAVVH 1

GCCGTGAGGTATTTGCATTCT AVRYLHS 1

GGTCCGTGGGTCCGGGTTGAG GPWVRVE 1

CGGCCTTACCTCCGGGTGCGG RPYLRVR 2

ATGGGTGGGCCGGCCTTGCCT MGGPALP 1

CGGCGGCTTGGCGCTCTGTCC RRLGALS 1

GATGAGGCTGTCTCTTCGTGT DEAVSSC 1

ATCGACGACGCGATGTGTGTT IDDAMCV 1

GGTCGGTTCAAGTCCGGCGGT GRFKSGG 1

AGGGTTCGTAGCGATGCCCCG RVRSDAP 1

CGTGGGGTTCTCAGGATTCAT RGVLRIH 1

GTTCGCCACTGTCGGGGCGTG VRHCRGV 1

TTGTTCTCGACTTGTTGTGCG LFSTCCA 1

AATTGCCAGACGTTGGTGAGG NCQTLVR 1

AGCCCTCGGTACCGTGACGGG SPRYRDG 1

TGCATTCGGAATCACGTCCGT CIRNHVR 1

TACGGGCGGATCACGGAGCGC YGRITER 1

GTGCTCAGGGAGGTTCGCCAG VLREVRQ 1

CCGTGCAGCAGGCTGTGGCCG PCSRLWP 1

TATCAGGTTCGGTCGTTGCCT YQVRSLP 1

CGTCATGGGCCGGGTGGCCAT RHGPGGH 1

CGTGGTATTTGGTTGCGGGTT RGIWLRV 1

ACCGGGACTCACTTGGACTTT TGTHLDF 1

CTGCGTTACAGGAGCACCATT LRYRSTI 1

GCGGCTGCTGTGCACCGCGGT AAAVHRG 1

GTTGCGACTAATGGGGGGTGT VATNGGC 1

GGCGGCCTGTGCCGCACCGCG GGLCRTA 1

TCTCAGGCGGGCCGTTTGTGT SQAGRLC 2

GGGCGGCCTCGGGTCGCGCCT GRPRVAP 1

CGGCTGGTCAGCATGCCTGTG RLVSMPV 1

GCGCCCATTAGGGCGTTGGCT APIRALA 1

CTCCGCGGCTCTAACATTCCG LRGSNIP 1

GGCGTGGCTAGGCCCCTTGAT GVARPLD 1

GGCGAGCGCACGGTGCTCTTG GERTVLL 1

TGGGCTTCGGCGAATCAGTCT WASANQS 1

TGTGACGGTTCTTGGCCTGGT CDGSWPG 1

GGGGTGCCCGCGGCGAGGACT GVPAART 1

TCGGGTACGTGTTTCAGCCAC SGTCFSH 2

GAGTGCGCGTTCTTGGCTACG ECAFLAT 1

AGGTCGGGGATCTACGGGGCG RSGIYGA 1

TGGCTTGAGACGGGTTTTGGG WLETGFG 1

TGTAACGTGATTTACGTTATT CNVIYVI 1

GTTTCTGGGAGGTTGATGCCG VSGRLMP 1

GTGAATACCTACGGGGGTAGG VNTYGGR 1

GTCGATCTGAGGGTCGTCGGC VDLRVVG 1

GGCTCTGCTGGTGCTCAGCGG GSAGAQR 1

AGTAAGTGGGTCTTCGAGCTG SKWVFEL 1

AGGCGTTTGCGCGCTTATCTT RRLRAYL 1

CCGTTGAGGCCGCCTGGGTGC PLRPPGC 1

GGCCGCGGGGTGTCGGCGGCT GRGVSAA 1

TGTGTTATGGGTTGCAGGAGG CVMGCRR 1

GTTCATATGCTTAGTACTCGG VHMLSTR 1

CGGGTTGCTACTAGGGACGCT RVATRDA 1

GACGGGTATTCCGACCTTTTT DGYSDLF 1

TTTATGCAGGCGCTGGGGCTC FMQALGL 1

GCTATCGTTCTTTATAAGTGG AIVLYKW 1

GGTCGCGAGACGGGCCTGCGT GRETGLR 1

GGCACGACCAATTGCAGTTAT GTTNCSY 1

ACTTGTTGCGCTTCCTCCCCG TCCASSP 1

CTGGTTCAGCTCGAGTCTGGT LVQLESG 1

TATTCGGGCTACTGCGTTGGT YSGYCVG 1

TGGCGCGGCTCGGGTTTGCGT WRGSGLR 1

AATGACAGCCTGGGGGCTCGT NDSLGAR 1

GCGTACGGTGGGTTGGATTAC AYGGLDY 1

TACGCGCGTGTGCGTGTGGTT YARVRVV 1

ATTGTGAGCTGGATGCGCGGT IVSWMRG 1

TGGAGGTCTCGTGGTCATGGC WRSRGHG 1

GGGGTGGTTAGCTCCTACGTG GVVSSYV 1

CGTGGGGTGACTTGCTGGCTT RGVTCWL 1

TTCGAGCGGTCCAGCCGTGTT FERSSRV 1

TGCGGGTGTGGCTTGCGCCGG CGCGLRR 1

TCGGACGCCTCTTCGGTGGAT SDASSVD 1

GTTGTGTTCGAGTGGTCTCAT VVFEWSH 1

GATCCTGGGCACAGCGCGATT DPGHSAI 1

GCTTTCTGTACTGGGTGCCCC AFCTGCP 1

ATTCTCGACCTTTCCGCCGCG ILDLSAA 1

CATCACCGCTGGTGGCATGCT HHRWWHA 2

GTCCTTATTATTTGGGGGGAG VLIIWGE 3

AAGCCGCGCTGGAGTTCGCAG KPRWSSQ 1

GTGAGTTGTAACTCGACGCTT VSCNSTL 1

GCCGGCTACGGCGGGGACTGG AGYGGDW 1

GCGACTCGCTGCATCGTTCTG ATRCIVL 1

AGGATTACCGTGAGCCACGGT RITVSHG 1

TTGCGTGTCAGTCGGACGCGT LRVSRTR 1

CTGGGGAGGGCCAGCCGTCCT LGRASRP 1

CACAGGCCTTCGGGCTGTGTG HRPSGCV 1

GCTTCGCTTAGGGCGGTGCGG ASLRAVR 1

CTCACGCGCAGGCATTTGGCT LTRRHLA 2

CCGGCGCCCCTTCGGGGGAGT PAPLRGS 1

CGTGGCGAGAGCTATCTGGCG RGESYLA 1

TGCAGGCCCTATTCGCGTCCG CRPYSRP 1

CACCCTGGCGCCAGGTGTTGG HPGARCW 1

CGCGATTCGTGCGCCTCCCCG RDSCASP 1

CTTTGGATTGCTGTCGGGGCT LWIAVGA 1

TACCGGTTCGGTATGGACCCG YRFGMDP 1

GGCGTGAGTTCCCGCGCTCCG GVSSRAP 1

CTGAAGCTGGGGGAGTGTCCG LKLGECP 1

TGGTGCATGAAGTGTGATGGG WCMKCDG 1

GAGTGTGAGGTTCGCACCGAG ECEVRTE 1

TTCGACCGGGTTTGCTGCTCT FDRVCCS 1

GCGGGTTACGTGGGCGATAGT AGYVGDS 1

CTGGGCTCTTCGGCCAGCGTC LGSSASV 1

CTGTTTGCCGCGTTTGGCAGC LFAAFGS 2

CTGCCGGGTTCTCTTAGGCGG LPGSLRR 1

AGTTTTTACGTTTGGTGCACT SFYVWCT 1

TTTTGGAGCTTGTGCAGCTCT FWSLCSS 1

CCGTCGGCGTGGGTCGGCGTG PSAWVGV 1

GTGAATGGCGCGGTCAGTAGG VNGAVSR 1

TCGGCTCAGTTGTACGTCCTC SAQLYVL 1

CCGAGGGTGGCTAGTCACTCG PRVASHS 2

CATAGCATGCTGTTTGACCCT HSMLFDP 1

CAGCAGCTTCCGTGGAAGCGG QQLPWKR 1

GGGAGTCGGCTTGTGAGCGTT GSRLVSV 1

AACGCTGATGGCTGTAGTGTG NADGCSV 1

GGTGGGTCGGTCGGCGACGGT GGSVGDG 2

CGTGGGATGAGCTGCTCCGCG RGMSCSA 2

TGTGCCACTAGGTGCCTGGCT CATRCLA 2

AATCGCGAGGTGTTGGTGCTT NREVLVL 1

TTTGCTAAGGTGGGTCGGAGT FAKVGRS 1

GTGGGGGTCACTTCGCATAGC VGVTSHS 1

GGGGCGTGGTTGGGCGTTGGG GAWLGVG 1

GTTGGCCTGGTCTTGGTGGGT VGLVLVG 1

TTGAGGTGTCGCGGCCAGGGG LRCRGQG 1

TTCGTCGAGAAGGTTAAGGCT FVEKVKA 1

TTGGTGGTGGACTTCGCCGCG LVVDFAA 1

GGGGAGCCCTTCCGTACTTTC GEPFRTF 1

CTCTGTCCTAGGGAGTTTCGC LCPREFR 1

TGGCCGGTCCGGGTCGCTCTT WPVRVAL 1

CGGGGGAGGATCTCTTATGAC RGRISYD 1

ATGCCGGGTGATGCGCGGTGG MPGDARW 1

TGTGCTGTTATCCTCAAGAGT CAVILKS 1

GGCGTCGGGTCCCTGCTTTAT GVGSLLY 1

ACTAGGACGTCTTGCTACAGT TRTSCYS 1

AACCGTCCGGGTAGGTCTCTG NRPGRSL 1

ACTGTCCCCTCCGATGCTCTT TVPSDAL 1

TGGCGGGACACTTGTCATCGC WRDTCHR 1

TGGAAGGCCTACGGTCACGCT WKAYGHA 1

CAGGGCGTCCGCGTTGGCCCT QGVRVGP 1

ACTAGGGATCAGTTCGGTGGT TRDQFGG 1

CTCTGGGGTCGTGAGGGCCTG LWGREGL 1

CATGACCACAACTCTCGGCGT HDHNSRR 1

ATGTATTACGTCGTCGGGTTT MYYVVGF 1

AGCGCGAGCTCGGGTTTGGGC SASSGLG 1

GACCGGCTGGAGGCGCGTAAT DRLEARN 1

CGTCCTAGGCTCTCTGCGCTT RPRLSAL 1

CGCCGCTCTAGGGAGCCTGCG RRSREPA 1

GTGCGCAGCGTGGCTGAGCGT VRSVAER 1

AACAGGCGCACTGGTGTCTCC NRRTGVS 1

GCGGGGCTCGGGTTCCGGTGT AGLGFRC 1

GTCCTGGTTCAGGTCAATCCG VLVQVNP 1

TCGAGGCCCTATGGCGAGTGC SRPYGEC 1

CTGCTCGGGGTGAAGGGCCGT LLGVKGR 1

GCCTTGCTCAGCGGCTTTTCT ALLSGFS 1

GTTTCGGTCACCAAGGCTATT VSVTKAI 1

TGCGTCGGCTCGACCTTGCAC CVGSTLH 2

GGGAGCGCGGATGGGCGGCGG GSADGRR 1

TCTATGCTGTCGTGGCAGTGT SMLSWQC 1

GTGGTGTGGGTCTGGTCTAGT VVWVWSS 1

CGTGTGGCGTTTCGGGTGTGT RVAFRVC 1

GCGCGTCGCCCTAGCAGGTCG ARRPSRS 1

AACGGCTTGGAGAACCTGATC NGLENLI 1

ACCGAGTGCTGTTTCGTTTCT TECCFVS 1

GCGAGTCTGATGCTGTACCAT ASLMLYH 2

ATGCAGACGAGCAATTGGGCT MQTSNWA 1

TTGGTCCGGCTCAACGCCCGT LVRLNAR 1

TGTCTCCGTATGGCGCTGGGG CLRMALG 1

CACTCGTTCAGCGAGTCCTCG HSFSESS 1

TGCTCGACGGTGAGGAGGGGG CSTVRRG 1

CATCCGTGTAGGCGCGCGGGG HPCRRAG 1

TGCTGTGTCTCCGGGCCGCGT CCVSGPR 1

TTGTGTCCCCGGGCCCTCCGG LCPRALR 1

GTTCACCGCTGGGCTGGGCTC VHRWAGL 3

CTGCTGCGCCGCCCGGTTATT LLRRPVI 1

TTCGAGTTGTCCGGCCGTAAT FELSGRN 1

GGGTGCTACAATTGGGGCTCC GCYNWGS 1

TGTCCGCGTTATGTGGTGCGG CPRYVVR 1

CTGCGGTCCGGCCAGGGTTGG LRSGQGW 1

CTGGGGCTCAGCAATGGTGAG LGLSNGE 1

CTGGTCGCGTCGGTTTGGACG LVASVWT 1

ATCGTGCTGGGGACCTGTTTG IVLGTCL 1

AATTGTACGCGCCAGAGGGCG NCTRQRA 1

GGCGCTCGCGTGCGGAAGATT GARVRKI 1

ACTAAGCCCAGGCTTGGTTGG TKPRLGW 1

ACTGCTGCCTCGTTTGAGTGC TAASFEC 1

CGTTTGCAGATCCGGTGTAAG RLQIRCK 1

GACTCGCGGCGTTTGTCCCTT DSRRLSL 1

ACTCTTGCGTCGCGGTCTTTT TLASRSF 1

GGTTGCACCTACTGCTCCCTG GCTYCSL 1

TCTCAGGGTTTTGGCGCGGGC SQGFGAG 1

AGGCTTCCGCTTTGCTCGCCT RLPLCSP 1

ACTTCTGGCAGTCTTAGCCCT TSGSLSP 1

GGCCGGCACTCGGGGCGGTTG GRHSGRL 1

TGCGAGTTCTTGCTCGCGGGT CEFLLAG 1

GAGTCGTTTGGTTGGAGTGCT ESFGWSA 1

TGGTCGGGTCGTATTAGCCTT WSGRISL 1

TGGTTGAGGCGGTCCGGTATT WLRRSGI 1

CTTGTTACGGACCGGCACGAT LVTDRHD 1

GAGCGGGGCCTGCTGTGTACT ERGLLCT 1

GAGTCGCTGAGGGGGCATCAG ESLRGHQ 1

CGTTCGCGCGCGTTTTGTCGG RSRAFCR 2

GGCGGTCTTAGCAGCGCCGGT GGLSSAG 1

CACGCCATCAGCGGTTGTCCT HAISGCP 1

GGCGGCGTGATTACGCTTGGC GGVITLG 1

TTTAGGTCCAGGCTGGCGACT FRSRLAT 1

TCCCTTCGCTGGGTCACTTCG SLRWVTS 2

TTTTTCTTTACTAATCTTATT FFFTNLI 1

ATTCCTTGCTCTGGCACTCCT IPCSGTP 2

GAGACGTTGAGGGTTTTGTGC ETLRVLC 1

ATGAGCGCCTGTACCGCGAGT MSACTAS 2

GCGCGTTGTACGGAGTCGCAG ARCTESQ 1

TGCCAGGTTCACATGTTGCTG CQVHMLL 1

TGCGGTGGGGTGCCGACTGCG CGGVPTA 1

TCCCCTCTTTGCGGCATCGAT SPLCGID 2

CGTAACAAGGGGGCGCACCCT RNKGAHP 1

TTGAATGGTGGCGACCTGGGG LNGGDLG 1

GCCGGGCGCAGTCGCTCGCAT AGRSRSH 1

AATGTCTGGTCTGCGATCCTC NVWSAIL 1

GTTGCGATCCCTTTTCGGGTG VAIPFRV 1

TCTTGGGATTCGTTCTATACG SWDSFYT 1

TGGCGGGCCACCCGCATTATG WRATRIM 1

TTGGGCCTTTGGACGTGGCGG LGLWTWR 1

GCTTTGTTTATTGAGGTGTGT ALFIEVC 1

GGTTACCCGGCCGTGGTGGGT GYPAVVG 1

GGTAGTCATTTGAGTCTCTCC GSHLSLS 1

CTGTGGAGGCCTAAGTCCTGG LWRPKSW 1

GGCCAGTCCAAGTGTATCTGC GQSKCIC 1

GGGCTCAGCAGCTTCGGTACT GLSSFGT 1

GTTCTTTTCATGGTTGGGTCG VLFMVGS 1

GGGGTGATTTGTTGGACGTTT GVICWTF 1

GACTTGCTGCGCCAGGGGAGT DLLRQGS 1

CATTGTGCGAGTGGGTTTTCT HCASGFS 3

GCGGCCCCTGGCCAGAATATT AAPGQNI 1

GACCGCCACGGTGACCTGGGT DRHGDLG 1

ATTTCGCCGAGGGCCGACCTG ISPRADL 1

GGTATTCAGCGGCGGTCGTTC GIQRRSF 1

TCGGCGGGCAAGGCCTGGAAC SAGKAWN 3

GCGGGCGCTAGGGTCTCCCTG AGARVSL 1

CTTACGTCGTTGCCGCGTCGT LTSLPRR 1

GTTGCGGCTTGTAGTTTTGGG VAACSFG 1

CGGAGTGGTTTGCTCGCGCTG RSGLLAL 1

CTGGTGCGGGGCGGCGATCCT LVRGGDP 1

CAGCGGGATGCGGGGAGGTCG QRDAGRS 1

GAGGGGGTCGGGTGGGTGCTT EGVGWVL 1

GGGGCTACGACGGTCTCGCTG GATTVSL 1

CATTACACGGTGCTGACGTTG HYTVLTL 1

CTTTATGACTGTAAGGCGCTC LYDCKAL 1

TCGGAGTCCCTGGAGTTCGGG SESLEFG 1

TTGGGCGCTCAGCTTTTGTCC LGAQLLS 1

TTCCCTCTTAGCGGCGTTGAT FPLSGVD 1

TCGTTGCGCAGTAGGTGTGAG SLRSRCE 1

CGCTACCGCTGGGATTGGCCG RYRWDWP 1

CTGGGCGGTCTCTGTTTCGTG LGGLCFV 1

CGTGTGGACGAGGTGAGTCGC RVDEVSR 1

TTCTGCGAGACCGACTCTTGT FCETDSC 2

CTCAACTCTGCCGACGCGCAG LNSADAQ 1

ATTCGCGACCGGATTGTTTGG IRDRIVW 1

AGGGTTGGGCTGCGGATCGCT RVGLRIA 1

ATTACGCGGACGCCTATCTCT ITRTPIS 1

TTGGTGAAGTCGGGGAATATG LVKSGNM 1

TGGCTCTCCGGCGCCGTCTGG WLSGAVW 2

GTCTGCCGGCTCGAGAGGCTT VCRLERL 1

GTGTGTTCTGTTGCGGGTCGC VCSVAGR 1

CACCCCTTTAGTAACGTGGGC HPFSNVG 1

AGCTCGGTGTTCCGGTGGTGC SSVFRWC 1

CGCAGGAGTTTCCGGGAGATT RRSFREI 1

TCTAGGTGCTGCGCTTATTAT SRCCAYY 1

GGTCTTCATCTGTTGGAGGAT GLHLLED 1

GATGGTCTCCGCAGGGGCACG DGLRRGT 1

GCGGACTCCACGGGGTTCCGG ADSTGFR 4

GTTTCCATGAGGAGGGGTTCG VSMRRGS 2

GGCACTCTCTGGTGGGGCTCT GTLWWGS 3

GGGCTCCGGAGTGTTCATCCG GLRSVHP 1

GGCGTTCTCAGCACGTTGGCT GVLSTLA 1

GTCGTCTCGGTTGTGGAGTTG VVSVVEL 1

CAGCAGTCGTATGGCCTGGCG QQSYGLA 1

CATTTGAGCGCGCGGGCGGCT HLSARAA 1

AAGTTTTCCCTTCACTTGCAG KFSLHLQ 1

GAGGTCTTGACGGCGGCTCCC EVLTAAP 1

CTGACGGTGATGGCCGGTGCG LTVMAGA 1

CCGTTTAGCAGCGGTTTGGCT PFSSGLA 1

GATAATGACGAGTCGAGGCAG DNDESRQ 1

GTGCACTGCGGTAGCGCCACG VHCGSAT 1

CGGCGTCTCTACGGCCTCCTT RRLYGLL 1

GGTAGCAATGCTGCGATGGCT GSNAAMA 1

TCTTGCGCGGTCTTGAGGTTG SCAVLRL 1

CGCCCCTGCTGGGTTCTCCTC RPCWVLL 1

TCGGAGGTCGGCGGCTCTGCG SEVGGSA 1

GCCAGGGATGTCGGTGTGCCG ARDVGVP 1

TATCGTTTGGGTCGGAGGTGT YRLGRRC 1

TTCCAGAGGCTTCGTCTTGCT FQRLRLA 1

TATTACTACAGGCAGAGGTAT YYYRQRY 1

CTGCTGAAGTGCGGTGTTGGC LLKCGVG 2

CCTTGTGGGACTACGATGTCG PCGTTMS 1

TCCGGTGTGGGGCGGACGTGG SGVGRTW 1

AAGCGCTCGGCGCTCGCGTTT KRSALAF 4

CTGCGGGGTATGTCGGCGGCT LRGMSAA 1

AATGCCGGTAGCTTGTGCGCT NAGSLCA 1

GGGCGCCGTTGCGCTCGCTTT GRRCARF 1

GTGCAGTGGGAGTGGAGGCCT VQWEWRP 1

CGCGGTGCGTTCGGTTCGTTT RGAFGSF 1

CAGCTGCGTACGCGGTGGCTT QLRTRWL 1

ACGAGGTCGAGGTTCGGCAGT TRSRFGS 1

TACTATGGTTTGCGGTCGGCT YYGLRSA 1

GGGTTGTTGTGCTACTGGGTT GLLCYWV 1

ACGTTTCTCCTGGTTCGTTCG TFLLVRS 1

TGTCTGCCGAGGCTCTCGGTG CLPRLSV 1

GTGCTGTATCATTGGGAGATT VLYHWEI 1

GGGCGGGTGGCCAATCGGCAT GRVANRH 1

TCGGAGGTTTGTTTGGTGGCG SEVCLVA 1

GAGCAGTGGCTGGACAGCAGC EQWLDSS 1

CTCCGCGCTGTTTCGGGCGCG LRAVSGA 1

CCTCGTGGCGCTGGTTGTTCG PRGAGCS 1

AGGTATGGGTCGGGCGCCGCT RYGSGAA 1

TACCGCAGGAGCGCGCCTGTT YRRSAPV 1

CGGGTGTTCGGTCGGCTGCCG RVFGRLP 1

GGTTGCGTGCGCCTGCTCCGG GCVRLLR 1

GCTTTGAGCAGGCCGATCAGT ALSRPIS 1

GACGCGCTGCCGTGGTCCCCT DALPWSP 1

TACTGCGAGGGCCACAGCGTT YCEGHSV 1

GGCGTTGAGGGCGGGAGTTGT GVEGGSC 1

AGGTACGTGTATCGGTCGAGT RYVYRSS 2

CGCACGCACACGCGGTACGAT RTHTRYD 1

AGTGATCACGTTAACGTTACG SDHVNVT 1

GTGGTTTCCAGTGGCGAGCGC VVSSGER 1

GAGGAGCTCTCGTCTCGTCGG EELSSRR 1

TATTTGCGTCGGGGGAGTGAG YLRRGSE 1

GGCCCGGGCGGGTGGCGGCTT GPGGWRL 1

TGCATGTTCGCGACCGCGAAT CMFATAN 1

TGGGTCACGCTTCGTAGCGGG WVTLRSG 4

ATTGGCCGTAGTTGCTGCGGG IGRSCCG 1

CCCGGGCGGTTTGTTGGCGGG PGRFVGG 1

ATGTTGGGTGATACCTGTGGT MLGDTCG 1

CATGTGAATTTTGACTGCTCT HVNFDCS 1

AGCGGTCCTCATTCGGGGAGT SGPHSGS 1

ATGCAGACCTCGCACGCGGCG MQTSHAA 1

GGCGGGGGCTTCCATTCGGTT GGGFHSV 1

AACGGGAGGGGGGGCGAGCGT NGRGGER 1

TGCTGTGAGGCGAACGGGGTC CCEANGV 1

CTCCTCGTCGTTATGTTGCGT LLVVMLR 4

GCTCGCTGGATCACGATTTTC ARWITIF 2

GCTTCGGCGGATAGGGAGCTT ASADREL 2

GATCCGTTTTGCAGTTCGAGC DPFCSSS 1

ACCTCGTGGAGGTGGTGGAGT TSWRWWS 1

CTTGTGGAGGAGGAGACTCTT LVEEETL 1

GCGAGCGAGGGCAGTGTTAAT ASEGSVN 1

CGTCGCCGGGCCCTCTGGGAG RRRALWE 1

TCGCGTCGCAGCAGGTGCGAG SRRSRCE 1

CGTGGTCTGCTTGTGATCGCG RGLLVIA 1

GCTTGGGGTCTCCTTAGTACC AWGLLST 1

TACCTGCACGTCGAGCTCCAC YLHVELH 1

GCGGGGGACTATTCCTATGGT AGDYSYG 1

AGGACTCGGGTTTTGGTTCCT RTRVLVP 1

CACGTCCATTGGAGGATTGTC HVHWRIV 1

AATGGCTACCTGAGGCAGTGC NGYLRQC 1

CACGGGGTTATGCGCGGTTTT HGVMRGF 1

GTGGAGTGGAGGACGCTGATT VEWRTLI 1

GGTCATCGCCCCTGGATCTGT GHRPWIC 1

CATGTGTTCCGCGCGCTCCGG HVFRALR 1

CTTACGTACATGCGCTGTTAC LTYMRCY 1

CTGCGCGGCACTGGCGGGACT LRGTGGT 1

GCGAGGCTCCGGTTGTATTCT ARLRLYS 2

ACGGGGCTCACGGGGTGTATG TGLTGCM 1

TGTGTGAGGCCGGTGTACGTG CVRPVYV 2

AGGAGCGGTTCGTGTGTGCGT RSGSCVR 1

ATGTTGAAGTCGGGTGCCGGG MLKSGAG 1

TGGTATCGTTTTTACCAGCCG WYRFYQP 1

CGCCGGGGCGACAGGTATCTT RRGDRYL 1

GTCGCCCCTAGGAGTTCCGTG VAPRSSV 1

GTTGCTCAGTTTACGGGTCCT VAQFTGP 1

CGGACTCTCAACTTGAGCGAT RTLNLSD 1

CGTAACGGCACTCGTACGAAG RNGTRTK 1

GTCAGGATGCCGTCTCATGCT VRMPSHA 1

GTCGGGCGGTTTTGGGAGCGT VGRFWER 1

CGCGTGGAGGTCGGTGGTGCT RVEVGGA 2

AATTGCACCGCGTCCAAGAAC NCTASKN 1

CGTGACGGCATGAGCTACTGT RDGMSYC 1

GTCCAGTTGGGGCGGCTCTAT VQLGRLY 1

CTGGGCATGGTCTCGGTTGGG LGMVSVG 1

GGCGAGATTAGCTCTCTTCGT GEISSLR 1

GGCGGCTGTGGTTGTGGCCCT GGCGCGP 1

AAGGAGCGGCTGCGCTGGAAT KERLRWN 1

GGTAGCGTCAGGCGGTTCCGT GSVRRFR 1

CTGTGTTTCTCTCTGTTGGGT LCFSLLG 1

GTTAGGAGTATGCCGTATAGG VRSMPYR 1

CTGCACCGGACCGGTACTGGT LHRTGTG 1

GATGGGGCCTGGCGGTTCGGT DGAWRFG 1

TATTCGTCCTGTAGGCGGCTC YSSCRRL 1

GCTATTTTCCGCGACAAGCGG AIFRDKR 1

TGGTGCAATTCTCAGAGGCCG WCNSQRP 1

GGGGGTCTTCGGCGGCGCCTC GGLRRRL 1

CGTTCCCTCATTGTCGTTGTT RSLIVVV 1

AAGCTCGCCGAGTGCAGCTCT KLAECSS 1

AATCAGTTGGCGGCCAGGCCT NQLAARP 1

TTGGCCGGGCTTTGGATCCCG LAGLWIP 1

GTTGACGGCCGCACCCGGAAG VDGRTRK 1

AGTTACATCTCCGCGTCTGGC SYISASG 1

AGCCGCGTCCGTGTTGACTCG SRVRVDS 1

GTTCCGCGGTGTGTGGCTCGT VPRCVAR 1

GGCTGTTTTGGTGGGACCCGG GCFGGTR 1

GGCGATTACTACTTCGGCACC GDYYFGT 1

AGGTGGGTGTATAGGTGGTCC RWVYRWS 2

GTGTACCATTTGGGCCGGCCG VYHLGRP 1

CGCCGGGTGGTTCTTTGCTAC RRVVLCY 1

AGTTTGGACGTCAGCGGGACG SLDVSGT 1

TACGCTCAGAGGGAGCTGCCC YAQRELP 1

CGCGTCGCGTCGTCCCAGGTT RVASSQV 1

GGTAGTCATATGCTCCCGTGG GSHMLPW 1

AACCCGGGTGCTGGCAGCGCT NPGAGSA 1

GGCTTTAGTGACAGCAGGTCT GFSDSRS 1

AATTGTTACTGGGGTGGGTCT NCYWGGS 1

ATCGTCAGTTCTCGGCAGCTT IVSSRQL 2

CGGTATCAGAACGCGGGTGCT RYQNAGA 1

GCCGTTGATCTGGCGATCTGG AVDLAIW 1

GGCTGTTACCAGCCTTTCCTG GCYQPFL 1

AAGTTCGGCAGGCACGTCCAT KFGRHVH 1

CACGTGTGGGGGTGCTTCTGT HVWGCFC 1

TGGCTTAGTTTGAGCGTTCGC WLSLSVR 1

GGCCATCTTTCGATGTTGGCC GHLSMLA 2

AACCGTTCGTCGTTCTCTAAG NRSSFSK 1

GGTCCTAGTGGCTGTCTGCAT GPSGCLH 1

CCTGGCTTTTTGTTCGTCATT PGFLFVI 1

AGGCTGGAGGCGCTGGGCTCG RLEALGS 1

CAGTTTATCTGTGGTGAGGTT QFICGEV 1

GTCGCTTGGGGCTGGTGGACT VAWGWWT 1

ATTGCCTATCGTAGCAACCGT IAYRSNR 1

GGGCGGACTTGCAGCTTTCGG GRTCSFR 1

GACTTTCACAGGCGCTGGGAG DFHRRWE 1

GTCCTCTATGTCGTCGCGGGG VLYVVAG 1

TGCTATGACCTGCTCTTGGCG CYDLLLA 1

CTCTTCTATTCCTCCGAGGAT LFYSSED 1

TCCTCGGCGTGGATCGTGGAG SSAWIVE 1

TTTGGCCATCGCAGTGTGTCT FGHRSVS 1

GCGATGCTCGTGACGCCTCGT AMLVTPR 1

GGGCGGAGGGCGGGCGGTTCG GRRAGGS 1

CGCAGCGTCAGGATGTGCAGC RSVRMCS 1

GCGGGGCCCTCCGGCCGTCTT AGPSGRL 1

GGGCTGCGCACCGTCAGCGGT GLRTVSG 1

AGGATCTGTGGTATGAAGATG RICGMKM 1

CAGCATATCAAGTCCGGGTCC QHIKSGS 1

TTTGTGAGTTGTCGGGAGCGG FVSCRER 2

GTGTGGCGGAGGTGCACCTCC VWRRCTS 1

TCCTACAGCCTGTCGTGGCTT SYSLSWL 1

TGTGGGGCGTATTTTTGCCCG CGAYFCP 1

AGGCGTATTTGGCACTCGGTT RRIWHSV 1

TGGGACACGACGAGGTTGAAT WDTTRLN 1

GTCAGGTGGGTTCAGCTCCCT VRWVQLP 1

TGCCAGGACCCTGGCCGTCGG CQDPGRR 1

AGCCTGGCTGGGAGGCTCAGG SLAGRLR 1

GACCTTGACGGTGCCGATCCG DLDGADP 1

TATGTTCTTTGGGTCGGTCCG YVLWVGP 1

TACGGCTGGTTTGTGAGGCGG YGWFVRR 1

AGGTGCGGGAAGCCGTATCGT RCGKPYR 1

GTCTACGGGACCGTCCTCAGG VYGTVLR 1

GATCGTGGCAAGCTTCCCAGC DRGKLPS 1

CGGTCGTATACTGCGAAGTGG RSYTAKW 1

TCTGGGTGGAACCTGGAGGTT SGWNLEV 1

CGGCCCGGGAAGACCGGGACG RPGKTGT 1

GGTAGGGAGCGGTGGGGTGTG GRERWGV 1

GGGCACATCACTACCCATGGG GHITTHG 1

GGTATTGTGCGCTGGCCTAGT GIVRWPS 1

AAGGGGGAGAGGGTCTGGCAG KGERVWQ 1

GTCTCGGATATCAGTTCGGGG VSDISSG 1

GTCCGGCTCAGCGTCGGTCAT VRLSVGH 1

GGCTCGCGTCGTTGCGCTTGT GSRRCAC 1

ATTGTGGGGGGTGCTGTGACT IVGGAVT 1

GACTGCCCTCTCGCGTGCGGG DCPLACG 1

TTGCTGCCGTCGGCTACTATT LLPSATI 1

TACTCCGTGTACATCAGCTGC YSVYISC 1

GCCGTGGCTATGAACAACTGT AVAMNNC 1

AACGGTTCCACGAGGTATATT NGSTRYI 1

TTTTCGGGGACCTGGGTCGCT FSGTWVA 1

TTCGTCGGTCCGGCTAGGGTT FVGPARV 1

TGTTTTAAGGAGTTTTATCGG CFKEFYR 1

GTGGAGGGCAGGTTTATTCGT VEGRFIR 1

GTTAATGTTCTCTGGCCGCCG VNVLWPP 1

AAGGCGGGCTTGCCGCGTGTT KAGLPRV 1

CCGTGCTGTGCGATTAGGGAG PCCAIRE 1

GGCGGGTGTCGGGAGTGTGGG GGCRECG 1

CTGCGGCGTGTCCGTAATAGC LRRVRNS 1

CAGCACCTCGTGTGGGTTCTT QHLVWVL 1

AGGGTCAGGTCGGTGGAGGAT RVRSVED 1

ATGCACGGCAATCAGTTCGAT MHGNQFD 1

GCGCATTTGCTGCGCTCTACT AHLLRST 1

TGCGGCTGCACTAGCGGGCGG CGCTSGR 1

AGGAGCATGCGGGAGTGTCGT RSMRECR 1

GAGCAGCCTAGCATCGCTTAT EQPSIAY 2

GGCTGGGAGAGCAATTCTCTG GWESNSL 1

ATGTGGTTGATCGCGTCGGTT MWLIASV 2

AACGTTAAGATGGAGGCCATC NVKMEAI 1

ATTAGCGCGAAGGTTGTGCGT ISAKVVR 1

GTTCGCGGCACGTTTAATCTG VRGTFNL 1

GACCTTGAGGGGCGCGGTCTG DLEGRGL 1

TCCATTGGGAGGGTCAAGCGG SIGRVKR 1

GCGGGGCGGATTTCTTTGGCG AGRISLA 1

AACCGTGATTGCGCGAAGTGG NRDCAKW 1

ATGCTGCAGATGTGGCGGCGT MLQMWRR 1

GGTGCGGAGCAGGAGGGGTGG GAEQEGW 3

TCCTTGTCTACCGGGCCGACT SLSTGPT 1

AACGCTGTCGTGTCTGGTAGT NAVVSGS 1

CACAGCAGTGAGGGCAGGAGT HSSEGRS 1

AGCCGGGCGGCTCTGGTGTCT SRAALVS 1

CCCGCCGCGAGTCTGACGCTT PAASLTL 1

GGTGTGCGTGGCGGTTTTAAG GVRGGFK 2

CGCGTGCCTCCGGTCATGCCT RVPPVMP 1

TCCCTGCGCGCTACTTCGGCG SLRATSA 1

AGTAGTCTTGATTCTCGGCGG SSLDSRR 1

AGCTACCGCGTGTCCGGGCCT SYRVSGP 1

GTGGTTGCCGGTTTGAATATT VVAGLNI 1

GGCGTTCAGTGGCGCCGGGCG GVQWRRA 1

TGGGGTGGCTTCTCGGTGGGT WGGFSVG 1

ATTCGGTCGTGGGGCACTGAT IRSWGTD 1

GGTACGGGGTGGTTCTGGGCT GTGWFWA 1

TGTGGCTTCCGTTGTGGGTCT CGFRCGS 1

TTCTGGAGCGTCGTTGAGCCG FWSVVEP 1

AGGGTGCCCTGGGAGCCTCGT RVPWEPR 1

ATGATGACGTGGCTCGCGCCT MMTWLAP 1

TGCAGTTCTGTCGATGACAGT CSSVDDS 1

GGCTGGTTTTTCTACCGGAAT GWFFYRN 1

TTGACTCGTGCCGTCACCGGT LTRAVTG 1

TCCCTGACCCTTTTGGCCGGG SLTLLAG 1

ATGCAGGGCGGTGTCGCTCGG MQGGVAR 1

CTTGGGTTGCAGAGCTATTTT LGLQSYF 1

CGGGCTAAGTTTTGTGAGCTT RAKFCEL 1

GGGCTGGATGCTCGTTGCCCT GLDARCP 1

ACCAAGGTCCGGAGCTCGCTT TKVRSSL 1

GACCGGGTGCGTGGGGTGCCT DRVRGVP 1

ATGATTGGTATGGTTTGCGGT MIGMVCG 2

AGCTCGTGTGTCGTGAGCTGT SSCVVSC 2

GTGTTCGAGATGGCGCTCCCT VFEMALP 1

TCGCCGTGTCGGTGTCGTTGT SPCRCRC 1

GGTGGCCTTGCGTCCAGTCTT GGLASSL 1

CAGCTCCACCTGCAGTATACG QLHLQYT 1

AGCCATGGTATGCATCAGCTT SHGMHQL 1

ACTACTTGTGGGCCCGCGAGT TTCGPAS 1

TGTAGGCGGTGCTCGGACACT CRRCSDT 1

GGTAGGTGGCGTGCGGGTGTC GRWRAGV 1

GTCATGCAGCGGGCTAGGCTT VMQRARL 1

ATTGGGTGTAGCGGGTGTAGT IGCSGCS 1

CGCGAGAGTAGTGAGATCCCT RESSEIP 1

AATATCCGTAATCGCATCGGG NIRNRIG 1

CCTCCTTTCGTGGAGGTCGAT PPFVEVD 1

CGCGTGAGTAGTTCGGATTGC RVSSSDC 1

TTTGCGTGTTCGGGTCAGACT FACSGQT 1

TTTCTTGGTGCGAACTTCCGT FLGANFR 1

GCGGTCTTGAAGGTTAGGCTT AVLKVRL 1

GCGGCGCTTGTCTGCGAGATT AALVCEI 1

TGTATTATGTCGTACGCCGGG CIMSYAG 1

CTTAGCTGTAGGCAGGTTGGT LSCRQVG 1

CCGGTGGGGATTGCGATCGGT PVGIAIG 1

GCGTGCACCACGGCCGCGGGC ACTTAAG 1

ACGGGGGTGAGCTGTCTTTGG TGVSCLW 1

GCTCCGATTCGGAGGCCGGCT APIRRPA 1

AAGCGGGGTTGTACCAAGCGG KRGCTKR 1

TGTGCCTTGCGTATTCGTGGG CALRIRG 1

TCTGTCAGGGAGTTTGTCCAT SVREFVH 1

GCTGTCTCGAGGGCTCCGTCG AVSRAPS 1

ACGGTGGACGGTTGGTGCTGC TVDGWCC 1

CGTGTGCCCAGCTTTGGGTTG RVPSFGL 1

CGGTTGCTGGCTCATCAGACC RLLAHQT 1

GAGTGGGGCGGTTGCACGAAT EWGGCTN 1

TGTCCTGCTCGTTCCAGGCGT CPARSRR 2

CTGCGGAGGGCTCGGCATAAT LRRARHN 1

GTCCATGGCAGCGTGTGTCTC VHGSVCL 1

GGTCGGCGGACCACTGAGAGT GRRTTES 1

TCCGGGCCGGATGGCACGCGT SGPDGTR 1

CGGCCGGCGCTGGACACCATT RPALDTI 2

CGTGATCGGCTGCTGTTCCGT RDRLLFR 2

GGCGCGGTGGCGTTCATGTAT GAVAFMY 1

GGGCTGGCCGATTGGCGGGTT GLADWRV 1

TCGTCGGCGCTGCATCAGGAT SSALHQD 1

TCTGGGCGGGTGGGTGGCTTT SGRVGGF 1

AGGGACAGGGCGCATCCTCCT RDRAHPP 1

CGGTGGTTTAGCAGGGGTGCT RWFSRGA 1

GTTAGTCCCCCTCTGGGTTCG VSPPLGS 1

AAGGTTGCTGCCCATCGCGCG KVAAHRA 1

ACGGTGGCTATGGGCGGTCAG TVAMGGQ 1

AGCTTCGCTGCCCGGGCCACT SFAARAT 1

GTTGACATCTGTCGTTTTTCG VDICRFS 1

ATTCGCTATTTGCTGATCCCG IRYLLIP 1

GATATTTATTTGGTGGCCAGG DIYLVAR 1

AACGGTTGTGCGCTCGGGGAT NGCALGD 1

GTTTGGCCGGGCTTCGCTTCC VWPGFAS 1

AACTTTGTCACGCTTGCTATT NFVTLAI 1

GGGAGTGGGGGCTGCGCCGGG GSGGCAG 1

GCCGGGATGACGTCTTTCCCT AGMTSFP 1

TCGGTGGTTCGGGACGAGAGG SVVRDER 1

GCGCGGAGTACCGTGCTGCAG ARSTVLQ 1

AGCTCTTTCGCGCCGTCCGCG SSFAPSA 1

GCTTTTGCTCGGCGTTGCACT AFARRCT 1

AGTCTGTCTGGCTTCGTTTCT SLSGFVS 1

GGGGTGGGCCCTCACTGTCTT GVGPHCL 1

CATGAGCGTGGTGGCACTGAT HERGGTD 1

GGTGGTGCCGGGCGCGATTCG GGAGRDS 2

CTGCGCTGGATTCAGTCCGCG LRWIQSA 1

TCTCTGGTCTGGCACAGCAAT SLVWHSN 1

TCGGCGCGGTTGGTCGCCGGG SARLVAG 1

TCGTCCATGACTTACTGTGTT SSMTYCV 1

GGGTTTGGGGCGGTCTACACG GFGAVYT 1

ATGCATATCTCCGCGGGGAAT MHISAGN 1

TTCCGGGTCGCGCGCGGCTGG FRVARGW 2

GGGCAGAAGCCCGAGGACTCT GQKPEDS 1

TACGGTGCGGTCGTCACCGCT YGAVVTA 1

TCTGCGTGTGTCTTCCGTCTT SACVFRL 1

TTCGGTCTTTTGGATAGTGGG FGLLDSG 1

TGCCTGGTGATCTGCATCAGC CLVICIS 1

GTGGCGGTCGTGCAGACGGGT VAVVQTG 2

AATCACGCCAGGAGCTACTTT NHARSYF 1

GGCAATGTGGAGCTGGTGAAG GNVELVK 1

TCTGCTGCTGCCTATGGGTGG SAAAYGW 1

GTGGGCAGTGCGGACCGGCTT VGSADRL 1

CGGACTTCTTTCGTTCGGGCG RTSFVRA 1

GGGTTTAAGGGTCACCAGAGC GFKGHQS 1

GGCGGCTTGAAGGTGAATGGC GGLKVNG 1

TGTTGCGACAGTAGTTTCCCT CCDSSFP 1

GGGCTGCTGATTGCGTATAAT GLLIAYN 1

AGGCCCGTGCACCGGTACAGC RPVHRYS 2

CGTAGGTGGTCGATCGTGAGG RRWSIVR 1

GCGCCGGCCTGGGCTCGCTAC APAWARY 1

CTTTCGCTCGATGGTTACTGT LSLDGYC 1

TCGTCCCCTGCGCGGCTTCGG SSPARLR 1

GGGGCCGGGCGTGCCCTGGCG GAGRALA 1

GATGTGAGCCAGGTGTTCGCT DVSQVFA 1

GACCGTACGAGTCTGGTGGTG DRTSLVV 1

CATTGGGAGGCGGGTGACAGG HWEAGDR 1

GACAGGGGTCCGGGGTACCAG DRGPGYQ 1

CCGGCGCGGTGTCGGCCGGGT PARCRPG 1

GGCGCGACGGTCTCTCCGTGC GATVSPC 1

GGTGCGCCCGGCTCGGAGCTT GAPGSEL 1

GAGCGCCACGGCAAGGCCAAT ERHGKAN 1

GGGAGGTTGTCGTGGCTCTTT GRLSWLF 2

TTTGAGTGGCTCGGGCCGACG FEWLGPT 2

TGCTGCATTGATGTGACCAGT CCIDVTS 2

TGGGGTGAGGAGGAGTTGCTG WGEEELL 1

TTGGATGAGCGGTGCACCTTG LDERCTL 1

GCTCCTTTTCGCTTCCGTTCG APFRFRS 1

TGCATTGGCAGCCCGAGGTAT CIGSPRY 1

TTCTTGTATCAGCAGTCCAGG FLYQQSR 1

TTCCGCTCTCGGGCGAATATT FRSRANI 1

GGTTACGTCGTGGTCGGTCGT GYVVVGR 1

TCGGTGGGCGGGCTTCCTGTC SVGGLPV 1

TTGGTGTACGGGGAGAGGCCT LVYGERP 1

TCGTGCGGTTGGTACACTACC SCGWYTT 1

CATCTGTTGTGCTGCTCGATC HLLCCSI 1

CACACGCGGAGGACCGCGCGT HTRRTAR 1

TGTCAGCGGCATTTTTGTCAG CQRHFCQ 1

GAGTGTGTGTGGGCGGGTTGT ECVWAGC 1

ATGGCCGTGGGCGACTTGGTG MAVGDLV 1

GGTGGGGTCCTGGGGACCTCT GGVLGTS 1

GACGGGAATAGGCTCGGGGCG DGNRLGA 1

GATCAGGACCGTCTCTATTAT DQDRLYY 1

CGGTGTGGGGGGCAGTTGAGT RCGGQLS 1

TGTAAGATCTCGGACACTCTT CKISDTL 2

GGTGTCTCGGTGCTCGTGTGC GVSVLVC 1

ACGAGTGCGGGGCCGTTTCGG TSAGPFR 1

CGGGCGTCGGGTGGGTGCCCT RASGGCP 1

TGCAAGGCCAGCAGGGTGTTG CKASRVL 1

GATTGGCTCGCGATCGGGTCT DWLAIGS 1

GTTGTCCGGTCGAGGCCGTGG VVRSRPW 1

TCTCATCACTGGTGTAAGCGG SHHWCKR 1

TGTCCTTTGAGGCTGATTTGG CPLRLIW 1

TGTTCCGCTACGCGCTACGGG CSATRYG 3

GTTATGCATCGCAGCCGTCCT VMHRSRP 1

GGTGCGCGCCTCGGTCAGATT GARLGQI 1

AGGATGGACACTGTCTGGAAT RMDTVWN 1

GCGAGGTCCTCGCGGATTCGG ARSSRIR 1

GCGGGTCAGGCGTTCGCCCTG AGQAFAL 1

GAGCTGCTGCGGTACAAGCCG ELLRYKP 1

TGCCATGGCAGCATGTCGAGT CHGSMSS 1

GTGCCGGAGATTCAGGGGATG VPEIQGM 1

CTCTGTTGGCTGTGGGTGAAG LCWLWVK 1

GTCTCGCGCGGCTTGAGCGGT VSRGLSG 1

TTTGGGGAGCGGTATGACTAT FGERYDY 1

CTGCGCGGTAGGCTTCTGTTC LRGRLLF 3

GCGTTGCGCGGGCCCTCGCCT ALRGPSP 1

GCGTCGTTCGCCCTTGGTGGC ASFALGG 1

CCGCCGCGGAGGAGTTATAGT PPRRSYS 1

CGGTTCCCTATGTATCTCTCG RFPMYLS 1

TGTCAGCAGTGCGGGGTGGTT CQQCGVV 1

GACATGGCTAGGCGGGGCAGT DMARRGS 1

TTCTATCACACGGTCGTTGGG FYHTVVG 1

TACACGCGTCACGGGGTTCTG YTRHGVL 1

AGCCGCAAGCAGGTCAGGGAG SRKQVRE 1

AGTGTCCGCGGTACCTTCACC SVRGTFT 1

CATTTCCGCGCTCTCACGGCG HFRALTA 1

GGCGACGTCAGGACGACCCGG GDVRTTR 1

CGGGGGTACATGTGGATTTCC RGYMWIS 1

GGCACCAACCCCTTTTGCATG GTNPFCM 1

CCGCGCACGGGCGGCCGCGTG PRTGGRV 1

TACCCCTATGCGAGTGGCGAC YPYASGD 1

CACGTGCCTGCCCCGTTCCTT HVPAPFL 1

TGTCTCCATCTGTGCTTGTCG CLHLCLS 1

AGCTTTCAGCGCCACAGTCGG SFQRHSR 1

CGCTTGGGGTTTGTGGAGTTC RLGFVEF 1

TGTTTCTGCGGGGACAGCATG CFCGDSM 1

CCTAGTCGCGGCACGGAGCGG PSRGTER 1

GCGCTCGCTAGGTCCAGTAAT ALARSSN 1

CGCGCGTGGAGGATTAGTCCT RAWRISP 1

GAGGGTCGCCTGGCTTGGTAT EGRLAWY 1

CGTGCGGCCGGTTATCACGCT RAAGYHA 1

ACCGGCCGCGCCAGGCTGTCG TGRARLS 2

GTCTACGGGCCGCGGTGTTTG VYGPRCL 1

ATTTGGGCGAGTGGGTGGCTT IWASGWL 1

CGGTTGGGCTTCGACTTCTGC RLGFDFC 2

GCGAGTGTGCAGTACGGGGCT ASVQYGA 1

GCCGGGCGGAAGTGGCCCTGC AGRKWPC 1

TTGCGGGTCGGTGACTACCAG LRVGDYQ 1

GGTTCCGGGCGCAACGGGGAG GSGRNGE 1

GTTATGTACACCTGGAGTGTG VMYTWSV 2

GTCCTTTCGCCCGTCGCGGGC VLSPVAG 1

CTCAGGTTTCGTAAGTGGTGG LRFRKWW 1

GGTCCGAGCCAGGCGGCTAGT GPSQAAS 1

ATCCAGTCTCTTGGCACCTGG IQSLGTW 1

GTTCCTTCCAGTAGTGCGTCT VPSSSAS 1

ATGCGTTGCTGCGGCTGGCCT MRCCGWP 1

GGGTTTTGTTTGGCGGGCTAT GFCLAGY 1

TTCCATGACTGCAGCGACGCG FHDCSDA 1

ACTGGGTTTGCGGGGATCTAT TGFAGIY 1

GATTTGCTCATTTGTTCTGTT DLLICSV 1

GTGGCGCCGCTTGGCTCTGCT VAPLGSA 1

AGGCTCGTGGAGATTGCGCGG RLVEIAR 1

CCTGATGTCCATCTGACGGGC PDVHLTG 1

GAGTGGTGTAGGCGGACGCCT EWCRRTP 1

CGTACGCGCATGGAGAGTGGT RTRMESG 1

TCTGTCGTCCGCCTGCGCGCT SVVRLRA 1

CTCAGGCTCGTCGGGAAGATT LRLVGKI 1

GCGTGGGAGGTGGGGAGGCCT AWEVGRP 1

TGGCGGCGGGGGGGGTATTAC WRRGGYY 1

ACGTCTAAGCGTGCTCTGTAT TSKRALY 2

TGCTATGTCCTTACTAGCGCT CYVLTSA 1

TGTCGGGTGTATTCGGGTCGC CRVYSGR 1

GTCAGCCGTAGCGCGACTGTT VSRSATV 1

CCGACGTACACGAGGCGCGGT PTYTRRG 2

GCCCGCTGTTGTTATGCTGTT ARCCYAV 1

GTCTACCTGTGGCCGCGTAGT VYLWPRS 1

AATAGGTTTTTCGTGGTCTGG NRFFVVW 1

GCGCGGTGTGTTTTTAACACT ARCVFNT 1

GTCAGTGTCGTCGATGGCGGC VSVVDGG 1

CGCGCTGGCTGGCGCGATGCT RAGWRDA 1

GCTTTCCGTTCTGTGGTGCGG AFRSVVR 1

AATTGGGATGGCGCGGGCGGT NWDGAGG 1

AATCATCGTGAGTCGGGTGTG NHRESGV 1

AGGCTCCAGGTGAACTATGCT RLQVNYA 1

CGGCGCAGCTGGACCTCGTCT RRSWTSS 1

GATGGTGAGGCGGACCGGTCC DGEADRS 1

TTGAATAGGCGGGTGTCGGGC LNRRVSG 1

GGTATTCATAGGGCGATGCGG GIHRAMR 1

TCTCAGATCATGGTCCGTTGT SQIMVRC 1

GCGCGCCAGGCGGGTGGGCCG ARQAGGP 1

TTCAGTATCGTGGTTACGGGC FSIVVTG 1

CGGTGCGTTACGGGGGGTTTG RCVTGGL 1

CGGCGGGCGCTCCGGTGGCCT RRALRWP 1

GCGGCTCATCCCAGTCTTACG AAHPSLT 1

GCGGCCGCTCTGTCTGTTGGG AAALSVG 1

GGGGCGCTGCGTCCCTCGCCT GALRPSP 1

AGGGCCCCGGTTCGGGGTTAT RAPVRGY 1

TTGCATTCGCGGCTCGGCAGT LHSRLGS 1

TCCAGTCTTTTGGAGGTCAGG SSLLEVR 1

TTGGACGTTAGCGGTTTTCCT LDVSGFP 1

GTCTCGCGGGTGCCGTCTAAT VSRVPSN 1

GGCGATTCCGGTCTGCTTGGT GDSGLLG 1

GAGGGGCCTCCTACTGCCCAG EGPPTAQ 2

GTCCGCGCCTCGACGATCGCG VRASTIA 1

CTGGTCCCTTTGCTTTCCCCT LVPLLSP 1

ACTCGGATGTGGCTTAGGCGG TRMWLRR 1

GTCCGTTCTGTCGAGATGAGG VRSVEMR 1

TTGAGGATGCCGTCTTGCCGC LRMPSCR 1

GATCTTTGTAGCGATGTTCGT DLCSDVR 1

GGCAGGAGCGGGCTGTCGCGG GRSGLSR 1

ATTAGCGAGTTGAATCCCGTC ISELNPV 1

ATGGGTGACAGCGGCGCTATT MGDSGAI 1

ATGTGGCGTGTGGTCCGGGCT MWRVVRA 1

CTGTCCTTCGTGGCTTTTTCT LSFVAFS 1

GCTCGTTTGGGCCCGGATGCT ARLGPDA 1

GTCAGGCTGTCCATGCATGCT VRLSMHA 2

GGGCCTATCACGGAGCGTTTT GPITERF 1

TTGGGCTGGTTGGGCACCAAG LGWLGTK 1

GGGGCGATCCTCGACGGCACG GAILDGT 1

CTTTGGTTGGGCACTATGTTG LWLGTML 1

GCTGAGAGGTCGCCTGCGATG AERSPAM 1

GGTGGGCATCTCCCCGCGCGT GGHLPAR 1

TCGTTCGATTGCGACACTGGG SFDCDTG 1

TGGCCTCGGAAGATTTTGCTT WPRKILL 1

TGTGGGGCGAGGTGGGTTAGG CGARWVR 1

TCTCACCTCCAGGCCTGTGAT SHLQACD 1

GACAGCGGCTCCATGAAGGAT DSGSMKD 1

CACGGTTACTTTTTCATGTGT HGYFFMC 1

TGGCGTGGCGAGGCCTCGCGT WRGEASR 1

TACGGGAGGTCGGTGGTTCCT YGRSVVP 1

TGCGATTACTCGAGGCTTTCT CDYSRLS 2

ACTCCTACTGGGGCGAGTTCT TPTGASS 1

TCCTTCCTCTTTCCTTCGCGC SFLFPSR 1

GCCATGTGTTGCTACAGTTGT AMCCYSC 1

TGGCGCTTCCGGCTGGTTCCT WRFRLVP 1

CCCCTGTGGGCGGATGTCCGT PLWADVR 1

GAGCGTCAGTGTGTGGTTCCT ERQCVVP 1

GTCCATAGGGTGATGGTCACC VHRVMVT 1

TTGGCTGCGTGGCGGGGTTCC LAAWRGS 1

CTGTGGTCTAGTGGTATGTTG LWSSGML 1

TGGCTGGTCCTGGCGGTTCTG WLVLAVL 1

TTCCCCATCTTTCTCGTGTCG FPIFLVS 1

GGGATGGCGAGCACGTCGGCG GMASTSA 1

GGCATGCCGAGGGATGATTGG GMPRDDW 1

CGCGAGGGGGGGGGTCCTCCG REGGGPP 1

CACGCGTTCTGTACCATTGCG HAFCTIA 1

GGGGCGCACAGGCAGCCGGCG GAHRQPA 1

GGGCGCATCGGGTGGCGGGCG GRIGWRA 1

AATGTGGTGTGGGCTCCTAGG NVVWAPR 1

CGGCATTTTCTCGTTTACCCT RHFLVYP 1

CACTCTTATGGGCGGGGGCCT HSYGRGP 1

TGCCTGTCGTGGATGTGCGGT CLSWMCG 1

TATGCTTACGGTGCGTTTCCT YAYGAFP 1

ATGGGCGATGGCAAGCACGTG MGDGKHV 1

CTTGCGTATCGCCCGGCGCCG LAYRPAP 1

TCGGAGTTCAGGACGTGTCGC SEFRTCR 1

TGGCTGGTCAGGCGGCATTGG WLVRRHW 1

ATGTCTGCGGATAGTTGTTAT MSADSCY 1

GCGGGCTACGGTGGTCTCTGT AGYGGLC 1

ATTCCGGGCGACGGCCGCACT IPGDGRT 1

CGGGCTGTCGTGGAGATTCCC RAVVEIP 1

TACGATGTGTCCGGCAGTCAT YDVSGSH 1

GTTAACCTCAGCGGTAAGTTG VNLSGKL 1

GCGGGGCGCTGCTTCAGCTGT AGRCFSC 1

CAGGCGCGCGGTGTGCGGCCC QARGVRP 1

TTCTGCGGGAGGATCACCTCG FCGRITS 1

CGTTGGGACAGCGGCGATTAC RWDSGDY 2

TGGCATCTGGACCTGTATGGG WHLDLYG 1

GATGCCTTGTCGCGTGGTTGT DALSRGC 1

CGGGAGGTTAGCGGGAGCAGG REVSGSR 1

GGCGTGTGGGCCCAGTTCCTG GVWAQFL 1

GTGCACCAGTTCAGGTCTATT VHQFRSI 1

CGCACTTTCATTTGGAGCGGT RTFIWSG 2

GCGAGCAGTCTGTCGGGCACG ASSLSGT 1

GTGGGCGTCGGCACGGTCTAT VGVGTVY 1

GTGTGTCACGGGGGCGTCGGT VCHGGVG 1

GCCAACCTTGCCCTTACCTAT ANLALTY 1

TTTGCTGCGAGTAGGCGGGAG FAASRRE 1

AAGCGGCTTCGGCCGACGGAC KRLRPTD 1

CGGGGTAGTCTTGGGGCGAGG RGSLGAR 1

TGGGCGCGCGATCGGAACTCG WARDRNS 1

CGGGCTGGGAGCGGTTGCGTT RAGSGCV 1

TGCTGTTCCTCTCGTTGCTCC CCSSRCS 1

GTGCCGAAGACGCACGGTTAT VPKTHGY 1

CGGGGGGCCTGGTGGTTCGGG RGAWWFG 1

TTGCTGTCGCCCAGGTCGAGT LLSPRSS 1

CATGACCCGGTGTTGGGTTTG HDPVLGL 1

TGTAACAGGGCTGACTGTTTC CNRADCF 1

CCGGCGATTCGGATTTTCGCT PAIRIFA 1

GCCAGGGTTCAGCAGATCAGC ARVQQIS 1

TTCTCTCGCAGGCTGTCGCGT FSRRLSR 1

GTGCGGTTGTTGTGGGAGACT VRLLWET 1

TCTGTTTTCGCTCTCATGTAT SVFALMY 1

GTCAGCAATTGGGCCTTGCTT VSNWALL 1

CAGTGGTGGCTTGGGTGCCAT QWWLGCH 1

TGTCTTGGCTCTCTCAAGCCT CLGSLKP 1

TCTCCGATCTGCAAGAGTGGG SPICKSG 1

GGGCCGTGCTCGAGGGGTGTT GPCSRGV 1

CTGGGTGACAGGTTCTGCCGT LGDRFCR 1

CACGTGGAGCAGTTCACTAGT HVEQFTS 1

GGGGTGGAGCCTTGGGCCTCT GVEPWAS 1

CGGAGGAGCTTTAGCTATTCT RRSFSYS 1

TGCCGGGCGGATCTTGTGACT CRADLVT 1

GCCTGTGGGGAGTATTGCAGT ACGEYCS 1

GCCCGGAGTCGCGTCTGCGGT ARSRVCG 1

GTTCTGGATCGTGGTCGGAAC VLDRGRN 1

TGTCAGCGCTCGTGTGCGCAG CQRSCAQ 1

ATCGGCACGCCCGCTGGCTCT IGTPAGS 2

GTTCTGCTGGATTGCCAGAGG VLLDCQR 1

CTGCGGATGCGGTCTGAGGCG LRMRSEA 1

GTGCCGGTGGGGGTGCCCGGT VPVGVPG 1

GGCGTCCGCGCTGTGTCTAAT GVRAVSN 1

TGCGAGAAGGCGCTCGGTCGG CEKALGR 1

TGTGGGTGTTGGATCGTGCTG CGCWIVL 1

GTCTCTGTGAGTACGGCGGGT VSVSTAG 1

ATTGCGGTTTCTTCTTTTACC IAVSSFT 1

TGGGGCCGCACTTTGATTCAC WGRTLIH 1

GGGCTGGTCAGTGTTCGTTGC GLVSVRC 1

AAGGCCGGGGTCGGGCGGCGG KAGVGRR 1

GTGCCTGACTTCTATTGTGGG VPDFYCG 1

GCCGGGGTGGTGGTCATTCTT AGVVVIL 2

GCGCGTGCGTGCGCCTTTCTG ARACAFL 1

TACGAGGTGCGCGGGCGGTTT YEVRGRF 2

TCTGTCCCCTATGGGGCGCGG SVPYGAR 1

TGTTATTGCCGGAGCCGCATC CYCRSRI 1

GGGCTCCTGACTTTCATTAGT GLLTFIS 1

GGGGCTGTGTGGGTGCACGAT GAVWVHD 1

TGGGATCCCGGGCTCGCGTGT WDPGLAC 1

TCTTTTACGCTTTGTGTGTGT SFTLCVC 1

CTGGGCGGCACGGGTCAGGTT LGGTGQV 1

GCCTTTGAGTTTCACGGGCGT AFEFHGR 1

AGGGCGTGGGAGTCGCATCGG RAWESHR 1

GCTCAGTTGCGGCGGGCGCCT AQLRRAP 1

AGGCCCCGCTGGGGCGAGCGT RPRWGER 1

GTGTTTATGGTCGTTTCTCGT VFMVVSR 1

ACGAAGGTGTCGGAGATTTTC TKVSEIF 1

CCTTGCCGTCAGGGGGCGTCT PCRQGAS 1

GGGCGTCAGAGGTTCCTTTTG GRQRFLL 1

ATGCACGATCGTATTCGGATG MHDRIRM 1

TGCCCGCACGGTCATGGTCAG CPHGHGQ 1

GTTGTCCTCAAGTCTGTGTAT VVLKSVY 1

AATCCGCTTCTCTGGGTGGCT NPLLWVA 1

CGGTTGGGCACCGGCTCGTGG RLGTGSW 1

CGCCGGGAGACGTCCGACCCT RRETSDP 1

GGGGCCCGCTGGGGCCCGAAC GARWGPN 1

CGTGGTGTGCTTTGGCCGCAG RGVLWPQ 1

TTCAGGGGCACGCGCGGGTCG FRGTRGS 1

CCTTTCCAGAAGCCCGGGCAT PFQKPGH 1

TGTTATCTTACGTACAGTGAG CYLTYSE 1

GATGAGCTTTCGTGCCAGTGT DELSCQC 2

CATTGGGTTGGTCGCTGGGCT HWVGRWA 1

CTGCAGACTGATGACTCGGAG LQTDDSE 1

TCTTTGCGGACCGGCGTTTGG SLRTGVW 1

TGTAGCTGCGACCTGCTTGGT CSCDLLG 1

GCGCTGAGCGATGTCTACCAC ALSDVYH 1

TGGTTGGTTCGCGGCCCGATG WLVRGPM 2

AGCCGTGCGCTGCCGGACCTT SRALPDL 1

AATGTGCCGCATGGGGTCCCG NVPHGVP 1

AGGTGTTCCTGGTGGCCTCCG RCSWWPP 1

TGCTCTGGCGTCGTGTCCTCT CSGVVSS 1

GAGCTCATCCGTGCCGCTGAT ELIRAAD 1

GAGGAGGGCGAGCGCTCTTAC EEGERSY 1

TATTCTGCGAGCCGGGTTGGT YSASRVG 1

CCGCTGCCGAGGGGCGTCTCT PLPRGVS 1

TGCAGCGGGGTGTGGGTTAGT CSGVWVS 2

TCCTGGCGCGAGCCGGCGCGG SWREPAR 1

AGCGTCTGTCTCCCTGGCCAG SVCLPGQ 1

CGGCTGGGCAGGGACCCTTGG RLGRDPW 1

GTTGATCTCCATGTCTTTGTT VDLHVFV 1

TGCTCGGAGGCGTTGAGCAAT CSEALSN 1

AGGGTCCAGAGGGGTGGTTTG RVQRGGL 1

TTTTGGATCAGGCTCATGCAT FWIRLMH 1

TACACGCTGACCGGCGGTCAT YTLTGGH 1

GGCTGCGATAGGCGTCGCTCG GCDRRRS 1

TGCGGGCGCTATGCCGCGATT CGRYAAI 1

AGTGATTACCGTGGCTCCAGG SDYRGSR 1

TCTCGCTGCACTGGCTGTGCG SRCTGCA 1

GCGGCGGCGTTTAACCGGCAG AAAFNRQ 1

GCGCTTAGCGGCGGTATCTCT ALSGGIS 2

GTCACCGGGCGCTGGTGGACG VTGRWWT 2

TCGAGCTCGCTGCGCGGCGGC SSSLRGG 1

GCCATTATTTCTTTCTGGGCG AIISFWA 1

AGCGAGTTGGCGTTCAGCATT SELAFSI 1

GGTTTGACTCTGGTGCGCTGG GLTLVRW 1

TCGATGGACCTGTATAGTCCT SMDLYSP 1

AGGGGCGCGTCCTCGGTCTGT RGASSVC 1

TCGGTCCTCGTTGTTCGGAAG SVLVVRK 1

GGCTGCAGCAGGAAGGTCTGT GCSRKVC 1

TGCCACCGGGCTGTGAGTCGG CHRAVSR 1

TTCCGGGGGCGCCTTGGGGTT FRGRLGV 1

TTCTATGGCTATGGGTGTCGG FYGYGCR 1

ATGGGCCGCATCAGCAGCTCC MGRISSS 1

ACTGGGTTTAGTCTCGTTACT TGFSLVT 1

GGGTGGCGTTATTTGAGCACT GWRYLST 1

ACTAGTTTGGTGGACTTTCGG TSLVDFR 1

AGGCGGGAGGGCGTTCTTGTT RREGVLV 1

GCTGCGGGCGCCGTCGGGCTT AAGAVGL 1

ATTGCGCTGCTCCGCCGCTGC IALLRRC 1

ATTGAGACCTTCGAGAGGGTG IETFERV 1

CCCGGGGTCGGTCCCTATCAC PGVGPYH 1

TACTTTTACGGTGAGGCTGTC YFYGEAV 1

TACGTCCAGCGGGTTCGCTAT YVQRVRY 1

GGTGGCGGCTGCCTTTTGGAT GGGCLLD 1

CATCGGTGGAAGGTTTGTAGG HRWKVCR 2

GTGGCCGGGCGTTTGATGGCG VAGRLMA 2

CTCTGCGGGGTGAGCACGGTT LCGVSTV 1

CTTGATGCCACCGGTAAGGGT LDATGKG 1

GGGCAGGTGAGGCGCGGCGCT GQVRRGA 1

TCTTCTTGGCGCGAGCTGCAT SSWRELH 1

TTCTGGCGCTGGCGCAAGATG FWRWRKM 1

TGGGACTGGCGCCCTGTGGAG WDWRPVE 1

GCTGATGCTGGGGAGGCGGAG ADAGEAE 1

GCGTTGGTTTGGCTGCACCGG ALVWLHR 2

GCGACCGCTAGCGGTAGTGTC ATASGSV 1

CTGGTTATTCCCATCCTGCTC LVIPILL 1

GTGGAGGTGGCCGTCAGCTCT VEVAVSS 1

GGCGGCGTGGCGTTCCCGTAT GGVAFPY 1

CCTCGTCTTAGTGGCCTGTCT PRLSGLS 1

TCGAGCAGGGAGGCTGATATT SSREADI 1

CGCTCTTACTCTGGGAAGTAT RSYSGKY 1

ACTTTTGCGATCAGTCCGTCT TFAISPS 1

CAGCTCTTCTGGCTTGCCACG QLFWLAT 1

GTGGATCGGTGCCCTGTCACT VDRCPVT 3

GGGGCGGCTATCGTTAGTAGC GAAIVSS 1

AAGTACCACGCGATCCGTCGG KYHAIRR 2

GCGCTGGAGCGGGCTTGGTCT ALERAWS 1

GGCCTGCTTGCTTGGTGCAGT GLLAWCS 1

ACCATGGGGTGTGCGTCTCGT TMGCASR 1

TCGACGGCCATTTTTCCGGCG STAIFPA 1

GCTCCGGCGCACTCGGGGACT APAHSGT 1

TTTCGCTTCATGTCTGCCGTT FRFMSAV 1

GAGGTCCTGCACAAGGGGTAT EVLHKGY 1

TGTCATGGCCGCATGGGCCCG CHGRMGP 2

TTCAGTAGCAGCGGTAGGGCG FSSSGRA 1

TTGGTTCAGATCCTTATGCGG LVQILMR 1

GGTGACGAGGTGGGGCGGCGG GDEVGRR 1

CATCTTTCGAGTCTGTCGACG HLSSLST 1

GAGTGGCGCGCTTCCGCTCTG EWRASAL 1

CACGACCTGTTGGGGCAGTGG HDLLGQW 1

GTGTTTGTGTTGGCGATGCCC VFVLAMP 1

TCCGCGTGTGGTTGGCGCGTG SACGWRV 1

GGTGGCTTGTCTGGTCTGCCT GGLSGLP 1

AGTCCTCGCGAGGGGCCGATT SPREGPI 1

GCTTGGGCGCTTCAGGGGGAT AWALQGD 1

GGGAAGCGTGCGAGGTTGACT GKRARLT 1

GCTGACCATGGCAGGGGTGTT ADHGRGV 1

CAGCTCAGCAGGAAGCGCGGT QLSRKRG 1

ACGTGCCACTGGTGCACGTCT TCHWCTS 1

GCGAGCGTCCGCGCTCCGAAT ASVRAPN 1

GGCTCGGGCCGCGACTCGCGT GSGRDSR 1

CGGCGCTGGGCGGTCGGCGGT RRWAVGG 1

CCTGAGGTGAGCTGGGTGCTT PEVSWVL 1

GATGGGCCTGCGAGCTATGTT DGPASYV 2

AGTTGGTGGTGGGTCATCATT SWWWVII 1

CCTCTCCGGGGCACTGATCCT PLRGTDP 2

ATCGGCTGTGGCCTGTCCGTT IGCGLSV 1

CCTATCGATTTCTGGCGCGCG PIDFWRA 1

CGTTGTAAGTACTGGTCGTTT RCKYWSF 1

TGCCTGACGTATGGGGCTCTG CLTYGAL 1

CGCGGGTCGAGCGGGATTTGT RGSSGIC 2

ATCAGGGTGCGGTGCACCTGG IRVRCTW 1

GCGTACGTCTCGCGTGGGCCG AYVSRGP 1

TGCCGTCGTGCGGTCATTGCT CRRAVIA 1

TCGCCGAATTCGGGCGCCATT SPNSGAI 1

CGGAGGAAGGCGTGCTGTACG RRKACCT 1

GTGTATATGCTTGGCTGGAGG VYMLGWR 1

CGCTGGCGCACCATTTTCTCG RWRTIFS 1

TTGACGGGGGCGCGCCTTCTG LTGARLL 1

ACCAGGTGGGTGCCGGTTTCT TRWVPVS 2

AGGCTGCCGCGGTTGAGGCAT RLPRLRH 1

TTTCATGCCGCTCTCGTGTGT FHAALVC 2

CGGATGTCCATTTCGCGCCAT RMSISRH 1

CGGCTCACCAGTCCTGAGGGT RLTSPEG 3

TCGATCGTGTTTAATAATGAG SIVFNNE 1

TCGGAGGTTCAGCGTAGTCCT SEVQRSP 3

TTCGGCTCTACGTTCTTTGGT FGSTFFG 1

GGTATTAAGAGTGTGTGTAGG GIKSVCR 1

GGCATGCTCATGCCCTTTGGG GMLMPFG 2

TGGCAGCCCTCTGGCTGGTAT WQPSGWY 1

TTGTGGCGGTGGGTGGTGGCT LWRWVVA 1

ATCACTTTCGGTACGTTGTGT ITFGTLC 1

GCCTTTCGGTTCCCCGAGGCT AFRFPEA 1

GCCTTCAATGGCTGGCTTCAT AFNGWLH 1

CCGTACCCTTCTGGCAGTTCG PYPSGSS 1

CGCCGTGGGATGGGTGCTCGT RRGMGAR 1

CCCCGCGCCCAGGTGTGTGCT PRAQVCA 1

GCTGTTCCGGTGCCTACGCTT AVPVPTL 1

GGGTACAGCCACGCGTGCGTT GYSHACV 1

CGGAGTGCGGCCTCTGACGTT RSAASDV 1

GACAGGGCGCGTGCGCTTGTT DRARALV 1

AGTTCGTCTCAGGGTCCTGCT SSSQGPA 1

GAGGAGGGCGCGCCCTCTTGT EEGAPSC 1

GGTCTTCGTGTTGGGTCGATG GLRVGSM 1

CCCGAGCAGGACTGGTGTCGC PEQDWCR 1

CGTGTGAGGTTGGGCGGCCGG RVRLGGR 1

CCGGAGAGCTGTATCGTTCCC PESCIVP 2

AGGTCCCAGGCGCTCGCGCGC RSQALAR 2

GTTCAGCGCGATAGGGCTCCT VQRDRAP 1

TTCCAGCCCTGCGGCGTTGAT FQPCGVD 1

CGGGTCTGGTTGACGCAGCAC RVWLTQH 1

GGGGTGGGGACGTGCGGTACC GVGTCGT 1

TGGCGGTTTATGGGTGTGAGG WRFMGVR 1

AGTCTCCCGAGCGTGGCTTCT SLPSVAS 2

CCCCGGCATTCGGCGTTGATC PRHSALI 1

CTTCCGTTGAGGGTGAACAGT LPLRVNS 3

GGCTCTCACTGGGCTGTGCGG GSHWAVR 1

GCTGCGGCTGACTATGCTCGT AAADYAR 1

AGGTGGTCCCGTCTCGTCGTT RWSRLVV 1

TTTACGCTTAATTGCTGGTGT FTLNCWC 1

CTCGCGACGAGCTATCATTTG LATSYHL 1

GTTGAGCACTGGCGCATGGCG VEHWRMA 1

TGGACCTCGCGTAAGAAGGGT WTSRKKG 2

ACTACGCGGAGGGCCCGGCCT TTRRARP 1

GTCTGGCTGGCGCAGTGGCCT VWLAQWP 1

GCGTCCGCCATCTACCGTAGT ASAIYRS 1

CAGCGGCGGGGTGCGCTGTTT QRRGALF 1

GCCCGGGACAGCTGCGGGCCT ARDSCGP 1

GCGGGGTACTCCTGGCGGGCG AGYSWRA 1

GACTGTGTCAGGCGCTGTGTG DCVRRCV 1

TGCTTTTACAGGGAGGGGGTT CFYREGV 1

TGGTTTCTCGTTGTTGGGACT WFLVVGT 1

GGTGCCTGTGTCGTGCGCGAT GACVVRD 1

CACTGGATGGTGGGCCAGCGT HWMVGQR 1

GGTGCCCCGCCTTTCTCTAAG GAPPFSK 1

GAGCGCCGGCCTGACGCGAGG ERRPDAR 1

GAGCGGGTTGCCTTGTGTAGG ERVALCR 1

AATTCGTCGTTGTCCACGGCT NSSLSTA 1

GACGACGTTGATTGTGGGCCG DDVDCGP 1

ACGCAGACCTGGACTATGGGG TQTWTMG 1

CTGCATCTCAGCGCGTTTGTC LHLSAFV 1

GGGAACCTTACTGTCTCGCAG GNLTVSQ 1

CTTTGGTGGACCGCGTTGTGT LWWTALC 1

TCCCGGGCGCGTCCGGTGGCT SRARPVA 1

ACTTATCTGACTGCGAACCGT TYLTANR 1

TTGATCCTCAGGATCCTGGGG LILRILG 1

GCGGCTATTTCCAGTTGTATT AAISSCI 1

AGTCTTCCGGTTGAGTCGGGT SLPVESG 1

GCTACCTGTTGTCAGTCCATG ATCCQSM 1

ACGGTCGTCCCCTACGGGGTG TVVPYGV 1

ATGTCTCGGATTCCGGGCTGT MSRIPGC 1

GTGGTCAGGAGTAAGCGTTCG VVRSKRS 1

GGTCTCGGCTGGGACGTTTAT GLGWDVY 1

CGTCCGGCGGGCATGGGCCTG RPAGMGL 2

GCTGACAAGAGCGCGGGCTTG ADKSAGL 2

TACCCGGCCTGGGTGTACCAG YPAWVYQ 1

AGGCAGGTGCGGGAGCGGATG RQVRERM 1

TGCCGTCCCTTCTTCTGGCCT CRPFFWP 1

TCCGCTAGCTGGCAGTCTCAT SASWQSH 1

GAGATGGTGTTGCCTTCGTCT EMVLPSS 1

CGCGATGATATGTCGTCTATT RDDMSSI 1

TCGCTTCGGCTGCGGCCGTAC SLRLRPY 1

TGGGATCTGCAGGAGGTCCGC WDLQEVR 1

ACTCTGAGCTTTATTTACGCT TLSFIYA 1

TGGATTTGGAGCGGGGTTCTG WIWSGVL 1

TCGTGCGCGTCTAGGCGCTAT SCASRRY 1

AGGCCTGTGGCTACCTCTCTG RPVATSL 1

ACCCTGCGGTCGTGTCGCTGT TLRSCRC 1

GGTCCTCACAGCGCGAGGGGG GPHSARG 1

TCTTTGACTCATCTGTGCCAT SLTHLCH 1

GCGTACCTTATTATTTGCAGT AYLIICS 1

TGGGACCTTGATGGTTACCGG WDLDGYR 1

GCGGCTCGCAGGGCCCCGCTG AARRAPL 1

GATGTGTTCGCGCCCGATGCG DVFAPDA 1

TTTTGCGGCTTGGAGTGCACT FCGLECT 1

CGGCCGGGCCGCCGTGTTGGT RPGRRVG 1

CGCATCGTCACTCGCGGTCAT RIVTRGH 2

TGGCCCGGGGCTAGCGTTGGT WPGASVG 1

CATCAGCGCTGTATCGGTGCT HQRCIGA 1

TGGTACGTCTTTCGTTGGGCT WYVFRWA 1

GGGCTGGGGTGTCTGATGTGT GLGCLMC 1

GGCTGTCGTCACCAGCTCGTT GCRHQLV 2

TATGCCGGCAAGACGAGTTGG YAGKTSW 1

GATCGGTCCGGCTTCGGTTGG DRSGFGW 1

CGGCAGGCTGATAGGGGCAGG RQADRGR 1

GGTGAGGGCGGTTCTGCTGTG GEGGSAV 1

ACTTGTTGCGGGAGCGCGGGT TCCGSAG 1

GGTGCGGACCTTTCTGCCCCG GADLSAP 1

GCGCAGGATCGGTACCTCTGT AQDRYLC 1

CGGGGGGGGAGCTACGACGAG RGGSYDE 1

GGGGGTTACCTCCTTGATGCG GGYLLDA 1

TTGCTGTCCTGGGAGGTTTCT LLSWEVS 1

CCGCACAGCGTGTTGAACCGC PHSVLNR 1

TTGGATGTTCCGGCCGGTTTC LDVPAGF 1

TGTACGGTCATGGTTTTGTCC CTVMVLS 1

GGGTTCTGGCCGGTCGCTGGT GFWPVAG 1

ACGTGGGTGCAGCGTGTGCAT TWVQRVH 1

GTGTTGGCTAGGGGTTTTGGG VLARGFG 1

GTTGGCTCCCGGTACTCGCGG VGSRYSR 1

GGTACTCTTGGTGGCGCCTGT GTLGGAC 1

GGTGGGAGGGCGCGTGACGGT GGRARDG 1

TTGAAGGTGAACAACCGTTGG LKVNNRW 1

TTGCGCAGTAGTGTCTCTGCG LRSSVSA 1

GCCCAGTGTTGCGGCTCCCCG AQCCGSP 1

GTCGATACTCCTAAGGGGGCT VDTPKGA 1

GGTCTTGTTAGTATGAGGCCT GLVSMRP 1

TGTCTCCCGAGTCTGGCCATC CLPSLAI 1

CAGTCCAAGCGTGTGACGAGT QSKRVTS 1

ATGCCGCAGGACGGCCCGTGT MPQDGPC 1

GCGGTGGTCGGGAACCAGCCG AVVGNQP 2

GTCTGGAGCTTGACCTGTTCG VWSLTCS 1

GTGTCTAGGATCTTGTCGCGT VSRILSR 1

CTGTGTCTGGAGGTCTCTAGT LCLEVSS 1

CCGAATCCGAGCAGGGACCAT PNPSRDH 1

CCCCGGGTTTTCGATGTTACT PRVFDVT 1

GTGCACTGCGTGGGCTTTGTG VHCVGFV 1

GTGGTCCGGGGGCATGTGGGG VVRGHVG 1

CGCAGGCATCCGACCTGTCGT RRHPTCR 1

GGGTACGGGCCGCACTGCCCC GYGPHCP 1

CAGTCTGACTGTTTGCCGTGG QSDCLPW 2

CTGGACCACGGGTATTCCTTT LDHGYSF 1

CCGTCGGGGTTGAACGCGATT PSGLNAI 1

TTCGGGTCCTCGAAGTTGGCG FGSSKLA 1

AGCCTCGCGGACTGGGGTGTT SLADWGV 1

GCTTGCCGTCCGAAGAGTGGC ACRPKSG 1

CATGAGGAGGTGACTCGGGTT HEEVTRV 1

CTCTGGTTCGGCGGCGCGGTT LWFGGAV 1

CGGGGTTATAACGTTAAGTGC RGYNVKC 1

TTGGTTAGGGGCGACGATCTG LVRGDDL 1

TGGGTTACTTTCGATCTTTGT WVTFDLC 1

GTCCCCTGGTTTAATGCGTTG VPWFNAL 1

GTCGCTTGGGTGGTGTTTTGT VAWVVFC 1

TTCGAGGACCCGCTTTACCTT FEDPLYL 1

AGCTTCCAGTACTGTGTCAGG SFQYCVR 1

CGGGGTGTTCTGTTCGGCGTT RGVLFGV 1

GTCGAGATTGCGTTGGGTCCT VEIALGP 1

CTGCGGGTCAGTGGCCCGGCG LRVSGPA 1

TGCACTGCCGCGGGTTGGCAT CTAAGWH 2

TGGGCGCTGATTGATCGGACT WALIDRT 1

CGCTTCGGCTGCGCCGTTTTT RFGCAVF 1

TCTGCGGGCGGCGACCGGAAT SAGGDRN 1

GTGTCTCTCACGACTGCCGTG VSLTTAV 5

AGTCGGTCGGGCGCCTTGGCT SRSGALA 1

GCGGCTACGGGGCCCATTGGC AATGPIG 1

GTTCGGTACTTTAGCTATGGG VRYFSYG 1

TTGGATGACTCGACCGGTGGG LDDSTGG 1

TTTCGTAACCAGGTCGGTAGG FRNQVGR 1

ATGCTGGTCGATGGTGCTCCT MLVDGAP 1

GTTAAGTCGAACTGCAGTTGG VKSNCSW 1

CACACTATTAAGGGCCCGAGG HTIKGPR 1

ACGTATCTGTGTAGGTTCCCC TYLCRFP 1

TGGGAGGTGCCCGACATCGCG WEVPDIA 1

GTTGGGGCTCCTGCTCCTGCG VGAPAPA 1

GGTTTGACTTCTAGCACGCAG GLTSSTQ 1

TGCAACCTCAGGAAGGACACT CNLRKDT 1

GCGCACCTGGGGGTCGCGCTG AHLGVAL 2

TTGGCGCGCGGGCAGCTCTGG LARGQLW 1

GCTCCGCGTCCGTACCGGCGG APRPYRR 1

TTGCGGGGGTCCGGGGCTGAG LRGSGAE 1

CTTACGCACAAGGCTGAGCGT LTHKAER 1

GGGAAGGCGAACGGCGCGTCG GKANGAS 1
[truncated: 1,264,748 more chars]
